# Function Analysis of the ERF and DREB Subfamilies in Tomato Fruit Development and Ripening

**DOI:** 10.3389/fpls.2022.849048

**Published:** 2022-03-04

**Authors:** Li Zhang, LiJing Chen, ShengQun Pang, Qun Zheng, ShaoWen Quan, YuFeng Liu, Tao Xu, YuDong Liu, MingFang Qi

**Affiliations:** ^1^College of Agriculture, Shihezi University, Shihezi, China; ^2^College of Bioscience and Biotechnology, Shenyang Agricultural University, Shenyang, China; ^3^College of Horticulture, Shenyang Agricultural University, Shenyang, China; ^4^Key Laboratory of Special Fruits and Vegetables Cultivation Physiology and Germplasm Resources Utilization Xinjiang of Production and Construction Crops, Shihezi University, Shihezi, China; ^5^Key Laboratory of Agricultural Biotechnology of Liaoning Province, Shenyang Agricultural University, Shenyang, China

**Keywords:** AP2/ERF, tomato (*Solanum lycopersicum*), ERF, DREB, DRE/CRT, GCC box, EAR motif, yeast one-hybrid

## Abstract

APETALA2/ethylene responsive factors (AP2/ERF) are unique regulators in the plant kingdom and are involved in the whole life activity processes such as development, ripening, and biotic and abiotic stresses. In tomato (*Solanum lycopersicum*), there are 140 AP2/ERF genes; however, their functionality remains poorly understood. In this work, the 14th and 19th amino acid differences in the AP2 domain were used to distinguish DREB and ERF subfamily members. Even when the AP2 domain of 68 ERF proteins from 20 plant species and motifs in tomato DREB and ERF proteins were compared, the binding ability of DREB and ERF proteins with DRE/CRT and/or GCC boxes remained unknown. During fruit development and ripening, the expressions of 13 DREB and 19 ERF subfamily genes showed some regular changes, and the promoters of most genes had ARF, DRE/CRT, and/or GCC boxes. This suggests that these genes directly or indirectly respond to IAA and/or ethylene (ET) signals during fruit development and ripening. Moreover, some of these may feedback regulate IAA or ET biosynthesis. In addition, 16 EAR motif-containing ERF genes in tomato were expressed in many organs and their total transcripts per million (TPM) values exceeded those of other ERF genes in most organs. To determine whether the EAR motif in EAR motif-containing ERF proteins has repression function, their EAR motifs were retained or deleted in a yeast one-hybrid (YIH) assay. The results indicate that most of EAR motif-containing ERF proteins lost repression activity after deleting the EAR motif. Moreover, some of these were expressed during ripening. Thus, these EAR motif-containing ERF proteins play vital roles in balancing the regulatory functions of other ERF proteins by completing the DRE/CRT and/or GCC box sites of target genes to ensure normal growth and development in tomato.

## Introduction

Plant hormones are involved in vital processes of complex signal transduction pathways and affect the expression of various genes at different time periods and in different organs, which regulate plant growth, development, and defense responses. To ensure survival and reproduction, diverse hormones, such as auxin (IAA), abscisic acid (ABA), ethylene (ET), gibberellin (GA), cytokinin (CTK), and jasmonate (JA), are synthetized and regulate different life activities in their metabolic networks (Davies, 1987; Pieterse et al., 2009). In these complex networks, transcription factors (TFs) are critical regulators that play essential roles (Yamasaki et al., 2013).

Among these TFs, AP2/ERF is widely distributed in the plant kingdom and plays important roles in regulating growth and development (Chen H. C. et al., 2021; Guo et al., 2021; Jia et al., 2021). With updates to the plant genome database, more AP2/ERF genes have been identified. Thus far, 147, 291, 170, 163, and 136 AP2/ERF genes have been found in *Arabidopsis thaliana* (*A. thaliana*) (Dietz et al., 2010), *Chinese cabbage* (Song et al., 2013), *Salvia miltiorrhiza* (Ji et al., 2016), rice (Akhter Most et al., 2011), and melon (Ma et al., 2015). Currently, RNA sequencing of many species has been conducting, laying an important foundation for the study of AP2/ERF gene families during plant growth and development. *Jatropha curcas* L. *JcERF035* was identified in the roots and leaves under Pi deficiency conditions by RNA sequencing and its overexpression affected root development in *A. thaliana* (Chen et al., 2018).

AP2/ERF family members have been divided into 5 groups according to *A. thaliana* classifications: ERF, DREB, AP2, RAVs, and soloist (Nakano et al., 2006; Dietz et al., 2010). These subfamilies exhibit different structural characteristics. Among them, differences between ERF and DREB subfamily members is that the 14^th^ and 19^th^ amino acids of the DREB proteins are valine (V) and glutamate (E) in the AP2 domain, but alanine (A) and aspartate (D) in ERF proteins. This difference affects the ability of proteins to interact with DRE or GCC boxes during the regulation of their downstream target genes during transcription (Sakuma et al., 2002). It also suggests that ERF and DREB subfamily members may act in different regulatory pathways. For example, *PUCHI*, an ERF subfamily gene, regulates lateral root development, floral meristem identity, and organ initiation in *A. thaliana* (Hirota et al., 2007; Chandler and Werr, 2017). *A. thaliana DREB2A* overexpression enhanced drought and heat tolerance in transgenic plants (Sakuma et al., 2006a,b). Additionally, DREB2A affected leaf senescence by interacting with radical-induced cell death 1 (RCD1) under heat stress (Vainonen et al., 2012). Thus, an ERF protein is often involved in several regulatory networks, which causes some ERF proteins to exert the same or opposite function during different processes. AtERF1 activates the defense-related gene, *PDF1.2* (Berrocal-Lobo et al., 2002), while AtERF4 represses *PDF1.2* in biotic stress tolerance (McGrath et al., 2005). Moreover, AtERF2 and AtERF5 are activators and AtERF3 is a repressor and they regulate downstream target genes during transcription in defense responses (Fujimoto et al., 2000).

Although different ERFs may exhibit opposing functions in different vital processes, an ERF may function in several of these processes. For example, *AtDREB1A* in transgenic *A. thaliana* plants resulted in the dwarfed phenotypes and freezing and dehydration tolerance, whereas *AtDREB2A* transgenic plants exhibited slight growth retardation (Liu et al., 1998). Additionally, wild-type *A. thaliana* plants overexpressing *AtERF53* exhibited unstable drought tolerance, while *rglg1rglg2* double mutant plants overexpressing *AtERF53* exhibited stable drought tolerance, as RGLG1 and RGLG2 together negatively regulate *AtERF53* transcription (Cheng et al., 2012). However, *AtERF7* overexpression decreased the sensitivity of guard cells to ABA and increased water loss during transpiration, which reduced drought tolerance in transgenic plants. Contrasting results were found in *AtERF7* RNA interference plants (Song et al., 2005). These studies suggest that although the regulatory pathways of DREB and ERF proteins differ, they can achieve the same effects in different vital processes.

*Solanum lycopersicum* (tomato), as an important fruit vegetable, is widely planted in many countries. Tomato fruit is highly nutritious and has a unique flavor, and can be eaten raw, boiled, or processed into ketchup or juice. Thus, improving the fruit yield and quality of tomato is the primary goal of tomato production. To achieve this goal, our understanding of the underlying molecular mechanisms of different vital processes must be enhanced, including seed germination, fruit ripening and softening, flower development, and defense responses to biotic and abiotic stresses. Among these processes, ERFs as regulators or repressors play important roles that affect different gene networks. In this study, we identified, corrected, and analyzed all ERF and DREB subfamily members based on *S. lycopersicum* genome database versions 2.0, 3.2, and 4.0. To understand the functions of ERF and DREB subfamily members, several RNA sequencing databases from NCBI SRA data library were used to analyze gene expression levels during the tomato growth and development. In addition, the inhibitory function of the EAR motif in several ERF proteins was tested by yeast one-hybrid assay, and gene expression profiles were analyzed by qRT-PCR during fruit ripening. These results will help establish the regulatory networks of ERF and DREB subfamilies, and uncover effective ways to improve tomato yield and quality.

## Materials and Methods

### Plant Materials and Growth Conditions

The tomato (*S. lycopersicum*) ‘AC’ cultivar was grown in a greenhouse for 22 ∼ 25°C 16 h light (150 μEm^–2^s^–1^)/16 ∼ 18°C 8 h dark. Three samples of the green pulp and color-breaking pulp for 1, 3, 6, and 9 days were collected and utilized for gene expression analysis. The fresh young leaves, flowers, or shoot tips of tomato plants were gathered, frozen in liquid nitrogen, and utilized to clone the CDS of target genes.

### Identification of ERF and DREB Subfamily Members in Tomato

The genome sequences of *S. lycopersicum* were downloaded from a database of gene annotations, SGN, (Release v2.0, v3.2, and v4.0^1^). The hidden Markov model (HMM) profile of the AP2 domain (PF00847) was downloaded from the Pfam database^2^. HMMER v3.3 was used to search for candidate AP2/ERF genes from the tomato genome database. The default parameters were used and the cutoff value was set to 0.001. All of the candidate AP2/ERF proteins with only a single AP2 domain were selected as candidate ERF proteins. The Pfam, SMART^3^, and NCBI CDD databases^4^ were used to validate the candidate ERF proteins. Finally, the identification results of the 3 genome versions (2.0, 3.2, and 4.0) and NCBI database were compared to determine the final ERF subfamily members of *S. lycopersicum*.

### Phylogenetic Analysis

Multiple sequence alignments of the tomato ERF proteins were performed using CLUSTAL W based on the complete sequences. To understand the relationship among the tomato ERF proteins, a phylogenetic tree was inferred using the maximum likelihood method based on the Whelan and Goldman model (Whelan and Goldman, 2001) of MEGA v7.0 with the following parameters: JTT + G model, partial deletion with 80% site coverage cutoff, and 1,000 bootstrap replications (Kumar et al., 2016).

### Gene Structure and Conserved Motif Analyses

According to the cluster analysis results of the tomato ERF gene subfamily, the structural domain analysis of the ERF protein sequences of different groups was conducted using Jalview software (Waterhouse et al., 2009). Homologous alignments were compared using T-Coffee software (Notredame et al., 2000). The protein sequences of non-conservative regions were deleted. The alignment results were preserved in EPS format. Conserved motifs of the tomato ERF subfamily proteins were identified using the Multiple Em for Motif Elicitation (MEME) online tool v5.1.1^5^ with the following parameters: number of occurrences of a single motif distributed among the sequences within the model, 0 or 1 per sequence; maximum number of motifs, 20; optimum width of each motif, 6–50 residues.

### Transcriptome Data Source and Bioinformation Analysis

Transcriptome sequencing data were downloaded from the NCBI SRA database^6^ using the SRA toolkit. The project number of transcriptome data used in this article is as follows: PRJNA507622 (*S. lycopersicum*, 30 tomato organs) (Penin et al., 2019) and PRJNA528656 (*S. lycopersicum*, fruit). Every organ includes three biological repetitions in these data.

The transcripts per million (TPM) expression values of the transcriptomes of different organs in tomato were obtained using the SRA toolkit and Salmon software (Patro et al., 2017). Subsequently, the TPM values were processed to quantify of gene expression levels of the original data. The expression heat map of the ERF genes in different organs was drawn using R-pheatmap based on the TPM values.

### RNA Isolation and cDNA Synthesis

Total RNA was extracted using the TaKaRa MiniBEST Universal RNA Extraction Kit (TaKaRa, Kyoto, Japan). First-strand cDNA was synthesized using the PrimeScript*™* IV 1st strand cDNA Synthesis Mix (TaKaRa, Kyoto, Japan). The first-strand cDNA was utilized in the expression and amplification of *SlERF* genes.

### Quantitative Real-Time PCR Assay

qRT-PCR was used to analyze the expression of 14 *SlERF* genes during tomato fruit ripening. cDNA was used as a template with the primer pairs shown in Supplementary Table 1. The *Sl-Actin* gene was used as internal control. The reactions were performed in triplicate for each sample using the TB Green^®^ Premix Ex Taq*™* (Tli RNaseH Plus), Bulk (TaKaRa, Kyoto, Japan) on an qRT-PCR system under the following conditions: 95°C for 30 s, 40 cycles of 95°C for 5 s and 60°C for 30 s, and 1 cycle of 95°C for 15 s, 60°C for 1 min, 95°C for 15 s, and 60°C for 15 s. The dissociation curve was used to validate the specificity of each primer pair. Each experiment was repeated three times. The relative expression level of each gene was calculated and each result was reported as mean (±SE) of three independent experiments. ANOVA was used to identify statistically significant difference among genes (*P* < 0.05).

### Yeast One-Hybrid Assay

To explore whether EAR motif-containing SlERF proteins played a repression role, 14 EAR motif-containing *SlERF* genes were selected to construct Y1H vectors by Matchmaker Gold Yeast One-Hybrid System Kit (Clontech, Mountain View, CA, United States). First, the pAbAi vector was cut by *Sal*I restriction enzyme, 3× DRE and 3× GCC elements were, respectively, inserted into the linear pAbAi vector, and then the recombinant vectors were transformed into Y1H gold yeast competent cells (primers in Supplementary Table 1). The yeast cells were selected on a plate without uracil for selective glucose synthesis and the positive yeast colonies with 3× DRE or 3× GCC elements were identified by the colony PCR analysis (Matchmaker Insert Check PCR Mix 1, Clontech, Mountain View, CA, United States). The yeast strains with 3× DRE or 3× GCC elements were cultured on SD/-Ura medium with 50, 100, and 150 ng/mL of aureobasidin A (AbA) to select the minimum inhibitory concentration. Second, the complete and deletion EAR motif of 14 SlERF CDS were amplified (primers in the Supplementary Table 1) and inserted into the SmalI-linearized pGADT7-Rec AD vector by the In-Fusion PCR Cloning Kits, and then the AD-prey recombinant vectors were transformed into the YIH gold yeast competent cells with 3× DRE or 3× GCC elements. The yeast cells were selected on an SD/-Leu/AbA plate and identified by the colony PCR analysis (Matchmaker Insert Check PCR Mix 2, Clontech, Mountain View, CA, United States). Each screening was performed three times.

## Results

### Sequence Correction of ERF and DREB Subfamily Genes

To ensure the sequence accuracy of all AP2/ERF genes, the Pfam model (pf00847) of the AP2 domain downloaded from the Pfam website was used to search the tomato v4.0 protein database. A total of 166 AP2/ERF proteins with an AP2 domain *E*-value < 0.001 were obtained. Among these proteins, 20 had ≥2 AP2 domains, while 146 proteins had single AP2 domain. Among the latter, 3 proteins with the B3 domain were RAV-type AP2/ERF proteins. Thus, there were 143 ERF subfamily proteins with a single AP2 domain. The 143 protein sequences were submitted to the Pfam, CDD, and smart websites for conservative domain analysis. Subsequently, 140 tomato ERF subunit proteins with a single AP2 domain were identified. The sequences of these proteins were compared in 3 tomato genome sequencing protein databases (versions 2.0, 3.2, and 4.1) (Supplementary Table 2); 27 genes were found to be different. The protein and CDS sequences of these 27 genes were compared and confirmed according to the tomato genome and NCBI databases (Supplementary Tables 3, 4). Finally, the corrected protein sequences were used for subsequent analyses.

### Characteristics, Polarity, and Chemical Structure Analysis of the 14th and 19th Amino Acids in the AP2 Domain

Among the 140 ERF proteins with a single AP2 domain, the 14th amino acid of the AP2 domain was Valine (V) in 57 genes. Among these 57 proteins, the 19th amino acid of the AP2 domain was glutamic acid (E) in 30 proteins, aspartic acid (D) in 4 proteins, asparagine (N) in 1 protein, glutamine (Q) in 4 proteins, histidine (H) in 6 proteins, leucine (L) in 10 proteins, alanine (A) in 1 protein, and V in 1 protein (Table 1 and Supplementary Table 5). These 57 proteins were identified as DREB proteins. Additionally, the 14th and 19th amino acids of the AP2 domain were isoleucine (I) and D, respectively, in SlERF2-5, SlERF10-6, and SlERF10-8. The codon of I was AUA/AUC, GUA/GUG/GUU/GUC for V, but GCA/GCG/GCU/GCC for A. The characteristics, polarity, and chemical structure of I and V were hydrophobic, non-polar, and aliphatic, while A was neutral, non-polar, and aliphatic (Table 1 and Supplementary Table 5). Thus, I can only be a V mutation. Accordingly, the 3 proteins were identified as DREB proteins. In the 19th amino acid of the AP2 domain, the hydrophilic amino acids included E, D, N, Q, and H, the hydrophobic amino acids included L and V, and the neutral amino acids included A. The negative charged amino acids (E and D), uncharged amino acids (N and Q), and positively charged amino acids (H) were polar; the non-polar amino acids included L, A, and V. Additionally, H had a heterocycle chemical structure, while the others were aliphatic (Table 1). These differences may affect the functionality of DREB protein interactions with DRE and GCC boxes.

**TABLE 1:** The 14th/19th amino acid analysis of the DREB subfamily AP2 domain.

Gene number	14th	19th	14th codon	19th codon	14th/19th Characters	14th polarity	14th chemical structure	19th polarity	19th chemical structure
30	V	E	GUA/GUG/ GUU/GUC	GAA/GAG	Hydrophobic/hydrophilic	Non-polarity	Aliphatic	Polarity with negative charge	Aliphatic
4	V	D	GUU	GAU/GAC	Hydrophobic/hydrophilic	Non-polarity	Aliphatic	Polarity with negative charge	Aliphatic
1	V	N	GUU	AAC	Hydrophobic/hydrophilic	Non-polarity	Aliphatic	Polarity, without charge	Aliphatic
4	V	Q	GUA/GUG/ GUU	CAA	Hydrophobic/hydrophilic	Non-polarity	Aliphatic	Polarity without charge	Aliphatic
6	V	H	GUU/GUC	CAU/CAC	Hydrophobic/hydrophilic	Non-polarity	Aliphatic	Polarity with positive charge	Heterocycle
10	V	L	GUA/GUG/ GUU/GUC	UUG/CUU/ CUA/UUA	Hydrophobic/hydrophobic	Non-polarity	Aliphatic	Non-polarity	Aliphatic
1	V	A	GUA	GCA	Hydrophobic/neutral	Non-polarity	Aliphatic	Non-polarity	Aliphatic
1	V	V	GUG	GUU	Hydrophobic/hydrophobic	Non-polarity	Aliphatic	Non-polarity	Aliphatic
2	I	D	AUA	GAC	Hydrophobic/hydrophilic	Non-polarity	Aliphatic	Polarity with negative charge	Aliphatic
1	I	V	AUC	GUU	Hydrophobic/hydrophobic	Non-polarity	Aliphatic	Non-polarity	Aliphatic

Among the 80 ERF subfamily members, the 14th and 19th amino acids of the AP2 domain were A and D in 70 proteins. Additionally, there was an A and tyrosine (Y) in 1 gene, A and N in 1 protein, threonine (T) and D in 1 protein, serine (S) and D in 4 proteins, E and D in 1 protein, glycine (G) and N in 2 proteins, and I and V in 1 protein (Table 2 and Supplementary Table 6). In the 14th amino acid of the AP2 domain, the neutral amino acids included A, T, S, and G, and the hydrophilic amino acid included E. The non-polar amino acid was A, the polar amino acids without charges were T, S, and G, and the chemical structure of these amino acids is aliphatic. In the 19th amino acid of the AP2 domain, D, Y, and N comprised the hydrophilic amino acids, the negatively charged amino acid (D), the uncharged amino acids (Y and N) were polar, and the chemical structure of these amino acids was aliphatic (Table 2). Thus, the 80 proteins with a single AP2 domain were identified as ERF subfamily members. These differences may affect the functionality of ERF protein interactions with GCC boxes.

**TABLE 2:** The 14th/19th amino acid analysis of the ERF subfamily AP2 domain.

Gene number	14th	19th	14th codon	19th codon	Characters	14th polarity	14th chemical structure	19th polarity	19th chemical structure
70	A	D	GCA/GCG/ GCU/GCC	GAU/GAC	Neutral/hydrophilic	Non-polarity	Aliphatic	Polarity with negative charge	Aliphatic
1	A	Y	GCA	UAU	Neutral/hydrophilic	Non-polarity	Aliphatic	Polarity without charge	Aromatic
1	A	N	GCU	AAU	Neutral/hydrophilic	Non-polarity	Aliphatic	Polarity without charge	Aliphatic
1	T	D	ACG	GAU	Neutral/hydrophilic	Polarity without charge	Aliphatic	Polarity with negative charge	Aliphatic
4	S	D	UCU/UCA	GAU/GAC	Neutral/hydrophilic	Polarity without charge	Aliphatic	Polarity with negative charge	Aliphatic
1	E	D	GAA	GAU	Hydrophilic/hydrophilic	Polarity with negative charge	Aliphatic	Polarity with negative charge	Aliphatic
2	G	N	GGA	AAC	Neutral/hydrophilic	Polarity without charge	Aliphatic	Polarity without charge	Aliphatic

### Phylogenetic Analysis of ERF and DREB Proteins

**FIGURE 1:**
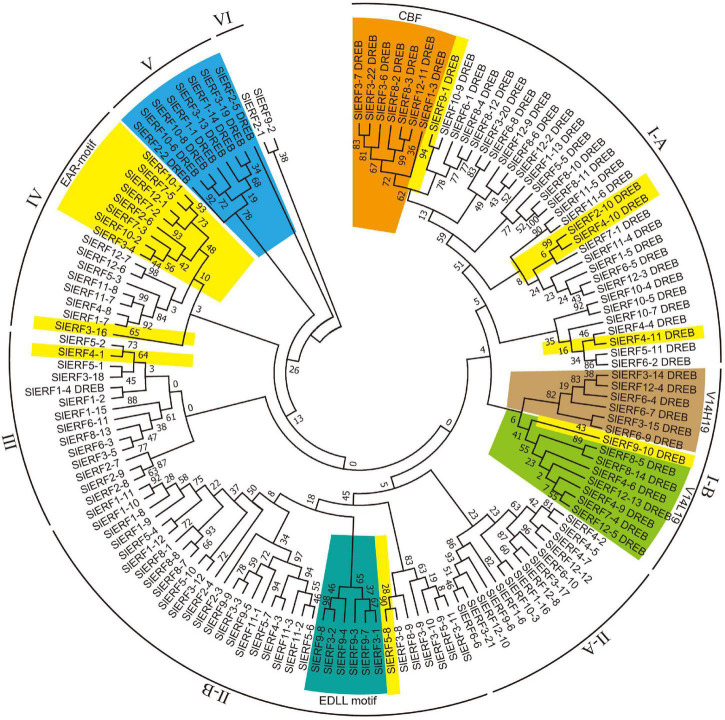
Phylogenetic analysis of 60 DREB and 80 ERF proteins in tomato. Blue, DREB proteins in group V; yellow, EAR motif-containing proteins; orange, CBF proteins; emerald green, EDLL motif-containing proteins; yellow green and latin yellow, respectively indicate DREB proteins with V14L19 and V14H19. Maximum likelihood method was used to structure phylogenetic tree based on the Whelan and Goldman model of MEGA v7.0 (Whelan and Goldman, 2001), parameters: JTT + G model, partial deletion with 80% site coverage cutoff, and 1,000 bootstrap replications (Kumar et al., 2016).

To understand their genetic relationships, the protein sequences of the 60 DREB and 80 ERF subfamily members were classified into 6 groups (Figure 1). The I group included 51 DREB proteins. Among these proteins, 37 and 14 proteins differentiated into the I-A and I-B subgroups, respectively. The I-A subgroup included 30 proteins with V14E19, 3 proteins with V14Q19 (SlERF12-9, SlERF1-13, and SlERF7-1), 1 protein with V14A19 (SlERF11-4), 1 protein with V14V19 (SlERF1-5), and 2 proteins with V14L19 (SlERF6-5 and SlERF12-3) (Supplementary Table 5). Seven CBF proteins (SlERF3-7, SlERF3-22, SlERF3-6, SlERF8-2, SlERF8-3, SlERF12-11, and SlERF1-3) clustered together and were in the I-A subgroup (Figure 1). Additionally, the I-A subgroup included 4 proteins (SlERF9-1, SlERF2-10, SlERF4-10, and SlERF4-11) with EAR motif (DLNxxP or LxLxL) (Supplementary Table 5). However, the I-B subgroup only included 6 proteins with V14H19 and 8 proteins with V14L19. SlERF9-10 and SlERF8-14 showed EAR motif in the I-B subgroup (Figure 1 and Supplementary Table 5).

Group II included 49 ERF subfamily proteins. Among these proteins, 14 and 35 proteins differentiated into the II-A and II-B subgroups, respectively (Figure 1). All members of the II-A subgroup belonged to ERF proteins with A14D19. In the II-B subgroup, there were 33 proteins with A14D19, 1 protein with T14D19 (SlERF1-10), and 1 protein with S14D19 (SlERF1-11) (Supplementary Table 6). In the II-B subgroup, 6 proteins with the EDLL transactivation motif (ExxxxDxxxLxxxL) clustered together (SlERF3-1, SlERF9-7, SlERF9-3, SlERF9-4, SlERF3-2, and SlERF9-8) (Figure 1). SlERF5-8 was also an EAR motif-containing protein in the II-B subgroup. Groups III and IV included 13 and 16 ERF subfamily proteins, respectively. However, group III also included a DREB protein with V14D19 (SlERF1-4) that clustered with an ERF-type protein (SlERF1-2). One protein with S14D19 (SlERF1-15) and 1 EAR motif-containing protein (SlERF4-1) were clustered into group III. Among these proteins in group IV, there were 2 proteins with S14D19 (SlERF3-16 and SlERF12-1), 2 proteins with G14N19 (SlERF12-6 and SlERF12-7), and all others belonged to proteins with A14D19. Additionally, 9 EAR motif-containing proteins (SlERF10-1, SlERF7-5, SlERF12-1, SlERF7-2, SlERF2-6, SlERF7-3, SlERF10-2, SlERF3-4, and SlERF3-16) were in group IV (Figure 1 and Supplementary Table 6). Group V had 8 DREB proteins, including 3 proteins with V14D19, 1 protein with V14Q19, 1 protein with V14N19, 2 proteins with I14D19, and 1 protein with I14V19 (Figure 1 and Supplementary Table 5). Group VI included 1 protein with E14D19 (SlERF2-1) and 1 protein with A14D19 (SlERF9-2) (Supplementary Table 6).

### Motif Analysis of ERF and DREB Protein Sequences

**FIGURE 2:**
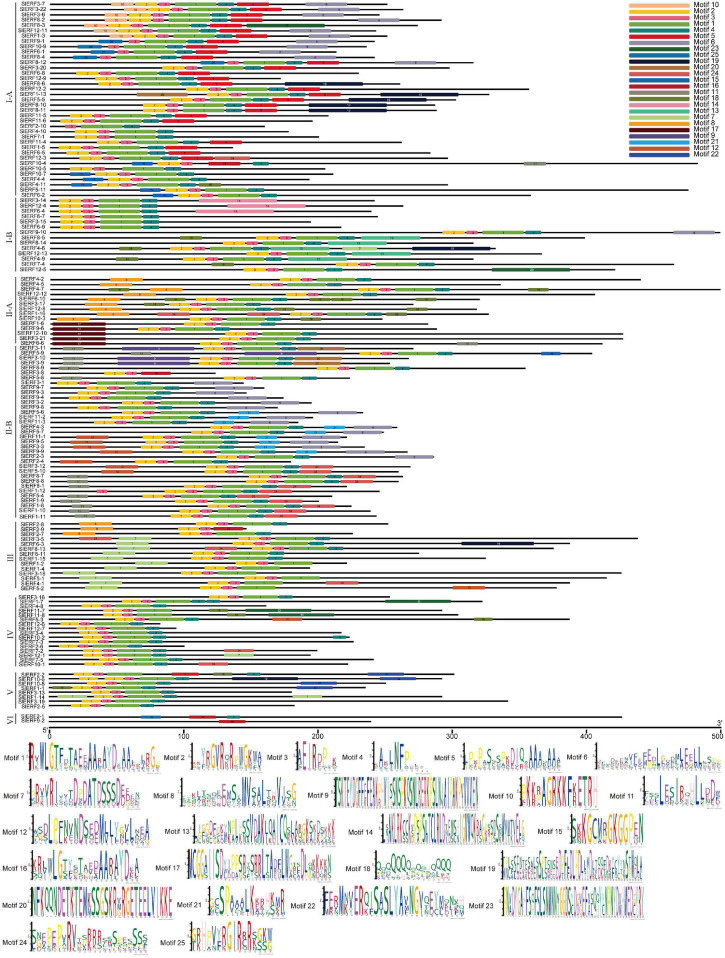
Twenty-five motif predictive analysis of 60 DREB and 80 ERF proteins in tomato using a Multiple Em for Motif Elicitation (MEME).

To understand the constructional characteristics of ERF and DREB proteins, a Multiple Em for Motif Elicitation (MEME) analysis was conducted to calculate the possible motifs of the 140 proteins. β1 of the AP2 domain was located on the left of motif 2, β2 was located on the right of motif 2 and left of motif 3, and β3 and α were located on motif 1. All 140 proteins, except SlERF9-1, SlERF10-9, SlERF6-1, SlERF8-4, SlERF8-12, SlERF3-8, SlERF2-1, and SlERF9-2, had motifs 1, 2, and 3. SlERF9-1, SlERF10-9, SlERF6-1, SlERF8-4, and SlERF8-12 had only motifs 1 and 3, as well as a same sequence to motif 25 in front of motif 3. SlERF3-8 had motifs 2, 3, and 16. Motif 16 had a similar sequence as motif 1. SlERF2-1 and SlERF9-2 did not have motifs 1, 2 and 3, but had a similar sequence as motif 16 with motif 1 (Figure 2). These results suggest that SlERF9-1, SlERF10-9, SlERF6-1, SlERF8-4, SlERF8-12, SlERF3-8, SlERF2-1, and SlERF9-2 may have the low ability to bind with GCC or DRE boxes.

In addition to motifs 1, 2, 3, 16, and 25, some motifs were located on both sides of the AP2 domain of many ERF and DREB proteins. For example, motifs 10 and 20 were near the left of the AP2 domain in 7 CBF and SlERF1-13, respectively, while motifs 4 and 5 were near the right of motif 1 and especially motif 4 (Figure 2). These findings suggest that motifs 4, 5, 10, and 20 may be involved in the process of AP2 domain binding with GCC or DRE boxes. However, motifs 6–9, 11–15, 17–19, 21, 23, and 24 were relatively far away from the AP2 domain, may be located in the transactivation or repression domains, and may be involved in regulating the expression of their downstream target genes. However, some ERF and DREB proteins, including SlERF4-10, SlERF7-1, SlERF10-5, SlERF6-7, SlERF3-15, SlERF6-9, SlERF3-16, SlERF12-6, SlERF12-7, SlERF3-4, SlERF7-3, SlERF2-6, SlERF3-13, and SlERF2-5, did not have other motifs, except motifs 1, 2, 3, and 4 (Figure 2). Nevertheless, a few of these proteins had a typical EAR motif, including SlERF4-10, SlERF3-16, SlERF3-4, SlERF7-3, and SlERF2-6. These proteins bound to DNA with the AP2 domain and repressed the expression of downstream target genes with the EAR domain. However, SlERF7-1, SlERF10-5, SlERF6-7, SlERF3-15, SlERF6-9, SlERF12-6, SlERF12-7, SlERF3-13, and SlERF2-5 especially protein sequences with <100 amino acids (SlERF12-6 and SlERF12-7) may competitively inhibit other ERF and DREB proteins (Figure 2).

### The Special Amino Acid of AP2 Domain May Affect Protein Binding With DRE/CRT and GCC Boxes

**FIGURE 3:**
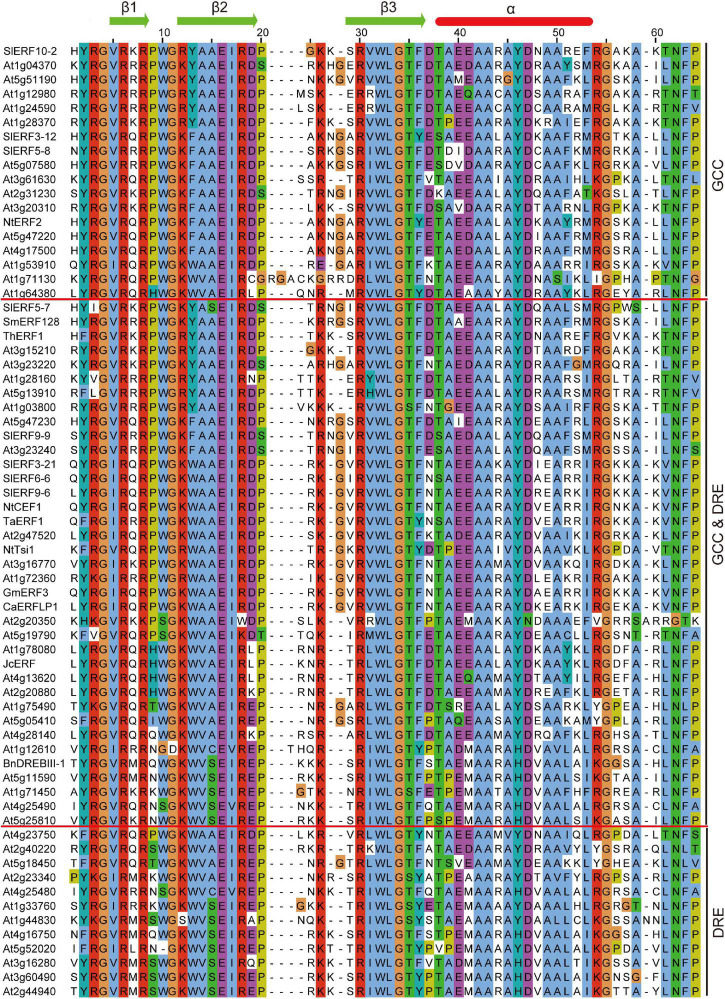
Comparison of the AP2 domain amino acid sequences of 49 *A. thaliana* and 19 other species ERF proteins. SlERF10-2, SlERF3-12, and SlERF5-8 (Tournier et al., 2003); At1g04370, At5g51190, At1g12980, At1g24590, At1g28370, At5g07580, At3g61630, At2g31230, At3g20310, At4g17500, At1g53910, At1g71130, At1g64380, At3g23220, At1g28160, At5g13910, At1g03800, At2g20350, At5g19790, At4g13620, At2g20880, At1g75490, At4g28140, At1g12610, At1g71450, At4g25490, At2g40220, At5g18450, At2g23340, At4g25480, At1g33760, At1g44830, At4g16750, At5g52020, At3g16280, At3g60490, At4g23750, and At2g44940 (Gong et al., 2008); NtERF2 (Solano et al., 1998); At5g47220, At3g15210, and At5g47230 (Fujimoto et al., 2000); SlERF5-7 (Huang et al., 2004), SmERF128 (Zhang et al., 2019), ThERF1 (Wang et al., 2014), SlERF9-9 (Klay et al., 2018), At3g23240 (Solano et al., 1998), SlERF3-21 (Wu et al., 2008), SlERF6-6 (Zhang et al., 2004), SlERF9-6 (Zhang Z. et al., 2009), NtCEF1 (Lee et al., 2005), TaERF1 (Xu et al., 2007), At2g47520 (Lee et al., 2015), NtTsi1 (Park et al., 2001), At3g16770 (Büttner and Singh, 1997), At1g72360 (Licausi et al., 2010), GmERF3 (Zhang G. et al., 2009), CaERFLP1 (Jae-Hoon et al., 2004), At1g78080 (Lin et al., 2008), JcERF (Tang et al., 2007), At5g05410 (Sakuma et al., 2002), BnDREBIII-1 (Liu et al., 2006), At5g11590 (Wei et al., 2005), and At5g25810 (Sun et al., 2008).

Previous studies found that some DREB and ERF subfamily proteins only bound to DRE or GCC boxes, but most of these proteins can also interact with these boxes. However, the correlation between the characteristics and binding ability of DREB and/or ERF subfamily proteins remains unclear. To distinguish the difference between DREB and ERF proteins during binding with DRE/CRT or GCC boxes, the AP2 domain amino acid sequences of 49 *A. thaliana* and 19 other species ERF proteins, including 8 tomato ERF proteins, were compared. The binding assays of the 68 ERF proteins with DRE and GCC boxes were completed through an electrophoretic mobility shift assay (EMSA), yeast one-hybrid, or proteome chip assays. Among these proteins, there were 42 protein AP2 domains that included P9, 5 included H9, 5 included S9, 6 included N9, 3 included Q9, 2 included K9, 2 included T9, and 1 included I9 (Figure 3). Only 19 proteins bound with GCC box, including 17 ERF with P9, 2 DREB with 1 P9 and 1 H9. Additionally, 37 proteins bound with DRE/CRT and GCC boxes, including 23 ERF with P9, 14 DREB with 1 P9, 4 H9, 4 N9, 2 Q9, 1 T9, 1 K9, and 1 I9. Only 12 proteins bound with DRE, including 1 ERF with P9, 11 DREB with 5 S9, 2 N9, 2 K9, 1 Q9, and 1 T9 (Figure 3). These results suggest that almost all ERFs with P9 and H9 can interact with GCC box, and most can also bind with DRE/CRT. All DREB with S9 can interact with DRE/CRT, but other DREBs with N9, K9, Q9, T9, and I9 can also bind with DRE/CRT and/or GCC boxes. The A14 and A15 amino acids of ERF AP2 domain were conserved, but the 13th amino acid may be Y, F, or W. The W13 and V14 amino acids of the DREB AP2 domain were conserved, but the 15th amino acid may be S, A, or C (Figure 3). These characteristics of ERFs and DREBs may affect the ability of proteins to bind with DRE/CRT and GCC boxes.

In tomato DREB subfamily members, there are 10 DREBs with S9W13V14S15 (SlERF3-20, SlERF5-5, SlERF6-8, SlERF8-10, SlERF8-11, SlERF8-12, SlERF9-1, SlERF10-9, SlERF11-4, and SlERF12-9), 1 DREB with S9W13V14C15 (SlERF8-4) (Supplementary Table 3), and 1 DREB with S9W13I14A15 (SlERF10-6), which suggests that these 11 DREBs may bind with DRE/CRT. There were 4 DREBs with H9W13V14S15 (SlERF12-4, SlERF6-7, SlERF3-15, and SlERF6-9) and 9 DREB with H9W13V14A15 (SlERF3-14, SlERF9-10, SlERF8-5, SlERF8-14, SlERF4-6, SlERF12-13, SlERF4-9, SlERF7-4, and SlERF12-5) (Supplementary Table 3), which suggest that these interact with the GCC box. Seven CBF proteins exist in tomato DREB subfamily members, including 5 CBF with N9W13V14C15 (SlERF3-7, SlERF3-22, SlERF3-6, SlERF8-2, and SlERF1-3) and 2 CBF with D9W13V14C15 (SlERF8-3 and SlERF12-11). There were 3 DREB with N9W13V14S15 (SlERF6-1, SlERF8-6, and SlERF1-13), 5 DREB with K9W13V14S15 (SlERF11-5, SlERF11-6, SlERF1-5, SlERF6-5, and SlERF12-3), 4 DREB with K9W13V14A15 (SlERF2-10, SlERF4-10, SlERF7-1, and SlERF1-4), 6 DREB with T9W13V14A15 (SlERF10-4, SlERF10-5, SlERF10-7, SlERF4-4, SlERF4-11, and SlERF5-11), 3 DREB with 1 Q9W13V14S15 (SlERF6-4), 1 I9W13V14A15 (SlERF6-2), and 1 A9W13V14S15 (SlERF12-2), 5 DREB with P9W13V14A15 (SlERF2-2, SlERF1-1, SlERF3-13, SlERF1-14, and SlERF3-19), and 1 DREB with P9W13I14A15 (SlERF10-8) (Supplementary Table 3). These DREB proteins may interact with DRE/CRT, some of which may also bind with GCC box.

In tomato ERF subfamily members, there were ERFs with 22 P9Y13A14A15, 21 P9F13A14A15, and 21 P9W13A14A15 (Supplementary Table 3). There were ERFs with K9Y13A14 A15 (SlERF3-18), P9Y13A14S15 (SlERF5-7), P9Y13G14A15 (SlERF12-7), P9Y13G14V15 (SlERF12-6), T9F13A14A15 (SlERF3-2), K9F13A14T15 (SlERF5-1), Q9F13S14A15 (SlERF1-11), P9F13S14A15 (SlERF12-1), Q9F13T14A15 (SlERF1-10), E9W13A14A15 (SlERF1-2), 2 with K9W13A14A15 (SlERF4-1 and SlERF5-2), and 2 with P9W13S14A15 (SlERF1-15 and SlERF3-16) (Supplementary Table 3). This indicates that P9A14A15 of the ERF AP2 domain may play an important role in binding with GCC box.

### Approximately 21 ERF and 15 DREB Genes Are Regulated by Auxin and/or Ethylene During Tomato Fruit Development and Ripening

**FIGURE 4:**
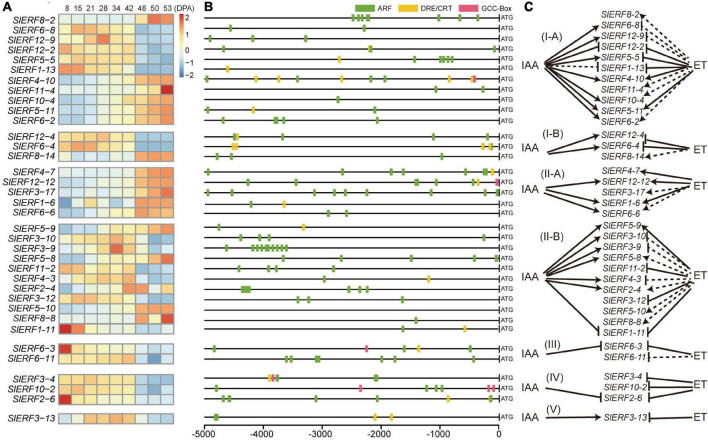
The relationship of DREB and ERF subfamily genes with tomato fruit development and ripening. **(A)** Heatmap analysis of 36 *SlERF* genes based on TPM values from 8 to 53 DPA. **(B)** ARF, DRE/CRT, and GCC boxes analysis of 36 *SlERF* gene promoters. **(C)** Relationship of 36 *SlERF* genes with IAA and ET signals. Black solid arrow, indicates direct positive regulation; black dashed arrow, indicates indirect positive regulation; black solid lines ending with bar, indicates direct repression; black dashed lines ending with bar, indicates indirect repression.

To explore the relationship of ERF family members with fruit development and ripening, the tomato fruit RNA-Seq data from NCBI SRA were analyzed (Supplementary Table 7). Among the 140 *SlERF* genes, the expression of 36 genes showed some regular changes from 8 to 53 days post anthesis (DPA). In the I-A group, the transcripts per million (TPM) values of *SlERF4-10*, *SlERF10-4*, *SlERF5-11*, and *SlERF6-2* increased during fruit development (from 8 to 28 DPA) and during ripening (from 28 to 53 DPA), whereas that of *SlERF8-2* and *SlERF11-4* did not change during fruit development and increased during ripening (Figure 4A and Supplementary Table 7). In the 5,000-bp promoter sequence of these genes, *SlERF8-2*, *SlERF11-4*, *SlERF10-4*, and *SlERF6-2* had 8, 2, 1, and 5 ARF binding sites, respectively; *SlERF4-10* had 6 ARF, 5 DRE/CRT, and 1 GCC box binding sites; *SlERF5-11* had 2 ARF and 1 DRE/CRT binding sites (Figure 4B). In addition, *SlERF4-10* had an EAR motif. These results suggest that *SlERF4-10* and *SlERF5-11* could be directly induced by IAA signal during fruit development and ET signal during ripening; *SlERF8-2* and *SlERF11-4* just responded to the ET signal during ripening, and *SlERF10-4* and *SlERF6-2* could be directly induced by IAA signal during development and indirectly regulated by ET interaction with other hormone signals during ripening (Figure 4C). In addition, the TPM values of *SlERF6-8*, *SlERF12-9*, and *SlERF5-5* showed the opposite trend with the above six genes, and those of *SlERF12-2* and *SlERF1-13* decreased during fruit development and ripening (Figure 4A and Supplementary Table 7). *SlERF6-8* and *SlERF12-9*, respectively, had two and three ARF binding sites; *SlERF5-5* had six ARF and one DRE/CRT binding sites; *SlERF1-13* had one DRE/CRT binding site; *SlERF12-2* had three ARF and one DRE/CRT binding sites (Figure 4B). These results indicated that IAA signal directly promoted *SlERF6-8*, *SlERF12-9*, and *SlERF5-5* and inhibited *SlERF12-2* and *SlERF1-13* expression, but ET signal directly repressed *SlERF5-5*, *SlERF12-2*, and *SlERF1-13* expression and indirectly inhibited *SlERF6-8* and *SlERF12-9* expression (Figure 4C).

In the I-B group, the TPM values of *SlERF12-4* and *SlERF6-4* slightly increased during fruit development and markedly reduced during fruit ripening (Figure 4A and Supplementary Table 7). Moreover, the *SlERF12-4* promoter showed four ARF, two DRE/CRTs, and one GCC box binding sites (Figure 4B). Interestingly, one GCC box closed to one ARF binding sites between −4,491 and −4,501 bp, and one ARF site showed a 5-bp overlap with one DRE/CRT sites within −190 to −197 bp (Supplementary Tables 8, 9). The *SlERF6-4* promoter showed four ARF and two DRE/CRT binding sites (Figure 4B), and two ARF site showed a 5-bp overlap with two DRE/CRT sites within −259 to −252 bp and −136 to −129 bp (Supplementary Tables 8, 9). These results suggested that *SlERF12-4* and *SlERF6-4* were directly induced by IAA signal and inhibited by ET signal (Figure 4C). The TPM value of *SlERF8-14* did not clearly show regularity during fruit development, but significantly increased during fruit ripening (Figure 4A and Supplementary Table 7). However, its promoter only showed three ARF and no DRE/CRT or GCC box binding sites (Figure 4B), which indicated that *SlERF8-14* may be indirectly induced by ET signal interacting with other hormones during ripening and was not affected by IAA signal during fruit development.

In the II-A group, the TPM values of *SlERF4-7* and *SlERF3-17* did not visibly change during fruit development but significantly increased during fruit ripening; the TPM values of *SlERF12-12*, *SlERF1-6*, and *SlERF6-6* continuously increased during fruit development and ripening; the TPM value of *SlERF1-6* first increased and then decreased during fruit development, and continuously increased during fruit ripening (Figure 4A). In addition, *SlERF4-7* promoter had seven ARF and two DRE/CRT binding sites. Moreover, one ARF site showed a 5-bp overlap with one DRE/CRT site from −1,623 to −1,629 bp (Supplementary Tables 8, 9). The *SlERF12-12* promoter had six ARF, two DRE/CRT, and one GCC box binding sites, and one ARF site showed a 5-bp overlap with one DRE/CRT site from −438 to −444 bp (Supplementary Tables 8, 9). The *SlERF1-6* promoter had one ARF and one DRE/CRT binding sites. In addition, the promoters of *SlERF3-17* and *SlERF6-6* had nine and two ARF binding sites, respectively (Figure 4B). These results indicate that ET signal may directly induce *SlERF4-7*, *SlERF12-12*, and *SlERF1-6* expression and indirectly regulate *SlERF3-17*, and *SlERF6-6*; IAA signal directly improved *SlERF12-12*, *SlERF6-6*, and *SlERF1-6* expression but did not affect *SlERF4-7* and *SlERF3-17* expression during fruit development (Figure 4C).

In the II-B group, the TPM values of *SlERF5-9*, *SlERF5-8*, and *SlERF2-4* almost showed a continuously increasing trend from fruit development to ripening, while that of *SlERF5-10* and *SlERF8-8* did not change during fruit development and obviously increased during ripening (Figure 4A). Moreover, the *SlERF5-9* promoter had two ARF and one DRE/CRT binding sites, and one ARF site showed a 5-bp overlap with one DRE/CRT site from −3,318 to −3,324 bp (Supplementary Tables 8, 9). The promoters of *SlERF5-8*, *SlERF2-4*, *SlERF5-10*, and *SlERF8-8* had 5, 7, 0, and 1 ARF binding sites, respectively (Figure 4B). Four ARF sites in the *SlERF2-4* promoter were detected between −4,244 and −4,363 bp. In addition, *SlERF5-8* had an EAR motif. These results suggested that IAA signal could directly induce *SlERF5-9*, *SlERF5-8*, and *SlERF2-4* expression and did not affect *SlERF5-10* and *SlERF8-8* expression during fruit development; ET signal directly induced *SlERF5-9* transcription and indirectly enhanced *SlERF5-8*, *SlERF2-4*, *SlERF5-10*, and *SlERF8-8* expression by interacting with other hormones during fruit ripening (Figure 4C). However, the TPM values of *SlERF3-10*, *SlERF3-9*, and *SlERF4-3* visibly increased during fruit development and decreased during fruit ripening; the TPM values of *SlERF11-2* and *SlERF3-12* did not change during development and decreased during ripening (Figure 4A and Supplementary Table 7). Among these five genes, the promoters of *SlERF3-10*, *SlERF3-9*, *SlERF11-2*, and *SlERF3-12* had 4, 10, 4, and 3 ARF binding sites, respectively. The *SlERF4-3* promoter had one ARF and one DRE/CRT binding sites (Figure 4B). In addition, 9 ARF sites of the *SlERF3-9* promoter were clustered between −3,611 and −4,185 bp. The TPM value of *SlERF1-11* continuously decreased from fruit development to ripening. The *SlERF1-11* promoter had one ARF and one DRE/CRT binding sites. These results suggested that *SlERF3-10* and *SlERF3-9* could be directly induced by IAA signal during fruit development and indirectly inhibited by ET signal during fruit ripening; *SlERF11-2* and *SlERF3-12* expressions were not affected by IAA signal and were indirectly inhibited by ET signal; *SlERF1-11* expression was directly inhibited by IAA and ET signals, while *SlERF4-3* expression was directly induced by IAA during fruit development and repressed by ET during fruit ripening (Figure 4C).

In the III group, the TPM value of *SlERF6-3* was reduced during fruit development and ripening (Figure 4A and Supplementary Table 7). Moreover, the *SlERF6-3* promoter had four ARF, two DRE/CRT, and one GCC box binding sites (Figure 4B), and one ARF site showed a 5-bp overlap with one DRE/CRT site at −1,607 to −1,613 bp (Supplementary Tables 8, 9). This indicated that *SlERF6-3* may be negatively regulated by IAA and ET. In addition, the TPM value of *SlERF6-11* did not show obvious changes during fruit development and reduced during fruit ripening; the *SlERF6-11* promoter had seven ARF binding sites (Figures 4A,B). These findings suggested that IAA signal do not regulate *SlERF6-11* expression through the ARF pathway, but ET signal may indirectly inhibit *SlERF6-11* expression via other pathways (Figure 4C).

In the IV group, the TPM values of *SlERF3-4* and *SlERF10-2* did not obviously change during fruit development and decreased during fruit ripening (Figure 4A). The *SlERF3-4* promoter had four ARF, two DRE/CRT, and two GCC box binding sites, and one ARF, one DRE/CRT, and one GCC box sites were situated within the −3,760 to −3,776 bp (Supplementary Tables 8, 9). The *SlERF10-2* promoter had four ARF, one DRE/CRT, and three GCC box binding sites, and one ARF site showed a 5-bp overlap with one DRE/CRT site within −1,065 to −1,071 bp (Supplementary Tables 8, 9). These results suggested that *SlERF3-4* and *SlERF10-2* were not regulated by IAA signal during development but directly repressed by ET signal during ripening (Figure 4C). In addition, the TPM value of *SlERF2-6* continuously decreased from fruit development to ripening (Figure 4A and Supplementary Table 7). The *SlERF2-6* promoter had seven ARF and two DRE/CRT binding sites, and four ARF sites were located within −4,574 to −4,689 bp (Supplementary Tables 8, 9). These results indicate that *SlERF2-6* expression could be directly inhibited by IAA signal during fruit development and by ET signal during ripening (Figure 4C).

In the V group, the TPM value of *SlERF3-13* (DREB) increased during fruit development and decreased during ripening (Figure 4A and Supplementary Table 7). Moreover, its promoter had two ARF and two DRE/CRT binding sites (Figure 4B). These findings suggested that *SlERF3-13* could be directly induced by IAA signal during fruit development and directly repressed by ET signal during ripening (Figure 4C).

### Expression Analysis of EAR Motif-Containing *SlERF* Genes in Tomato

**FIGURE 5:**
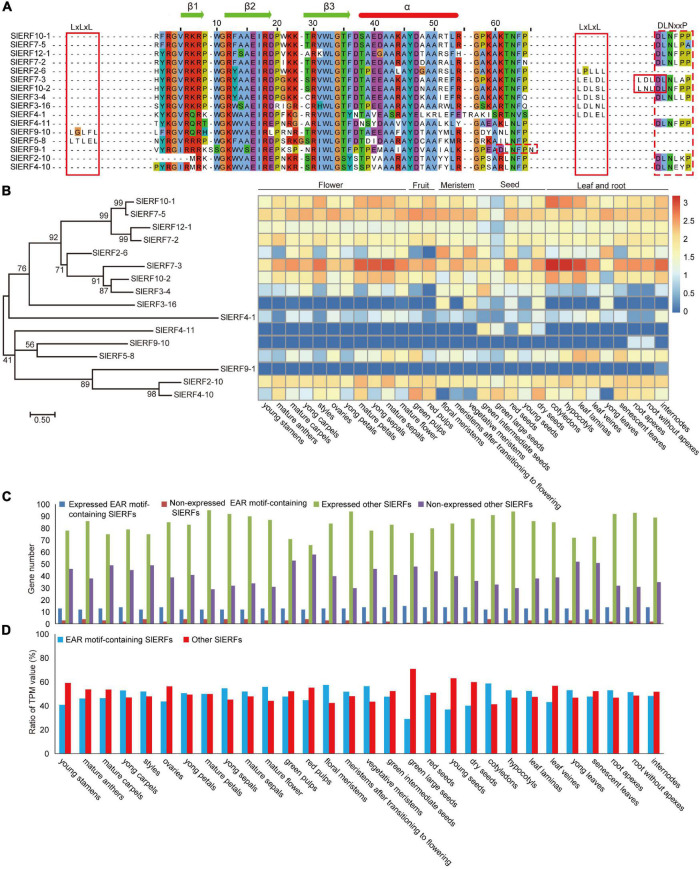
Expression levels of the EAR motif-containing *SlERF* genes in 30 tomato organs. **(A)** AP2 domain and EAR motifs analysis in the EAR motif-containing SlERF proteins. **(B)** Heatmap analysis of all EAR motif-containing *SlERF* genes based on TPM values in 30 tomato organs. **(C)** Gene numbers analysis of the expressed and non-expressed genes among EAR motif-containing *SlERFs* and other *SlERF* genes in 30 tomato organs. **(D)** The total TPM ratio analysis of the EAR motif-containing *SlERFs* and other *SlERF* genes in 30 tomato organs.

Among the 140 SlERF proteins, 11 ERF and 5 DREB subfamily proteins that have one or two typical EAR (LxLxL or DLNxxP) motifs were found. SlERF10-1, SlERF7-5, SlERF12-1, SlERF7-2, SlERF4-11, SlERF9-1, SlERF2-10, and SlERF4-10 have a DLNxxP motif in C-terminal; SlERF2-6, SlERF3-16, and SlERF4-1 have an LxLxL motif in C-terminal, but SlERF9-10 and SlERF5-8 have an LxLxL motif in N-terminal. In addition, SlERF7-3, SlERF10-2, and SlERF3-4 show not only an independent LxLxL motif but also a DLNxxP motif in C-terminal. However, the DLNxxP motif of SlERF7-3 and SlERF10-2 connect with an LxLxL sequences and form a strong repressive motif (Figure 5A).

To understand the function of the 16 EAR motif-containing *SlERF* genes, the TPM values of these genes were analyzed in flowers, fruits, meristems, seeds, leaves, and roots (Supplementary Table 10). Among these genes, *SlERF10-1*, *SlERF7-5*, *SlERF12-1*, *SlERF7-2*, *SlERF7-3*, *SlERF10-2*, and *SlERF2-10* were largely expressed in almost all organs; *SlERF2-6*, *SlERF3-4*, *SlERF4-10*, and *SlERF5-8* were expressed in most organs such as flowers, seeds, and leaves, but *SlERF2-6* and *SlERF3-4* were not expressed in fruits, in contrast to *SlERF4-10* and *SlERF5-8*. In addition, *SlERF3-16* was only expressed in floral and vegetative meristems, seeds, and young leaves; *SlERF4-11* was only expressed in seeds, *SlERF8-14* and *SlERF4-1* were low expressed in almost all organs, and *SlERF9-10* and *SlERF9-1* were not expressed in almost all organs (Figure 5B and Supplementary Table 10). These results indicate that *SlERF10-1*, *SlERF7-5*, *SlERF12-1*, *SlERF7-2*, *SlERF7-3*, *SlERF10-2*, and *SlERF2-10* are involved in regulating the development of almost all organs, other genes except *SlERF9-10* and *SlERF9-1* play roles in regulating the development of part organs.

Among the 16 EAR motif-containing *SlERF* genes, at least 12 genes were expressed in every organ, especially 15 genes in green large seeds (Figure 5C and Supplementary Table 10). In addition, most genes without the EAR motif were expressed in every organ such as the maximum 95 genes in mature petals and the minimum 66 in red pulp (Figure 5C and Supplementary Table 10). However, total TPM values of 16 ERFs with the EAR motif showed a very high ratio of all ERF genes in every organ, and exceeded genes without the EAR motif in most organs such as 55.84% in mature flowers, 57.53% in floral meristem, 56.50% in vegetative meristem, and 58.72% in cotyledons (Figure 5D and Supplementary Table 10). These results suggested that the EAR motif-containing *SlERF* genes may play important roles in balancing regulatory function of other ERF and DREB subfamily genes to downstream target genes during tomato growth and development.

### The EAR Motif of Most SlERFs Functions in Gene Repression in Tomato

**FIGURE 6:**
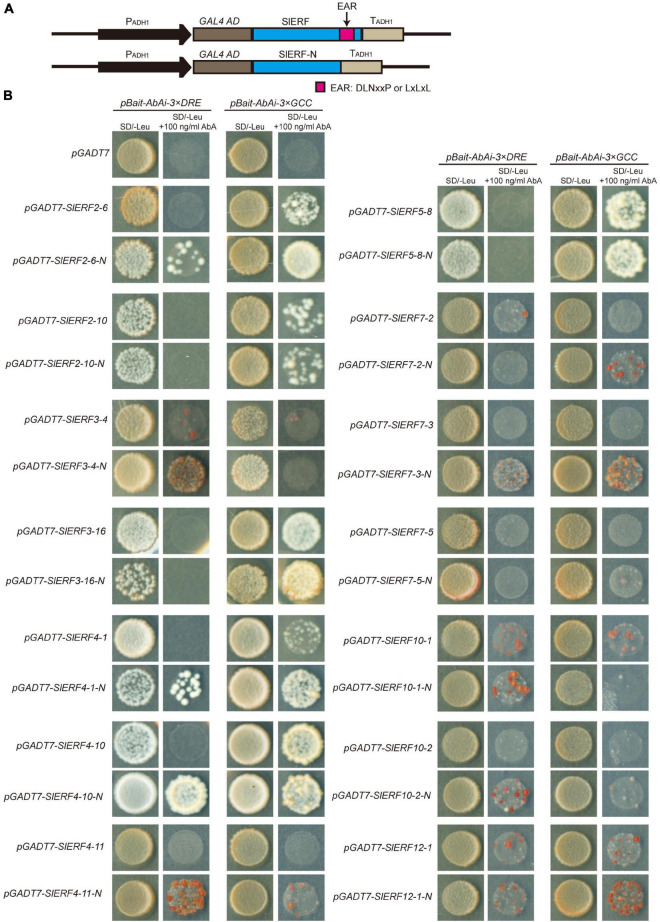
The repressing function analysis of the EAR motif in SlERF proteins by a Y1H assay. **(A)** Construction of the Y1H recombinant vectors. **(B)** The activation ability analysis of 14 SlERF proteins retained or deleted EAR motifs in a Y1H assay.

To understand whether the EAR motif plays a repressing role in ERF proteins regulating the expression of their target genes, a yeast one-hybrid experiment (Y1H) was used in this study. Out of 16 ERF proteins with the EAR motif, 14 these were selected for the construction of the *pGADT-SlERF* and *pGADT-SlERF-N* carriers. In the *pGADT-SlERF-N* carriers, the EAR sequences of the ERF proteins were deleted (Figure 6A). In Y1H, the yeast cells with *pBait-AbAi-3* × *DRE* and *pGADT* carrier did not grow in the yeast medium supplemented with 100 ng/mL AbA. The yeast cells with *pBait-AbAi-3* × *DRE* together with *pGADT-SlERF2-6*, *pGADT-SlERF2-10*, *pGADT-SlERF3-16*, *pGADT-SlERF4-1*, *pGADT-SlERF4-10*, *pGADT-SlERF4-11*, *pGADT-SlERF5-8*, *pGADT-SlERF7-3*, or *pGADT-SlERF7-5* carriers also showed the same appearance with the yeast cells including *pBait-AbAi-3* × *DRE* and *pGADT* carrier (Figure 6B). However, after the EAR sequences of these proteins were deleted, parts of these grew in the yeast medium supplemented with 100 ng/mL AbA such as SlERF2-6, SlERF4-1, SlERF4-10, SlERF4-11, and SlERF7-3. In addition, the number of the yeast cells with *pBait-AbAi-3* × *DRE* together *pGADT-SlERF3-4* or *pGADT-SlERF10-1* was low, but that of yeast cells without the EAR sequences of the two proteins significantly increased in the yeast medium supplemented with 100 ng/mL AbA. The yeast cells with *pGADT-SlERF7-2*, *pGADT-SlERF7-2-N*, *pGADT-SlERF12-1*, or *pGADT-SlERF12-1-N* carriers showed a few colonies, and those of *pGADT-SlERF2-10*, *pGADT-SlERF2-10-N*, *pGADT-SlERF3-16*, *pGADT-SlERF3-16-N*, *pGADT-SlERF5-8*, *pGADT-SlERF5-8-N*, *pGADT-SlERF7-5*, or *pGADT-SlERF7-5-N* did not grow in the yeast medium supplemented with 100 ng/mL AbA (Figure 6B). These results suggested that the EAR motifs of the SlERF2-6, SlERF4-1, SlERF4-10, SlERF4-11, SlERF7-3, SlERF3-4, and SlERF10-1 proteins repressed the expression of their target genes, of which the promoters had DRE/CRT elements, with those of SlERF7-2 and SlERF12-1 imparting weak effects, and the DNA binding domain of SlERF2-10, SlERF3-16, SlERF5-8, and SlERF7-5 did not interact with the DRE element.

In the yeast cells with the *pBait-AbAi-3* × *GCC* and *pGADT* carrier, AbA could inhibit cells growth. This appearance also showed in the yeast cells with *pBait-AbAi-3* × *GCC* together *pGADT-SlERF3-4*, *pGADT-SlERF4-11*, *pGADT-SlERF7-2*, *pGADT-SlERF7-3*, *pGADT-SlERF7-5*, or *pGADT-SlERF10-2*. However, after the EAR sequences of these genes were deleted, the yeast cells including SlERF4-11-N, SlERF7-2-N, or SlERF7-3-N proteins could grow; those of SlERF3-4-N, SlERF7-5-N, and SlERF10-2-N proteins did not grow in the yeast medium supplemented with 100 ng/mL AbA (Figure 6B). The yeast cells with *pBait-AbAi-3* × *GCC* together *pGADT-SlERF2-6*, *pGADT-SlERF2-10*, *pGADT-SlERF3-16*, *pGADT-SlERF4-1*, *pGADT-SlERF4-10*, *pGADT-SlERF5-8*, *pGADT-SlERF10-1*, or *pGADT-SlERF12-1* carriers could grow in the yeast medium supplemented with 100 ng/mL AbA. After the EAR sequences of these genes were deleted, the yeast cells, including SlERF2-6-N, SlERF3-16-N, SlERF4-1-N, or SlERF12-1-N proteins, showed more growth, but those of SlERF2-10-N, SlERF5-8-N, or SlERF4-10-N proteins showed scarce changes compared to SlERF2-10, SlERF5-8, or SlERF4-10 proteins. In addition, yeast cells, including SlERF10-1-N proteins, did not even grow in the yeast medium supplemented with 100 ng/mL AbA (Figure 6B). These results indicated that the EAR motifs of SlERF2-6, SlERF3-16, SlERF4-1, SlERF4-11, SlERF7-2, SlERF7-3, and SlERF12-1 proteins repressed target gene promoters within the GCC box, and those of SlERF2-10, SlERF4-10, and SlERF5-8 might not effectively offset the activation of the AD domain when ERF proteins bind to the GCC box, and the AP2 domain of SlERF3-4, SlERF7-5, SlERF10-1, and SlERF10-2 might not or weakly bind to the GCC box.

### Some EAR Motif-Containing SlERFs Are Involved in Fruit Ripening

**FIGURE 7:**
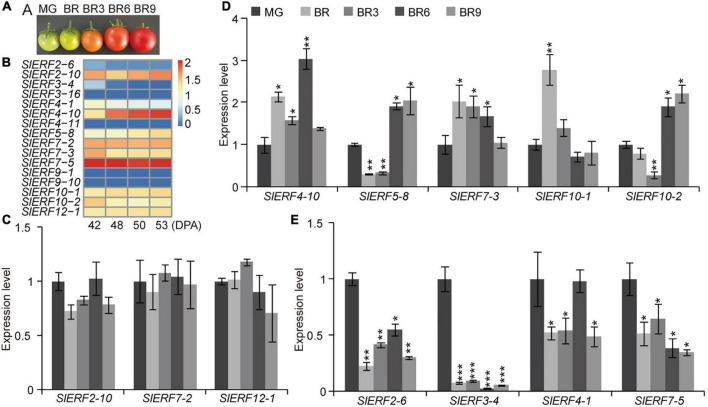
Expression levels of the EAR motif-containing *SlERF* genes in tomato fruit ripening. **(A)** Tomato fruit ripening images. **(B)** Heatmap analysis of the EAR motif-containing *SlERF* genes based on TPM values in tomato fruit ripening. **(C–E)** The expression levels of 12 EAR motif-containing *SlERF* genes in tomato fruit ripening. MG, mature green; BR, the beginning of the breaker stage; BR3, BR6, and BR9, 3, 6, and 9 days after the BR, respectively. Student’s *t*-test: *p < 0.05, **p < 0.01, and ***p < 0.001.

To investigate whether ERFs with the EAR motif regulated fruit ripening, the expression levels of 14 ERFs with the EAR motif were analyzed by qRT-PCR at fruit different ripening stages (Figure 7A). The results indicated that the expression levels of *SlERF2-10*, *SlERF7-2*, and *SlERF12-1* did not change (Figures 7B,C). In addition, *SlERF9-1* and *SlERF9-10* were not expressed in tomato fruits (Figure 7B). Therefore, these five genes were not involved in regulating fruit repining. At the beginning of the breaker stage (BR), *SlERF4-10, SlERF7-3*, and *SlERF10-1* were up-regulated, whereas *SlERF2-6*, *SlERF3-4*, *SlERF3-16*, *SlERF4-1*, *SlERF4-11*, *SlERF5-8*, and *SlERF7-5* were down-regulated. Three days after the BR, only the expression levels of *SlERF4-10* and *SlERF7-3* increased, whereas that of other genes, except for *SlERF2-10*, *SlERF7-2*, *SlERF10-1*, and *SlERF12-1*, decreased. Six days after the BR, *SlERF4-10, SlERF5-8, SlERF7-3*, and *SlERF10-2* were up-regulated, and whereas the other 5 *SlERF* genes, except for *SlERF2-10*, *SlERF4-1*, *SlERF7-2*, *SlERF10-1*, and *SlERF12-1*, were down-regulated. Nine days after the BR, only *SlERF5-8* and *SlERF10-2* were up-regulated, *SlERF2-10*, *SlERF4-1*, *SlERF4-10*, *SlERF7-2*, *SlERF7-3*, and *SlERF10-1* did not change, and six other genes were down-regulated (Figures 7D,E). These results indicate that *SlERF4-10* and *SlERF7-3* played regulatory roles at the BR for 1–6 days, *SlERF5-8* and *SlERF10-2* at the BR for 6–9 days, *SlERF10-1* only at the BR for 3 days, *SlERF2-10*, *SlERF7-2*, and *SlERF12-1* were not affected with the increase in ethylene release, while *SlERF2-6*, *SlERF3-4*, *SlERF3-16*, *SlERF4-1*, *SlERF4-11*, and *SlERF7-5* were inhibited by ethylene.

## Discussion

### The Ability of ERFs Binding With DNA Was Not Dependent on the AP2 Domain but Required the Motif Near the AP2 Domain

Ethylene responsive factor, as a specific transcription factor in plant, is involved in the life cycle of higher plants, including growth, development, abiotic, and biotic stresses (Chen Y. et al., 2021; Ji et al., 2021; Tian et al., 2021; Xing et al., 2021). However, the ERF family always includes numerous members in many plants (Zhu et al., 2018; Xu et al., 2020; Zong et al., 2021), while their functions are hard to distinguish in different life activities. Although the tomato genome has been sequenced and analyzed three times so far, there are still errors in the sequence analysis of many genes. In this study, we identified and analyzed 140 *SlERF* genes from tomato genome data v4.0 and then found that 26 of these showed errors in genome data v2.0, v3.2, or v4.0 such as initial codon missing prediction, part sequences deletion, intron missing prediction, and nucleotide deletion (Supplementary Table 2). Thus, correcting these errors as much as possible will help us better investigate *SlERF* functions.

In early reports, the 14th and 19th amino acids of AP2 domain were used to distinguish DREB and ERF subfamily proteins including V14E19, and A14D19 were, respectively, named DREB and ERF proteins. Moreover, DRE/CRT and GCC boxes only, respectively, interact with DREB and ERF proteins (Sakuma et al., 2002). For the 14th amino acid of AP2 domain, valine (V) had hydrophobic, non-polar, and aliphatic properties, while alanine (A) was neutral, non-polar, and aliphatic (Tables 1, 2). For the 19th amino acid of AP2 domain, both glutamic acid (E) and aspartic acid (D) were hydrophilic, aliphatic, and shared polarity with negative charge (Tables 1, 2). Therefore, the 14th amino acid of the AP2 domain could play an important role in ERF protein binding with DRE/CRT and GCC boxes, while the E19 or D19 of the AP2 domain might not affect this binding ability of ERF proteins with DRE/CRT and GCC boxes. For example, At5g19790 with V14D19 and At1g75490 with V14E19 could interact with both DRE/CRT and GCC boxes (Gong et al., 2008). Among 140 SlERF proteins, 60 and 80 were, respectively, divided into the DREB and ERF subfamilies. However, there were only 30 DREB proteins with V14E19 and 70 ERF with A14D19. In addition, the 19th amino acid of AP2 domain in some DREB proteins showed D, N, G, H, L, A, or V; that in some ERF proteins showed Y or N; the 14th amino acid of AP2 domain in two DREB proteins showed I and that in some ERF proteins showed T, S, E, and G. Property differences of these amino acids could have a certain effect when proteins bind with DRE/CRT and GCC boxes. In tomato, SlERF3-12 and SlERF5-8 with A14D19 only bind to the GCC box (Tournier et al., 2003), but SlERF9-9, SlERF3-21, SlERF6-6, and SlERF9-6 with A14D19 could interact with both DRE/CRT and GCC boxes (Zhang et al., 2004; Zhang Z. et al., 2009; Wu et al., 2008; Klay et al., 2018). In addition, SlERF5-7 with S14D19 was also demonstrated to bind with both DRE/CRT and GCC boxes (Huang et al., 2004). Furthermore, the AP2 domain consisted of three β-sheets and one α-helix; the arginine and tryptophan residues in the β-sheet could bind to the GCC box (Allen et al., 1998). In *Brassica napus*, BnDREBIII-1 interacted with DRE/CRT and GCC boxes, in contrast with BnDREBIII-4. The difference in their AP2 domain involved the 37th amino acid of the AP2 domain located in the α-helix (Liu et al., 2006). In this study, we compared the AP2 domain sequences of 68 ERF and DREB proteins from 20 species; the ability of these proteins binding with DRE/CRT and GCC boxes was assessed by EMSA, YIH, or proteome chip assays. We found that ERF proteins with P9A14A15 showed strong binding ability with the GCC box; in contrast, proteins with S/N/Q/K9V14S15 showed strong binding ability with DRE/CRT. However, some ERF proteins with P9A14A15 or S/N/Q/K9V14S15 could interact with DRE/CRT and GCC boxes (Figure 3). In addition, the MEME analysis results suggest that motifs 2, 3, 1, and 4 located in the AP2 domain, others are distributed outside the AP2 domain. Thus, motifs relatively distant from the AP2 domain such as motifs 7, 8, 9, 11, 12, 14, 17, 18, 19, 22, 23, and 24 may also act as a part of transactivation domain; those near the AP2 domain such as motifs 5, 10, 13, 15, 20, 24, and 25 may help the AP2 domain to bind with DRE/CRT and GCC boxes (Figure 2). For example, the PKKPAGR signature sequence of *A. thaliana* CBF1 located left of the AP2 domain, mutations within this motif reduce CBF1 binding with DRE/CRT element and the expression level of COR gene (Canella et al., 2010). Thus, the ability of the ERF protein to bind to DNA was not dependent on the AP2 domain but involved other amino acids near the AP2 domain.

### ERFs Are Involved in Fruit Development and Ripening Through Responses to Auxin and Ethylene Signals

The IAA promotes tomato fruit development, while the ET release improves fruit ripening. However, it is a complex regulatory mechanism that involves several metabolic networks. For example, MADS-box transcription factor ripening inhibitor (RIN) mutant showed incompletely ripened fruits (Ito et al., 2015, 2017), tomato organelle RNA recognition motif-containing proteins 4 (ORRM4) positively regulated fruit ripening (Yang et al., 2017), and DNA demethylase gene *SlDML2* was involved in DNA methylation of many genes during ripening (Lang et al., 2017). In this study, 36 ERF genes showed regular changes during tomato fruit development and ripening. The promoter of 35 of these showed ARF binding elements, but only 19 ERF genes were up-regulated during fruit development, which indicated that these genes may be directly induced by IAA signal (Figures 4A,B). However, *SlERF12-2*, *SlERF6-3*, and *SlERF2-6* were down-regulated during fruit development, suggesting that IAA signal negatively regulate their transcription. During fruit ripening, 17 ERF genes were up-regulated, but only promoters of *SlERF1-6*, *SlERF4-10*, *SlERF5-11*, *SlERF4-7*, *SlERF12-12*, and *SlERF5-9* showed the DRE/CRT and/or GCC boxes. As such, these may be directly induced by other ERF proteins. Interestingly, promoters of other 11 genes had no DRE/CRT and/or GCC boxes, indicating that these may be induced to regulate fruit ripening by ET interacting with other hormone pathways (Figures 4A–C). In addition, 19 ERF genes were inhibited by ET signal; 12 of these showed the DRE/CRT and/or GCC boxes and others did not harbor these elements (Figures 4A,B). These results suggested that ET signal directly or indirectly repressed these ERF gene expressions through other ERF proteins or other hormone pathways to improve fruit ripening. Early reports have demonstrated that *SlERF5-8* (*Sl-ERF.B3*) directly regulate *Sl-Aux/IAA27* to integrate ethylene and auxin signaling in tomato seedling development (Liu et al., 2018), while our results indicated that *SlERF5-8* expression continuously increased during fruit development and ripening, and its promoter had 5 ARF and no ERF binding elements (Figures 4A,B). In addition, the SlERF5-8 protein also had an EAR motif (Figure 5A). Thus, *SlERF5-8* and IAA and ET signals were found to have a negative feedback regulation relationship. Moreover, *SlERF5-10* (*SlPti4*)-silenced plants showed ABA accumulation, decrease in ET signals, and orange fruits; *SlERF5-10* could be induced by exogenous 1-aminocyclopropane 1-carboxylate (ACC) (Sun et al., 2018). However, *SlERF5-10* expression increased during ripening, but its promoter did not have ARF, DRE/CRT, and GCC boxes. These results indicated that *SlERF5-10* and ET signal had a feedback regulation relationship, i.e., *SlERF5-10* positively regulated ET biosynthesis and then ET signal indirectly induced *SlERF5-10* transcription during ripening. Thus, our results suggested that some ERF genes can be directly or indirectly induced by IAA and/or ET signals and feedback regulate IAA or ET biosynthesis during fruit development and ripening in tomato.

### Most EAR Motif-Containing SlERFs as Inhibitors Balance Other ERF Functions During Tomato Growth and Development

The EAR motif-containing proteins repressed transcription of their target genes through recruiting histone deacetylase (Yang et al., 2018), but proteins without EAR motif did not have the same recruiting ability. In plants, the EAR motif-containing proteins play many important roles in regulating plant growth, development, and defense response (Chen et al., 2008; Jin et al., 2018; Lakehal et al., 2020; Lim et al., 2020). For example, *AtERF115* can repress adventitious rooting in *A. thaliana* through the JA and cytokinin signaling pathways (Gu et al., 2002); *AtERF4* inhibits the expression of JA-responsive defense gene and antagonizes JA inhibition of root elongation (McGrath et al., 2005). In this study, we found that only 11 ERF and 5 DREB proteins were present that included EAR motif in tomato (Figures 1, 5A), but it is unclear whether these ERF proteins balanced with other ERFs without EAR motif proteins by competing between common target sites. In this study, we found that at least 13 EAR motif-containing ERF genes were expressed in several floral organs, red pulp, cotyledons, and senescent leaf, and at most 15 EAR motif-containing ERF genes were expressed in green large seeds (Figure 5C). However, most of other 124 ERF genes are expressed in every organ, such as the maximum 95 genes in mature petals and the minimum 66 genes in red pulp (Figure 5C). In addition, the TPM value ratio of EAR motif-containing ERF genes exceeded that of others in tomato 16 organs, especially in cotyledons (Figure 5D). Therefore, the EAR motif-containing ERF proteins may control the activation function of other ERF and DREB proteins by competition the binding sites of their target genes, and finally balance the expression of their target genes to ensure normal growth and development in tomato.

**FIGURE 8:**
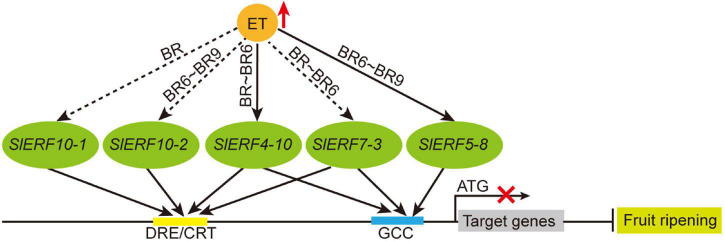
The relationship of ET and EAR motif-containing SlERF genes during tomato fruit ripening. Red upward arrow, indicates increase; black solid arrow between ET and SlERF genes, indicates direct induction; black dashed arrow, indicates indirect induction; black solid arrow between SlERF and DRE/CRT or GCC boxes, indicates interaction; black line ending with bar, indicates repression; red cross, indicates transcriptional inhibition.

However, whether these proteins act as repressors remains unclear. Thus, 14 of these genes (*SlERF9-1* and *SlERF9-10* were hardly expressed in the tomato 30 tissues) were analyzed in a YIH experiment. Our results indicated that SlERF2-10, SlERF3-16, and SlERF5-8 only interacted with GCC box and its activity was hardly affected by EAR motif (Figure 6B). This may be because EAR motifs do not effectively offset the activity function of AD domain. However, the activities of other 10 proteins, except for SlERF7-5, were more or less affected by the EAR motif. SlERF3-4, SlERF4-11, SlERF7-3, SlERF7-5, and SlERF10-2 hardly promoted GAL4 gene expression, but they deleted EAR motif significantly enhanced GAL4 gene expression under 100 ng/mL AbA condition (Figure 6B). Besides, SlERF3-4 only interacted with DRE/CRT element, while SlERF7-3 could bind with DRE/CRT and GCC boxes (Figure 6B). Early studies indicated that the expression level of *SlERF10-2* (*LeERF3b*) was markedly increased in low-ethylene tomato fruit containing an ACC oxidase sense-suppression transgene as well as the ethylene insensitive mutant never ripe (Nr) (Ouyang et al., 2016). However, our results showed that *SlERF10-2* was firstly down-regulated in the BR 3 days and then up-regulated in the BR 6–9 days (Figure 7B). A similar trend was also found in the transcriptome data (Figure 7A). In addition, the overexpression of *SlERF10-1* (*SlERF36*) caused early flowering and plants senescence, and affected stomatal density, photosynthesis, and plant growth (Guo and Wang, 2011). In this study, the expression of *SlERF10-1* was only increased in the early BR and subsequently recovered the normal level during fruit ripening (Figure 7B). It suggested that *SlERF10-1* and *SlERF10-2* repressed the expression of genes related to fruit ripening. In addition, the expression of *SlERF4-10*, *SlERF5-8*, and *SlERF7-3* also increased during fruit ripening (Figure 7B). Moreover, *SlERF4-10* and *SlERF5-8* could be induced by ET signal (Figures 4A–C). Expressions of *SlERF2-10*, *SlERF7-2*, and *SlERF12-1* were not affected (Figure 7C), and those of *SlERF2-6*, *SlERF3-4*, *SlERF4-1*, and *SlERF7-5* were suppressed by ET signal during fruit ripening (Figure 7D). Thus, the increase of ET directly promotes *SlERF4-10* and *SlERF5-8* and indirectly induces *SlERF7-3*, *SlERF10-1*, and *SlERF10-2* expression, these SlERF proteins inhibit their target gene transcriptions through binding with the DRE/CRT and/or GCC boxes of target gene promoters and then improve fruit ripening (Figure 8), while others did not regulate ripening or act in some basal metabolism.

Collectively, this work highlighted that there is scope to further understand the balance relationship of EAR motif-containing ERF and other ERF proteins. Our work showed the potential function of ERF and DREB subfamily genes to regulate fruit development and ripening in tomato and demonstrate the inhibition function of EAR motif in tomato ERF proteins. Our findings can further expand our understanding of the function of ERF and DREB subfamily genes in tomato.

## Data Availability Statement

The datasets presented in this study can be found in online repositories. The names of the repository/repositories and accession number(s) can be found in the article/Supplementary Material.

## Author Contributions

LZ, MQ, and YDL conceived and designed this study. LC, SP, QZ, SQ, and YFL carried out genome-wide identification and sequence analysis of the AP2/ERF genes. TX, LZ, and YDL carried out expression analysis of the AP2/ERF genes. LZ, MQ, and YDL drafted and revised the manuscript. All authors read and approved the final manuscript.

## Conflict of Interest

The authors declare that the research was conducted in the absence of any commercial or financial relationships that could be construed as a potential conflict of interest.

## Publisher’s Note

All claims expressed in this article are solely those of the authors and do not necessarily represent those of their affiliated organizations, or those of the publisher, the editors and the reviewers. Any product that may be evaluated in this article, or claim that may be made by its manufacturer, is not guaranteed or endorsed by the publisher.

## Footnotes

1
http://solgenomics.net/organism/solanum_lycopersicum/genome

2
http://pfam.xfam.org/

3
http://smart.embl-heidelberg.de/

4
https://www.ncbi.nlm.nih.gov/cdd

5
http://meme-suite.org/tools/meme

6
https://www.ncbi.nlm.nih.gov/sra/
