# Supplementary material for: Function Analysis of the ERF and DREB Subfamilies in Tomato Fruit Development and Ripening
Source: Front Plant Sci. 2022 Mar 4;13:849048. doi: 10.3389/fpls.2022.849048 (PMC8931701; doi:10.3389/fpls.2022.849048)
Supplement: Supplementary file 2 [file Table_2.DOCX]

**Supplementary Table S2** Accuracy and error analysis of all genes with single AP2 domain in tomato by comparing sequences of DNA, CDS, cDNA, and protein among genome sequencing version 2.0, 3.2, and 4.0

| **Gene name** | **Type** | **Gene ID** | | | **Accuracy** |
| --- | --- | --- | --- | --- | --- |
|  |  | **V2.0** | **V3.2** | **V4.0** |  |
| SlERF1-1 | DREB | Solyc01g005630.1.1 | Solyc01g005630.2.1 | Solyc01g005630.3.1 | 100 |
| SlERF1-2 | ERF | Solyc01g008880.1.1 | Solyc01g008880.2.1 | Solyc01g008880.3.1 | 100 |
| SlERF1-3 | DREB | Solyc01g009440.1.1 | Solyc01g009440.2.1 | Solyc01g009440.3.1 | 100 |
| SlERF1-4 | DREB | Solyc01g014720.1.1 | Solyc01g014720.2.1 | Solyc01g014720.3.1 | 100 |
| SlERF1-5 | DREB | Solyc01g057080.1.1 | Solyc01g057080.1.1 | Solyc01g057080.1.1 | 100 |
| SlERF1-6 | ERF | Solyc01g065980.2.1 | Solyc01g065980.3.1 | Solyc01g065980.4.1 | [1] |
| SlERF1-7 | ERF | Solyc01g067540.1.1 | Solyc01g067540.1.1 | Solyc01g067540.2.1 | [2] |
| SlERF1-8 | ERF | Solyc01g090300.2.1 | Solyc01g090300.2.1 | Solyc01g090300.2.1 | 100 |
| SlERF1-9 | ERF | Solyc01g090310.2.1 | Solyc01g090310.2.1 | Solyc01g090310.3.1 | [3] |
| SlERF1-10 | ERF | Solyc01g090320.2.1 | Solyc01g090320.3.1 | Solyc01g090320.4.1 | [4] |
| SlERF1-11 | ERF | Solyc01g090340.2.1 | Solyc01g090340.2.1 | Solyc01g090340.3.1 | [5] |
| SlERF1-12 | ERF | Solyc01g090370.1.1 | Solyc01g090370.2.1 | Solyc01g090370.3.1 | 100 |
| SlERF1-13 | DREB | Solyc01g090560.2.1 | Solyc01g090560.3.1 | Solyc01g090560.4.1 | [6] |
| SlERF1-14 | DREB | Solyc01g091760.2.1 | Solyc01g091760.2.1 | Solyc01g091760.3.1 | [7] |
| SlERF1-15 | ERF | Solyc01g095500.2.1 | Solyc01g095500.3.1 | Solyc01g095500.3.1 | 100 |
| SlERF1-16 | ERF | Solyc01g108240.2.1 | Solyc01g108240.3.1 | Solyc01g108240.3.1 | [8] |
| SlERF2-1 | ERF | no | Solyc02g030210.3.1 | Solyc02g030210.4.1 | [9] |
| SlERF2-2 | DREB | Solyc02g067020.1.1 | Solyc02g067020.1.1 | Solyc02g067020.1.1 | 100 |
| SlERF2-3 | ERF | Solyc02g077360.1.1 | Solyc02g077360.1.1 | Solyc02g077360.1.1 | 100 |
| SlERF2-4 | ERF | Solyc02g077370.1.1 | Solyc02g077370.1.1 | Solyc02g077370.1.1 | 100 |
| SlERF2-5 | DREB | Solyc02g077810.1.1 | Solyc02g077810.1.1 | Solyc02g077810.1.1 | 100 |
| SlERF2-6 | ERF | Solyc02g077840.1.1 | Solyc02g077840.1.1 | Solyc02g077840.2.1 | [10] |
| SlERF2-7 | ERF | Solyc02g090770.1.1 | Solyc02g090770.1.1 | Solyc02g090770.1.1 | 100 |
| SlERF2-8 | ERF | Solyc02g090790.1.1 | Solyc02g090790.1.1 | Solyc02g090790.1.1 | 100 |
| SlERF2-9 | ERF | Solyc02g090800.1.1 | Solyc02g090800.1.1 | Solyc02g090800.1.1 | 100 |
| SlERF2-10 | DREB | Solyc02g093130.1.1 | Solyc02g093130.2.1 | Solyc02g093130.3.1 | 100 |
| SlERF3-1 | ERF | Solyc03g005500.1.1 | Solyc03g005500.1.1 | Solyc03g005500.1.1 | 100 |
| SlERF3-2 | ERF | Solyc03g005510.1.1 | Solyc03g005510.2.1 | Solyc03g005510.2.1 | 100 |
| SlERF3-3 | ERF | Solyc03g005520.1.1 | Solyc03g005520.1.1 | Solyc03g005520.1.1 | 100 |
| SlERF3-4 | ERF | Solyc03g006320.1.1 | Solyc03g006320.1.1 | Solyc03g006320.1.1 | 100 |
| SlERF3-5 | ERF | Solyc03g007460.1.1 | Solyc03g007460.2.1 | Solyc03g007460.3.1 | 100 |
| SlERF3-6 | DREB | Solyc03g026270.1.1 | Solyc03g026270.3.1 | Solyc03g026270.3.1 | 100 |
| SlERF3-7 | DREB | Solyc03g026280.2.1 | Solyc03g026280.3.1 | Solyc03g026280.3.1 | 100 |
| SlERF3-8 | ERF | Solyc03g093530.1.1 | Solyc03g093530.2.1 | no | [11] |
| SlERF3-9 | ERF | Solyc03g093540.1.1 | Solyc03g093540.1.1 | Solyc03g093540.1.1 | 100 |
| SlERF3-10 | ERF | Solyc03g093550.1.1 | Solyc03g093550.1.1 | Solyc03g093550.1.1 | 100 |
| SlERF3-11 | ERF | Solyc03g093560.1.1 | Solyc03g093560.1.1 | Solyc03g093560.1.1 | 100 |
| SlERF3-12 | ERF | Solyc03g093610.1.1 | Solyc03g093610.1.1 | Solyc03g093610.1.1 | 100 |
| SlERF3-13 | DREB | Solyc03g114440.1.1 | Solyc03g114440.1.1 | Solyc03g114440.1.1 | 100 |
| SlERF3-14 | DREB | Solyc03g116610.2.1 | Solyc03g116610.3.1 | Solyc03g116610.3.1 | 100 |
| SlERF3-15 | DREB | Solyc03g117130.2.1 | Solyc03g117130.3.1 | Solyc03g117130.3.1 | 100 |
| SlERF3-16 | ERF | Solyc03g117230.1.1 | Solyc03g117230.1.1 | Solyc03g117230.1.1 | 100 |
| SlERF3-17 | ERF | Solyc03g118190.2.1 | Solyc03g118190.3.1 | Solyc03g118190.4.1 | 100 |
| SlERF3-18 | ERF | Solyc03g119580.1.1 | Solyc03g119580.1.1 | Solyc03g119580.1.1 | 100 |
| SlERF3-19 | DREB | Solyc03g119800.1.1 | Solyc03g119800.2.1 | Solyc03g119800.3.1 | 100 |
| SlERF3-20 | DREB | Solyc03g120840.1.1 | Solyc03g120840.2.1 | Solyc03g120840.3.1 | 100 |
| SlERF3-21 | ERF | Solyc03g123500.2.1 | Solyc03g123500.4.1 | Solyc03g123500.4.1 | 100 |
| SlERF3-22 | DREB | Solyc03g124110.1.1 | Solyc03g124110.2.1 | Solyc03g124110.2.1 | 100 |
| SlERF4-1 | ERF | Solyc04g007180.1.1 | Solyc04g007170.3.1 | Solyc04g007170.3.1 | [12] |
| SlERF4-2 | ERF | Solyc04g012050.2.1 | Solyc04g012050.3.1 | Solyc04g012050.3.1 | 100 |
| SlERF4-3 | ERF | Solyc04g014530.1.1 | Solyc04g014530.1.1 | Solyc04g014530.1.1 | 100 |
| SlERF4-4 | DREB | Solyc04g050750.1.1 | Solyc04g050750.1.1 | Solyc04g050750.2.1 | [13] |
| SlERF4-5 | ERF | Solyc04g051360.2.1 | Solyc04g051360.3.1 | Solyc04g051360.3.1 | 100 |
| SlERF4-6 | DREB | Solyc04g054910.2.1 | Solyc04g054910.3.1 | Solyc04g054910.4.1 | 100 |
| SlERF4-7 | ERF | Solyc04g071770.2.1 | Solyc04g071770.3.1 | Solyc04g071770.3.1 | 100 |
| SlERF4-8 | ERF | Solyc04g072300.1.1 | Solyc04g072300.1.1 | Solyc04g072300.1.1 | 100 |
| SlERF4-9 | DREB | Solyc04g072900.1.1 | Solyc04g072900.1.1 | Solyc04g072900.1.1 | 100 |
| SlERF4-10 | DREB | Solyc04g078640.1.1 | Solyc04g078640.2.1 | Solyc04g078640.3.1 | 100 |
| SlERF4-11 | DREB | Solyc04g080910.1.1 | Solyc04g080910.1.1 | Solyc04g080910.1.1 | 100 |
| SlERF5-1 | ERF | Solyc05g009250.1.1 | Solyc05g009250.1.1 | Solyc05g009250.1.1 | 100 |
| SlERF5-2 | ERF | Solyc05g009450.1.1 | Solyc05g009450.2.1 | no | [14] |
| SlERF5-3 | ERF | Solyc05g013540.1.1 | Solyc05g013540.1.1 | Solyc05g013540.1.1 | 100 |
| SlERF5-4 | ERF | Solyc05g050790.1.1 | Solyc05g050790.2.1 | Solyc05g050790.3.1 | 100 |
| SlERF5-5 | DREB | Solyc05g050830.1.1 | Solyc05g050830.2.1 | Solyc05g050830.3.1 | 100 |
| SlERF5-6 | ERF | Solyc05g051180.1.1 | Solyc05g051180.2.1 | Solyc05g051180.3.1 | 100 |
| SlERF5-7 | ERF | Solyc05g051200.1.1 | Solyc05g051200.1.1 | Solyc05g051200.1.1 | 100 |
| SlERF5-8 | ERF | Solyc05g052030.1.1 | Solyc05g052030.1.1 | Solyc05g052030.1.1 | 100 |
| SlERF5-9 | ERF | Solyc05g052040.1.1 | Solyc05g052040.1.1 | Solyc05g052040.1.1 | 100 |
| SlERF5-10 | ERF | Solyc05g052050.1.1 | Solyc05g052050.1.1 | Solyc05g052050.1.1 | 100 |
| SlERF5-11 | DREB | Solyc05g052410.1.1 | Solyc05g052410.2.1 | Solyc05g052410.3.1 | 100 |
| SlERF6-1 | DREB | Solyc06g035700.1.1 | Solyc06g035700.1.1 | Solyc06g035700.1.1 | 100 |
| SlERF6-2 | DREB | Solyc06g050520.1.1 | Solyc06g050520.2.1 | Solyc06g050520.3.1 | 100 |
| SlERF6-3 | ERF | Solyc06g051840.1.1 | Solyc06g051840.1.1 | Solyc06g051840.1.1 | 100 |
| SlERF6-4 | DREB | Solyc06g053240.2.1 | Solyc06g053240.3.1 | no | [15] |
| SlERF6-5 | DREB | Solyc06g054630.1.1 | Solyc06g054630.2.1 | Solyc06g054630.3.1 | 100 |
| SlERF6-6 | ERF | Solyc06g063070.2.1 | Solyc06g063070.3.1 | Solyc06g063070.3.1 | 100 |
| SlERF6-7 | DREB | Solyc06g065820.2.1 | Solyc06g065820.3.1 | Solyc06g065820.3.1 | 100 |
| SlERF6-8 | DREB | Solyc06g066540.1.1 | Solyc06g066540.1.1 | Solyc06g066540.1.1 | 100 |
| SlERF6-9 | DREB | Solyc06g068360.2.1 | Solyc06g068360.3.1 | Solyc06g068360.3.1 | 100 |
| SlERF6-10 | ERF | Solyc06g068830.1.1 | Solyc06g068830.2.1 | Solyc06g068830.2.1 | 100 |
| SlERF6-11 | ERF | Solyc06g082590.1.1 | Solyc06g082590.1.1 | Solyc06g082590.1.1 | 100 |
| SlERF7-1 | DREB | Solyc07g042230.1.1 | Solyc07g042230.1.1 | Solyc07g042230.1.1 | 100 |
| SlERF7-2 | ERF | Solyc07g049490.1.1 | Solyc07g049490.2.1 | Solyc07g049490.3.1 | 100 |
| SlERF7-3 | ERF | Solyc07g053740.1.1 | Solyc07g053740.1.1 | Solyc07g053740.1.1 | 100 |
| SlERF7-4 | DREB | Solyc07g054220.1.1 | Solyc07g054220.1.1 | Solyc07g054220.1.1 | 100 |
| SlERF7-5 | ERF | Solyc07g064890.1.1 | Solyc07g064890.1.1 | Solyc07g064890.1.1 | 100 |
| SlERF8-1 | ERF | Solyc08g007230.1.1 | Solyc08g007230.2.1 | Solyc08g007230.3.1 | 100 |
| SlERF8-2 | DREB | Solyc08g007820.1.1 | Solyc08g007820.1.1 | Solyc08g007820.1.1 | 100 |
| SlERF8-3 | DREB | Solyc08g007830.1.1 | Solyc08g007830.1.1 | Solyc08g007830.1.1 | 100 |
| SlERF8-4 | DREB | no | Solyc08g007840.2.1 | Solyc08g007840.3.1 | [16] |
| SlERF8-5 | DREB | Solyc08g008305.1.1 | Solyc08g008305.1.1 | Solyc08g008305.1.1 | 100 |
| SlERF8-6 | DREB | Solyc08g066660.1.1 | Solyc08g066660.1.1 | Solyc08g066660.1.1 | 100 |
| SlERF8-7 | ERF | Solyc08g078170.1.1 | Solyc08g078170.1.1 | Solyc08g078170.1.1 | 100 |
| SlERF8-8 | ERF | Solyc08g078180.1.1 | Solyc08g078180.1.1 | Solyc08g078180.1.1 | 100 |
| SlERF8-9 | ERF | Solyc08g078190.1.1 | Solyc08g078190.1.1 | Solyc08g078190.2.1 | [17] |
| SlERF8-10 | DREB | Solyc08g078410.1.1 | Solyc08g078410.1.1 | Solyc08g078410.2.1 | [18] |
| SlERF8-11 | DREB | Solyc08g078420.1.1 | Solyc08g078420.1.1 | Solyc08g078420.2.1 | [19] |
| SlERF8-12 | DREB | Solyc08g080290.2.1 | Solyc08g080290.3.1 | Solyc08g080290.4.1 | [20] |
| SlERF8-13 | ERF | Solyc08g081960.1.1 | Solyc08g081960.2.1 | Solyc08g081960.3.1 | 100 |
| SlERF8-14 | DREB | Solyc08g082210.2.1 | Solyc08g082210.3.1 | Solyc08g082210.4.1 | [21] |
| SlERF9-1 | DREB | Solyc09g009240.1.1 | Solyc09g009240.1.1 | Solyc09g009240.1.1 | 100 |
| SlERF9-2 | ERF | Solyc09g059510.2.1 | Solyc09g059510.3.1 | Solyc09g059510.3.1 | [22] |
| SlERF9-3 | ERF | Solyc09g066340.1.1 | Solyc09g066340.1.1 | Solyc09g066340.1.1 | 100 |
| SlERF9-4 | ERF | Solyc09g066350.1.1 | Solyc09g066350.1.1 | Solyc09g066350.1.1 | 100 |
| SlERF9-5 | ERF | Solyc09g066360.1.1 | Solyc09g066360.1.1 | Solyc09g066360.1.1 | 100 |
| SlERF9-6 | ERF | Solyc09g075420.2.1 | Solyc09g075420.3.1 | Solyc09g075420.3.1 | 100 |
| SlERF9-7 | ERF | Solyc09g089910.1.1 | Solyc09g089910.1.1 | Solyc09g089910.1.1 | 100 |
| SlERF9-8 | ERF | Solyc09g089920.1.1 | Solyc09g089920.1.1 | Solyc09g089920.1.1 | 100 |
| SlERF9-9 | ERF | Solyc09g089930.1.1 | Solyc09g089930.2.1 | Solyc09g089930.3.1 | 100 |
| SlERF9-10 | DREB | Solyc09g091950.1.1 | Solyc09g091950.1.1 | Solyc09g091950.1.1 | 100 |
| SlERF10-1 | ERF | Solyc10g006130.1.1 | Solyc10g006130.1.1 | Solyc10g006130.1.1 | 100 |
| SlERF10-2 | ERF | Solyc10g009110.1.1 | Solyc10g009110.1.1 | Solyc10g009110.1.1 | 100 |
| SlERF10-3 | ERF | Solyc10g050970.1.1 | Solyc10g050970.1.1 | Solyc10g050970.1.1 | 100 |
| SlERF10-4 | DREB | Solyc10g076370.1.1 | Solyc10g076370.2.1 | Solyc10g076370.3.1 | 100 |
| SlERF10-5 | DREB | Solyc10g076380.1.1 | Solyc10g076380.2.1 | Solyc10g076380.2.1 | [23] |
| SlERF10-6 | DREB | Solyc10g078610.1.1 | Solyc10g078610.1.1 | Solyc10g078610.1.1 | 100 |
| SlERF10-7 | DREB | Solyc10g080310.1.1 | Solyc10g080310.2.1 | Solyc10g080310.3.1 | [24] |
| SlERF10-8 | DREB | Solyc10g080650.1.1 | Solyc10g080650.2.1 | Solyc10g080650.3.1 | 100 |
| SlERF10-9 | DREB | Solyc10g083560.1.1 | Solyc10g083560.1.1 | Solyc10g083560.1.1 | 100 |
| SlERF11-1 | ERF | Solyc11g006050.1.1 | Solyc11g006050.1.1 | Solyc11g006050.1.1 | 100 |
| SlERF11-2 | ERF | Solyc11g011740.1.1 | Solyc11g011740.1.1 | Solyc11g011740.1.1 | 100 |
| SlERF11-3 | ERF | Solyc11g011750.1.1 | Solyc11g011750.1.1 | Solyc11g011750.1.1 | 100 |
| SlERF11-4 | DREB | Solyc11g012980.1.1 | Solyc11g012980.1.1 | Solyc11g012980.1.1 | 100 |
| SlERF11-5 | DREB | Solyc11g042560.1.1 | Solyc11g042560.1.1 | Solyc11g042560.1.1 | 100 |
| SlERF11-6 | DREB | Solyc11g042580.1.1 | Solyc11g042580.1.1 | Solyc11g042580.1.1 | 100 |
| SlERF11-7 | ERF | Solyc11g045680.1.1 | Solyc11g045680.2.1 | Solyc11g045680.3.1 | 100 |
| SlERF11-8 | ERF | Solyc11g045690.1.1 | Solyc11g045690.2.1 | Solyc11g045690.3.1 | [25] |
| SlERF12-1 | ERF | Solyc12g005960.1.1 | Solyc12g005960.2.1 | Solyc12g005960.3.1 | 100 |
| SlERF12-2 | DREB | Solyc12g008350.1.1 | Solyc12g008350.2.1 | Solyc12g008350.3.1 | 100 |
| SlERF12-3 | DREB | Solyc12g009240.1.1 | Solyc12g009240.1.1 | Solyc12g009240.1.1 | 100 |
| SlERF12-4 | DREB | Solyc12g009490.1.1 | Solyc12g009490.2.1 | Solyc12g009490.3.1 | [26] |
| SlERF12-5 | DREB | Solyc12g013660.1.1 | Solyc12g013660.2.1 | Solyc12g013660.3.1 | 100 |
| SlERF12-6 | ERF | Solyc12g038440.1.1 | Solyc12g038440.1.1 | Solyc12g038440.1.1 | 100 |
| SlERF12-7 | ERF | Solyc12g038450.1.1 | Solyc12g038450.1.1 | Solyc12g038450.1.1 | 100 |
| SlERF12-8 | ERF | Solyc12g042210.1.1 | Solyc12g042210.2.1 | Solyc12g042210.2.1 | 100 |
| SlERF12-9 | DREB | Solyc12g044390.1.1 | Solyc12g044390.2.1 | Solyc12g044390.3.1 | 100 |
| SlERF12-10 | ERF | Solyc12g049560.1.1 | Solyc12g049560.2.1 | Solyc12g049560.2.1 | 100 |
| SlERF12-11 | DREB | Solyc12g056430.1.1 | Solyc12g056430.1.1 | Solyc12g056430.1.1 | 100 |
| SlERF12-12 | ERF | Solyc12g056590.1.1 | Solyc12g056590.2.1 | Solyc12g056590.2.1 | [27] |
| SlERF12-13 | DREB | Solyc12g056980.1.1 | Solyc12g056980.1.1 | Solyc12g056980.1.1 | 100 |

[1]: Solyc01g065980 (SlERF1-6)

The initial codon of the gene predicted in v3.0 is wrong, resulting in the translation of the first four amino acids; the protein sequence in v4.0 translates more than 21 amino acids before the initial codon; the protein translation in v2.0 is right.

[2]: Solyc01g067540 (SlERF1-7)

The last amino acid of the protein in v4.0 is not translated; the protein sequences in v3.0 and v2.0 are right.

[3]: Solyc01g090310 (SlERF1-9)

The protein sequence in v4.0 is not translated from the beginning, but there are two amino acids in front; the protein sequences in v3.0 and v2.0 are right.

[4]: Solyc01g090320 (SlERF1-10)

The annotation of the gene in v4.0 is wrong, resulting in the deletion of the sequence; the termination codon of the gene in v2.0 is wrong, and the protein sequence of the gene in v3.0 is right.

[5]: Solyc01g090340 (SlERF1-11)

The protein in v4.0 is not translated from the start codon, the protein sequence in v3.0 and v2.0 is right.

[6]: Solyc01g090560 (SlERF1-13)

The protein translation in v4.0 is wrong, the protein sequence in v3.0 and v2.0 is right.

[7]: Solyc01g091760 (SlERF1-14)

The last two amino acids of the protein in v4.0 are not translated, the protein sequence in v3.0 and v2.0 is right.

[8]: Solyc01g108240 (SlERF1-16)

The protein sequence in v3.0 and v4.0 translates more than 21 amino acids before the initial codon; the protein translation in v2.0 is right.

[9]: Solyc02g030210 (SlERF2-1)

The gene in v4.0 is not ERF; the protein sequence in v3.0 is the same as NCBI XP_004231573.1. Therefore, the protein sequence in v3.0 is right.

[10]: Solyc02g077840 (SlERF2-6)

The protein sequence in v4.0 is right. The protein translation in v2.0 and v3.0 is over termination codon.

[11]: Solyc03g093530 (SlERF3-8)

The gene prediction in v4.0 is error, resulting in the deletion. The protein sequence in v3.0 is right.

[12]: solyc04g007170 (SlERF4-1)

Solyc04g007180.1.1 in v2.0 is the same gene with solyc04g007170.3.1 in v3.0 and v4.0.

[13]: Solyc04g050750 (SlERF4-4)

The protein sequence in v4.0 not translated to the end, the last two amino acids are missing. The protein sequence in v3.0 and v2.0 is right.

[14]: Solyc05g009450 (SlERF5-2)

The annotation of the gene in v4.0 is wrong, resulting in the deletion of the sequence; both ends of protein sequence in v3.0 are wrong, the protein sequence in v2.0 is right.

[15]: Solyc06g053240 (SlERF6-4)

The annotation of the gene in v4.0 is wrong, resulting in the deletion of the sequence; the protein sequence in v2.0 and v3.0 is right.

[16]: Solyc08g007840 (SlERF8-4)

The first 50 amino acids of the predicted protein in v4.0 are deleted, which contained one AP2 domain. The gene is not included in v2.0. The protein sequence in v3.0 is the same as NCBI XP_004244718.1. Therefore, the protein sequence in v3.0 is right.

[17]: Solyc08g078190 (SlERF8-9)

There are two redundant amino acids in front of the starting codon of protein sequence in v4.0, and the translation starting position in v2.0 and v3.0 is wrong.

[18]: Solyc08g078410 (SlERF8-10)

The gene sequences in v2.0, v3.0, and v4.0 are wrong. The gene sequences were again predicted in the genome sequence according to *Solanum pennellii* XP_015084630.2.

[19]: Solyc08g078420 (SlERF8-11)

The gene sequences in v2.0, v3.0, and v4.0 are wrong. The gene sequences were again predicted according to the genome sequence and NCBI data.

[20]: Solyc08g080290 (SlERF8-12)

The protein sequence in v4.0 was translated less of the first start codon. The protein sequence in v2.0 and v3.0 is right.

[21]: Solyc08g082210.4.1 (SlERF8-14)

The 55 bp nucleotide sequences between 753 and 808 bp in CDS region were mistakenly identified as an intron and deleted in v2.0, v3.2, and v4.0, and then leaded to decode. The correct sequences of the gene showed in the XM_004245472 sequences.

[22]: Solyc09g059510 (SlERF9-2)

A sequence in v2.0 is missing, which exists in v3.0 and v4.0.

[23]: Solyc10g076380 (SlERF10-5)

7 amino acids are missed in the end of protein sequences in v2.0, and 159 amino acids were added in the end. The protein sequence in v3.0 and v4.0 is right.

[24]: Solyc10g080310 (SlERF10-7)

Two exons of the gene DNA sequences in v2.0 and v4.0 are deleted as predicted intron. The protein sequence in v3.0 is right.

[25]: Solyc11g045690 (SlERF11-8)

The protein sequence in v2.0 has less one amino acid Q, and that in v3.0 has less 10 amino acids. The protein sequence in v4.0 is right.

[26]: Solyc12g009490 (SlERF12-4)

Four amino acids are missing at the end of protein sequences in v4.0, six amino acids are wrong at the end of protein sequences in v2.0, and the prediction protein in v3.0 is wrong.

[27]: Solyc12g056590 (SlERF12-12)

There is a missing sequence in front of the start codon of protein sequences in v2.0, v3.0, and v4.0, while the gene includes the previous sequences in the NP_001332990.1 sequences.
