# Supplementary material for: Function Analysis of the ERF and DREB Subfamilies in Tomato Fruit Development and Ripening
Source: Front Plant Sci. 2022 Mar 4;13:849048. doi: 10.3389/fpls.2022.849048 (PMC8931701; doi:10.3389/fpls.2022.849048)
Supplement: Supplementary file 3 [file Table_3.DOCX]

**Supplementary Table S3** Protein sequences of all ERF and DREB subfamily members in tomato

> Solyc01g005630 (SlERF1-1_DREB)

MEQQQQQQLQPKHQKQRKFVGVRQRPSGKWVAEIKNTTQKIRMWLGTFDSAEEAARAYDEAACLLRGSNARTNFHNHTPVSPALSMKIRNLINHKKSLNKINHKSTSTSSSSIPTSSTIHQQTQISNDDAYKPDLNFYDQITCSNFDHTSTFANDFDTILLDDENVDNFHDVVPKEIIEDHTQFENMNVERQISTSLYAMNGVNEYWDNFHDSSNNNNNNDWDLPMLYQMFCPS*

> Solyc01g008880 (SlERF1-2)

MCSLMDILNFFFVLFSFNEAMTTKDELKKNEALEKSSKVVRVKKFTKQLGKSSIIMPHVIRIYMQDNDATDSSSDDEENVQGGKSKRNKIICNEIIIEKKNTKVVSKRMSSKKKRDKKLLLENVEKYRGVRRRESGRWAAEIRYGRKEARRRWLGTFDTSREAALAYDKAAIEIKGANALTNILDPPPKESTPSTPCHQ*

> Solyc01g009440 (SlERF1-3_DREB)

MDFEDEQTSSSSSSSDHDIKEKNIDHSSCSMNNNIQFKRRAGRKKFKETRHPLYRGVRKRNGEKWVCEIREPNKKTRIWLGTFTTPELAARAHDVAALALRGSNALLNFPGSAWSLPKAKSSSPQDIQIAVLQVNEELIVSSSPSEEPSCKPKDDEVNSLMEFMDEEAMFNMPIFIDSMAEGMLLTPPAMKRGFNWGDVEEDVEFTLWKD*

> Solyc01g014720 (SlERF1-4_DREB)

MASKDELHENEVVKNQSKDARGNKTNLMGDCSVVLQRVVRIYITDNDATDSSSDEEENHQGENSKRQKRICKEIIIKNGKTNVTSKMVSSKEKNVTKTHQENVKKYRGVRQRKWGSWVAEIRDIRINKRRWLGSFATAYEAALAYDKAAIEIKGPNALTNILKPPPKEIDPINH*

> Solyc01g057080 (SlERF1-5_DREB)

MSSGAKYRGIRKRKWGKWVSEIRVPGSSERLWLGTYTSPEAAAVAHDIAYYCLRPESSSSLHKLNFPSMLPPNVQPGMSPTSVQKVASDAAMAIDAQFLTTPTPAQQHADNYG*

> Solyc01g065980 (SlERF1-6)

MCGGAIISDYDPAGSFYRKLSARDLWAELDPISDYWSSSSSSSTVGKPDSALSPVTHSVDKPNKSDSGKKGNKTVKVEKEKSSGPRPRKNKYRGIRQRPWGKWAAEIRDPQKGVRVWLGTFNTAEDAARAYDEAAKRIRGDKAKLNFPAPSPPAKRQCTSTVAAADTPPALLLESSDNSPLMNFGYDVQYQSQTPYYPMEMPIVSEDYELKEQISNLESFLELEPSDQFSGIVDSDPLNVFLMEDFASTHHQFY*

> Solyc01g067540 (SlERF1-7)

MSSSNSTPISSDNQLPFGMFEPIRTPTGYSWLQRNTALCQPSEKRGRRKQTEPGRFLGVRRRPWGRYAAEIRDPTTKERHWLGTFDTAQEAALAYDRAALSMKGTQARTNFIYTSDSANTNFPSLISPFEHVQNMLNPNNTHFNFNTHINKNTKTKTTTTTNSDESSSYGSSPNENNSFVFLNDDMNNNNNNNTNSGYLLDCIVPDSCLKPPPQQPQEPSSTPQSKMNGEIENTYYDTNGFFDMSSSWESSCELSAMTMNHSSNNMNHQMMDDVADGSYYYPNMEINNYDHLMMMPNHETTNAAAAAAAFGDVEFGYTTLF*

> Solyc01g090300 (SlERF1-8)

MYQQKSTTISDSDLSVLENIKFHLVNDSDFSQILSMFDPINISHADIINSPNSSYGSSTSAAEISWGDMITNIDSPWQCIDKLEHEEAPKEEPLVARGVHAPGDWNRYRGVRRRPWGKFAAEIRDPDRKGARLWLGTYGTPEDAALAYDQAAYKIRGSKARLNFPHLIGSDISEPVRVAPRKRCHSSQSSLVEYTSSKKRKL*

> Solyc01g090310 (SlERF1-9)

MNPSDFSLLQSIQHHLLNDSDFPNIFSAIDSNNTPTDFTQNNFDYGELTPLINSSTTLQANEKSEVEESETVVASVKHAPKDWKRYRGVRRRPWGKFAAEIRDPDKKNARLWLGTYETPEDAALAYDQAAFKIRGSKARLNFPHLIGSGVPEPARVNPRRRSHSPESSCENGTPRKLYFI*

> Solyc01g090320 (SlERF1-10)

MNINMKFSLSDFDFLESVKQHLLNDFDFFKYFSPMNLNNVELPNSAISSLGSSLSIESHEKFEYEEEIIKGPNMVVARQKNTPEDWRRYIGVRRRQWGTFTAEIRDPNKKGARLWLGTYETPEDAALAYDQAAFKIRGSRARVNFPHLIGSNMPKPARLKVRHHTSSLKPSSFSSTSLKNGIRKKKIDLINSIAKTKAKVKLNFFCQTLEKYLPQ*

> Solyc01g090340 (SlERF1-11)

MSIVIDDDEIFSLPSLDELESITHLLYDDDSDFFETLSPMSLDVTTLLPNIPTSNSIESPVTPEETKEPSVACEDAPQDWRRFIGVRRRQWGTFSAEIRDPNRRGARLWLGTYESPRDAALAYDQAAYKIRGTKVRLNFPDLIGSDVPMPPRVTARRRTRSRSRSPEPLTTSSSSSSSSSSSSSSSSENGTKKRKIDLINSIAKSKLLCGMDLQMLIQM*

> Solyc01g090370 (SlERF1-12)

MQGKISLDTEQNFLDTMQHLFNDSDFSQILSEIQTPRQYSTTPDHQNAIAQDNIPILLQEIEENKESTTRLSEHRPKKYKGVRRRPWGKYAAEIRDPERKGCRLWLGTYETPEDAALAYDRTAFRLRGSKAVLNFPHLIETNVTEINRVRPRRRPRSPEFSSSSPPPPPPPPYVDESNNNNTDGSISKRRNVELINSLATVNNLDCQNIMEKYLTSDYYFA*

> Solyc01g090560 (SlERF1-13_DREB)

MEDHNEFSISSSTNSTTSSFTSCCSNNNCLCSTSTTTCSTSSTSSNNCLHESIAKTSSNDHYKCKSKKSAKKNQDGNEEEDNTKIRRKKQCVVDEKHPTYRGVRKRNWGKWVSEIRQPRKKSRIWLGTYPTAEMAARAHDVAALAIKGDSAYLNFPHLADQLPRPASASPKDIQAAAAKAAAASIFLEENSVESSQLTGLHSSHSSTNLASDNVQELLNSPSMDHDDPFFDLPDLIIDRLDQNDRYQYPVSTWQLAGADSGMFRLDEPFLWECY*

> Solyc01g091760 (SlERF1-14_DREB)

MATDLENTKTTITTNTNTTNSSSTEAKKSSITRKFVGVRQRPSGRWVAEIKDSSQRVRLWLGTYDTPEEAAHAYDEAARALRGENARTNFASTTPNSDMDQSNILHSKNGLSFSSIKAKLSKNLQSIMARNSENKSSSSKSITRVSDHFTFARIFHFKNNYDQPYQNHRHVDMNKVVQPSIRVLPHDVTDNNNNNDSSWENSSSVSDCSSEWAAFRQLGLDCDNYGSDGSEYFVGSDPLMAGWMSSPDIMSSTSTNEGSSRSKRFKVSSSVVVPPTFTESPLHDAQNYVPF*

> Solyc01g095500 (SlERF1-15)

MESQKIKKKLVHKTITTKYDHHNKWTPKVVRICYTDCDATDSSSDDDDDERNRVKKYVTEIKFEKKMAAADVRKSLNSNKKKKKAIDLKRDENVKKFRGVRQRPWGKWSAEIRDPVKKTRVWLGTFDTAEEAAMKYNIAAIQLRGADAIINFIETPFPKENAITSVSDYDSTGECENLCSPTSVLRQNNNNNDKDNEDAIAIDTKIMNDESKKMEMDENGFMFDDNLPLMDQSFLKDFFDFRSPSPLMDDVLLPGFSDGMGLLPEVLSIHGNRMLDEDLETCKWANDFFQDVC*

> Solyc01g108240 (SlERF1-16)

MHWLNKRFRQEAGMNSNSNSLQNNNQFQQQQPRLTGDEEYSVMVATLKNVINGNIPTQNYQEFNVFSPYNYSTATTTTNVTSSSSPSTSMSTSFEQVLGVSAEQEPCQFCRIQGCLGCDIFGTTFSSSSSAPAAVAAPVADNKKKSSSSSTATVAIAKKKKKNYRGVRQRPWGKWAAEIRDPRKAARVWLGTFTTAEEAARAYDKAAIEFRGPRAKLNFSFADYTVDTQEQQSTLSSSPQQLPEEPQQSQTANNNSDYGNEIWDQLMGDNEIQDWLTMMNFNGDSSDSGGNVHSF*

> Solyc02g030210 (SlERF2-1)

MASLSSEPTAKTEGSSGNDAGGGETSEAMGHIGTDQLLLYRGLKKAKKERGYTAKERISKMPPCTAGKRSSIYRGVTRHRWTGRYEAHLWDKSTWNQNQNKKGKQVYLGAYDDEEAAARAYDLAALKYWGPGTLINFPVTDYTRDLEEMQNVSREDYLASLRRKSGGFSRGISKYRPLSSRWDLQFGRVPGADYFNSLHYGDNATVDNEYIGGFCMDRKIDLSSYIKWWGGNKARQTDSHLKISEETKVGCPEDIDNELRASELSIQQTEPYEMPRLGVYQENKNHKSSTLSAVSILSQSAAYKSLVEKVAKKKEKVENDENENKSTINRVDRGKMIEKSSPDSGSERLGAAFLNAGGLSINRNLHPLTPLLSGPLLTNYNSIDPLTDPVLWTSIVPNFHTGSSRTAEVHKSEASSDYTLFQQED*

> Solyc02g067020 (SlERF2-2_DREB)

MGRKRKAEGLMKIQRPRKKFVGVRQRPSGRWVAEIKDTIQKIRVWLGTFDTAEEAARAYDEAACLLRGPNTRTNFWTSSSPSSNSALPQKITKLLLSRLREQNKSAAAAAADSSSSTTSLAEIDHQQQKQQEKIGNRVVDFSDSLYTDYLNYPEDNVTENNVIAPITRELTSIVQNQEINFQPVNNYEIIEIGEAINIDVEDIESDIDFQFRFSPFDLAEELSMDFGEETSIVSEAMRRMNYERKFSASLYAFNGITECLKLKMKSGGVTRSDQLSRIQNACKRNLVKERENEERNAGND*

> Solyc02g077360 (SlERF2-3)

MTTHHVENNNQEQDQVACEEILENVWANFISKNDQNSQKVTNEYCCEQYWEQLPILERLPSLGRWISMGAETWEDILNGIIIPSHNNENSNDESTCKDVVNVEKKEEKKKMVHYRGVRRRPWGKYAAEIRDSSRKGARVWLGTFSTAEEAAMAYDKAALRIRGPKAYLNFPHEMVAQAIGISNGPCEKEWTFSSSSQYNSRKRVSRDWNMYENLDEINQLPMEKKIMRSMEEDLFNDLDILEFEDLGSDYLDSLLSSL*

> Solyc02g077370 (SlERF2-4)

MVPTPQSDLPLNENDSQEMVLYEVLNEANALNIPYLPQRNQLLPRNNILRPLQCIGKKYRGVRRRPWGKYAAEIRDSARHGARVWLGTFETAEEAALAYDRAAFRMRGAKALLNFPSEIVNASVSVDKLSLCSNSYTTNNNSDSSLNEVSSGTNDVFESRC*

> Solyc02g077810 (SlERF2-5_DREB)

MAQNSNINVRVGSSEMRYKGVRKLSTGNYIAEIKVTSQRRFVWLGTFDTAEEAARAYDAAARQYLGPRTVINFPPLTNQKDVKRSRDYLQKLNRKDVMINTCASSSGSGETLMPNQSSDQEEFRRFTTVTGVSVMMSMVRDRSLIEALTREGLILPDPEIIDTQPLNLELTLAPPGTMANK*

> Solyc02g077840 (SlERF2-6)

MASTREGHYRGVRKRPWGRYAAEIRDPWKKTRVWLGTFDTPEEAALAYDGAARSLRGAKAKTNFPLLRRRRRSLLPLLLHSISTFHLITGGLPPPDVGS*

> Solyc02g090770 (SlERF2-7)

MDNLSSFTHKLTHQQESHYMVSALKHVFSGAAVDGRADGEAAQLLWEVENAATSDNSMPRRSINVHPVHVELELGKKKQRRRRNTKKEFRGVRQRPWGKWAAEIRDPHKAQRLWLGTFVTAEDAARAYDKKAVEFRGNKAKTNFPLKEYIDDHDNSSPMMKVEEHENDQNVMNGDENNNNGDDFWATLEDDRLVKFITQDMSH*

> Solyc02g090790 (SlERF2-8)

MNRFRQDSLLEENNSYPLAHKLTENQELYYMVSALRHVVSGAGAGDVRANGEAAQLLLEVQNASGGSSSTSANSMPRRSSINVELGKGKKRRRRNMKKEFRGVRQRPWGKWAAEIRDPHKAQRLWLGTFATAEDAARAYDKKAIEFRGEKAKTNFPISEYTARAVNDEINKLASVVMEEERELLVQVLNGGDVAVNGENGSSIQNGDEDDFWETLEDDGLVKWICSA*

> Solyc02g090800 (SlERF2-9)

MTILCEDSLLEQENVSYTLTHKLTQEQENYYMVSALRHVVSGTGDNDEASQLLLEVENASSGNLACRSIDVELEKGKKRRRRNTKKEFRGVRQRPWGKWAAEIRDPHKAQRLWLGTFATAEDAARAYDKKL*

> Solyc02g093130 (SlERF2-10_DREB)

MRKWGKWVAEIREPNKRSRIWLGSYSTPVAAARAYDTAVYYLRGPSARLNFPELLVGDGGLNDLSAASIRKKAIEVGAQVDAVQNSLATHHNHTEEKVHSETASPSELKPCWFQEKPDLNLKPEPEDPEVDYW*

> Solyc03g005500 (SlERF3-1)

MDEVKYRGVRKRPWGKYAAEIRDTNRQGGVRVWLGTFSSAEEAARAYDKAAYNMRGHLAILNFPEEYNLPRSSSHFYNNSSSMPSSSSSSNVHARNDQQGNRQVLELEYLDDHVLEELLDCDQHANNRK*

> Solyc03g005510 (SlERF3-2)

MSMWCVPPKKNLSHIIINMDSSSSSSSSKGKKVIQLEKENSKDSNDLIKYRGVRKRTWGKFAAEIRDPTRQGARQWLGTFDTAEDAARAYDKAAFNLRGHLATLNFPNEYYSKLNDPHYYNYRTSLNVNIPSRSLERGISSIGHQDKEIIEFEYLDDSVLEELLGAEDPKKIIRK*

> Solyc03g005520 (SlERF3-3)

MNSSSNFLYNLNFNTSSLPFNINDSDEMLLYDLLAQADSDTTTLITNSTTMAKPTPSSKEKNYRGVRRRPWGKFAAEIRDSTRNGIRVWLGTFDSAEDAALAYDQAAFSMRGTSAILNFSVERVVESLHEMKFHVEEGCSPIVSLKMRHSMRKRKFNKKNKVSREVVKDDSNYNVNIVVFEDLGVDYLEQLLGSSDHSNSSNDDRGSWWDE*

> Solyc03g006320 (SlERF3-4)

MAPKPKCTTAATAATAAVVKEVHYRGVRKRPWGRYAAEIRDPGKKCRVWLGTFDTAEEAARAYDKAAIEFRGAKAKTNFQMQQQPDDVIRSPSDTSTVESSSAAVAVAKAPAMVESIPLDLSLGSSSSAVGIFSSGVGKFLFQNSPPMYYFQGAGVIRNGGGGGGGSGDGGGAGASGAGGMMKSESDSSTVIDFMGNNFKPKAKFDLNLLPTPEDM*

> Solyc03g007460 (SlERF3-5)

MDYHSLCPIKYTEHRNVIRKVTKPSLVKSKKLSEAAKSSQLNPSVPRTVRISVTDPDATDSSSDEEDLLFGRRRVKKYINEISIETAVKCEVSSGNGKTVNKRAPEPLQTKQKPMKVQPPPSAGAARKFRGVRQRPWGKWAAEIRDPARRVRLWLGTYDTAEEAAMVYDNAAIKLRGPDALTNFSTPAKAEPEPEPEPEPEISALSHSGYESGNESRNIPSPTSVLRCTMSQSESGSGQVHVSEECPSVQGSMECEQTVQPFVQCAAEPLIPSAIPQDVEECQGETSMIPDYSSDYLPTDIPFLNDFFNFDGSAAEQTLLEDSTMTAVTSTTTTTTPNEFGDLCNDSLDFGNDFLFNDADFAEFGSFDDLGDLGMDDFSQDNSVVDYSSVDSLLAI*

> Solyc03g026270 (SlERF3-6_DREB)

MFYSDPRIESCSSFSDSIRANHSDEEVILASNNPKKPAGRKKFRETRHPVYRGVRKRNSGKWVCEVREPNKKTRIWLGTFPTAEMAARAHDVAAIALRGRSACLNFADSAWRLPTPDSSDTKDIQKAAAQAAEIFRPLKSEEEESVVKDQSTTPDDMFFMDEEALFCMPGLLTNMAEGLMVPPPQCTEMGDHVEADDMPLWSYSI*

> Solyc03g026280 (SlERF3-7_DREB)

MNIFETYYSDSLILTESSSSSSSSSFSEEEVILASNNPKKPAGRKKFRETRHPIYRGIRKRNSGKWVCEVREPNKKTRIWLGTFPTAEMAARAHDVAALALRGRSACLNFSDSAWRLPIPASSNSKDIQKAAAQAVEIFRSEEVSGESPETSENVQESSDFVDEEAIFFMPGLLANMAEGLMLPPPQCAEMGDHCVETDAYMITLWNYSI*

> Solyc03g093530 (SlERF3-8)

MISKEEEKLTSVGSNPDRKFSGSGNGSSPADEGTKKQYRGVRRRPWGKYAAEIRDPTWKGSRVWLGTYETDVDAARAYDCLRGRKAILKFPFDAGNLIPQVTIGRKRRIDQ

> Solyc03g093540 (SlERF3-9)

MGSSQEIYTSLDSIREHLLDDDVTFMEYYCSKSCFSFQTSNLDHTSKTEYDGFFEFEAKPHVISSNSPKQSNLRERKPSLNIAIPAKPVVVVENVESEKKHYRGVRQRPWGKFAAEIRDPNRKGTRVWLGTFDTAVDAAKAYDRAAFKLRGSKAILNFPLEVANFKQQNDETKTEMMSSGSKRGRGETEELVIKKEDERVVPMASPLTPSNWSTIWESGDGKGIFDLL*

> Solyc03g093550 (SlERF3-10)

MGSPQEIYTSLDSIREHLLDDDVAFMEYYCSKSCFSFQTSNLDHTSKTEYDGFFKFEAKPHVISSNSPKQSNLRERKPSLNIAIPAKPIVVVENVESEKKKHYRGVRQRPWGKFAAEIRDPNRKGTRVWLGTFDTAVDAAKAYDRAAFKLRGSKAILNFPLEVANFKQQNDETKTEMKSSGSKRMRGETEELVIKKERKLQEERVVPMASPLTPSNWSTIWDGTGIFEVPPLSPLSQLVMI*

> Solyc03g093560 (SlERF3-11)

MGSPQETCTSLDLIRQHLFDESLDQTCFSFETTQTSNLDDIASFFNATSKTEYDGFFEFEAKRHVIHSNSPKQSNLRERKPSLNVAIPAKPVVVVENVEIEKKHYRGVRQRPWGKFAAEIRDPNRKGTRVWLGTFDTAVDAAKAYDRAAFKLRGSKAILNFPLEVANFKQQNDETKTEMKSSGSKRVRGETEELVIKKERKIEEERVLPTAAAPLTPSSWSTIWDEKGIFEVPPLSPLSQLVMI*

> Solyc03g093610 (SlERF3-12)

MYQLPTSTELTFFPAEFPVYCRSSSFSSLMPCLTESWGDLPLKVNDSEDMVIYGFLQDAFSIGWTPSNLTSEEVKLEPREEIEPAMSTSVSPPTVAPAALQPKGRHYRGVRQRPWGKFAAEIRDPAKNGARVWLGTYESAEEAALAYDKAAFRMRGTKALLNFPHRIGLNEPEPVRVTVKRRLSESASSSVSSASESGSPKRRRKGVAAKQAELEVESRGPNVMKVGCQMEQFPVGEQLLVS*

> Solyc03g114440 (SlERF3-13_DREB)

MENSQSPSKSLNNSSKNIQQKINPISSNDGKRFIGVRQRPSGRWVAEIKQTSQKLRLWLGTFDKAEEAAMAYDSAARLLRGKNAKTNFNNHGIFKPNEENYSLLEKNPRLYQLMKHAIMKKFAGKYQNNECLETEEVVLVEESINKEEICAIQLQGSSKVYSSVIVAPSFSNNEKSYQL*

> Solyc03g116610 (SlERF3-14_DREB)

MVQAKKFRGVRQRHWGSWVAEIRHPLLKRRVWLGTFETAEEAARAYDEAAVLMSGRNAKTNFAVQAMDENKKDNYKSNNSTLSGSSSSLSAILSAKLRKSCKSPSPSLTCLRLDTESSNIGVWQKRAGARPDSSWVMTVEFGKKKMINDNEHIIIPDENVTSSSTFLSQDNSIEIEQGKECGVMNEEERMALQMIEELLNRN*

> Solyc03g117130 (SlERF3-15_DREB)

MSRPQQKYRGVRQRHWGSWVSEIRHPSLKTRIWLGTYETSEDAARAYDEAARLMCGSTARTNFPYNATESSRFLSSALIAKLQRCNMSSLTATSRRPGKTRLEDKKENEISTLVRDTGDGEERQSESASQQYMKALEDEHIEQMIEELLDYGSIEMCSVRNE*

> Solyc03g117230 (SlERF3-16)

MYNSLSQKAEPFFFSTQNPLYPNEPNRHHNFSIDNTNYSLDPFWDNYSVSANENFNEKSPVLEGIAAVVGEHVLFGHSNNNNNNQNKNDDPNSSAISILKRTCPEEKKKNNNNVQSVEKSYRGVRKRPWGRWSAEIRDRIGRCRHWLGTFDTPEEAARAYDAAARWLRGSKARTNFQIPPIVPLPTSPTSTSSSSNSSREMKKKNKNGGSIANNQRKCSVVTSAAHLFSSNELSKGVSVTVELDLNLGFRRK*

> Solyc03g118190 (SlERF3-17)

MKRSSSNNDQRDEKDTSNIFPIYSSARSQHDMSAMVSALSQVIGNSSSSASGDSSSVHVNPLTLIQQHQSQSSTQDQERRRYRGVRQRPWGKWAAEIRDPKKAARVWLGTFETAEGAALAYDEAALRFKGNKAKLNFPERVQGQFFQCYDQPATSSNNTSEQNYPNVHHYADLLLRTDNNIDLNFDVSPNTFYHSFDISQSSMEVPVYHEEQQQVITTHEEEEEDFVKYRGSHFGNSTSSGGTK*

> Solyc03g119580 (SlERF3-18)

MDGCISSVRKVRIVYDDPDATDSESDDDQNAARFDKNVNRIKRVVKEIVIPVVSWENDFKKCSKLDNIRIKDSKKIHENKKVQLKSTALPKGVRMRKWGKYAAEIRDPSQGKRIWLGTFETVEAASQAYEAKRAEFDRIISLGKGKNLSPGPAECSMACTSHPTNGKNRVYSHPSPSSVLDVPTSSAAAPVESNENLTRDMARMPDSGSEDFSLSFEDQMLHEFIKQRQGISELIEHPLIEQGSISNSVMEMTEVNIRKKTKARQPTIASCKILTKGTEDYSNDKSIFSVLNEPTIMSPIHTELLHLNIEETAVTGNSLKLLGFDDNALFDKDISQLFDPYADAICLDNTFQCCDGWNECVYCKIFKDEVDLDEVDLRWLDAVLV*

> Solyc03g119800 (SlERF3-19_DREB)

MKPPPPQPNDGIRTRRKPSSRGHPRFVGVRQRPSGRWVAEIKDSLQKVRLWLGTFDTAEDAARAYDQAARTLRGANARTNFELPASDSQQGSSIVENSEPFNFEEACRTEEPENSLVGALKAKLFNSKNSRSFIQAYASNSSSELASKVKPSVPCSEIKKTSTQSEKLPKIGHVKNCSDYLFRGNHNLDYISLMTNDYQPMISSSQCQDNQLYSGTDSAIMWLNEQGTLPWGEPQMSQVQDEVSFDTTININNNINTNIFGGGNSTTATCTWPVSVSEPTVDLSAFGGIDVLNGSIMMPDMNGLTHTSSVTTEQQFLQFENGLWGVGDSAGWDPFLLSSV*

> Solyc03g120840 (SlERF3-20_DREB)

MAEVEVQSSEADSSNTTSSVSSSSSSSLSVNSMHKSVFDSSNKLPEEKKRRTKGTKKQKSINNNSESRHQIYRGVRMRSWGKWVSEIREPRKKSRIWLGTYPTAEMAARAHDVAAVSIKGNSAILNFPHLIDSLPRPLSKSPRDIQAAAALAASMRDPPSSSSSVSSSITTTISAGSEELCEIIELPNLEESDDSKTDLTLSESVEGLLYSPWWADHSTDFCGYFLEQSAAGAGESLISCSFETLKWAC*

> Solyc03g123500 (SlERF3-21)

MCGGSIISDYIDPSRTSRRLTAEFLWGRFDLGKKQKNPNNYHSKAKHLRSEVVDDFEADFQDFKELSDDEDVQVDVKPFAFSASKHSTGSKSLKTVDSDKDAAADKSSKRKRKNQYRGIRQRPWGKWAAEIRDPRKGVRVWLGTFNTAEEAAKAYDIEARRIRGKKAKVNFPDEAPAPASRHTVKVNPQKVLPEESLYSLQSDSAIMNSVEDDHYDSFGFFEEKPMTKQYGYENGSSASADTGFGSFVPSAGGDIYFNSDVGSNSFECSDFGWGEPCSRTPEISSVLSAAIECNEAQFVEDANSQKKLKSCTNNPVADDGNTVTMVPEELPAFEPQMNFFHLPYMEGNWDASGGNFLNTSATQNGGENAMDLWSFDDVPSLMGGIF*

> Solyc03g124110 (SlERF3-22_DREB)

MDIFESYYSNSFVESLLSSSLSISDTNNLNHYSPNEEVIILASNNPKKPAGRKKFRETRHPVYRGIRKRNSGKWVCEVREPNKKTRIWLGTFPTAEMAARAHDVAAIALRGRSACLNFADSVWRLPIPASSNSKDIQKAAAEAAEIFRSEEVSGESPETSENVQESSDFVDEEALFSMPGLLANMAEGLMLPPPQCLEIGDHYVELADVHAYMPLWNYSI*

> Solyc04g007170 (SlERF4-1)

MLKPLSSKFENLGRNMKKKVDTNRLVRKIRIVCNDPDATDDSSDDDSRCKRFVREIKLQIGNSFNLRKASEIECSFQDSNNGEKKTKKEGLVKPLIQPRPAGGLLSKYKGVRQRKWGKWAAEIRDPFKGRRVWLGTYNTAVEASRAYELKRLEFETRAKISRTNVSKQSSGSMVSEYQNQSQNVASGVSDDYAESSVSRTSHSLSSSSVLELDTLTSVSASAPILRLNGPNDNEKVSNVAPLEANVVEQEVPELAMMEETLPLSQIGESMDLDLELESFLIGADDFNQHLDEFVVNDFEDPPVYLIEGDEQLPTGLPDFDDFNFDGYNESFSWMDDAPRTNGTPLNIACP*

> Solyc04g012050 (SlERF4-2)

MCKFKVANYRKKGNYKAIIRDDEEEESGINNNDNINVMFSGNINREEEMSMMVTALTRVITGEDRILNQENNNNNIIGSSSLNEFSGGVGEKRGREEQQYFLSDIISSSKVGEDSSINRTIASNTPTTEATFIYTTPTYDHNNNTNIIDCDQPRRRYRGVRQRPWGKWAAEIRDPYKAARVWLGTFDTAEDAARAYDETALKFRGSKAKLNFPENVRLLPTSSMEKGSLTNTFPSNSVESNLEFLTKKKVLTSTFPAENNMVFSTERQFMTDTFPTEYVPVSSIISPNTSPNTLSTISFFPNSNPIAHTQLSNSNSDDINTQYFLNGDFQRGNSSLSLLNQSMFSTTFQYSSSSPSSSSSSRVPFVPSMYFSGSQPTSQSSENDFRATSSDSAHHPPK*

> Solyc04g014530 (SlERF4-3)

MESSSPKTQYPNFNFFQDQSSLPWNDQHFLDEYLTNIDQNNDHSLPGSTCSFLTSKESYRREVSSSNLHQLPRSWSSSNDTNSSKESNNRHEIEEVTSHHHDKNNSTKHYIGVRKRPWGKYAAEIRDSTRNGIRVWLGTFNTCEEAALAYDQAALTMRGPLALLNFPMDKVRESLENIKYICEDGISPAAVLKATNKMRRVKHKRNRKKRNVLVFEDLGAELLEELLTSTSSN*

> Solyc04g050750 (SlERF4-4_DREB)

MEECGKSRIESVESKRKSRSRKGCMRGKGGPENASCTYRGVRQRTWGKWVAEIREPNGGARIWLGTFNTSVEAARAYDDAARRLYGSDAKLNLSEQESTDVGIIEDGDGECSVLEEASIFKDGNGKYLVWDTPAPSLLGVDFHGDATTCFNWKNQTEMMYF*

> Solyc04g051360 (SlERF4-5)

MCNIVHYKVANSNDNRSSRQDDEGINVFNTMFQGNINREEEMSVMVSALTRVVVGNHPSENIENHHQNNTLISRGVGEKRGRDEVLLHGTNSSHMILSSGGEGSSIRTTREATFIYTNSTNNSIIDESVNNQVRRRYRGVRQRPWGKWAAEIRDPYKAARVWLGTFDTAEGAARAYDEAALTFRGSKAKLNFPENVTLLVPSSIQQPIYSSPDPAISPYRSNFIIGHTSTEVEPILHTNPSNFIEPIAHTSSLYRSNFIERNHHMVQQEPYFQAGSTSGGSDFHQTTNSSNSSIYDHPSSSSG*

> Solyc04g054910 (SlERF4-6_DREB)

MAAAIDVYSSSSNLSDPLTEELMKALEPFMKGILQIQAQIQFQNQQQQLQLLHQQQQSLVPMKQTGATSSQKATKLYRGVRQRHWGKWVAEIRLPKNRTRLWLGTFDTAEEAALAYDKAAYKLRGEFARLNFPHLRHQLNNEFSDFKPLHSSVDAKLQAICQSLANPKSDDSCSKSNSKPRKSKTAAVSVDSNSAQESSSKSEITTDDSLKEEFSYPENGTIKIEASSSSSPPTPSEESSSSSESDITFLDFAEPSFDESENFFLPKYPSVEIDWAAL*

> Solyc04g071770 (SlERF4-7)

MCILKVANQGDSGKYDRIPSTAGDSETTTNEGIPQPYEQSQSFEEMLQQQIQQETEYLMSESANPMYTGYSQSRDMSAMVTALTHVVSGRREAEWGYRPDISGVTTSFGGGGSGSIYSANSPSSSSSGSWAGQKRRRDQEESVTGEQAQRGYGGIGEFKNGESSSSVKLEEDTSLATPQTSSISTTTASQTPPQASEVTGEETGERKRRYRGVRQRPWGKWAAEIRDPHKAARVWLGTFDTAEAAAKAYDDAALRFRGNRAKLNFPENVRLLPQQQQQPTTRLAISTSSSTAAPRFQLMSAASTRSPSPSPFFFQSYNQPPRQSDQQHQNQQQQLFQSSDMARDYWEYSQLLQNPIDFHGGQQSSSLLEQMLLASSLGVLHSHTFPSSSSSSLATSAASSTTSPAYPLFYSAQQSRFFQPQTHQNQSNSSSNSSNFPPPFWTSSGHYPPSSS*

> Solyc04g072300 (SlERF4-8)

MENITNTPKLHVRNLVRRSSRHSTKYHGVRRRPWGRYAAEIRNPNTKQRHWLGTFDTAEEAALAYDISSINFCGIENARTNFVYPFSSFPSPYNNSPAPPSPPPQPLPPPPPSTPELEVVENKCMKIEMNDDINDDDESLVIASILQSFRYTNTLDKLSL*

> Solyc04g072900 (SlERF4-9_DREB)

MNSQIFSTGFSGYGMEQQGSIGLNQLTPIQIQQIQAQINFQNQQQQQQQQMMLQTAHHASTMNFLAPKPVPMKQSGSPPKPTKLYRGVRQRHWGKWVAEIRLPKNRTRLWLGTFDTAEEAALAYDKAAYMLRGDFARLNFPQLRHNGNLIGGDFGEYNPLHSSVDAKLKDICQSLAQGKSIDSKKKKTKGLSAEKAAVVKMEEEESKTAEVGSESDGSHSGSGGSSPVTELIFPEFTEEEPTWDMSENFLLQKYPSHEIDWASL*

> Solyc04g078640 (SlERF4-10_DREB)

MEGEKRKQRQHQQDKPYRGIRMRKWGKWVAEIREPNKRSRIWLGSYSSPVAAARAYDTAVFYLRGPSARLNFPECIVDDHEIHDLSAASIRKKATEVGARVDALQTAIHNSTVNSVESNCNSNSKSTRMMMKPDLNEYPSPESCDEDN*

> Solyc04g080910 (SlERF4-11_DREB)

MSSNSKKLIPATSRKGCMRGKGGPENANCTYKGVRQRTWGKWVAEIREPNRGARLWLGTFDNSYDAAVVYDAAALKLYGAEAKLNLPHLYNNQAQAQAQIQNSKPITIMSPSLSPVSTTTAQASSPSVTSVYNVASPSTWSVGSDDSSFYFNSHDFGIHNDIPSAFNLIDINKTADDYSVDNATNNNQSSEVLGGEMFRDLNMNLPEIDDSSIWEEAKATTSFQEAVNDPGIGGYNLDDDLNFPPWCG*

> Solyc05g009250 (SlERF5-1)

MKSKIKQDCIDENEVIEIERTIRVKISDPDATESSSDDEKQEKRPKIIVHEIVQKKVKIQSFLLNSKNPLDFYQLPPPRVRKRKYQKKGVSAKSGNPPLMADSQNLDLTKVKTRFSLKNRSPLSFEQSSGKLPPMVRKRKSGKFATEIRDPFSKKRIWLGTFNTPEEASEVYQSKKLEFQEKLEKARNANVDKVISAKFELGSSSSSAPPLMADYQNTDSSNESVDRLKKAKNAKENMVISAKFELGSSSSSDPSLIADSQNTDSSNESGDILKKSKNAKEKMVIFANSEPGSSPSEPILMVDEIDEQLNKAINANVAKGISSKSELGSSSSDQVDAQTSDSSNGVEESDEDLWMGQWIQISGLMDDVSGSFVGT*

> Solyc05g009450 (SlERF5-2)

MKMSESRRQSQKSKIREESARPMRKIRIVCDDPEATDSSDDEGVDVSKPKRFVREIYLPVVSSFTLKKVPETESSCQDSNNGDKKRAKTPKTPSGPRPSSSKYRGVRQRKWGKWAAEIRDPFKSRRVWLGTYNTAEEASHAYEMKRLEFEAMAKSNSNTDVSEKSSNNECNNKNHRKVNNAGCVSEEDDSAQSLVSHTSHTSPASVFEMDSLTSGSAAVSEVNNNDKLVVQLVDVDKMGLMEDSLSLADIGARMDFDTEMDMFFAGNDNFDQNLDDFAVNDFEDLPICGLDEQLPAGLPDFDFDFDFDGYNEACAWMDDVAATPLMNGTTTTTPLNIALCP*

> Solyc05g013540 (SlERF5-3)

MEDAMRRLNQETDVPLQNTTNSSTVNKTRSSCSSNKRSLKDTATGPSAVRYRGVRRRPWGRYAAEIRDPQSKERRWLGTFDTAEEAACAYDCAARAMRGVKARTNFVYPPCPTPTQPTSTNDALFNIHSSYKLTSPYYHQSSNTLKDLSNRPFFHSSSPYGSTGRAHVIGQKSNDSLNMLLFRELLSSNSSNNTNNLNVTSSMNMPNLYEQLPNFTMNRNTNSFGSYLPNSSNPVIPSSSVMTTQVPKFDNTVHCTINNNNSSSGATANDDSAAGMDFFPSESSDSGLLEEALNGFFPKPKPIKSVPSSLPNYEFCNIFSQQPQQQEQINNGGLNSDFGLLSSSLSSFPVDYFPGNLQVAPGDNIMGDIFQYPDLLSIFAAKLQNA*

> Solyc05g050790 (SlERF5-4)

MHQENSTQFDLSILDSPLEHQFNGFNLESISFDRSIDHTWEKIDKNEDNIPRSSSQVSNNWKRYRGVRRRPWGKFAAEIRDPKKKGARLWLGTYVTPEDAALAYDRTAFKLKGTRALLNFPHLIDTNVAELNRVRPKQRSRLSEIIKGEEIPSPKRRNPQLINNIAKVNSIIHMFEITSLAFHPQGENK*

> Solyc05g050830 (SlERF5-5_DREB)

MKNQEEIINLKNRHDFISNGLSFPSNIATSPSSSSSSSSFSSNNALVSKKCQSSKKIEEEKKKKKINNDDEDKHPTYRGVRKRSWGKWVSEIREPKKKSRIWLGTYPTAQMAARAHDVAALAIKGCSAHLNFPHLVDQYPHPASTCHKDIQAAAAKAAAIPFPEEDEEEEDQIESDQVELRNCHPSTNLFLENAKESLNSPSREDDDTFFDLPDLSIDVVDQTNSYWCTMSTWQQLIGADTMVYRLDEPFLWE*

> Solyc05g051180 (SlERF5-6)

MILKIQDEKQSSDSVESSISLEDTSSSDTYSFNKENGFHSERPKKEIKHYIGVRARPWGKFAAEIRDSTRNGIRVWLGTFNSAEEAALAYDQVAFLMRGPTTCLNFPVERVSKMLEETEICNFFKNGLSPAAALKEKHKRRSSSNISRKKKQKVNHEEEINNNNNNNNNNVDNNNNNNNNNNNNNNNVFTFEDLGSELLDELLSEYSNSN*

> Solyc05g051200 (SlERF5-7)

MSSPLEIDTSFSHSNLLFLEDESSWSNTHDPFVDIDEYLPIIIPCNDEEIVVESSNTSTTTTTTTTSKVASIQNIHHDQEEVTSIEKKHEDDQEKHYIGVRKRPWGKYASEIRDSTRNGIRVWLGTFDTAEEAALAYDQAALSMRGPWSLLNFPMEHVKKSLENIEYSCKDGLSPAAVLKATHKTRRVKHKRSSRKKKNENLENVFVFQDLGVELLEELLMTSS*

> Solyc05g052030 (SlERF5-8)

MTKQDEGLTLELIRQHLLEDFTTTESFIDSLNSCFSDHISSSDDISPVFTSVKTEPSTSNSLSDSPNSSYPNEPNSPISRYFNLRSDFPEFKIDSDTILSPVFDSSAGSNEDNNKKKNYRGVRRRPWGKFAAEIRDPSRKGSRIWLGTFDTDIDAARAYDCAAFKMRGRKAILNFPLDAGKSGAPANVGRKRRRENKMELV*

> Solyc05g052040 (SlERF5-9)

MDKPHKWLPCSNQEGPKKPPLKLKFSTLYNLHFHSSFSTQFNYQQYFSRKKMDSSSLEMIRQHLLDDVVFMETCSSSSSSSLETTSSTLYSQTSSNSESLESLTSEIKLESNFSVYPDFINTPQSSNLESVSRFFDNSTIEFQAKPQKKRSFNDRKPSLNISIPSVKKTEEPKTGEVKTGEPKTEEPKTGEVKTEYSVKEKMVENSEKKRYRGVRQRPWGKFAAEIRDPTRKGTRVWLGTFDTAMDAAMAYDRAAFRLRGSKAILNFPLEVSNFKQENHEIEKNVVNLNSNTNSCGKRVRGEMENDDGIVMKKEVKREQMVATPLTPSNWSSIWDCGNGKGIFEVPPLSPLSPHSNFGYSQLLVS*

> Solyc05g052050 (SlERF5-10)

MDQQLPPTNFPVDFPVYRRNSSFSRLIPCLTEKWGDLPLKVDDSEDMVIYGLLKDALSVGWSPFNFTAGEVKSEPREEIESSLEFSPSPAETTAAPAAETPKGRHYRGVRQRPWGKFAAEIRDPAKNGARVWLGTYETAEEAAIAYDKAAYRMRGSKAHLNFPHRIGLNEPEPVRVTAKRRASPEPASSSGNGSMKRRRKAVQKCDGEMASRSSVMQVECQIEQLTGVHQLLVI*

> Solyc05g052410 (SlERF5-11_DREB)

MMLPMDYTRKKKSRSRKDAPKNVAETLAKWKEVNEKLDACDDDGRKPVRKVPAKGSKKGCMKGKGGPDNGRCKYRGVRQRTWGKWVAEIREPHRGRRLWLGTFDTAIEAALAYDEAARAMYGPCARLNLPDYYASSKESSKDDSSLPTVSRSDSNTASSFSEVCPAGDMMRGRANVPAARHEDRSIEIDGARTGSNEIGTPLSSLREEAEDETKEVSDKSETFTPLSSLREQAEDEAKQVLDKSETFEIKDEPAACSYDSWDIGQEDLGNFCLDDEMFDVNELLGMMDSTPVDASAPSQDVGFVPPKQEQYAYDPSYQLHSAAYDANQLSNPAYQLDNADDQFSNPLYQLDNAGVDTLEGLQQMEQQSPIEVDYDFDFLRPGRQEDFHFCLDELDVLDF*

> Solyc06g035700 (SlERF6-1_DREB)

MAAYHFNDNSPSLENLSPTGGGSSTRHPNFRGIRQRNGKWVSEIREPRKTTRIWLGTFPIPEMAAVAYDVAALALKGPDAQLNFPDRAYSYPVPASLSAADIRTAAANAAAARAPPLSEINTAAGGGQGQEFVDEEEIFGMPKLLDDMAEAMLVSPPRMHQYDESPENSDADSLWGYP*

> Solyc06g050520 (SlERF6-2_DREB)

MAIMDEAANMVCVPLDYSRKRKSRSRRDRTKNVEETLAKWKEYNEKLDNEGKGKPVRKVPAKGSKKGCMRGKGGPENWRCKYRGVRQRIWGKWVAEIREPKRGSRLWLGTFGTAIEAALAYDDAARAMYGPCARLNLPNYACDSVSWATTSASASASDCTVASGFGEVCPVDGALHEADTPLSSVKDEGTAMDIVEPTSIDEDTLKSGWDCLDKLNMDEMFDVDELLAMLDSTPVFTKDYNSDGKHNNMVSDSQCQEPNAVVDPMTVDYGFDFLKPGRQEDLNFSSDDLAFIDLDSELVV*

> Solyc06g051840 (SlERF6-3)

MDHHSSLCPIKYTEHKRTIRKVTKPSVIKPKKVSDVRKSSEYNPRTVRICVTDPDATDSSSDEDELFGRKRVKRYISEISIESPSVNDVKTLSSGNGKKRVAEGSQAKQKALKGKEVADKTVRKFRGVRQRPWGKWAAEIRDPARRVRLWLGTYDTAEEAAMVYDNAAIKLRGPDALTNFITPPIKEKPEVNVASNSGYESGDESHNLSSPTSVLRFRSSESSEEAEPGLEDIKENCTVLVEEEPNSEHLECQGETLTVIPDYSNDYLPTDVSFLDDYFNFADAEQSLFNDTTSFTNDDLFSSWDFTNDSVLDPEICKFDDSFLDLGALEVDNYFKDIGDFSSVDVLMAL*

> Solyc06g053240 (SlERF6-4_DREB)

MVQSKKFKGVRQRQWGSWVSEIRHPLLKKRIWLGTFETAEEAARAYDEAAILMSGQVAKTNFPIVKESTDNNNDTTKFPLTSSSTLSSSMLNAKLRKCCKDPAPSMTCLRLDNDNCHIGVWQKTSGKHSSSNWITKIELGKKEEKPHDHQDMNAELGITKPLDEENRIAMQMVEELLNWNSPFSDEPITDHLSPSFSNSI*

> Solyc06g054630 (SlERF6-5_DREB)

MENNSTHDQPKFKGVRLRKWGKWVSEVRLPNSRDRIWLGSYDSAEKAARAFDAAQFCLRGPKAKFNFPDSPPDISGGQRLSPAEIQAVAARFANDYSPSVVQEIRRDDHHHDHEGNIGNINSHVINMEKDEISLSTTSCDRVVQMGTSNTVAEMDWAFHNDMMENYSYNASGPPPEYFCDPYYIVGGGGGAGVLDNLSSNLYSPPHFPQRTTPSYDDDDTGNGDDEHYSQQSFLWNF*

> Solyc06g063070 (SlERF6-6)

MCGGAIISDLVPPSRISRRLTADFLWGTSDLNKKKKNPSNYHSKPLRSKFIDLEDEFEADFQHFKDNSDDDDDVKAFGPKSVRSGDSNCEADRSSKRKRKNQYRGIRQRPWGKWAAEIRDPRKGIRVWLGTFNSAEEAARAYDAEARRIRGKKAKVNFPDEAPVSVSRRAIKQNPQKALREETLNTVQPNMTYISNLDGGSDDSFSFFEEKPATKQYGFENVSFTAVDMGLGSVSPSAGTNVYFSSDEASNTFDCSDFGWAEPCARTPEISSVLSEVLETNETHFDDDSRPEKKLKSCSSTSLTVDGNTVNTLSEELSAFESQMKFLQIPYLEGNWDASVDAFLNTSAIQDGGNAMDLWSFDDVPSLMGGAY*

> Solyc06g065820 (SlERF6-7_DREB)

MARAQQRYRGVRQRHWGSWVSEIRHPLLKTRIWLGTFETAEDAARAYDEAARLMCGPRARTNFPYNPNMPQTSSSKLLSTTLTAKLHKCYMASLQMTKTSPQGQKLAKNATNVQESVINSYKMKQQMLVPKPSVLLTHHDHHEEAKVVNLGVGVIRKVEDQVLEGIPQFVKPLEDDHIEQMIEELLDYGSIELCSNVVPSHQIQ*

> Solyc06g066540 (SlERF6-8_DREB)

MSKRIRESDEKGNKHPIYRGVRMRSWGKWVSEIREPRKKSRIWLGTYPTAEMAARAHDVAALSIKKDSSILNFPHLIDSLPRPISLSPRDVQAAAAEAAAMEDLNYVSSTSSVSSIDKITSASEELGEIIELPSLDGSFESEESKTELKISDSVDGWLYPPWWASDGDFDGYLFEQDAVGNSLILSNFEMMK*

> Solyc06g068360 (SlERF6-9_DREB)

MARPQQRFRGVRQRHWGSWVSEIRHPLLKTRIWLGTFETAEDAARAYDEAARLMCGPRARTNFAFNDTDSHSSSSTKYLSAALIAKLQRCQMKSLNMVNNRPGTMKLEDQNDRLSSCGNRGDHGITRRTVQMSVEMPVKYESQTENNNTQEFKSLEDHQIEQMIEELLDYGSIELSSVLQE*

> Solyc06g068830 (SlERF6-10)

MDRIRRGKRRYESEEKEDRNYNHMYSSARSQHDMSTMVAVLSQVIGNKSTTNTNSSSSSSAHHKPLLTLNHQSNTTAAMQNQLPQLNQQQGNNEKRRRQYRGVRQRPWGKWAAEIRDPEKAARVWLGTFHTAEDAAIAYDEAALKFKGNKAKLNFPERVQSTTDQFGISYLITNTNHQQHQFQPTNFLPNSDQLQQHHYSNHNADDLKFGVSPSFYHPTGFNPKALDLVEPSKSSSMTYLVQQASSHQVQEEPRYINHQQEDENNLKFSSYFGTYSSSGPTLGEFEDQK*

> Solyc06g082590 (SlERF6-11)

MTENSVPVIKFTQHIVTTNKHVFSEHNEKSNSELQRVVRIILTDADATDSSDDEGRNTVRRVKRHVTEINLMPSTKSIGDRKRRSVSPDSDVTRRKKFRGVRQRPWGRWAAEIRDPTRGKRVWLGTYDTPEEAAVVYDKAAVKLKGPDAVTNFPVSTTAEVTVTVTETETESVADGGDKSENDVALSPTSVLCDNDFAPFDNLGFCEVDAFGFDVDSLFRLPDFAMTEKYYGDEFGEFDFDDFALEAR*

> Solyc07g042230 (SlERF7-1_DREB)

MVRAKVKKEKKVKGDRYKGVRMRKWGKWVAEVRQPKSRDRIWLGSYDTAEEAARAYDAAVVCLRGPSAMINFPDDPPLISCDADNYKLSPSEIQVKASRHARSSSTRVSEELAAAAATVVVDHHHSRSAAVESVFFRDDLEFGCSSLDHGEVVLHDDLFDSARMWSF*

> Solyc07g049490 (SlERF7-2)

MRHRKSSELKRPGSDLLQQPDADPPRYRGVRKRPWGRFAAEIRDPIKKTRVWLGTFDTAEDAARAYDDAARALRGAKAKTNFNMLPLTDDPYDDEFELFPNPRPASSSMSSTLESSSGPRGGSSSKVTRMKIPRPVRPMEECRSDCDSSSSVVDDRCDVDQTSSFVTKQPLPFDLNLPPPSDNDGVDVDDLHVTALCL*

> Solyc07g053740 (SlERF7-3)

MAVKDKAVKGGNVKVNHGVKEVHYRGVRKRPWGRYAAEIRDPGKKSRVWLGTFDTAEEAAKAYDAAAREFRGPKAKTNFPFPAEMNNVGNNNSQSPCGSSTVESSSGETVVHAPNTRHAPLELDLTRRLGAAAEGGRGGVGYPILHQQPTVAVLPNGQPVLLFDSMWRPGVVSRPYQVVPATMEFAGVGAGVVTSVSDSSSVVEEKHYGKKGLDLDLNLAPPMEV*

> Solyc07g054220 (SlERF7-4_DREB)

MATPPEEPMEFDDNTFERQQRRPVFEEASMSNRRFKKIKSPERQSSVQQPFDHRNNPTPMAFPPPPSSSRLVFPFAFDGTQQSMESSSPLGANAMPLFHPQQQNQQQMISFSPQQCLYPPYFAGELGPSQNQQQMLRYWNETLNLSPRGRMMMMSRLGQDNRGYFRPQQVQVQPISATKLYRGVRQRHWGKWVAEIRLPRNRTRLWLGTFDTAEDAAMAYDREAYKLRGDNAKLNFPEHFIGKDRGETSTEANSSSITTHESSLPEHNSESLQLQTVNNEQLPPSPPPQPPPEGDNHDEDSGIGSSQVTTNSQSSELVWGDMAEAWFNATGWGPGSPVWDDLDTNNNLMFSPNLHFGNFSQQEPHDSDPHQHHDTNSDPSSPSCPMRPFF*

> Solyc07g064890 (SlERF7-5)

MRRSRAAAAARQVPATEVPVPAPVAGEHNGSGGSKEIRFRGVRKRPWGRFAAEIRDPWKKTRVWLGTFDSAEDAARAYDAAARTLRGPKAKTNFPLPSSHHLPPYPHHHQFNQSINPNDPFVDSRLYSQDHPLVSQRPTSSSMSSTVESFSGPRQPPRQQTAASVPSRKYPRSPPVVPDDCHSDCDSSSSVVEDGECDNDNIASSSFRKPLPFDLNLPAPMDDFSADAYADDLHCTALCL*

> Solyc08g007230 (SlERF8-1)

MIFENSEFMNDFELLESIRQHLLEDWDSPVTTVLKPSVKIEPEVSVSSPEMFDFTGFTAPAAAESAAEVKLEVELKTAAVKRTPASSKSMHYRGVRQRPWGKFAAEIRDPAKNGARVWLGTYETAEDAALAYDKAAYRIRGSRALLNFPLRVNSGEPEPVRVGSKRSLTTTSSESFSSSSSENESMMTKKTKKVCQLYT*

> Solyc08g007820 (SlERF8-2_DREB)

MNNDSSLYSSDINPLSATTPSNEEGILLLASSQPKKRAGRKKFKETRHPVYRGVRRRNNNKWVCELREPSQQKRIWLGTYSTPEMAARAHDVAALALRGNLATLNFADSRWGLPVPASKDPKDIRQAAVMAAQAFSQDTELVGVDYMNEEVNSNTIDEIKYQEIDIARANDGSSSDFGAKELCIDMENILCCNWGEDNDMLEMEGWQEKMAKGLLFSPTPRLGSCFSWDDVESDVEVSLWSYSI*

> Solyc08g007830 (SlERF8-3_DREB)

MNSDHFRVQDEGVFLLASSQPKKRAGRKKFKETRHPVYRGVRRRDNNKWVCEVREPSEQKRIWLGTYPTPEMAARAHDVAALALRGNLATLNFADSSWRLTVPISKDPEELRKAAIQAAEELVGVGYMNEEINYIEENIAIGGANGSDNSNLVGNEVNNNNNIQNMEMGNISCYNNWGDNNEMLEIEGSTWQEKMSEGHLFSPTPHYDSCFSWDDVESDGEVSLWSYNM*

> Solyc08g007840 (SlERF8-4_DREB)

MADTSNTTKSRTNTKHHVYRGIRCRSGKWVCEIREPRKTKRIWLGTYPTPQMAAAAYDVAALALKATQNIVLNFPHLVDSYPKLPPSPSPADIQRAAATAAEAMASLGYDDDRSLDRRDGAIGNERGSTEYPSSSGNYDMQIGDAQTTMGEDEYVDEEALFEFPNLVVNMAEAMMLSPPRINSFPSEDYSSGDFDTESLWSY*

> Solyc08g008305 (SlERF8-5_DREB)

MSYTYDEAAQIRSSLSQILLTSSTNTLDSIFSHCQEPKDQMTTTSPVLEPLCSSVYLRQIDLLQKIWEQNRKNITIPTSSTQTQLQESLYSQEFWEQNRANVTIPTTSTQTPLQESLYSQRYVSPNKKKLYRGVRQRHWGKWVAEIRLPQKRMRVWLGTYDNAEAAAYAYDRAACKLRGEYARLNFPNLRDPSELGFGDSAKMNALKNSVDAKIQAICQKVKRGRAKKGIVESEKKVAKDVNLDSSSSSSSLVGSESWSHSDMISQSSSDDGFLNSETCSVIGDCLMGPEYDNCYSTSITPQFERPVLESEFEDYSLATMPSFDPELIWAVLAS*

> Solyc08g066660 (SlERF8-6_DREB)

MNSVAAQDNKNAEIISNKSIKRNRDSSKHPVFRGVRMRNWGKWVSEIREPRKKSRIWLGTFPNPEMAARAHDVAALSIKGNSAILNFPELAGLLPRPATLSPRDIQAAAAKAAAMDKFDDDNNNNNNNNDNTSPNNSTSSTSSNMSTSSSLSSLVSAIDLATSEELTQIIELPKLGTSFELKNDFVFADCSAMETGWLYPEDGGMISTCFDPSLLWNY*

> Solyc08g078170 (SlERF8-7)

MFENIDFENDYAFLQSIKLQLLEDWEWENPLTSSDNSISTYSRNNSIESNTFSNEFDYSTDNLSNDFDCLTDKFLSDLINDNGFGYGSDPVIPNVKSEPEIWNFAEFAAAAEVRVEPPAQSVVTAPPQRLPTARHYRGVRQRPWGKFAAEIRDPAKNGQRVWLGTYETAEDAAFAYDKAAFRMRGSRAMLNFPLRVNSGEPEPIRVRSKKSSMSPECSSSSSDNAPGKRRKKVPQAV*

> Solyc08g078180 (SlERF8-8)

MYSNCELENDFSVLESIRRYLLEDWEAPLTSSENSTSSEFSRSNSIESNMFSNSFDYTPEIFQNDILNEGFGFGFEFETSDFIIPKLESQMSIESPEMWNLPEFVAPLETAAEVKVETPVEMTTTTTKPKAKHYRGVRVRPWGKFAAEIRDPAKNGARVWLGTYETAEDAALAYDKAAFRMRGSRALLNFPLRINSGEPDPVRVGSKRSSMSPEHCSSASSTKRRKKVARGTKQ*

> Solyc08g078190 (SlERF8-9)

MLMNFFALEKIRTHLLGEFSPRTLKFGAELTSSKCSDTVTESCDSESASSGSLPFDFFFDIEADLFQFGAGVSNSSSESANSVYNRTDFVKIESEPSISSSDYEENQSKRFQFKSEPQVFIDLTSPKSRKLCSASDRKPSLKIDLPPVKKYEWIDFGNSAQSNPIVSVPVKQIREAEEKRHYRGVRQRPWGKYAAEIRDPRRRGSRVWLGTFDTAIEAAKAYDKAAFTMRGSKAILNFPLEVGKTLSYSSSAVEGGVKRRREAVETEEEKVVKKCKEEESCPLTPSSWNFVFEQNCNGMFNLPPLSPLSPHLSCSQLIQI*

> Solyc08g078410 (SlERF8-10_DREB)

MEEKHQSPIIFNNYQTISSSSLTKRSISLKSSKQQTNQNKKIKSSTEKTKKMDDSKHPIYHGVRKRSWGKWVSEIREPRKKSRIWLGTFSTAEMAARAHDVAAIAIKGHLALLNFPELAHQFPIPSSKCAKDIQSAAAKAAALNISPRNFMEKQSTDELMEMPCSSEETESAESSLTSNSEDPFLDLPDLLMDLTQKFDKFCYYNSPWDLAGAVSFWPQEELYSWDYNHETETPLEVSQP*

> Solyc08g078420 (SlERF8-11_DREB)

MEEKHQSPIIFNNYQTISSSSLTKRSITLKSSKQQTNQNKKIKSSTEKTKKMDDSKHPIYHGVRKRSWGKWVSEIREPRKKSRIWLGTFSTAEMAARAHDVAAIAIKGHLALLNFPELAHQFPIPSSKCAKDIQSAAAKAAALNISPRNFMEKQSTDELMEMPCSSEETESAESSLTSNSEDPFLDLPDILMDLTQKFDKFCYYNSPWDLAGAESNDSNYSEFWPEEDLCLWDYSLEVTQL*

> Solyc08g080290 (SlERF8-12_DREB)

MADSQNSTKSTQIIQPPNQSSESTKIPFNTTLPPPLPPLPPPPAPPETHIQITNTLSSKLENTNTTPKTVIIPTAIMSSTSSSGLGSPSRSIGGKHAVFRGIRCRSGKWVSEIREPRKTTRIWLGTYPTPEMAAAAYDVAAMALKGNDAVINFPGNINSYPSLPPSPAAADIRKAAATAAALMKIESGETTSGTQPGTDDHRSLGNVHMMETRDHEYIDEEALFDMPNLLVDMAEAMMVSPPRMSSPRSDDSPEHSDAENLWSY*

> Solyc08g081960 (SlERF8-13)

MDQQTMLSSGVKYTEHRKQITMVRPAPPVTFNGRRRSSEMNAAGPRVVRITVTDADATDSSSDEGEQGFYGRQRVRKFVNEVRIEQSSHCNGSVNGVLRNGSSSETAPVVAAAPKRRKKTAGATTAASKLKVNHVKKFRGVRQRPWGKWAAEIRDPLRRVRLWLGTYDTAEEAAMVYDHAAIQLRGPDALTNFATPPATKISCSSYNSGEESHNDQRSPKSVLRCASTSFDESQNNEEAEAESLPILPSDIRDKNDISMSENFCDLPPFESFFPDDLFQFENPITISDLFFEPDTLPDYNNMLFGSSTTDYGFGSSSWPEEDFFQDFGDVFGSDPLVAL*

> Solyc08g082210 (SlERF8-14_DREB)

MDRIQENNSMSSIFDEGAQIRSSLSQLILTSSTNTFDTIFSHCQENNQMSNSPVFEPLGTSVYLRQRDLLQKFCQENIANISIPTTSKTIPFRNSLYTQSYKLPEKKKLYRGVRQRHWGKWVAEIRLPQNRMRVWLGTYETAEAAAYAYDRAAYKLRGEYARLNFPNVRDPSKLGFGDGEKMNAVKNAVDAKIQAICQRVKREKAKKAAKKKSENESKVKETMDSSSSTTTTTMISPSVSEDGLWRSEDSTCSVFGDCLKDPLMESEFDSCSLARMPSFDPELIWEVLAN*

> Solyc09g009240 (SlERF9-1_DREB)

MTTTTYGGKNIIIGGSNNSRTAPNNKSYSGRHPVYRGIRRRKSSGKWVSEIREPKSPNRIWLGTFPTPEMAAIAYDVAAIALKGPEADLNFPNSAASFPVPATSSARDIQTAAARAAAAIGAAGDALLAAAAVHREVVINEKGKLENVEIQNFEFMDEDLIFDMPNVLVNMAEGMLLSPPRFYMHDDDDQTTITYQNLWNYT*

> Solyc09g059510 (SlERF9-2)

MVSIRRRKLLGLCSGRSAFLVPLPKFSENGHFAEHRFFSNRPTSVHPMPSTDIDESKEKIAVKVVPGSSNSHASGSLMEQTAQQFPEVKRRKRHRRKHFENQEPCLMRGVYFKNMKWQAAIKVDKKQIHLGTVGTQEEAARLYDRAAFMCGREPNFELSEEEKQELRQFKWDDFLAFTRSAITNKKTRRRSGAGARRKFEPLTSALNSEEDEEEEEGGEPESNSFSASEDIEHDILFS*

> Solyc09g066340 (SlERF9-3)

MNEDNSTIKKKESCVGEVKYRGVRKRPWGKYAAEIRDTNNNGSRVWLGTYATAEDAARAYDKAAFQMRGRFAVLNFPHEYPSDHHSSSSYSMSSSSTSCSSVKQVIEIEYLDDKLLEELLGLESDKSQYNK*

> Solyc09g066350 (SlERF9-4)

MENEDQRGRRENKYRGIRRRPWGKYAAEIRDPNRNGARLWLGTFETAEEAARAYDRAAFSLRGHQAILNFPNDGHYHINNNTNVPLGVGPSYNTPSTMGNNMNMSSSSSASASASAFSDDRHDHGEKVEFEYLDNNLLDELLASQVHRHDNKRPKF*

> Solyc09g066360 (SlERF9-5)

MDYSSRDDLLFHYNSLPFNVNDTQDMLLYNLVAEGSSQETVNSSSSYGIKEEEVTSYEEERKDKNYRGVRKRPWGKYAAEIRDSTRNGVRVWLGTFDNAEEAALAYDQAAFAMRGSMAILNFPVEIVKESLNEMKCRFDGNCSPVIELKKRYSMRRKSVSRKNRARKDVVVFEDLGAEYLEELLISSESITNW*

> Solyc09g075420 (SlERF9-6)

MCGGAILADIIPPRDRRLSSTDLWPTDFWPISTQNVPLNPKRARPSTGGEQMKKRQRKNLYRGIRQRPWGKWAAEIRDPRKGVRVWLGTFNTAEEAARAYDREARKIRGKKAKVNFPNEDDDHYCYSHPEPPPLNIACDTTVTYNQESNNCYPFYSIENVEPVMEFASYNGIEDGGEEMVKNLNNRVVEEEEKTEDEVQILSDELMAYESLMKFYEIPYVDGQSVAATVNPAAETAVGGGSMELWSFDDVSRLQPSYNVV*

> Solyc09g089910 (SlERF9-7)

MELKLNMEQDPSNERRTNGGGGEIKYRGVRRRPWGKFAAEIRDSARQGARVWLGTFNTAEEAARAYDRAAYSMRGHLAILNFPEEYNLPSSSSHFYSAGSYSSSSMASSSSSSSSRQVLEFEYLDDKLLEELLDCDEEPNKRK*

> Solyc09g089920 (SlERF9-8)

MEGNSSRGKKAMQEKDEVKYRGVRRRPWGKFAAEIRDPTTRPGSRQWLGTFDTAEEAARAYDKAAFNLRGHLATLNFPNEYYNQLSCPPLYYGNNNNNIICSSTNNVSRGKGISSSSSTNYKGREIIELECLDNSVLEELLGVEDPKITKRK*

> Solyc09g089930 (SlERF9-9)

MDSSSSSSQFFYSMNSDLNSSDSSYEWSNFNTQSYLPFNVNDSEEMLLFGVLNAAHEETTSETVTSHRVKEEEVTSESEVIEAIPAKEKSYRGVRRRPWGKFAAEIRDSTRNGVRVWLGTFDSAEDAALAYDQAAFSMRGNSAILNFPVETVRDSLRDMKCHVDDDCSPVVALKKRHSMRKRSTNSKKVNSISKVVREVKMENVNNVVVFEDLGADYLEQLLSSSSSDQSSCDATYFSPW*

> Solyc09g091950 (SlERF9-10_DREB)

MENQSLKSESIMQKELPTCYKQVAIESLSRFFKDPHIFSDLNHEITFINSSPTKFSEFLPLNFHQNESSLSLSSSSSSLSTSSSYQHHQDQGNNNNIFPLNNFLDHQTCASSTNSLSSNYPTLGLFLQEPSILEISKRAAESLSNKNHKSSSAALFPMSSSIGHESQIHYKEVATTNPTNWLKMNQTITNCTTTKGFSDYWLSTTKTQPMKFKGSSRKSSLVHYEKSSSSSSSSSSSSSMIASQGKLFRGVRQRHWGKWVAEIRLPRNRTRVWLGTFDTAEEAAFAYDTAAYMLRGDYANLNFPHLKNQLKANSINGNITSLLEAKIRAISTQSKKKANNSSDRDEIIISPKGLSDDDATIKNTIVQNQEKEINCEKLMEITKVKNINNQENVDGVQLSRMPSLDMDMIWDALLVSDLS*

> Solyc10g006130 (SlERF10-1)

MRRGRATPAAAAAAVKPDGSGGLKEIRFRGVRKRPWGRFAAEIRDPWKKTRVWLGTFDSAEDAAKAYDAAARTLRGPKAKTNFPLPMYSQHHQFNRSLNPNDRLVDPRLYSQEAPIICQRPTSSSMSSTVESFSGPRPPRQQTAVLPSRKHPRSPPVEPDDCRSDCDSSSSVVEDGDCEGGNDNIVSSSLRNPLPFDLNFPPPMDDVYANSNDLYCTALCL*

> Solyc10g009110 (SlERF10-2)

MAPKEKIGAVTAMAMVNLNGISKEVHYRGVRKRPWGRYAAEIRDPGKKSRVWLGTFDTAEEAARAYDNAAREFRGAKAKTNFPKLEMEKEEDLKFAVKNEINRSPSQTSTVESSSPVMVDSSSPLDLSLCGSIGGFNHHTVKFPSSGGGFTGSVQAVNHMYYIEALARAGVIKLETNRKKTVDYLGGGDSDSSTVIDFMRVDVKSTTAGLNLDLNFPPPENM*

> Solyc10g050970 (SlERF10-3)

MQRSTKLSGEEEFSIMVAALTNVITGAATISSFPCTSGMSPPLFRVPEETERCQYCKINGCLGCNYFATSSAAAGVVNNNKALKIVGKTKKKKKNYRGVRQRPWGKWAAEIRDPRRAARVWLGTFTTAEDAARAYDRAAIEFRGPRAKLNFSFTDYTSIQQHNTTTPMQVLQQQQPAPSQLQQGINTEEEEFWDQLMNSDNEIQHYLYRESSDSANGYIAHSF*

> Solyc10g076370 (SlERF10-4_DREB)

MDFYDNRKVKRRRNGSDSIEEILLRWKNFNQEVNWNHEQVKKKRKSPGNGSNKGCMPGKGGPENSGCKYRGVRQRTWGKWVAEIREPVYISGQYKSKGKRLWLGTYSTAGDAAVAYDEAAKVMYGSNAILNFPNSSNGNITRTSSGQSSIDHEESVVDDEKKTEIESDLKDDDGGVVVNMDLSYDYANHGSPACSWTEEELEVITEENSEIELTNLECDSRFFHKSHVKVERPIMEEEIDEDEFVHNDVSNTIDVEPTVMFSKDDFSRLDETRNSNDQIVLQDMDFRSSENLNEDVSTRLEYMEHFLMDDNCSMEAANISDIICLTENHDEAFDFQRFLEESFDFELNYAKNEEQFDCTYAYNQQIDHQNSETNFEIRSDGIRKEKNLHGFGLDDFGASNNQRKI*

> Solyc10g076380 (SlERF10-5_DREB)

MQGKGGPENSSCKYRGVRQRTWGKWVAEIREPAYISGDNKSKGKRLWLGSFDSADEAAIAYDEAAKVMYGSNATLNFPNYSSNGSITRTSSLELSGQSCVDHEDLVLDGSKNDEIESDLKTSDTPNTDLSYDYVNHGSPACSWNEEDLEVIMEENSKNELIDSECNSEIFT*

> Solyc10g078610 (SlERF10-6_DREB)

MEINFQTHHHQKTEISMKKVTKSIKGRRRRSNNSNINSNSNKFVGVRQRSSGKWIAEIKDTTHKKIRMWLGTFETAEDAARAYDQAAFLLRGSNTRTNFVTTRVSADSQLAYRIKNLIDSKKIAKQNKQGNSVFGSTCSEKIQESHEKVLNSSDDKTFYNKPTTIANPMTQSQTSCDRLYEPSLTTCHHLNSSEAEDNDFYKPDMMSCSSSCSSQPQVSWGFEFAEELLDFRVMDESISEMGCTEFERMKVERQISASLYAVNGVQEYMENVHDIDDLSLWDLPPLCSLLS*

> Solyc10g080310 (SlERF10-7_DREB)

MDSYKRNPLKPWKKGPARGKGGPQNALCEYRGVRQRTWGKWVAEIREPKKRTRLWLGSFATAEEAAMAYDEAARRLYGPEAYLNLPHMRANFNPLNKSQKFKWFSSTNNFVSSLIPNTTGLLNLSAQPNVHEKEDEFSSHNNVAVRGIEDHEVAAEEANSFYNVNDELMFPSSIWNF

> Solyc10g080650 (SlERF10-8_DREB)

MEINFQKHQQKEAISTKFKGRKRSKGPNSFVGVRQRPSGKWIAEIKDTTQKIRMWLGTYETAEEAARAYDQAAVLLRGSNTRTNFLTTRVSHDSPLASRIRNLLNIKKTANEKSLDCLPDSTSSSSTNPISSENAEKSNLYEKLLSHDHESQFFDSNSVQSNTTSVTSLNTSNFSDPMSSENAKNKFEVMKVERQISASLYAVNGVQDYMQISQVSILGPVGYGPTTLPLRHSDLLILFLIFNLFYTMK*

> Solyc10g083560 (SlERF10-9_DREB)

MSGANNGSGSGSGSESGRHPVYKGIRRRKSSGKWVSEIREPRSPNRIWLGTFPTPEMAAIAYDVAALALKGRDTELNFPNSAPSLPVPATNSPRDIQTAAACAAAAIGAAGDALIGGRSNNSNSNSNSRTVSRDVEDNYLIPNNNEYNFMDEDLIFDMPNVIMNMAEGMLLSPPRLNHLPPDDDYIGGADQNLWNYP*

> Solyc11g006050 (SlERF11-1)

MDSSSSSSSYSYSQYHDHDLGFNENDTEDMLLLNVLSEAKEDSSSNNIHEETNEGSSKEVATSYRGVRRRPWGKYAAEIRDSTRNSVRVWLGTFDTAEEAALAYDQAAYALHGRVAVLNYPVEMVYRSLMEIDCRFEDGCSPVLALKKWHSMMKKGNNKSSSSNKKKEEKEMSYENVLVLEDLGADYLEEILMLSESNS*

> Solyc11g011740 (SlERF11-2)

MNHSFSIETSCYEEIKIKDEEEIIINFDNNIQDEENTQKGKQYIGVRKRPWGKYAAEIRDSTRNGIRVWLGTFDTAEEAALAYDQAALSMRGPTTCLNFGVEIVQESLRGFKYTSQEGSTSSPAQVIKEKHKKRNSKGKKTIKSNNQKNNNNVLVLEDLGTDLLDQLLSTKCSTSE*

> Solyc11g011750 (SlERF11-3)

MDLKIEDHDEKLISNIKVEVEVEVEDKKKHYIGVRKRPWGKYAAEIRDSTRNGIRVWLGTFDTAEEAALTYDQAAFCMRGPLTCLNFSVDKVRESLTKMEFINSKDGLFMSSPAAALKEKHKKRNGTTSSRKKKNSIKEENVLIFEDLGPDLLDELLSSEYSSCSN*

> Solyc11g012980 (SlERF11-4_DREB)

MVKTEQKSLSMSISSIATSNNNNMKTKKNKYKGVRMRSWGSWVSEIRAPNQKTRIWLGSYSTPEAAARAYDAALLCLKGPSASSNLNFPFNSTFYHHIDHHTCTTLSPKAIQRVAAAAAATPEQQNVGLVLSDNNNSTNPTISSPPQSSTSSSEDINDGCLLSNINTFDQEMSMIEQWYNFDSPKYNDMLHGTIFFDPPSMEEVYYEESSADIPLWSFC*

> Solyc11g042560 (SlERF11-5_DREB)

MENDQESKNNMDVYRGVRKRKWGKWVSEIREPGKKTRIWLGSYQKAEMAAAAYDVAAFHLKGERPNLRLNFPELIHTFPKPSSSRPEDVQMAAHEAAMRFKPSIDDHPEECGVGQVRVGLSPSQIQAINESPLDSPKMWMELAGALLPVREYTCPTDYFEDETPHHHESIWDF*

> Solyc11g042580 (SlERF11-6_DREB)

MSGITSEYRGVRKRKWGKWVSEIREPGKKTRIWLGSFETAQMAAAAYDSAAFHFRGHAAKLNFPELIEHFPKPASSSPEDIRLAAQQAAITVQNTNSMASSSHGHGAPVTVGLSPTEIQAINDFPMDSPHMWTTHEDINYTYEMSDYQEMDDCLWDYSSTNSF*

> Solyc11g045680 (SlERF11-7)

MDLIKSSTSSSTSKSTKPRKNNGTSNQSVKQKTEEQQSNFRYLGVRRRPWGRYAAEIRDPNTKERHWLGTFDTAEEAALAYDRAARSMRVNNKSNKPTRTNFVYSDMPHGYSVTCIVSPDDQYQHHHHHQQQQLLIFGQTENNPAPNVDYGAHFSHFSLSNMNNIGGDSYDDGEFALQHYCNPNYDVHMEDGYRYDSNTTSTELPPLPEDITSSGYYYNLSSEFPYSEMGYNSKNEMIIGTNNEYQYGASTITTTSSTTTTAGSFNYFGFDDCLQPLQDESNNNRLGYFFS*

> Solyc11g045690 (SlERF11-8)

MDLIKSSTSSSTSTSRKPRKNNGTSNQSVKQKTEEQQSNFRYLGVRRRPWGRYAAEIRDPNTKERHWLGTFDTAEEAALSYDRAARSMRVNNKSNKPNRTNFVYSDMPHGYSVTCIISPDDQYQHHHHHHHQQQQQQQQQQQQQQQQQQHLLVFDQTENAPAPNADYGAHFSQFSLSNMNNVGGDSCDGVEFVSQQYYNPNYDMHMEDCYRYNSKNKTTTELPPLPEDITSSGNYYNLNSEFPNSEMGYDSKNEMIIGTNNEYGASTMTTTTAGNFSYFGFDDCLQPLQDDPSNNNSLGYWFS*

> Solyc12g005960 (SlERF12-1)

MPRRKNPEINGSRSVVFQQSVIEPPRYRGVRKRPWGRFSAEIRDPVKKARKWLGTFDTAEGAARAYDTAARSFHGAKAKTNFPVLPPYGENQFENLQQSRPASSSMSSTVESSSDARALCKTMQPRIEIPRRIPMGEGHSDCDSSSSVVDGNCIGGGGGDDDQTSSFCKDPLPFDLNFPPPSDDFQIITPLCF*

> Solyc12g008350 (SlERF12-2_DREB)

MIIMSTEQPNCSESTESSCNSSSSSSPSSPSSVLLQPLPQINSKNRLKRCRGEEEVEEEEDVVVNNPNPKKMNKNNNNGSSTSVVSYVGVRMRAWGKWVSEIREPKKKSRIWLGTFATPEMAARAHDVAAMSIKGTSAILNFPQFSHLLPRPVTCSPRDIQNAAVKAAHMDHLNPKFSILPETSAATMTSSSSSLSLVSGVTSSSSSFQDDEESRPSPPELIPEATGQLSEIVELPKLGSSYELVESTQSLFESDEWWDNNYGNCEYFFGQDNYISSNMEFTGLENVVSTSFESFLWQH*

> Solyc12g009240 (SlERF12-3_DREB)

MVKPNSKDTEFSQSSSSSLYRGVRKRKWGKWVSEIRLPNSRERIWLGSYDTPEKAAKAFDAALFCLRGKGANFNFPENPPEIRNGRTMTPSEIQSAAAQFANNTEPELIRVGPRENSDLSSSSSEIFRAESPSVSVSDRVESEKTEITLGNDFIDVYRVESRVESEKTEMSLDNGFVDMFSSLGTVNDMSDFGIFPGFDDLSGEFFIPPPSSPQPSPLQMPNLESLEEENYLNYDGFQSQGTSFLWNF*

> Solyc12g009490 (SlERF12-4_DREB)

MVHSKKFRGVRQRHWGSWVSEIRHPLLKRRVWLGTFDTAEEAARAYDEAAILMSGRNAKTNFPITQDLDNNNNNNNNNNNNNNNNKNVKGKDQESSSSSSFSSPKALSEILHAKLRKCSKVPSPSLTCLRLDIENSHIGVWQKRAGPSSDSKWVMTVELQKKNNPKNINVHEGELNNNNNNNNNDNNNNSKNSCGEITIRSEMDEEERIALQMIEELLQR*

> Solyc12g013660 (SlERF12-5_DREB)

MKNIESIDNLQSIMSMYRPLKKLKISSSSQHNSLRSSGDGVEPVAQRIRARGYEPQFRFPFALDDPPLSSCHINWQSLTQNQTMISFAPQSWFNQCRIIDNGTRTTSMMNVYGTVSSPASKRYRGVRQRHWGKWVAEIRLPRKRTRLWLGTFESAEEAAFAYDVEAFRLRGTDARLNFPHLFLGDLAPFDNNINVTNSASPSTTSFAKNPFQIQHNKRFIRQCRNKKIVPSAVEQSTSVFDKEIMMQDKAAYNKPLWYDPSQDSVHNESLVHETSQTMMFDGSDFYEPTPKYTNNIQQENSDMWKNVLQPTPFGNYSICTDDNLQQQKQQCDFTITDPGFGDNVFDFSSKLLG*

> Solyc12g038440 (SlERF12-6)

MAFPPNDVLYKSVRNRPWETYGVEITNPIKKVHVWLGNFKTAEEAARDFDEAAKMYHDPNAKLNFPPTNEDRFQNSNNFET*

> Solyc12g038450 (SlERF12-7)

MAKKRNDVKATTLAVPPNEVRYKGVRKRPWGTYGAEITNPIKKVRVWLGTFKTAEEAARAFDEAAKIYHGPNAKLNFAPTNEDNLQNTNNFET*

> Solyc12g042210 (SlERF12-8)

MVDRRYGKRPFSSNEWEEKEDINFPIYSARSQHDMSAMVSVLSQVISNTNNTTNISSSSSMHEIDPLTLPQPTTNQIHEQGNQQRKRHYRGVRQRPWGKWAAEIRDPKKAARVWLGTFDTAEAAALAYDEAALRFKGNKAKLNFPERVQSGNTQYLTTTHQQQQQHYAFNNNNNNIPQMVTQPNLYQQHFPNVHHYAQLLRDGSNNNIDNMMNFGVSDQSSFYHHHQQGNFISPNTSLELQQQQQQASYYHNQQEDFLRYTMDFGNSSNYSTGPPSESNWMDFEPKK*

> Solyc12g044390 (SlERF12-9_DREB)

MAELVQSSSISVSKTEEKEKRTRDETYPVYRGVRMRSWGKWVSEIRQPRKKSRIWLGTYPTPEMAARAHDVAALSIKGNSAILNFPQLVESLPRPASISPRDVQAAAAMDELNSSVSSTLRHSESMETEDQLGQIIEIYKVKF*

> Solyc12g049560 (SlERF12-10)

MCGGAIISDWIPPSRSSSRLTADQLWGCADLQNKKRNKKKRNPSNYHSKRLRSENVDFEADFQDFKDFSDDEEAYSLDIKPFAFSASELSGTSAGSESLISVDANKEVEKSAKRQRKNQYRGIRKRPWGKWAAEIRDPQKGVRVWIGTFNTAEEAARAYDAEARRIRGNKAKVNFPDEASVPASRQAGKVNPRKVLSDESSNPVPPNTMLMNNLNSGYCDNVGLLEEKTKTLNGYEALCVTPVDTGPNPYPHPAAAGVYFNSDQGSNSFGPSDFWGETCSRTPDISSVLSAAIECDEAQFIEGVDLEEKPKSCTNNLVPNNVNTEHKPPEVFSTFESQLKFYQTPYSEGNMDVPVDAFLDADATQGVENAMDLWSFDELSSLMGGI*

> Solyc12g056430 (SlERF12-11_DREB)

MNNSSNSCSSIPSDEGSIILESSNKSKKRAAGRKKFKETRHPVFRGVRKRDNDKWVCEIREPTKQKRIWLGTYLTAEMAARAHDVAALALKGELAKLNFADSAWRLQVPVSKDPKELRQAAARAVEAFENVREVAPQENNIVVDCNMMVDENNNSNSCGIEEWVANMEEESLFSPNPCLFGSHFNWDDHVESDVEVSLWNYTI*

> Solyc12g056590 (SlERF12-12)

MCFLKVANSRKSSEFVRFTDTDDTQTTAVTAIGGGVEGGGQFDYSMYSGYCDSQARDMSEMVTEFTRVVSGQDYRPDTRCYSVNSPSPAYSSSSSGSRAGLKRSRDQQEFGTGLSSSSSVKIEEATSMVAPIPAFTTTITTTTTTGEGSSEETGGDRRRKYRGVRQRPWGKWAAEIRDPHKAARVWLGTFDTAEAAARAYDEAALRFRGNRAKLNFPENARLSSLPQTQNTVTSTISNPSPLIAQPTSFLNPIQSSDTTRDYWEYSQLLQNPGDFTDQQPSNLLEQMFVASSMAMLHSNTLPLISSSSSLATSATSSTSYPLLFSSYYTPQTNQIQGTNTSSTSTTSSSSFSTTFWSSSSQYPPSSS*

> Solyc12g056980 (SlERF12-13_DREB)

MATSTMDFWTTTLLDLNSSNSGGELMEALAPFIKSASSPSPSPSVSPSFDLQSSSLSTSFLYESFSSTSQPNMSSIGLNQAQIYLSQQVMPAVTFQNNNQYASYLGPKPVSMKQTGSPPKPPKLYRGVRQRHWGKWVAEIRLPKNRTRLWLGTFDTAEEAALAYDKAAYKLRGEFARLNFPHLRHNGSLIGSEFGEYKPLHSSVNAKLQAICQDLAQGKSIDTKKKRKVSSKAMMVEVEEKEYKKSKTTAEAGSESDGSGSGSGSGSGSGSSPISEYTFDSIWDMCSENYVLHKDPSQEIFNWASLL*
