# Supplementary material for: Function Analysis of the ERF and DREB Subfamilies in Tomato Fruit Development and Ripening
Source: Front Plant Sci. 2022 Mar 4;13:849048. doi: 10.3389/fpls.2022.849048 (PMC8931701; doi:10.3389/fpls.2022.849048)
Supplement: Supplementary file 4 [file Table_4.DOCX]

**Supplementary Table S4** CDS sequences of all ERF and DREB subfamily members in tomato

>Solyc01g005630.3.1 (SlERF1-1_DREB)

ATGGAACAACAACAACAACAACAACTACAACCTAAACATCAAAAACAAAGAAAATTTGTTGGTGTAAGACAAAGACCTTCTGGAAAATGGGTTGCTGAGATCAAGAACACAACACAAAAGATTAGAATGTGGCTCGGAACGTTTGATTCTGCTGAAGAAGCTGCTCGAGCCTACGATGAAGCTGCTTGTCTCCTTCGAGGCTCAAATGCTCGAACGAATTTCCATAACCATACCCCTGTTAGCCCTGCTCTTTCTATGAAAATTAGAAACCTAATCAACCATAAGAAAAGTCTCAACAAAATCAATCACAAAAGTACTAGTACTAGTAGCAGCAGTATTCCGACTTCATCAACTATTCATCAACAAACTCAAATATCGAATGATGATGCATATAAGCCGGATTTGAACTTCTATGATCAAATAACATGTTCAAATTTCGATCATACATCAACATTTGCAAATGATTTTGACACGATTCTTCTTGACGATGAAAATGTTGATAACTTTCATGACGTAGTACCAAAGGAGATTATCGAAGATCATACTCAATTCGAAAATATGAATGTTGAAAGACAAATATCAACATCACTATATGCTATGAATGGAGTCAATGAATATTGGGACAATTTTCATGATAGTAGTAATAATAATAATAATAATGATTGGGATCTCCCTATGCTCTACCAAATGTTTTGTCCAAGTTAG

>Solyc01g008880.3.1 (SlERF1-2)

ATGTGTAGTCTAATGGACATTTTGAACTTTTTTTTTGTTTTGTTTTCTTTTAATGAAGCTATGACCACCAAGGATGAGCTAAAGAAGAATGAGGCACTCGAAAAATCCTCAAAAGTTGTCAGAGTAAAAAAGTTCACCAAACAATTAGGTAAAAGTTCTATTATCATGCCGCATGTTATACGTATTTATATGCAAGATAATGACGCCACAGACTCATCTAGTGATGATGAGGAAAATGTTCAAGGAGGAAAGTCCAAGCGCAATAAAATAATATGTAACGAAATCATCATTGAAAAGAAAAACACCAAGGTCGTTTCAAAGAGGATGTCGTCCAAAAAGAAGAGAGATAAGAAACTACTTCTAGAAAATGTGGAGAAGTACAGGGGAGTTAGACGGCGAGAATCTGGAAGGTGGGCAGCAGAGATTCGATATGGAAGAAAGGAAGCTCGACGACGTTGGTTGGGAACTTTTGATACTTCTAGAGAAGCTGCTTTGGCTTATGACAAGGCAGCTATCGAAATAAAAGGTGCTAATGCTTTAACAAATATTCTCGATCCACCACCAAAGGAATCAACCCCATCAACACCGTGCCACCAATAA

>Solyc01g009440.3.1 (SlERF1-3_DREB)

ATGGATTTTGAAGATGAACAAACTTCTTCTTCATCATCTTCTTCCGATCATGATATTAAAGAAAAAAACATTGATCATTCATCGTGTTCGATGAATAATAATATACAATTCAAGAGGAGGGCAGGGAGGAAAAAGTTTAAGGAAACTCGACATCCATTATATAGAGGAGTTAGGAAGAGAAATGGAGAAAAGTGGGTGTGTGAAATACGCGAGCCAAATAAAAAAACAAGAATTTGGTTAGGTACTTTCACTACTCCTGAATTAGCAGCTAGGGCACACGATGTTGCTGCCCTAGCTCTCCGGGGAAGTAATGCTCTTCTTAACTTCCCCGGATCAGCTTGGTCTCTACCTAAAGCTAAATCTTCCTCCCCTCAAGACATTCAAATCGCGGTTCTCCAAGTTAATGAAGAATTGATTGTGTCGTCCTCACCCTCAGAAGAACCTTCATGTAAACCAAAAGATGATGAAGTGAATTCTTTAATGGAGTTTATGGATGAGGAGGCCATGTTTAATATGCCAATATTTATTGATAGCATGGCTGAGGGGATGCTTTTGACTCCACCAGCTATGAAAAGAGGCTTTAATTGGGGTGATGTTGAGGAAGATGTTGAGTTCACTTTGTGGAAAGATTGA

>Solyc01g014720.3.1 (SlERF1-4_DREB)

ATGGCTTCCAAAGATGAGCTTCACGAAAATGAGGTTGTCAAAAACCAATCAAAAGATGCTAGAGGAAATAAAACCAATCTAATGGGAGATTGTTCTGTAGTCTTGCAACGTGTTGTACGGATTTACATAACGGACAATGACGCCACAGACTCATCTAGTGATGAGGAGGAAAATCATCAAGGAGAGAACTCCAAACGACAAAAAAGGATTTGCAAAGAAATTATCATAAAAAATGGAAAGACCAATGTCACTTCAAAAATGGTATCCTCCAAAGAGAAAAATGTTACAAAAACGCACCAAGAAAATGTAAAGAAGTACAGGGGAGTTAGACAAAGAAAATGGGGAAGCTGGGTTGCAGAGATTCGAGATATAAGAATTAATAAACGACGTTGGTTGGGAAGTTTTGCCACTGCTTATGAAGCTGCTTTGGCTTATGACAAGGCAGCCATTGAAATAAAAGGTCCAAATGCTTTGACAAACATTCTTAAGCCGCCACCGAAGGAAATCGACCCCATCAACCATTAA

>Solyc01g057080.1.1 (SlERF1-5_DREB)

ATGAGTTCAGGTGCAAAATACAGAGGTATTCGGAAGAGAAAATGGGGGAAATGGGTGTCGGAAATTCGAGTTCCAGGTAGTAGTGAACGACTTTGGCTAGGTACTTACACTAGCCCTGAAGCTGCAGCTGTAGCACATGACATAGCCTATTATTGTCTCCGTCCAGAATCATCATCGTCTTTACATAAATTAAACTTCCCTTCCATGTTACCGCCTAATGTTCAACCTGGAATGTCACCAACATCTGTACAAAAAGTTGCCTCTGATGCTGCCATGGCTATTGATGCTCAGTTTCTTACAACACCAACACCTGCTCAACAACATGCTGATAATTATGGGTAA

>Solyc01g065980.4.1 (SlERF1-6)

ATGTGTGGAGGTGCCATAATCTCCGATTATGATCCCGCCGGAAGCTTCTACCGGAAACTTTCTGCTCGTGACCTCTGGGCTGAGCTGGACCCTATCTCCGACTACTGGTCCTCTTCTTCCTCATCCTCAACCGTCGGAAAACCTGATTCCGCTCTGTCGCCGGTGACTCACTCCGTCGATAAGCCTAATAAATCAGATTCCGGCAAAAAAGGTAATAAGACTGTGAAGGTTGAGAAGGAGAAGAGTAGTGGACCAAGGCCAAGGAAGAACAAGTACAGAGGAATAAGACAGAGGCCATGGGGAAAATGGGCTGCTGAGATTCGCGATCCACAGAAGGGTGTACGCGTTTGGCTTGGTACATTCAACACAGCAGAAGATGCTGCTAGAGCCTATGATGAGGCTGCTAAGCGCATTCGTGGTGATAAGGCTAAACTCAACTTTCCAGCCCCATCACCACCAGCTAAGCGACAGTGCACTAGCACTGTCGCTGCTGCTGATACACCACCAGCACTACTCCTTGAGAGTTCTGACAACTCTCCTTTGATGAACTTTGGATATGATGTCCAGTATCAGAGCCAAACTCCCTACTACCCCATGGAAATGCCCATAGTTAGTGAAGATTATGAACTGAAGGAACAGATTTCCAATTTGGAATCGTTCCTGGAATTGGAGCCATCTGATCAATTTTCAGGGATCGTCGATTCTGATCCTCTTAATGTTTTTCTGATGGAGGACTTTGCTTCAACTCATCATCAGTTCTACTGA

>Solyc01g067540.2.1 (SlERF1-7)

ATGTCTTCTTCTAATTCTACACCAATCTCATCAGATAATCAATTACCATTTGGCATGTTTGAACCCATTCGAACCCCCACAGGCTATTCTTGGTTACAACGTAACACAGCCCTGTGTCAGCCGAGCGAGAAACGAGGACGTCGTAAACAAACGGAACCCGGACGGTTCCTTGGGGTTAGACGTAGGCCGTGGGGTCGCTACGCGGCTGAAATCCGCGACCCCACGACTAAAGAGAGGCATTGGCTCGGTACGTTTGATACGGCTCAAGAAGCGGCTTTGGCTTACGATAGAGCCGCACTTTCTATGAAAGGCACTCAAGCTAGAACCAATTTTATTTACACTTCCGATAGTGCTAACACTAATTTCCCTTCGCTTATTTCACCTTTTGAACATGTTCAAAACATGTTGAACCCTAACAACACGCATTTCAATTTCAACACTCATATTAACAAAAACACCAAGACTAAAACCACCACCACCACCAATTCTGATGAGTCATCGTCGTATGGCTCATCACCTAACGAGAACAATAGCTTCGTTTTTTTAAATGATGACATGAATAATAATAATAATAATAATACAAATTCAGGTTATTTATTAGATTGCATTGTTCCGGATAGTTGCTTGAAACCACCACCGCAACAACCACAAGAACCTTCTTCTACTCCTCAGAGTAAAATGAATGGGGAAATAGAGAACACTTATTATGATACGAATGGATTCTTCGACATGTCGTCGTCATGGGAGAGTTCTTGTGAGTTATCAGCCATGACGATGAATCATAGTAGCAACAATATGAATCATCAAATGATGGATGACGTGGCAGATGGAAGTTATTATTATCCAAATATGGAGATTAATAACTATGATCACCTAATGATGATGCCTAATCATGAAACGACTAATGCTGCTGCTGCTGCTGCTGCTTTTGGTGATGTTGAATTTGGATACACTACTCTATTTTGA

>Solyc01g090300.2.1 (SlERF1-8)

ATGTACCAACAAAAATCAACAACCATCTCAGATTCTGATCTTTCCGTACTTGAAAACATTAAATTCCATCTTGTTAATGATTCCGATTTTTCTCAAATCCTTTCGATGTTCGATCCGATCAATATTAGTCATGCAGATATTATTAATAGTCCGAATTCGAGTTACGGTAGCTCTACATCAGCAGCAGAGATTAGTTGGGGAGATATGATAACTAATATTGATAGTCCGTGGCAATGTATCGATAAATTGGAGCACGAAGAGGCACCAAAAGAGGAGCCTTTAGTGGCACGTGGTGTGCACGCGCCTGGTGATTGGAACCGGTATAGAGGCGTCAGGCGGCGGCCGTGGGGTAAGTTCGCGGCGGAGATAAGAGACCCCGATAGGAAAGGTGCTAGGCTTTGGCTAGGGACTTACGGGACACCTGAAGATGCAGCATTGGCTTATGACCAAGCCGCTTATAAGATTCGTGGCTCTAAGGCTCGATTGAATTTTCCTCATTTAATCGGCTCGGACATATCTGAGCCAGTTAGAGTGGCTCCGCGTAAGCGTTGCCACTCGTCACAGTCATCATTGGTAGAATATACCTCTTCAAAGAAGAGAAAATTATAA

>Solyc01g090310.3.1 (SlERF1-9)

ATGAATCCATCTGATTTTTCTCTTCTTCAATCTATACAACATCATCTTCTTAATGATTCTGATTTTCCAAATATATTTTCAGCTATTGATTCCAATAATACCCCTACTGATTTTACACAAAATAATTTCGATTACGGGGAATTAACACCATTAATAAACTCTAGTACTACACTACAAGCCAACGAAAAATCCGAGGTTGAAGAGTCCGAGACAGTGGTGGCAAGTGTGAAACACGCACCAAAAGATTGGAAGCGGTACCGAGGAGTAAGAAGGAGGCCGTGGGGTAAATTCGCGGCGGAGATAAGGGATCCGGATAAGAAAAATGCGAGATTATGGTTAGGGACTTATGAGACACCGGAGGATGCAGCATTGGCTTATGACCAAGCCGCATTCAAGATTCGTGGCTCGAAAGCTAGGCTAAATTTTCCTCATTTAATCGGCTCGGGTGTGCCCGAGCCGGCTAGGGTGAACCCTAGGCGTCGATCGCATTCGCCGGAATCGTCATGTGAAAATGGAACACCAAGAAAACTATATTTTATTTAA

>Solyc01g090320.4.1 (SlERF1-10)

ATGAATATCAATATGAAGTTCTCTTTATCTGATTTTGATTTTCTTGAATCAGTAAAGCAACATCTTCTAAATGATTTCGATTTTTTTAAATATTTTTCACCTATGAATTTAAACAACGTCGAATTACCTAATAGTGCTATCTCAAGTTTAGGTAGCTCCCTTTCAATCGAATCACATGAAAAGTTTGAATACGAGGAGGAAATTATAAAGGGACCTAATATGGTAGTAGCGCGTCAGAAAAACACGCCAGAAGATTGGAGGCGGTACATAGGTGTCAGACGGAGGCAATGGGGTACGTTCACGGCGGAGATAAGAGATCCGAATAAGAAAGGTGCGAGGCTGTGGTTAGGAACATATGAAACCCCGGAGGATGCAGCATTGGCTTATGACCAAGCTGCTTTTAAAATCCGGGGCTCAAGGGCTCGGGTCAATTTTCCTCATTTAATCGGCTCAAACATGCCCAAGCCGGCTAGACTAAAAGTGAGGCATCATACGAGCTCACTCAAGCCATCGAGTTTTTCATCCACGTCATTGAAAAACGGGATAAGGAAAAAAAAAATAGATCTAATAAATTCTATAGCGAAAACGAAAGCCAAAGTCAAATTAAATTTTTTTTGTCAAACACTAGAAAAGTACCTTCCCCAATAA

>Solyc01g090340.3.1 (SlERF1-11)

ATGAGTATTGTAATTGATGATGATGAAATCTTCTCTTTACCTAGCCTTGATGAACTTGAATCCATCACACATCTTCTTTATGACGACGATTCCGATTTTTTCGAAACTCTTTCCCCAATGAGTTTAGATGTTACAACATTATTGCCTAATATTCCTACCTCCAATTCAATTGAATCCCCCGTAACACCGGAGGAAACAAAAGAACCATCTGTGGCGTGTGAGGACGCGCCACAAGATTGGAGGCGGTTCATAGGGGTGAGGCGGAGGCAGTGGGGCACGTTTTCAGCCGAAATAAGAGATCCAAATAGGAGAGGAGCGAGGCTGTGGCTCGGAACTTATGAGTCCCCGAGGGATGCAGCATTAGCTTATGACCAAGCCGCTTACAAGATTCGGGGAACCAAAGTTCGGCTTAATTTTCCTGACCTGATTGGCTCGGACGTACCTATGCCACCTAGAGTAACGGCTAGGCGTCGTACACGCTCACGCTCACGCTCACCCGAGCCATTAACAACTTCGTCCTCGTCATCCTCATCATCCTCGTCCTCGTCCTCGTCCTCGTCGGAAAATGGAACGAAGAAAAGGAAAATAGATTTGATAAACTCAATAGCAAAATCCAAATTACTTTGTGGGATGGATTTACAAATGTTAATACAAATGTGA

>Solyc01g090370.3.1 (SlERF1-12)

ATGCAAGGAAAAATATCCTTAGATACTGAACAAAATTTTCTTGATACAATGCAACATCTCTTCAACGATTCTGATTTTTCCCAAATATTATCAGAAATTCAAACTCCTAGACAATACTCAACTACTCCTGATCATCAAAACGCGATCGCACAAGATAATATCCCGATATTATTACAAGAAATAGAAGAAAATAAGGAGTCAACAACACGTTTATCGGAGCATAGGCCGAAAAAATATAAAGGAGTGAGAAGAAGGCCATGGGGAAAATACGCTGCGGAAATAAGGGACCCCGAGAGGAAAGGGTGCAGGCTATGGCTAGGGACATATGAGACACCTGAGGATGCAGCATTAGCTTATGATAGAACCGCGTTTAGGTTACGCGGTTCTAAAGCTGTGTTGAATTTTCCTCACTTGATTGAAACTAATGTGACGGAGATTAATAGAGTGAGGCCGAGGAGACGTCCTCGTTCCCCCGAGTTTTCATCGTCGTCTCCTCCTCCTCCTCCTCCTCCTCCGTACGTAGACGAGAGTAATAATAATAATACTGATGGTTCGATTTCAAAGAGAAGAAATGTTGAGCTAATTAATAGCTTAGCTACGGTGAACAATTTAGATTGTCAAAATATTATGGAGAAATATTTGACGTCTGATTATTATTTTGCTTGA

>Solyc01g090560.4.1 (SlERF1-13_DREB)

ATGGAAGATCATAATGAATTCTCCATTTCTAGTTCTACTAATTCTACAACTTCATCCTTTACAAGTTGTTGTTCTAATAACAATTGTTTGTGTTCTACTTCAACCACAACATGTTCAACTTCTTCTACTTCATCTAATAATTGTTTACATGAGAGCATTGCAAAAACCTCATCCAATGATCATTACAAGTGCAAATCCAAGAAAAGCGCGAAAAAAAATCAAGATGGGAATGAAGAGGAGGATAACACGAAAATCAGAAGAAAAAAACAATGTGTTGTTGATGAAAAACATCCAACTTATAGAGGAGTTCGTAAGAGGAATTGGGGAAAATGGGTGTCTGAAATTAGACAACCAAGGAAAAAATCAAGAATTTGGTTAGGGACTTATCCCACCGCTGAAATGGCAGCTCGAGCTCATGATGTTGCGGCTTTAGCCATCAAAGGTGATTCGGCTTACCTTAACTTCCCTCATTTAGCCGATCAACTCCCTCGACCCGCTTCCGCTTCCCCTAAAGACATCCAAGCCGCCGCTGCCAAAGCTGCAGCCGCCAGCATATTTCTTGAGGAGAATTCGGTTGAGTCGAGCCAGTTAACCGGGCTGCACAGCTCGCATTCCTCGACTAATTTAGCCTCAGATAACGTACAAGAATTACTAAATTCTCCCTCGATGGATCACGATGACCCGTTTTTCGATCTACCGGATCTTATCATCGATAGACTCGACCAAAATGACCGGTACCAGTACCCGGTTTCGACGTGGCAGCTGGCTGGAGCCGATAGCGGGATGTTTCGGCTTGATGAGCCGTTTTTGTGGGAGTGCTATTAG

>Solyc01g091760.3.1 (SlERF1-14_DREB)

ATGGCTACAGATTTAGAAAACACGAAAACCACCATCACAACCAACACCAACACCACGAATTCATCATCAACAGAAGCAAAGAAATCATCAATAACAAGAAAATTTGTAGGTGTAAGACAAAGACCCTCTGGAAGATGGGTTGCTGAGATAAAAGACTCGTCTCAACGTGTGAGATTATGGCTAGGAACATACGATACTCCTGAAGAAGCTGCTCATGCCTATGATGAAGCTGCTCGAGCCCTAAGAGGCGAAAATGCACGGACCAATTTCGCTTCTACTACTCCAAATTCCGATATGGATCAATCGAATATATTGCATAGTAAAAATGGTCTTAGCTTTTCATCGATAAAAGCAAAATTAAGCAAGAATTTACAAAGCATCATGGCTAGAAATTCAGAAAATAAGTCATCATCGTCCAAGAGTATTACTAGAGTAAGTGATCATTTCACATTTGCTAGGATTTTCCACTTCAAGAATAATTATGATCAACCATACCAAAATCATCGTCATGTGGACATGAATAAAGTTGTGCAACCAAGTATTAGAGTACTTCCACATGACGTAACCGATAACAATAACAATAATGATTCGTCTTGGGAAAATTCATCAAGTGTGTCTGATTGTAGTAGTGAATGGGCTGCTTTTAGGCAACTTGGGCTGGATTGTGATAATTATGGATCAGATGGAAGTGAGTATTTTGTTGGATCAGATCCTTTAATGGCTGGGTGGATGAGCAGCCCAGATATTATGAGTAGCACTAGTACTAATGAAGGATCATCAAGAAGCAAAAGGTTTAAGGTTTCTTCTTCTGTTGTGGTTCCTCCTACTTTTACTGAATCTCCATTACATGATGCTCAAAATTATGTACCATTTTAA

>Solyc01g095500.3.1 (SlERF1-15)

ATGGAATCACAAAAAATCAAAAAGAAATTAGTCCACAAAACTATCACTACTAAGTATGATCATCACAACAAGTGGACTCCTAAAGTTGTTCGGATTTGTTACACTGATTGTGATGCTACTGATTCTTCAAGCGACGACGATGACGACGAGAGGAATCGAGTGAAAAAATACGTTACAGAGATTAAATTTGAGAAGAAAATGGCTGCTGCAGATGTGAGGAAATCGTTGAATTCGAATAAGAAGAAGAAGAAAGCGATCGATTTGAAGAGAGATGAGAATGTTAAAAAGTTTCGCGGTGTCAGACAGAGGCCATGGGGAAAATGGTCTGCGGAGATTCGAGATCCGGTGAAAAAAACGAGGGTTTGGTTAGGTACTTTTGATACCGCTGAAGAAGCGGCTATGAAATATAATATAGCCGCTATTCAATTGCGCGGAGCTGATGCTATCATTAATTTTATTGAGACACCTTTCCCAAAGGAAAATGCGATCACTTCAGTATCGGATTATGATTCCACAGGGGAATGTGAAAACCTCTGTTCTCCCACCTCAGTTTTGAGGCAGAATAATAACAATAATGATAAAGATAACGAAGATGCGATTGCGATTGATACTAAAATTATGAACGATGAGAGCAAAAAAATGGAAATGGATGAAAATGGATTTATGTTTGATGATAATTTGCCATTAATGGATCAGAGTTTCCTTAAGGATTTCTTCGATTTTCGATCCCCTTCTCCATTGATGGATGATGTATTATTACCAGGTTTTAGCGATGGAATGGGATTATTACCAGAAGTGTTGAGTATTCATGGAAATAGAATGTTGGATGAAGATTTGGAGACTTGTAAGTGGGCAAATGATTTCTTCCAAGATGTTTGTTGA

>Solyc01g108240.3.1 (SlERF1-16)

ATGCATTGGTTAAATAAAAGATTTAGACAAGAAGCAGGAATGAATTCGAATTCGAATTCCCTCCAAAATAACAATCAATTTCAACAGCAGCAACCAAGGCTTACTGGAGATGAAGAGTACTCTGTTATGGTTGCAACTCTGAAAAATGTGATCAATGGTAATATTCCAACGCAAAATTATCAAGAATTCAATGTCTTTTCGCCATATAATTATTCTACTGCCACCACAACCACGAATGTTACTTCTTCTTCTTCGCCTTCTACTAGTATGTCTACTAGTTTCGAACAAGTATTGGGTGTCTCTGCTGAACAAGAACCTTGTCAATTTTGCAGAATTCAAGGTTGTTTAGGCTGTGACATTTTTGGTACCACATTTTCTTCTTCTTCTTCAGCGCCTGCTGCTGTGGCTGCTCCTGTTGCTGATAATAAGAAGAAGAGTAGTAGTAGTAGTACAGCTACAGTCGCAATCGCGAAGAAAAAGAAGAAGAATTACAGAGGAGTGAGACAGAGGCCATGGGGGAAATGGGCAGCAGAAATTCGTGATCCTCGAAAAGCTGCACGTGTTTGGCTGGGAACTTTCACTACGGCAGAAGAAGCAGCTAGAGCTTATGATAAAGCCGCCATTGAATTCAGGGGTCCACGAGCTAAATTGAATTTTTCATTTGCGGATTATACCGTTGACACTCAAGAACAACAGAGCACTTTATCTTCTTCACCACAACAATTACCAGAAGAGCCTCAGCAATCCCAGACAGCGAATAATAATTCCGATTATGGAAATGAAATTTGGGATCAATTGATGGGTGACAATGAAATTCAAGATTGGTTGACCATGATGAATTTCAATGGCGACTCTTCTGATTCTGGCGGGAATGTTCACAGCTTTTAA

>Solyc02g030210.4.1 (SlERF2-1)

ATGGCTTCCCTTTCCTCTGAGCCTACTGCCAAGACTGAGGGTAGCAGTGGCAATGATGCTGGTGGTGGAGAAACATCAGAAGCTATGGGGCATATAGGGACTGATCAACTGTTATTATATAGAGGACTGAAAAAAGCGAAAAAAGAAAGGGGTTATACAGCCAAGGAGCGCATAAGCAAAATGCCCCCATGTACTGCAGGGAAGCGCAGCTCCATCTATCGTGGTGTCACTAGGCATAGATGGACTGGTCGTTATGAAGCTCATCTTTGGGATAAAAGTACCTGGAATCAAAACCAAAATAAGAAAGGAAAGCAAGTTTATTTAGGAGCATATGATGATGAGGAGGCTGCAGCAAGAGCATATGATCTTGCCGCTTTGAAATATTGGGGTCCTGGAACCCTCATTAATTTCCCAGTGACAGATTACACAAGGGACCTTGAAGAGATGCAAAATGTCTCAAGAGAAGATTATCTTGCATCATTAAGAAGAAAGAGTGGTGGTTTCTCCAGAGGGATCTCTAAATATCGTCCGCTATCCAGTCGATGGGATCTGCAATTCGGACGTGTGCCTGGAGCTGATTACTTTAACAGCCTGCACTATGGTGATAACGCCACAGTGGACAATGAGTACATCGGTGGTTTCTGCATGGATAGAAAGATTGACTTGTCAAGTTACATCAAGTGGTGGGGAGGTAATAAAGCTCGTCAAACAGATTCGCATCTGAAAATATCGGAGGAAACAAAGGTTGGTTGTCCTGAAGATATTGATAATGAGCTTAGGGCTTCAGAATTATCAATTCAGCAGACTGAACCATATGAGATGCCCCGTTTGGGTGTGTACCAAGAAAATAAGAATCATAAAAGCTCAACATTATCTGCTGTGAGCATTTTGTCACAGTCTGCAGCATACAAGAGTTTAGTAGAGAAGGTTGCTAAAAAGAAAGAAAAAGTTGAGAATGATGAGAATGAAAACAAAAGCACTATCAACAGAGTCGACCGTGGGAAGATGATAGAAAAATCAAGTCCTGATAGTGGAAGTGAGAGGCTTGGAGCTGCATTTCTCAATGCTGGGGGATTATCTATTAACAGAAACTTGCACCCACTTACTCCACTCCTGTCAGGACCACTTCTCACTAACTACAACAGCATAGATCCCTTAACAGACCCTGTTCTTTGGACATCTATTGTTCCTAATTTTCATACAGGTTCTTCACGGACTGCTGAGGTCCACAAGAGCGAAGCCAGTTCAGATTATACTTTATTTCAACAAGAAGATTAA

>Solyc02g067020.1.1 (SlERF2-2_DREB)

ATGGGTAGGAAGAGAAAAGCTGAAGGACTCATGAAAATTCAACGACCGCGGAAGAAATTTGTTGGAGTTAGACAGAGGCCATCAGGCAGATGGGTTGCTGAAATAAAAGACACCATACAGAAAATAAGAGTATGGCTTGGGACTTTTGACACTGCTGAAGAAGCAGCAAGGGCTTATGATGAAGCGGCTTGTTTGCTTCGTGGACCAAATACTCGCACGAATTTCTGGACTTCTTCTTCTCCATCTTCCAATTCAGCTCTGCCTCAGAAAATCACTAAACTTCTTCTCAGCAGACTCAGAGAACAAAACAAATCTGCTGCTGCTGCTGCTGCTGATTCATCCAGTTCTACTACCTCTCTGGCTGAAATTGATCATCAGCAGCAGAAGCAACAAGAAAAAATCGGAAACAGAGTAGTTGATTTTTCAGATTCGTTGTATACTGATTATCTCAATTACCCTGAAGATAATGTAACTGAAAACAACGTGATTGCTCCAATTACAAGAGAATTAACAAGCATAGTTCAAAATCAGGAAATTAATTTTCAGCCTGTGAATAACTATGAGATTATAGAGATAGGAGAGGCAATTAATATTGATGTTGAAGATATAGAGTCAGATATTGACTTCCAATTTCGTTTTTCGCCATTTGATTTAGCTGAAGAGCTATCAATGGATTTTGGGGAAGAAACATCAATTGTAAGTGAGGCAATGAGGAGGATGAATTACGAGAGGAAGTTTTCAGCTTCACTCTATGCTTTCAATGGGATCACGGAATGTTTAAAGTTGAAGATGAAATCTGGAGGCGTAACGCGATCTGATCAATTATCCAGGATTCAAAATGCATGCAAAAGGAACCTAGTCAAGGAGAGGGAAAACGAAGAACGCAACGCTGGCAATGACTGA

>Solyc02g077360.1.1 (SlERF2-3)

ATGACTACTCATCATGTGGAAAATAATAATCAAGAACAAGATCAAGTAGCATGTGAAGAGATTCTAGAGAATGTTTGGGCAAACTTCATATCCAAGAATGATCAAAATTCTCAAAAGGTGACAAATGAATATTGTTGTGAACAATATTGGGAACAACTTCCAATTCTTGAAAGATTACCAAGCTTAGGAAGATGGATATCAATGGGAGCTGAAACTTGGGAAGATATTCTCAATGGAATTATTATTCCTTCTCATAACAACGAAAATTCAAACGACGAATCAACATGCAAAGATGTTGTCAACGTTGAGAAGAAGGAGGAGAAGAAGAAGATGGTGCATTATAGAGGGGTAAGAAGGAGGCCATGGGGAAAATACGCGGCTGAGATAAGGGATTCATCAAGAAAAGGAGCTAGGGTTTGGCTAGGGACATTTAGTACTGCTGAAGAAGCTGCTATGGCTTATGACAAGGCGGCTTTGAGAATTAGAGGTCCCAAGGCCTATCTTAATTTTCCACATGAAATGGTTGCTCAAGCTATAGGAATATCTAATGGTCCTTGTGAAAAAGAATGGACCTTTTCATCATCAAGTCAATATAATTCAAGGAAAAGGGTATCAAGGGATTGGAACATGTATGAAAATTTGGATGAAATTAATCAATTGCCTATGGAGAAGAAAATTATGAGAAGCATGGAGGAGGATCTTTTTAATGACTTGGATATCCTAGAGTTTGAGGATCTTGGAAGTGATTATTTGGATAGTTTATTATCCTCTTTGTAA

>Solyc02g077370.1.1 (SlERF2-4)

ATGGTTCCAACTCCTCAAAGTGATTTACCTCTTAATGAGAATGACTCACAAGAGATGGTATTATATGAAGTTCTTAATGAAGCTAATGCTCTAAATATTCCTTATTTACCCCAACGAAATCAATTACTCCCTAGAAATAATATTCTTCGTCCATTACAGTGCATAGGCAAGAAATACAGAGGAGTACGACGTCGTCCGTGGGGGAAATACGCTGCGGAAATTCGCGATTCGGCTAGACATGGTGCGAGAGTATGGCTAGGTACGTTCGAAACTGCTGAAGAAGCTGCGTTAGCTTATGATAGAGCGGCTTTTAGAATGCGAGGTGCTAAGGCACTACTTAATTTTCCATCTGAAATAGTGAACGCCTCTGTTTCAGTAGACAAATTAAGTTTGTGCTCAAATAGTTACACTACGAATAATAATTCAGATTCAAGTTTAAATGAAGTTTCAAGTGGAACTAATGATGTATTTGAATCAAGATGTTAA

>Solyc02g077810.1.1 (SlERF2-5_DREB)

ATGGCACAAAACAGCAATATCAACGTAAGAGTTGGGTCAAGTGAAATGCGTTATAAAGGTGTGAGGAAGCTTTCGACAGGGAACTACATCGCTGAGATCAAAGTTACGAGCCAGAGAAGATTTGTGTGGCTTGGAACGTTTGATACAGCGGAGGAAGCTGCTAGGGCTTATGATGCAGCTGCCCGACAGTATCTCGGCCCACGCACAGTTATAAATTTTCCTCCATTAACTAATCAAAAGGACGTCAAGAGAAGTAGAGATTATCTTCAAAAGCTTAATCGTAAGGATGTCATGATCAACACTTGTGCATCGTCTTCTGGATCAGGCGAAACTCTGATGCCAAATCAGAGTTCTGATCAGGAGGAGTTTCGTCGTTTTACAACAGTTACTGGAGTTTCTGTTATGATGTCAATGGTGCGAGACAGAAGCTTAATAGAGGCTTTAACGAGAGAAGGATTGATACTTCCGGACCCGGAGATCATCGATACCCAGCCCCTCAATCTTGAGCTCACTCTTGCACCGCCAGGGACCATGGCAAACAAATAA

>Solyc02g077840.2.1 (SlERF2-6)

ATGGCTTCCACTCGTGAGGGTCACTACAGAGGAGTGAGAAAGCGTCCCTGGGGCCGTTACGCCGCAGAGATTCGCGACCCGTGGAAGAAAACACGTGTCTGGTTAGGCACTTTCGACACTCCAGAAGAAGCGGCACTCGCTTACGACGGCGCCGCCCGTTCACTCCGCGGCGCTAAAGCAAAAACAAACTTCCCCCTCCTCCGCCGCCGCCGCCGCAGCCTTCTTCCGCTCCTCTTACACTCGATCTCAACCTTCCATCTGATCACCGGTGGACTTCCCCCTCCGGACGTAGGCTCATGA

>Solyc02g090770.1.1 (SlERF2-7)

ATGGATAATTTATCTAGCTTTACTCACAAACTGACTCATCAACAAGAATCTCACTACATGGTGTCAGCTCTCAAACATGTTTTCTCTGGAGCCGCCGTTGATGGTCGAGCCGACGGAGAAGCTGCTCAGCTTTTATGGGAAGTTGAAAATGCAGCCACGTCAGATAATTCAATGCCACGTAGGAGTATCAATGTTCACCCAGTACACGTGGAGTTGGAGTTGGGGAAGAAGAAGCAGAGGAGGAGGAGGAATACGAAGAAGGAATTTAGGGGAGTGAGACAAAGACCATGGGGAAAATGGGCCGCCGAGATCCGTGACCCGCATAAAGCCCAACGACTTTGGCTTGGTACGTTTGTTACTGCTGAAGATGCTGCTAGAGCTTATGACAAAAAAGCCGTAGAGTTTAGAGGGAATAAGGCAAAAACAAACTTTCCTCTTAAGGAATATATTGATGATCACGACAACAGTTCCCCAATGATGAAAGTGGAAGAACATGAAAATGACCAAAATGTGATGAATGGTGATGAGAATAATAATAATGGTGATGACTTTTGGGCCACATTGGAAGATGATAGACTTGTCAAATTTATCACGCAGGATATGTCCCACTGA

>Solyc02g090790.1.1 (SlERF2-8)

ATGAATAGATTCCGTCAAGATTCTCTACTTGAAGAAAATAACAGTTATCCCCTCGCACACAAGCTGACTGAAAACCAAGAACTTTACTACATGGTGTCAGCTCTACGACATGTTGTCTCCGGCGCCGGCGCCGGCGACGTCCGAGCGAACGGAGAAGCTGCTCAGCTTTTGTTGGAAGTACAAAATGCTAGCGGTGGTAGTTCATCCACGTCAGCTAATTCGATGCCACGTAGGAGCAGCATTAATGTGGAGTTGGGGAAGGGGAAGAAGAGGAGAAGGAGGAATATGAAAAAGGAATTTAGGGGAGTGAGACAAAGACCATGGGGAAAATGGGCCGCGGAGATCCGTGACCCGCATAAAGCCCAACGACTTTGGCTTGGTACTTTTGCTACTGCTGAAGATGCTGCTAGAGCTTACGACAAAAAAGCTATAGAATTTAGAGGTGAAAAGGCTAAAACCAATTTTCCTATTTCAGAATATACTGCTAGGGCGGTCAATGATGAGATTAACAAGTTGGCTAGTGTAGTAATGGAAGAAGAGAGAGAATTATTAGTACAAGTCCTTAATGGTGGTGACGTGGCTGTTAATGGTGAAAATGGTAGTAGTATACAGAATGGTGATGAAGACGACTTTTGGGAGACGTTAGAAGATGATGGACTTGTCAAATGGATATGTTCGGCTTAG

>Solyc02g090800.1.1 (SlERF2-9)

ATGACTATTTTATGCGAAGATTCTCTACTTGAACAAGAGAATGTGAGCTATACCCTCACACACAAACTGACTCAAGAACAAGAAAATTACTACATGGTGTCAGCTCTCCGACATGTTGTCTCCGGTACCGGCGATAACGATGAAGCTTCTCAGCTTTTGTTGGAAGTTGAAAATGCTAGTAGTGGTAATTTGGCATGTAGGAGTATTGATGTGGAGTTAGAGAAGGGGAAGAAGAGGAGAAGGAGGAATACGAAAAAGGAATTTAGGGGAGTGAGACAAAGACCATGGGGAAAATGGGCTGCGGAGATACGTGACCCGCATAAAGCCCAACGACTTTGGCTTGGTACGTTTGCTACTGCTGAAGATGCTGCTAGAGCTTACGACAAAAAACTATAG

>Solyc02g093130.3.1 (SlERF2-10_DREB)

ATGAGGAAGTGGGGAAAATGGGTTGCAGAAATTAGAGAACCCAACAAGCGTTCGAGGATTTGGTTGGGTTCGTATTCAACGCCGGTGGCTGCGGCTCGGGCTTATGATACGGCGGTCTATTATCTAAGAGGCCCTTCGGCCCGATTGAATTTTCCCGAGTTGTTAGTTGGTGATGGTGGGCTTAATGATTTGTCTGCTGCTTCGATTCGTAAAAAAGCCATAGAAGTTGGTGCTCAAGTAGATGCTGTACAGAACTCGCTTGCAACCCATCATAACCATACAGAGGAAAAGGTGCACTCTGAGACGGCAAGCCCCTCAGAATTGAAGCCTTGTTGGTTTCAAGAGAAGCCCGATTTAAACTTGAAGCCCGAGCCCGAAGATCCAGAAGTGGATTACTGGTAA

>Solyc03g005500.1.1 (SlERF3-1)

ATGGACGAAGTGAAATATCGAGGTGTTCGAAAGAGGCCATGGGGAAAATATGCAGCAGAAATTCGCGATACTAATAGACAAGGAGGGGTTCGTGTTTGGCTAGGGACATTTAGCTCTGCTGAAGAAGCTGCTCGAGCTTATGACAAGGCAGCTTATAACATGAGGGGACATTTGGCCATACTTAATTTTCCAGAGGAGTATAACTTGCCTCGAAGCTCTTCTCATTTTTACAACAATTCTTCATCAATGCCATCGTCGTCGTCTTCAAGTAACGTTCATGCAAGAAACGATCAACAAGGAAACCGACAAGTTCTTGAACTTGAGTATCTTGACGATCATGTTCTTGAAGAACTTCTTGATTGTGATCAACACGCAAATAACAGAAAATGA

>Solyc03g005510.2.1 (SlERF3-2)

ATGTCAATGTGGTGTGTGCCACCAAAAAAAAATCTCTCACATATAATAATCAACATGGATAGTAGTTCTTCTTCGTCTTCTTCTAAAGGAAAAAAAGTGATACAATTAGAGAAAGAAAATAGCAAGGATAGTAATGATTTGATCAAATATCGAGGCGTTCGTAAACGTACGTGGGGTAAATTTGCAGCGGAGATACGTGACCCAACGAGACAAGGGGCTCGTCAATGGCTAGGGACGTTTGATACGGCCGAGGACGCGGCTAGGGCCTATGATAAGGCAGCATTTAACCTAAGGGGACATCTTGCAACTTTGAATTTTCCAAATGAGTATTACTCTAAGCTTAATGATCCACATTATTATAATTATCGTACATCATTAAATGTTAATATTCCAAGTCGAAGTTTAGAGAGGGGAATTTCATCAATTGGTCATCAAGACAAAGAGATTATTGAGTTTGAGTATCTTGATGATAGTGTTTTAGAGGAACTTCTTGGAGCAGAAGACCCAAAAAAGATCATAAGAAAGTGA

>Solyc03g005520.1.1 (SlERF3-3)

ATGAATTCTTCTTCTAATTTTCTATACAATCTCAATTTCAACACCTCTTCCCTTCCATTCAACATAAATGACTCGGACGAGATGTTGCTCTATGACCTTCTCGCACAAGCTGACTCAGACACTACCACCTTAATAACAAATAGTACAACCATGGCCAAGCCAACACCTTCATCAAAGGAAAAGAATTATAGAGGTGTTAGGCGTCGGCCATGGGGAAAATTTGCGGCGGAGATTAGAGATTCAACTAGAAATGGAATTAGGGTATGGCTAGGGACATTTGATAGTGCTGAAGATGCTGCTTTAGCATATGATCAAGCTGCTTTTTCAATGAGGGGTACAAGTGCAATCTTGAATTTTTCAGTTGAAAGAGTTGTTGAATCTCTACATGAAATGAAATTTCATGTTGAAGAAGGTTGTTCACCTATTGTGTCTCTCAAGATGAGACATTCTATGAGGAAGAGGAAATTCAACAAGAAAAATAAAGTAAGTAGGGAAGTTGTAAAAGATGATAGTAATTATAATGTTAATATTGTAGTGTTTGAAGATTTGGGTGTTGATTATTTGGAACAACTTTTGGGTTCAAGTGATCATAGTAATAGTAGTAATGATGATCGCGGATCGTGGTGGGATGAATAA

>Solyc03g006320.1.1 (SlERF3-4)

ATGGCGCCCAAGCCAAAATGTACGACGGCGGCGACGGCGGCGACGGCGGCGGTGGTGAAGGAGGTGCATTACAGAGGCGTCCGGAAGAGGCCTTGGGGAAGGTACGCGGCGGAGATAAGAGATCCAGGGAAAAAATGCCGTGTTTGGTTAGGTACGTTTGATACCGCTGAAGAAGCAGCGAGAGCGTACGATAAAGCTGCGATTGAGTTTCGAGGTGCGAAAGCGAAGACTAATTTTCAGATGCAGCAGCAGCCGGACGATGTGATTCGGAGTCCGAGTGATACGAGTACGGTTGAATCGTCGTCTGCGGCGGTTGCGGTTGCGAAGGCGCCGGCAATGGTGGAATCGATTCCGTTAGATCTAAGCTTAGGAAGCTCATCATCGGCTGTTGGAATTTTTAGCAGCGGTGTAGGTAAGTTTTTGTTTCAGAATTCTCCGCCGATGTACTATTTTCAAGGAGCTGGAGTGATTCGTAACGGCGGCGGCGGCGGTGGTGGTAGTGGAGATGGAGGTGGTGCTGGTGCTAGTGGTGCCGGCGGGATGATGAAGAGTGAATCCGATTCATCAACTGTGATAGATTTTATGGGTAACAATTTCAAGCCAAAAGCTAAGTTCGACCTTAATCTTCTTCCGACACCGGAAGACATGTGA

>Solyc03g007460.3.1 (SlERF3-5)

ATGGATTATCATTCGCTGTGTCCGATCAAATACACTGAGCATCGGAATGTTATTAGAAAAGTTACTAAACCATCTTTGGTGAAATCAAAAAAACTCTCAGAAGCTGCAAAATCCTCTCAGTTGAATCCCTCTGTTCCAAGGACTGTTCGGATTTCTGTGACAGACCCAGACGCAACCGATTCTTCAAGCGATGAAGAAGACCTGCTTTTTGGACGGCGACGGGTGAAAAAGTACATCAATGAAATCAGTATTGAAACTGCCGTCAAGTGTGAAGTTAGCAGTGGAAATGGCAAAACGGTGAATAAAAGAGCACCAGAGCCTCTTCAGACTAAGCAGAAGCCAATGAAAGTTCAGCCTCCTCCATCGGCTGGTGCTGCTAGGAAGTTCCGCGGTGTGCGTCAACGGCCGTGGGGTAAATGGGCGGCTGAGATCAGGGACCCAGCTCGCCGTGTTCGGCTTTGGTTGGGAACTTACGATACTGCTGAAGAAGCTGCTATGGTTTATGACAACGCTGCTATTAAGCTCCGTGGTCCCGATGCTTTAACAAACTTCAGCACTCCTGCCAAGGCCGAGCCAGAACCAGAACCAGAGCCAGAGCCCGAAATCAGTGCTCTGTCTCACTCCGGCTATGAATCCGGGAATGAGTCCCGTAACATCCCCTCTCCAACTTCTGTTTTACGGTGTACAATGAGCCAATCAGAAAGCGGGTCCGGCCAGGTTCATGTTTCGGAAGAGTGTCCTTCGGTTCAAGGATCGATGGAATGTGAGCAAACGGTTCAACCGTTTGTTCAATGTGCTGCTGAACCGCTGATTCCATCAGCGATTCCGCAGGACGTTGAGGAATGTCAGGGCGAGACCAGCATGATTCCTGATTACTCAAGCGATTACTTGCCAACAGATATTCCATTCTTAAATGATTTTTTCAATTTCGATGGTTCAGCAGCTGAGCAAACGTTACTTGAGGACTCAACTATGACAGCCGTAACATCAACAACAACAACAACAACACCAAACGAATTTGGCGATTTGTGCAACGATTCATTGGATTTTGGGAATGATTTCTTGTTTAACGATGCTGATTTCGCTGAATTCGGCAGTTTTGATGATCTGGGTGATTTAGGAATGGATGATTTTTCTCAAGACAACAGTGTTGTTGATTATTCCAGTGTTGATTCTCTGTTAGCAATTTGA

>Solyc03g026270.3.1 (SlERF3-6_DREB)

ATGTTTTATTCGGACCCACGTATAGAATCTTGTTCATCGTTTTCTGACAGTATTAGAGCCAATCATTCTGACGAGGAAGTTATTTTAGCTTCAAATAATCCGAAGAAGCCAGCTGGCAGAAAGAAGTTTCGAGAAACTCGACATCCAGTGTACAGGGGAGTGAGGAAGAGGAATTCTGGAAAATGGGTTTGTGAAGTCAGAGAACCAAATAAGAAGACGAGGATTTGGCTTGGTACTTTTCCTACTGCTGAAATGGCGGCTAGAGCTCATGATGTGGCGGCTATAGCATTAAGAGGACGTTCAGCTTGTTTGAATTTTGCTGACTCTGCTTGGAGGCTGCCTACTCCAGATTCCTCTGACACTAAGGATATTCAAAAGGCGGCCGCTCAGGCCGCCGAAATCTTCCGACCTTTAAAGTCGGAGGAAGAAGAATCAGTGGTTAAAGATCAATCTACTACTCCAGATGATATGTTTTTTATGGATGAGGAAGCGTTATTCTGCATGCCGGGTTTACTTACGAATATGGCGGAAGGATTAATGGTACCTCCACCTCAATGTACTGAAATGGGAGATCATGTGGAAGCTGATGATATGCCTTTATGGAGCTATTCTATATAA

>Solyc03g026280.3.1 (SlERF3-7_DREB)

ATGAATATCTTTGAAACCTATTATTCAGACTCGTTAATTTTAACCGAATCATCTTCTTCTTCATCGTCATCGTCGTTTTCTGAAGAGGAAGTTATTTTAGCTTCGAATAACCCGAAAAAGCCAGCTGGCAGGAAGAAGTTTCGAGAAACACGGCATCCGATATACAGGGGAATCAGGAAGAGGAATTCAGGAAAATGGGTTTGTGAAGTCAGAGAACCAAATAAGAAGACAAGGATTTGGCTTGGTACTTTTCCTACGGCTGAAATGGCGGCTAGAGCTCATGACGTGGCGGCTTTAGCATTAAGAGGCCGTTCTGCTTGTTTGAATTTCTCTGATTCTGCTTGGAGGCTGCCTATCCCTGCTTCCTCCAACTCTAAAGATATTCAAAAGGCGGCCGCTCAGGCCGTCGAAATCTTCCGATCGGAAGAAGTTTCAGGAGAATCTCCTGAAACGTCAGAAAATGTGCAAGAGAGTAGTGACTTCGTGGATGAGGAGGCGATCTTTTTCATGCCAGGATTACTTGCAAATATGGCAGAAGGACTTATGCTACCTCCACCTCAATGTGCAGAAATGGGAGATCATTGTGTGGAAACTGATGCCTACATGATAACTTTATGGAATTATTCTATCTAA

>Solyc03g093530.2.1 (SlERF3-8)

ATGATAAGCAAAGAAGAAGAGAAATTAACTTCGGTCGGATCCAACCCGGATCGAAAATTCTCCGGGTCGGGTAATGGATCTTCGCCGGCCGATGAAGGAACGAAGAAGCAGTACAGAGGGGTTAGGAGGCGACCGTGGGGAAAATATGCAGCAGAGATAAGAGATCCGACCTGGAAAGGTTCACGGGTCTGGCTCGGAACTTACGAAACCGACGTGGATGCTGCCAGAGCTTACGATTGTCTGAGAGGAAGAAAAGCCATCCTTAAGTTTCCGTTCGATGCCGGAAATTTAATACCGCAGGTGACGATTGGCCGGAAGAGGAGAATTGATCAGTGA

>Solyc03g093540.1.1 (SlERF3-9)

ATGGGCTCTTCACAAGAGATTTATACTTCACTTGATTCCATTAGGGAACATCTTCTTGATGATGATGTTACTTTTATGGAATATTACTGCTCTAAATCTTGTTTCTCTTTTCAAACTTCAAATCTTGACCATACCTCAAAAACAGAGTATGATGGTTTTTTCGAATTTGAGGCAAAACCACATGTTATCAGTTCAAATTCCCCGAAACAGAGTAACTTGAGAGAACGGAAGCCATCTCTGAACATTGCGATACCCGCGAAGCCAGTTGTTGTTGTAGAGAACGTTGAGAGTGAGAAGAAGCATTACAGGGGAGTTAGACAGAGGCCATGGGGGAAGTTTGCAGCGGAGATTCGTGACCCAAATAGAAAGGGGACTCGAGTTTGGTTAGGAACATTCGATACTGCTGTGGATGCGGCAAAGGCATATGACAGGGCAGCGTTTAAGCTTAGAGGAAGCAAAGCAATATTGAATTTCCCACTCGAAGTTGCAAACTTTAAGCAACAAAATGATGAGACTAAAACAGAGATGATGTCATCAGGCAGTAAAAGGGGGAGAGGAGAAACAGAGGAATTAGTAATCAAAAAGGAAGATGAAAGAGTTGTTCCAATGGCATCACCATTAACACCGTCGAATTGGTCGACGATTTGGGAGAGTGGAGATGGAAAAGGCATTTTTGATTTACTTTGA

>Solyc03g093550.1.1 (SlERF3-10)

ATGGGCTCTCCACAAGAGATTTATACTTCACTTGATTCCATTAGGGAACATCTTCTTGATGATGATGTTGCTTTCATGGAATATTACTGCTCTAAATCTTGTTTCTCTTTTCAAACTTCAAATCTTGATCATACCTCAAAAACAGAGTACGATGGTTTTTTCAAATTTGAGGCAAAACCACATGTTATCAGTTCAAATTCTCCGAAACAGAGTAACTTGAGAGAACGGAAGCCATCTCTGAACATTGCGATACCCGCGAAGCCAATTGTTGTTGTAGAGAATGTTGAGAGTGAGAAGAAGAAGCATTACAGGGGAGTTAGACAGAGGCCATGGGGGAAGTTTGCAGCGGAGATCCGTGACCCAAATAGAAAGGGGACTCGAGTTTGGTTAGGAACATTCGATACTGCTGTGGATGCAGCTAAGGCATATGACAGGGCAGCGTTTAAGCTTAGAGGAAGCAAAGCAATATTGAATTTCCCACTCGAAGTTGCAAACTTTAAGCAACAAAATGATGAGACTAAAACAGAGATGAAGTCATCAGGCAGTAAAAGAATGAGAGGAGAAACAGAGGAATTGGTAATCAAAAAAGAAAGAAAATTACAAGAAGAAAGAGTAGTGCCAATGGCATCTCCATTAACACCGTCAAATTGGTCGACGATTTGGGATGGAACAGGTATTTTTGAGGTGCCACCATTGTCACCCTTGTCTCAGCTTGTAATGATATAA

>Solyc03g093560.1.1 (SlERF3-11)

ATGGGTTCTCCACAAGAGACTTGTACTTCACTTGATTTGATTAGGCAACATCTTTTTGATGAATCTCTGGACCAGACTTGTTTCTCTTTTGAAACAACTCAAACTTCAAATCTTGATGACATCGCAAGCTTCTTTAATGCTACTTCAAAAACAGAGTATGATGGTTTTTTCGAATTTGAGGCAAAACGACATGTTATCCATTCAAATTCTCCGAAACAGAGTAACTTGAGAGAACGGAAGCCATCTCTGAACGTAGCAATACCGGCGAAGCCTGTTGTTGTTGTAGAGAACGTTGAGATTGAGAAGAAGCATTACAGGGGAGTTAGACAGAGGCCATGGGGGAAGTTTGCAGCGGAGATTCGTGACCCAAATAGAAAGGGGACTCGAGTTTGGTTAGGAACATTTGATACTGCTGTGGATGCGGCAAAGGCATATGACAGGGCAGCGTTTAAGCTTAGAGGAAGCAAAGCAATATTGAATTTCCCACTCGAAGTTGCAAACTTTAAGCAACAAAATGATGAGACTAAAACAGAGATGAAGTCGTCAGGCAGTAAAAGGGTGAGAGGAGAAACAGAGGAATTAGTAATCAAGAAGGAAAGGAAAATAGAAGAAGAAAGAGTACTCCCAACGGCGGCGGCTCCATTAACACCGTCAAGTTGGTCGACGATTTGGGATGAAAAGGGTATTTTTGAGGTGCCACCATTGTCACCATTATCTCAGCTTGTTATGATATAA

>Solyc03g093610.1.1 (SlERF3-12)

ATGTATCAACTTCCCACTTCTACTGAGTTAACTTTTTTTCCGGCAGAATTCCCGGTGTATTGCCGGAGTTCAAGTTTCAGTAGTCTCATGCCATGTTTAACCGAATCATGGGGTGACTTGCCGTTAAAAGTTAACGATTCCGAAGATATGGTAATTTATGGGTTTCTACAAGACGCTTTTAGTATCGGATGGACGCCGTCAAATTTAACGTCCGAGGAAGTGAAACTCGAGCCGAGGGAGGAGATTGAGCCAGCTATGAGTACTTCTGTTTCTCCGCCGACAGTGGCTCCAGCGGCTTTGCAGCCTAAAGGAAGGCATTACAGGGGCGTTAGACAAAGGCCATGGGGAAAATTTGCAGCGGAAATAAGAGATCCGGCTAAAAACGGCGCACGGGTTTGGCTTGGAACTTACGAGTCGGCTGAGGAAGCCGCACTCGCTTATGATAAAGCCGCTTTTAGGATGCGCGGTACTAAGGCTCTATTGAATTTCCCGCATAGAATTGGTTTAAATGAGCCGGAGCCGGTTAGAGTGACGGTTAAGAGACGATTATCTGAATCGGCTAGTTCATCGGTATCATCAGCTTCGGAAAGTGGCTCGCCTAAGAGGAGGAGAAAGGGTGTAGCGGCTAAGCAAGCCGAATTAGAAGTTGAGAGCCGGGGACCAAATGTTATGAAAGTTGGTTGCCAAATGGAACAATTTCCAGTTGGCGAGCAGCTATTGGTTAGTTAA

>Solyc03g114440.1.1 (SlERF3-13_DREB)

ATGGAAAACTCACAATCCCCATCAAAGTCCTTAAACAACTCATCCAAAAACATCCAACAAAAAATAAATCCAATTTCCTCAAATGATGGAAAGAGATTTATTGGAGTTAGACAAAGGCCATCAGGCAGATGGGTTGCTGAAATCAAACAAACTTCACAAAAACTCAGGCTGTGGCTTGGTACTTTTGACAAAGCAGAGGAAGCTGCTATGGCTTATGATAGTGCTGCACGTCTTCTTCGTGGGAAAAACGCGAAAACAAATTTCAACAACCATGGAATCTTTAAACCTAATGAAGAGAATTACAGTTTGTTGGAAAAAAATCCAAGATTATATCAACTTATGAAACATGCCATCATGAAGAAATTTGCAGGGAAATATCAAAATAATGAATGTTTAGAAACAGAGGAGGTGGTATTAGTTGAAGAAAGTATAAATAAAGAAGAGATCTGTGCGATTCAATTACAGGGAAGTTCAAAGGTTTATTCATCAGTGATTGTCGCTCCTTCTTTTAGTAATAATGAAAAATCGTATCAACTTTAG

>Solyc03g116610.3.1 (SlERF3-14_DREB)

ATGGTACAGGCAAAGAAGTTCAGAGGTGTCAGGCAACGCCATTGGGGTTCTTGGGTCGCTGAAATTCGTCATCCTTTACTGAAGAGGAGAGTGTGGCTAGGTACGTTTGAAACTGCAGAAGAAGCTGCCCGAGCGTATGATGAGGCTGCTGTTTTAATGAGCGGACGTAACGCCAAAACAAATTTCGCAGTGCAAGCAATGGATGAAAATAAAAAAGACAATTATAAATCTAATAATTCGACATTATCAGGATCATCATCATCACTCTCTGCTATACTTAGCGCCAAACTTCGGAAGAGCTGTAAATCGCCATCGCCGTCTCTCACTTGTCTCAGGCTTGATACAGAGAGTTCCAACATTGGGGTTTGGCAGAAACGGGCCGGAGCCCGGCCTGATTCTAGCTGGGTCATGACGGTTGAATTTGGAAAAAAGAAGATGATTAATGATAACGAGCATATAATTATTCCGGACGAAAACGTAACTTCTTCGAGTACGTTTTTGTCTCAGGATAATTCGATCGAGATTGAACAAGGCAAAGAATGTGGTGTGATGAATGAAGAAGAACGAATGGCGCTTCAAATGATTGAGGAACTCCTCAACAGGAATTAA

>Solyc03g117130.3.1 (SlERF3-15_DREB)

ATGTCTCGACCACAACAAAAATATCGAGGTGTTCGCCAGCGCCATTGGGGATCTTGGGTTTCCGAAATTCGTCATCCTTCATTGAAAACACGGATATGGCTGGGAACCTATGAGACATCAGAGGATGCTGCGAGGGCATATGATGAAGCAGCGAGACTCATGTGTGGTTCAACAGCACGCACTAATTTCCCATACAATGCAACAGAATCTTCAAGGTTTCTGTCTTCTGCTTTGATAGCTAAACTTCAAAGATGCAACATGTCATCTCTCACTGCTACGTCAAGAAGACCTGGAAAGACAAGATTGGAAGATAAAAAAGAGAACGAGATATCCACGCTTGTTAGAGACACGGGAGATGGAGAAGAAAGGCAGAGTGAGAGTGCATCACAACAATACATGAAGGCACTTGAAGATGAACATATAGAACAGATGATTGAGGAATTGCTTGATTATGGATCTATTGAGATGTGTTCTGTTCGCAATGAATAA

>Solyc03g117230.1.1 (SlERF3-16)

ATGTATAATTCCCTTTCTCAAAAAGCTGAACCATTCTTCTTCTCTACACAAAATCCCCTTTACCCAAATGAACCAAATCGTCACCATAATTTCTCCATTGACAACACTAATTATTCCCTCGACCCATTTTGGGATAATTACTCTGTTTCTGCTAATGAAAATTTTAATGAAAAATCCCCTGTTCTTGAAGGAATCGCTGCTGTTGTTGGAGAACATGTTCTTTTCGGTCACAGTAACAATAATAATAATAATCAAAACAAGAATGATGATCCAAATTCATCAGCAATTTCAATTCTGAAACGCACGTGTCCTGAAGAAAAGAAGAAGAATAACAATAATGTTCAATCTGTGGAGAAGAGTTACAGAGGAGTGAGAAAGAGGCCATGGGGGAGATGGTCAGCGGAGATTCGCGACCGTATAGGGCGGTGCAGACACTGGCTGGGTACGTTCGACACCCCGGAGGAGGCGGCGCGTGCGTACGACGCGGCGGCGAGGTGGCTGAGAGGTTCAAAAGCTCGAACGAATTTCCAGATTCCTCCGATTGTCCCTCTGCCTACTTCACCAACATCCACGTCATCATCTTCAAATTCATCTCGCGAAATGAAAAAGAAGAACAAAAATGGTGGCAGTATTGCTAATAATCAAAGAAAATGCTCAGTTGTAACTTCAGCTGCCCATTTATTCAGCTCAAATGAATTAAGTAAAGGGGTAAGTGTCACTGTGGAGCTTGATCTCAATCTCGGCTTCAGGAGGAAGTAG

>Solyc03g118190.4.1 (SlERF3-17)

ATGAAAAGATCAAGCTCTAATAATGATCAGAGGGATGAAAAAGACACTAGTAACATATTCCCAATATACTCATCAGCTCGATCTCAACATGACATGTCTGCTATGGTTTCTGCTCTCTCTCAAGTCATTGGTAACTCTTCGTCTTCAGCTAGTGGTGATTCCTCCTCAGTGCATGTTAATCCACTAACCCTAATTCAGCAACATCAATCTCAATCGTCTACTCAAGATCAAGAAAGAAGGCGATATAGAGGAGTTCGTCAAAGGCCATGGGGTAAATGGGCAGCGGAAATTCGGGACCCGAAAAAGGCAGCTAGGGTTTGGTTAGGTACTTTTGAAACAGCGGAAGGTGCAGCACTTGCTTATGATGAAGCTGCTCTAAGATTCAAAGGAAACAAAGCTAAACTCAATTTCCCTGAAAGAGTTCAAGGACAGTTTTTCCAATGCTATGATCAACCTGCCACGTCATCAAACAACACATCTGAACAAAATTACCCTAATGTTCATCACTATGCTGATTTATTACTTCGTACTGACAATAATATCGATCTAAATTTCGATGTTTCACCAAATACTTTTTATCACTCTTTTGATATTTCACAATCATCAATGGAAGTTCCAGTTTATCATGAGGAGCAGCAACAAGTTATAACTACGCACGAAGAAGAAGAAGAAGATTTTGTGAAATATCGTGGATCACATTTTGGAAATTCTACTTCAAGTGGTGGAACTAAATAG

>Solyc03g119580.1.1 (SlERF3-18)

ATGGATGGTTGCATTAGTTCAGTGAGGAAAGTTCGCATAGTTTATGATGATCCTGATGCTACGGACTCTGAGAGCGATGATGATCAGAATGCTGCTCGTTTCGATAAAAATGTGAACAGAATCAAGCGTGTTGTTAAGGAAATTGTTATTCCTGTTGTTTCATGGGAGAATGATTTTAAAAAATGTTCTAAACTTGATAACATTAGGATTAAAGATTCCAAGAAGATTCATGAAAATAAGAAGGTGCAGCTAAAATCGACCGCGTTGCCTAAAGGAGTTAGGATGAGGAAATGGGGGAAATATGCAGCTGAGATCAGAGATCCCTCGCAGGGGAAAAGAATATGGTTAGGGACTTTTGAGACTGTGGAGGCGGCTTCACAAGCATACGAGGCAAAGAGGGCTGAATTTGATAGGATTATTTCATTGGGGAAGGGTAAGAATTTGAGCCCTGGTCCTGCTGAGTGTTCTATGGCGTGTACGTCTCATCCTACAAATGGGAAAAATCGTGTGTACTCACACCCTTCCCCGTCTTCGGTGCTAGATGTCCCCACATCATCAGCTGCTGCCCCGGTTGAGTCCAATGAAAATCTAACGAGAGATATGGCTCGAATGCCAGATTCAGGTTCTGAAGATTTTAGCTTGAGTTTTGAGGACCAAATGCTTCATGAATTTATCAAACAAAGACAGGGCATTTCAGAATTGATCGAACATCCTTTGATAGAACAAGGCTCGATCAGCAATAGCGTTATGGAGATGACTGAAGTGAATATCAGGAAGAAAACGAAAGCCAGACAACCAACGATAGCAAGTTGTAAAATATTGACAAAGGGAACTGAGGACTACAGTAATGACAAGTCCATTTTCAGTGTATTAAACGAGCCTACAATCATGTCTCCTATTCATACAGAGCTGCTTCACTTGAACATCGAGGAAACAGCAGTTACGGGGAATAGCTTGAAACTTCTAGGCTTTGATGACAATGCTTTATTTGATAAAGATATATCACAATTATTTGATCCATATGCAGATGCTATATGCTTGGACAACACTTTTCAATGCTGTGATGGTTGGAATGAGTGTGTTTATTGCAAAATTTTCAAAGATGAAGTCGACCTAGATGAAGTTGACTTAAGATGGTTAGATGCAGTTTTGGTATAA

>Solyc03g119800.3.1 (SlERF3-19_DREB)

ATGAAGCCGCCACCACCTCAGCCAAATGACGGTATTAGAACACGAAGAAAACCATCATCAAGGGGCCATCCAAGATTTGTTGGTGTTCGCCAAAGGCCATCAGGAAGATGGGTTGCAGAGATCAAAGACTCTTTGCAAAAGGTGAGGCTATGGCTTGGTACTTTTGACACTGCTGAGGATGCTGCAAGGGCTTATGATCAAGCTGCTCGCACATTAAGAGGGGCTAATGCTCGTACTAATTTTGAACTACCCGCGTCTGATTCCCAACAAGGTAGTAGTATTGTTGAAAATTCTGAGCCTTTTAACTTTGAAGAAGCCTGCAGAACAGAGGAACCTGAAAATAGTCTTGTTGGTGCACTTAAAGCTAAACTCTTTAACAGCAAGAATTCAAGATCTTTCATTCAAGCTTATGCCTCTAATTCATCTTCTGAATTAGCATCCAAAGTTAAACCATCAGTACCTTGTAGTGAAATCAAGAAAACATCCACACAAAGTGAGAAATTACCTAAAATTGGACATGTTAAGAATTGCTCTGATTACTTGTTCAGGGGAAATCACAATCTTGATTACATAAGCCTCATGACGAACGATTATCAGCCCATGATCTCATCGTCACAATGTCAAGATAACCAGCTGTATAGTGGGACAGATTCAGCAATAATGTGGCTTAACGAGCAAGGAACATTACCATGGGGAGAACCACAAATGAGTCAAGTCCAA

GACGAGGTTTCATTTGATACTACTATAAATATAAATAATAACATTAACACTAACATTTTTGGTGGTGGTAATAGTACTACTGCTACTTGTACATGGCCAGTTTCAGTTAGTGAACCAACGGTTGATTTGTCTGCATTTGGGGGAATAGATGTTTTAAATGGGTCAATAATGATGCCTGATATGAATGGACTAACACACACATCTTCAGTTACAACTGAACAACAGTTCCTGCAGTTCGAAAATGGGCTTTGGGGTGTTGGTGACAGTGCAGGTTGGGATCCCTTCCTTTTATCATCTGTGTGA

>Solyc03g120840.3.1 (SlERF3-20_DREB)

ATGGCGGAAGTTGAAGTGCAAAGTTCAGAAGCAGATAGCTCTAATACTACTTCCTCTGTTTCTTCTTCGTCGTCGTCTTCGTTGTCCGTCAATTCCATGCACAAATCGGTTTTTGATTCGTCAAACAAATTGCCGGAAGAGAAGAAACGGCGGACAAAGGGAACGAAGAAGCAGAAGTCTATTAATAATAATAGTGAGAGTAGACATCAAATTTACAGAGGAGTTAGGATGAGAAGTTGGGGAAAATGGGTATCTGAAATTCGTGAACCGAGGAAGAAATCACGTATTTGGCTTGGTACTTATCCTACAGCAGAAATGGCCGCTAGGGCACATGATGTTGCAGCGGTGAGTATAAAAGGAAATTCCGCCATTCTCAATTTTCCTCATCTAATCGACTCGTTGCCTCGTCCATTGTCAAAGTCACCTAGAGATATTCAAGCTGCTGCTGCTTTAGCAGCATCAATGAGGGACCCACCTTCTTCATCCTCGTCGGTCTCATCTTCAATAACAACAACAATATCAGCGGGATCTGAAGAGCTTTGTGAGATTATTGAACTGCCTAATTTAGAAGAAAGTGACGATTCAAAAACTGATTTAACGCTGAGCGAATCGGTTGAAGGATTACTGTACTCACCGTGGTGGGCAGATCACAGTACAGATTTTTGTGGTTATTTTCTGGAGCAGTCTGCAGCTGGCGCTGGAGAAAGTTTAATTTCTTGCAGCTTTGAGACACTGAAATGGGCTTGTTAA

>Solyc03g123500.4.1 (SlERF3-21)

ATGTGTGGTGGTTCTATAATCTCCGATTACATAGACCCTAGCCGGACTTCTCGCCGGCTCACCGCCGAGTTTCTATGGGGTCGTTTCGATCTCGGTAAGAAGCAAAAAAATCCCAACAATTATCACTCTAAAGCTAAGCATTTGCGATCTGAAGTTGTTGACGACTTTGAAGCCGATTTTCAGGACTTCAAAGAGTTATCCGATGATGAGGATGTTCAAGTCGATGTCAAGCCATTTGCCTTCTCTGCTTCCAAACACTCTACTGGTTCCAAATCTTTGAAAACTGTTGATTCAGACAAGGATGCTGCTGCTGATAAATCCTCTAAGAGAAAGAGGAAGAATCAATATAGAGGGATCAGACAGAGACCTTGGGGTAAGTGGGCAGCTGAAATACGTGACCCAAGGAAAGGGGTTCGGGTCTGGCTGGGAACCTTCAATACTGCAGAAGAAGCTGCCAAAGCTTATGATATTGAGGCGAGGAGGATCAGAGGCAAGAAGGCTAAGGTAAACTTTCCTGATGAAGCTCCCGCCCCTGCATCAAGACACACTGTTAAGGTGAATCCTCAGAAGGTCCTTCCTGAGGAGAGCCTGTATTCACTTCAGTCCGACTCAGCAATCATGAACAGCGTGGAGGATGACCATTATGATTCTTTTGGATTTTTTGAAGAGAAACCCATGACAAAACAGTATGGATATGAGAATGGGAGCAGTGCTTCTGCAGATACGGGATTTGGTTCGTTCGTCCCTTCAGCTGGCGGTGATATCTACTTCAACTCTGATGTAGGAAGCAACTCTTTTGAATGCTCTGATTTTGGTTGGGGAGAGCCATGCTCCAGGACTCCAGAGATATCATCTGTTCTGTCAGCTGCTATTGAATGTAATGAAGCTCAATTTGTTGAAGATGCCAATTCTCAGAAAAAGTTGAAATCATGCACCAACAACCCCGTAGCTGATGATGGAAACACCGTTACTATGGTACCTGAAGAGCTTCCAGCTTTTGAACCTCAGATGAATTTCTTTCATCTCCCATATATGGAGGGAAATTGGGATGCATCAGGTGGTAACTTCCTCAACACAAGTGCAACTCAAAATGGTGGTGAAAATGCTATGGACCTGTGGTCCTTTGATGATGTTCCTTCTTTAATGGGAGGTATCTTTTAA

>Solyc03g124110.2.1 (SlERF3-22_DREB)

ATGGATATCTTTGAATCCTATTATTCAAATTCTTTCGTTGAATCATTATTATCATCGTCATTATCAATATCTGATACTAATAATCTCAATCACTACTCCCCTAATGAGGAAGTTATTATTTTAGCTTCGAATAACCCGAAAAAGCCAGCTGGCAGGAAGAAGTTTCGAGAAACTCGACATCCAGTATACAGGGGAATCAGGAAGAGGAATTCAGGAAAATGGGTTTGTGAAGTCAGAGAACCAAATAAGAAGACAAGGATTTGGCTTGGTACTTTTCCTACGGCTGAAATGGCGGCTAGAGCTCATGACGTGGCGGCTATAGCATTAAGAGGCCGTTCTGCTTGTTTGAATTTCGCTGATTCAGTTTGGAGGTTGCCTATCCCTGCTTCCTCCAACTCTAAAGATATTCAAAAGGCGGCCGCTGAGGCCGCCGAAATCTTTCGATCGGAAGAAGTTTCAGGAGAATCTCCTGAAACGTCAGAAAATGTGCAAGAGAGTAGTGACTTCGTGGATGAGGAAGCGCTGTTTTCCATGCCAGGATTACTTGCAAATATGGCAGAAGGACTCATGCTACCTCCTCCCCAATGTTTAGAGATCGGAGACCATTACGTTGAATTAGCTGATGTGCACGCTTATATGCCTTTATGGAATTATTCTATATAA

>Solyc04g007170.3.1 (SlERF4-1)

ATGCTTAAGCCTTTGTCATCAAAATTTGAGAATCTTGGTAGAAACATGAAGAAAAAAGTTGATACAAACAGATTAGTGAGGAAAATTAGGATTGTTTGTAATGATCCTGATGCTACTGATGATTCCTCGGACGATGATTCGAGGTGTAAGCGTTTCGTTAGAGAGATTAAGTTGCAAATTGGCAACTCTTTTAATCTTAGAAAAGCCTCTGAGATTGAATGTTCATTTCAAGATAGTAACAATGGAGAGAAAAAAACAAAGAAAGAGGGTTTAGTTAAACCCCTGATTCAACCAAGGCCAGCAGGGGGTTTATTATCGAAATACAAAGGTGTTCGTCAACGAAAATGGGGGAAATGGGCTGCTGAAATTCGTGATCCATTTAAAGGCAGACGTGTTTGGTTGGGTACCTATAATACCGCTGTGGAGGCTTCTCGAGCTTACGAATTGAAACGTCTTGAATTTGAAACTAGGGCGAAGATCAGTAGGACAAATGTGTCTAAGCAGAGTTCTGGTTCAATGGTCTCCGAGTACCAAAATCAGAGCCAAAATGTTGCTAGTGGTGTCTCAGATGACTATGCAGAAAGCTCGGTATCACGTACTTCACACTCCTTGTCGTCATCATCAGTTCTTGAATTGGACACTTTAACTTCTGTATCCGCATCTGCTCCAATTTTAAGACTTAATGGCCCAAACGATAATGAAAAGGTGAGCAATGTTGCGCCTTTGGAGGCTAATGTTGTCGAGCAAGAAGTGCCTGAGTTGGCTATGATGGAGGAAACTCTACCTCTGTCTCAAATTGGTGAAAGTATGGATCTTGATTTGGAGCTTGAGTCGTTCCTCATTGGCGCTGATGATTTCAATCAGCATTTGGATGAGTTTGTTGTCAATGATTTTGAGGACCCTCCGGTTTATCTGATCGAGGGTGATGAGCAGTTGCCTACTGGTTTGCCTGATTTTGATGACTTCAATTTCGATGGCTACAATGAATCCTTTTCTTGGATGGATGATGCTCCACGGACAAATGGAACACCCCTCAATATAGCATGCCCATAA

>Solyc04g012050.3.1 (SlERF4-2)

ATGTGTAAATTCAAGGTGGCGAATTATAGAAAGAAAGGGAATTATAAGGCAATTATAAGAGATGATGAAGAAGAAGAAAGTGGGATTAATAATAATGATAATATTAATGTGATGTTTTCTGGGAATATTAATAGAGAAGAAGAGATGTCTATGATGGTTACTGCACTGACACGTGTCATTACTGGTGAAGATAGAATATTAAATCAAGAAAATAATAATAATAATATTATTGGTTCTTCATCTTTGAATGAATTTTCTGGAGGCGTTGGAGAAAAAAGAGGACGTGAAGAGCAGCAATATTTCTTGTCTGATATTATTAGTTCATCAAAAGTTGGTGAAGATTCAAGCATTAATAGAACTATTGCAAGCAACACACCAACAACAGAAGCAACATTCATATACACTACTCCAACATATGATCATAATAACAACACCAATATTATTGATTGTGATCAACCAAGAAGAAGATATAGAGGAGTGAGACAAAGGCCATGGGGAAAATGGGCTGCTGAAATTAGAGATCCATACAAAGCTGCTAGGGTTTGGCTCGGAACATTCGATACTGCTGAAGATGCTGCTAGAGCTTATGATGAAACTGCTCTTAAATTTAGAGGTAGCAAAGCCAAACTCAACTTCCCTGAAAATGTTAGGCTCTTGCCTACTTCTTCAATGGAAAAAGGGTCTCTGACAAACACATTTCCCTCAAACTCCGTGGAAAGTAACTTGGAATTTTTGACGAAGAAAAAAGTTCTGACAAGCACATTTCCTGCAGAAAATAATATGGTATTTTCAACAGAAAGACAGTTTATGACGGACACATTTCCCACGGAATATGTACCTGTTTCTAGTATTATTTCTCCTAATACTTCTCCAAATACTCTTTCAACAATCTCTTTTTTCCCTAATAGTAATCCAATAGCTCACACTCAGTTATCTAATAGCAATAGTGATGACATAAACACTCAATATTTCTTGAATGGTGATTTTCAAAGGGGCAATTCATCTCTAAGTTTGTTAAACCAATCCATGTTTTCTACAACATTCCAGTATTCTTCTTCTTCTCCATCATCGTCTTCTTCTTCTCGAGTTCCTTTTGTTCCGAGTATGTATTTTTCAGGTTCTCAACCTACTAGCCAGAGTAGTGAAAATGATTTTCGGGCTACTTCGTCAGATTCAGCTCATCACCCTCCAAAATGA

>Solyc04g014530.1.1 (SlERF4-3)

ATGGAATCTTCATCCCCTAAAACTCAATATCCAAATTTCAATTTCTTCCAAGATCAATCATCATTACCATGGAATGATCAACATTTCTTAGATGAATATTTGACTAACATCGACCAAAACAACGATCATTCTCTACCAGGAAGTACTTGTTCATTCTTAACCTCGAAAGAAAGTTATAGACGGGAAGTTTCCTCCTCCAACCTACATCAATTACCAAGAAGTTGGTCATCCTCAAACGATACGAATTCCTCTAAAGAAAGCAATAATCGTCATGAAATCGAAGAGGTCACGTCTCATCACCATGATAAGAATAACTCCACCAAACACTACATAGGAGTTAGAAAGAGACCATGGGGAAAATATGCAGCGGAAATAAGGGATTCAACAAGAAATGGGATTAGGGTTTGGTTAGGAACATTTAATACTTGTGAAGAAGCTGCTTTAGCGTATGATCAAGCTGCACTTACAATGAGAGGTCCATTGGCACTTTTAAATTTTCCAATGGACAAAGTAAGAGAATCACTTGAAAATATTAAGTATATTTGTGAAGATGGGATTTCACCAGCTGCTGTTTTAAAGGCAACAAATAAAATGAGACGTGTTAAACATAAGAGAAATAGGAAGAAGAGAAATGTTTTGGTTTTTGAAGATTTGGGTGCTGAATTGTTAGAGGAACTTTTGACGAGTACTTCCTCTAATTAG

>Solyc04g050750.2.1 (SlERF4-4_DREB)

ATGGAGGAATGTGGCAAAAGTAGGATTGAGAGTGTAGAATCCAAGAGGAAATCACGTTCTAGAAAAGGTTGTATGAGAGGTAAAGGTGGACCAGAGAACGCTTCATGCACGTATAGAGGCGTGAGACAAAGAACGTGGGGTAAATGGGTAGCAGAAATTAGAGAACCGAATGGGGGTGCACGAATTTGGTTAGGTACTTTCAATACTTCAGTGGAAGCAGCCAGAGCCTATGATGATGCAGCACGCAGGCTTTATGGATCCGACGCCAAACTCAATCTCTCAGAACAAGAATCAACAGATGTTGGCATAATTGAGGATGGGGATGGAGAATGTTCAGTACTTGAGGAAGCATCCATATTTAAAGATGGAAACGGCAAGTATTTAGTGTGGGATACGCCAGCCCCTAGTTTGCTTGGGGTCGATTTTCATGGAGACGCTACCACATGTTTCAATTGGAAAAATCAAACTGAAATGATGTATTTTTAA

>Solyc04g051360.3.1 (SlERF4-5)

ATGTGTAATATTGTTCACTACAAGGTGGCGAATTCGAATGATAACAGGAGTAGTAGACAAGACGATGAAGGGATTAATGTGTTTAATACGATGTTTCAAGGGAATATTAATAGAGAAGAAGAAATGTCTGTTATGGTTTCTGCATTAACTCGTGTTGTTGTTGGTAATCATCCTAGTGAAAATATCGAAAATCATCATCAAAATAATACATTGATTTCTAGGGGTGTTGGAGAAAAAAGAGGACGTGATGAAGTATTATTACATGGAACTAATTCTTCTCATATGATATTATCATCAGGTGGTGAAGGTTCAAGCATTAGGACAACAAGAGAAGCAACATTCATATACACTAATTCAACAAACAATAGCATTATTGATGAATCTGTTAATAATCAAGTAAGACGACGATACAGAGGAGTTAGACAGAGGCCATGGGGGAAATGGGCAGCTGAAATAAGAGATCCATATAAAGCAGCGCGTGTTTGGTTAGGTACTTTTGATACTGCTGAAGGGGCTGCTAGAGCTTATGATGAAGCTGCTCTTACATTTAGAGGTAGTAAAGCAAAACTAAATTTCCCAGAAAATGTTACATTATTAGTGCCTTCTTCAATTCAACAACCCATTTACTCCTCACCCGACCCAGCCATTTCTCCGTATCGTTCCAATTTTATTATTGGACATACCTCTACGGAAGTGGAGCCTATACTTCACACCAATCCTTCAAATTTTATTGAGCCTATAGCTCACACTTCATCTCTGTATCGTTCCAATTTTATTGAACGGAATCATCACATGGTGCAGCAGGAGCCGTATTTTCAGGCAGGTAGTACTAGTGGTGGAAGTGATTTTCATCAAACTACAAATTCTTCTAATTCGTCAATTTATGATCACCCTTCTTCTTCATCTGGATAA

>Solyc04g054910.4.1 (SlERF4-6_DREB)

ATGGCAGCAGCTATAGATGTATACAGCAGCAGCAGTAACTTGTCAGATCCTTTAACAGAAGAACTTATGAAAGCACTTGAACCTTTTATGAAAGGTATCTTGCAAATCCAAGCTCAGATCCAATTTCAAAATCAACAGCAACAACTACAACTATTACATCAACAACAACAGAGCTTAGTCCCAATGAAGCAAACGGGTGCTACTTCTTCACAGAAGGCTACTAAGCTTTATCGTGGAGTTAGACAACGCCATTGGGGCAAATGGGTTGCTGAAATTAGACTTCCTAAGAACAGAACTAGGCTTTGGTTAGGCACTTTTGATACAGCTGAAGAGGCTGCTTTGGCTTATGACAAAGCTGCTTATAAGCTAAGAGGTGAGTTTGCTAGGCTTAATTTTCCACATCTAAGGCATCAATTAAACAATGAATTCTCTGATTTCAAGCCTTTGCATTCCTCTGTGGATGCTAAACTTCAAGCCATTTGCCAAAGCTTGGCTAATCCCAAATCAGATGACTCGTGTTCTAAATCTAATTCCAAGCCAAGAAAGTCCAAAACTGCAGCAGTTTCAGTGGATTCAAATTCAGCTCAAGAATCTTCATCAAAGTCAGAAATCACCACAGATGATTCATTGAAAGAAGAATTCAGCTATCCAGAAAATGGTACTATCAAGATTGAGGCTTCATCATCATCATCACCCCCTACACCCTCTGAGGAATCATCATCTTCGTCTGAGTCTGATATTACTTTCTTGGATTTCGCTGAACCATCTTTCGATGAATCAGAAAACTTCTTTTTACCCAAGTACCCTTCCGTGGAGATTGATTGGGCAGCTCTTTGA

>Solyc04g071770.3.1 (SlERF4-7)

ATGTGCATATTAAAGGTGGCGAATCAAGGAGATTCCGGCAAGTATGACAGAATTCCGTCGACAGCAGGTGATTCTGAAACTACAACGAATGAAGGAATTCCTCAACCGTATGAACAGTCGCAATCTTTTGAAGAAATGTTACAGCAACAAATACAGCAAGAAACAGAGTATTTGATGTCGGAATCGGCTAATCCGATGTATACAGGGTATAGTCAGTCGAGGGATATGTCGGCAATGGTGACGGCGTTAACACACGTGGTTTCGGGTCGGAGAGAGGCAGAGTGGGGTTACAGGCCGGATATTAGCGGTGTTACGACTTCGTTTGGTGGTGGTGGTTCAGGGAGTATTTATTCGGCAAATTCACCGTCTTCTTCGAGTTCAGGATCATGGGCTGGACAGAAAAGAAGACGTGATCAAGAAGAAAGTGTTACTGGAGAACAAGCTCAAAGGGGTTATGGAGGTATCGGAGAATTTAAAAATGGAGAATCATCTTCCTCTGTTAAGCTCGAAGAAGATACAAGCCTGGCAACACCACAAACGAGCAGCATATCGACAACTACTGCGTCGCAGACACCACCGCAAGCATCGGAAGTAACAGGTGAAGAAACAGGGGAGAGGAAGAGGAGATACAGAGGAGTAAGACAGAGACCATGGGGTAAATGGGCAGCAGAGATAAGAGATCCACATAAAGCAGCAAGAGTTTGGTTAGGCACATTCGATACAGCAGAAGCAGCCGCTAAAGCTTATGATGACGCCGCTCTTAGATTCAGAGGAAACAGAGCTAAACTCAATTTCCCTGAAAATGTTCGGTTATTACCACAACAACAACAACAACCCACAACAAGATTAGCCATATCAACCTCATCATCAACCGCAGCTCCGCGATTCCAATTAATGTCTGCAGCATCAACGCGATCACCATCACCATCACCATTTTTTTTTCAATCATATAATCAACCTCCGCGTCAATCTGATCAGCAGCATCAGAATCAGCAACAACAGTTATTTCAGAGCTCAGATATGGCTAGAGATTATTGGGAATACTCGCAATTACTTCAAAATCCAATAGATTTCCATGGAGGACAACAATCATCATCTTTGTTAGAACAAATGTTACTAGCTTCATCATTGGGAGTGTTACATTCACATACATTTCCTTCTTCGTCATCTTCTTCATTAGCAACTTCTGCAGCTTCTTCCACTACTTCCCCTGCATATCCCCTGTTTTACTCTGCTCAACAATCACGCTTCTTTCAGCCACAAACTCATCAAAATCAAAGCAATAGCAGTAGCAACAGCTCAAATTTTCCTCCACCTTTTTGGACTAGTTCCGGCCACTATCCACCTTCTTCTAGCTAA

>Solyc04g072300.1.1 (SlERF4-8)

ATGGAAAATATTACTAATACTCCTAAATTGCATGTTAGAAATTTAGTTAGAAGAAGTTCAAGACATTCAACAAAATATCATGGTGTAAGAAGAAGGCCATGGGGTAGATATGCTGCTGAGATTAGAAATCCAAATACAAAACAAAGACATTGGCTTGGTACATTCGATACTGCTGAAGAAGCTGCTCTAGCTTATGATATTTCTTCTATTAATTTTTGTGGTATCGAAAATGCACGTACTAATTTCGTATATCCTTTTTCGTCTTTTCCTTCACCTTATAATAATTCTCCGGCACCACCATCACCACCACCTCAACCACTACCACCACCACCACCGTCAACGCCGGAGTTGGAAGTGGTTGAAAATAAATGCATGAAGATTGAGATGAATGATGATATTAATGATGATGATGAGTCTCTTGTTATTGCTTCTATTTTACAAAGTTTTCGTTATACAAACACGTTAGATAAGCTGTCTCTTTGA

>Solyc04g072900.1.1 (SlERF4-9_DREB)

ATGAATTCCCAAATCTTTTCAACTGGGTTTTCTGGGTATGGAATGGAGCAACAGGGTTCAATTGGGCTGAATCAGTTAACCCCAATTCAGATCCAGCAAATTCAAGCTCAAATCAACTTTCAAAACCAACAACAACAGCAGCAGCAGCAGATGATGTTACAGACTGCCCATCATGCTTCCACCATGAATTTCTTGGCTCCAAAGCCGGTTCCAATGAAGCAATCTGGGTCGCCACCAAAACCCACGAAGCTCTACAGAGGTGTTAGACAACGCCACTGGGGTAAGTGGGTCGCTGAGATCCGTTTGCCTAAGAACCGAACCCGCCTTTGGCTTGGTACATTTGACACCGCTGAAGAAGCTGCTCTGGCTTACGACAAGGCGGCGTATATGCTTCGTGGCGACTTTGCTCGACTGAACTTCCCTCAACTCCGCCACAACGGCAACCTAATCGGCGGCGACTTTGGTGAATACAATCCATTGCATTCCTCAGTTGATGCTAAGCTAAAGGACATATGCCAAAGCTTGGCACAGGGGAAGAGCATTGACTCTAAGAAGAAGAAAACCAAAGGGTTGTCGGCGGAGAAAGCGGCGGTGGTGAAGATGGAGGAAGAGGAGAGCAAAACAGCAGAAGTTGGATCCGAAAGTGACGGGTCCCATTCCGGTTCCGGTGGATCATCGCCGGTGACCGAACTGATATTCCCGGAGTTCACTGAGGAAGAGCCAACTTGGGACATGTCAGAAAATTTTTTGTTGCAGAAGTATCCATCTCATGAAATTGATTGGGCCTCTCTATAA

>Solyc04g078640.3.1 (SlERF4-10_DREB)

ATGGAAGGAGAGAAGAGAAAACAGAGGCAACACCAGCAAGATAAGCCATACAGAGGAATACGGATGAGGAAGTGGGGTAAATGGGTAGCTGAAATTCGAGAACCAAACAAACGCTCTCGAATTTGGCTTGGTTCTTATTCTTCCCCTGTTGCTGCTGCTCGTGCTTACGATACCGCTGTATTTTACTTGCGAGGTCCTTCAGCTAGGCTTAATTTCCCTGAATGTATAGTGGATGACCATGAAATTCACGATCTATCAGCTGCTTCTATTAGAAAGAAAGCTACTGAGGTAGGTGCTCGAGTTGATGCTCTGCAAACTGCGATTCACAATTCTACTGTTAATTCTGTTGAATCAAACTGTAATTCGAATTCTAAATCTACAAGGATGATGATGAAGCCTGATTTGAATGAATATCCTAGCCCGGAAAGTTGCGACGAAGATAACTGA

>Solyc04g080910.1.1 (SlERF4-11_DREB)

ATGTCATCAAACTCCAAGAAGTTGATTCCAGCTACATCCCGCAAGGGATGTATGCGGGGCAAAGGTGGCCCCGAAAATGCTAATTGCACGTACAAAGGTGTACGTCAACGCACTTGGGGTAAATGGGTAGCCGAGATCCGCGAACCCAATCGCGGAGCTCGGCTTTGGCTCGGCACCTTTGATAATTCTTATGACGCTGCTGTTGTCTATGACGCTGCGGCCCTTAAGCTCTATGGTGCCGAAGCCAAACTCAATTTACCCCATCTTTACAACAATCAGGCCCAGGCCCAGGCCCAAATCCAGAACTCTAAGCCCATTACCATCATGAGCCCATCTCTATCTCCCGTTTCAACTACTACTGCTCAAGCTTCTTCTCCGTCTGTAACTTCCGTTTATAATGTGGCTTCGCCGTCAACTTGGAGCGTAGGAAGTGATGATTCATCCTTTTATTTCAACAGCCACGATTTCGGTATCCATAATGATATTCCATCCGCATTTAATTTAATCGACATTAATAAAACAGCTGATGATTATTCCGTTGATAATGCTACTAACAATAATCAAAGCAGTGAAGTACTTGGTGGAGAAATGTTTAGAGATCTAAACATGAATTTACCAGAAATTGATGATTCATCAATTTGGGAAGAAGCTAAGGCAACAACTTCATTTCAAGAAGCAGTGAATGATCCAGGAATAGGTGGATACAACTTGGATGATGACCTTAATTTTCCTCCATGGTGTGGTTAA

>Solyc05g009250.1.1 (SlERF5-1)

ATGAAATCCAAAATCAAACAAGATTGTATTGATGAAAATGAAGTAATTGAAATTGAGAGGACAATAAGAGTGAAAATTTCAGACCCAGATGCTACAGAATCATCTTCAGATGATGAAAAACAGGAAAAAAGACCGAAGATTATTGTTCATGAGATTGTTCAGAAAAAGGTAAAGATTCAATCTTTTTTACTTAATAGCAAAAACCCATTAGATTTTTATCAATTGCCTCCTCCTAGGGTTCGAAAAAGAAAATATCAGAAGAAGGGTGTTTCTGCAAAATCTGGAAATCCGCCATTAATGGCGGATTCTCAGAATTTGGATTTAACGAAGGTAAAGACTCGTTTTTCACTAAAAAACAGAAGCCCTTTGAGTTTTGAACAATCTTCTGGTAAATTACCTCCTATGGTTCGAAAGAGAAAATCTGGAAAATTTGCTACTGAAATTAGAGACCCATTTAGCAAGAAAAGGATTTGGTTGGGTACTTTTAATACTCCTGAAGAAGCTTCTGAGGTTTATCAATCTAAGAAACTTGAGTTTCAGGAGAAGTTGGAAAAGGCTAGAAATGCAAATGTGGATAAGGTTATTTCTGCAAAATTTGAACTTGGGTCTTCTTCTTCGAGTGCTCCGCCATTAATGGCTGATTATCAAAACACAGATTCATCTAATGAGTCAGTTGATAGATTGAAGAAGGCTAAAAATGCAAAAGAGAACATGGTTATTTCTGCAAAATTTGAACTTGGTTCTTCTTCATCGAGTGATCCGTCATTAATTGCTGATTCTCAAAACACAGATTCATCAAATGAGTCAGGTGATATATTGAAGAAGTCCAAAAATGCAAAAGAAAAAATGGTTATTTTTGCAAACTCTGAACCTGGTTCTTCTCCTAGTGAGCCAATATTAATGGTGGATGAGATTGATGAGCAATTGAATAAGGCTATAAATGCAAATGTGGCAAAGGGTATTTCTTCAAAATCTGAACTTGGTTCTTCTTCTAGTGATCAAGTTGATGCACAAACCTCGGATTCATCGAATGGGGTTGAAGAAAGTGATGAAGATTTGTGGATGGGGCAATGGATACAAATTTCAGGCTTGATGGATGATGTCTCGGGGTCATTTGTCGGGACTTGA

>Solyc05g009450.1.1 (SlERF5-2)

ATGAAAATGTCCGAGTCTCGGAGACAATCTCAGAAATCGAAGATTAGGGAAGAATCAGCCCGACCCATGAGGAAAATCCGGATCGTTTGTGACGACCCGGAAGCTACTGATTCATCTGATGATGAAGGGGTTGATGTATCGAAACCCAAACGATTTGTTAGGGAGATTTATCTTCCTGTTGTTAGCTCTTTTACCCTGAAAAAAGTACCTGAAACTGAAAGTTCTTGTCAAGATAGCAACAATGGTGACAAAAAGAGAGCTAAAACCCCTAAAACCCCGAGTGGCCCTAGGCCCTCGTCTTCGAAATACCGAGGTGTTCGTCAGCGTAAATGGGGTAAATGGGCTGCTGAAATTAGGGATCCTTTTAAATCTAGGAGGGTCTGGCTTGGTACTTATAATACTGCTGAAGAAGCTTCTCATGCTTATGAAATGAAAAGGCTTGAATTCGAAGCGATGGCTAAGAGTAATTCGAATACTGATGTGTCTGAGAAGAGCTCGAATAACGAATGCAACAACAAGAATCATCGAAAGGTCAACAATGCTGGTTGTGTATCAGAGGAGGATGACTCTGCTCAGAGTTTGGTCTCACATACTTCACACACATCTCCAGCATCAGTTTTTGAAATGGATTCATTGACTTCTGGTTCTGCTGCAGTGTCTGAGGTTAACAACAACGACAAACTCGTCGTGCAGCTAGTGGATGTTGATAAGATGGGATTGATGGAGGATAGTTTATCGCTTGCTGACATTGGTGCACGAATGGATTTCGATACGGAGATGGACATGTTCTTTGCTGGGAATGATAATTTTGATCAGAATCTTGATGACTTTGCTGTGAATGATTTCGAGGATCTTCCAATATGTGGACTTGATGAGCAGTTGCCTGCTGGTTTACCTGATTTTGATTTCGACTTTGATTTTGATGGATACAATGAAGCTTGTGCTTGGATGGATGATGTAGCAGCTACTCCATTGATGAATGGAACAACAACAACAACACCCCTCAATATTGCATTATGCCCATAA

>Solyc05g013540.1.1 (SlERF5-3)

ATGGAAGATGCAATGAGAAGACTTAATCAAGAAACTGATGTTCCACTCCAAAACACTACAAACTCTTCCACTGTTAATAAAACTAGATCTTCTTGTTCATCAAACAAAAGATCACTCAAAGATACTGCTACTGGTCCCTCCGCGGTGCGATACCGCGGAGTTCGTCGTCGTCCTTGGGGACGTTATGCAGCTGAAATTCGTGACCCTCAATCAAAAGAAAGAAGGTGGCTCGGTACTTTTGATACCGCTGAAGAAGCTGCTTGTGCTTATGATTGCGCTGCTAGAGCAATGCGCGGTGTTAAAGCACGTACTAATTTCGTCTACCCACCTTGTCCTACTCCTACCCAACCCACTTCTACCAATGACGCTTTGTTTAATATTCATTCGTCTTATAAATTAACATCGCCTTACTATCATCAGAGTTCTAATACTCTTAAGGATTTGTCTAATAGACCCTTTTTTCATTCCTCCAGTCCTTATGGTTCTACGGGGCGTGCTCATGTGATTGGTCAGAAAAGCAATGATTCTTTGAATATGCTCCTGTTTCGTGAACTCCTGAGTTCAAATTCCAGCAACAATACTAATAATTTGAACGTGACTAGTAGTATGAACATGCCTAATTTGTACGAACAGCTGCCGAATTTCACGATGAATCGTAATACCAACAGCTTCGGTTCGTATTTGCCTAATAGCAGCAACCCCGTGATTCCCAGTTCTTCTGTCATGACTACTCAGGTCCCCAAATTCGATAACACTGTCCACTGTACTATCAACAACAATAACAGTAGTAGTGGTGCTACCGCAAATGATGATTCTGCTGCAGGGATGGATTTTTTCCCATCTGAGTCATCTGATTCTGGATTATTAGAAGAGGCTTTAAATGGTTTTTTCCCAAAGCCCAAGCCCATTAAATCTGTACCATCATCATTACCTAATTACGAATTTTGCAATATTTTCTCTCAACAACCGCAACAACAGGAGCAGATTAATAATGGTGGTTTAAACAGTGATTTTGGATTATTATCATCGTCATTGTCATCATTTCCAGTGGATTATTTTCCAGGAAATCTTCAGGTAGCACCAGGAGACAACATAATGGGAGACATATTTCAGTATCCAGATCTTCTCAGTATTTTTGCAGCCAAGCTGCAAAATGCTTAA

>Solyc05g050790.3.1 (SlERF5-4)

ATGCATCAAGAAAATTCAACACAATTTGATCTAAGCATTCTTGATTCACCACTAGAACATCAATTCAACGGCTTCAACTTGGAATCAATCTCCTTCGATCGATCCATCGATCATACGTGGGAGAAAATAGACAAAAATGAAGATAATATTCCTCGTTCATCATCACAAGTATCGAATAACTGGAAGAGATATAGAGGCGTACGTAGGCGTCCGTGGGGAAAATTCGCCGCCGAAATTAGAGATCCGAAAAAAAAAGGTGCTAGGCTTTGGCTCGGAACTTACGTGACCCCCGAGGACGCGGCATTGGCTTATGATCGAACCGCGTTTAAATTGAAGGGCACACGCGCTTTATTGAATTTTCCTCATTTGATTGACACGAATGTTGCTGAGCTGAATAGAGTTCGGCCTAAGCAACGTTCGCGATTATCCGAAATAATTAAGGGGGAAGAAATTCCGAGTCCAAAAAGAAGAAATCCTCAGCTAATAAACAACATAGCTAAAGTCAATAGTATTATACATATGTTTGAAATCACTAGTTTGGCATTTCATCCTCAAGGTGAAAATAAATAA

>Solyc05g050830.3.1 (SlERF5-5_DREB)

ATGAAAAATCAAGAAGAAATAATCAACCTCAAAAATCGTCATGATTTCATTTCTAATGGCTTGAGTTTTCCTTCAAATATCGCGACATCACCATCATCATCATCTTCTTCTTCTTCTTTTTCCTCGAATAATGCATTAGTTTCAAAAAAGTGTCAGAGCTCGAAGAAAATTGAAGAAGAGAAAAAGAAAAAGAAGATAAATAATGATGACGAAGATAAGCACCCAACCTATAGAGGGGTACGTAAGAGGAGTTGGGGCAAATGGGTGTCCGAAATTCGCGAACCAAAAAAGAAATCAAGAATATGGCTAGGTACTTATCCAACGGCTCAAATGGCAGCTCGAGCTCATGACGTGGCAGCTTTAGCCATCAAAGGATGTTCGGCTCACCTTAATTTTCCTCATTTGGTTGATCAATACCCACATCCGGCTTCCACTTGTCACAAGGATATCCAAGCAGCAGCCGCAAAGGCTGCTGCCATCCCATTTCCCGAGGAAGACGAGGAAGAGGAAGATCAAATTGAGTCGGATCAAGTTGAATTACGAAATTGCCATCCATCAACAAATTTATTCTTGGAAAATGCCAAAGAATCACTAAATTCTCCATCAAGAGAGGATGATGACACATTTTTTGACTTGCCTGATCTTTCTATTGATGTGGTGGATCAAACTAATAGTTATTGGTGCACAATGTCCACGTGGCAGCAGCTAATTGGAGCTGACACTATGGTATACCGGCTTGATGAGCCATTCCTATGGGAATAG

>Solyc05g051180.3.1 (SlERF5-6)

ATGATTTTGAAGATTCAAGACGAGAAACAAAGTTCAGATTCAGTAGAATCATCAATATCACTCGAAGACACTTCCTCAAGTGATACTTATTCTTTCAATAAGGAAAATGGCTTTCATTCAGAACGACCTAAGAAGGAGATAAAACACTATATAGGTGTTAGAGCAAGGCCATGGGGAAAATTTGCGGCTGAAATTAGGGATTCAACTCGGAATGGAATTAGGGTTTGGCTTGGAACATTCAATAGTGCTGAAGAAGCCGCGTTAGCTTATGATCAAGTTGCATTTTTAATGAGAGGTCCAACAACTTGTCTAAATTTTCCAGTTGAAAGAGTTAGCAAGATGTTGGAAGAAACAGAAATTTGCAATTTCTTCAAGAATGGATTGTCACCAGCAGCAGCCTTAAAAGAGAAACACAAAAGGAGAAGTAGTAGTAATATTTCAAGAAAAAAGAAACAAAAGGTAAATCATGAAGAAGAAATTAACAATAACAATAATAATAATAATAATAATGTTGATAATAATAATAATAATAATAATAATAATAATAATAATAATAATAATGTTTTTACATTTGAAGATTTGGGAAGTGAACTATTGGATGAACTCTTGTCTGAGTATTCTAATTCAAATTAG

>Solyc05g051200.1.1 (SlERF5-7)

ATGTCAAGCCCACTAGAGATAGATACTTCATTTTCACATTCCAATTTGTTGTTTTTGGAAGATGAATCATCATGGAGTAATACTCATGATCCATTTGTTGATATTGATGAATATCTACCAATAATTATACCATGTAATGATGAAGAAATAGTAGTAGAATCCTCAAACACTAGTACTACAACAACAACAACAACAACATCAAAAGTAGCAAGTATCCAAAATATTCATCATGATCAAGAAGAGGTAACATCCATAGAGAAAAAACATGAAGATGATCAAGAAAAACATTATATTGGAGTTAGAAAGAGGCCATGGGGTAAATATGCATCAGAAATTAGGGATTCAACGCGTAATGGAATTAGGGTTTGGTTAGGAACATTTGATACTGCTGAAGAAGCTGCTTTAGCTTATGATCAAGCCGCATTATCAATGAGGGGTCCTTGGTCTCTACTCAATTTTCCAATGGAACATGTTAAAAAATCTCTTGAAAATATTGAGTATTCTTGTAAAGATGGATTATCTCCAGCTGCTGTTTTAAAAGCTACTCATAAAACTAGAAGAGTCAAGCACAAGAGAAGTAGTAGAAAGAAGAAGAATGAGAATTTGGAAAATGTTTTTGTTTTTCAAGACTTGGGAGTTGAATTATTAGAAGAGCTTTTAATGACTTCATCATAG

>Solyc05g052030.1.1 (SlERF5-8)

ATGACGAAACAAGATGAAGGATTAACATTAGAACTCATACGACAACATCTCCTCGAAGATTTCACAACTACAGAATCATTCATCGACAGTCTCAATTCTTGTTTTTCCGATCACATCTCCTCCTCCGATGACATCTCCCCTGTTTTCACTTCAGTAAAAACAGAGCCATCTACATCCAATTCCCTCTCAGATTCACCCAATTCCTCATACCCAAATGAACCCAACTCCCCAATTTCCCGTTACTTCAATCTCCGCTCCGATTTCCCTGAATTCAAAATCGATTCAGATACCATCCTCAGTCCAGTTTTCGACAGCTCCGCCGGTTCTAATGAAGACAATAATAAGAAGAAGAATTACAGAGGGGTAAGGAGAAGGCCATGGGGGAAATTTGCGGCGGAGATAAGAGATCCAAGTCGAAAAGGATCGAGGATTTGGTTGGGTACTTTTGATACTGATATTGATGCTGCTAGAGCTTATGATTGTGCAGCGTTTAAGATGAGAGGAAGAAAAGCTATTCTGAATTTTCCGTTGGATGCCGGAAAATCTGGTGCTCCGGCGAATGTTGGCCGGAAAAGGAGAAGAGAGAACAAGATGGAGTTGGTGTAG

>Solyc05g052040.1.1 (SlERF5-9)

ATGGATAAGCCCCACAAGTGGCTTCCTTGTTCTAACCAAGAAGGCCCTAAGAAACCTCCATTGAAATTAAAATTTTCCACCCTTTATAATCTCCATTTTCACTCTTCATTTTCCACTCAATTCAATTACCAACAATATTTTTCAAGAAAAAAAATGGATTCTTCTTCACTAGAAATGATAAGACAACATCTTCTTGATGATGTTGTTTTCATGGAAACTTGTTCTTCTTCTTCTTCTTCTTCATTAGAAACAACAAGTAGTACACTTTATTCTCAAACCTCATCGAATTCGGAATCTTTAGAATCATTAACCTCTGAGATCAAACTTGAAAGCAATTTCTCTGTTTATCCTGATTTCATCAATACACCTCAAAGTTCAAATCTTGAATCTGTCTCTCGTTTTTTCGATAACTCAACTATTGAATTCCAAGCTAAACCCCAAAAGAAAAGAAGTTTCAATGATCGAAAACCTTCGTTAAACATTTCGATTCCTTCTGTTAAGAAAACAGAGGAACCAAAAACAGGGGAAGTAAAAACAGGGGAACCAAAAACAGAGGAGCCAAAAACAGGGGAAGTGAAAACAGAGTACTCTGTTAAGGAGAAAATGGTAGAAAATTCGGAGAAAAAGCGATACAGAGGAGTGAGACAAAGGCCATGGGGGAAATTTGCAGCGGAGATTCGTGACCCAACTAGAAAGGGGACACGAGTTTGGTTAGGAACATTCGATACTGCAATGGATGCAGCCATGGCATATGACAGAGCAGCATTTAGGCTCAGAGGGAGTAAAGCAATCTTGAATTTTCCACTTGAGGTAAGCAATTTCAAGCAAGAAAATCATGAGATTGAGAAAAATGTTGTGAATTTGAATTCGAATACGAATTCTTGTGGGAAAAGGGTGAGAGGGGAAATGGAGAATGATGACGGAATTGTAATGAAGAAAGAGGTGAAAAGAGAACAAATGGTGGCAACTCCATTAACACCTTCAAATTGGTCTTCAATTTGGGATTGTGGAAATGGAAAAGGTATTTTTGAAGTGCCACCTTTGTCACCATTATCACCACATTCAAATTTTGGTTATTCTCAACTTTTGGTATCATAG

>Solyc05g052050.1.1 (SlERF5-10)

ATGGATCAACAGTTACCACCGACGAACTTCCCGGTAGATTTTCCGGTGTATCGCCGGAATTCAAGCTTCAGTCGTCTAATTCCCTGTTTAACTGAAAAATGGGGAGATTTACCACTAAAAGTCGATGATTCCGAAGATATGGTAATTTACGGTCTATTAAAAGACGCTCTAAGCGTCGGATGGTCGCCGTTTAATTTCACCGCCGGCGAAGTAAAATCGGAGCCGAGAGAAGAAATTGAATCGTCGCTTGAATTTTCACCTTCTCCGGCGGAGACCACGGCAGCTCCGGCGGCTGAAACACCGAAAGGAAGACATTATAGAGGCGTTAGACAGCGTCCGTGGGGGAAATTTGCGGCGGAGATTAGAGATCCGGCGAAGAACGGAGCTAGGGTTTGGCTTGGAACGTACGAAACAGCTGAAGAAGCTGCAATTGCTTATGATAAAGCTGCTTATAGAATGAGAGGATCAAAAGCACATTTGAATTTCCCGCACCGGATCGGTTTGAATGAACCGGAACCGGTTCGAGTTACGGCGAAAAGGCGAGCATCGCCGGAACCGGCAAGCTCGTCGGGAAACGGTTCCATGAAACGGAGAAGAAAAGCCGTTCAGAAATGTGATGGAGAAATGGCGAGTAGATCAAGTGTCATGCAAGTTGAATGTCAAATTGAACAATTGACAGGTGTCCATCAACTATTGGTCATTTAA

>Solyc05g052410.3.1 (SlERF5-11_DREB)

ATGATGTTACCTATGGATTATACAAGGAAGAAGAAATCAAGGAGTAGAAAAGATGCACCAAAGAATGTAGCGGAGACACTTGCGAAGTGGAAAGAGGTGAATGAGAAATTAGACGCGTGTGATGATGATGGAAGGAAGCCAGTGCGTAAAGTTCCTGCTAAAGGATCAAAGAAGGGATGTATGAAGGGTAAAGGAGGACCGGATAACGGGCGGTGCAAGTATAGAGGTGTCAGGCAAAGGACATGGGGTAAATGGGTTGCTGAGATAAGGGAGCCACATAGAGGGAGAAGGCTTTGGTTGGGTACGTTTGATACGGCAATTGAGGCTGCTCTTGCTTATGATGAAGCTGCAAGGGCGATGTATGGTCCTTGTGCAAGGCTGAATCTCCCGGACTATTACGCCTCGTCGAAAGAGTCTTCCAAGGATGATTCTTCGTTGCCTACTGTATCTCGTTCCGACTCAAATACAGCATCAAGTTTCTCTGAGGTGTGCCCTGCTGGTGACATGATGAGAGGAAGAGCCAATGTTCCTGCGGCAAGACATGAAGATAGATCAATAGAAATTGACGGTGCTAGGACTGGTTCTAATGAAATTGGAACGCCTTTGAGCTCATTGAGAGAGGAAGCGGAAGACGAGACCAAGGAGGTGTCAGATAAAAGTGAGACTTTTACACCTCTGAGCTCGTTGAGAGAGCAAGCGGAAGATGAGGCTAAGCAGGTGTTAGATAAAAGTGAGACCTTTGAAATTAAAGATGAGCCTGCGGCTTGTAGTTATGATTCATGGGATATCGGACAAGAGGACCTAGGTAACTTTTGTTTGGATGATGAGATGTTTGATGTAAATGAGCTGTTAGGCATGATGGACTCGACTCCAGTAGATGCCTCAGCTCCAAGTCAGGATGTTGGTTTTGTTCCTCCTAAGCAAGAGCAGTATGCCTATGATCCTTCATATCAATTGCATTCTGCTGCCTACGATGCCAATCAGTTGTCAAATCCAGCATACCAGTTGGACAATGCCGATGACCAGTTCTCAAATCCATTATATCAGTTGGATAATGCAGGTGTTGATACATTGGAAGGTCTGCAGCAAATGGAGCAACAATCACCTATCGAGGTTGATTATGATTTTGATTTTCTGAGACCAGGCCGGCAAGAGGACTTCCACTTCTGCTTGGATGAGTTGGATGTCTTGGATTTCTAA

>Solyc06g035700.1.1 (SlERF6-1_DREB)

ATGGCTGCTTATCATTTTAATGACAATTCTCCTTCATTAGAAAATCTTTCACCCACTGGGGGAGGAAGTAGCACTAGGCATCCCAATTTCAGAGGAATTCGACAGAGGAATGGGAAATGGGTCTCCGAAATTCGCGAGCCTCGAAAGACCACACGTATATGGTTAGGTACTTTTCCCATTCCTGAAATGGCAGCTGTCGCTTACGATGTTGCAGCTTTGGCATTAAAAGGTCCTGATGCGCAATTAAACTTTCCTGATCGTGCATACTCCTACCCTGTCCCTGCTTCTCTGTCAGCCGCAGATATTCGTACTGCGGCTGCTAATGCTGCTGCTGCTAGAGCACCTCCTTTGTCAGAAATCAATACAGCAGCAGGAGGAGGACAAGGGCAAGAGTTTGTGGACGAAGAGGAAATATTTGGAATGCCGAAATTGCTTGATGATATGGCAGAGGCAATGCTTGTTAGCCCGCCAAGGATGCATCAGTACGACGAATCACCTGAAAACTCTGATGCAGATAGTCTCTGGGGTTATCCATGA

>Solyc06g050520.3.1 (SlERF6-2_DREB)

ATGGCTATTATGGATGAAGCTGCTAATATGGTTTGTGTGCCGTTGGATTATAGTAGAAAGAGGAAATCAAGGAGTAGAAGGGACAGAACAAAAAATGTGGAAGAGACACTAGCTAAATGGAAGGAGTATAATGAGAAACTAGACAATGAAGGGAAAGGGAAGCCAGTGCGTAAAGTTCCTGCTAAAGGTTCAAAGAAGGGGTGTATGAGAGGTAAAGGGGGACCAGAAAATTGGCGGTGTAAATACAGAGGTGTTAGACAGAGGATATGGGGTAAATGGGTTGCTGAGATTAGGGAACCTAAAAGAGGTAGTAGGTTATGGTTGGGTACATTTGGTACAGCAATTGAAGCTGCTTTAGCATATGATGATGCTGCAAGAGCTATGTATGGTCCTTGTGCAAGGCTTAATTTGCCAAATTACGCGTGTGATTCTGTTTCCTGGGCAACTACATCTGCATCTGCATCTGCATCTGATTGCACCGTTGCTTCTGGTTTCGGCGAGGTATGTCCGGTTGATGGTGCTCTTCATGAAGCTGACACACCATTGAGCTCAGTGAAAGACGAAGGGACCGCGATGGATATTGTTGAACCTACGAGTATTGATGAAGATACGCTTAAGTCTGGATGGGATTGTCTAGATAAATTAAATATGGATGAGATGTTTGATGTAGATGAGCTATTGGCTATGTTAGATTCTACTCCAGTTTTCACCAAGGACTACAATTCAGATGGAAAGCACAACAATATGGTATCAGATTCGCAATGTCAGGAGCCGAATGCAGTGGTAGATCCTATGACTGTTGACTATGGCTTTGATTTTCTGAAACCAGGCAGGCAAGAAGATCTTAATTTCAGTTCGGATGACCTTGCATTCATAGACTTGGATTCTGAACTTGTCGTTTGA

>Solyc06g051840.1.1 (SlERF6-3)

ATGGATCATCACAGTTCGCTTTGTCCGATCAAATATACCGAACACAAGAGAACAATCAGAAAAGTTACTAAGCCTTCCGTTATTAAGCCTAAGAAGGTATCAGATGTTCGGAAATCCTCGGAGTATAATCCGAGGACTGTACGGATATGTGTTACTGATCCAGATGCAACTGACTCTTCAAGTGATGAAGATGAGCTTTTTGGGCGTAAGCGTGTGAAAAGATACATTAGTGAAATCAGTATTGAGAGTCCATCAGTTAATGATGTGAAAACTTTGAGTAGTGGTAATGGGAAGAAGCGAGTAGCTGAAGGTTCGCAGGCGAAGCAGAAGGCGTTGAAAGGGAAGGAAGTGGCTGATAAAACGGTTCGGAAGTTCAGGGGAGTGCGTCAACGGCCGTGGGGTAAATGGGCTGCTGAAATCAGAGACCCAGCGCGTCGCGTTAGGCTCTGGTTAGGTACTTATGATACTGCTGAAGAAGCTGCTATGGTTTATGATAATGCAGCTATTAAGCTCCGTGGACCAGATGCTTTAACTAATTTCATCACTCCTCCAATAAAAGAGAAGCCGGAAGTGAATGTGGCTTCGAATTCGGGGTACGAATCTGGTGATGAATCACACAATCTTTCTTCTCCTACCTCTGTTTTGCGGTTCAGAAGTAGCGAATCCAGTGAAGAAGCAGAACCCGGTCTCGAAGACATAAAAGAAAATTGCACAGTATTGGTGGAAGAAGAACCAAACAGTGAGCACCTTGAGTGCCAAGGCGAGACACTTACAGTCATACCAGACTATTCAAATGACTACTTACCCACAGACGTTTCCTTCTTAGACGATTATTTCAACTTTGCAGATGCAGAACAGTCACTGTTTAATGACACAACAAGTTTCACCAATGATGATTTGTTTTCGTCTTGGGATTTCACAAATGACTCTGTGTTAGACCCAGAGATCTGCAAATTTGATGATTCATTTCTAGACTTGGGTGCATTAGAAGTGGATAATTATTTCAAAGACATTGGAGATTTTTCCAGTGTTGATGTACTAATGGCGTTATAG

>Solyc06g053240.3.1 (SlERF6-4_DREB)

ATGGTACAATCAAAGAAGTTCAAAGGTGTCAGACAACGCCAGTGGGGCTCTTGGGTTTCTGAAATTCGCCATCCGCTGCTGAAGAAAAGGATATGGTTAGGAACATTTGAGACAGCAGAGGAAGCAGCAAGAGCGTATGATGAAGCAGCTATCTTGATGAGTGGACAAGTTGCGAAAACAAACTTCCCAATTGTGAAGGAGAGTACTGATAACAACAATGACACTACTAAATTCCCTTTAACTTCTTCATCAACACTGTCTAGTAGCATGCTGAATGCAAAGCTGAGGAAATGTTGCAAGGATCCAGCACCTTCCATGACCTGTCTGAGGCTCGATAACGACAACTGTCATATTGGAGTGTGGCAAAAAACATCAGGGAAACATTCAAGTTCTAATTGGATAACAAAAATCGAACTTGGAAAGAAGGAGGAGAAGCCTCATGATCATCAGGATATGAATGCTGAACTTGGCATTACTAAACCATTGGACGAAGAAAACAGAATTGCTATGCAAATGGTGGAAGAACTACTCAATTGGAATTCTCCGTTTTCTGATGAACCGATTACTGATCATCTCTCTCCGTCTTTTTCCAACTCTATATGA

>Solyc06g054630.3.1 (SlERF6-5_DREB)

ATGGAAAACAATTCTACTCATGATCAGCCAAAGTTCAAAGGAGTTCGGTTGAGGAAATGGGGGAAATGGGTTTCTGAAGTCAGATTGCCTAACAGCCGCGACAGGATTTGGTTGGGCTCCTATGATTCTGCCGAAAAAGCCGCAAGGGCTTTTGACGCTGCACAGTTCTGCCTCCGCGGGCCCAAAGCCAAATTTAACTTTCCTGATAGTCCGCCGGATATATCCGGAGGACAGAGGCTTTCGCCCGCGGAGATACAGGCTGTAGCAGCTAGATTTGCGAATGATTACTCGCCGTCAGTGGTACAGGAGATACGCCGTGATGATCATCATCATGATCACGAGGGCAATATTGGAAATATTAATTCACACGTGATAAATATGGAGAAAGATGAGATTTCACTGTCAACAACTAGTTGTGATAGGGTGGTCCAAATGGGCACTAGTAATACTGTTGCTGAAATGGATTGGGCTTTTCATAATGATATGATGGAAAACTATTCATACAATGCAAGTGGGCCTCCACCTGAATATTTTTGTGATCCATATTATATCGTCGGAGGAGGAGGAGGAGCAGGAGTACTCGATAATTTATCGAGTAATCTCTACTCACCACCACATTTTCCGCAAAGAACAACTCCTAGTTATGACGATGATGATACTGGCAATGGTGACGATGAACATTATTCTCAACAATCCTTCCTATGGAACTTTTAA

>Solyc06g063070.3.1 (SlERF6-6)

ATGTGTGGTGGTGCAATTATCTCCGATTTGGTACCTCCTAGCCGGATTTCTCGCCGGTTAACCGCTGATTTTCTATGGGGTACATCCGATCTGAACAAGAAGAAGAAGAACCCTAGTAATTACCACTCAAAGCCCTTGAGGTCTAAGTTTATTGACCTTGAAGATGAATTTGAAGCTGACTTTCAGCACTTCAAGGATAATTCTGATGATGATGATGATGTGAAGGCATTTGGCCCCAAATCCGTGAGATCTGGTGATTCAAACTGCGAAGCTGACAGATCCTCCAAGAGAAAGAGGAAGAATCAGTACCGGGGGATCAGACAGCGTCCTTGGGGTAAGTGGGCAGCTGAAATACGTGATCCAAGGAAAGGTATTCGAGTCTGGCTTGGTACTTTCAATTCAGCCGAAGAGGCAGCCAGAGCTTATGATGCTGAGGCGCGAAGGATCAGAGGCAAGAAAGCTAAGGTGAACTTTCCTGATGAAGCTCCAGTGTCTGTTTCAAGACGTGCTATTAAGCAAAATCCCCAAAAGGCACTTCGTGAGGAAACCCTGAACACAGTTCAGCCCAACATGACTTATATTAGTAACTTGGATGGTGGATCTGATGATTCGTTCAGTTTTTTCGAAGAGAAACCAGCAACCAAGCAGTACGGCTTCGAGAATGTGTCTTTTACTGCTGTAGATATGGGACTGGGCTCAGTTTCCCCTTCAGCTGGTACAAATGTTTACTTCAGCTCTGATGAAGCAAGTAACACTTTTGACTGCTCTGATTTCGGTTGGGCTGAACCGTGTGCAAGGACTCCAGAGATCTCATCTGTTCTGTCGGAAGTTCTGGAAACCAATGAGACTCATTTTGATGATGATTCCAGACCAGAGAAAAAACTGAAGTCCTGTTCCAGCACTTCATTGACAGTTGACGGTAACACTGTGAACACGCTATCTGAAGAGCTATCGGCTTTTGAATCCCAGATGAAGTTCTTGCAGATCCCATATCTCGAGGGAAATTGGGATGCATCGGTTGATGCCTTCCTCAATACAAGTGCAATTCAGGATGGTGGAAACGCCATGGACCTTTGGTCCTTCGATGATGTACCTTCTTTAATGGGAGGTGCCTACTAA

>Solyc06g065820.3.1 (SlERF6-7_DREB)

ATGGCTAGGGCACAACAAAGATATCGAGGAGTTCGACAGAGACATTGGGGTTCTTGGGTCTCCGAAATTCGCCATCCATTGTTGAAGACAAGAATTTGGTTAGGCACTTTTGAGACAGCAGAAGATGCAGCAAGAGCATATGATGAAGCAGCAAGGCTAATGTGTGGTCCAAGAGCTAGAACTAATTTCCCATACAACCCAAACATGCCACAAACATCTTCCTCTAAGCTACTCTCAACTACATTAACAGCCAAGTTACACAAATGCTACATGGCTTCACTTCAAATGACCAAAACCTCACCACAAGGACAAAAATTAGCAAAAAATGCAACCAATGTTCAAGAAAGTGTTATTAATTCC

TATAAAATGAAACAACAAATGTTGGTACCAAAGCCATCAGTACTATTGACTCATCATGATCATCATGAGGAAGCTAAAGTAGTCAACTTGGGAGTGGGAGTAATTAGGAAAGTTGAAGATCAAGTACTTGAGGGTATACCACAATTTGTCAAGCCACTTGAAGATGATCACATTGAACAAATGATTGAAGAATTGTTGGATTATGGATCCATTGAGCTTTGCTCTAATGTTGTTCCTTCTCACCAAATCCAGTGA

>Solyc06g066540.1.1 (SlERF6-8_DREB)

ATGTCAAAGCGAATAAGAGAGAGTGATGAAAAAGGTAATAAACACCCAATTTATAGAGGAGTTCGTATGCGAAGTTGGGGAAAATGGGTGTCTGAAATTCGCGAACCGCGTAAAAAGTCACGCATTTGGCTCGGGACTTATCCAACTGCAGAAATGGCGGCTAGAGCACACGACGTCGCTGCATTGAGCATAAAAAAGGACTCATCGATTTTAAATTTCCCTCACCTAATTGACTCATTGCCTCGCCCAATTTCACTCTCACCGAGAGATGTTCAAGCTGCTGCAGCTGAAGCAGCTGCAATGGAGGATCTAAATTATGTCTCTTCTACATCCTCTGTTAGTTCAATTGACAAAATCACGTCGGCGTCGGAGGAACTGGGTGAAATTATAGAGCTTCCAAGCTTGGACGGAAGTTTTGAGTCGGAAGAATCGAAGACCGAATTGAAGATCAGTGATTCAGTGGACGGGTGGCTGTACCCACCGTGGTGGGCATCAGATGGCGACTTTGATGGGTATCTTTTTGAGCAAGATGCTGTTGGAAACAGTTTAATTCTTAGCAACTTTGAAATGATGAAATAA

>Solyc06g068360.3.1 (SlERF6-9_DREB)

ATGGCTCGTCCACAACAACGATTTCGTGGTGTCCGTCAGCGACATTGGGGTTCCTGGGTTTCCGAAATTCGACATCCTTTACTGAAGACGAGAATATGGCTAGGTACATTTGAGACAGCAGAGGATGCAGCGCGAGCATATGATGAAGCTGCAAGGCTCATGTGTGGACCAAGAGCACGGACGAATTTCGCATTTAACGATACTGATTCTCATTCATCATCATCGACCAAGTATCTATCTGCTGCATTGATAGCTAAACTACAACGATGCCAGATGAAATCTCTTAATATGGTGAATAATCGACCTGGAACGATGAAATTAGAAGATCAAAATGACCGTTTATCGTCATGTGGAAACAGGGGAGATCATGGCATTACTAGAAGGACTGTGCAAATGAGTGTTGAAATGCCGGTAAAATATGAAAGCCAAACAGAGAATAATAATACGCAAGAATTCAAGTCACTTGAAGATCATCAAATTGAACAGATGATTGAAGAGTTGTTGGATTATGGATCAATTGAACTCTCTTCTGTTTTGCAAGAATAA

>Solyc06g068830.2.1 (SlERF6-10)

ATGGACCGAATTAGACGTGGAAAGAGGCGCTATGAATCCGAGGAAAAAGAAGATAGAAATTACAATCACATGTATTCATCAGCTCGATCTCAACATGATATGTCTACTATGGTTGCAGTCCTCTCTCAAGTTATTGGCAACAAAAGTACTACTAACACGAATTCTTCTTCTTCTTCCTCCGCACATCATAAGCCATTATTAACCCTAAATCATCAGTCTAATACTACTGCAGCTATGCAAAACCAATTACCTCAGCTCAATCAACAACAAGGGAATAATGAGAAGAGAAGAAGACAGTACAGAGGTGTGAGGCAAAGACCATGGGGTAAATGGGCGGCCGAAATCCGTGACCCGGAAAAAGCTGCTCGTGTATGGTTGGGTACTTTTCACACTGCGGAAGATGCTGCAATTGCATATGATGAAGCTGCACTTAAATTTAAAGGAAACAAAGCTAAACTCAACTTTCCAGAACGGGTTCAATCCACAACGGATCAATTTGGGATATCATACCTAATTACTAATACGAATCATCAACAACATCAATTTCAGCCCACTAATTTTCTCCCAAATTCTGATCAATTACAACAACATCACTACAGTAATCATAATGCTGATGATTTGAAATTTGGCGTCTCGCCGAGTTTTTATCACCCTACAGGGTTCAATCCTAAAGCACTCGATTTAGTGGAACCTTCGAAATCATCATCGATGACATATTTGGTACAACAAGCATCGTCTCATCAGGTACAAGAAGAACCAAGGTATATTAATCACCAGCAAGAAGATGAAAACAATCTGAAATTTTCATCTTATTTTGGCACTTATTCAAGCTCAGGACCAACTTTGGGGGAGTTTGAAGATCAAAAATAA

>Solyc06g082590.1.1 (SlERF6-11)

ATGACGGAAAATTCAGTTCCGGTGATTAAATTCACTCAACACATAGTAACTACAAACAAGCATGTTTTTTCTGAGCATAACGAAAAATCCAATTCAGAGTTACAAAGAGTTGTGAGGATTATACTTACAGATGCCGATGCTACAGATTCTTCCGATGATGAAGGCCGGAATACTGTACGGAGAGTGAAGAGGCACGTGACGGAGATCAACCTTATGCCGTCAACCAAATCGATCGGCGACAGAAAACGAAGATCGGTGTCTCCGGATTCTGACGTCACTCGTCGGAAAAAGTTTAGAGGCGTTCGTCAAAGACCGTGGGGTCGTTGGGCTGCAGAGATTCGGGACCCGACCCGGGGAAAACGGGTGTGGTTGGGTACTTATGACACCCCAGAAGAAGCAGCTGTCGTTTACGATAAAGCTGCAGTTAAGCTCAAAGGTCCTGACGCCGTTACCAATTTTCCGGTATCAACAACGGCGGAGGTAACGGTGACGGTTACGGAAACCGAAACCGAGTCTGTTGCCGACGGTGGAGATAAAAGCGAAAACGATGTCGCTTTGTCACCCACCTCAGTTCTCTGTGACAATGATTTTGCGCCGTTTGACAATCTAGGGTTCTGCGAAGTGGATGCTTTTGGTTTCGACGTTGATTCACTTTTCCGGCTGCCGGATTTTGCTATGACGGAGAAATACTACGGCGATGAATTCGGCGAATTTGACTTTGACGATTTTGCCCTTGAAGCTCGATAG

>Solyc07g042230.1.1 (SlERF7-1_DREB)

ATGGTGAGGGCTAAAGTAAAGAAAGAGAAAAAAGTGAAAGGGGATCGTTATAAGGGTGTTCGTATGAGAAAATGGGGGAAATGGGTAGCTGAAGTACGGCAACCGAAAAGCCGTGACAGAATATGGTTAGGTTCTTACGATACTGCGGAGGAAGCGGCTAGAGCTTATGATGCAGCGGTGGTTTGTTTACGTGGACCGTCCGCGATGATTAATTTCCCGGACGATCCACCGCTGATTTCATGTGATGCTGATAATTATAAGTTGTCACCTTCCGAAATTCAAGTGAAGGCATCCAGACATGCACGTAGTAGTAGTACTAGAGTAAGTGAAGAGTTGGCTGCAGCTGCTGCTACCGTGGTTGTAGACCACCACCACAGCAGATCAGCAGCTGTGGAGAGTGTGTTTTTTAGAGATGATTTGGAGTTTGGATGTTCTTCTTTAGATCATGGGGAAGTAGTGTTGCATGATGATTTGTTTGATAGTGCTAGAATGTGGTCCTTTTGA

>Solyc07g049490.3.1 (SlERF7-2)

ATGCGCCACCGGAAGTCGTCGGAGCTGAAAAGACCAGGATCTGACCTCCTTCAACAGCCTGACGCCGACCCACCTCGTTATCGAGGTGTTCGTAAACGGCCATGGGGTAGATTCGCAGCAGAGATTAGAGATCCGATTAAAAAGACTCGAGTTTGGCTGGGTACCTTTGACACAGCTGAAGACGCCGCACGCGCTTACGACGATGCTGCACGCGCTCTCCGTGGAGCTAAGGCGAAAACTAATTTCAATATGTTACCTCTAACAGATGATCCTTATGATGATGAGTTTGAGCTTTTCCCCAATCCGAGACCGGCTTCTAGCAGTATGAGCAGTACGTTGGAATCGTCTAGTGGGCCTCGTGGCGGATCGAGTAGTAAGGTGACCCGGATGAAGATTCCTCGCCCAGTTCGTCCGATGGAGGAATGCCGGAGTGATTGCGATTCGTCGTCGTCTGTGGTGGATGATCGGTGTGATGTTGATCAAACGTCATCGTTTGTGACCAAACAACCTCTGCCGTTCGATCTGAATCTGCCACCTCCGTCGGATAACGATGGAGTTGATGTTGATGATTTGCACGTCACCGCTTTATGCCTCTAA

>Solyc07g053740.1.1 (SlERF7-3)

ATGGCTGTGAAAGATAAGGCTGTGAAAGGAGGTAATGTGAAAGTGAATCATGGAGTTAAGGAAGTTCACTACAGAGGTGTAAGGAAGAGGCCATGGGGTCGTTACGCTGCGGAGATTCGTGACCCGGGTAAGAAGAGTCGGGTCTGGCTGGGTACTTTTGATACGGCGGAGGAAGCGGCTAAGGCTTACGATGCCGCTGCCAGAGAGTTTCGTGGACCTAAAGCGAAGACGAATTTCCCCTTTCCGGCGGAGATGAATAATGTTGGTAACAATAACAGTCAGAGCCCGTGTGGGAGCAGTACCGTGGAGTCATCCAGCGGAGAAACGGTTGTTCACGCGCCTAATACGCGACACGCGCCGCTGGAGCTGGATCTCACGCGCCGTCTCGGTGCCGCTGCTGAAGGTGGACGTGGAGGTGTCGGCTACCCGATCTTACACCAGCAGCCGACGGTGGCGGTTCTGCCGAACGGTCAGCCGGTTTTGTTGTTTGATTCTATGTGGAGACCGGGAGTTGTTAGTAGGCCGTATCAGGTTGTACCGGCGACGATGGAGTTTGCCGGTGTCGGTGCCGGAGTTGTTACTAGTGTGTCGGATTCGTCTTCCGTTGTGGAAGAGAAACATTATGGGAAAAAGGGACTTGATCTTGATCTTAACCTTGCGCCACCTATGGAAGTTTAA

>Solyc07g054220.1.1 (SlERF7-4_DREB)

ATGGCGACACCACCAGAGGAACCAATGGAGTTTGATGATAATACTTTTGAAAGACAACAAAGGAGGCCAGTTTTTGAAGAAGCTTCCATGTCTAATAGGCGTTTCAAAAAAATCAAAAGTCCAGAACGTCAATCCTCTGTTCAACAACCGTTTGATCATCGTAATAATCCGACGCCAATGGCGTTTCCTCCACCTCCGTCATCGTCAAGGCTTGTTTTCCCTTTTGCCTTTGACGGTACGCAGCAGTCCATGGAAAGTTCGTCACCATTGGGAGCGAACGCGATGCCTCTGTTTCATCCACAGCAGCAAAATCAGCAGCAAATGATATCTTTTTCGCCTCAACAGTGTCTTTACCCTCCGTATTTTGCAGGGGAGTTAGGGCCATCGCAGAATCAGCAGCAGATGTTGAGGTATTGGAATGAGACGCTTAATTTAAGTCCGAGAGGAAGAATGATGATGATGAGCAGATTGGGGCAGGATAACAGGGGATATTTTAGGCCTCAACAGGTGCAAGTACAGCCAATTTCCGCTACGAAGCTATACAGAGGAGTGAGGCAGAGACATTGGGGGAAATGGGTAGCTGAAATTCGTCTTCCTCGTAATAGGACTCGTCTTTGGCTTGGTACTTTTGATACAGCTGAAGATGCAGCTATGGCTTATGACCGCGAGGCGTATAAGCTAAGAGGAGACAATGCTAAGCTCAATTTCCCCGAACACTTTATTGGTAAAGACAGAGGAGAGACATCTACAGAGGCTAATTCGTCTTCAATTACTACTCATGAATCTTCCTTGCCTGAACACAATTCTGAAAGTTTACAATTACAGACTGTAAATAACGAACAACTGCCTCCGTCACCGCCCCCACAGCCACCACCAGAAGGTGATAACCATGACGAGGACTCGGGGATAGGATCGAGTCAGGTGACTACTAATTCACAATCATCTGAGTTAGTATGGGGTGATATGGCGGAGGCTTGGTTCAATGCAACTGGTTGGGGTCCAGGTAGCCCAGTTTGGGATGATTTAGACACAAATAACAACCTCATGTTTTCACCTAATCTTCATTTTGGGAATTTCAGT

CAACAAGAGCCTCATGATTCTGATCCTCATCAACATCATGACACGAATAGCGATCCATCTTCGCCTTCATGTCCTATGAGGCCGTTCTTTTAG

>Solyc07g064890.1.1 (SlERF7-5)

ATGCGGAGAAGCAGAGCAGCCGCTGCGGCGAGACAAGTTCCGGCGACGGAAGTTCCGGTACCGGCACCGGTGGCCGGAGAACACAACGGATCTGGAGGATCTAAGGAGATAAGGTTCCGTGGAGTTCGAAAGAGACCATGGGGAAGATTTGCAGCAGAGATTAGAGATCCATGGAAGAAAACTAGGGTTTGGTTGGGTACTTTTGATTCTGCTGAAGATGCTGCTCGTGCTTATGATGCAGCAGCTCGTACTCTTCGTGGACCTAAAGCTAAAACTAATTTCCCTTTACCTTCTTCTCATCATCTACCTCCATATCCTCACCATCATCAGTTCAACCAAAGCATCAACCCTAACGATCCCTTTGTCGATTCCCGGTTATACTCTCAGGACCACCCATTAGTTTCACAGAGACCTACTTCAAGCAGCATGAGTAGTACGGTGGAGTCCTTCAGTGGACCACGGCAGCCGCCGCGGCAGCAGACGGCAGCTTCCGTGCCTTCCAGAAAGTATCCCCGGTCACCGCCTGTTGTCCCGGACGATTGCCATAGCGACTGTGACTCATCGTCTTCTGTCGTTGAAGACGGTGAATGTGATAACGACAACATCGCTTCTTCCTCTTTCAGAAAGCCGTTGCCTTTCGATCTAAACTTACCGGCACCGATGGATGACTTCAGCGCCGACGCATATGCCGATGATCTTCACTGCACAGCACTATGTCTTTGA

>Solyc08g007230.3.1 (SlERF8-1)

ATGATATTTGAAAACAGCGAATTTATGAATGATTTCGAACTTCTCGAATCGATTAGGCAACATTTACTTGAAGATTGGGATTCCCCTGTTACAACAGTGCTAAAGCCGTCCGTTAAAATTGAGCCGGAAGTCAGTGTGAGTTCGCCGGAAATGTTCGATTTCACCGGATTTACAGCTCCGGCAGCGGCGGAGTCGGCGGCGGAGGTGAAATTGGAAGTGGAATTGAAAACAGCGGCGGTGAAGAGGACACCGGCGTCGTCGAAATCGATGCATTACAGAGGAGTAAGACAGAGGCCATGGGGAAAATTCGCGGCGGAAATTAGGGATCCGGCGAAGAATGGGGCGAGAGTGTGGCTGGGAACCTACGAGACGGCGGAGGATGCGGCATTGGCTTATGATAAGGCGGCGTATCGAATTAGGGGTTCGCGTGCATTGTTGAATTTTCCGTTGAGGGTTAATTCGGGCGAACCCGAACCAGTTCGGGTTGGTTCGAAGAGATCGTTAACAACGACCTCTTCGGAGAGTTTTTCTTCGTCATCATCAGAAAATGAATCGATGATGACGAAGAAAACAAAGAAAGTTTGCCAACTTTATACTTGA

>Solyc08g007820.1.1 (SlERF8-2_DREB)

ATGAATAACGACTCGAGTTTGTACTCATCAGATATCAACCCATTAAGTGCAACAACACCTTCCAATGAGGAAGGAATACTATTATTAGCATCGAGCCAACCCAAGAAACGTGCAGGGAGGAAGAAGTTCAAGGAAACTCGCCACCCGGTTTATAGGGGAGTGAGGAGGAGGAATAATAACAAGTGGGTTTGTGAGCTACGTGAGCCTAGTCAACAGAAAAGAATATGGCTAGGGACTTACTCTACTCCTGAAATGGCGGCTCGAGCTCATGATGTTGCTGCATTAGCTCTTAGAGGCAATCTAGCCACTCTGAACTTTGCTGACTCTCGATGGGGATTGCCAGTGCCGGCATCAAAGGACCCCAAGGACATACGACAGGCAGCAGTTATGGCTGCACAAGCTTTTTCTCAGGATACTGAATTAGTCGGAGTTGACTATATGAATGAGGAAGTTAATTCCAATACCATCGATGAAATAAAGTATCAAGAGATAGATATTGCGAGGGCTAATGATGGGAGTAGTAGTGATTTTGGCGCAAAGGAATTGTGTATAGATATGGAGAACATTTTATGTTGTAACTGGGGAGAAGATAACGACATGTTGGAGATGGAAGGATGGCAAGAAAAGATGGCAAAGGGGCTTTTGTTTTCACCAACTCCGCGTTTAGGTAGTTGTTTCAGTTGGGATGATGTAGAAAGCGACGTGGAGGTGTCTTTGTGGAGTTATAGTATTTGA

>Solyc08g007830.1.1 (SlERF8-3_DREB)

ATGAATAGTGATCACTTTAGAGTTCAAGATGAAGGAGTATTTCTACTAGCTTCGAGTCAACCTAAGAAACGCGCAGGAAGAAAGAAGTTCAAGGAAACTCGTCATCCAGTTTATAGGGGGGTGAGGAGAAGAGATAATAACAAGTGGGTGTGCGAGGTGCGTGAGCCGAGTGAGCAAAAAAGAATATGGCTAGGGACTTACCCGACTCCTGAAATGGCGGCTCGAGCTCATGACGTAGCTGCATTAGCACTTAGAGGTAACCTAGCTACTTTAAATTTCGCCGATTCTAGTTGGCGTTTGACTGTACCGATATCAAAGGATCCTGAAGAATTACGTAAGGCTGCCATACAAGCAGCCGAAGAATTAGTTGGAGTCGGTTATATGAATGAGGAAATCAATTATATTGAAGAAAATATTGCTATTGGTGGGGCCAATGGAAGTGACAATAGTAATTTGGTGGGTAATGAAGTGAATAATAATAATAACATACAAAATATGGAGATGGGTAACATTTCATGTTATAATAATTGGGGTGACAATAATGAAATGTTGGAAATTGAGGGATCAACATGGCAAGAGAAGATGAGTGAGGGGCATTTGTTTTCACCAACTCCCCATTATGATAGTTGTTTTAGTTGGGATGATGTGGAAAGTGATGGGGAAGTGTCTTTGTGGAGCTATAACATGTAA

>Solyc08g007840.3.1 (SlERF8-4_DREB)

ATGGCTGACACTTCAAATACTACAAAGTCAAGAACAAATACGAAACATCATGTATATCGCGGTATACGATGTAGAAGTGGGAAATGGGTGTGTGAAATTAGAGAGCCACGTAAGACAAAAAGGATATGGTTAGGTACATACCCTACACCACAAATGGCTGCTGCTGCCTATGATGTTGCTGCCTTAGCTCTTAAGGCTACTCAAAATATTGTACTCAATTTCCCTCATCTTGTTGATTCATATCCAAAGCTCCCTCCCTCTCCATCTCCGGCTGATATACAACGTGCCGCGGCCACCGCCGCTGAGGCAATGGCCTCACTAGGCTACGATGACGATCGGTCGTTAGATAGGAGAGATGGAGCTATAGGTAACGAAAGGGGTTCAACTGAATATCCTTCGTCATCAGGAAATTATGATATGCAAATAGGAGATGCGCAAACGACGATGGGTGAGGATGAATATGTTGATGAGGAAGCATTGTTTGAATTTCCAAATTTGGTTGTGAATATGGCGGAAGCAATGATGCTAAGTCCCCCTAGAATAAACTCGTTTCCATCGGAAGATTATTCATCGGGGGATTTCGATACTGAAAGTCTTTGGAGCTACTAA

>Solyc08g008305.1.1 (SlERF8-5_DREB)

ATGTCTTACACATATGATGAGGCTGCTCAAATTAGATCTTCTTTATCTCAAATCCTATTAACAAGTAGTACCAACACTTTAGACTCAATATTTTCTCATTGCCAAGAACCAAAAGATCAAATGACTACAACTAGTCCTGTTCTTGAACCACTATGTTCTTCAGTCTATCTCAGACAAATAGATTTGCTCCAAAAAATTTGGGAACAAAATAGGAAAAATATCACAATTCCAACAAGTTCCACACAAACCCAACTTCAAGAATCTTTATATTCTCAAGAATTTTGGGAACAAAATAGAGCAAATGTGACAATTCCAACAACTTCCACGCAAACCCCACTTCAAGAATCATTATATTCACAAAGATATGTTAGTCCAAACAAGAAAAAGTTGTATAGAGGAGTGAGACAGAGGCATTGGGGAAAATGGGTGGCTGAAATAAGACTTCCTCAGAAGAGAATGAGAGTTTGGCTTGGAACTTATGATAATGCTGAGGCTGCAGCTTATGCTTATGACAGAGCAGCCTGTAAACTTCGCGGGGAATATGCACGATTGAACTTTCCAAATCTACGCGATCCAAGTGAGTTGGGATTCGGAGATAGTGCAAAGATGAATGCTTTGAAGAATTCTGTGGATGCTAAGATTCAAGCTATTTGCCAGAAGGTTAAAAGGGGAAGAGCTAAAAAGGGAATTGTTGAAAGTGAGAAAAAGGTTGCAAAAGATGTGAATTTGGATTCATCATCTTCTTCTTCTTCATTGGTTGGTAGTGAAAGTTGGAGTCATAGTGATATGATTTCACAAAGTAGCTCTGATGACGGATTCTTGAATAGTGAGACTTGCTCTGTTATCGGTGACTGCCTGATGGGGCCAGAGTACGATAACTGCTATAGCACTAGCATCACACCTCAATTCGAAAGGCCTGTGCTGGAATCAGAGTTCGAAGATTATTCCTTAGCAACAATGCCTTCTTTTGATCCTGAGTTGATATGGGCAGTTCTGGCTAGCTAG

>Solyc08g066660.1.1 (SlERF8-6_DREB)

ATGAATTCAGTAGCAGCGCAAGATAATAAGAATGCGGAAATTATTAGTAATAAGTCGATAAAGAGAAATCGCGATAGCAGCAAGCATCCTGTTTTTAGAGGCGTTCGAATGAGGAATTGGGGAAAATGGGTGTCAGAAATTCGCGAGCCACGTAAGAAATCACGTATTTGGCTTGGGACTTTTCCAAATCCAGAAATGGCAGCTAGAGCTCATGACGTGGCAGCTTTAAGCATAAAAGGTAATTCAGCTATACTTAACTTTCCTGAACTAGCCGGTTTACTTCCTAGACCGGCTACACTTAGTCCGCGTGACATTCAAGCAGCCGCAGCTAAAGCCGCAGCAATGGACAAGTTCGATGATGATAATAATAATAATAATAATAATAATGATAATACTTCTCCGAATAATTCAACTTCGTCAACATCATCAAATATGTCCACATCTTCATCTTTATCATCGCTGGTATCGGCGATAGATTTGGCAACATCAGAAGAATTGACGCAAATAATTGAGTTACCGAAACTAGGTACATCTTTTGAATTGAAGAATGATTTTGTTTTTGCGGACTGTTCCGCTATGGAAACAGGCTGGTTATATCCTGAAGATGGAGGAATGATTTCTACCTGTTTTGATCCTTCTTTGTTGTGGAATTATTAA

>Solyc08g078170.1.1 (SlERF8-7)

ATGTTTGAAAATATAGATTTTGAAAATGATTATGCCTTTCTTCAATCCATTAAGCTTCAACTACTTGAAGATTGGGAATGGGAAAATCCATTAACAAGCTCAGATAATTCAATCTCAACTTATAGCCGAAACAATAGTATTGAGTCTAATACTTTTTCTAATGAGTTTGATTATTCGACTGACAATTTATCGAATGATTTTGATTGTTTAACTGATAAATTCCTCTCCGATTTGATTAATGATAATGGCTTTGGATATGGATCGGATCCTGTTATTCCAAATGTGAAATCGGAGCCTGAAATTTGGAATTTCGCGGAATTTGCGGCTGCGGCGGAAGTGAGAGTGGAACCACCAGCACAATCTGTTGTGACAGCGCCGCCGCAGAGGCTGCCGACGGCGAGGCATTATCGAGGTGTGAGACAGAGGCCTTGGGGAAAATTTGCGGCGGAAATTAGGGATCCGGCGAAAAATGGGCAAAGAGTGTGGTTGGGTACGTATGAGACGGCGGAGGACGCAGCGTTTGCTTATGATAAAGCGGCGTTTCGCATGAGGGGTTCACGTGCAATGCTGAATTTTCCACTGCGGGTTAATTCCGGTGAGCCGGAACCCATTAGAGTTAGGTCGAAGAAGTCATCAATGTCGCCGGAATGTTCTTCATCGTCGTCGGATAATGCGCCGGGGAAGAGGAGGAAGAAGGTCCCTCAAGCGGTATGA

>Solyc08g078180.1.1 (SlERF8-8)

ATGTATTCAAATTGTGAACTAGAAAATGATTTTTCAGTACTCGAATCAATTAGAAGATACTTACTTGAAGATTGGGAAGCTCCATTAACGAGCTCTGAAAACTCAACATCCTCAGAGTTCAGCCGGAGCAACAGCATTGAATCCAATATGTTTAGTAATTCATTTGATTATACACCTGAAATTTTTCAAAATGATATTCTTAATGAAGGATTTGGATTTGGATTTGAATTCGAGACTTCTGATTTTATAATCCCTAAATTAGAGTCACAAATGTCAATCGAATCACCTGAAATGTGGAATTTACCGGAATTTGTGGCTCCATTAGAGACGGCGGCGGAGGTGAAAGTTGAAACACCGGTTGAGATGACAACTACGACGACGAAGCCAAAGGCAAAGCATTATAGAGGTGTGAGAGTGAGGCCATGGGGGAAATTCGCGGCGGAAATTAGAGATCCGGCGAAAAATGGAGCACGAGTTTGGCTCGGTACATATGAGACGGCGGAGGATGCGGCGTTGGCTTACGACAAGGCGGCTTTTCGCATGCGGGGATCACGTGCATTGCTGAATTTTCCGTTGAGGATTAATTCCGGTGAACCGGATCCTGTTAGAGTTGGATCGAAGAGATCGTCAATGTCGCCGGAGCATTGTTCATCGGCGTCGTCGACGAAGAGGAGGAAGAAGGTTGCTCGTGGAACAAAGCAATAA

>Solyc08g078190.2.1 (SlERF8-9)

ATGTTGATGAATTTTTTCGCGTTAGAGAAAATCAGAACTCATCTTCTCGGAGAATTTTCACCGAGAACTTTGAAATTTGGGGCGGAATTAACGAGTAGTAAGTGTAGTGATACTGTGACGGAGAGTTGTGATTCTGAATCTGCTAGTTCTGGTTCTCTTCCTTTTGACTTTTTCTTTGATATTGAAGCTGATTTGTTTCAATTTGGCGCTGGAGTTTCTAATTCCAGCTCCGAATCAGCTAATTCGGTGTATAATCGAACAGATTTTGTTAAAATTGAATCGGAGCCGTCGATTTCAAGCTCTGATTATGAGGAGAATCAGAGTAAACGCTTTCAGTTTAAATCGGAACCTCAAGTATTCATCGATCTTACTTCACCGAAATCAAGGAAATTGTGTAGCGCTAGTGATAGGAAACCTTCACTGAAAATTGATTTACCTCCGGTGAAGAAGTATGAATGGATCGATTTTGGTAATTCGGCTCAATCGAATCCGATTGTTTCGGTTCCTGTGAAACAGATTAGGGAAGCTGAAGAGAAGAGGCATTACAGAGGAGTGCGGCAGCGGCCATGGGGAAAGTACGCGGCGGAGATCCGAGATCCGAGACGCCGTGGTTCTAGAGTTTGGCTTGGAACATTTGATACTGCAATTGAAGCGGCGAAGGCTTATGATAAAGCTGCATTTACGATGCGAGGAAGTAAAGCGATTCTGAATTTTCCTTTGGAGGTAGGGAAGACTCTGTCGTATTCAAGCTCCGCCGTGGAAGGCGGCGTGAAGCGGCGGAGAGAAGCGGTGGAAACTGAAGAAGAGAAGGTGGTGAAGAAGTGTAAGGAAGAGGAGTCATGTCCATTAACGCCGTCAAGTTGGAATTTCGTTTTTGAACAGAACTGTAACGGAATGTTTAACCTCCCTCCATTATCACCGTTATCTCCTCACCTAAGCTGTTCGCAGCTTATTCAAATATGA

>Solyc08g078410.2.1 (SlERF8-10_DREB)

ATGGAAGAAAAACATCAATCTCCCATTATTTTCAATAACTATCAAACAATTTCTTCTTCATCTCTAACAAAAAGATCAATATCCTTGAAATCATCGAAACAACAAACCAATCAAAACAAGAAAATCAAATCCAGTACTGAAAAAACCAAGAAAATGGACGATTCAAAACACCCAATTTACCACGGTGTTCGAAAACGAAGCTGGGGAAAATGGGTGTCTGAAATTCGCGAGCCGAGGAAAAAATCAAGGATTTGGCTTGGTACATTTTCTACAGCTGAAATGGCTGCCAGAGCACATGATGTAGCTGCAATTGCCATTAAAGGTCACTTAGCTTTACTCAATTTCCCTGAATTAGCTCATCAATTTCCTATACCATCTTCGAAATGCGCTAAAGATATTCAATCTGCAGCCGCAAAGGCTGCAGCTTTGAATATTTCACCAAGAAATTTTATGGAAAAACAGAGTACAGATGAGTTAATGGAAATGCCCTGTTCTTCTGAAGAAACAGAGTCAGCAGAATCATCTTTGACGAGTAATAGCGAAGACCCTTTTCTTGATTTGCCTGATCTTCTTATGGATCTCACTCAAAAATTTGATAAATTCTGTTACTATAATTCACCGTGGGATTTGGCTGGAGCTGTAAGCTTTTGGCCTCAAGAAGAGCTCTATTCGTGGGATTACAACCATGAAACTGAAACCCCACTCGAAGTTTCACAGCCATAA

>Solyc08g078420.2.1 (SlERF8-11_DREB)

ATGGAAGAAAAACATCAATCTCCCATTATTTTCAATAACTATCAAACAATTTCTTCTTCATCTCTAACAAAAAGATCAATAACCTTGAAATCATCGAAACAACAAACCAATCAAAACAAGAAAATCAAATCAAGTACTGAAAAAACCAAGAAAATGGACGATTCAAAACACCCAATTTACCACGGTGTTCGAAAACGAAGCTGGGGAAAATGGGTGTCTGAAATTCGCGAGCCGAGGAAAAAATCAAGGATTTGGCTTGGTACATTTTCTACAGCTGAAATGGCTGCCAGAGCACATGATGTAGCTGCAATTGCCATTAAAGGTCACTTAGCTTTACTCAATTTCCCTGAATTAGCTCATCAATTTCCTATACCATCTTCGAAATGCGCTAAAGATATTCAATCTGCAGCCGCAAAGGCTGCAGCTTTGAATATTTCACCAAGAAATTTTATGGAAAAACAGAGTACAGATGAGTTAATGGAAATGCCCTGTTCTTCTGAAGAAACAGAGTCAGCAGAATCATCTTTGACGAGTAATAGCGAAGACCCTTTTCTTGATTTGCCTGATATTCTTATGGATCTTACTCAAAAATTTGATAAATTCTGTTACTATAATTCACCATGGGATTTAGCTGGAGCTGAAAGCAATGACTCTAATTACAGTGAATTCTGGCCTGAAGAAGATCTCTGTTTGTGGGACTACAGCCTCGAAGTCACACAATTGTAA

>Solyc08g080290.4.1 (SlERF8-12_DREB)

ATGGCTGACTCTCAAAATTCTACCAAGTCCACCCAAATAATTCAACCTCCTAATCAATCAAGTGAAAGTACGAAAATACCCTTCAACACTACCCTTCCTCCACCACTACCACCGTTGCCGCCGCCTCCGGCGCCACCGGAGACACATATTCAAATAACCAATACATTATCATCAAAATTAGAAAACACAAATACAACTCCAAAAACCGTTATTATTCCAACTGCAATCATGAGTTCCACCTCATCGAGCGGCTTGGGATCTCCGAGCCGCTCAATAGGCGGAAAACATGCGGTATTCCGCGGAATACGATGTAGGAGTGGGAAATGGGTGTCAGAAATTCGAGAGCCACGTAAGACTACGAGAATATGGTTAGGTACTTATCCTACACCCGAAATGGCTGCTGCTGCATACGACGTCGCTGCAATGGCTTTAAAAGGAAACGACGCCGTAATTAACTTTCCCGGTAATATTAATTCGTATCCTTCACTCCCTCCTTCGCCCGCCGCGGCGGATATACGAAAAGCTGCTGCCACCGCCGCCGCCCTGATGAAGATTGAATCCGGAGAAACCACCTCGGGAACTCAACCAGGTACGGATGATCATCGGTCATTGGGGAATGTGCATATGATGGAAACTAGGGATCATGAATACATTGATGAAGAAGCATTATTTGATATGCCTAATTTGCTAGTTGACATGGCGGAGGCAATGATGGTTAGCCCTCCAAGAATGAGCTCACCGCGGTCTGATGATTCACCAGAACATTCTGATGCAGAAAATCTATGGAGCTATTAA

>Solyc08g081960.3.1 (SlERF8-13)

ATGGACCAACAAACCATGTTGTCTAGTGGTGTTAAGTATACCGAACATAGAAAGCAAATCACTATGGTTAGACCAGCACCTCCGGTGACTTTTAATGGTCGGAGGAGAAGCTCTGAGATGAATGCTGCCGGTCCTAGAGTTGTAAGGATTACTGTTACTGATGCTGATGCTACTGACTCTTCGAGTGATGAAGGGGAACAGGGGTTTTATGGCCGTCAAAGAGTGAGAAAGTTTGTGAATGAAGTTCGGATAGAGCAGAGCAGCCACTGTAATGGAAGTGTAAATGGGGTTTTGAGAAATGGGTCATCGTCGGAAACTGCTCCCGTTGTCGCTGCAGCACCGAAGAGGAGGAAGAAGACCGCCGGAGCAACAACGGCGGCGAGTAAGCTAAAAGTGAACCATGTTAAAAAGTTTCGCGGCGTACGGCAACGGCCTTGGGGCAAATGGGCGGCAGAAATCAGAGATCCACTGAGACGTGTACGGTTATGGTTAGGCACTTACGATACTGCTGAAGAAGCTGCCATGGTTTATGACCACGCGGCTATTCAGCTACGTGGGCCTGACGCGCTCACAAACTTCGCCACTCCTCCGGCGACGAAAATCAGCTGCTCCAGCTACAACTCCGGCGAAGAATCGCACAACGATCAACGCTCGCCGAAGTCCGTTCTACGATGTGCTTCAACTTCATTTGATGAATCTCAGAATAACGAGGAAGCCGAAGCTGAGTCATTACCGATTTTACCCTCCGATATCAGGGACAAAAATGACATTTCCATGTCGGAAAACTTCTGCGATTTACCACCGTTCGAGAGTTTCTTCCCCGATGATTTGTTTCAGTTCGAGAACCCGATTACCATATCCGACTTGTTTTTTGAACCGGATACTTTACCCGACTACAATAATATGTTATTCGGGTCAAGTACTACAGATTACGGTTTCGGATCTTCATCCTGGCCCGAAGAGGACTTCTTTCAGGATTTCGGAGATGTATTCGGGTCTGACCCGCTTGTGGCTCTTTGA

>Solyc08g082210.4.1 (SlERF8-14_DREB)

ATGGACAGAATTCAAGAAAACAACTCTATGTCTAGTATATTTGATGAGGGTGCTCAAATTCGATCTTCATTATCTCAACTAATTCTAACAAGTAGTACAAACACATTCGATACAATTTTTTCTCATTGTCAAGAAAATAACCAAATGAGTAATAGTCCTGTTTTTGAGCCTTTAGGTACTTCAGTCTACCTCAGACAAAGAGATTTGCTACAAAAATTCTGTCAAGAAAACATAGCAAATATCTCAATACCAACAACTTCGAAAACAATCCCTTTTCGAAACTCTTTGTATACACAAAGTTATAAGCTTCCAGAGAAGAAAAAACTATACAGGGGAGTCAGACAGAGGCATTGGGGAAAATGGGTTGCTGAGATAAGACTTCCACAGAATAGAATGAGAGTTTGGTTAGGTACTTATGAAACTGCTGAAGCTGCTGCTTATGCTTATGATAGAGCAGCTTATAAGCTTCGTGGCGAATATGCAAGATTGAATTTTCCTAATGTTCGCGATCCGAGTAAGTTGGGATTTGGAGATGGAGAGAAAATGAATGCTGTGAAGAATGCAGTGGATGCTAAGATTCAAGCTATTTGTCAGAGGGTGAAAAGGGAGAAAGCTAAAAAGGCTGCAAAGAAAAAGAGTGAGAATGAGAGTAAAGTTAAAGAAACTATGGATTCATCATCTTCTACTACTACTACTACTATGATTTCACCGAGTGTTTCTGAAGATGGATTATGGAGAAGTGAGGATTCAACTTGTTCTGTTTTTGGTGATTGCTTGAAGGATCCATTGATGGAGTCTGAGTTTGATAGTTGTTCACTTGCAAGAATGCCTTCTTTTGATCCTGAATTGATATGGGAAGTTCTTGCTAATTAG

>Solyc09g009240.1.1 (SlERF9-1_DREB)

ATGACAACTACTACTTATGGTGGAAAGAACATTATTATCGGGGGCAGTAACAACTCTAGAACTGCCCCCAATAATAAATCTTATTCAGGGCGTCACCCCGTTTACCGAGGAATAAGGAGAAGAAAAAGTAGTGGAAAATGGGTGTCAGAAATTCGCGAGCCAAAATCCCCTAATAGGATTTGGCTCGGGACGTTTCCGACACCCGAAATGGCCGCCATCGCGTATGACGTGGCAGCCATAGCCCTAAAAGGTCCCGAGGCGGATCTTAACTTCCCGAACTCGGCTGCCTCTTTTCCTGTTCCTGCCACGAGCTCTGCTCGTGACATCCAGACAGCTGCAGCCAGGGCGGCTGCAGCTATTGGGGCAGCCGGGGACGCGTTGTTAGCCGCGGCTGCGGTGCATAGGGAGGTAGTAATTAATGAAAAGGGTAAACTGGAGAATGTGGAAATTCAAAATTTTGAGTTTATGGATGAGGATTTGATATTTGATATGCCAAATGTTCTTGTTAATATGGCTGAAGGGATGTTACTTAGTCCACCAAGGTTTTATATGCATGATGATGATGATCAAACTACTATTACCTACCAAAACTTGTGGAATTATACCTAA

>Solyc09g059510.3.1 (SlERF9-2)

ATGGTGAGCATCCGAAGGCGCAAGCTGTTAGGACTTTGCTCTGGACGGAGCGCCTTCTTGGTTCCACTGCCAAAATTCTCTGAGAATGGGCACTTCGCAGAACATCGTTTCTTCAGCAACAGACCCACTAGTGTACACCCAATGCCATCAACTGATATTGATGAGTCAAAAGAGAAAATTGCTGTAAAAGTTGTTCCTGGATCATCGAATAGTCATGCCTCTGGCTCCTTGATGGAGCAAACCGCTCAACAATTTCCAGAAGTTAAACGCAGAAAGCGACACAGGAGAAAGCACTTTGAAAACCAAGAACCATGTCTCATGAGAGGTGTCTACTTTAAAAATATGAAATGGCAAGCTGCAATAAAGGTTGATAAAAAACAAATCCACTTGGGTACAGTTGGCACTCAAGAAGAAGCTGCTAGACTGTATGACAGGGCCGCTTTTATGTGTGGGAGGGAACCGAATTTTGAGCTTTCCGAGGAAGAGAAGCAGGAACTAAGACAATTCAAATGGGATGATTTTTTGGCATTTACACGTTCTGCGATTACTAATAAGAAAACTCGAAGAAGAAGTGGGGCTGGTGCACGGAGGAAATTCGAGCCTTTAACTTCAGCACTGAACAGTGAGGAGGATGAGGAAGAGGAGGAGGGAGGGGAGCCGGAAAGCAACAGTTTTTCAGCCTCTGAAGATATAGAGCACGACATATTGTTCTCTTGA

>Solyc09g066340.1.1 (SlERF9-3)

ATGAATGAAGATAATTCAACTATTAAGAAGAAAGAATCATGCGTAGGAGAAGTAAAATATAGAGGAGTAAGAAAGAGGCCATGGGGAAAATACGCGGCGGAGATAAGGGATACTAATAATAATGGATCGAGGGTATGGCTAGGAACTTATGCTACTGCGGAGGATGCTGCTAGAGCTTATGATAAAGCTGCATTTCAAATGAGAGGACGTTTTGCGGTACTTAATTTTCCTCATGAGTATCCATCTGATCATCATTCTTCTTCTTCGTATTCAATGTCATCTTCGTCAACGTCGTGTTCATCAGTGAAACAAGTTATTGAAATTGAGTACTTGGATGATAAGTTATTGGAGGAACTTCTAGGCTTGGAATCTGACAAATCTCAGTATAATAAGTAG

>Solyc09g066350.1.1 (SlERF9-4)

ATGGAGAACGAAGATCAAAGAGGGAGAAGAGAGAACAAATATCGAGGCATACGACGAAGACCATGGGGTAAATATGCTGCTGAGATACGTGATCCTAATAGGAATGGTGCACGTCTATGGTTAGGTACTTTTGAAACAGCTGAAGAAGCGGCTAGAGCTTATGATAGAGCCGCGTTTTCTCTAAGGGGTCATCAAGCCATTCTCAACTTCCCTAATGATGGACATTATCATATCAATAATAATACTAATGTACCTCTAGGTGTTGGACCAAGCTATAACACGCCATCTACAATGGGGAACAATATGAACATGTCCTCTTCTTCTTCTGCTTCGGCTTCTGCTTCTGCTTTTTCTGATGATAGACATGATCACGGAGAGAAGGTTGAGTTTGAGTATTTGGATAACAATCTTCTAGACGAACTTCTTGCTTCACAAGTTCATCGCCATGATAATAAACGTCCTAAGTTTTAG

>Solyc09g066360.1.1 (SlERF9-5)

ATGGATTATTCATCTCGGGATGATCTTCTTTTTCACTATAATTCACTTCCATTTAACGTTAACGATACACAAGACATGTTACTTTATAATCTTGTTGCTGAAGGATCATCGCAAGAAACAGTGAATTCGTCGTCTAGCTATGGAATAAAGGAAGAGGAAGTGACCTCATATGAAGAAGAAAGAAAAGATAAGAATTACAGAGGTGTTAGAAAGAGGCCATGGGGTAAATATGCTGCTGAAATTCGTGATTCTACGAGGAATGGTGTTCGTGTATGGCTAGGTACATTTGATAATGCTGAAGAAGCTGCGTTAGCGTATGATCAAGCTGCATTTGCTATGAGAGGTTCGATGGCCATACTTAATTTTCCTGTGGAGATAGTTAAGGAATCGCTAAATGAAATGAAGTGTAGATTTGATGGTAATTGTTCGCCGGTGATTGAATTGAAGAAGAGATATTCAATGAGGAGAAAGAGTGTTAGTAGAAAAAACAGAGCAAGAAAAGATGTTGTGGTTTTTGAAGATTTGGGTGCTGAGTATTTAGAGGAATTGTTGATTTCTTCAGAAAGTATCACAAATTGGTGA

>Solyc09g075420.3.1 (SlERF9-6)

ATGTGTGGTGGTGCAATTCTTGCTGATATCATTCCTCCTCGTGACCGCCGTTTGTCATCCACCGACCTATGGCCGACTGATTTCTGGCCAATTTCCACCCAAAATGTTCCTCTCAACCCCAAACGAGCTCGACCCTCTACAGGTGGTGAGCAGATGAAGAAGAGGCAAAGGAAGAATCTTTACAGAGGGATAAGACAACGTCCATGGGGTAAATGGGCTGCTGAAATTCGTGACCCGAGAAAAGGGGTTAGGGTTTGGTTAGGTACTTTCAACACTGCTGAAGAAGCTGCAAGAGCTTATGATAGAGAAGCTCGTAAAATCAGGGGTAAGAAAGCTAAAGTTAATTTCCCCAATGAAGATGACGACCATTACTGCTACAGTCATCCAGAGCCCCCTCCCTTGAACATTGCTTGTGATACTACTGTTACTTACAATCAAGAATCAAATAACTGTTACCCCTTTTACTCAATCGAGAACGTTGAACCTGTTATGGAATTTGCAAGTTATAATGGAATTGAAGATGGAGGAGAGGAGATGGTGAAAAATTTGAATAACAGGGTTGTAGAGGAAGAGGAGAAAACAGAGGATGAAGTGCAGATACTTTCTGATGAGCTGATGGCTTATGAGTCATTGATGAAGTTCTATGAAATACCGTATGTTGACGGGCAATCAGTGGCGGCGACGGTGAATCCAGCGGCGGAGACCGCCGTGGGCGGTGGCTCGATGGAGCTTTGGAGTTTTGATGATGTTAGTCGTCTACAACCAAGTTATAATGTAGTTTAA

>Solyc09g089910.1.1 (SlERF9-7)

ATGGAATTAAAATTAAACATGGAACAAGATCCTAGCAATGAAAGAAGAACTAATGGTGGTGGCGGAGAGATAAAATATCGAGGCGTACGTAGGCGTCCGTGGGGTAAATTTGCAGCGGAGATACGTGACTCAGCACGACAAGGGGCACGTGTATGGCTAGGGACATTTAATACTGCGGAAGAAGCAGCAAGAGCTTATGATAGGGCAGCTTATTCAATGAGGGGACATTTAGCTATACTAAATTTTCCTGAGGAGTATAATTTACCTAGTAGCTCTTCACATTTTTATAGTGCCGGTTCTTATTCTTCATCATCAATGGCTTCATCATCATCGTCATCGTCCTCGAGGCAAGTTCTTGAATTTGAATATTTGGATGATAAGTTGTTGGAGGAACTTCTTGATTGTGATGAAGAGCCAAACAAGAGGAAATAA

>Solyc09g089920.1.1 (SlERF9-8)

ATGGAGGGAAATTCATCTAGAGGAAAAAAAGCCATGCAAGAGAAGGATGAAGTAAAATACCGCGGTGTTCGTAGGCGGCCGTGGGGTAAATTCGCGGCGGAGATACGCGATCCAACCACCAGGCCCGGTTCCCGACAGTGGCTAGGTACGTTTGATACTGCTGAAGAGGCGGCTAGAGCTTATGATAAGGCAGCATTTAACCTGAGAGGACATCTTGCTACCTTAAATTTTCCTAATGAGTACTATAATCAACTTTCTTGTCCTCCTTTATATTATGGTAATAATAATAACAACATTATTTGTTCATCAACAAATAATGTTTCAAGAGGAAAAGGAATTAGTTCATCATCATCAACAAATTATAAAGGTAGGGAAATTATTGAACTTGAATGTCTTGATAATAGTGTTCTTGAAGAACTTCTTGGTGTGGAAGATCCAAAAATTACAAAAAGAAAATAA

>Solyc09g089930.3.1 (SlERF9-9)

ATGGATTCTTCTTCTTCTTCATCTCAATTCTTCTACTCAATGAATTCTGATTTAAATTCATCAGATTCTTCATACGAATGGTCCAATTTCAACACACAATCTTATCTCCCTTTCAACGTGAACGACTCCGAAGAGATGCTTCTCTTCGGAGTTCTTAACGCTGCTCATGAAGAAACAACATCCGAAACAGTCACATCGCATCGCGTTAAAGAAGAAGAAGTTACCTCAGAATCCGAGGTTATTGAAGCAATACCGGCGAAGGAGAAGTCGTACCGAGGTGTTAGGAGGCGTCCATGGGGTAAATTCGCAGCGGAGATAAGAGATTCTACGAGAAATGGGGTTCGAGTATGGTTAGGGACATTTGATAGCGCGGAAGATGCTGCTTTAGCTTATGATCAAGCTGCGTTTTCAATGAGGGGTAATTCTGCCATTTTGAATTTTCCAGTGGAGACCGTTAGGGATTCGCTACGTGACATGAAATGTCACGTAGACGATGATTGCTCCCCTGTAGTGGCGCTTAAAAAGCGCCACTCCATGAGGAAAAGGAGCACGAATTCCAAAAAAGTTAATAGTATTAGTAAAGTAGTGAGGGAAGTTAAAATGGAAAATGTAAATAATGTAGTTGTGTTTGAAGATTTGGGTGCTGATTATTTAGAACAACTTTTAAGTAGTAGTTCAAGTGATCAAAGTAGTTGTGATGCAACTTATTTTAGTCCATGGTAA

>Solyc09g091950.1.1 (SlERF9-10_DREB)

ATGGAGAATCAGTCTCTAAAATCAGAATCCATCATGCAAAAAGAATTGCCAACTTGTTATAAACAAGTAGCCATAGAATCATTATCTAGATTTTTCAAGGATCCACACATTTTTAGTGATTTAAATCATGAAATTACTTTTATAAATTCTTCTCCAACAAAATTTTCTGAATTTCTTCCACTTAATTTCCATCAAAATGAATCGTCATTATCATTATCATCATCATCATCATCATTATCGACTTCTTCAAGTTATCAACATCATCAAGATCAAGGGAATAATAACAACATTTTTCCTTTAAATAATTTTCTTGATCATCAAACATGTGCTTCTTCAACAAATTCACTATCAAGTAATTACCCAACTTTAGGGTTATTTTTGCAAGAGCCTTCCATTTTAGAGATTTCAAAAAGGGCAGCTGAATCTCTAAGCAACAAGAATCACAAGTCATCATCCGCGGCGTTATTCCCTATGTCTTCATCAATAGGTCATGAGAGCCAAATTCATTATAAAGAGGTTGCAACTACAAATCCAACAAATTGGTTGAAAATGAATCAAACCATAACAAATTGTACCACAACAAAAGGTTTTAGTGACTATTGGCTCAGCACTACAAAAACTCAACCAATGAAATTCAAAGGATCATCAAGAAAATCATCACTTGTTCATTATGAAAAGTCATCGTCGTCGTCATCATCATCATCTTCTTCTTCTTCTATGATCGCTTCCCAAGGGAAGCTTTTTCGAGGAGTAAGACAAAGGCATTGGGGAAAATGGGTGGCGGAAATAAGGCTACCAAGAAATAGGACTAGGGTTTGGCTAGGGACATTTGATACTGCTGAAGAAGCCGCTTTTGCTTATGATACCGCGGCTTATATGCTAAGAGGTGATTATGCAAACTTGAATTTCCCACATTTGAAGAATCAATTAAAGGCTAATTCTATCAATGGGAACATAACATCACTCCTTGAGGCGAAAATTCGAGCAATTTCAACACAATCAAAAAAGAAGGCTAATAATAGTAGTGATCGCGATGAAATTATTATATCTCCAAAAGGGCTATCGGATGATGATGCAACAATCAAGAACACGATCGTTCAGAATCAAGAGAAGGAAATTAATTGTGAGAAACTAATGGAGATTACTAAGGTGAAGAATATTAATAATCAAGAAAATGTTGATGGTGTTCAATTGAGTAGAATGCCATCTTTGGATATGGATATGATTTGGGATGCACTTCTTGTTTCTGATTTATCATGA

>Solyc10g006130.1.1 (SlERF10-1)

ATGAGAAGAGGCAGAGCAACTCCGGCGGCGGCGGCGGCGGCGGTGAAGCCAGATGGATCTGGAGGATTGAAGGAGATTAGGTTTCGTGGAGTTCGGAAGAGGCCATGGGGGAGATTTGCTGCAGAGATTAGAGATCCATGGAAGAAAACTAGGGTTTGGTTAGGTACTTTTGATTCAGCTGAAGATGCTGCCAAAGCTTATGATGCTGCAGCTCGAACTCTTCGTGGACCTAAAGCTAAAACTAATTTCCCTTTACCTATGTATTCTCAGCATCATCAGTTCAATCGAAGTTTAAACCCTAATGATCGGTTAGTTGACCCGAGATTGTACTCACAAGAAGCTCCGATCATTTGTCAAAGACCTACATCGAGCAGTATGAGTAGTACTGTGGAATCATTCAGTGGACCGAGACCGCCACGTCAGCAAACGGCGGTTTTGCCTTCGAGAAAACATCCTAGATCGCCGCCGGTTGAGCCGGATGACTGCAGGAGTGATTGTGACTCATCGTCTTCTGTTGTTGAAGATGGTGATTGTGAAGGGGGAAATGACAACATCGTTTCTTCATCTCTCAGAAATCCACTGCCTTTCGATCTCAACTTTCCACCTCCGATGGATGATGTTTATGCTAATTCAAATGATCTTTACTGCACAGCACTATGTCTTTGA

>Solyc10g009110.1.1 (SlERF10-2)

ATGGCGCCTAAGGAAAAAATTGGTGCAGTTACAGCTATGGCAATGGTGAATTTAAATGGAATTTCGAAAGAGGTGCATTATAGAGGTGTAAGGAAGAGGCCATGGGGGAGATACGCGGCGGAGATTAGAGATCCTGGGAAAAAAAGTAGGGTTTGGTTAGGTACTTTCGATACTGCGGAGGAGGCGGCTAGAGCTTATGATAACGCTGCTAGAGAATTTCGTGGAGCGAAAGCGAAAACTAATTTTCCGAAATTAGAAATGGAAAAAGAGGAAGATCTGAAATTCGCTGTGAAAAATGAAATCAATCGGAGTCCGAGTCAGACTAGTACTGTGGAGTCATCGAGTCCGGTTATGGTTGATTCATCATCGCCGTTAGATCTAAGTCTCTGTGGATCAATCGGCGGGTTTAATCATCATACGGTTAAGTTCCCGAGCTCCGGTGGAGGTTTTACCGGTTCGGTACAGGCGGTGAATCATATGTACTATATAGAAGCACTTGCACGCGCCGGAGTTATAAAGTTAGAAACAAATCGGAAGAAAACGGTAGATTACCTCGGTGGTGGTGACTCTGATTCATCAACGGTAATTGATTTTATGCGTGTTGACGTGAAATCAACCACCGCCGGTTTAAATCTGGATCTCAACTTTCCTCCACCGGAAAACATGTGA

>Solyc10g050970.1.1 (SlERF10-3)

ATGCAGAGGTCAACAAAACTTAGTGGTGAAGAAGAGTTTTCAATCATGGTTGCTGCTTTAACCAATGTGATTACTGGTGCTGCAACTATTTCATCATTTCCTTGTACGAGTGGTATGTCGCCGCCCTTGTTTCGCGTACCGGAAGAAACAGAGCGTTGTCAGTACTGCAAAATAAATGGTTGTTTAGGCTGCAACTATTTTGCAACCTCATCAGCTGCAGCTGGTGTTGTTAACAACAACAAGGCATTAAAGATTGTTGGGAAGACGAAAAAGAAGAAGAAGAATTACAGGGGAGTGAGACAGAGACCATGGGGAAAATGGGCAGCGGAAATTAGAGATCCAAGAAGGGCAGCTAGAGTATGGCTTGGAACATTTACTACAGCTGAGGACGCAGCTAGAGCTTATGACAGAGCAGCTATTGAATTTAGAGGTCCAAGAGCTAAGCTTAATTTCTCATTTACAGATTACACTTCAATTCAACAACACAATACTACTACACCCATGCAAGTGCTGCAACAACAACAACCAGCTCCCTCGCAGTTACAACAAGGAATAAACACAGAAGAAGAAGAGTTCTGGGATCAATTGATGAATTCGGACAATGAAATTCAACATTATCTTTATAGAGAATCATCTGATTCTGCTAATGGCTATATTGCTCATAGCTTCTAG

>Solyc10g076370.3.1 (SlERF10-4_DREB)

ATGGATTTTTATGATAACAGGAAAGTGAAAAGAAGGCGTAATGGATCTGATTCGATAGAAGAGATTTTATTGAGATGGAAAAATTTCAATCAGGAAGTTAATTGGAACCATGAACAAGTGAAGAAGAAGAGAAAATCTCCGGGTAATGGTTCAAACAAAGGTTGTATGCCAGGCAAAGGCGGTCCTGAGAATTCGGGTTGTAAATACAGAGGAGTGAGACAGAGGACTTGGGGTAAATGGGTGGCTGAAATTCGCGAGCCTGTTTATATTAGTGGTCAGTATAAGAGCAAAGGAAAGCGACTTTGGCTTGGTACTTACTCGACTGCTGGTGATGCTGCTGTTGCTTATGATGAAGCTGCTAAGGTTATGTATGGATCCAATGCCATACTTAATTTCCCCAATTCGAGTAATGGTAACATTACTAGAACATCGAGTGGTCAATCGTCGATTGATCATGAAGAGTCGGTGGTTGATGATGAGAAGAAAACTGAAATTGAGTCGGATTTGAAAGATGATGATGGTGGTGTTGTTGTAAATATGGATTTAAGTTATGATTATGCGAATCATGGTTCGCCTGCTTGTTCCTGGACTGAAGAGGAATTGGAAGTTATCACGGAGGAGAATTCCGAGATAGAATTAACCAATTTGGAGTGTGATTCAAGATTTTTTCATAAATCTCATGTTAAAGTTGAGAGGCCGATAATGGAAGAAGAAATTGATGAGGATGAATTTGTACATAACGACGTCTCAAACACAATAGACGTGGAGCCAACCGTCATGTTTAGCAAAGATGACTTCTCGAGGCTTGATGAAACTCGTAATTCTAATGATCAAATTGTGCTACAAGACATGGATTTCAGATCTTCTGAAAATCTGAACGAAGATGTTAGTACGCGTCTAGAATACATGGAGCATTTTCTAATGGATGACAATTGTTCGATGGAAGCAGCAAATATATCAGACATCATTTGTTTGACAGAGAACCATGATGAAGCTTTCGATTTTCAGAGGTTTTTAGAAGAATCGTTTGATTTCGAGCTAAACTATGCGAAGAATGAGGAGCAATTTGATTGTACATATGCATACAACCAGCAAATTGACCACCAGAATTCAGAGACAAATTTCGAGATTCGATCAGATGGAATTCGGAAGGAGAAAAACTTGCATGGTTTCGGATTAGATGACTTTGGAGCAAGCAATAACCAGAGGAAAATATAG

>Solyc10g076380.2.1 (SlERF10-5_DREB)

ATGCAAGGCAAAGGTGGTCCTGAGAATTCGAGTTGTAAATACAGAGGAGTTAGGCAGAGGACTTGGGGTAAATGGGTGGCTGAAATTCGCGAGCCTGCTTATATTAGTGGTGATAATAAAAGCAAAGGAAAGCGACTTTGGCTAGGTTCTTTTGATAGTGCTGATGAGGCTGCTATCGCTTATGATGAAGCTGCAAAGGTTATGTATGGATCCAATGCCACACTCAACTTCCCCAATTATTCGAGTAATGGTAGCATTACTCGAACATCTTCACTTGAATTGAGTGGTCAATCGTGTGTTGATCATGAAGATTTAGTGCTTGATGGTTCAAAGAATGATGAAATTGAGTCGGATTTGAAAACATCAGATACACCAAATACAGATTTAAGTTATGATTATGTTAATCATGGTTCGCCTGCTTGTTCCTGGAATGAAGAGGACTTGGAAGTAATCATGGAGGAGAACTCTAAAAATGAATTAATTGATTCGGAGTGCAATTCAGAGATATTTACCTAA

>Solyc10g078610.1.1 (SlERF10-6_DREB)

ATGGAAATCAACTTTCAAACACATCATCATCAAAAAACAGAAATCTCAATGAAAAAAGTAACCAAATCTATCAAGGGAAGAAGAAGAAGAAGCAATAATAGTAATATTAACAGTAATAGCAACAAGTTTGTTGGTGTAAGGCAAAGATCTTCAGGAAAATGGATAGCTGAGATCAAAGACACAACACATAAAAAAATAAGAATGTGGCTTGGAACTTTCGAAACTGCTGAAGATGCTGCTCGTGCTTACGATCAAGCTGCTTTTCTCCTTCGTGGATCAAACACACGAACGAACTTTGTTACTACTCGTGTTTCAGCAGATTCACAACTTGCTTACAGGATCAAAAATCTTATTGATAGTAAGAAAATTGCAAAACAGAACAAACAGGGGAACTCTGTTTTTGGTTCAACATGTAGTGAGAAAATTCAAGAAAGTCATGAAAAAGTGCTTAATTCTAGTGATGATAAAACCTTTTATAATAAGCCTACAACTATTGCGAATCCCATGACTCAGTCTCAAACAAGTTGTGATCGGCTATATGAACCCTCACTGACAACGTGTCATCATTTAAATTCATCAGAGGCGGAGGACAACGACTTTTATAAGCCTGATATGATGAGTTGCTCGAGTTCGTGTAGTTCACAACCTCAAGTGTCATGGGGATTTGAATTTGCTGAAGAATTGTTAGATTTTCGTGTTATGGATGAGAGTATAAGTGAAATGGGATGTACAGAATTTGAGAGAATGAAAGTGGAAAGGCAAATATCAGCATCACTTTATGCTGTGAATGGTGTTCAAGAATACATGGAGAATGTTCATGATATTGATGATTTATCTCTTTGGGATCTTCCTCCTTTATGTTCTTTGTTATCTTAA

>Solyc10g080310.3.1 (SlERF10-7_DREB)

ATGGATAGCTACAAAAGAAATCCACTAAAACCATGGAAGAAGGGCCCGGCAAGAGGGAAAGGGGGCCCGCAGAACGCGTTATGTGAATACCGAGGCGTTCGACAGAGAACATGGGGTAAATGGGTAGCAGAAATAAGAGAGCCTAAAAAGAGAACTAGGCTATGGTTAGGATCTTTTGCTACAGCTGAAGAAGCTGCAATGGCTTATGATGAAGCAGCAAGAAGATTATATGGACCAGAAGCTTACCTTAATTTACCTCATATGAGAGCTAATTTCAATCCACTGAACAAATCACAAAAGTTCAAATGGTTTTCATCAACCAACAACTTTGTTTCATCATTGATACCAAATACCACTGGCTTACTCAACTTAAGTGCTCAGCCAAATGTTCATGAGAAGGAAGATGAATTCTCATCACACAACAATGTAGCCGTTCGTGGTATAGAAGATCATGAGGTAGCAGCAGAAGAGGCTAATAGCTTTTATAATGTGAATGATGAGCTTATGTTTCCATCTTCAATATGGAACTTTTGA

>Solyc10g080650.3.1 (SlERF10-8_DREB)

ATGGAAATTAACTTCCAAAAACATCAACAAAAAGAAGCAATTTCAACAAAATTCAAAGGGCGAAAACGAAGCAAAGGGCCTAATAGTTTTGTTGGTGTAAGACAAAGGCCATCAGGAAAATGGATAGCTGAGATTAAAGACACAACACAAAAGATAAGAATGTGGCTTGGAACTTATGAAACTGCTGAAGAAGCTGCTCGTGCTTACGATCAAGCTGCTGTTCTTCTTCGTGGATCAAACACACGAACAAACTTTCTTACAACTCGTGTTTCTCATGATTCTCCTCTTGCTTCACGCATTAGAAATCTTCTCAACATCAAGAAAACCGCGAATGAGAAAAGCCTGGACTGCTTACCTGATTCCACAAGTAGTAGTAGTACTAATCCAATTTCAAGTGAAAATGCAGAAAAGAGCAATCTTTATGAAAAATTACTTTCTCATGATCACGAAAGTCAATTTTTCGACAGCAACTCTGTTCAAAGTAACACTACTAGTGTGACTAGTTTAAACACCAGCAACTTTTCTGATCCAATGTCAAGTGAAAATGCAAAAAATAAATTTGAGGTAATGAAAGTGGAAAGACAGATATCAGCATCACTTTATGCAGTGAATGGTGTTCAAGATTACATGCAAATTAGCCAGGTTTCGATCCTGGGACCTGTGGGTTATGGGCCCACCACGCTTCCGCTGCGCCACTCTGATTTGTTGATACTGTTTCTGATATTTAATTTATTTTACACAATGAAATGA

>Solyc10g083560.1.1 (SlERF10-9_DREB)

ATGTCTGGAGCAAATAACGGATCAGGATCAGGATCAGGATCAGAATCAGGACGGCACCCGGTATATAAAGGGATAAGAAGAAGGAAAAGTAGCGGAAAATGGGTGTCAGAAATTCGGGAGCCGCGTTCCCCTAATCGGATTTGGCTCGGCACATTCCCGACCCCGGAAATGGCTGCCATCGCTTATGACGTGGCAGCACTTGCACTTAAGGGTCGGGATACCGAGCTGAATTTTCCAAACTCCGCCCCCTCTTTACCTGTCCCCGCTACCAACTCGCCCCGGGATATCCAGACTGCTGCGGCCTGTGCGGCCGCAGCAATTGGAGCAGCCGGGGACGCGTTAATAGGAGGGCGGAGTAACAATAGTAATAGTAATAGTAATAGTAGAACCGTTTCGAGAGACGTGGAGGATAATTATTTGATACCTAATAATAATGAGTACAATTTTATGGATGAAGATTTGATATTTGATATGCCAAATGTTATTATGAATATGGCTGAAGGGATGCTACTTAGTCCACCACGTCTCAACCACCTTCCTCCAGATGATGATTATATTGGTGGTGCTGATCAAAATCTATGGAATTATCCATAG

>Solyc11g006050.1.1 (SlERF11-1)

ATGGATTCTTCATCTTCCTCATCATCATACTCATATTCACAATATCATGATCATGATCTTGGTTTCAATGAGAATGACACTGAGGACATGCTTCTTCTTAATGTTCTATCCGAAGCCAAAGAAGATTCATCATCTAATAATATTCACGAAGAAACTAATGAAGGATCATCAAAAGAAGTGGCTACTAGTTATAGAGGTGTAAGAAGGAGGCCATGGGGTAAATACGCGGCTGAAATAAGGGATTCAACTAGAAATAGTGTTAGGGTTTGGTTAGGGACATTTGACACTGCTGAAGAAGCTGCATTGGCTTATGATCAAGCTGCATACGCGTTGCATGGGAGAGTGGCGGTGCTAAATTATCCAGTGGAGATGGTGTATCGATCGTTGATGGAGATTGATTGTAGATTTGAAGATGGATGTTCACCTGTTTTAGCTTTGAAGAAATGGCATTCTATGATGAAAAAGGGTAATAATAAGAGTAGTAGTAGTAATAAAAAGAAGGAAGAAAAAGAAATGAGTTATGAAAATGTGTTGGTTTTAGAGGATTTAGGTGCTGATTATTTGGAGGAAATTTTGATGTTGTCTGAAAGCAATTCCTAG

>Solyc11g011740.1.1 (SlERF11-2)

ATGAATCATTCTTTTTCAATAGAAACAAGTTGTTATGAAGAGATCAAAATCAAAGATGAAGAAGAGATTATTATAAATTTCGATAATAATATTCAAGATGAAGAAAATACACAAAAGGGTAAACAATACATTGGAGTTCGAAAACGTCCATGGGGAAAATATGCAGCTGAAATTAGGGATTCGACGCGAAATGGAATTAGGGTTTGGCTAGGTACGTTTGATACTGCTGAAGAAGCTGCTTTAGCTTACGACCAAGCTGCACTCTCAATGCGTGGTCCTACAACATGTCTTAATTTTGGAGTCGAGATAGTTCAAGAATCGTTAAGAGGATTTAAATATACGAGTCAAGAGGGTTCAACATCGTCTCCTGCTCAAGTTATAAAGGAAAAACACAAGAAGAGAAATAGTAAAGGTAAAAAAACAATAAAATCGAATAACCAAAAAAATAATAATAATGTATTGGTCCTTGAAGATTTAGGTACTGATTTATTAGATCAACTTTTATCTACTAAGTGTTCTACTTCTGAATAA

>Solyc11g011750.1.1 (SlERF11-3)

ATGGATTTGAAGATTGAAGATCATGATGAAAAACTAATTTCAAACATTAAAGTCGAAGTTGAAGTTGAAGTTGAAGATAAGAAAAAACATTATATAGGTGTTCGAAAAAGGCCATGGGGAAAGTATGCAGCTGAAATTAGGGATTCAACAAGGAATGGAATTAGGGTTTGGCTTGGAACGTTTGATACTGCTGAAGAAGCTGCATTGACTTATGACCAAGCTGCGTTTTGCATGAGAGGTCCTTTAACTTGTTTGAATTTTTCAGTAGATAAAGTGCGCGAATCGCTAACAAAAATGGAATTTATCAATAGCAAAGATGGATTGTTTATGTCAAGTCCAGCTGCAGCCCTAAAAGAGAAACACAAGAAGAGAAATGGTACTACTTCATCAAGAAAAAAGAAGAATAGTATTAAAGAAGAAAATGTGTTGATATTTGAAGATTTAGGTCCTGATCTATTAGATGAACTCTTATCATCTGAGTACTCATCTTGTTCAAATTAA

>Solyc11g012980.1.1 (SlERF11-4_DREB)

ATGGTGAAGACAGAGCAAAAAAGTCTATCAATGTCAATATCATCAATAGCAACAAGCAATAACAATAACATGAAGACAAAGAAGAACAAGTACAAAGGAGTTAGAATGAGAAGCTGGGGATCATGGGTATCAGAAATAAGAGCACCCAATCAAAAAACAAGAATCTGGTTAGGTTCCTATTCTACCCCTGAAGCTGCAGCTAGAGCCTATGATGCTGCACTTTTATGCCTAAAAGGCCCCTCTGCTTCCTCAAATCTCAACTTCCCTTTCAACTCTACTTTCTATCATCACATTGATCATCACACTTGTACAACTCTCTCTCCTAAAGCCATTCAGAGAGTAGCCGCGGCTGCCGCGGCCACTCCTGAACAACAAAATGTAGGGTTAGTACTGTCAGATAATAATAACAGTACTAACCCTACTATTTCATCTCCACCACAATCTTCTACTTCTTCTTCTGAGGATATAAATGATGGTTGTTTATTATCAAATATCAATACATTTGATCAAGAGATGTCAATGATTGAACAATGGTACAACTTTGATTCACCAAAGTACAATGACATGTTACATGGTACAATTTTCTTTGATCCACCTTCAATGGAGGAGGTTTATTATGAAGAATCATCAGCAGATATACCTTTATGGAGTTTTTGCTAA

>Solyc11g042560.1.1 (SlERF11-5_DREB)

ATGGAAAACGATCAAGAAAGTAAGAATAATATGGACGTGTATAGAGGAGTAAGGAAGAGAAAATGGGGGAAATGGGTGTCTGAGATACGCGAACCAGGAAAGAAAACCCGAATATGGTTGGGGAGTTATCAGAAAGCAGAAATGGCTGCTGCTGCTTATGATGTTGCTGCATTTCATCTAAAAGGTGAGAGACCGAACTTAAGGCTCAATTTCCCTGAATTAATCCATACATTCCCAAAACCCTCAAGTTCAAGACCTGAAGACGTACAAATGGCAGCTCATGAAGCGGCAATGAGGTTCAAACCGTCAATTGATGATCATCCTGAGGAATGTGGTGTAGGCCAGGTGAGAGTAGGTCTCTCACCTAGTCAAATTCAGGCGATTAATGAATCCCCTTTGGACTCACCCAAAATGTGGATGGAGTTAGCCGGGGCTCTATTACCTGTCAGAGAATACACTTGTCCCACCGATTATTTTGAGGATGAAACCCCACACCATCATGAGTCCATTTGGGATTTTTAA

>Solyc11g042580.1.1 (SlERF11-6_DREB)

ATGAGTGGTATAACATCAGAATATAGAGGTGTTAGAAAGAGGAAATGGGGTAAATGGGTGTCTGAAATCCGTGAGCCAGGGAAAAAAACGAGGATATGGCTAGGGAGTTTTGAGACAGCACAGATGGCTGCAGCTGCGTATGATTCAGCTGCATTTCACTTCAGGGGACATGCAGCTAAGTTAAATTTCCCAGAATTGATTGAACATTTCCCTAAACCAGCAAGTTCTTCGCCTGAAGATATACGTTTGGCTGCTCAACAGGCTGCAATTACAGTTCAAAACACGAATTCAATGGCCTCTTCTAGTCATGGACATGGAGCACCAGTTACAGTAGGATTATCTCCAACTGAAATTCAAGCTATTAATGACTTCCCTATGGATTCACCACATATGTGGACTACTCATGAAGACATCAACTATACTTATGAAATGAGTGATTACCAAGAAATGGATGATTGTCTTTGGGACTACTCATCAACTAACTCATTCTAG

>Solyc11g045680.3.1 (SlERF11-7)

ATGGATTTGATCAAATCATCGACTTCATCATCAACTAGTAAGAGCACGAAACCAAGAAAGAACAATGGTACTAGTAATCAAAGTGTCAAGCAAAAAACAGAGGAACAACAGAGCAATTTTCGATATCTGGGAGTAAGGCGAAGGCCATGGGGTCGATATGCTGCGGAGATAAGAGATCCAAACACGAAAGAAAGGCATTGGCTCGGAACATTTGACACTGCTGAAGAAGCTGCGTTGGCTTATGATAGAGCTGCACGTTCCATGCGTGTTAACAACAAATCTAACAAACCAACTCGGACTAACTTTGTCTACTCTGACATGCCACATGGCTATTCGGTTACTTGTATAGTCTCCCCTGACGATCAATATCAACACCATCACCACCACCAACAACAACAGTTATTGATTTTTGGTCAGACCGAGAATAATCCTGCTCCTAACGTCGATTATGGGGCCCACTTCAGTCATTTTTCCCTCAGCAACATGAATAATATTGGTGGCGATAGTTATGATGATGGAGAGTTTGCGCTACAACATTACTGTAACCCCAACTACGACGTGCATATGGAAGATGGTTACAGGTATGATAGTAATACAACATCAACAGAATTGCCACCATTGCCAGAGGATATAACAAGCAGTGGTTACTACTACAACTTGAGTAGTGAATTTCCATATTCAGAAATGGGGTATAACAGTAAGAATGAAATGATAATTGGTACAAATAATGAATATCAATATGGAGCAAGTACAATCACTACAACAAGTAGTACAACAACAACAGCTGGGAGTTTTAATTATTTTGGTTTTGATGATTGTTTACAGCCACTGCAAGATGAATCCAACAATAATCGTTTGGGTTATTTCTTCTCATAA

>Solyc11g045690.3.1 (SlERF11-8)

ATGGATTTGATCAAATCATCCACTTCTTCATCAACTAGTACGAGCAGGAAACCAAGAAAGAACAATGGTACTAGTAATCAAAGTGTCAAGCAAAAAACAGAGGAACAACAGAGCAATTTTCGATATTTGGGAGTAAGGCGAAGGCCATGGGGTCGATATGCTGCGGAGATAAGAGATCCAAACACGAAAGAAAGGCATTGGCTCGGAACATTTGACACTGCTGAAGAAGCTGCGTTGTCTTATGATAGAGCTGCACGTTCCATGCGTGTTAACAACAAATCTAACAAACCAAATCGGACTAACTTTGTCTACTCTGACATGCCACATGGCTATTCGGTTACTTGTATAATCTCCCCTGACGATCAATATCAACACCATCATCACCACCACCACCAACAACAACAACAACAACAACAACAACAACAACAACAACAACAACAACAACAACATTTATTGGTCTTTGATCAGACTGAGAATGCTCCAGCTCCTAATGCCGATTATGGGGCCCACTTCAGTCAGTTTTCCCTCAGCAACATGAATAATGTTGGTGGCGATAGTTGTGATGGTGTAGAGTTTGTGTCACAACAATACTATAACCCCAACTACGACATGCATATGGAAGATTGTTACAGGTATAATAGTAAGAATAAAACAACAACAGAATTGCCACCATTGCCAGAGGATATAACAAGCAGTGGTAACTACTACAACTTGAATAGTGAATTTCCAAATTCGGAGATGGGATATGACAGTAAGAATGAAATGATAATTGGTACAAATAATGAATATGGAGCAAGCACAATGACTACAACAACAGCTGGGAATTTTAGCTATTTTGGTTTTGATGATTGTTTACAGCCATTGCAAGATGATCCATCCAACAATAACAGTTTGGGTTATTGGTTCTCATAA

>Solyc12g005960.3.1 (SlERF12-1)

ATGCCCCGTCGGAAAAATCCGGAGATAAATGGATCAAGATCCGTTGTTTTCCAACAGTCGGTGATTGAGCCACCTCGTTATAGGGGTGTACGTAAACGGCCATGGGGTCGATTTTCTGCTGAGATAAGGGATCCGGTGAAAAAAGCTCGTAAATGGCTGGGAACGTTTGATACAGCGGAGGGTGCTGCACGAGCTTATGATACTGCTGCAAGGTCTTTTCATGGAGCTAAAGCAAAAACTAACTTCCCAGTATTGCCTCCTTATGGGGAGAATCAATTTGAAAATCTTCAACAATCAAGGCCGGCGTCGAGTAGTATGAGTAGCACGGTGGAATCATCGAGTGATGCGCGTGCATTGTGTAAAACGATGCAACCGCGGATTGAAATTCCCCGCCGGATTCCGATGGGGGAAGGTCATAGTGATTGTGATTCGTCGTCTTCTGTGGTGGATGGTAATTGCATTGGCGGCGGCGGTGGCGATGATGATCAGACGTCGTCGTTTTGTAAAGATCCTCTGCCGTTTGATTTAAACTTTCCACCTCCCTCTGATGATTTTCAAATCATCACCCCTTTGTGTTTCTAG

>Solyc12g008350.3.1 (SlERF12-2_DREB)

ATGATAATAATGTCTACAGAGCAACCAAATTGTTCAGAAAGTACTGAATCTAGCTGCAACTCTTCTTCTTCTTCGTCGCCCTCATCGCCATCTTCCGTTCTTCTTCAGCCACTTCCACAAATCAATTCAAAAAATCGACTCAAAAGATGCAGAGGTGAAGAGGAAGTAGAAGAAGAAGAAGACGTGGTCGTTAATAATCCAAATCCGAAGAAGATGAATAAAAACAACAACAATGGCAGCAGTACTAGTGTTGTTTCTTATGTAGGTGTACGAATGAGAGCATGGGGAAAATGGGTATCCGAAATCCGTGAACCTAAAAAAAAATCACGGATTTGGTTAGGTACCTTTGCTACCCCGGAAATGGCGGCGCGTGCTCACGACGTCGCCGCCATGTCCATTAAAGGTACCTCAGCTATACTCAATTTTCCCCAATTTTCACATTTACTGCCCCGACCCGTCACGTGCTCCCCACGTGACATCCAAAACGCCGCCGTAAAAGCCGCTCACATGGATCACCTAAATCCAAAATTCTCAATATTACCCGAAACCTCTGCGGCGACGATGACCTCATCGTCATCGTCGCTGTCGTTAGTTTCGGGCGTTACATCCTCCTCGTCGTCGTTTCAAGACGACGAGGAGTCAAGACCGTCGCCTCCGGAGCTGATTCCGGAGGCGACGGGGCAGTTGAGTGAGATAGTGGAGCTGCCAAAATTGGGATCGAGTTACGAATTGGTTGAATCAACTCAGAGTTTATTTGAATCAGATGAATGGTGGGATAATAATTATGGAAATTGTGAATATTTTTTTGGACAAGATAATTATATTAGTAGTAATATGGAATTTACAGGATTAGAAAATGTGGTTTCAACCAGTTTTGAGAGTTTTTTATGGCAACATTAG

>Solyc12g009240.1.1 (SlERF12-3_DREB)

ATGGTGAAACCCAATTCGAAAGATACTGAATTTTCACAGTCGTCATCGTCGTCGTTGTATCGGGGAGTACGGAAGAGGAAATGGGGGAAATGGGTATCGGAAATTAGATTACCTAATAGTAGAGAAAGGATTTGGTTGGGTTCTTATGATACGCCGGAAAAAGCTGCTAAAGCATTTGACGCCGCACTTTTTTGTTTGCGGGGTAAAGGGGCTAATTTCAATTTCCCGGAAAATCCGCCGGAGATTAGGAACGGAAGGACGATGACTCCGTCGGAGATTCAATCGGCGGCGGCGCAGTTTGCGAATAATACAGAACCCGAACTTATCCGGGTCGGGCCAAGGGAGAATTCGGATCTTTCTTCTTCCTCATCGGAAATATTTCGAGCGGAATCACCGTCGGTGTCAGTTTCCGATAGAGTAGAAAGTGAAAAGACGGAAATAACCTTGGGTAACGATTTTATCGATGTGTATCGGGTGGAGTCTAGAGTGGAAAGTGAAAAGACGGAAATGTCCTTGGATAACGGATTTGTTGACATGTTTTCGTCGTTAGGAACGGTTAATGATATGTCTGATTTTGGAATTTTTCCGGGGTTCGATGACTTATCGGGTGAATTTTTTATACCACCACCATCGTCACCACAACCATCACCACTGCAAATGCCTAATTTGGAATCATTAGAAGAAGAGAATTATTTGAACTATGATGGATTTCAATCACAGGGGACTTCATTTCTTTGGAATTTTTGA

>Solyc12g009490.3.1 (SlERF12-4_DREB)

ATGGTACATTCAAAGAAGTTTAGAGGTGTGAGGCAACGCCATTGGGGTTCTTGGGTTTCTGAAATTAGACACCCTTTGTTGAAAAGAAGGGTATGGTTAGGGACATTTGATACAGCAGAAGAAGCAGCAAGAGCATATGATGAAGCAGCAATATTAATGAGTGGAAGAAATGCAAAAACTAATTTTCCAATAACACAAGATTTGGATAATAATAATAATAATAATAATAATAATAATAATAATAATAATAATAATAAAAATGTAAAGGGTAAAGATCAAGAATCATCATCATCATCATCATTTTCATCACCAAAAGCTTTATCTGAAATACTTCATGCCAAGTTGAGGAAATGTAGCAAGGTACCATCACCATCTCTCACTTGTTTAAGATTGGATATTGAAAATTCACACATAGGAGTTTGGCAAAAACGTGCTGGCCCTTCTTCTGATTCAAAATGGGTAATGACAGTTGAACTTCAAAAGAAAAATAATCCCAAAAATATTAATGTTCATGAAGGAGAATTAAATAATAATAATAATAATAATAATAATGATAATAATAATAATTCCAAGAATAGTTGTGGTGAAATAACTATTAGAAGTGAAATGGATGAAGAAGAAAGAATTGCATTGCAAATGATTGAAGAATTGCTTCAAAGATGA

>Solyc12g013660.3.1 (SlERF12-5_DREB)

ATGAAAAATATCGAATCAATTGATAATCTTCAATCCATAATGTCAATGTATAGACCTTTGAAAAAGCTCAAAATTAGCTCATCATCACAACATAATAGCCTTCGATCAAGTGGAGACGGGGTGGAGCCTGTAGCTCAGAGGATTAGAGCACGTGGCTACGAACCACAGTTTCGTTTTCCTTTTGCTCTAGATGATCCTCCATTAAGCAGTTGTCACATTAATTGGCAATCACTTACTCAAAATCAAACTATGATTTCTTTTGCACCTCAAAGTTGGTTTAATCAGTGTCGAATCATTGATAATGGAACGAGGACGACTTCAATGATGAATGTTTATGGCACTGTCTCCTCCCCTGCATCTAAACGATACAGGGGAGTTAGACAACGCCATTGGGGAAAATGGGTAGCAGAGATTCGCCTTCCTCGTAAAAGAACTAGACTTTGGTTAGGCACGTTTGAAAGTGCTGAAGAAGCTGCTTTTGCTTATGACGTCGAGGCCTTTAGACTCAGAGGAACAGATGCACGTCTTAATTTCCCACATCTGTTCCTCGGAGACCTGGCTCCGTTTGATAATAACATAAACGTGACTAATTCAGCATCGCCAAGTACTACTTCATTCGCGAAAAATCCTTTTCAAATCCAACACAATAAACGGTTCATCAGGCAATGTCGGAACAAAAAAATAGTACCTTCAGCTGTGGAACAGAGTACCTCTGTTTTTGACAAAGAAATAATGATGCAGGATAAGGCCGCATACAATAAACCCTTGTGGTATGACCCTTCCCAGGACTCTGTGCATAACGAGAGTTTAGTGCACGAGACTTCTCAAACAATGATGTTTGATGGGAGCGATTTTTATGAACCGACACCAAAATATACTAATAATATACAACAAGAAAATAGTGATATGTGGAAGAATGTACTTCAACCTACTCCATTTGGGAATTACAGTATTTGTACTGATGATAATTTGCAGCAACAAAAGCAGCAGTGTGATTTCACTATTACTGATCCTGGCTTCGGGGACAATGTTTTCGACTTTTCCTCCAAACTCCTCGGGTAG

>Solyc12g038440.1.1 (SlERF12-6)

ATGGCTTTTCCACCAAACGATGTGCTGTATAAAAGTGTCAGAAACAGGCCATGGGAAACATATGGAGTAGAGATTACAAACCCCATCAAGAAGGTACATGTGTGGCTTGGTAATTTCAAAACAGCTGAAGAAGCTGCTAGGGACTTTGATGAAGCGGCAAAGATGTATCACGATCCTAATGCCAAACTCAATTTCCCGCCGACGAATGAGGATAGATTCCAAAATTCTAATAATTTTGAAACCTAA

>Solyc12g038450.1.1 (SlERF12-7)

ATGGCGAAGAAAAGAAACGATGTGAAGGCGACGACGTTGGCAGTACCACCAAACGAGGTGCGGTATAAAGGTGTGAGAAAGAGACCATGGGGAACATATGGAGCTGAGATTACAAACCCCATCAAGAAGGTACGCGTGTGGCTTGGTACTTTTAAAACAGCTGAAGAAGCTGCTAGGGCCTTTGATGAAGCAGCAAAGATATATCACGGCCCTAACGCCAAACTCAATTTCGCACCGACTAATGAGGACAACCTCCAAAATACTAATAATTTTGAAACCTAA

>Solyc12g042210.2.1 (SlERF12-8)

ATGGTGGATCGAAGGTATGGGAAGAGGCCCTTTTCATCCAATGAATGGGAGGAGAAAGAGGATATTAATTTCCCTATTTATTCAGCTCGATCTCAACATGATATGTCTGCTATGGTTTCTGTTCTCTCTCAAGTTATTAGCAACACAAATAATACTACCAATATCTCTTCGTCATCGTCGATGCATGAAATTGATCCACTAACCCTACCTCAACCAACAACAAATCAAATTCACGAACAAGGGAACCAACAAAGAAAACGACATTATAGAGGTGTGAGACAAAGACCATGGGGAAAATGGGCTGCTGAAATACGCGATCCAAAGAAGGCTGCACGAGTATGGCTAGGGACATTTGATACTGCTGAAGCCGCGGCACTTGCTTATGATGAAGCAGCACTTAGATTCAAAGGAAACAAAGCTAAACTCAATTTTCCTGAAAGAGTTCAATCAGGAAATACTCAATATCTTACTACAACTCATCAACAACAACAACAACACTACGCATTTAATAATAATAATAATAATATTCCTCAGATGGTCACTCAGCCTAATTTATATCAACAACATTTCCCTAATGTTCATCACTATGCACAATTACTTCGCGATGGAAGCAATAATAATATTGATAATATGATGAATTTTGGGGTTTCAGATCAATCGAGTTTTTATCATCATCATCAACAAGGTAATTTTATTTCACCGAATACGTCGTTGGAATTACAACAACAACAACAACAAGCAAGTTATTATCATAATCAACAAGAAGATTTTCTAAGATATACAATGGATTTTGGTAATTCTTCAAATTATAGTACTGGACCACCTAGTGAAAGTAATTGGATGGATTTTGAACCTAAAAAGTAA

>Solyc12g044390.3.1 (SlERF12-9_DREB)

ATGGCGGAGCTTGTACAATCATCTTCCATATCCGTGTCAAAAACAGAGGAAAAGGAGAAACGAACAAGAGATGAAACTTACCCAGTATACAGAGGAGTTCGAATGCGAAGTTGGGGAAAATGGGTTTCTGAGATTAGACAACCGCGTAAGAAATCGAGAATTTGGTTGGGTACTTATCCAACACCTGAAATGGCTGCACGAGCTCATGACGTTGCTGCGCTTAGCATCAAAGGGAATTCAGCGATTCTCAATTTTCCTCAACTTGTTGAATCGTTGCCTCGTCCAGCGTCAATTTCACCTAGAGATGTTCAAGCAGCAGCTGCCATGGATGAACTAAATTCCTCTGTTTCTTCGACATTGAGACACAGTGAATCGATGGAAACGGAAGATCAATTAGGTCAGATTATTGAAATCTATAAAGTTAAATTCTGA

>Solyc12g049560.2.1 (SlERF12-10)

ATGTGTGGTGGTGCCATAATCTCCGATTGGATACCGCCGTCTCGATCTTCGAGCCGACTCACCGCCGACCAGTTATGGGGTTGCGCCGATCTGCAAAACAAGAAGAGGAACAAGAAGAAGAGGAATCCTTCCAATTATCACTCTAAGCGCTTGAGATCTGAGAATGTAGACTTTGAAGCTGATTTTCAGGATTTTAAGGATTTTTCTGATGATGAAGAAGCTTATAGTTTGGATATCAAACCATTTGCTTTCTCTGCTTCTGAACTCTCTGGAACCTCTGCTGGATCCGAATCACTGATATCTGTTGATGCAAACAAGGAAGTTGAGAAATCTGCCAAGAGACAGAGGAAGAATCAGTATAGGGGGATCAGAAAGCGTCCTTGGGGTAAGTGGGCAGCTGAAATACGTGATCCACAGAAGGGGGTCCGAGTTTGGATTGGAACTTTTAATACTGCAGAAGAAGCTGCCAGAGCTTATGATGCTGAAGCTCGGAGGATCAGAGGCAATAAAGCTAAAGTAAACTTTCCAGATGAAGCTTCAGTGCCTGCCTCGAGGCAAGCTGGTAAGGTGAATCCTCGGAAGGTTCTTTCTGATGAGAGCTCTAACCCAGTTCCACCCAACACCATGCTTATGAACAACTTGAATAGTGGATATTGTGACAATGTGGGCCTGCTCGAAGAGAAAACAAAGACTCTGAATGGCTACGAAGCTTTGTGTGTGACTCCTGTAGATACAGGACCTAACCCATATCCCCATCCAGCTGCTGCTGGTGTTTACTTCAATTCTGACCAAGGAAGTAACTCTTTTGGCCCCTCAGACTTTTGGGGAGAAACATGTTCAAGGACTCCAGACATATCATCTGTTCTGTCAGCTGCTATAGAATGCGATGAAGCTCAATTTATTGAAGGTGTCGACCTGGAGGAGAAACCAAAATCTTGTACCAACAATTTGGTGCCTAATAATGTGAACACTGAACACAAGCCACCTGAAGTATTTTCAACTTTTGAATCCCAGTTGAAGTTCTATCAGACACCATACTCAGAAGGAAATATGGATGTACCAGTCGATGCCTTCCTCGATGCTGATGCTACTCAGGGTGTTGAAAATGCTATGGACCTTTGGTCCTTTGATGAGCTTTCTTCTTTAATGGGAGGCATCTGA

>Solyc12g056430.1.1 (SlERF12-11_DREB)

ATGAATAACTCGAGTAATTCGTGTTCTTCGATACCTTCAGATGAAGGAAGTATTATATTAGAATCGTCTAACAAGTCGAAGAAGCGAGCGGCCGGGAGGAAAAAGTTCAAAGAAACACGTCACCCGGTGTTTCGAGGAGTGAGAAAGAGAGACAATGACAAGTGGGTTTGCGAAATTCGCGAACCCACTAAACAAAAGCGAATATGGTTAGGTACGTACCTAACCGCGGAAATGGCTGCACGTGCTCATGACGTAGCTGCATTAGCGCTTAAAGGAGAATTAGCCAAGCTGAATTTTGCGGACTCGGCTTGGCGTTTACAAGTTCCGGTGTCTAAGGACCCGAAAGAGTTACGCCAAGCGGCTGCTAGAGCCGTCGAGGCGTTCGAGAACGTGAGGGAAGTAGCACCACAAGAGAATAATATCGTAGTAGATTGTAATATGATGGTCGATGAAAATAATAACAGTAACTCATGTGGGATAGAGGAATGGGTTGCAAATATGGAAGAGGAAAGTTTGTTTTCACCAAATCCATGTTTATTTGGGAGTCATTTCAATTGGGATGATCATGTGGAAAGTGATGTTGAGGTGTCCTTGTGGAATTATACTATTTGA

>Solyc12g056590.2.1 (SlERF12-12)

ATGTGCTTTTTAAAGGTGGCGAATTCAAGAAAATCAAGTGAATTTGTTAGATTTACAGATACAGATGATACACAAACCACCGCCGTGACTGCGATCGGCGGCGGTGTTGAAGGCGGTGGTCAGTTTGATTATTCGATGTATTCAGGGTATTGTGATTCTCAGGCGAGAGATATGTCGGAGATGGTGACGGAGTTTACACGTGTGGTATCGGGTCAGGATTATCGACCCGATACCAGATGTTATTCGGTTAATTCACCGTCTCCGGCTTATTCTTCGTCCAGCTCGGGTTCTAGAGCTGGACTGAAGAGAAGCCGTGATCAACAAGAATTTGGAACTGGGTTGTCATCTTCTTCCTCTGTTAAAATTGAAGAAGCTACAAGTATGGTTGCACCAATTCCCGCTTTCACAACCACAATCACAACCACGACCACAACAGGTGAGGGTTCGAGCGAAGAAACAGGAGGAGATAGGAGGAGGAAATACAGAGGTGTACGACAACGACCATGGGGTAAATGGGCAGCGGAAATAAGAGATCCACATAAAGCCGCCAGAGTTTGGTTAGGAACATTCGATACAGCAGAAGCTGCAGCAAGAGCATATGATGAAGCTGCATTGAGATTTCGAGGAAACAGAGCAAAACTCAACTTCCCTGAGAACGCCAGATTGTCATCGTTACCACAAACACAAAATACTGTAACGTCAACAATCTCCAATCCATCCCCTCTAATAGCTCAACCAACGTCGTTCCTCAATCCTATCCAGAGTTCAGATACAACAAGAGACTACTGGGAATACTCACAATTGTTGCAAAATCCAGGAGATTTTACGGATCAACAACCATCAAACTTATTGGAACAAATGTTCGTTGCCTCATCGATGGCAATGTTGCATTCAAACACATTGCCATTAATATCTTCGTCTTCATCGTTAGCTACATCAGCAACTTCTTCAACGTCATATCCCCTGTTATTTTCGAGTTATTACACACCACAAACTAATCAAATTCAAGGAACCAACACAAGTAGCACCAGCACCACTAGCAGCTCAAGTTTTTCTACAACATTTTGGAGTAGCTCTAGCCAATATCCTCCATCTTCTAGTTAA

>Solyc12g056980.1.1 (SlERF12-13_DREB)

ATGGCTACTTCTACTATGGATTTTTGGACTACTACTCTTTTAGATCTTAACTCATCAAATTCTGGTGGTGAGCTTATGGAAGCACTTGCACCTTTTATTAAAAGTGCTTCTTCTCCTTCTCCTTCTCCTTCTGTTTCTCCGTCTTTTGATTTACAATCTTCTTCTTTATCAACTTCATTTCTTTACGAATCATTTTCTTCAACGAGTCAACCCAATATGAGCTCAATTGGGCTGAACCAAGCCCAAATCTATCTATCGCAGCAAGTTATGCCAGCTGTCACTTTCCAAAATAATAATCAGTATGCTTCTTACTTGGGGCCAAAGCCCGTTTCCATGAAGCAAACGGGCTCGCCCCCAAAACCCCCAAAGCTATACCGCGGTGTTAGACAACGCCATTGGGGGAAATGGGTGGCAGAGATTCGATTGCCTAAAAACAGGACCCGGCTTTGGCTAGGTACATTTGATACTGCTGAGGAGGCCGCCTTGGCATATGACAAGGCGGCTTATAAACTCAGAGGCGAGTTTGCTCGTTTGAATTTCCCCCATCTCCGTCATAACGGATCATTAATTGGGAGTGAATTCGGTGAGTATAAGCCGCTTCACTCCTCAGTTAATGCTAAGTTACAAGCCATTTGTCAAGACTTGGCACAAGGAAAGAGCATTGATACTAAAAAGAAGCGAAAAGTGTCTTCTAAGGCGATGATGGTGGAGGTGGAGGAGAAAGAATATAAGAAAAGCAAGACAACGGCAGAAGCTGGGTCGGAAAGTGATGGATCCGGATCAGGGTCAGGATCAGGATCTGGTTCTGGATCATCACCAATTTCGGAATATACTTTCGATTCAATTTGGGACATGTGTTCAGAAAATTATGTGTTGCACAAGGATCCATCTCAAGAGATTTTTAATTGGGCTTCACTACTATAA
