# Supplementary material for: Function Analysis of the ERF and DREB Subfamilies in Tomato Fruit Development and Ripening
Source: Front Plant Sci. 2022 Mar 4;13:849048. doi: 10.3389/fpls.2022.849048 (PMC8931701; doi:10.3389/fpls.2022.849048)
Supplement: Supplementary file 5 [file Table_5.DOCX]

**Supplementary Table S5. The 14^th^, 19^th^ amino acids and EAR motif statistic of 60 DREB subfamily genes**

| Gene name | Gene ID | Subfamily | EAR motif |
| --- | --- | --- | --- |
| *SlERF1-3* | Solyc01g009440 | DREB (VE) |  |
| *SlERF2-10* | Solyc02g093130 | DREB (VE) | DLNxxP |
| *SlERF3-6* | Solyc03g026270 | DREB (VE) |  |
| *SlERF3-7* | Solyc03g026280 | DREB (VE) |  |
| *SlERF3-20* | Solyc03g120840 | DREB (VE) |  |
| *SlERF3-22* | Solyc03g124110 | DREB (VE) |  |
| *SlERF4-4* | Solyc04g050750 | DREB (VE) |  |
| *SlERF4-10* | Solyc04g078640 | DREB (VE) | DLNxxP |
| *SlERF4-11* | Solyc04g080910 | DREB (VE) | DLNxxP |
| *SlERF5-5* | Solyc05g050830 | DREB (VE) |  |
| *SlERF5-11* | Solyc05g052410 | DREB (VE) |  |
| *SlERF6-1* | Solyc06g035700 | DREB (VE) |  |
| *SlERF6-2* | Solyc06g050520 | DREB (VE) |  |
| *SlERF6-8* | Solyc06g066540 | DREB (VE) |  |
| *SlERF8-2* | Solyc08g007820 | DREB (VE) |  |
| *SlERF8-3* | Solyc08g007830 | DREB (VE) |  |
| *SlERF8-4* | Solyc08g007840 | DREB (VE) |  |
| *SlERF8-6* | Solyc08g066660 | DREB (VE) |  |
| *SlERF8-10* | Solyc08g078410 | DREB (VE) |  |
| *SlERF8-11* | Solyc08g078420 | DREB (VE) |  |
| *SlERF8-12* | Solyc08g080290 | DREB (VE) |  |
| *SlERF9-1* | Solyc09g009240 | DREB (VE) | DLNxxP |
| *SlERF10-4* | Solyc10g076370 | DREB (VE) |  |
| *SlERF10-5* | Solyc10g076380 | DREB (VE) |  |
| *SlERF10-7* | Solyc10g080310 | DREB (VE) |  |
| *SlERF10-9* | Solyc10g083560 | DREB (VE) |  |
| *SlERF11-5* | Solyc11g042560 | DREB (VE) |  |
| *SlERF11-6* | Solyc11g042580 | DREB (VE) |  |
| *SlERF12-2* | Solyc12g008350 | DREB (VE) |  |
| *SlERF12-11* | Solyc12g056430 | DREB (VE) |  |
| *SlERF1-4* | Solyc01g014720 | DREB (VD) |  |
| *SlERF1-14* | Solyc01g091760 | DREB (VD) |  |
| *SlERF2-2* | Solyc02g067020 | DREB (VD) |  |
| *SlERF3-19* | Solyc03g119800 | DREB (VD) |  |
| *SlERF1-1* | Solyc01g005630 | DREB (VN) |  |
| *SlERF1-13* | Solyc01g090560 | DREB (VQ) |  |
| *SlERF3-13* | Solyc03g114440 | DREB (VQ) |  |
| *SlERF7-1* | Solyc07g042230 | DREB (VQ) |  |
| *SlERF12-9* | Solyc12g044390 | DREB (VQ) |  |
| *SlERF1-5* | Solyc01g057080 | DREB (VV) |  |
| *SlERF11-4* | Solyc11g012980 | DREB (VA) |  |
| *SlERF3-14* | Solyc03g116610 | DREB (VH) |  |
| *SlERF3-15* | Solyc03g117130 | DREB (VH) |  |
| *SlERF6-4* | Solyc06g053240 | DREB (VH) |  |
| *SlERF6-7* | Solyc06g065820 | DREB (VH) |  |
| *SlERF6-9* | Solyc06g068360 | DREB (VH) |  |
| *SlERF12-4* | Solyc12g009490 | DREB (VH) |  |
| *SlERF4-6* | Solyc04g054910 | DREB (VL) |  |
| *SlERF4-9* | Solyc04g072900 | DREB (VL) |  |
| *SlERF6-5* | Solyc06g054630 | DREB (VL) |  |
| *SlERF7-4* | Solyc07g054220 | DREB (VL) |  |
| *SlERF8-5* | Solyc08g008305 | DREB (VL) |  |
| *SlERF8-14* | Solyc08g082210 | DREB (VL) |  |
| *SlERF9-10* | Solyc09g091950 | DREB (VL) | LxLxL |
| *SlERF12-3* | Solyc12g009240 | DREB (VL) |  |
| *SlERF12-5* | Solyc12g013660 | DREB (VL) |  |
| *SlERF12-13* | Solyc12g056980 | DREB (VL) |  |
| *SlERF10-6* | Solyc10g078610 | DREB (ID) |  |
| *SlERF10-8* | Solyc10g080650 | DREB (ID) |  |
| *SlERF2-5* | Solyc02g077810 | DREB (IV) |  |
