# Supplementary material for: Function Analysis of the ERF and DREB Subfamilies in Tomato Fruit Development and Ripening
Source: Front Plant Sci. 2022 Mar 4;13:849048. doi: 10.3389/fpls.2022.849048 (PMC8931701; doi:10.3389/fpls.2022.849048)
Supplement: Supplementary file 6 [file Table_6.DOCX]

**Supplementary Table S6. Statistic of the 14th and 19th codons of 80 ERF subfamily genes**

| Gene name | Gene ID | Subfamily | EAR motif |
| --- | --- | --- | --- |
| *SlERF1-6* | Solyc01g065980 | ERF (AD) |  |
| *SlERF1-7* | Solyc01g067540 | ERF (AD) |  |
| *SlERF1-8* | Solyc01g090300 | ERF (AD) |  |
| *SlERF1-9* | Solyc01g090310 | ERF (AD) |  |
| *SlERF1-12* | Solyc01g090370 | ERF (AD) |  |
| *SlERF1-16* | Solyc01g108240 | ERF (AD) |  |
| *SlERF2-3* | Solyc02g077360 | ERF (AD) |  |
| *SlERF2-4* | Solyc02g077370 | ERF (AD) |  |
| *SlERF2-6* | Solyc02g077840 | ERF (AD) | LxLxL |
| *SlERF2-7* | Solyc02g090770 | ERF (AD) |  |
| *SlERF2-8* | Solyc02g090790 | ERF (AD) |  |
| *SlERF2-9* | Solyc02g090800 | ERF (AD) |  |
| *SlERF3-1* | Solyc03g005500 | ERF (AD) |  |
| *SlERF3-2* | Solyc03g005510 | ERF (AD) |  |
| *SlERF3-3* | Solyc03g005520 | ERF (AD) |  |
| *SlERF3-4* | Solyc03g006320 | ERF (AD) | LxLxL and DLNxxP |
| *SlERF3-5* | Solyc03g007460 | ERF (AD) |  |
| *SlERF3-8* | Solyc03g093530 | ERF (AD) |  |
| *SlERF3-9* | Solyc03g093540 | ERF (AD) |  |
| *SlERF3-10* | Solyc03g093550 | ERF (AD) |  |
| *SlERF3-11* | Solyc03g093560 | ERF (AD) |  |
| *SlERF3-12* | Solyc03g093610 | ERF (AD) |  |
| *SlERF3-17* | Solyc03g118190 | ERF (AD) |  |
| *SlERF3-18* | Solyc03g119580 | ERF (AD) |  |
| *SlERF3-21* | Solyc03g123500 | ERF (AD) |  |
| *SlERF4-1* | Solyc04g007170 | ERF (AD) | LxLxL |
| *SlERF4-2* | Solyc04g012050 | ERF (AD) |  |
| *SlERF4-3* | Solyc04g014530 | ERF (AD) |  |
| *SlERF4-5* | Solyc04g051360 | ERF (AD) |  |
| *SlERF4-7* | Solyc04g071770 | ERF (AD) |  |
| *SlERF5-1* | Solyc05g009250 | ERF (AD) |  |
| *SlERF5-2* | Solyc05g009450 | ERF (AD) |  |
| *SlERF5-3* | Solyc05g013540 | ERF (AD) |  |
| *SlERF5-4* | Solyc05g050790 | ERF (AD) |  |
| *SlERF5-6* | Solyc05g051180 | ERF (AD) |  |
| *SlERF5-7* | Solyc05g051200 | ERF (AD) |  |
| *SlERF5-8* | Solyc05g052030 | ERF (AD) | LxLxL |
| *SlERF5-9* | Solyc05g052040 | ERF (AD) |  |
| *SlERF5-10* | Solyc05g052050 | ERF (AD) |  |
| *SlERF6-3* | Solyc06g051840 | ERF (AD) |  |
| *SlERF6-6* | Solyc06g063070 | ERF (AD) |  |
| *SlERF6-10* | Solyc06g068830 | ERF (AD) |  |
| *SlERF6-11* | Solyc06g082590 | ERF (AD) |  |
| *SlERF7-2* | Solyc07g049490 | ERF (AD) | DLNxxP |
| *SlERF7-3* | Solyc07g053740 | ERF (AD) | LxLxL and DLNxxP |
| *SlERF7-5* | Solyc07g064890 | ERF (AD) | DLNxxP |
| *SlERF8-1* | Solyc08g007230 | ERF (AD) |  |
| *SlERF8-7* | Solyc08g078170 | ERF (AD) |  |
| *SlERF8-8* | Solyc08g078180 | ERF (AD) |  |
| *SlERF8-9* | Solyc08g078190 | ERF (AD) |  |
| *SlERF8-13* | Solyc08g081960 | ERF (AD) |  |
| *SlERF9-2* | Solyc09g059510 | ERF (AD) |  |
| *SlERF9-3* | Solyc09g066340 | ERF (AD) |  |
| *SlERF9-4* | Solyc09g066350 | ERF (AD) |  |
| *SlERF9-5* | Solyc09g066360 | ERF (AD) |  |
| *SlERF9-6* | Solyc09g075420 | ERF (AD) |  |
| *SlERF9-7* | Solyc09g089910 | ERF (AD) |  |
| *SlERF9-8* | Solyc09g089920 | ERF (AD) |  |
| *SlERF9-9* | Solyc09g089930 | ERF (AD) |  |
| *SlERF10-1* | Solyc10g006130 | ERF (AD) | DLNxxP |
| *SlERF10-2* | Solyc10g009110 | ERF (AD) | LxLxL and DLNxxP |
| *SlERF10-3* | Solyc10g050970 | ERF (AD) |  |
| *SlERF11-1* | Solyc11g006050 | ERF (AD) |  |
| *SlERF11-2* | Solyc11g011740 | ERF (AD) |  |
| *SlERF11-3* | Solyc11g011750 | ERF (AD) |  |
| *SlERF11-7* | Solyc11g045680 | ERF (AD) |  |
| *SlERF11-8* | Solyc11g045690 | ERF (AD) |  |
| *SlERF12-8* | Solyc12g042210 | ERF (AD) |  |
| *SlERF12-10* | Solyc12g049560 | ERF (AD) |  |
| *SlERF12-12* | Solyc12g056590 | ERF (AD) |  |
| *SlERF1-2* | Solyc01g008880 | ERF (AY) |  |
| *SlERF4-8* | Solyc04g072300 | ERF (AN) |  |
| *SlERF1-10* | Solyc01g090320 | ERF (TD) |  |
| *SlERF1-11* | Solyc01g090340 | ERF (SD) |  |
| *SlERF1-15* | Solyc01g095500 | ERF (SD) |  |
| *SlERF3-16* | Solyc03g117230 | ERF (SD) | LxLxL |
| *SlERF12-1* | Solyc12g005960 | ERF (SD) | DLNxxP |
| *SlERF2-1* | Solyc02g030210 | ERF (ED) |  |
| *SlERF12-6* | Solyc12g038440 | ERF (GN) |  |
| *SlERF12-7* | Solyc12g038450 | ERF (GN) |  |
