# Supplementary material for: Function Analysis of the ERF and DREB Subfamilies in Tomato Fruit Development and Ripening
Source: Front Plant Sci. 2022 Mar 4;13:849048. doi: 10.3389/fpls.2022.849048 (PMC8931701; doi:10.3389/fpls.2022.849048)
Supplement: Supplementary file 8 [file Table_8.DOCX]

**Supplementary Table S8. The ARF, DRE/CRT, and GCC box analysis of 15 DREB and 21 ERF subfamily gene promoters**

>SlERF8-2

TATCACAAATTCTATAAGCTAAGAAAATAAATATATTGTTATAGACTAACACGTGTCTATAATGAATAACAATTCGAGTTTATACTATTCAGATGTCAATTCATTAAATACGATAACATCTTCTAATGAAAAAGAAATACTATTATTAGCATCGAGCCGGCCCAAGAAACGAGCCGGAAGGTAGTTGTTTCAGTTGGGATGACGTAGAAAGTGACCTTGAGGTGTCTTTGTGGAGTTATAGTATTTGATACTAACTAGCTACATAGTTACAAATTACAAATACATATTAGTATTTGATAAGAAATAGAAAATGAAATAAATAATACGATCAATAAAAGTGTCTAAAAACAAAGTTTCCCTTGCAAAAGGTAATACTAACAAAAACCATTTTGTTCTAATTATTATATATTTATATTTGTAACAGTAAAACTCGTGGTAGAGATGAGTCAACTATTAAAAGCTTATAAAAAAGTGATGCTTATAATATATAAATTCATGACATAAAGATGCCTTACGTAGGTATTGCATATGTCATCTTCTTTTTTCTTGTTCTTCCATTTCTCATGTTGCAAAGAAAATTACTAAGAAAATACCATTACAAGAAAATAAGCAATAAAATTCATCGGTATTTTTGAGTTGATCAATAGTAAATGGGAGTTCGGAGTTTAAAGGGTACAAAATATTAATAAAAATTTCAAAATGGTATATAAAATTTTATATGTCAAAAAGGCAAAATCGCTGAAACAACAGCGATTTACTCCACCGAAAAAAGCAAAATCGCTGCCACTGTAGCGATTTTGCAAAATACAATTTTTTTTAAAAAAATTAAAATCACTACTTAGGCAGCAATTTTTATAATTTAAAATATTTTTTTAAAAAATAAAATCACTGTCTAGACAGCGATATAAAAAATTTTTAATTTTAGATTTTGTGAAAAAGTTGTGTTGATTCTCTCCTGTTTCACGTTTGTCTTAGGATAGATAGCTCTATCAAGCTAGCTAGTCTGACTAAAGCTCCACTAATTAATAGACATATGTCATTTTCTAAATAATAACGATTTAAATTTTTTTAAAAACTTTTTTTAAAAAAAATCGCTGCCTAAGTAACGATTGCAATTTTGGTTTTTTTTAAAAAACACATTTTGCAAAATTGCTGCAACTGCAGCTATTTTGCTTTTTCCGGTGGAGTAAATCGCTGTTGTTCCAGCGATTTTGCCGCTTTGGTTAATATAAAATTTTATATATCGTCTTGAAATTTTTATTAATATTTTGTACCATTTAGACTCCGGTCTCTAATAAATGGCCATATGTAGATAAATATTTACTCTCACATGGTGTTTATACAAAATTAATTATAATAATTAATTAATCTTTAACAGTAAAACACATGCTTAATGGAACATAAATTCTCCAAAACTTGAAATTACATTGAGATATAACCTATGAAATATTTATAAACTTATACACGATTAAAAGTCATTTGTTACTTGAAATGTAATGACCCTCTTAGTCGTTTTATGAATTTCAATAATTATTTTTATATTAACCTTTTTAGTAGCGTTTATAAATAATTTATAACTTATAAAAATGAGTAACATGATTTTTATGAGATCTAATAAAATTTTTTTATTACTTATAATAATGGAAAAGAAAAATAAATAAAAGTTCAATAATAGATAAATAAAGTCAAAAAAAAAAAGAGGGGAAAAGGGAGGAGAAAAACCGAGATTTTCATCCCAATTAGGCGTTAAGGTAATGTTTCTGATCTTTTCCATTAAATTTTTATGTGGTTATGAACAGATTTTTAGGTTAAAAACGAGTATAATTTATGTTAAATTAAGAAGTGAACAAACAAAAAAAATTACACTATAGAAAAATGAGACCAAAAAAGGGAAAGAGAGGAGAAAAATTGAGATTTTCATCTCAAAGGCGTTAAGGTAATGATTCTCGTCTTTTCCATTAAATTTTATGTAGGTATAAACATATTTTTAAGTTAAAAATGCATCTAATTAATGCTAAATTAAGAAAATATAGGTCTAGGGTTCTTACAACAAAATTTTCATTTGGGGGTCGTATAACAATCTGTTTTGGCTGAACTTTTAGCATGCGAGTTTAATTTATCATGGGTAAACATGGTCTAAAAGAAAATTTCAGCCTCGAATTTGATGACTCGGGATGACCTTATTTGATTTTCGAGCTGAAACTAAATATAGTAATATGAGTGTCATTAGATTTTTATTCTTATGAGGTTCATATATTTGATAGATTGGAAGCACTTTGAAATTTTATGAAAGACGAAGACTCAAGTCCCGAAGTGATTGCTCAATTATTTGAGCAGATCTCTAGACCCTTAAGTGCATAGAATCATGTATTTTCCTGTGTGATGTGTTGAGGGTAGCTTGAAAGGTTATTGATTAATTGTAGATGTTGTATCACGATTGTATCGTTGTGAATTGTGCATTGTTATGAAAATGACCATCTCTTCATTATTTGTGGACGTGTCACCTGCAATATTTTTGAGACATGATTATGATAGATGTTACGTGAATTGAGGGAAAATAGGAAATTAAAGAGGATGCACTATTTTTTTGAAGGACGTGTTGCATGCCGCGACAGATATTGTAATTGGAGGGACGTATCGCACGCCGTGACTGATACTATTATTGAGGGACATGTCGCAACGAATTTATTATCGAGTTTTGTATCACACACCGCGACGAATGCATGAACAAATATGTCCCCATGGGTCCCGAACTAAGAGACAACGGATGTAGATCATTTGATCAGACATGCATTACGATACTTGACATTGCATTGCATATCTTTATCATTGGTGAATTTGATATTGTGTGTTTTGATCTTGTGAGTTCCTTTATGTGGAACTTGTGATTGATGAATATTGAGCTTGTTGTTGAGGATATAAAATTGTTAGAGTGTTGTTGTTGAGATGTGATATATGTAATAATAACTGTTAGGTTAGGCAAGATTCTTATACAGGTTAGTTGTGGAGGTTCGGTTGGATGTAAGTAGTACCCGTATTCTATTCCCTTAGCTTGTGTTTAGAAGTTTACTTGCAAAGTATCATGTGGTTTGGTACGCACCCCTTGCTTCTAATTTTTTGTCGATTACAAGTCCAGATCTTCGTGATACTTTCTATTCTCCTCTTTTCTTAGATTTCATGTGGGAGATTTGTGAGATAGTTGTTTGTCATCTCAGCAAATCTTCTTACACTTATTTGTGATCTTGTTCAATTTTGGAAATAATATCATTTAAGACTAGTAGTTTTTCTTTTGAATCAATTGTAACCAATTAAAAATTTGTACACGTGATAATCAAATTTCAAGAGTTAAAATAAATTGACATTGTATTCATCTTTAGTATTCGTGTTAAATTTTCATCCTTATTAACTTTCACATATTATCGTTATATTTAAATTTAAATAAACTTGTTTTAATAAAATAAGACGAGTGTCATCAATTCTCACCGAATGTGTGGCACCCGTGTGTTCTTTTTCCTGTGAACACAGTGTTTATGTCCCATTGACCCAAAATTGGAAAGGGAAGTTCCACCCACCCCACACCCAACTCCCATACCCCACAATTTTTATGGAAAAATACAAGAACCCCATTTTTAAATCTATCTCCAAAACTTTGCCAACTCACTACTTCAAGTCTTCAACCAATAAAACTACTAGTATATTCTTCTTCAGTTTTTCTATTTAAAGGATGATACACAAATATTTTTTTAATTTATTACAAAATGTCAGAGATATATTTATACTATATTAAGGTCCTATTATTTTTTAACATATTTTATTAATAATTTTCTAACTCTTTTCGGCCTACTTGATACTATCTTGTGGGGTCAATATTGATTGATTTTTTTTTCAAGCTAGTGCCACGTAAGCCAAAAAAGGATAGAAAATTAATTATAAAATAAGTTCAGGAGGGTAATAAAACCTAGTATAGTATAAATATGTCTCTGAGATTTCGAGCATAGATTGAAGGGCTACTAGTACATTTTTCCTAAATAAAATAATGTAACTTAATTTTGTAAACTTGTTCACATTATCAATTTCAAACTTAGATAAAAGAGTTGAATTATGAATGATCAATTGAAAATATACATATATATATATAGATATTGAGATTTTACTTTTACAATTACACTAGATTTTTGTTGTTTGTTATAATACTAAGCATGTAATACTTCATCTGTATCTGTCAATTTGTGCGTCTTACTTTTTTTTAAAAAAAATTCATCTGAAAGAACAAAAACGCCACCTTCCCCTTTTAGTAGCTATTTAATTCCAACTTTTAAGGTGACATAGTTGAGACAATAAAATTTAAGATAACAAAATTTGAAATATTTTTTAACTTTCTGAAATAGGTAAATTCTTTTATATATGTAAAAAGAGAATGTGAGTAGCTTCGGGTCAGTCGATGAATCAATCAATTTTTATCCTTCTATTTTCTTTTCCACTTTTTCTGTTTATAATTAATATTTTTTCGTTTAAATTTATTTGTCTTACCTTCTCTTTTAAAATATTAAGTATCAACAATTAATAACTTAATACTTTTTAAAAAATTATACTAAAACCTTAATTAACGCTCAATATTTCACATGTGCCACATCATTTGAGACAGAAGAAGTTGAAAAATTACATCAAAAGATTATTATAAATTATAATAATTAATAAGTTAAAATATTTAAAAATTATATTAAGATAACAAACTCTTGAGTTGTATCTAGCAATTGACCCCACGAAGAAAAAGAAAAGTTGTTACTTTCTTTTAAGAAAAATAATTTTGCGTGTCTATGTCGTAGGCACGTGCTTTCACTTTCTTTTATAATTCATAATCCAAAAGTGTATCCAAAATCTATATAAACCACTGTCAAACTTAACCAATATTCTAAACAAACTTACTTCTAGCCAATATCACAACACACTTCAAACTCTAAGAAAATCAGTAGCTTGTTATAGACTGACACGTGTCTAAAATGAATAACGACTCGAGTTTGTACTCATCAGATATCAACCCATTAAGTGCAACAACACCTTCCAATGAGGAAGGAATACTATTATTAGCATCGAGCCAACCCAAGAAACGTGCAGGGAGGAAGAAGTTCAAGGAAACTCGCCACCCGGTTTATAGGGGAGTGAGGAGGAGGAATAATAACAAGTGGGTTTGTGAGCTACGTGAGCCTAGTCAACAGAAAAGAATATGGCTAGGGACTTACTCTACTCCTGAAATGGCGGCTCGAGCTCATGATGTTGCTGCATTAGCTCTTAGAGGCAATCTAGCCACTCTGAACTTTGCTGACTCTCGATGGGGATTGCCAGTGCCGGCATCAAAGGACCCCAAGGACATACGACAGGCAGCAGTTATGGCTGCACAAGCTTTTTCTCAGGATACTGAATTAGTCGGAGTTGACTATATGAATGAGGAAGTTAATTCCAATACCATCGATGAAATAAAGTATCAAGAGATAGATATTGCGAGGGCTAATGATGGGAGTAGTAGTGATTTTGGCGCAAAGGAATTGTGTATAGATATGGAGAACATTTTATGTTGTAACTGGGGAGAAGATAACGACATGTTGGAGATGGAAGGATGGCAAGAAAAGATGGCAAAGGGGCTTTTGTTTTCACCAACTCCGCGTTTAGGTAGTTGTTTCAGTTGGGATGATGTAGAAAGCGACGTGGAGGTGTCTTTGTGGAGTTATAGTATTTGA

>SlERF6-8

TGATACTATATTGACCATTTGATTTCACTGTGTTATGTTCTTGAGCAATCTTCCTCAGTCTATTTTCTACATGTACTTGTCCCCTTTATCAAACGTGATCATTTCTAATTCGTCCAATCTAGTATGATCACACATCCATCAATCTTAGAAGCCCAACATTCCTTCCCACATAATATTAAATGGTTCACAATCATTCGATAGAACTTGACATTCACTTTTTTAGAAACCTCTTAAGTCACTCCTCCATTTGAGCCAGTCTTCTTTGATTGTACCAATACATTTTCTGAATAAATTGATTTACATAATTAACAAAACAAAAAATAGAATAGAAAGATTGATGCTTTATATTTTAGTTATAACATCTATTCCTACAAGATATGTGTAGAGAAGTGAATTTGGGCTAGCTCAAGAATGGAACGATTGCTGATTTCAGTGTCTCGACTTAAAATTCCCATTCCCTTATCTCACGGAACAAGAACAAATCCACAAATATACTCCACCATTAACTAATGTTTATTAATTCCCCAAACACAGACTGGGCATTCACTCCCCCGCCCCCAAATGAATCTGATGTACTAAAAGGCAAAAGCTTCAACAACAAATCGACATTACTCGATTAAGAAATACAAAGGAGCAAATCGTTCGCGTTGACTCTTCCCGATTTGAGGATTCAACAATTCGATGCATACAAGTAAAGATAAAAGAGTAAATTTTAGGAGAGAACTTACTGATTGTGTAAACTGGAAATTCGGAGAGCGAAAAGGGAAGCCATTGAAAAGGCGAGGAAAAAGGAAAGAAGAAAATGGATAGGGGTGAAAAGGGAGTATTTAAAAGCGAAGCCATCGAGGCAGAGAGAGAAATAAATCTGCAAATTGATATTATTTGGCAGTTCCATACCTAAATCCAACTTGGGATTTAGCTCCAACCAGCTACCCCTAAATTCAATTTAGTTTGTTACGAGGAAAATCGACGGTATCACTGATTAAGAAGTGGCACAAAAAAAAAAATTAAACGAAATTGAAGTTAAAAAAAAAATTAAATTTATATTAAAAAGTCTACATTTTTATATGAATGAGTTAGGAAAGGAAAAGTACATCATAATCTTTGTTAGTGGGCAACTATGAATTGCTATATTTTATTTTTTTGTCCTAATTTATGTCATATGCTTTTTAATAAGACAGAGAATATTGTTTTTTAGAAAGTTAAAGAAAATTATGACCAAAAATAAATTAAAGATGCGTGTGGGTTACAAATTATCATATAAAAAGGTAAAATAAAATAGTTTATTTTAAAAAAGTGAAGTGCATCACATCACAAAAGTATAATAATTTTTAAATAAATTTATGAATATAGTAAATTTTATGTAATTGTTACGTATGAAACTTATATTTTAAGAGGTTTAAAATACTTGAGTTACGTAGTGAAATTTAAATATCATGATATTTATGTTATGAATGTAATAATTTAAAGAATGCGCACCTTTCATGAAGAAAAAGGGTTATTTGTATTTAGAGTGAGCTCGTGAATTATAAGTGAAGAGAAAGTGTAAAAAAGACGAGCAATTATATGGTGCTCAAAACTTAAATGTGCAAGAAAGACTCACATTTTCAAAACTGCTAATGATTTAGATTAATTTCTAGTGTTGATGGTGCTCACGACAAGTTATATAGTAGAAAAATAAAAACAAACATATCGTGAATCATGATCATTAATATTATAACACAATTATTGAAACGGACTTGTGACAATTGACAAATCAAGATAAAAGGTGATTCTTTTTTTAAAAAAAGATAAAAAGTGATTTATTACAAAAGATAATAATTTATTGAAAATTATGTATTGATATTTTTTCTTATTTTTAAGTGTTTAATACTATCTCAAAAATATTTATATGGTACTTTTAAGTAACTTTTGGGTGGTGGGTGGGGGATCAATGTACAAAATATGTATACTGGCTAAGAAAATAAACAGTGAATTCAGTTGGCTATTTGCGTAAAGATCCTTTAGTGATTCCTAGAGGATTTCGGCCTATTTTCTCTTTGGGCCTTTTTAACTATGAAAATGAAGCGTCTATTTTGGGTCCAGCTTCTAAGGCCTTTTCTAGTAGCCCAAAATTCCTTAATCCTTTTACCCATCTTTTATTATGGAAAATTATGCGATTAAGTAAATTTATACTATTAAATTACTCATCATAGTTATAATTTATTATAATTATTATTCACGACTAATATAATGCATTAATTATGTGGACTAACTTCGAGTTTGTATAATTAGTCACGCTTGTATATGTATAATTCGCCAGAATATACAAATACATATGTATAATATACAATTATCTACTAATATATATATACAATTCATCTCTCTCCCACTATTTGTCCTCTCTCTCTCGTATCTCTCAATCTCGCTCGCCTCCCTCCTTTCTCTTCCAATCCCGCTCTTCTCTCTCCTCCCTCTCCCAATCTCACTTGCCATATATACAAATGCATATGTATAATATACAATCCACCTCTCTCCCACTCTATGCCCTCTTTCGCTCGCCTCTCTTCTCCCTCTCCCATTCTCGCTCGCCTTCTCCTCCCCCTCCCAGTCTCACTCGCTTCTCTCCTCCCTCTCAATTTGCTTGCCATATATGCATATACATATGTATAATATATAATTATCTAACCGATATACATATATAATTCACCTTCGTTCATCTTTTTTTTGTCTCTCTCCTCTCTCCTCTCTCTTCCAATTTTACTCGCCTCTTATCATCCATATAACATTTACCTACGAATTATATTATCATATTATAACTATAGTTAATAATTAAGATATTTTTAAATGGCTATATATGAAAGTTGTCCATTTATTTTGCCAGCCCTTACCTCATCAATTGTTAACAAAATGATTATGACTTAGGCATTAGTATTATAATGCCTGTTGGTTGGTGTAAGGCCGTTTGGATTAATACATTCAAGACAAGTGTCACTTTTCTCGAAGTATCTTGCTACTGAAAAAATAAACAAGTTTTTGTGCTGTGACTATCACAAGAATGTCATAAGTAGAATAGGGAAAAAGAGAGGATTGTTACAGAAATGATGAACCTGGTCCAAAATTAGTATTTTTAACTGTACTAGTACTGCACTTTATTACTCTATCTCCTTTTTGTAGGGTTTGAACAAAAATACGAGGTAATATTTGAAGTTAAGTTTAAAATTGTATTTGAATATATATTAGTTTTAAAAAGTAAAAAATAATTTTATGAAATGAGTCAAAATTACTTTAAAATTAGTAACTCATCTTCCATTTTCCTTTTCAAAAAATCAGTGCAATTTATGCACACGCAATTTTGCCAAACGGCTACTTATTAGTTTCATTAATCGTAAGTCAACTAAACATTCATCCGTCACTAACTGTGACGTGCTATTTAATTTTTAAACAAATCAAAGATATATGTTGTAAAATGATGTACACGGAAAACTGCGAAACTATTAGCTATTTTGATATTTATAGATTCGAACGGTGAAAATAATATCTTTTAAAATATAAAATATTATATTGCATACAGTAAATTCCCTTTGTGATCCAATTATTTTTCCCGTAGTTAATGCCTTTTAAATGTTGTTAAAGAAAATCGTCTTTTCATTTTCACAAAATAAGAAAACAATATGCATGCTTATATATTCTATCCTCTCATATTGGACTAGTGAAATTATATTTAATATTTTATTATCATTATTCGTTTAGAATAAATAAATGAGGCTTATTTATTTTTAAATTAGAGAAAGTAGGAGGTGGCCTTTCACCAATATAAGGAGATGGGTCATGTTGTGATTTTAAAGTGGAGCACGTGTTAAGGGACAAAGGTATAATGGTTGCATCCCATGTATGTACAAATGAAAGAAGAGTACGTGGATGATTTTAATTTACAAGCTATAAATAACTACATTTAAGAACATGGACTTTTATTGAGCTTTTTTGGGACCCTTTTTAGCATGATAGTGACTATACCTTCTTGTTGGTTAGTTTTCTAAAATAATGCTTTGGGACTTTGGAAGGTTTTCATCTTCACTAATGGAAGAAATAATGCAGTATGGAAAAAAAAATATTCCATCAATTTCATTATTTTTAAATTAAAAATAGCAATGCTATTTTTTTCATGTTTTATTGTTAATGACGATAAAAAGTTCATATATTTTATTTGAAATTACAAAATATGAACTTTTTATTTCTCTTAAACTCGTTGTCCAGAAAAATTCTGGTATTTAAAATGAAATATATGGAGTATATTACTTGGTTAAATTAAATGTAGTTATGATAATCAGTTATTCATAAAAAAGTGAGATGAACTAGACAAAAAAACCATGATAGGTTACCTATTCAAATACGCTAATAAATTTAGAAGTACTTTACAGTTTACACAATCAATATGTATATTTTAAACTACTTTAAATCATTTTTGTTAGTATTAAAGATGCATTAGTAGTGGTTGGTTAATCGTCAATTCGTCATCCATATTTTTAATAGAAATAAAGTGTTATTTTAACGTTCAAAGTAAATCGTACGACAAATTTAATAACCTAAACCTAATATTTTACGTATTGTTTGTCTATATTACAGACGTTCTTTGTAATTTGACAAGTAATAATTCTCTACACCTGACATATATAAATATATATATTAAGCTTGTTCAGAATGTATGATAATGAAGTCCATTTCTCTCTGAGAAAAACACACTCCAATTTCCTCTCTCCCAAAAATACTTCAATTTTTGTCTTTTTCCATGTTTCATTCCCATTCTTCTCTAAAGTAGAAATTGACACATTTGATTATTTTTTTCTCCCCTGTTTTCATAAAGGAACTCTTCGATCCTAATATATTAATACAGTATGTCAGAATTCAATTGATCATAAATATTTCTCTTTCTTCTCAACATCCATCTTATAATAATTCGTATTTATAATTATAAGATTAAAGTTAAGGTATTTACTATTCGAGGAAATTATACTAATTAAATGTCAAAGCGAATAAGAGAGAGTGATGAAAAAGGTAATAAACACCCAATTTATAGAGGAGTTCGTATGCGAAGTTGGGGAAAATGGGTGTCTGAAATTCGCGAACCGCGTAAAAAGTCACGCATTTGGCTCGGGACTTATCCAACTGCAGAAATGGCGGCTAGAGCACACGACGTCGCTGCATTGAGCATAAAAAAGGACTCATCGATTTTAAATTTCCCTCACCTAATTGACTCATTGCCTCGCCCAATTTCACTCTCACCGAGAGATGTTCAAGCTGCTGCAGCTGAAGCAGCTGCAATGGAGGATCTAAATTATGTCTCTTCTACATCCTCTGTTAGTTCAATTGACAAAATCACGTCGGCGTCGGAGGAACTGGGTGAAATTATAGAGCTTCCAAGCTTGGACGGAAGTTTTGAGTCGGAAGAATCGAAGACCGAATTGAAGATCAGTGATTCAGTGGACGGGTGGCTGTACCCACCGTGGTGGGCATCAGATGGCGACTTTGATGGGTATCTTTTTGAGCAAGATGCTGTTGGAAACAGTTTAATTCTTAGCAACTTTGAAATGATGAAATAA

>SlERF12-9

TTCTATTCTTGTCATCCCAAAACCCGTAACCAAACAAATCAGAACCATATCCTATGAAGTAACACTTCACAGCCTTGGCGTCAAGCTTGTCTCTCTTTTCTGGATCAACATGAAGATAAGCAGTGCAACCAAAAGTCCTCAAGTGTGAGTACTTGAGCTCTTTTCCTGTCCACACCTCCTCTGGAATCTTGAACCCTAAAGGAACTGATGGTCCCCTGTTGATCAAGTTTGCAGTTGTGCTCACAGCATCCGCCCAAAGTGTTTTGGGCAGCCCACAATGTATCCTCATACTCCTTGCACGCTCATTCAATGTTCTGTTCATCCTCTCAGCAATTCCATTCTGCCTTGCCTTACCAGGAACTGTTCTCATCAATCTGATTCCCTCAGCTGCACAGAATGCCTTGAACTCTGATTTGTCATACTCTCCTCCATTGTCAGACCTTAGACATTTGACTTTCAAACCGGTCTGATTCTCAACTTCAGCTTTCCACTTCTTGAAAGTTGCAAACACATCTGACTTATGCTTCAAGAAGTAAACTCATACCTTCCTGCTAAAATCATCAATGAAGGTAACATAGAATTTGGATCCTCCAAGTGATGATACTGGAGATGGTCCCCAAGCATCTGTATAAACCATTTCTAACCGCACTTTCTTTGGCTCTCTAGGAGTCTTTGTGAAGCTTACCCTTTTCTGTTTGCCCATAACACAGCTCTCGCAAAGACCCATATCAACAGACTTCAGACCCTCTAATGCTCCTTTTGCAACCAGCATCATCATTCGTTTAGTACTTATGTGTCCAAGTCTGTTGTGCCACAGACATGAACCGGAAGCACCTTCAGCAACAACGACCATGTTTATACACCCTGCAGTGGTGTACAAGGTTCCAGATTTGGTGCCACGAGCTACTACCATAGCACCTTTCACGATCTTCCACGAACCTTTCCCAAACTCTGCTGCATATCCCGTACTATCCAACTGACCAACAAAGATCAGCTTTTTCTTGATCCCAGGAATATATCTGACATCCTCCAATGTCCACTGGTTTCCCGAGGTGGTCTTTATGCAAACATCCCCCTTTCCTTCAATCTCTAAGGCTTTATTGTCAGCAAGATATACTTTTCCAAAATTTCCAGATTTAAAATTTTGGAACAACTCCTTGCTTGTAGACGAATGGAAAGATGCACCAGAATCCAAAATCCATGATTCAACCGGGCTGTTCACACTAAGGATTAGAGCATCCCCAATGTCCTCTGCTGAATTTACAGAATCACATCTCAAATTTATGATTCTGTTTCTTCTTTGGCTTTGTACAGTTCGTCCGAAAGTGTTCTTTTTCTCCACAGTTCCAACAAGTCACGTTTGATCTGTTCAGTGATTTTACTCGATTCTTTGATTTTGATCGACCATGTTGATTTTGGCCTTTCGTCTTACTTCTCCCCCTTCGATCAACGCTGAGAGCACTGCCCGATGAATCTCCCGCTTCTCGTTTGCGAATACTTTCGCTAAGAACAACATCATGGATTTCATCAAACTTCAGTTTCTTAGATCCACGGGAACTGGTAATCGCAGCAACAACAGTATCCCAAGACTCGGGCAGAGATGACATCAAAATCAACGGCTTAATTTCATCTTCGAAATTAATATCCACAGAATTGAGTTGACTCACAATCATATTGAACTGGTTTATATGATCAGAAATGGATCCATTCTCAGACATCTGTAAATTGAACAATCTACGCATTAAATATACCTTGTTCATAGCCGATGGTTTTTCATACATGTTTGACAGTGCCTTCAACAGACCAGACGTAGTCTTCTCCTTCACGATGTTGAACGCCACGTTTCTTGACAAAGTCAACCGGATCAACCCTAGAGCCTGCCGATCCTTGAGTTTCCACTTCTCCTCCGTCATGGATTCCGGCTTCACCCCGGTCCGGCATCTTCCAGAAACTGAAATCGGATCCATCAAACTTCTCGATTCCAAGCTTTGAACTATCCATCTTCAGTGATCGTGTTGAATCTCCTTAGCTCTGATACCAGTTGTTAGGATCGAATTCACGCACACACACTAGATGAATGAAGAACACAAGAACTTTCAAGAGAAAGAGATGAGAGATCTAGAGAGAGAAAGAGAAAACCCAATATTTCGTGGTAAAATCCCGTGAAGAAACTTCCACGGTGGTGAGGTATATTTATATTAATAAATCAGAGTATTTTTGGTTACAGAGAATAAATAGGAAAACCTAAAATAGAGAATAAATAGGAAAACCTAAAATAAATCAGGCTCCACTACCTAACACTATGTCCCCTCTTACTGAAATCTCGCTCACCACTCTCTTTTTTCGGTCATCTCTCCCTCGCCTCTCTCATCTCCCTTCCTAGTCTCTCTCTCGCTTCTCTTCTACCAATCTCTCTTACTTTATACAAATACAAATGCATAATTTATGTTTGTGTTTGTATAAAGCGAAAGAGATGGGATATACAAATACATACTTATCTCTCTGCCTTGTATACTTATAATTATAAAAATATATTTTTTTCCTTTTCTAATTCTCTTTTGCCTTTCTCTCTCTGTTGCGCTCTATACAAACGTAAATTATACAAATACAATGTATATTTGTATTTGTATAAAACAACAGAGATTGGATATACAAATTCAAATGTTTTAGTTCGATTCAATTGTGTACGAAATCAAATTGTGTACAAATATATAAATACAATAATTGATACATATACATATTAGTTACATATATATAATTATCTAACTGATATACAGATACAATTTAGCTCTATCTACTCTCTACCCTCCCCCCCTTCCCTAATCACGCTCACAAAAGTTAAAAATAACTTAAAATACGTTTAATATGACTTATAGAAGTTAACAATGGTACTCCTTTACTTCTCATATTTTTAACCTAACAAAGGCTTAAGTTTGACTTTTTTAGAAACTATTTTTTAACCTTTGACTCTTTTAAAACCTATTATAGTTAACCTCTCACATCTCCTTAATTTATGATATATTCGAGCACCTTCTCTTCACATGCTCAAACTAGCGCTCAACCTCACTTTCCTCATCTCCATTTTTCTAAACATGTTCGTTTGTTTGATCAACACCTTATCTCATACAATACAATAAGTCTAATCATTTATATTTAAATCTTCGTAACATATTTTTATCATATATTATCTCAACTGTATACCTCCATTTCAATACGATATGTAACATCATTATCTATCTCCTTATTTTCTCAAATTATAAACTCAAAACTATTTGAAATTTCCTCAATTGAATATCACTAATGTATCAATCTTTAATTCCACCTATGTTCACACGTTACATCACTAATATAACGGAAAATCATTAATATAGGAACACTGAGCAATCTCTAACGAACACATAAACGAAAATACGTGGATGATTATGATATTGAGCTGAAAAAGACCACAGTAAACGTATGTGTTCAGAAATATAACACCTTTTTAATTAGCTAAATCTACCATTATTTGTAAGTAATTTAAATAAGAAATGTTATTCTACTCATTAATCTCTCAAATATTGAAATACAATGATAGTACTACTTGATAATTTATTTTACTTATGAATAAATATTATTTTAAAGCTTTAATTTCATTTCCAAAATTAATTTTCACTTTATGTTGTTTTCGAGCAACGTAAGCTCATTAAATATACTTTGTTTTTTTGTTTTTTTTTGCTAATTTTTACTATTTTATTTATTATACACATAAAAAAGAAACTTTTATGTGAATAGGTGTCCAATTACACCTCGTGACGTATTTATATAGTGACCTCGTTAGTGAAGTGGCAATGTGATTTTCATCACTTTAATGTGATTTCACTTTTTTATTTTATTGTATCAATGTGTTGTGTTGGTTGGGTAGAAAAAACTTATTCCATGATTGATAATTTATTGTGTGAGGCTATTTAATGTGAGGCAATAGTTTCTTAGGTTGATACACTTTTTCAATCAGAGAGTGAATTTAATTTTTTATTCCTTATACCTAAATATTTTTGTGATTGACTTTTTGGTTTTGACTTGGAAATTTTATTTCAAAATTTTTAACTTATTTTGATTATTTTAACTTAAAATTTGTTATTTGGTAGTGTACTGGTAAAACCAATGCATGCATTGGTTTTATTTATTAGTCAGTGGCAATATCATAATTTTGACCGAGGAGTGTCAAAATATAAAGACATAAAATCGTGTAAAAGTCAAAACGTGTCAATATATATATATATATATATATATATATATATATATATATATATATATATATATATTATCTAACTATACAATGTAATTCTTCGACGAAGGGATATTGTAGGTTACTCCACAACTGCTATTAATAATACCTTGTTTCATATACTCCCTCTGTCCACTTTATTTGTCATGTTGTGTTTTTTTCTAAAGTCAATTTGACTTATTTTTAAAGATAAATTAGATTATGTTAATTTAATAATTTTTTTTAAAAAAAATATATATTTAAAAACTTATGAAAAGTACTATAAATTACAATTTTTTTTGCATATAAATATGATGAAACTATACATCATAAATTATTGGTTAAAGTTTTTATAATTTAACTCTAAAAAGAAAAACATAACAATTAATTATGAATAGAAAAAATATTTTTTATACTAGCATTAATTATACACTCTATTATATGTTAAGATATATATTATTAAAATTATAAATTTCTAAGCATTAATAGCGCAAGTGAATTCAAATACATGCAATAATATGATTAAAGATCCAATTACGTCTTAATTATTTTATTTTTACCTCTTTTAATTTTTTTCGACGAAGAATATGCATCGGACTATCCTTTATGTTTCTCTTTCATTCTCGTTAGCATGGTAATTCGAATTACATGATTTTTCTTCTCTCTTCGTATATATATATATTCCCTACTTTTTTGGCATTCGATACAAACTTCTCTGAGCATCTGTGTAACTGTCCACTTCAATCACCATTAATTAATGGCGGAGCTTGTACAATCATCTTCCATATCCGTGTCAAAAACAGAGGAAAAGGAGAAACGAACAAGAGATGAAACTTACCCAGTATACAGAGGAGTTCGAATGCGAAGTTGGGGAAAATGGGTTTCTGAGATTAGACAACCGCGTAAGAAATCGAGAATTTGGTTGGGTACTTATCCAACACCTGAAATGGCTGCACGAGCTCATGACGTTGCTGCGCTTAGCATCAAAGGGAATTCAGCGATTCTCAATTTTCCTCAACTTGTTGAATCGTTGCCTCGTCCAGCGTCAATTTCACCTAGAGATGTTCAAGCAGCAGCTGCCATGGATGAACTAAATTCCTCTGTTTCTTCGACATTGAGACACAGTGAATCGATGGAAACGGAAGATCAATTA

>SlERF12-2

AGATCGAACGAGTTTGAATTTATTTTGTTACCCCTATTATGTACCTTCCTAGGAAAAGAATTGCGCCTTTTGTTTCATATAATTATGACATTTTTTTCTTTTTTACTTCATCCCAAAAAAAGGTCACTTTATTACAACAATAATTTAATTTTAAAATTCTATATTTATTCTTAATTAAATAATTTGCAAATCACATAGATCTCTAAGTATTGTATTAGATTATAAATTTCAAGTCTTATTATTCTTGAATATTATAGCAAATTAAATAGTATTGTATAAAATAAAACGGAGAGAAAAATATTATGGGAGTCGAAGAATGTCTCATAGACCTTTTTCTTTGAATTTTTAACTGATATTTATGTTTTAATAACAAATCTATTCTCTTCGTACCAAATAGGTGACATTTTTCAAAACATGAGGATCAATTAATGCATTAAATTTTTTTTTATGAAATTTGAAATTAAAGTTATGTGCATAGCAACTTAAATGTATATATTATAGTTAGATCACAAGTTTTTATAAGAAAAATATCTGAAAAAAAAGATAATGTTAAAAAAATATTGGACCATCATTATAATAAATATAAGAAGTTAGAAGAAGAATCATTGACGAGATATGTGGACACTTTTTTTTAATAATTATTCTTGAGAATATAAGTTATAACTTATATACTATAGTTTGATAATATAAATATATTAGTTTAACACTTATGATATGCATATATATATAGGTTAAACTTCTTTAATGTAATGAAATGTAGTATATGAAATACTATGCACCACCAAAAAAATCATTAGTTTTTCAATTGGAAAATGTTTCATAGTCATTCTCATCGATATTGTGATTGAATTAGTAAGAAAAGAAAAAATCATAGTATTTTTACATGAAAAATTAGAAATTTTTTCAGTATTTTAATAATTTTTTTTTTTTTAATAATGCATCTTTTAAGTGATTTAATACGATAAAAAAATATTTTTTATAACATAAATTTCTCACTAATTTAAATATATTTCTAACACTAATGATAGTTAAATTTTTTAGATATGACTTATATAATTGTATTAAATATGTTATTATTATTATTATTATTGTTATTTGAAATTCATGATTGTAAAAATATTCTATTTGTGATATTAAAGCATATAGAAGATGTTTAATATTTTATTTTGGAGTATATAGCTAAAAATAGTATCTCATTCTTTCACATGCCATAGCTACTAAAAAGGCTGACAAAGAGATCTAAAGACAACCTTTGACTTCCCCTAACTATTTTTTTTACTATGTAAGGTGAATATTTTAGTTCTTTCTCCATTTTCCAAGTAACTCAAACATAGCAATATAAAAATCTTTGATGAAAAGGAAAAATAATTACCCCATTTAAATCCAAAGTCAAAACTTTCAATATTTTTTTTTTACCTCAAATATGTAAATGTAAATATCTATTAATAGAGTCTCGAGAGAGGAGGAGAAAGTTATAGTGTTATATTATTTAAAAAAGAATAAAATTATTTCGTTATTAAAGGTTGTTTGATTCAGTGGCGGAGTCAGAATTTTCTTAAAGAGTGTTCAGAAAAAGCAACTTGTTATATTAAGAGTGTTCAAAATAGGTTATTTATTTCTTTTATCATTTTACTTGTATATATGCTAAATTATTTTCGACAAAGAGTGTCTAATTGGACACCCTTGACAGTATATGGCTACGCCACTGATTTGATCGTTAATTAAAATTATATACTAATGCAAATAATATATGAACTAATTAATTGCATAGGGTTTGGTTTTCATTTATAACTTCATTCATAGGTCAAACTTCTAGTGATTCACCTCTGATTTAAGATAAAATAATATATATTTATTGAGATCATACTAAATTAAAATGATCATAATTAAAATTATATTTGGTGCTAAAATTTTATACTGAAACAATTAAAAAGCTACCAAACAAGATAGTTTTATTAGTTATGTTACTTTAATACCTAAATAATTCCTTCTTGTTAAGACTTCTCTTTAATGTTTTAATAATTAAGAAGCTTTTATAGTAATAGTAGCATACTTACAATTACAAAGCTTTTTTCTTTTTTTTAAATTATGTGTTATATAAATTTAAAACGACTGCCGTAAATTAAAAAAATGACAAAAAGAAAAAAGAGAAATTTTGAAATTGAGTTAAATGTGTTTCAAGTATAAATTTATGTGGAAGATAATTTGATCACCATATATGAAACAATAAGAAGTTGTTCGAATTTTAATATAGAATCGCTTTCTGTGAAAAGTCTTTAATAATTCTTTAAAGTAAAATCTTTAAAATTGACTTTAGATTAGTCGAAGTTTAATACAAATATTAAATATCGAGCGGAAATAAAAAAGAAAATAATTAACCAAAAAAAAAAAGATTTCACACCATATGTTTATGGTTAGTGAAGAAAAAAGTGTCACTTAGAAAAAGGGCTTTGACCTTTTTAGATGGTTAAATCTAGGCTTAATCTTCTCCTAGAATTCTAAGCACACCTTAGTATTCTAAATTATTATTTTAATTAATAACATTAATAATAATTAATTCTTGTTCTCACTTTCATGTGTTTTAAAATAATAAATCAAAATCTACCCCCACCTCCTGTGTTTTTAATTAATGCTTGTCATTTAAGTCTCTTTCCCTTTTTGCAAGTGTTTCTGAATTTGAGTATATTATTTATTAATTAAATAAATAATTAATTAATCAAATTTAATAATTAATTAATTAAATTTTTTTCTCTGGTTTTGTGCTCAGTAATTAAGGAATTGATGGGTAAAGGCTTCGGTCGGTTGTGAGGCCTTTTCTTGTCTCCCCTCACTTAAATCTTTTTGTTTGACCATTTGGTATTTAATTATTTATAATTTAATTAATTTAAATATATTTATTATAAATAAAATACTGGAATCTAAAATCGATAATGAAAATAGATAAAAAAAAAATTATATATAAATAATGCTATTTGCATTAATTGTTATGAAATATAGTGAGATTTTTATTTATTCTTTTGAAACATATATCAAAATATATTAATATATCTGATTGAATATGTCACTACTTGAAAAAATAATTAAAATTGACAGATTTTGTTGGGGTTTTTTTTTTAAAAAAAAATACGTCTGTCGATTTTTATTATTTATTTTTAGAAAAAAAATCTTTAGAAAATCAATAGAGTCCCTTTGTTTGTTTAAAATGGAAATTGAGGGTATTTTTGAAGTGGAAAATGCATTTTGAGGATATAAATAAAATTATATTAATTTTTTATTTAAGAAAATTGAGGAAATATATCTTTCGATTTACTTTAATCAACCTGTCATTTTTAACAGTCAACTTCTTAAACTTAGAATCGATTTTAGGTAAAGAAAAAGAAAAGGTAAGATTGTTTTTTTAAAAAAAAAAATTTAGGCCCTCGATTATTTATCTTTTCTGATAGTATATATATATGTATCAATTTATGTATGTGTTTTTAAAAACAAAACTATATATATGTGTGAAAAACAAAACTAAATATATATAGATAATAGAATCTAATATATAAGGCATGCTCTCAAATTGTACATGTGTAACAATAGCCATATATATTGACGTCCAGCTGTACATAAATAGTATGAATAAATAATGTGCTATCATATGTTACACAGTATCATAGTCTATAAGAATTTCACATTATTATCATTAATATAGATGATTTATAAGTTATATAAATATTTATAATTTATTTTAATTATTTTTAAAAAAAATATCAAATTAAATAATAACATATCAATATCAATTGAATGGAGAGAGTATAGTATATAAATGTTAAACTTAACTAGTTAGCACATGCATTCACATTTGTTTCCAATATTCAGTATTGATTTAATATGTTTATTTATTAATATTAAGTAAACTAATAATTTTACTATGACATTTTTTAATAAGAATAATATTAGTATTTTAGAAAATAATTATGATTAATATCGTAGCATACTCAATAAAATCTCATTAAGTGAAATATGAGGAGAGTAGAGTGTATATAGATCTTAACACTATTTCGAAGAAGTAAAGATGTTGTTTTCGAAAGACTCTTGGCTCAAGTACATCAAACTCAAGTAAAAGAAGCACAAAATAATGAAAAAAAGCATAACTATTAATAAGAAGAGTAAAGTCTAAGAGAAAGAAAGAAATAACTATAACAGCAAAATAGTGTGGTAATCGAAAATACAATACACAACATAAAGGAAGAACTATAAGGGTACGACTAATACTACGACAGGCACTAACAAATCTCTATCTACGCACGTCAACTCTTTTACTAACTTTTTATCTTAATACGCAACCTCCACTCTTTCCTATCCAGAGTCATGTCCTCTTGTATTATATCGTAGCATGTATAATCGCTTCTTCTCAATATTTTTTTAACATACATCCACCCCTCTGAGTATCCTTATAACGAGTCTTTTACACGTTCTCACTAAGGTGTTATCGCTCCTGTTTTATACACGTCTAAATTATTTTAATCTTGCTTTTCTTAATTTTTTCACCATAAAAATCGCTCACATTTTCTTTCGAATAACCTCATTACAAAACCAAGAGATTCAATAATAAACTATTTTTTATTATAAAAATATTATTTTTAATATATAATAAACAAGTAAAAATGTTAGGGCGGAGGATATTTCTCTTGCCCCCTAAAATATATTTTCAAAGAAAAAAAAAAGAAACTTCACCTTTATCAAAGCTCTTACTATATATAAACTAGCTTCTACTTGACTAATAAAAACTCAATTGTTCTCTCTCTCATCAACCCTCCTCTCCTCTCCCCTCTCTCTCTATATTCTATTTTCACACACTAAAACAGAGCAGCTCGTCATTTCTTCAACATCTCTGTGTATACACTGCGTTCAGTTTATTGAGAGTTTCGCAAATTTACAGACATAGTTTTTTCGCGAAAAAAGAGAGAGAAGAAAACAGAGCAAACGTAACAAAAACAGAGCAACACAATTCATACATGATAATAATGTCTACAGAGCAACCAAATTGTTCAGAAAGTACTGAATCTAGCTGCAACTCTTCTTCTTCTTCGTCGCCCTCATCGCCATCTTCCGTTCTTCTTCAGCCACTTCCACAAATCAATTCAAAAAATCGACTCAAAAGATGCAGAGGTGAAGAGGAAGTAGAAGAAGAAGAAGACGTGGTCGTTAATAATCCAAATCCGAAGAAGATGAATAAAAACAACAACAATGGCAGCAGTACTAGTGTTGTTTCTTATGTAGGTGTACGAATGAGAGCATGGGGAAAATGGGTATCCGAAATCCGTGAACCTAAAAAAAAATCACGGATTTGGTTAGGTACCTTTGCTACCCCGGAAATGGCGGCGCGTGCTCACGACGTCGCCGCCATGTCCATTAAAGGTACCTCAGCTATACTCAATTTTCCCCAATTTTCACATTTACTGCCCCGACCCGTCACGTGCTCCCCACGTGACATCCAAAACGCCGCCGTAAAAGCCGCTCACATGGATCACCTAAATCCAAAATTCTCAATATTACCCGAAACCTCTGCGGCGACGATGACCTCATCGTCATCGTCGCTGTCGTTAGTTTCGGGCGTTACATCCTCCTCGTCGTCGTTTCAAGACGACGAGGAGTCAAGACCGTCGCCTCCGGAGCTGATTCCGGAGGCGACGGGGCAGTTGAGTGAGATAGTGGAGCTGCCAAAATTGGGATCGAGTTACGAATTGGTTGAATCAACTCAGAGTTTATTTGAATCAGATGAATGGTGGGATAATAATTATGGAAATTGTGAATATTTTTTTGGACAAGATAATTATATTAGTAGTAATATGGAATTTACAGGATTAGAAAATGTGGTTTCAACCAGTTTTGAGAGTTTTTTATGGCAACATTAG

>SlERF5-5

ATATATATTAATAATTTATAACAAGTTTAATATTTAATAGTTAATAATTCAGATATAGTAATCTCTACGTAACTAAAAAATATCTACATAACTAATATTCATATAGCTAATACCCGCATAATTAAATCCAACATAACCAAATCCTGCATAACTAATCTTCACATAGTTAATACCTGCACAACTAAACTCTGCATAAGTAATACCTGTGTAACTAACTAAACCCTCCATAGCTAATACCTGCATAACTCTAACCAGTAACTAACAAAACCCCTAATTAATTAATAGTACAAGAATAGTTCTACTATTAAGATGTATAATGTAACTATGTGAGTAATAGATGTTAGTGTTCTAGTTATAGTACAAAATGTCATTTGCATTTACTTTGACTTTTTGAGCTTAGTTTTCAAAGGTGAACATCGACTTGTTATAGTATAATGGTAATAGATATAAGTAAATTTTGGATAATAATTATTTTGATCCTTTTATAAAGAAATCTTGTTTAATTTTGGAAAAATATTTAGTACGATTCATATATTATTTATACTTAGTAAATAATACATTTGTTATTATATTTTCTAATTTCTCCAATAATCTAAGTTGCTCCAAACTACTTTTGATACTGTCAATAACGATAGTATTTCAAAAAAAAAAAAAGATCAAAATAATATATTCTAAAAAAAAGGAAAAAAGAGAGTGAAATTAAGGAAATTAACAATTTTGTTATTCTAAGTTAGAATTAATTAGGGAAAGCAAATCGAAGGACTATTAATTAATTAATTTTTCTTAGCATGTGATGCATATGTATTCCATCAACTATTTAAAATATGTGGTATTTTGGTTTTATTTTATTCTTAAAATAAGTGATATTATAGATTTTTTTTTTGTGAAAAAATTATCTTTGTTTATATAATCAAGAACTATAATTTTTTTTTTCTTTGAATAGGGTAAATTAATAAAAAAAAAACTTTTGATTTTTTAGAAATGAATAATTTCTTAAGAGTTATATCCCGGTTTAAAAAGTTAATTATTTTGAAATTGAGAAAATTATATTTTAAAATTTTAATTTCTTATATATATATATATATATATATATATATATATATATATATATATGTATATATATATATATAATGATTTCGATAAATGTAGAAACACAAGGACAAAATCTTCTTGTCCATTTCCCATTATACAAAGCGCACATTCAAAAAAAATTTAAAAAATGAATGACAGTACTAATTAATTAGTGCGTCAATTTTTTGTTCTCTTGTAAATATTCTATCGATTCTTTTTTACTTACTATATTTAAATTTGACATATTTAGTAAGAAAATATTAATTGGTAAGTTTATTTTATCACAATATTTCTTTTAATAGAAATTGAGTATTAAGTCTTAAAACTAAGACTAAAATGTGGAGAAAAATAATTGTATTTTTTTTAACTGGTATAAATTGACAAATAAAAATAATTTTATTTTAAATGGTAAAGTAAAAATGAATCGTGGGGAGTATCTTTTATTGGTCAACTTACTGAAGTATGATTCTTTGCTGTTTTTTCTTTTTTTTTAGAAAAAACTATAGAGAAAATGATTTCTTATATTTGAGGATAGTTTTAAAATTTATCTTTAAATTATGTATTGAATAGTTCTGATTTTTTTAGTTTGGTAAAAAATAATATTTTTGACCTTTGTCAAATATTTAAATATGTTTGCTAGATTAAATTTAATAATAGGGAAAAAGATTTACCATAAATATCATGAAAGACTTTCTACTTGCATAAAATAAATGCACATTAAAAGCTAAAAATATATTTTTAAAAATAATAAAAATTACGTCTCAAAGAATAGCATCGAAAAATCATTTACTTTCTAAAAATAAATAATTTCTTAAGAGTTATATCCGGACTGATCAATACCATTTATTTTAAACTTAAAAAAAATCTATTTTTTAATTTTAATTTTAATTTCTTAAAACTTATCAATATATTTATTCGAAAAACTCGAATTGATTTCGACAAATGTAAAACACAAGGACAAAAGCTTCTTGTCCATTTCCCATTATTCCAAAAGCGCAGTTTTTTTAAAAAAAAATAATTAAATTCAATAACAGTACATATTAATTGGAAAAACAGTACTATTAAATTATTATGGAGTACGATTTGTTTTTTTAATTTCTTTTAACAATAGAGAAACTGGTTTCTTATATTTTTCGATAAAATTAAAATAATCATTTAATTATTTATTGAGTAGTTCTAATTTTTTAATTTGATAAAAACCAATATTCTTAATGTCGGTCAGACTCAGATATTTAAACAGGTTTGCTAGATTTGAATGGGAATTATTTTTTTTATCATGAAAGGGTATCCAATCAAAATAATTATGCAACCTAATAAAACATTACATGCTAAAAATATATTTTAAAATATAGGGAAAATTACATGTCAAAATGTTGCATCAAAATTTAGAAATAAGTCAAATATATCAAACACCACAAAAATTGTAGAAAAAATCAAGAGTTATTGTCAAAATTCATAGATAAAGATTTATAAGTCTTTTACTATCTATTTTTAACTAACTCTGTATTAACTCCGATTAAAAAATTATTTGTCTTTTAGATAACTTTGCTATATGTGATTTTAGATTTATCTCATTCTGTTTTTTTTTTCAAAGGCTTTATTCACCATAGTTTCACATTTACAAAACGATATAGAATTAATATGGCTAAACTATTTCAAATGACCTTTAGATTTTACTCTCAATGTATGTTGCTTACATCTTGTAGATATGATATCCTTTCATTGTATCTAATCCTATATAATTATATATTCGATTGTCATGAATGTTGCCCTCGAGGACATGTACAAGAAGGTCTTTTTTGATTCCTATCAACATGGTGCATCTCTTAGTAGATAGGCGGAGACTTTGATCGTAGAAATTAATTAATAGACATTTTTCTTAATAAAGAGAATGCAGGATGAATGCTTTTTTCATTCGCTTCAATAATCATCTTCTGAATATGTTGATAACTTACTCATCAATCACTACTATATAAAATTTTCGATCTTATATTTATTTTTTAGAATTACCTCTCACTTTAATAAACGTCCTTCTCTCGTGTAGCATTTCGATATCGATCACTTCTCCATTTAAATAATAATAAATCTAGATATTTTAAAATAAAAATAAAAATTGACACCACAATCTCATCTAGTCAAACTAATATTAACAAAGCTTGGGAATTTCTTTTATTTGTCTTTTTTTTCAAAGGATTTTGTTACTTGAATTAGGGCAAGTTGTGAAGAAATGCAGGAATTTGCACAACTTGAAGAAGACATATTAACAAAGCCTAGCATTTTATTTTAGTTTTCCTTGAAAAGAGGGGAAGGCAAGGAGAATGACCTTATGATAATCAAATAATTTTGCACCCATATAACATATACTATAAGAGAGTAGTTTAATTAATTGAAATATCACTCAATTTTCAAGGTATGTCATTGCTAACTACAATAAATTAGTCCACAATAAAGTTTTGACCTAGTAGTCAATGAAGGCACGGTTGAAAAATCTGATGTCTCGTGTTCAAAATTCAATGGAGGTACATTGGGTGATTCTTTCCATGCCTTAATGGACAGAGTTATTTGGTACATGTTATTGATGGGAGGTGACTATAAATATCACCTAACACGGACAAACCATCGGTCTTCAAAAATAAATTTCTTAAAAAAAGAAACAAAAGTTAACTATATACCGAAAAATGTCACTCTCTCATTCTCAAATAATTATTTGTTGTGATTTTCTTTTATATTCCCTCTGAGAAATATTCTATCCGTTTATTTTTATTTGGTCATTATTTTTAAAGTAGATTTTTACATTATTTGTCATTTTTAACATATCAGGAAAAAATATTTTTTTCTAATATACTTAAATCAATCGTTGATCCAAAGTAAGCAAATAGAAATGAACGAAGGATATATAATTAAAAATTACTTATTAATTACTCTTCATTAGTGTCTGAACAACCATAATAATACTCCATAAATAAGAATATAAGATGTTTATTTGAGACAGAGAATAGTGATTAGTGAAGAATTCAACATGATTAATTAATTCTTAAATAAGAATATAAGATGTTTATTTGAGACAGAGAATAGTGATTAGTGAAGAATTCAACATGATTAATTAATTCTTAAAATGTGCAAAGTATTTTGTCTCATTAAATGGTCGACACAAAAGTAAATGGAAAATGAATGGACAAAGTCATTTGTAACCCTACTAGGGTCTTATGTCAAACTTTCATGTGCAATACATGGTTTTTGATTATGACATAATTACTCTACCCTATACATGGCCAAAATACATCTTATAGGCTTTTAAACACATAATATTAGTTTTACCTTTTGATTTTCAAGCTAAGGTTCGAAACTCGCATTGAAACTTTGACTAAATTTGAATTGCACACTGCATTATAATTCTCTCCTTATCTGGGACTCGAATCTTAAACTTCTGATTAATGGTGAGATAGTTTTACCATTGCATTACAACCTATGTTAATCATATTATGTTATGTTATATATTCTTTATAACTGTGAAAAGATACTCGAATAAACGAAATTATTATATAGAAGTTTGTCCGTATTTATTTCATATTTTCATTTTTTTTCCTCTCCAATGTAGACAAGACCAATAAAAAAAATTGTGAATAGTTAAAAAGGAAAAAAAAATAATGTATGGCAATTACCACATGGGTGGCAAAATAGATGAACTGTTGACACCCTTGCTTGAAAAAGGTCCTTTTCATCTTTATTTCCTTTAATTTATTATTTTATTATTTAAATGAACACAACAATATTTTTTTATATATATACAAGAAGGACCACAAAGCAAAACAAACTATCAATATTTCTATTTTAATATATTTTCCTTTCTACTTTCTTTACTTGCTACAACAATACTTCACAAGAAAGACAATAAGCTATAACCACTTTAGCTAAAGAAGAAAAAAGTTATGAAAAATCAAGAAGAAATAATCAACCTCAAAAATCGTCATGATTTCATTTCTAATGGCTTGAGTTTTCCTTCAAATATCGCGACATCACCATCATCATCATCTTCTTCTTCTTCTTTTTCCTCGAATAATGCATTAGTTTCAAAAAAGTGTCAGAGCTCGAAGAAAATTGAAGAAGAGAAAAAGAAAAAGAAGATAAATAATGATGACGAAGATAAGCACCCAACCTATAGAGGGGTACGTAAGAGGAGTTGGGGCAAATGGGTGTCCGAAATTCGCGAACCAAAAAAGAAATCAAGAATATGGCTAGGTACTTATCCAACGGCTCAAATGGCAGCTCGAGCTCATGACGTGGCAGCTTTAGCCATCAAAGGATGTTCGGCTCACCTTAATTTTCCTCATTTGGTTGATCAATACCCACATCCGGCTTCCACTTGTCACAAGGATATCCAAGCAGCAGCCGCAAAGGCTGCTGCCATCCCATTTCCCGAGGAAGACGAGGAAGAGGAAGATCAAATTGAGTCGGATCAAGTTGAATTACGAAATTGCCATCCATCAACAAATTTATTCTTGGAAAATGCCAAAGAATCACTAAATTCTCCATCAAGAGAGGATGATGACACATTTTTTGACTTGCCTGATCTTTCTATTGATGTGGTGGATCAAACTAATAGTTATTGGTGCACAATGTCCACGTGGCAGCAGCTAATTGGAGCTGACACTATGGTATACCGGCTTGATGAGCCATTCCTATGGGAATAG

>SlERF1-13

AGACATCCTATATGTCACCTTCACTTCTGCAAACATAACTTTTCAATGAAATGTAATGTGTCAGGAAGAGATTGAAGTTGTGGAGGGTATAGAAGGACCACTTAGCTTACATATACATCAGATTAAATTAAATTATATGATTATGGACAAGTGCAATGTGTAAGGGAATTGATTGTTGTACTATATTTTTAGAGGAAGTATGAGAATCACACACTTGGCATACAACATTTTCACCATAATTAAATTTATCGACATTCCCAAAACACTTTTGATTGATAAAAATCAAAACAAAATACAAGAATAATAGTAATGACTACAAATTCTAATATTCCATGGTGTCAAAACATGATGTAAAGTCACTAACACACACAAAGGGAAAAAAAAAATTCAGACATAAAATTCGTGGTTAGTTTGTTGCTAAGTCATGATTTTTGATAATTATAGGATTAGTTATTTATTTTTATTGTTTAACTACGTCGTTTGTTATATTCTCTTTTTTTTTTTGATAATTATATGGTGTTGGCTTGAATATTATATATTTTGGCGTAGACAAGATGACATTTCTAAAAATTAAATATGATGTATATTTAGTATAAGTTCTAAAAATTATATACCAAGAATATTCAATTATATCAATCAAAACATATTTGCTATCACTCATTAGTGTTTTAAAAGATAATTTTAGGACTCAATTTAAGGCGTGGACATTGGCAAAAATGCCGTGTGAGACTTAGTTATGTGAATTTTACGTCCTATTATAAGCCTTAAATAAGCTTAACGCCTAACGGTCAAAGGTAGCTAGTAGATATTTCATAAAGGCATAATACATATATTGAATTCTAAACTTGGCTTCAAATTTTACTTTGATCTTCAACTTTCATAATGCACAAATATATACTTTAACTATCCAACTTTTAAATACATAAACACATGAGTCCTACATGGCAAAATACACGTAAGACACCACGTAGGACACAAAATGACATGTGTGTCTATTTTGTTTAACTTTATAAAAGTTTAAGTGTCTACTTGTGCACACCCAAAGTTGAAGGCATGAATGTGATTCGAAACCAAGTTAAAGGGCATATTTATATATTCTACCTTTCATAAATTATTTGTTCGAATTTTTTAATTTGTTAGCACTGTTGATCCTCAAAAAATCTAATAAATAAATGATGCTTGATAATTTTTTTCATCTAAAGAAATAAAAAATTGAAAATTACTCAAATAACAAGTTATAATATTATATATTTAGTACGTAAGAATATTGTGAAGTGAGAATATCACTTGACATTTCCTTTAAAATACATAGCCAAATTTTTCATCGTTATTTATTGATAGTCTTCATAGTTTTGTCTTATATTTCGAAATTATCATACTTTATTAATTAATAATTAATTAAAATCAAAGCAAATTTTTATTTATTCACGATTAAGTGATAATACTAACAAATTGGAGGGAGTAGTTATATATCATGGCACCCATTTAATAGTTGTGAGTATTATTATTTCCTTCCTTACAAAATATTTATGGTATTTTTTGTTTACATATTCATTAATAAAATATTAATTAAAAGAGATGTTTGACTATCTTTTTCTTATTTGTGTCCTAAGATATACTTTCTCGATCCAACAATAATTGTTCACTATATTATTTTGAGATGTCTAGTAGCATTACTTGTTCTCTTAATTAATTTAATGGATAATCTTACATGAAGTTTCTAATTTATCCTAAACATTAGTTATATTCATTTTCACGTTAAATTTTTCAAATATTGTACAAAATTAATAGTGGATAAATATTATTACGCAAAGAAAGGACTAATCTCACTTTATTAATAAATATTTATTCTATTTATATGTAATTATTAAGGTGTTACATCACTTTTTTGTCTTCAATATTCATTATGGTTGCTTCAATACTCTCTTAACATATTTCCGATGTTTAAAACATCCATGAGGGTATATTCAAATTAAACACTAAATTTTATATCAAATTTGATGTCAAATACTATTTTAGTATAAATCAACTCTAATCTATTGCACCAAATTAAACACTATTTTAGTATAAATCAACTCTAATATATTGTACCAAATTTTACACCAAAAAGGAACATTTCTCTCTTCATTATTATATTATTATTTCTTATTTCATTTATATTTTCTTATTTCATAAAATAAATTCTTTCTAAAATATTTTTCATATATAATTTATATTTTTTTATAGTCATTTAATATAAAATTATTTTATATCACAATTTTCTAAATAATATAAATTCCTAAAAAATATTATACATTATACATAATAATGAATAATACAAATAAAAGTGAAGTCATTAATGAAATACAATTAAATAAACATTACATAATTGAAAAAATTATTTTAAATTCTACATAAATATTCAACTTTTAAGATCACTACATGGTGCTCTCATAAATATTCTATTATAACAGTACGTAGTTCAAAATGAGCTTTTTTTTAGAATTAACATAAATTTAATTTTTTCTTAAAAAAATAATATATAGAAAAATTAATTTATAAAAAAATCTAATTTTTATATCTTAAATTAATATTATCAAAACATTACATTAAAAATAATTAAGTATACAAGAGATTTAATAAAAATATAATTTATATATATATAGTGTTTAATTAACAGTTTTGTATAATGAAATAATATTAAAATATTCAATAAAGTGAATTGGTGTGATGAAAAGTGTTACACTAAATTTGGTGTTGGATTGGAGCTCCAAAACACCATATTCTACACTAAATAAGAATTTGATGTAAAATTTAATGTTAGGTTCGAGATGATCTAAGGGATGACGTGGCAACTGAAAGCTCTGTGAAAAAAAAAGAAAAAAAATTAAAATTTTGAAATAGTGAAGAAGAAGAAAAGAAAAGAAAGTATTGAAAGTTAAAAAAATGGTGAACAACAAGGAAGAAGAACTAATTAATTTTAAAAAATATTTAAGAAAAACTAATAAATAAGTAAAAAAGAAATAAAAATTAATGATGTGGTGTCTACATGATGATGTGATGCTTACGTTGCTATGATGTGGCAGGTGAGAGTGGTGTTAAAGTCTCAGGGTATAAAAAAAATCTCTTGAAAGATATTAAGGGAGTATTTAAACATCGATGTAGTTTAAATGCTAAAGTAAGTTATGTTGTCAAGTTTAAGGAGATATTTATGTATTTGGTTTGACGTATTAAAAAATACTCCTAATAGGAATTGCATTAAAAAAAATTATTTATAAATATATCCTCTAGAGTTATGATGAAAAAAATGCCGAGGAAATTTTAAGGGGAAATTATGTTTTCAACAATGCTAACGTTTGTATTAATTATCTTTACATTATTAATACCAAGAAATTCATGGAATGGAATCAATAATACATTCTTCGATACATAATAGAGTGTAATAGCATAAAAAAAATGTAACAAACAAAGTACTACTTATACACAAAATTATACTACATACTTTAATTTTGTTAATACACTCAACCAAATGACCCCTTAATTAGTTTTTTGGTACTTTATGGATAAAACTTTTTAACGTCGTCTAAAATAAATCAATACTTTTAGAGGGATTATGTCTTTTTTTAGCTGAGTTAGGTCTGAATGGATAAAGAAAATATATATGGTAATTTTTAAAAGTCAAGTAACATTTTTTAAATTAAAAAAATAACTTCAGGATACATTTATCATTCAATCTGTTAACGAAAAAGCTAAAGTGATAAGTATAATTATTAAACGACGAGAGTAAATATACACCATTTGTTAAACGACAATGATATACGTTATAATTTTTCACAAAAAAAAAAATATTTGGAGGACCCTCACCTAAAACGTGAAATTTGAATGTTCATGAATCATGAGGAGTCTGTCAAATTAAATTGGAATATTATATGAAAGGTACCGCAAAAAATTCACAGCAAAAAGACAATGAAGTACTACTAGTTGAGCATAGGGTAATAAAGTATGTAGATATTTACAATTCCATAAATAAAATTCTAATAACCACTAATTTTTTCTATATCCCATTGGGTCATTATCAATACATGAAAAGTGGATAAATAGTAACAAGTAAATTAAGAGTGTGTTTTAATATATTTTAAAATTTTCTTTTTTTAATCTATTTTTGAAAAATATTTCTTTCTAAAAAAAAGAGAAAATGATTTTTCAATATAAGAATAAACAAAATTTACCAAGTTCCACCTTCACTTTCATGATATTTTTAGTTATGTATATATTAAATTATTTCACTTATTTTGATGAAAAACATTTTTAATGAAACAAAAAATAAAAATTCAAATAGACTATTTGTTCCAATTAACAAGTGCAATTTGGAAAGCAAAATCTGGTGGGAGGACCCACTTTTTGAACTGGATTTTGGACCCACTACATGTGACCCATGTGTGGTAATTGTATTTTTTTTTTAAATATTATAATTTTAAAGAAGTTTAATTTTTATTCACCTATTTTTGAGAAGCTCCAAAAAGTCTTTGATTAATTGATTCAATGGAACTCAATATGTTAATGTTAGTCATGTGTATTGATGTTCCATTTTATTGTCTTTTTCCTAAATATAAATCACAAATAAACATTTTTATTGCAACATGGAAGAATTTTGTTTGCTCAATAATGCTATTTAAATAGTTTTTATTCTTTTATGTGCATTTGATTGGTCAACTTCTGTTTCCAACAGTTTTAATATTGGATTATTTGATTTTTCATGTTTGTTGAATATAATTTACAATAAAATCAATCAATTGATAGAAAAGAACAAAAAGTTATTAGGTAAATCTCTCCTAGCTTGCCAGAGTGGAAGAAAATGGTAATATTCTTAAACTACTATTTAATAGCCTGACATTGCCAACTACAATTAATTATATTTATAATTTCTAAAATTTATGTAGTATCATACAATAAATTAAAGAAAAAAGGGCATATTTTATTTATTGTTTTTACTAAATTTAGTTACTATGGTCTATGAGTGACTAATGGGTATTTATATTCAATTTTCAAGCAGACCGATTGAACATTTTTTTTTTTTGCGATAGTAAATTAAATAATTTTTTGAAGTCAGAAGAAGCTATTTATGTGTTATTAAATAGAAATAAGAAATATCATTCGTTAAAAATTTTATCCGTGGTCAATTTTTTTTTGTTAATGAATCCATTTATATTACTTGGTATAATTATCCTATTATAATAATTATTTTGATATGAAATTTCGTAAATAAATCTTATTTGTGCAAACACAAGATCTCAATAAAAGACATGGATACCCTACAATAGGGCCCCTAGGTCAACTAGTTTTAATTTCATGTGTATTGTATGATTTTTATATAATGAGCACAATTGCTCTCTCATCATTTTCATTTATTATATTATATTTTAAAAATAAATGTCTATTTTTATTTGTTCAGTTTAAAAAAAATTAAAAAACAATTACAAGTCATATATATATAGTAAAAATTAGTTTTATTTATTGGTTCTTATATGAATGTGTCAATTTAAAATTAGATGGGAGAAAGTATATGTGGCTGCTATGTTAAGTATGCAAGGTGCCAAAAGAGTACTAGACTATGAAAAAATTGCCAAAACAATAATGCTACTACTAATTATTATTATAATTAATAGAAAAAGTAAAAGAGAAAGAAGAAGAAGAAAAGAAAATATGTATATAATATTTTATACAAAATAAGCAAAGCAAATAAGCACATGGTTAACAAAACAAGTGAACTGTAGATACCCTTGTGGCAATAAGGTCCCATTCTTCTTTATTTCCTTTTCCTCTCCTTTCCTTTATTTAAACCAACACAAATACAACCATTTGTATTATTATTATTATTATTACCACCACTATACATCACTAGAAAGAAAAAAATAAAAATCACAATGGAAGATCATAATGAATTCTCCATTTCTAGTTCTACTAATTCTACAACTTCATCCTTTACAAGTTGTTGTTCTAATAACAATTGTTTGTGTTCTACTTCAACCACAACATGTTCAACTTCTTCTACTTCATCTAATAATTGTTTACATGAGAGCATTGCAAAAACCTCATCCAATGATCATTACAAGTGCAAATCCAAGAAAAGCGCGAAAAAAAATCAAGATGGGAATGAAGAGGAGGATAACACGAAAATCAGAAGAAAAAAACAATGTGTTGTTGATGAAAAACATCCAACTTATAGAGGAGTTCGTAAGAGGAATTGGGGAAAATGGGTGTCTGAAATTAGACAACCAAGGAAAAAATCAAGAATTTGGTTAGGGACTTATCCCACCGCTGAAATGGCAGCTCGAGCTCATGATGTTGCGGCTTTAGCCATCAAAGGTGATTCGGCTTACCTTAACTTCCCTCATTTAGCCGATCAACTCCCTCGACCCGCTTCCGCTTCCCCTAAAGACATCCAAGCCGCCGCTGCCAAAGCTGCAGCCGCCAGCATATTTCTTGAGGAGAATTCGGTTGAGTCGAGCCAGTTAACCGGGCTGCACAGCTCGCATTCCTCGACTAATTTAGCCTCAGATAACGTACAAGAATTACTAAATTCTCCCTCGATGGATCACGATGACCCGTTTTTCGATCTACCGGATCTTATCATCGATAGACTCGACCAAAATGACCGGTACCAGTACCCGGTTTCGACGTGGCAGCTGGCTGGAGCCGATAGCGGGATGTTTCGGCTTGATGAGCCGTTTTTGTGGGAGTGCTATTAG

>SlERF4-10

AAACGATTTTTATTTTTTTGAAATATATTTTATTGTCTGTCACAACATTGAGCAAATCAATATTTTATATTTAACCTGATTTCAGTTTAACCCGCTCATTTAACACACGGGTGTTGAAGCCTCATTCGCATAAAAGTTGAAACTTGTTAATCTCATTGATTGTATTATTAACAACAAAACACAGATACATTTTGTGGGGTGTGGGGATTGCCTGTAAAGTCTCTTTCTATACCTAAAACACAACTAATTTTATTTCAATATTTGTTGCATTTTAAAATGATCCTCTCCCTTCCTCCCACGCACATACCAAATATTTAAGTCTTTATCAACTTTTGTATTAATTTATTTGTTTTGATTAAACTAATTAGGGAGTAATACTATATATCAATCAATCATTTAGGTGTCAATCCAAAATTAATTGAAGTTTATTATGTTAAATTATTCTATTTATCCATTAATTATATTTGTCATTAGTTCCATAATATTCTACTCTTTTGTTTGAAATTGGATATATCTGTATGCTTCTTGCTCTTTTATGTCCATTTTTACTAAATTCTCTTTATGATAAAATGCCTTTTTTCTCTTTGTGGATAGAACATTCTGTTTTGTGTTGTATATATCATAGTTCGTTATGTATTTGGCAAAAAATGAAATTGTACATCGGTGATCGATAATCGTCAATTTAATCTTTTAAATTTTTCGTCATTTTATTTATTTAATTAAAAATTTTATCATAAAATCAATCTAAAAATATAAGTGAGAATACTAATAAAAATAATAAAATACAATTATTACGTTTACTTAATCTACTCAGAATGAATCAACAATAGTATATAGTGATGTTGATCTTCCAAGTTGATTAGAAATATGTCGGCGTTACGTTCTCAAAGAGTAAAAAACGTGAGAATCATAAGAAGTTGTTATTCATGTGAAATAAATAAAAATAGAAAAAAAAATACAAAATAATATAATACATATATCATGAGGAGTAGCACCAAAGCAAACTTCTATATTAAATTTTTCTGTAATTAGCAGCCGATTTATTTGTATTGCGGCGGACGCAAGCAAAGTAAGCTTATCGATTTTATTTTTTTAATATAAACTATGATCTATTAATTTATATTTCATTTATAAATAATTTTTTTTATCTTGAACGTGTAAAAATTGTGTCCAAAATATTATATTTGTTTGTATTAAGCAGTCGATTGCTTTGTATTTGCGGCGGACTTAAGTTATATATATCTGAAAATAGGCAAGTCGGCAAAACCCCACATGTCCCTATTTTCATTTTTTAGCTCGGTGCGCATTTACTCTAACTAATATTATAAATTAAATAAAGTGGCAAACCTCTTTAAACATGTTTAGGTTTTTAATACAAAATTTTTTAAACACTAACTATATATGATTTCGATAATATATTTTGATAACATTTATTTATTAGAATTGCATCAAATATATTAGGCAAAACTCATTCGATCACTATAATAAAATAATTTTTGATCATATTAAATATTAATATTAATAAAAAGTGTTAAAGTTTTTATAATATTAATTAAATGTCATTAGAATCAATGTCGCAAAAAATTTAGAAACATATATCAGAAATGTCAGTTGCCACTAATAATATATATTTAGTGACAATAAAAAAATAATTACAACTAATAATTATTTTTATTATAGTGAATAAATCAAAAGTGAAATAAGATTTAATGTTCATCAGATTTAATCTTTTTTTTGTACTAATAACCTACTTTTGTAAAAGAGCTAGTTTCATTAGAACGTGAAAGATTCGGAATAGCTAATGCAAAATCTTAAGGTAATGAACAAACTCAGATAGGTTTAATATAATACTCTAAATACAATATAATACTATTTGTATTTGGTGAAATTTATAATTTTTTAAACAGTTTGTACAGAATTAAAAATAAGATCGTATATGATATACAAAGCACGAAAACTCTTAATATAACATGATATTATTTATTTTTTGTGGAATTCATATGAAATTTTCACATAATTATTAAAATATTAAATTATGAAAAGGATAGCGCAATAACCAAAACGTTTCATATTAGTAGTAGTTTAAAGATATTACTTGGATTACTTAAAAATCCAGTATCAACAAAAAATAAAAGAAAAAATAAAAGTCATACTATATAATATTTTAATTTTTTATTATATTACATAGAAATAGAGTTCGGATTTGAAACGGAAGTAAGGTTATATGTAAATCTGAAATCAGTGGCATGTACATGTGTTTACAAGTTTATTAATATATATAATTTGCGTGGACATATAAAAGTCACTCTCAAAGGCCACCACACCGACTTACTTTCACACAATTTGTTTTTTCCGTTTTGATGATATAACCTATAGTATTTATTTTTGCAATTATAATAATATTAATTACCGCCCCTCCTAAAACTTCAACCAACTTGTCAACAATCTAATTAAAGTCATCCTCATCTTTAATTGTTAATCCATTTTAGTCAAAATTCATTGTATTTTACTTCATTTGAGTTCTACCACTAGATTGTGTTGGCAAAGATATATATATATATATATATATATATATATATATATATATATAGTGGAAAAGAAAAGAAAATGCACTATAATGAAAAATATATTTTGCAATAGTATTAATCATAACTACTAAAGTCATTATCAATATTTTTTAATTAAAATTTGTTAAAAAAAATGAAAAAAGTCTCACATTAGTAGTTAACAATATATCGGTAGTCAACAATTTTTCTTCCTTTGATCTAGTTTTTGGGGTCTGAATTAGACGTAGGATCTAATTTTACAATACTAAATTAGAGACATTTACATTGCATCTGATAAGACATATTTAACGATAATTAAATCGTTATTATTAATTAGTTATAGTTAAAGATGATCTTTGATGTAATGACGAGGCAAAATAAGAAAGAGGAAAAATAAAAATAAATTGTAAAAGTATAAGTTTTTTACGTGCTTACATACACTTGTCATTGTATAAAACCTGAATATAATTTCATTCTTAATTTATTTAATATTAGACTTCGTATTATGTTATTCAGACATTGCAAAATTAATCTTAGACATTTATGATATTGAATATAATTATCTCATAAATATATTGTTTAAGTGTCCAATTATAACATGACGTGCTGCTTCAATGGCTCGATATAAAAGACGATCAGTTCTACAGCTTTTAATGTCATATCTTCCAAAATCAAATTATGTACTGTCCGATTTGCAACCCCTAAAAGTTACAGTTAGGTTTGATGTCTATTTATTAGTACTATTTTAATTGTTTTCCTAAGTTGTTATAATAGCATATTCATCAACATCTTTAAATTAAATCACCAGTTGGGATCCATAATCAGTCAGCTCCGAATTGAGATTTCTTGGCATTATATATTTTTAAATTAAAATATTATGCTTAAAAAAGATGTTTTATTGATTTAAGTGAAGATGTTTCCACATTTGTCGTTATCGTTGAACAACAATCAAAAAAAAATTATAAAAATTAAACTACATAAATATATTAATATGATTACAAATATATCTAGTCGTCTATCGATATGATTATAGTGAATAAAAGACTTAAAATTATATTTATGAAAATGAAAAAATCATATTAAAAATATGGCTCAAAATATAAGCACTATGGTATATAAGTTTAAATTTGATTAAATTTTAAAATATTAATAATGAATGAGAAAGGCATTCAATGTCCATATCGGCCAAATAACGATTGATATCTTTTGACTTAATTTATTTTGAAGACTACTTTAAATTGTAAGTTACAACTAAAAAGCAACCTAATTGTACCCTTTGTGGCTCTTCCTTATTTTTTTTAATAGCGATTACAATACTATTAATTCAAATTTGCGTCACATAAGATCTTATTTACTAATTCGCGATAATTTTAACCTTTTGCCATTCGTATCACATTTAAAACGCTTGATCTTTGCGTGAAGAGGAAGTGTGACACCACAATTTAGTTCAATTTCATGTATGTATTTTGTTTATACACAATAAATATATAAATAAATAAAAAAGAAGATAAAATGAGTAACATTATTATCAAAAGTTAGTGTATTTAGCCTAGTAAATTTTAGAAGTCATTTGATTTTAGACCAAAAAAAAAAAGAAAAGGGAAGAGCCGACCATCAACACCATTTTTAATAACCTCAATGATTATTCTGTACCTCTTTAGATAATTATTTGAAGAGTTGAATTTCAAGTTTCCTGTCTACAAAAGCTATTTTTAGCTAAACATGTGGAAAAATTGATGAGTATCAATTATTATCCACTTTATTATAAATCATTATTTCAAAATATTTATTAATTTATAAATAAAATTATCTTTTTCCCCCACACATTTAGCTAAAAATACCTTAATAAAATAATATATAAATATCTTTTTATTATTGATAATGCATTAGGCTAACTTTTATCGGTTATCGAATGACCCTTGAATCGTAGAATAAAATTATTCCTCCCATAATTAATTAGTGAAATTGTTGTGAAAAATAGCTCCGATGAAATATGTCGGCCGATTTTGTACTTATCTTTAAGGAAGATCAACGTGGCGGCCGTGCCTAGACGACAGACTAGCAGCCAAGCAAAGAAAACAAATTACTCTCCCTTTATGCGTGCTCCATATGCTCTCTTTCCTCAATCATCCACACAAACGCGAACAACGTAGCGGAATAGTAAATACTCTTTCATTAATAAATTTTTATTTAAAATATTTTATTAAAAAGTTAAAATTTACATATTTAAATTTAATTAAATTCTAATATTAATATCACATCATCATACACCACCCACCCTGGGTTTTTCTCTTTCCATATATAAATACAAAATTTATCTCTATATTCCAACCTCCATATATATTCTAATAACAAACGTTTCTTCTCATATATCACATTTTCTCCTTTCTCCATCTTCTTTCTTCTGAAAAAAACTAACAAAACAAGTGAATATATTTAATTTTTTTCAGTATGATGGAAGGAGAGAAGAGAAAACAGAGGCAACACCAGCAAGATAAGCCATACAGAGGAATACGGATGAGGAAGTGGGGTAAATGGGTAGCTGAAATTCGAGAACCAAACAAACGCTCTCGAATTTGGCTTGGTTCTTATTCTTCCCCTGTTGCTGCTGCTCGTGCTTACGATACCGCTGTATTTTACTTGCGAGGTCCTTCAGCTAGGCTTAATTTCCCTGAATGTATAGTGGATGACCATGAAATTCACGATCTATCAGCTGCTTCTATTAGAAAGAAAGCTACTGAGGTAGGTGCTCGAGTTGATGCTCTGCAAACTGCGATTCACAATTCTACTGTTAATTCTGTTGAATCAAACTGTAATTCGAATTCTAAATCTACAAGGATGATGATGAAGCCTGATTTGAATGAATATCCTAGCCCGGAAAGTTGCGACGAAGATAACTGA

>SlERF11-4

TTATATATTGATTTTAAACCTCGTGAATATAAGATAGAGGTTGACTTAGTGGCTGAAGGGATCAAATCTCAAATCTTAAGCATTACTTTTTTTTTCAAACTTCTTAATGAAAATTCTGGATCGATCACCGTAGGAGAGGGTCTACACTGGATTCACATGAATCCATGCTTTCCTCCCAAGATCATATATAATAGTGTTATATTTTTCAAAAATCATCTAAATATAAATGTATGAATCCACACTTGAAGTATATATCATATAATGGCATGCGATGATGATTGGTTACTGCACCTCTCTAAATGAGGTTAGAAATATATATAATTTTTTTTTATCTTGTTTTTTTTTTCTAAAATTGATTAACATCTCTCCGAGTAGTTTGGATAGATAAAAATAATTTTAAAATTTTCAGTAATAAAAATGTCAAATCGTTCAAAATTTATATCTTAATCTACTTTTTCGTAATCGTACACTCATTATCTTAAAATCCTAGATACATTTCTGTATCGTGCACATAGCCGGACATATTATGACATCTATCCACGTAACAAAGACTCTATTCCAACAATAACTCACAATTCATACAATAAAACGTAAATCGCACTAGACAAGTCCCACATCACGCGACTAGGTCATGAGGGTGAGAGAGAATATGTTATTATGTGGAGTGATATTGATAGATACAATAAATAAGAAAAAATAGAAACGCGTTTTAAAAAGGACAGAAAAGAAACAGCTGTTTTCCCTTACAACAAATAAAATCTATTATTATTATTATCATATACATCTTATATCTTTATTTACTGTTCTATTGCGCGTCTTTACATTTAATTTCCCCCTAATTCTTATTACTACCTTCTTGCTTGTTTCATAATTATTATTATTATTATTCATAGAATTAAAAAAAAAGTTAGTATGAGATAATTGCACCCATAACACCTTAATTTTTTAATAATTTGCTTGAAAAACTTATAACAAACCTCTCTTTATTATTTTAAGAAGGGTTACACTTACTCAGGTCAGTCTTAAAAAGAATAACACAATTTTTTAAAAATAATTTAGACACAATTCATTTTTAACATATATATGACTTATTTTTATATTATGAATTTTGAATATATATATTTTTTTTATTTTTTAAATTTTGTGTCAGTTCAAATTAGATGGTAGAAATTTTGTGTGTATGCTAATGCTAGTAGTAGCATATTAAATATTTATATATTCCTATGGCCAGCCCTTCTTCTCTTTGAGAGTACACTTTGTTGCTGCAGAATCCAGGAAGCTGCTAATGACAGAATCCTATGTTAATTTCTCTCTCTTTAGCACTTAATTATTTTTTCAACACATAGAATATTTAGATGCAAGATATGATGATGACGACCTTTCTTCATTAAGACATGTCATTTTTATAATAATAATAATAATAATAGTAATGTTCTTTCACAATTACATTTTTGCCCACTTCTTATTCATTTACTACTCTAATATTTCTTTTTAATTTGTTGAATTAGCAAAGTATAAACAAGGATCAGAATTTATAGTTTTTAAATGTCTAGGTAATATTCTAAAAATGTTGAGATATGCTTTCTAATTTCGTAATTAAGAATCTAATAACCTCTATATTGTTCAGACCTTTCACTTGTGAAATTTCGATCGGTATGTTTTTGTAATAAAAAAAAGGAGATGTTAATTTATCAAATAGTTAAGTTTAGGAAGAAATAGAGAATATTTTGAAGAATGAAAAGATTGTTGAAGAAATAGACAAACAGACTAAATATTGTTGATAAGTCATGAAGGTATCAAATCAGTTTGTAGTCAGCTTTAGTGTAATGCAATATTATCCTCATCATTTTGGTGATATCATTTTAACAAAATAATATAATAGTATTAATGTGATGAAATAATTAAAGAGAAAATTTCAGAAATCACAAACGTATATAATAGACATAACAGAGCTCTATAGTTATAGTTTTGATAATTGTATTTCATAATTATAGTATTGAATTGTTTAAGGTTGTTGTTTTATATCTGTACAAGTTCGTATATTTCGCTTGCGAATATACAGATAAGATAAATATATATAAATTATGTATTTGTATATTTAGAGATTTTTCAACACTCTGTTTGTATGCCAATATATAGGATAAACATATATAAAATTTTTAATTTATATAATCAGGAACCAAATTATACAAATTCTGAATTTATATAAATCGAGGGTATGACATGATTGGGCAAAATTGTAGCTACGAATTAGAATTTTTTTCTAAAACGATAGTTATAACATTTAATATGATTTGACTACTTGCTATTCTCCACATTTTTTCCATAATTATACTCATTTCGATTTAATTTATTTGGTTTATCTTGACTTAACACAATTTTTTTTTTTAAAAAATCTTGAATTTTGTGTATTAATAAAAATTTTGTTTAAAAAGGAAATGATCATTCTATTGAAGTAAATAAAAAAAATAGATCAAATAAATTAAAATAAAAGAAATAATACTTATTTCCTTTTGTAAATTGTGTATTCGTACGATCTAAAAACAAACAACAATATACTCACAAATAAAATCTAAATATTAAAAATAAAATATATGCAAACCCTCAATAAAAAGAAAAGGTACACGACCGTCTTAAATAAATATAAATTAAAATGAAAAATAAAATAAAATCATACACATTAGATTAAGAATAAGAAAATAAACAACACTACTAAAAAGATATTACTTCCTCCGTTTGAAAAAAAAAACGATCTTCTTCTAATTTTAGTCTGTTTTAAAAAAGATAAGGTCTTTCTTTTTGTAATATTTTAATTTTAATATTTCACGTAACATGTTTAAAGTCACAAGATTAAAGTATATTTTATACATTTGACATAATTTTAATTTAAGACCACGAGATTCAAAAATCTTCTTTAATCTCTTAAACTCAGTGTAAAAAAATTAAACTAAATCATTATTTGTGAACAAAACGAGTATCTTCATACTACTTCTTCTTATTTTATTTTAATTTATAAGAAGTTTATAAAATAATTTTGTACAAATCAAAAATTACTTTGTTGAGATAACTTGTGGCTCAAGTCAAGTAATGCAGAAAAGATATCAGCGCGTATGTACTTACGTACTTTTTAGGATCCTTGCTGGTATCCAGACTGCCCCATTTTTCCTTTTACCTTATACTCTAGAAAATTAGTACCATAATATATTAACGTGTTATTTAATGGTATAATATATAAATATAATTTTTAATTTAATGTTAAGAAGTAATTATAATTTTTAATTTTGGATGTATAGAAGCTAGATAACACTTAAATTTGTATATAATTGAGCAAATAAATTTATTTGTTCTACGTGGCATCCTAGATGAGAATTTGGTGTCATACGTGACGTTTTATGTGTTATGCCATATAGGACAAGTCTGTCTATACTTGTTCAATTTTATACAAGTTTAAGTGTATACTTGTGCTACTCAAAATTAAAGAGCATAGTTGTCTATTTGAAGTTGGGTTCAGAGTCATATTCTGTATTATGCCTTATTTAATTTGATCTCGACTATTATTTGTGTTCTCTAATTATGAATGTTTGTAGATAGACATTTAAATTTGTATTAAATTGAAGAAAATGTATTCTGCTTGATAATTTGTATTAGGCAATGCATACTTATTTAATTTTAATTAAATTTAAAACTATATTTATATTGTATATTTCAAATTGAATAACATAAATATTAAATAAAATTAAATTAAATATATTTATATACCTCATGAAATCTATGAAACTTTTCTCTTCCTGGTTTAATTTGTTAGTATTTGACTGCAATTATTTTTTCCAAGCTGTCAAGAAGCTCTAAAAAGTCTTAAAAGGGTAAAATTTTATCATTTTAAGCATTAAATATTCTCTAGAAAGAAAAAAAAAAGGTATAAATGAGACAGGAGCACATGATAGTTTTGACATAGTCTTTATCGTTCGAAGGAAAAAAATATTTTGAATATTACATGTTACATACACATATAACGATTTGGTTTCTAAAATGCAAAAAAATAGTAGTCCAAGATTATTTTTACTCTCATCATTTCAATTCTATTTTTCATCTATTTAAAAAAAAATAATTCAAATTTAACAATTTTTTAATTTTAATTTTTCACCCAGTGAAACCACAACATGAATATAACAGAGCAAATAACAGAAATCACATATTTTTGACACAAAATTATCATTTGTCTTTTATAAGTTTCTAATTATAGAAATTCCTCAAATTGATACAAAAATATAAGCTTTGATACGATGCGTAAGATACATTAGATTTTATACATGATGCACTAATTTGATATATATTTTATATATAATCTAATATGATGCATAATTTATTGCACGAGATAAACTAATCCGATGCACGAGATACATTAATCTGATGTCCTAATACATTAATCTGATGCGCTAATACATTAATCTGATGCGCGAAAATGAGGAATTTTAAAAATTTGTAAAACTAATAGAGGATAATGGTAATAAAAAAAATTTAAAGGTGAGTTTTCTATCATTTTTCCAATATAATATGCATAATTAATCTTTTAACTTTTTACTTCCTTCTTAAATAAAATTATATCATATAAAATCAAGATGGAAGAATTTCTTTTTTTTTGTTAAGCTAAGGACAAAATTGTCATTTGGATGGATAGTAAAAGGACATTTATCCAAGTTCTACATTGGCTTTAAAGTACTGGAAAATGTCTGTCCTCCACTCTGATGTTTACATGTAGACATTATACTCTCTTCACTAAAATGATCTCCTCACTTCAATGCTCACCCCCCCCCCCCATAAATCACCCTACCCCCACCCCCAACTCAAATTAGCCCATACCATCTATAAATAATCATCCCTTCCTCTAACTTCAACATCACTTCATCAATATCCATCTCTAAAACATAAACACACACAAAAAAAAGAAAGAAAAATAAGCAAACAGTACAACTAACACAATTTTTTTCAAGATTCAAGCAATGGTGAAGACAGAGCAAAAAAGTCTATCAATGTCAATATCATCAATAGCAACAAGCAATAACAATAACATGAAGACAAAGAAGAACAAGTACAAAGGAGTTAGAATGAGAAGCTGGGGATCATGGGTATCAGAAATAAGAGCACCCAATCAAAAAACAAGAATCTGGTTAGGTTCCTATTCTACCCCTGAAGCTGCAGCTAGAGCCTATGATGCTGCACTTTTATGCCTAAAAGGCCCCTCTGCTTCCTCAAATCTCAACTTCCCTTTCAACTCTACTTTCTATCATCACATTGATCATCACACTTGTACAACTCTCTCTCCTAAAGCCATTCAGAGAGTAGCCGCGGCTGCCGCGGCCACTCCTGAACAACAAAATGTAGGGTTAGTACTGTCAGATAATAATAACAGTACTAACCCTACTATTTCATCTCCACCACAATCTTCTACTTCTTCTTCTGAGGATATAAATGATGGTTGTTTATTATCAAATATCAATACATTTGATCAAGAGATGTCAATGATTGAACAATGGTACAACTTTGATTCACCAAAGTACAATGACATGTTACATGGTACAATTTTCTTTGATCCACCTTCAATGGAGGAGGTTTATTATGAAGAATCATCAGCAGATATACCTTTATGGAGTTTTTGCTAA

>SlERF10-4

TGTTTGTGTAATTTGTTTAGTACAAAGTTTATATGTCTTATCATTTGATTGATTCATTTCTTAATTGTTGTTAATAAATATGATATTGAATGTTATATATGTTTACTAAAGGTTTTTAATTCTAAATTTACATAATTAGAGAACATCATAACCACTCCTATATTAAATTGTTTTATTTCTTATTCATGTTGTTATACTTCCTATACATGAACAATTTATGTATTAAATTGTTGTCAAAAAATGTAATTAAATATCCTAAAACACTGGTAATACTATTCCTTTTGGATTGTGGGAGATCTTTAATGTTATTAGGTGGGGTGGACAATATTATATCATTTTTAATTAGAGGTTCAAATTCAATTGAAATGAAATTGCTAATGCTCTACCCTTTTAATTAAAATTTGTCGATTCAAATACGAATTTATTGAGATTTAAAATAAATATCAAATATTGAGTGGTTAATCAAAAAGGATTGGAGATTTTTGGTTTGAAGGCAATCTCATACTTGGCTCTTTAGCTTTGCTCGAGTGTCAAAATAGATATCAAACATCGAACGTTAAGTTGTAAATAAAAGATCGGAGATATTTGGTTTGAAGGCAACCTCATACCTTGATTGTTGTCATTCTTCCCATTCACCATATTTCACCCACACACTATTTAGACTTAATATTTTGATTCAATATTTTTATCATCAAACAATAAAAATAGTGAATCCATTAGAAAAGATGAGAACCTCGTTAATGTGCATCTGTTTCCATAGGTTCATTACTAAGCTGACTGTTGATTGATTATATCAAGGGTAACATCTAGCAACAATCATTAACCATCGTATCACATGAACTATATGATATACGTACGCTCAAAAATTATTGAGAAAATATATATGTAAAATTCAATTTAATCGGTAAAAATTGATTCCTTTTTATAGATTTATTACAACAAAATTTGTCAATTCAACAACAAAAGTTTCTCCCAATGGAATGGTTTTCAAGCCTCTGCCTAAGATCGATTTACTATCTCTTCTTTAGCTTTAACTTGAACACACGTGAATCACTCAATCGAAAAAGAGGAAGACATTAGGAGCACTCATTCCTTGTACCCGGGGAACTGATTCATAAGAAAGGGCTATACCAGAAAAGGAGTTTACGGTGAATTCCCATAGACACTATTTATTTCGAATAAAAAAGGAGCAGCATAAGCCTTTCGGTCTTATATATCATTAAAAAAATAGATATATCATTTAAAAAAAATAAAAGCATGAAAATAATGCAATGGTGATATTTAGTAGAATGCACATATCATTAAAAAAATAGAAGGGAAAAGAATTAAATCAATTTATAATTATCTAAACTCTTATCAATATCCACTACACAAATCTATTACTATTCAAAAGTCTTCTTCTTCTTTTTCAAGCCAAATAAAAGCATGACTATAATGTTATCGTGAAAATGAAGGATAACTTCGAACAGGATATACATTATAAAACAAAAAATATAAATTTATACATAAAATAATGGAGGAATAGCATAAGATTTCATATTTAATATTTAAAGGTTATGAATACGAAAAGTTGAAAATTGTGAAAGTTATGATATTTACACATTATAATATCTAATAAAGAAGATATAGTAGAAGGTGAAAAGTTGAGTACTTGAATATAAAGACTATGAATAACTAGAAGAAGTCGATGTTGTGGATCCTTAAAAGATCTTCAATGATTTCTAGACCTCAATGATATTGAAAGCAAATATGGTTAGTAAGCTACCGAGATTTTGACTGAACGAGAGTCAACATAATAGATATAATAAGACTTTACAATTATAATTTTGATAATTATATAGTATAATTATAGTTTTATTTGCAATAGAGACTAGTTTGTATATTTTTATTCATTGTGTATATATAAATAAGGTAAATAAATACAATAATTCACTTTGTATAAACGATAAAATAATTCACTTTTTTCATACAACAATTCACTTTATATCAATTATTATAGAAAATTAGAATCGTTACTCAGAACATAGCCGATCTCCTACCAATTATTTTTTAAAACCTTAGCCATGATTAATTCAAATTTCTCCAATTAATATGTTCTATAGATGAGAAGAAGAAAAAATAAAAAGGGTGTGCTTTTAGTTAAAATTTGTATTTAACAATTTGATAGACATTTCAAATCAAAGATGATAAATATTAAATTAGATTCAATAGTATTTTTAAAGAAAAAATTGATAGGGCGTAGGGGAAGAATATAAAGTGCATGAGACATCGAACTCATACCAAAAGTGAAGGCGGATTTACGTGAATTGGTGGGGGTGCTATGAGCCTATGACATACAATAATCTCGAAAAAATGTTATATATATATATATATATATATATATATATATATATATATATATAAAAATTTTGCCAGCACCTAATTTTTGCTTGCTCATTTTTTTCTTTTTTGTCTTTTTTCCTTTTTTTTCTTTTCTTTCCTTTTTTTGTTTTTTTCAATAATGTGTAAAAGGCATAAAATATTTTTCATTTATTCGTATTTATTTACTATTCTTTATTAATTCAAAATCGACTCTTTTTTATGAATAATTTTTAGTTTTCTTACTTTCTTTTTCACTAATTATTAATCTCTTGTTATGAGTGTTGTATGTTTATTCTTTATCAATTATTGAGTATTGAATTATGAACGTCACCTAACCATTATTAAAATTATATTTAAATAGTAGTTTTTAAGAAATATAAATTTTTAAACGATTGAATATTATTATTAGATTATTTTTAAAAAATTTAAAGGTTCTTTCGATTTTCTCTAATGTTCTTATTAGCAAGATGACTAGGCTGAAACTAAGATTTTTATTCTTTCCCTACGATATTCTCCAACAACACATAAATTCAATGGAATATGAATATATTAAGTTAATGTCATTAGATGGTTTGTACAAGCTTAAATTTTAGTATGAAAAAACTTCTTTTACCTCAAAATTTAAGTGAATAAAACCTCTAATTTAAAAAATGACATAAAAATTGAATTGTTTAGTTATCTCTGATCTCAGAATATCTAAAAGAAATTAATACCAACACGCATACTTAAAATTAATAATCCAAATTATAACTCGAAATTAAAACAATTAATCATTTTATCTTAATTGAACGATGTGATTTCCACTCCTCTAAATCATAGATCCGTCTCTTAGTTCATATATAATGTGATAATTGAGGGGCATTCTTTACTCTTTAGCCCATAAAACAAGCTAGAGATATTTATAAAACAATAAATAAATAGAGGATATTTGGCCCTTGTCCATTAATTGAATTTAGTTCAATGACATTACTTGAACTTTTATCTTATAATGTACTTATTTTCAACCATGCAAGTAAATTACACATTTTATGGTAACCTTTTCATATTGTCTTATTATACTTTTCTCTTTGCGAGTAAGTCACATAAATTATATAAAGTCAATATCGATCAAATAAAATGAAACAGAAAGAATATTATGTATTTTCTTATGTGGCCTTTTGGTATTAACTTTGCGCACAAGAAACAAATAGTCACAAAATTGTAATGTAGAAACTGAAAGATCTCTACAGAAGTGGAAAGAATAAAAAAATACACAAAAATCGTGGTCACCAAGAAAATAGACAGGACAGCCACCAAAGCATTAAAAGATAATAACATTTTTTTTGCCTTTAATTTATAAAATATATTTGATTTCAAATGTGATATTATTTAATTTGACATATAATTTAAAATAAATAAAAAGCTTTTAATTCATCATTAGAACACATCTTATATATTTATGTAGTTGAAAACACGATATTATGCATAATTCTATGAGACTTGACAAAAAACTTTGTACATATTTTAATTATTTAAAAGGAGGCGAATCAAGTAGAAAACACGGACGATACTAACAATAGAGTACCATTATATTATTTTACAATAGTGTAAAAATAATTTTCAGCAAAAACAAAATACTTATATAACTGGAGAAAACAAATTAATTAGATCACACGTAATACTAATATTGAATATTCAACAAGAAAAATTTAAAACCTTACAAGAAAAAAAAATATTTAATACTTCATAACTTGTTATTCAAGGCCTTTGAAATTTCTTGTGGATTAAATATAGTAATAATTACTATCCGAGATGCTACAAATTAATTTACCTTCAATAATGTGAATAACATTATAGTTTGTGAAGTTGAGACTATTGAACGTTAATCGAGATTGTTTGTGTTCTTTTGACCGCACAAAAAAATATATTATTATTTTCATAACAGTGTTATAATACTTTTTTAATGCTAGCAGCATAAAATATCATCATAATCTACTCTCTATTGATTCAAACAAAATTAAATTCTAAAGAGAAACATTGATGATTGATGATTACAAAAATATATTTATCATTTATGATAATTAATAGGAAACTATCATTAATTATCATAGATGTCCGTGCTTTCAATCTCCTAGGCCCAAAGGCAAAAGATTATTTTTAAACTTGCTTTTAACATATAAATTTAATAGGTATGATTTCATTTTTTTTTTTGATTTTAAAAAGTCTTTGCCTTTGTACCCAAGGAAAATCCCTTAATTAATTAGTTACATAAGATAAGCATAGTAAATACTGTTAAGTAATTCACACCATCACTACACGTTACTACCCAAAAAAAAAAAAACGCCTTATTTGTTTATTCATTTAATTTTTATCGAAAAAGGAATTCCAATCCCAATTAAGCTAGGAAAAGGATTTCAACATGTTAAAACAAATATATAAATACACAACAAATTCGTAAAAAAGACTATTCATTGGCCAAAACCAAAATACAAAACTTCCAATCAAAACATTTATATATATATATATATATCTTCGATGGCTTCTCACATTGTCCAGAGGTGATCTCTTGCCAACTTTGTAGTACTTAGTATTCTCAATTGATGATTAGTTTCAGTTTGTATGGTAATTCTCTGTCCTAATTTTAAACTGTTTATATAAGAATGGATTTTTATGATAACAGGAAAGTGAAAAGAAGGCGTAATGGATCTGATTCGATAGAAGAGATTTTATTGAGATGGAAAAATTTCAATCAGGAAGTTAATTGGAACCATGAACAAGTGAAGAAGAAGAGAAAATCTCCGGGTAATGGTTCAAACAAAGGTTGTATGCCAGGCAAAGGCGGTCCTGAGAATTCGGGTTGTAAATACAGAGGAGTGAGACAGAGGACTTGGGGTAAATGGGTGGCTGAAATTCGCGAGCCTGTTTATATTAGTGGTCAGTATAAGAGCAAAGGAAAGCGACTTTGGCTTGGTACTTACTCGACTGCTGGTGATGCTGCTGTTGCTTATGATGAAGCTGCTAAGGTTATGTATGGATCCAATGCCATACTTAATTTCCCCAATTCGAGTAATGGTAACATTACTAGAACATCGAGTGGTCAATCGTCGATTGATCATGAAGAGTCGGTGGTTGATGATGAGAAGAAAACTGAAATTGAGTCGGATTTGAAAGATGATGATGGTGGTGTTGTTGTAAATATGGATTTAAGTTATGATTATGCGAATCATGGTTCGCCTGCTTGTTCCTGGACTGAAGAGGAATTGGAAGTTATCACGGAGGAGAATTCCGAGATAGAATTAACCAATTTGGAGTGTGATTCAAGATTTTTTCATAAATCTCATGTTAAAGTTGAGAGGCCGATAATGGAAGAAGAAATTGATGAGGATGAATTTGTACATAACGACGTCTCAAACACAATAGACGTGGAGCCAACCGTCATGTTTAGCAAAGATGACTTCTCGAGGCTTGATGAAACTCGTAATTCTAATGATCAAATTGTGCTACAAGACATGGATTTCAGATCTTCTGAAAATCTGAACGAAGATGTTAGTACGCGTCTAGAATACATGGAGCATTTTCTAATGGATGACAATTGTTCGATGGAAGCAGCAAATATATCAGACATCATTTGTTTGACAGAGAACCATGATGAAGCTTTCGATTTTCAGAGGTTTTTAGAAGAATCGTTTGATTTCGAGCTAAACTATGCGAAGAATGAGGAGCAATTTGATTGTACATATGCATACAACCAGCAAATTGACCACCAGAATTCAGAGACAAATTTCGAGATTCGATCAGATGGAATTCGGAAGGAGAAAAACTTGCATGGTTTCGGATTAGATGACTTTGGAGCAAGCAATAACCAGAGGAAAATATAG

>SlERF5-11

TATGTAAAAATTTAAGGAATCCCATAGTGTAATTCAATAATTACACTCTGTCTCGACAAATTTAGTATTTACTAAAAATCCCTTAAGATTTTTGTGCCGTGATACTTTAGACTCTAGAGATACATGGTAAATGATACATGGTCAATTTAAACTGTTAAGAAAAAATGGGGAAAAAACGGTACATTTAAACTTGTTAACTAAAATAGCTTAATTTACGGTAACAAATCATTAATCAGCATTAAATTAGCACGTAATTGAATATTAATTTCAAATTTTGAAAATAAAAGATTATATCATCTATTTTGAATACAATTTTCAAAACCGCATGATACATAGAAAACATTATGATACACAACAAATTCATGTATCAATGCATCGTCTAAAACACTATACACACTGATTCAAAATGTATCAGCAAGCACACTTGATGGATTCAAAATGTATCAATGCATCATCTAAAACACTATACACACTTGATGGATTCAAAATGTATCAGCAAGCACACTGATTCAAAATGTATCAGCAAACACTTCATGTATCAATGCATCGTCTAAAACACTATATACACTGATTCAAAATGTATCAACAAGCACACTTGATGGCGCAGTTATTGAAATTAATCACTGATATATAATAAAAATTTACGGTAAAAATTGGGAATAATTTCAGATTCAGTTAGTAGGATTCTTTTTTTAACATTAAAGACAAAAAAGAATAGATTGATTTTTCAAAGAGTTCAAAACTATAAACAAATATATATAATTTAAAACTCTTTCATAGAGAAAAATTAAGAAGAAAAATTGGGATCGTCTGTGATAATTGGGTCGGCATCGACCCGATTTTCAGCAAAATCATCAACTTGGATTTGATCAAGCCCAAGTACATATCCCATGGCAAAGGCACAATCAAACCCAGGCTTTATTGAGAGAGAGAGAAAACGTCTTTTCCAAGCACCACATGAGCACAAGCATCCAATTTTCAGAAATTTCAAATATTTTGAATTTTGAAATTTAAAATTTCATATTAAATGTTGTGAAACATTTTTAATTAAAATTCAAAATTCAAAATTCAAAAAGTGATTCAGAAGATTGAGTGTTTTAGTGGAGAAAAAATAGGCATGATACGGGGAAGTGTGTGTTATAGGAGAGAGAAAATTATGTATCTCTAAACTTCTACTAAAATTAAAAAAAAGGAATTATGTAATATTTAAAAAAAAGGGGAGGGGAAAATTAGAAAATACTAAACTTATATTTGTGTATTTAAGTTATTTTTCCTAAAACATATTTGTGTAATTGATATCATAGAGTAAATGGTACCAATAATGGGCCTAGTTTACTTTGTCCCAATAGCAAGGAAGATATGCATTGGCCCAATTTCTACTAAGTGTTTTAAAAGGGGAAAGTTATCATCTATTGCGACGCAATGTTGAGAAACGTGTTACCTTATTCTGTTTTTATTTATATGATATTATTTAAAGTATCATAAATAAAAAAATTAAAAAATAATTGACTTGATACAATTTTAAACTTTTATATGTTTAGAAATAATTTCACTTAAAGTTTTAAAGGAGAAATGCTAATTTTTATTAAAAAAATAACACTCTTTTAAAATTTACTAAAATAATGTATTACAACAATTTTAAAAGTTATACATTAAACACATCGACAATGCATTATAATTTTCTATTCATACTTATAAAATTATAATGAACTTGTTAAAATTCTTCTTTCTATTTAAATTTGTTTGTCTAGGTATTTTTATTAATTTATTTGTTTAAAATTAAAATGGTCATTGAGAGGTAAGTGATGACATGGGCCTCTTATCTAACGCGTCAAAGCATTCCGGCAAATTGATAAGTGTAACACATGCACGTGTGGAGATCGTATCTTATTGGTCCCACTTTATCCTTATAAAACGATCGCTAAAGTGTTCGATATTTATATTGAAATTTTTCTTTTTTTATTTGGTATTTAATAGTGAGATGTTCTTTTAAAATATAAATAGTTTTATATTTAAAAGGTAATTTAACAATGAAAAATATTTATTATTTCATCATAATTCAGATAATTATAACAAAAATGATCTATCAATGTCATCACACAATTCTTATTAATTGAATTTATTCTTAAATATATGATCGTGAATCAATTTATTATTCAATTAAAAATATTTTATATAAATACGATCTTTAACTTAATTTTAGCTAATACATACGATAAGTAGTCAAGTGGTCAACTTTATTATCAAATCAAACATTTAAACAATGATCCAAAAATAATCTTTTACACTTAATCCTTCATCTTGTGGGCTAAACAAATACAAGGTGTATATATTATTAATAAAATATTGTTTGGTTAATTGTTTTTTTTAAACGTCTATATCCTTAAGATATTGTTGGCTATTTTGTATTACTTGATATTAAAATATTTAGAATAGCAACTTTAATGAACTTGCATGAAACAACAAAATTTGAAGAAATATTTTCAAAATCAGTAAAAAAAAAAAGAATATGAAATAGATAAAATAGAGTAAAGATATCAAATGATGAAAATTGAGTCTACTAAATGTATACTGTGTTTTTAAAAAAATTATTCTCTTAAAGTATTTGAAATTAATGACATATATCTTTCTAGAATAAAATGATCTTACTCAACTGTTCAAAATATCTATAAACACAGCATTAACGAACCACTCAACTCTAAAAATACTAAAATTTGTTTTTTGCAGAATAAGAAAAAGTTTAGAAAATTTATTGTTAAAAAATGAGAGAAAATCTCTTTATATGTTGACAACAAATAGTAGTATAAACATGTGATTTTTGTGCCTTATCGGAAAGCCCAAATTTTTAAAAAGTTACGGCTTTTAGAAAAAATCACAACTTTTCAAAACAGACACACCCTTCGGAAAAGTCACAACCCTTCACAGAATTCACAACATTTGAAAAAATTCACAATCCTTCGAGAAAATCACAATTCTTCAGAAAGTCACAATCTTTCAAAAAAGTCACAACTTTTCAGATAGTCACAATCATTAGAAAAAGTCACAACTCTTCAGAAAAATTTCAAACCTTCATAAAAATCACAATTTTTTTAAAAAAATCATAACCCTTTTTCTGAAAAATCATAATCGTTTGAAAAAAATCGAAACTCCTAAAAAATAACAACTCAATCACAAAAATTAGAGCTTTAATACCTTTCAAATTCCAACAAATACATCTCAATTATTACATCCATAAATATATATACACACATCTTCTTCTTTCTTTTTTCAATTACCTATTCTCACAATGATAGTCTAACTATCAACCTAAATTCAACATGATATATTATTCTATGAAATTTCTAAACGATAACTGTCACACAAGTAACTTTTATCACCAAAGTCCATTAATATTTTTTGGTGTACATCTTCAAAACTCCAAATATGAGTAATATCTACTATTGCACTAAGTTAATAAAAAAAAATTACTCTTATTTATAAACATACTTCAAAAATCAGTTATCAATACATATTTAGAATATACAGATCTACCCTCTTCTCGATTAGCTTATTACGTATTCGGGCAGCTGTAATCTCTTCACTAAAACAAATTAGATCAGTCTAGAACTGAAAGTGGTATAATTGTAAATTCATTGAACTCAAAGGTCAAAGGTTTAAACAAAACATGATTTCTTCTACCTAAAGAAAAGTTGAAACCAAAACGTTCTCGAATTTTCTGCTGACACGTGTCGTGTCCCGCGGTAGTCAAATAGCCACGATTCAAACAAATCTTTGCCACGTCACCACATTTAGAAACACAAGATATTTTATAAATAAAATAAAATATCTGCGGATGTCTATAAATACCTGTTCAGTTTTCCTTATTTTCTCTGTTTTCTTTTCTGGAAAAAAATTTTAACTCTCAAGAAAGTACTGGAACATACCAAAATCACATATAAGGAAGGAGGAGGAGAAATTAGTGTATTCGTGAAGCGGAAAACTTCAAATAATTGTTGATTAAAGAGATTGTCAAGTTTTGGAACTGTATAATTGTGAGGTGAGTTCATTTCATCCTTGATTTTTTTTATTGCAGTTTGAATATATGTTCGATTACTCGTTGGAGTTTTTTTTGAGTGTATGTGAGAATTTTGTTTGTTTGTTTGTTTTTTGAGTGGTTTGACGGAGATGACATCGCTTCTTAGGGTTTTAATTGGATGATGGAATTGGCATTGCGATTTCATGTTTTGTGTTGTATTAATTGAATTGATGTTTTGTGTTGTAGTTATTGGGTTGATTTGTTATTTATCAAATGAAAATGAGTTATGATCTCAAAAAACTCACATTTGAAATTATCTGAACGAATTTGTTATAAGAGGAATTGAATCGATTTGTTAGTTCATAGTGTGAAATTAGATTTTCGAATGGAAGCAATTGGTTTCTTAGTGGCGGAGAAATGAGGATTATTTTTTTTATCTAGAAAGGTTATCTATCTTCTGTATATGAATTTTTTAAAATTAAGAGAGCGAATTGAATTTTTAAGACTGATTTTGAGAAACCAATTCTGAATTGGTAAGTGATAGTGTGAAATTAGTCTTTTGGAGGAAAACAATAGGATACTTGGTGATGAAGAAAAGGGAGTTATTTCTGTTTAGGAAGGTTTACTTTCTTCTCGATCTTAATTTATGTGATAGTTTCGACCATACTCGAGGGATCTTTTGAGTAATCAACACTGAATTGGTAAGAGATAGTGTGGAAGAGGATTATGAACTGGATTTTTGGATGGAAGCAGCAGGTTACTTGGTGGCGAAGAAGAAGAATATTTTTTCTCTATTTTATTAGGTTACTTGTAGAGAAAGGAAGTATTTTTTTTTAATTGAAGGCTGCGGCTTGACTTGTTTGCGAAGGCTTTTTGCTGCTAGCTTGGTTTATAAATACTAGTAGTAGTAACATAGTTGACATTTTTTCTCTTCTTCTTGACTGACAGAAACAGATATATGGCTGTGCTTGATCGAACTTCAAATATGATGTTACCTATGGATTATACAAGGAAGAAGAAATCAAGGAGTAGAAAAGATGCACCAAAGAATGTAGCGGAGACACTTGCGAAGTGGAAAGAGGTGAATGAGAAATTAGACGCGTGTGATGATGATGGAAGGAAGCCAGTGCGTAAAGTTCCTGCTAAAGGATCAAAGAAGGGATGTATGAAGGGTAAAGGAGGACCGGATAACGGGCGGTGCAAGTATAGAGGTGTCAGGCAAAGGACATGGGGTAAATGGGTTGCTGAGATAAGGGAGCCACATAGAGGGAGAAGGCTTTGGTTGGGTACGTTTGATACGGCAATTGAGGCTGCTCTTGCTTATGATGAAGCTGCAAGGGCGATGTATGGTCCTTGTGCAAGGCTGAATCTCCCGGACTATTACGCCTCGTCGAAAGAGTCTTCCAAGGATGATTCTTCGTTGCCTACTGTATCTCGTTCCGACTCAAATACAGCATCAAGTTTCTCTGAGGTGTGCCCTGCTGGTGACATGATGAGAGGAAGAGCCAATGTTCCTGCGGCAAGACATGAAGATAGATCAATAGAAATTGACGGTGCTAGGACTGGTTCTAATGAAATTGGAACGCCTTTGAGCTCATTGAGAGAGGAAGCGGAAGACGAGACCAAGGAGGTGTCAGATAAAAGTGAGACTTTTACACCTCTGAGCTCGTTGAGAGAGCAAGCGGAAGATGAGGCTAAGCAGGTGTTAGATAAAAGTGAGACCTTTGAAATTAAAGATGAGCCTGCGGCTTGTAGTTATGATTCATGGGATATCGGACAAGAGGACCTAGGTAACTTTTGTTTGGATGATGAGATGTTTGATGTAAATGAGCTGTTAGGCATGATGGACTCGACTCCAGTAGATGCCTCAGCTCCAAGTCAGGATGTTGGTTTTGTTCCTCCTAAGCAAGAGCAGTATGCCTATGATCCTTCATATCAATTGCATTCTGCTGCCTACGATGCCAATCAGTTGTCAAATCCAGCATACCAGTTGGACAATGCCGATGACCAGTTCTCAAATCCATTATATCAGTTGGATAATGCAGGTGTTGATACATTGGAAGGTCTGCAGCAAATGGAGCAACAATCACCTATCGAGGTTGATTATGATTTTGATTTTCTGAGACCAGGCCGGCAAGAGGACTTCCACTTCTGCTTGGATGAGTTGGATGTCTTGGATTTCTAA

>SlERF6-2

ACACTACAATGGACCAGATAAAATAATTGTTGAGAATGGATCAAAGTTAGCCATAACACATGTTGAAATTATATCTGGAACATGTCTAAAAGTTAAAAGAAGTTCTTGAAGTTCCTAAGATCAATAAAAAGAATTATTCTCAGTTAGTAAGCTTGCAAAGGATAATCGTTGCATTCTTGAATTTGATGAAACTAACTTTGTTGTAAAGGACAAGAGGACACTGCTGGTCAAATGATCTAACAGAAATAAACTTTATGCTTTGAAAGATAATAATCTCTATGCTTTAAATGTTGCACACAATTGAAACACGTCAGACAACATGTAGCACACTAGATTAGGACACCCTAGTTTGAAGTCTTTAGAAATTTTGAGTAGTAATAGATGCATCAATATTAGTAGTTGAAATAAAAATCCCTATTGTTTCTTCTGATTGTTAGTTGGAAAGAGTTGTAAACTTCAATTTGATTTGAGAAATAAAATTGAGTAAGGTTTGAGAACTAATATTGAGAAAGAACCTTTATTGAAAATTCATTGTGACTTTTGGGGACCTAATCTTGTCAAATCTTCTAACATATGAAATATTATGACATTTATTGATGATCACACAAGATATACATAGATTTATTCACTGAAAAAGAAATCTGAATTCATTGAGGTCTTTCTAAAGTTTCAGAGGGAAAACAATTTTCTAAAGAGATTAAGATTTTTCAGTGTGATAGAGGTGGTAAGTTTATCTAAACAAATTTCATTAAGCATTTGGAAGATTTGGGATTGTGAGATATATTTCATGTCCTAACACCTTGAACAAAATGGAGTTGCAGAGAGAAAACACAGGTGTATTGTGGAGAGTGGCTTAACTCTACTACTTCATGCTAATCCACCTTTGTTTCTTTGGGTTGAGGCTTTCCTTACTGCAGTATTCCTCATCAATAGACTGTCTTCCTCAGTCCTAAAGATGGTGACTCTATTTGTTAAGTTGTATGGGGAACAACCTGATTACAATAACCTAAAAGTGTTTAGGTGTAGATGTTTTTCATATATCAAGGGTAGCAACAAGTTTAATCTAAAGACTTACCCTTGTGTGTTCATAGGGTACAATAACTTACACAAAGGATATAGATGTTATCATACCTCTACAATGAGAGTTTACATATCACACATGTTTTGTTTGATGAAAACACATTATCTAGTGTGTCTCCAAAGCAATCTCAGACTAACATTGATGTCTCACCATATTTTGCTACTTTTGTTAAATCTCTCTAAACTGCAGGCACATGATAATTCTGATTCAGGGAAAGTACAAACTGCTAGTACAAACATTAATAGGGATAATGCCGCGACAACTCATATAATCATTAATGATGAGAGTACTATTAGCCTCTCATGTACATGATCAGATGTTAAGGAACCTGTTGAAGGTGTTGATCCGTACAACTCTATTAAACCTCCAATTGTAGATTTTGGATTTGCTAGTAGTAGTGAGGAACAAGTTGCACTTACTGTTCCAACCAATGAGCATCATCAAACAACTACTTTAGTTGATTACTAGTTAACCATCAATCTAACAATGCTATAGTTCATCAACTACTTTGGTTGATGTACAAGTTAAGATACTCCTTGCACCACAAGGGCATCATATGATCACCAGGCACAAGGCGAAGAAACATCACTTGTCACTTGTTGCTCGCAGCAGCACAACACTTTTAAGAGAACCAAATACTATTAAGGAAGCACTAAAGTCACCTTATCGGCTTGTAGCAATACATAAATAAATTGATGCTCTACATACTAACAAAACATGGATTTTGGTGCCCAAATCTTTTGGTATGAACTTGATTGGCTCAAAATGGGTGTTCAAGACAAAATTAAAAGTTGACTGGACAGTAGATAGATACAAGGCCAAACTTGTGGAAAGAGGATTCTCGCAACTTGGATGGATATATTTTGAGGAAATATTTAGTCTTGTGGTTAAAGCCACAACTATTAAGGTGGTTCTATCCATTGTTGTTAGTTCAAAGTGGGAAGTTAGACAACTAGATGTCAAAACTGTCTTTCTCCATGGCTTCTTACTAAAGGAGGTGTACATGAGTCAACCTCCTGGTTTTATTGATCCCTAGTACCTTCAACATGTGTGTCTACTTAAAAAGACATTGTATAATCTTAAACAAGCACCAAGAGCCCAATTTGATAGGTTTAACATACATCTCTTACACCTTGGTTTCATTTGTAGTAAGATTGAGCCTTCCTTATTCACTTTTACAAACTTGTAAAGGCAAAATATTTCGCTTGCTATATGTGGATAATATTATTGTCACAAGAAGTAATCCATCTCAAGACTCAGAGTTGCTTCTATAACTTTGGAAAGAGTTTGCTATGAAAGATCTTGACCCTTTACACTTCTTTCTCGGAGTTGAGGTGAAGTATTTCGAAGAAGGAATTCACTTAAATCAAAGCAAGTATGTTGTTGAACTGCTGGCCAAGACAGAGATAACTTTGGCAAGGCTGTAGCTACTCTTTTGGTTCAAAAGCATGGTCTGCATGAAGCTGTGGGAAGTTTTATAGATGTCTAACTTTATAAAATGATAGTAGAGAGCCTTCAGTATTTGACCCTCGCATGACCTGATATCATTCATGTTGTGAATTTAGCAAGCCAGTTTATGCAAAGCCCAATTTTAAAAATTTTCAAAGGGTAGAAAAGATTCTCAGGTATATCAAAGATACTTTACACTTGGGACTCAAAATTATTTCACAATAACCGTGTAGATTGTATGACTACTCATCTGATGATTGGGGAGGTTGTACCACCACTAGGAGATCAAGTATGGGCTACATCTACCGAAGTGCAAATTGTATTTCTTAGACCTCCAAGAAACAAACCATAGTAGCCCGATCGAGTGTTGAAGTCGAGTGTAGAGCACTAGCCTTCACTGTCGCAGAATTGACTTGAATTTTATATATTTTGCATGACTTTGGAGTGTTCCTTCGATTTGTTCCTACGTTGTATTGTGATAACATAAGTGCCTTGTACATGATAGTTACTCCAGTTATGCATGCTAGAACCAAACATGTTGAAATGAACTATCATTTTGTTTGTGAGAAAATGCCCAAGGGACAACTTCTTACTCAATTTGTAAAGTTTAAGGATTAACTAATTGATATTCACACAAAGTCTTAACAAAATAGGTTTTCTCAGGTTTTTGCAGCAAGGTAAGAGTTACAGTTCTTTCACTCACTAGATTGAGGGGAAGTGTTGAAGGAAACTTGAAGACTAATGGAACATGTCGAAGGAACAAAGAAGCAGGCTGACGAGTTAGAGTTAGAGTCATACTAAGATTAGACTACTAAATCCTAAGTTATGTACGACTAAGCTACTGTATATTGTAGGTTTCAACTAGGATAAGGTATTTAAGTTATAATACGACACTACTCCTGTAAATAATTGTGCTGTAGTACGATTCCTAATATAACTAAAATAGAATTTTTTTTCTTATATAAGTAGTTTGTAACTCAATATTATTTTCATCCATACATCCTTTATTTTTTCATACTTACGTGAGATTTCTATAATCCAAAATAGTATAGTGAACAACTATTATTTGACGATGAGAATATTTGTTGGTGATATACGATGCTAAATTTATTAACTCAAAACGCATGAAACATTATTTAGTTTTAAAACTTGAGTAATGTACATTAATTTGGTGTCTTACCTATTATAGGTTCAAATTCAAAGGTATAATTGTTAGATATGAAACCCATATGGAACACTTTTTTTAATTAGATTAGATTTTACTATAAATATATCGAATAAAATGAAAAAAAGAAGAAGATAATTTGGTGTCTTTGTAAGATGTGCCAACTTATTGATCCATGTGATTCACTAAATCAAATGTATGTGCTTGCAACTTGCAAAACTATTGGCACCGTTAGTTTGGTAACCAGTTTTAGGCCTCCTGAGTCCTCATAAATGATTTTAGACCAATCGCGAACCGAGGGGCATAAAGGTAATTAAGCTGATTATTTAGTTTTACTGTTTCTCGAATTATTTGTTTACTTTTAAATTGACATGCTCATTAAAAAAGTAATTATTAACACAATGAAGTTATAATTTTATCTATATTAATTATGAAAAAAATGAATTAAAAATTCAGATTTTTTTAAAAAAAATTTAAATAATTAATTGAAATTATATTTTGTTCTAAAAAAATAAATAAATAATTATAGAGAGGGAGTAATTAATTAAAGTAAATATAGGAACTATTTTACAAAAAATATAGAACGAGTGGTTCGCGAAGGTTCACGTGACACGTGCTGAATTCCGCGGCATCCCAACAGCCACGACTCGACTAAATAATTGCCACGTTACCATATTAAGAGATTTTTAAATAAATAGACTCCCTATTGTCTAATTTATTTGATTTACTTAAAAATGTCTATCACTTATTTTATTTTCGAGAGATAGTAATTTTGTAAAAGAAAATTTATAAACTTATCTATCAAGTTACATTTATTTTAGAAAATTAAAGGATATAGTATAAAATGAGATGAAGAGAATACAACACGAAAAAGGAGATATACTAGAAATATCTAGAGAAGGCTTATAAATACTCGAGTAGCTCTGTTGCTAACAGATTTCTGATTCTAATAATCCTCTACAAGCTTTTTGTTAAGAGACTAAAAAAAACAAAAAAACCTTGAAGAACTCACCAATTTGATCAATTCATTTATTAAAAAAAAAACAGAGAAGAAAAGAGGTATGTAGCCTAATTTCATTCATTCAATTTCATCTATATGTGATTTAGTTTGGTCAGATCAATCTTTTCTTTTCTTGCTTCTTTTTAATGTTATCTGAACTTATGTTTAAGTACTTCTCTTATCTTTAGAGTCTTGTGTTTCTTGAAATGGATTGTGATATTGCTCTTAATGATGAGTAATTCTATGATTGTATAATTAATTATAGTTGTGGGGAGTAAAATGATATGTTTTGTCTACTTACAGAAATGGCTATTATGGATGAAGCTGCTAATATGGTTTGTGTGCCGTTGGATTATAGTAGAAAGAGGAAATCAAGGAGTAGAAGGGACAGAACAAAAAATGTGGAAGAGACACTAGCTAAATGGAAGGAGTATAATGAGAAACTAGACAATGAAGGGAAAGGGAAGCCAGTGCGTAAAGTTCCTGCTAAAGGTTCAAAGAAGGGGTGTATGAGAGGTAAAGGGGGACCAGAAAATTGGCGGTGTAAATACAGAGGTGTTAGACAGAGGATATGGGGTAAATGGGTTGCTGAGATTAGGGAACCTAAAAGAGGTAGTAGGTTATGGTTGGGTACATTTGGTACAGCAATTGAAGCTGCTTTAGCATATGATGATGCTGCAAGAGCTATGTATGGTCCTTGTGCAAGGCTTAATTTGCCAAATTACGCGTGTGATTCTGTTTCCTGGGCAACTACATCTGCATCTGCATCTGCATCTGATTGCACCGTTGCTTCTGGTTTCGGCGAGGTATGTCCGGTTGATGGTGCTCTTCATGAAGCTGACACACCATTGAGCTCAGTGAAAGACGAAGGGACCGCGATGGATATTGTTGAACCTACGAGTATTGATGAAGATACGCTTAAGTCTGGATGGGATTGTCTAGATAAATTAAATATGGATGAGATGTTTGATGTAGATGAGCTATTGGCTATGTTAGATTCTACTCCAGTTTTCACCAAGGACTACAATTCAGATGGAAAGCACAACAATATGGTATCAGATTCGCAATGTCAGGAGCCGAATGCAGTGGTAGATCCTATGACTGTTGACTATGGCTTTGATTTTCTGAAACCAGGCAGGCAAGAAGATCTTAATTTCAGTTCGGATGACCTTGCATTCATAGACTTGGATTCTGAACTTGTCGTTTGA

>SlERF12-4

TTAAATTAGCGAATATTCGTATAGTTTGAGCGATCAATAGTCACCTATCAATTAATTAAAGTTAAAATTGCTGAATAAGAAATTATAAATACCAAAATTAAAGTTTATTGGTATCTAATAGGGATAGGCATAAACACCGGAAAACCAAAACATTGAATCGAATTTTTTTAGAATTTCGGTTTTGATATTTCGGTATTCGATTCGATATACTTTTTTTATATTTTTCGATATTTCGGTTCGATTTTTGATATTTTGATTTAAAACAAAATATTATACTATAATTTATATTATATTTATATTGTATTAATTAATAATTATTAATGTTAAATAATTTTTTTTTTAAAAAAGAATTTGAAACCCTATGTAATGTTCCTGTTCCTAATTTAATTTTTAATATTCATCTTGCAGCCTCCTCTTCCTTTTGCAGGCAGCAGCTCTTCCTCTTCTTTCTCTTCCTCTTTCAGGCAGCAGTAGCCGTCGCCTGCCAGCAGCAGCAACCGCCGCCAGACACCAGCAGCAGCAGCCTCCGCATGCAGCGACTTCAAGTCTTCAACCGACGATCCAGCCCAGTCAGATATTCGATACACAATTAATAAAAATCAAAATTAAAAAATTAAACCGAATTAATAAAAATCGAATCGAAATATTTTAGTTCGGTATTCGGTACATAATTTACAAGAACCAAAAACTGAAAAAAAAAATCGAACCGAAATATCGAATGTCCATCCTTAATAACTAAGAAGCTAAAAGTACTAATATCGCTTTATTCTTATATAATTTAATGAGACGATTTTGATCCTCGTAATTTTAGTTTAAAGATGTTACACCTTCATTATGCATTCTAAATTCTTCTATGTATGAATTTTCACTAAGTTAATGAATTCTTAAGCGTTAATCATTCAATTATCAATATAATTACCTTTAAAAATTACTTTGTATTATTACATATTTGAGGATAATATAATTCTAAAAATAATTTAAATATCGTATACTTCTTACCTATAGCCAGGGGAGCCAAAAATCATACATTTTAGTAAATTAAAGGTTAAATATGCTTAATAATATATCATGAAGATCAAATTAGAATTTATCCATAATTGAGGGACTAGAAGTGTAATTATCCCTTCCATTAATATGTGAAGTGACAAAGGACCCGTTTGGATGGGCTTAATAAAAACAGCTTTAAAAAAACATTTTTGAAAGTGCTGAAACTTATTTTTAAAATAAGCTATTATGCGTTTGGATAAAAGTGCTGAAGTTAAAAAAAGTTGTTGATGTGTTTGGCAAATAAGTGCTGATAAACAGCTTTTTAAATCAATATGTCTGAAATACCCAGTTGTTAACATAATAATAGTTAATTAATTTATATTTTACAACCAATAAATAATTATATTTTGCTATCATTCACATATTTCTTTCTCATCACAAATTATTTATAAGAGGAATATAAACTTATTATAGCTTTTAAAGATATATAATTTGAATAGATTAAAGAACGATTTAAGATTTTATTTTAGTTTCATCTATAGGTAATAATAATTGTCTATCATTCACATATTTCTTTATTATCACAAATTATTTATAAGAGGAATATAAATTTTATTTAAGTTATATGTGCAACTTATTTTACATTTTAATAATATATAATTTGAATAGATCACTTGAAAGATTTAAGATTTATTTTATTTTCATTCATTAATAATAGTAATTGTCTATCATCCACACGTGAAAAGGGAAAAATAGAAAAAAGGAGATGTTAGGGTTATGTGGGTAATTTGGAGATTATATAAAAATATTAAGGGCAAAAAGGTAAAAATGTGGTTAACTTAAAACAACTTATAAGCTGGGAAAAAAAAAGCACCCTACCCCAACTTTTAATTTTTGGCTTAAAATAAGTTTTTTTAACTTAAAATAAGTTGTTTTGAGTATTGCCAAACAGCTAAATAAGTCAAAAACCAGCTTTTAAGTTAGTTTGACCAGCTTTTAAGTTGAGCCAAACAGGCTCAAAGCCTAATGAATTTTTCTAAAGATGCATTTTAACTTGATTGAAATGTTTCAACTAATTTACTACCTAAAACAAAAAAACTTGCTATTATATATAGAGAGAGAGTTAGACTTTGTATATTACCAAATTTCATTGCACTATAAGACTTACTGAAATGTATATATATTTTGAGGTTCGATAATTTTAAAATAACATTGAAAAGTTTTATTTTGAAATAAAATAATTTCTACTAAAAACAATATTATACTTTAAATTTGAATTTAAAATATTATATATTTGAGCTCAAGATATAATATCGTTACTTTCCGTTTAAAAATGATATTACTTACTGTTCTATCACAACTTGTAATATTTAATTAATGTAATATCACTTTTTTTTTTATTTATCACATGAATGTACTCCTCTAAATGAAGTTAAAAATATAGTCTATGTTATGAGTACGAAGCTTTATAGTTGATAATAAATGTTGTACCAAAAGTCTTAAATTTTTGATAAATCAATATTGTCTTTTTCTTTTGGTACAAATTCTAAGATTATTAATTATTATCTGAACTATTATACTCATTTTATTTACCTTTTTATAGATAGTTAATAATATTCAAATGTACTTATTTCCAAATCAACTTTTTTTATTTTTTGTACTTTTTATTTTCTTTTTTATCGATTGTGCTTTATTTTATGTGATATATTTTCTCTTTTTTAATTTGTTGCAAAAAGACAGAAACGTTTTATATTTATAAATAATTTAATTTTAATTTTTCGCTTTGATTCTATACATTCATAAAGTCTTGGTCATATAGATTGCATCATATATTTAAAATTTTATGTTAACAAAAGTTTTTTTTAATGTGTGACTTATTAAAAAGATTCACGTATTACTGAAACGAATCGAGTACTCTTCCCGTCCCAAAATAAATATACTTTTTTTCTTTTTAAGAGTTAATTTTGATCAATAATCAAAGCTTAAAATTAATAGGTTTAAAAATCAAAATTTAGTCATTCAAAAATTATATGAGATATATTATATAAGTTGTAATTCTACTCACGATCGACAATATAATTTAACAAGTACATTTAAAACTATCGATAATCGATAATCTTTCATATAATTTAAATTTCATCAAAATAAAGACGATAGATAATTTGAAACAAAAGAATCTAATTATAAAGCATAAATTATTAATAAACAAACGCATAAAAGTCATGGGTAGTTGGATTATTTGTTATCTTGAGCCTCATTTTCTAACCATTAATTGCTAGTCAAAAAGCAAAAGCTATTAAACTAGAAATATTGTAATTATTTTGAGAGTATGTCAAAACCTAGTCAAAAGGCTATTACCAAGAGAGTGTTATCTTTTGTTACTTTTATTATTGTAAATCATAAAAAATAATTACTTTAATTAAGTGATTATCCATCATTGTTAGCATTTATTTTCATAGTCACCAAATTTTCAAAATTGAACTGTATTTTTGAACTAATATATAAGTACATTTACTTTTACTAAAAAAAGAAAATAAAGACTTGTTTGTTCTTGGCTAAAAGTATGAAACTTATTTGCCTTTCGAAGTTAAGAAGTCATTTTCGATAGAAAATATCGTTATGAAATCGAAGAGAGAATAAATAATAACGTACTCACCGCTATAAACAGGAATTGATGATGCATAAATTATGTAACTAAATAAAAATATTCGTTGTTAATTTTATTTAGTAATGAATTATCATGACATTTTGCAAGTATCAATAGATTTTGTCAAAATTTTATATATTCATAAAATATCTGTATACGAATTTTTTGATAATAAACTCAATCAGTATTGTATTCCGTTTGATTTAAATTTATTATAAGAATTCATAAACTTCAAAATGTTAAGTTAAATTGTCTCTTATTATACATCAATAGTTTTTGAAACTAGTAATTGCTAGTCAAAGAAAAAAAATAACTTTTAGCGCTATTAAATATTGATATTAATATAGAGTAATAAAGTCTTTATCGGTATTAGTTAAGTGCCATCAAAACCAATATCGCTAAAGACTTTAGAAACATACAAAGAGTGACAATTGCCGCTAAAAATACATTTTTGATGTAGTGTAGTCAAAGAACAAAACATTGTACTAGAAGTATATGCAACTTAACCCTAAAAATATGTGTTTTTGGTTACTTTTAATTTGTAAAAATTAACTATATAATTGATGATGATCATATTATTACTTACTAAAAGTATATGCAATTTAATTCCCTAAAAATATTTGTTCTTTATACAAATTTTTATTGTTACTTCATGTTTGACGATAGTTATATGACTCTAAAACAATGCAAAGCTAGTATACATGGGCGGATCTACCTAGCTTTCGATCGACCCTACGTTACGTAAATTTAACAGCTTTTATTAAAATTCTATATTTTATTATTAAAAATAGACTAACATTTTTAAAAATTTCTTTGTTATTAATCTATCGCTAAATCTACTCATATTCAGCATAACCTTTTGATCAATCATAAAAGAATTAAAATTTTCAAAAGTTTTGTTATTATTTATCAGTCGCTAAATTATTCGTATTCAGCATAACCTTTTGATCAATCCTAAAAAGAACTAAAATTTTCATAAGTTTTGTTATCATTTATCAGTCGCTAAATTACTCGTATTAGTTCCGCTAACACATAAATGTATTTAAATATAAATTTATCTATTATTATATATTAACTTAATATAAAAAACAATAAATTTCATCTTTATATTTGTTACAATATTTTTTAGGTGATGTTGTTAATTGCTCTAGCTAGTACAAAATTAGTTCACCGACATATAAGTGTATGGTTTAAAAACAATTGGCTATATATAATTTATGTGGACATTTGTAGTTTTGCACAAAGTCATATCATATCATATTAATTCATTTAGCAACTTTTTTTTAATTATTATTTATTTATATCTCTTCTTCTTCTTTACTTCACCAAAAATTCTTCAATTATAATTATCTTTTTATTAAAAAAAATGGTACATTCAAAGAAGTTTAGAGGTGTGAGGCAACGCCATTGGGGTTCTTGGGTTTCTGAAATTAGACACCCTTTGTTGTAAGTTATTTCTTTCGCAAATAATTTTTTTTTTTAATTAACGAATCAGAATGATAAAAGAATCGTATCTAAATATATCGTGATAGAATAAAGATTGCTTTGCCACTTATATGAATATACATTTTTCATATAAATATATAACATTTCTCTTTCTCAATTTCATTGCTTTTATCTTAACTTTAACTTATCAATGTTACATTGTTGCTATCAGAAATCAATCAATTTTCGTTAGAAATAACTTCGTTGATTGCTTTATTTTTTCGATAGATGATTGATCATTTCTATAATATATTCACTCATAACAGTTACTTGAATATACAAAAGTCATTTTCTGACGTAAAAGTTTAGTTTTGTAGAATCGTAATAACTTTGTCAGAAATTTGACCTTTCAATCATTTGTGTTTTTTTTTATCTAGTTACTTTAACATTATAAAAAATAAATTTCCTTACTTTATTGTTATTATAGTGAATAAATATCAATTTTTATTTTATTTTTTTCCTTTTCTAATTATAATATAAATATATATGTGTTATAGGAAAAGAAGGGTATGGTTAGGGACATTTGATACAGCAGAAGAAGCAGCAAGAGCATATGATGAAGCAGCAATATTAATGAGTGGAAGAAATGCAAAAACTAATTTTCCAATAACACAAGATTTGGATAATAATAATAATAATAATAATAATAATAATAATAATAATAATAATAATAAAAATGTAAAGGGTAAAGATCAAGAATCATCATCATCATCATCATTTTCATCACCAAAAGCTTTATCTGAAATACTTCATGCCAAGTTGAGGAAATGTAGCAAGGTGAGCACCCTTACACCTCCATCTCCGCGTCCTCATTTTGTATTTCCTTTTTTTCATGTTTTAAAAATACGCTTTTCTGATAGGTTTTCATTAAAAATAGACTTGTCAGATTTGAAAATATACTTTAGTTACGTTGGAAATCTTAGCTTTTGAGAGGGATTCTAATAAAAAAGTATATCTTCGAAATGTGCTTGTCGAAAGTTATTCCAACGTCCATTTTCTATCTGAAATTTCAAATTTTAGAGGTATTTTCGTAGAAATCACGTCTATCGAAAAAAACATGAGTTACTAATAGTAATTAGTCTATTTTCTATTATGCTATTTTTCTTTTGGATAATGCACAAGTATCCCCTCAACCTATGTTTAAAATCTCAGAAACACACTTATACTATTTTTCTTTCGGATAATGACATTACCCAACGCTGATTGACTTTTTTTTTCAAATTAGTATCACGTAGGTCGAAAAGGAGTAGAAAATTACTTATAAAATAAGTTCAGGAGGGTAATAAGACCTTAACATAGTATAACTGTGTCTCTAAGATTTCAAGCATAGGTTGAGGAGATATTTGTGCATTTTTTCTTTTTTTTTAGCATATTCTAAAAAATAATAACCTTTCTATTTTTGGAAATAATTTAATTTTTTTTATTCTAGCTTTAATAACATGACTTTGTAATTAACATAACAAGTTAAAAAATCTTTACTAAATATCATATATTATCCCTTTATTTTAAATGATGTGTCACTTTATGTACTTAATATTGTCACTATTAAAAAATCACTAAACGAATTTAACTTGTCGATAACATTTTCAATAAAATTTTATATGGAAAATCACTGACTAATCCATATACTTCAAACATATTATATATTATTTTTCATATAAAATATATTTCTAGTGCAATTAATTTATTTATTTTTTATATATGTAGGTACCATCACCATCTCTCACTTGTTTAAGATTGGATATTGAAAATTCACACATAGGAGTTTGGCAAAAACGTGCTGGCCCTTCTTCTGATTCAAAATGGGTAATGACAGTTGAACTTCAAAAGAAAAATAATCCCAAAAATATTAATGTTCATGAAGGAGAATTAAATAATAATAATAATAATAATAATAATGATAATAATAATAATTCCAAGAATAGTTGTGGTGAAATAACTATTAGAAGTGAAATGGATGAAGAAGAAAGAATTGCATTGCAAATGATTGAAGAATTGCTTCAAAGATGA

>SlERF6-4

ATCTACTTTCCCAAAGCTTCGTTCTTCCTCTCTCTCAATCGATCGTTTCTCCCTCTCTCTCTGTTCTTTTTCTTTTTCTTATTCAAACCCTCTTTCTTTTACCCTAATTAGCATATAATTAAGTATAAAAGATGATAAAATACTCCACTACTTGTTTCAAGGTTCTCTCTTTTAACACCCAAGTAATTAAATTATTAACATTAAACCACTAGCTTAATAATTATAAGCAGGAATAGTCCAAAACGCCCCTTAAAATATTTAACAGAAATCCGACCCAGTCAGGGTCACGCAGCCTGTGACGGTCCGTCACAACTGTGACGGTCCGTCCTGCACGTCCGTCACAAAGTTCAGAGAGTTAATTCTGCGGAAGGATTTGTGACGGTCCGTCGAGCCCTCAACGGTCCGTCCTGCCATTTCGTTGTGAAGTTTAGAGAGTCGATCTCAGTACCCAAATTTCTAAATTCTAAGTGTTTTGGAACGAGACACCCTCGACGGTCCGTCGTGCCCATGACGGTCCGTCGTGGGATCCATCGACCCAGACAGTGATTTTCAGAAATTAACTCTGCTGCTCCAAATGACTAAACAGGTCGTTACAATTTTAAATTTGTTGCGAACACCATATGATTAGAGCATAGGTGCATTTTGAACTTTAATCACTAATCTAAGAGATTATTATGAAGAACCTTACATTTGACCGAGTAAACGACTTTTATTACGAAGTGTTTACAATTTAGTGTATTTTCTTTTTGGGTAGTAGACAATTTAGTGTATTAATTAAACATAAAAAGGGTTGATTCAATTCTTACAATCTCCATTTTCCTTCTTGATCCCCATAGATGAATTTAGACAATTTAGGGGTTGTTTGTTTACTGGTTAGAGTTATGCAGATATTAGTTATGCAGGGTTTAGTTATGCAGGTATTAGTTATGCTAGGTTTAGTTAATGTAGGTATTAGTTATGCAGAGTTTAGTTATGCAGGTATTAGTTGTGCATGGTTTAGAGATTACAATAAATGAATTATTAACTATTTAATATTAAACTTGTTGTAAATTATTGATATATGTTATTTGCAAAATAATAATACATGCTAATAAAATATGAAATATATTGAATAATATATAATGCTAATATTTAATTAGAATATATACCGTTAAAAAATATAAATAAATTTCTAATATTATAAAAATATTTATATTTGCATAAATCAATCAAAATGGTAAGTATATAAAAAGAAATGAAAATAATCTATATATTATAAGAAATGAAAGCAACACATGTTGATGGAAAACAATAAAGTTTCTAATTAAAAAAAAATACAGTCTACCAGTAGCGGAAGAGAGGGTTACCATACATACAAGAGTCTTCCAAGAAATAAAAAGGGAAAAGGGTCAAATATGCCCCTAAACTATTTAAAAAGGTCTAGATATACCCTCCGTTTAAAGTTTGGTTCACTCATGCCCTCGCCGTCCAACTTTTGGTCCAAATATGCCCTTATGGGCGTTAGTTGTCATGTTGGATATATCCAACTCATTTTTCATTTCTTTAAATTCCACATTGAATTGAGATATCATTTTGACCTTATCACATAACATTTATATGAAATGGAAAGATATTCGGATTCATAAACATCTAATCCGACCCATAAATCAGCTCCTATTAAATTCACTATCCGACCCATTTTTAGCAATTTTGTTTAATTTTTATTATTGCAATAAATCCCGAAAATGAGTGATTGATTAATAAAAATAGAAAATATATGAAAAAAGTATAAAAGTAACGCCAAAAATTCACAAATAAATATAATAACCTTAAATCTAACATTTCAAATTTTTTTTAGATTTTTAGTTTTCGATATATCTCGAAAATAAGTAATTGATTAATAAAAAATATGAAAAGTATGAAAAAAATATAAATTAACACCAAAAGTTCACAAATAAATATAGTAACATTTCAATCTACATTTTTTTAAATTTTTTTTATATTTTTTGGCAAATCCCAAATATGAGTAATTTTTTAATAAAAGGGAAAAATATGAAAACAAAATAAAAAATATACGTCAAAATTCACAAATAAATATAGTTAACTTAAATTCAATAATTTCAATAATTTTTTTTTTTTTTTCTTCATGTATTCCTATTTTTTATTTCTGAATTTTTTGCGTAATTTTTATATTTTTTTGTTAATAAACTCATTTTTGGGATTTGTCAAAAAAAATAAAAATTAAAAAAATATTGAAATTGTTGAATTTAAGGTTACTGTATTTATTTTTTAATTTTTGCGTTAATGTTATATTTTTTTTCATATTTTTTATTAATCAATTACTCATTTCGGGATTTATCTAAAAGAAATAAAAAAAATAAAAAAAGTGTTTAAATTGAATTTAAGGTTACTATAATTATTTATGAATTTTTGGCGTTAATTTTATACTTTTTTCATATTTTCCTATTTTTTTTATTTATCAATTACTCATTTTCGAAATTTATCAAAAAAAAATAAAACAAAATTGTTGAAAATTGGTCGGATAATTTATTTAAAAGGGGTTGATTTATGGGTCGGATTAGATGTTTATGAGTCTGAATATCTTTCCATTTTCATATAAATGTTATGTGGTAAGGCCAAAATGACATGGCAATTCCATGTGGCATTTAAAGAAATAAAAAATGAGTTGGATATGTCCAACATGGCAACTAACGTCCATAAGGGCATATTTGGACCAAAAGTTGGATGGCGAGGGCATCAGTGAACCAAACTTTAAACAGAGGGTATATCTAGACCTTTTTGAATAGTTTAAGGGCATATTTGACTCTTTTCCCAAATAAAAATGGTCTAAATATAAAAAAAAATAAAAACAACAAACTTATATATAGGAAAACAATAAAGTTTCCAATTAAAAATAATAAAATAAATTAATAGGGAAAATATGTAATTTATCACTTTAATAAATGTAACTAATACTCATATAACATATTCCACCTTCTACCCTGCATAAATTTATACATAGATTCCCACATAAGTTATATTGGTATTAATTATGGACTACAAAAATGCAACCAAACATGGTATACGTTATGTTGACTTTTCTACCTAAACAAAAATTTCCATCAAACATAGTATAACTTAACATACATGAATAAAATGTAAGTTATACCGTAATCAAACGATCCCTTAGTGTATTAATTAAACATAATAAGGGTATTTGAGTCATTTATATAACAACATGACTATATATGAGCCATTTTTATAATAAGAGGTATATCAACTTGAAATGACAAAGTTGATAGATATATCAGACCATTTTTCCTAATAATCATAATGATTGAGTTTGTAATTTAATATTTAGTAGAATTTTTAGACGTGAACCCGTAATTTGACTTTAGACCAATCCACATTATTATCACAAAATCAAAAGAAAAAAAATGATAAGGTAAGTACATAAAGGTTGATATTATTTCCCATGTGAATAATGATATTGTATGTTGAAGTGTGTGACCGTCTTTTCACATAAATGTTGATATTATTTCGTATGCGAATAATGATGTTCACTTTAACAAAAATAACTTTTAGTGGCAATAAATAGACACATTAATAAAAAGTGCTAACGTCTTTACCGGCATTAGTTAAGTGTCATTAGATTCAACATTGCTAAAGGTTTTAGGAACATATACTAAGAATGCTAATTGTCACTAAAAATACATATTTAGCGGCAATTGAGCTATTGCCGTTAAAGATCATTTGTTATGTAGTGATTGTATGTTGAAGTGTGTTGTCATCTCATCTACCTATGCAAATAATGATATTGTATGTTGAACTGTGTGATCATCTTATTTAAAAGTTTAATTTGTTAGAGAGAGCATACTTTTATTAGTTAATGATATTGGCAAAATTATACAATAAGCTTTAGTATTTGTACTTATCTCTAGCAAGATGAGTAATGTTGTTAAGATTATCAATTGTCATGTCATATGCAATTATAGCTTCTTTTTTTCAAATAGGTATAGGAAGGGAAAATATGATAGAAGATTATAATGTAGGGAATCGAACGCTCACCAGCAAAGTGAAAGTTCATGCATCCAGCCAACCAACTGAGCTACTAAAATTCCCAACGGCTATAGCTTTTATAATATCGATGGAGTAATAACAAACTAAGAACACTATCTTCTTCTTCTTCTTCTTCTTTAGTTCACACCACTTCCATTGAATGTTTTTTCGTCTTTTGTAAGACAGGTCAAACTCTTGTATGCAAATACAATTTGATAATTCCCAGAAATTAAAGCAAAGTGATCATCATTGTGACAAACATCAAAGTCCTCCTTCTTTTCCTCTACTTATGTTTACCCCCCAACCAAATATAGTTAGGAACTTAACAATACTCCAACTAAGTAATGTCAAAGTTGTATCCATACTAGGTTTTCTGAAAAGAGTATTAATTGATGATCAAAAATTCAAGTTAGAAGTTCAGTAGAGTTTGAAAAAGATGGAGAGAAGTAACGCGTTTCGATCTTCTTCTTCTTTTTTTTTTCCAAATAAAGCCAGACTCCAACTTTGTCCTAATATATCAAGTACTATAACTTTCACATTTACTCCCCTGCATTTCAAGTCAATAGATGAGCAGGTGAACTCTTTTATTGCACTCTCCAACCCCCACAATCATCTACACAACAAACAATTATATATAATAACACGTTACGTTAGTGTCATGAATCATCATAGTCCATCTCGATCTAATTATATTCAGATTGAAACCGACAATGTTAGATAATCAGTATTTTTAGGACGACTAAATGTGAACAGTAATGTAGTATTTGTAGACATAAACTACTTACCTTTTCTATAAATTCGATCATTAAGCTTTTCCTCCTCTTCAACCGACACCATCTTCTCTTTCTGTGATGGCTTTAATTCTTCTCACCTAACCCTCATTTCATCATTCATTTCTGTGTCTACCTGAACTTCTGCAACTTTTTTTTTTCTTTTTTTAAATTTGTCCCCCTCTTAACAACATGGTACAATCAAAGAAGTTCAAAGGTGTCAGACAACGCCAGTGGGGCTCTTGGGTTTCTGAAATTCGCCATCCGCTGCTGTAAGTTTTTCTCACTCAGTCATAAAACTACAACTACACTGATTGATTTACCAACATTCTTTCTTTGCTGGTGCGATAAAAATGTCTTTTAACTTTGCTCTCTAGTATGTACATGATGAGTCGATTTTTTTGTTGTTTTTGTTTATAGGAAGAAAAGGATATGGTTAGGAACATTTGAGACAGCAGAGGAAGCAGCAAGAGCGTATGATGAAGCAGCTATCTTGATGAGTGGACAAGTTGCGAAAACAAACTTCCCAATTGTGAAGGAGAGTACTGATAACAACAATGACACTACTAAATTCCCTTTAACTTCTTCATCAACACTGTCTAGTAGCATGCTGAATGCAAAGCTGAGGAAATGTTGCAAGGATCCAGCACCTTCCATGACCTGTCTGAGGCTCGATAACGACAACTGTCATATTGGAGTGTGGCAAAAAACATCAGGGAAACATTCAAGTTCTAATTGGATAACAAAAATCGAACTTGGAAAGAAGGAGGAGAAGCCTCATGATCATCAGGATATGAATGCTGAACTTGGCATTACTAAACCATTGGACGAAGAAAACAGAATTGCTATGCAAATGGTGGAAGAACTACTCAATTGGAATTCTCCGTTTTCTGATGAACCGATTACTGATCATCTCTCTCCGTCTTTTTCCAACTCTATATGA

>SlERF8-14

AGGGTAAATTTGAAGAATAAGACGATTAAAGAGTAATGAAAATAATATTAACAGAGGAACTAAACAATACTCGACTAATTTTTATGTTTTATCCTAATTTAAGATGATATAATTAAAAAGAAACTGTGATTAAAACCAATCACTAATTGACACGTTTCGATATTTCAGATAAGTATTCAGTTTGATGACGTGTCAATCATCTAGATCGAGACAGGTGTCAAGTAGGTCCCACCTATCTCCATTTGCTGAGTTGTTTAAAATATGACCAAGTCTATCAAATACTGCCAAAAAAAAACTTTTTGTAATTGTCATGCTGTGTTAATTAATTGGACCCCACACATCATAATCATTTTATTTTTCTTTTAAAAAATAATATTTGAAATTAATAATATTAGAGACGTGTGGATATGTTAAATTCTTTTTTAGTTGACAATACGACTTTTATGACTGGCGACAACTCAAAGTTTAGGAAGAAGTTAGTATACAACTTTAAAGATTGAACTTAAAGTGAAAAAGAAATTTATATTGACAATATAACTAAAATTCGTCAAATTTAATTCACGAATTTATTTTTACAAGCAACTTTGTAGAGCTAATAAAGAAAGGAGGAAGGGAACGATTAAAATTTAAATCATACTTATTTTGTGAAAGGTTTTTGTTTGTATCACTGAACAACACTTCTTCTGTATTTTTTTTACCTGATACAATTTTTAAAAAAGTAATAAAAAAAATTAAAATATTTGATTTTAAATTAAAAATATGTGGCTTATATTGAAATATAATTTAATATGACATGGGAAAGTCTAATTTTGTCATTTTAAACAGATTATAAAAAAATTGAAATAAAAAGAGTATTATAATTATGTGATTGACCTTTTTTTCTAATTTGGTCATATAAATTCAATTTTTAATTTTTCTTTCTCATTTTTTTTGACCAAGCAAACTAATTGGGAGTCAATTTCAGTCATTATTATAGCCATAACCTCTAATTGGATAAATAATTACTGCTTTCAATTAATGGAATTAGAGAAAATGAAAGGCAAATAATACCAAGTCAAATTTTATGATGAAAAAGGTTGGACTATTTAGACAATTTGATAGAAATAGTTGAGTCGTGTATTTTATAAACTTCTAAAAATAATTTTTTCACAAATAAAAAAATTGTGAGACTTAATCCATCAAAATACTACATATATAAGTTTATTACATATATACACTAATGTCATGTTAAAATAAAAGATCTATTTATGAGTTTAGTTCGAGAAATTTCAATAAGTCATTAATAAGTTCTGTCATTAAATATAAACAAAAATAATCTCGTTTTAGCATGGTCAAAAGTTCGAGATTACTCCTTATGGGATCTTAATCAGATGTGTTTGGATTCGAATCTTGCTTTTTATCTACATTTAAAAGTCTGGACTCCAACCCATCACATTACACCAGTTATGCACCCATAACTTCCTAGCAACTTTATTACTAAGTAAATAAACATTTTAATGATTATCTTATTTTCTAATATATAGTATCTTTTTTCTATAATAAACCTTACGCTACGTGAATTTGAAATGCTAAACACAAGGGGAAACAATATCTTCTCATCTACATAATTATTCATTACATAAATAATATCTTCCACTATAATATCCTCATAATAAGTAAGATATTTTAACCAAACAATCTCAAAAGTGAACAAAAGAAAATCAAAGAATGTGAAATAGAGGGCCTAAAGAGTACGGTTTTGTATTGACTTTTTAGGTTGATAAGTTAGGTGTATATCACATGCAAAAGCTATTTTGGGCCGTTGATACATGGAATGGGCCCATTTACTACACGGGTCTTGTCTTCTAAAGGCCCCATCATCAATTTTCACCTTTATATATTTAATGTTATTTAAGGTCCCACAAATTTTAATTTCAAACTTTTTCCATGATTAACACCATTCACGTCTAGCAAAATTTGTTAGCGTGATGGATCGAGAGTTCTGTTGAGACGAATGAGATTTTTCTACTCGTAATTAGAAGTTTTGTGTATGAATTTATGTAATAGGGAAATTTTTGACAAAACACATTTAGTAATGAACAAGAACAAGATTCTTCTACTGTAACTAGAGATTTTAGATTCGAGTTGAGAATGTTTTTTTTAGTAAAAAATAGTTTTTTTATATGATTATGAGCAAATTTAAATTAGTCAGAGCGTGGAAAAAATCTTTAAAACTTTAAAAGCAATTATGGAATTAGTTTTTTTAACAAATACACTACACTTAAATCTCAAATCAAAACATAGATGAAAGAAAAATTAGGTAGTCTATTTTTAGCTCCATTATAGAGCAAATTTTTAGCATTAGGTATTGTTGATATTTGTTTGGGTAATATTATACGTGTAATTAGTAATGAATGGTTTCAAATTGAAGCCCTATATTTGAAATAAATCTTTTTCAAATTCTGCGTAAACGTAAAATATGAGTATTACTTTTTTTTATTTCACTTCAAAGATGAAGCATAACAATTAAATAAACTAATAGTAATAAAATTGGATGAATGGTACTTTTTTTGACTTTTGTTGCATAATATAATGATGCATTTCATTTCATATACCATGCTCCAAGTTTGGTGGAATTTTGAGATTTAAAAAGTGTTTTAGCATATGACTTGTCTTTTCCTTTTATTAGTAAGAGGCAACTAATTTCAAAGTTGCTTTTTGATATAGTGATTTTTCCAAATTTAGTTAAACATGCATGACAAGTTAGAGCTGGAAAAATTATAATATTAGAACAAAAAGAGATACTTATAACTTCAACCATCAAAATCAATTAGTTATAATTAGTTTTAAATTCCAATCTCTAAATCATTTTTATTAATATTTTAAAATTTTAATCTTAACAATCAGGTGTTTCTACTGATATATTCGAATTTTACTAGTCTACTGTTCTTTTTCTTATCATACACGCTCGAAATCTAAACTAATTCTCAAATTCCTATGCGTAAAATTAGGACGGAAATAAGGTGCAAACTTCTAATCATATTAAACCCTATCCCATTACCTACCTACCTTCCATGCCTTGCACTATTCACTAGCTTATGCCAACCTAAAATATATTTTTTCAATAAATAAGTAAATTTTACAACAAAAGATTAAAGGTTTTAAATGAAACAAAAAAAAAGGAAAAAAAGCAAACTTCTGCCAAAAAAATAGGAAGATAGAAATAAATGAATAAGTGAAGACGTGGCACAAAAAATTAAATGGAAGAAAGCTGTAAGTTGAAAGTTGAAACTAGAAGCAAAAGGCTATCCTTCTCTCTCTTCTTGTTTTAATCGTAATTCCATTTTCAGTGTTCATATTAGAATTCGATTAATTTATATTTTTGTTATAAAAAACTTATATAAGAGGAAAGTACTCGTTAAAAAGATTTTTTTTATATATTCAGAACTCGTATTTGAAATCTTTAATTAAAAATAAAGAAATTTTTATGACTATATCAATCTCACGATAATCCTGATTGGTAAAGAGTCTTTATCTCTCAACCGTATTGAATGCTATATAATAAATTCATAAAGTTGCTTTTTGATTTCTCGGCAATTAAGTTAAGTCGATAGAGAGTGACCACTCTTTTTGTGTTTTTAGGTCCACACGGTAATTGCCTTTGCCCAAGTCAAAATTACCCTTTCCACAGTGTGAACCCATCCCAAAACCAACCAACCAACCACCCCAACAAGTAAAATTATACCCCCTTTATATTGTTAAAACACTTTTTTTGAGGTTTAAACACTTTTTTTGAGGTTTAGAAAGTTGAAATTCGTAATCTAGTAAAACGTTAATTCGCTTTTTATTTAAATTTAATTGTATTTTTCCTTCTTTTAATAACTTAAAAGAATGCAATAGCGAAAATATATGTACAAGGAAGATTATCGATTGTATATCTATTGATCAAGTGTTAAAAAGAATGGAGCTAAAGGTTAAAAGAAGTTTATAAGGTTAAATTTTGTTTACGTTTATATAGTAGATGATGACTTTCTTGGTAAAAATCTTGTCGCAATTATTGGAAGGAAGCATATTCAACTAAAACAAAAAAAATCACCTTAACCTAGTATTGGTTGATGAAGGCATGTAGTTCGAACACTAAATTGAACGTATGATCAAGACTACATTATATAAATCAAAATTTAAAAAAAAAACGTTTTTCTTGAAAGTTGCTCTACCAGAAACAACCTTCTACCAAAAAAAAAAATAGGGGTGAAGATTATCGTACACCTCATGCTCTCTCAACCACACTTGTGAGAGATACGTTGTTGTTTGATTAATATTGCACTAAAACCTTATGTTATGTTTCCACTTTATTACTATCTCAGAGTCTTCCCAACACTCCCACCGGGGTGGAGCTATCATCAACAAAAAATTATTGTACATTGAATTCTCTGAACTTGTTCGTGTGTTTACTTCTTAATACTATGAACGTCTTCATGAAAATCTCTTCATCCCACCACACCCCCAAACACACACAAAAATAGAAACAGTTCAAGTAAATTGATCAGTTCACTTTAAACAAAACATGGAACCACTTTACTGACACTTACTGCAAGGGTAATATAGAAATAGATTGCCTAGAGGACTTGGTAGATAAGAGTAAAAAGTTGAATCATAAATGCCAACTTTTAAAATAGAAGAAAAAAAAAGTTATGGGACCTTAACATTAAGCACAAAAGATCCTTTTCTCAACAGTTATAAATACAAGCAAAAGAGGATAACAAAAACACAAGTGACATCAAACTCAAAAAAAAAAAACAAATCTTTCTCCAATTGTCCCTTCTTTTTTTTTTAGCCCTCTAAAGTTTTCGCAATTAGAAACAAAATTAAGTTTTTCAAACTGTCTATAACTCACTAAAGGCCTCGGGTTTCTTCATTGATCCTCTTTTTTTCTCTGGATCAGTAAAAAAAAGGGGCAATTCCTCTGTTTTACTCTGTTTTCCTGAACTCTGTTCTCTCATTTTATGAAGTTATTATAAATGGACAGAATTCAAGAAAACAACTCTATGTCTAGTATATTTGATGAGGGTGCTCAAATTCGATCTTCATTATCTCAACTAATTCTAACAAGTAGTACAAACACATTCGATACAATTTTTTCTCATTGTCAAGAAAATAACCAAATGAGTAATAGTCCTGTTTTTGAGCCTTTAGGTACTTCAGTCTACCTCAGACAAAGAGATTTGCTACAAAAATTCTGTCAAGAAAACATAGCAAATATCTCAATACCAACAACTTCGAAAACAATCCCTTTTCGAAACTCTTTGTATACACAAAGTTATAAGCTTCCAGAGAAGAAAAAACTATACAGGGGAGTCAGACAGAGGCATTGGGGAAAATGGGTTGCTGAGATAAGACTTCCACAGAATAGAATGAGAGTTTGGTTAGGTACTTATGAAACTGCTGAAGCTGCTGCTTATGCTTATGATAGAGCAGCTTATAAGCTTCGTGGCGAATATGCAAGATTGAATTTTCCTAATGTTCGCGATCCGAGTAAGTTGGGATTTGGAGATGGAGAGAAAATGAATGCTGTGAAGAATGCAGTGGATGCTAAGATTCAAGCTATTTGTCAGAGGGTGAAAAGGGAGAAAGCTAAAAAGGCTGCAAAGAAAAAGAGTGAGAATGAGAGTAAAGTTAAAGAAACTATGGATTCATCATCTTCTACTACTACTACTACTATGATTTCACCGAGTGTTTCTGAAGATGGATTATGGAGAAGTGAGGATTCAACTTGTTCTGTTTTTGGTGATTGCTTGAAGGATCCATTGATGGAGTCTGAGTTTGATAGTTGTTCACTTGCAAGAATGCCTTCTTTTGATCCTGAATTGATATGGGAAGTTCTTGCTAATTAG

>SlERF4-7

CGTAATACATTGAAAGATTCTTGAGCTAATTTTGTAAAATTAATTATACAATTATGAGACAAAAGAAACTTCTAGTGCTTTGATCTTCTGGTAAAATTGCATCATATGATGTGTGGAAAACATTATATTTTGGTGAAGGAAAATATAATGAATTTATCGAGTTTTAAATTTTACGTCACTAAGCATCTCAGTCTTTGGAATTTCGAAATTGAAAAGGATAAGACATACGTAAGACTTGCTTTCTGATGCAGACGTGACCAAAAAAATTTTAATGCAGAAAGATTAAAATGCAATTAAAAATTGACTTTCTTAAAAATATGAACAAGAAATGAGCATACATACAAGAAGAAAAAAAATATATAAGCTAATTAATGCGAAGGTAAATATAGTAGTATTTATAGTTTAATGAGAGGAAATTTAAGTTATTTACTCACTATTTACAATCATACCAAAGAAAAAGATGTTAAAAATTTAAATCTGTATCAATAAAAAAATAATTACATAGATTATATGTTACTATATTTTATAAGAATAATAATAATAATATCGTATTTAATATTTTAAAGATATTTAATATTTAAGGTACTAAAGTGATGATATCATTTTTTTTAGCATGTAACAAAAAAATAAATATTACATTTAATTTAACATTAAAAATCAATTCATCAGATATTAAATTATTCAATATTATATAAGGAGGTAACAACTCGATCTCTTAATCAATAATGGCAATGTAATAATCCATTTTTTCCCTTTTCTTATACAAAAGGTGGTTATCAACTTATTACTATGTTAAGATATTTTCCCACCAAGATTAGTCCATCAACTTTAACAGTTCGACGGCACAAATAAGGTGCTCGAGAAGCTTTTTTTTTATATATATATATATCTAAATCGATAAAATCTTTTATTAATATATTAACAATTAAAATAGCTAATTAATTGAGCTAACGAGAACATGGATGAGAAAAGTTTATTATCGGATAAAGTTACTTTAGACATATATTTTCAAAATTATAAAAAATTTGCTAGTTATATCATCCTGTAATTGTGTGCATAGTATGGTAAATCCTCACATATTCATTAACCAGTACTTACTAAAATTCTTTTAAATTAATAACTTTTTATCGTATGCTCTGCATAACATATTTAAAATTATAAAAAAATTTAAAAATATTTTAACACATATATATATTTAGAATTCATAAGATAATTTTTTTTTAATTTTTTTAATTTCATGCCGGATATAGTTAAGAACCATAAAGTGAAATTGAGAAGAGTAATAAATTACATTATGAATAAATGATTTATATAAGATAGACATTTAATTTTTTTATATATATATATATATATATATATATATATATATATAGCATTGAATAGTCTAAAGGAGCTATTTATTCAATCATTTGGTGTAAGAAAAGTCTTTATCATTTTTATTGCCACACCACTCTATCATAAATCGTAGAAAATCCAAAAAGTAAAAATGAAAAATAGATGCCTTTAAATTCTAATCCTCAAAATATAGATTTGCTATTGAATTTTATGGTGATATTTAAGTCACTATACATACCAAATAGAGGATAACCGTGAACAAAAATTATTCTCAACGTCTACATCATTAAAATATACCATCTTTTAATCATATATATCTCCCTTTAATTTATTTTGTCTTTTTCCTACAGATATAGCTTCGATTTGTTACTCAAAAGTGTGTGTTGAAAGTACAGTTCCAAAATACAAATTAAAGAATCAAGCATTTGAATAACTACCAATTATTTGATTTGACTGGAGATAATTATCTTTTGTTTTATTGTTAGCAACAATAACAATATTTAATTTGTAATTTTTGCAAACCTAATCTTTAATCATTTTTTTTTAGCAATATGAAAGAATAATTGATGAATCATTTTTTCAAGATCGAACTTGGACCCAAAGCTGAAGTCAAAGCTAATTATCAATTATGTAGGGGAACTATAAATATTGAGTTCAATTCATATTTTTTTAAAAAATAAAATCAATAAATGTTATATTTGGAACTCAATTTTCAAGTGCGTAAAGATTCGTACTTGAACACACTTATCGGGTTTTGGATTGTTTGGCTTGAAAATAATTTATAGTTTAACGTAGAAATTAATTATGTTGGGTTAACTTTTGCTGATTGTTTAACTTATCAGTTATTATATAAAAAATGATGTATTAATACAAACTTTTAGGTAAAAGTTATTTGTTTATAAAAAATCTCCATTATATCTAATATAGAAAAAGAGAGCTTAGGGATATTCTGTTATTTTCATTATTTTATTTTGAGATAATTAGTTTTGATACTATTATTTCTCAGACAAGTACGAGTGCTACTAATTACTAGTACTCATATAATTAATTTATTTTAAAATTAGTAACCAAATAAATAAGACATTACCGCTGTTTGGACATAATTCCAAATAATGATTTAAAATTAAGTTGAGATCTTTCGAATTTATAAAATTGTACTTATTTCTTATAAAATGAAACCTCATAATTTGTAAGAATTATCAAAACTTACATGACTATTACTCAATCCTACCAAATAGTATATCGTAGATCATAAATAAGGTATCAAATATCTTAAAATGTAAAATTAATAATCAAATGTTTTTATATATATATATTTTTTAAAATAACATTTGTGATTTTACGAAGATTCACTAGTTGAAAATTTAACAATTGTAGTAACTAGAATTTTTATATATAAATATTTTATGTAATATGAACAATCTCTTTATATCAAGTAGGAACTCTTTTTTTTTTTAGTAAAACTTAAAATTATGAATTATTACTTTTGTAAAGTATAAACTCGTACAACAAATTTTACATATAAAAGGAATAAAAACCACAATTCATATTTTTTCACGATACTTTTGAAAATTTCATAGATTTAAAATCTTAATTTGAATTTGAAATTTTATGCAAATTTCATAAAATAAACACTGATTTAAACTCATGCTACTAAATTCCATCATCAAACATATATATATATATATTATATGCATAAAGGACTATTCAAACTATCTAATATTAATTAAAAAATTCTTTTATTTTTTATTTTTCATTTAATGTTCAAAGTCTGTATTAGAACTTTAAATAAATTTAAATTACTTTATTGATCCTACAATTTAGAAATAATGTCTCTAACAAGATTTATCCTAAACACAAGATTCAAACTAAAATTTGTAATTAAAAATAAAATAATTCCACCGTTATACCATTGCCATATTAATAGTATAGAAGAAATTCAATGAACCTAATTAATATTGGGTAAGAAGCACCATATAAATAATGAAAAAGGAAAACAAGTAAAGTCGAAGATGAACTCTTACCATAAAGTATAAACCGACAATTCCATAGATGCATATAATGTCACCATTATTATCAGTCAAACTGTGTGAACATAAATAACTTCATGTTTTTCTTTTTGAGCAAGCATCAACACATTTTTCTAATTATTTATGGTGTACTTCAAAAATAAATTTAAAATAGAATAAATCAGCACTCCTTTCTTTTCAATGATGAAAAAAATAAATAATAAATTTGGTCCCTCATATATTCACCATATATATATTATGGTCCTTTTAAAATGTGTTCTATTTACAAATTTAAAGAAAAAAAAATACATTCTTGTTAAAAAACAGTAAAATTTTGATGTCATTTATTGATAGTTAAACCATGAAATAATTAATTTAAAATATACTAAATAAGATATTACAAAGACTCAAATAAGAGTTTATCAATAAGCTGATGGAGCAAAGTGCTATAAAATACATTTAATTTTTATGCACTGATTTTTGTATTAGTCTGCTTTTAAAAAGAGATCATATTTATAGAATTTTATTATCTTTATCGTAGCATATTACTTGTTTAATTTTTTTAAAAAAATTAATATTTAAAATTTAAAATCTTAAATTCAAAAGTCTCTCATGCTACATAAAACTTTGTATCAATGGAGGACATGTATTTATGCATGTATTTAGTTAATATTCTATTCATTTTAACTTATCACCATTATACTTTATCTATTTTTTAAAAAGTTTTTTTAGTACTTTAGTTTCAATTTTTCTTACGATGACATATTTAAAAAGTTTAAGAACATCTAATACATTTTATAAATTTATGAATTCACATAATTCAAAAAGTTTCTTCTTTTTTTTTTTGTAAGTTTTTCTTTTTTTAAAGTTTAGTTAAATCACAATTAGATAAATATTTTCTTAACAAAGGAAGTACTTCCTCTATTTCATCTTATTTGAAATACATGTAGATCTGATTCATCTTTTAAATTTTTTACTTAAATGTATATTTTACTAAATTATCAATAATTTTTTTGCAATTTTATATTTAACTAACATTATTTTATATAGTAGTTATCTACTATTAAAATGAACAAGTACTCCCTCTGTCTGTAATTAAAAATAATTATTTTATATTAATTGTTATGAATAAATTATTAAATCTTTTATTTAAAAAATATTTTAAAGAGTGAATTAATAAATGACTTTTTTTATATATATATATATTTTCTTAAACATGGTGCAAAATGTAATGACTAATATAAAATGAAGGGAAGAAACAATAAATTGACAAGTGATTAACATTAAAATTTTGTGCAAATCTATAAGAAGAAAGTTACTCCAAAAATTAGATACTCTTTATCTACTGTAGCATTGACGGCTGTTCAACGATAAATTTTCCTCCTCCTTATAAATCCCCCCTGTCTCTCTATATTGTCAGCTGGAAAGTGAAATTTTTGTCTGATCATAACATCTTCTCTTTAACATAAATAGCTGATGAATTGAGTACTCAAATAGATTTTCAATTAATTAACCCATCAATTCATATAAAGATTTTCAAAGTCGGTGATTTTGGATTCATTATTATAAAGTGTTTTTCATCTTCTTTATCAATGAAAAGGGTGTTCATACTTGTTGTTTTCTTGAAAATTTGAGGTGTTTTTTATATAGTAGTATATGTGCATATTAAAGGTGGCGAATCAAGGAGATTCCGGCAAGTATGACAGAATTCCGTCGACAGCAGGTGATTCTGAAACTACAACGAATGAAGGAATTCCTCAACCGTATGAACAGTCGCAATCTTTTGAAGAAATGTTACAGCAACAAATACAGCAAGAAACAGAGTATTTGATGTCGGAATCGGCTAATCCGATGTATACAGGGTATAGTCAGTCGAGGGATATGTCGGCAATGGTGACGGCGTTAACACACGTGGTTTCGGGTCGGAGAGAGGCAGAGTGGGGTTACAGGCCGGATATTAGCGGTGTTACGACTTCGTTTGGTGGTGGTGGTTCAGGGAGTATTTATTCGGCAAATTCACCGTCTTCTTCGAGTTCAGGATCATGGGCTGGACAGAAAAGAAGACGTGATCAAGAAGAAAGTGTTACTGGAGAACAAGCTCAAAGGGGTTATGGAGGTATCGGAGAATTTAAAAATGGAGAATCATCTTCCTCTGTTAAGCTCGGTTCGTATCGATACTTTCCACTTATTAAACTCTCTTCCGTTCTTTTTAGTTGGAAATATTAGGAAATCACCGTAACTCACGCTATTATTTCACCCGATATAAGTCTATCGGACGAGCTAAATATTTTGGGTTCATTTCAAGATTCTATCCTTTTATTTTATTTTTCTTCTCAATTTTCAAATTGTTCTCTGTTTTGGACCGGATATATGCTCTGTTTTCTTTATCTTGTATTGATTGATTTTGACTCTTTAAAACTTGATTTTGATTCCATATCATCTTCTAATGGTTGGTTCCAGAAGAAGATACAAGCCTGGCAACACCACAAACGAGCAGCATATCGACAACTACTGCGTCGCAGACACCACCGCAAGCATCGGAAGTAACAGGTGAAGAAACAGGGGAGAGGAAGAGGAGATACAGAGGAGTAAGACAGAGACCATGGGGTAAATGGGCAGCAGAGATAAGAGATCCACATAAAGCAGCAAGAGTTTGGTTAGGCACATTCGATACAGCAGAAGCAGCCGCTAAAGCTTATGATGACGCCGCTCTTAGATTCAGAGGAAACAGAGCTAAACTCAATTTCCCTGAAAATGTTCGGTTATTACCACAACAACAACAACAACCCACAACAAGATTAGCCATATCAACCTCATCATCAACCGCAGCTCCGCGATTCCAATTAATGTCTGCAGCATCAACGCGATCACCATCACCATCACCATTTTTTTTTCAATCATATAATCAACCTCCGCGTCAATCTGATCAGCAGCATCAGAATCAGCAACAACAGTTATTTCAGAGCTCAGATATGGCTAGAGATTATTGGGAATACTCGCAATTACTTCAAAATCCAATAGATTTCCATGGAGGACAACAATCATCATCTTTGTTAGAACAAATGTTACTAGCTTCATCATTGGGAGTGTTACATTCACATACATTTCCTTCTTCGTCATCTTCTTCATTAGCAACTTCTGCAGCTTCTTCCACTACTTCCCCTGCATATCCCCTGTTTTACTCTGCTCAACAATCACGCTTCTTTCAGCCACAAACTCATCAAAATCAAAGCAATAGCAGTAGCAACAGCTCAAATTTTCCTCCACCTTTTTGGACTAGTTCCGGCCACTATCCACCTTCTTCTAGCTAA

>SlERF12-12

AAAGCGATCAAGATTAATTAATTATTTATTTTTTAATCTTATTTTCTTTTATTTATGGTTTCTTAATAGACATAGCGATAAATAAATAGTATTGAGGTAAGTTCAACTACTTTGACAATTTATTGTAAATTAATTTTTTTTAGATATTATTAATGTTTGGCAAAGAGCCGTGCTTTGACTGTACGGAATACTATATAAAATTAAATTAGATCGTTGAAACTTTTTTCAAAAAAATTAAATTGAATCGATTTATAATTTACTAGTTTTTCTAATGTCGACAGGAAATAAATTATATTGATATAAGAATAACTCCAAAAGATTATTCAATCTTAAAATTTGATATTTTTTTTAAAAGAAATAATAATGGTGAGATATCTTATATTTGGTGACGCAATTCTAACTCATTTGTCAATCTATTGATGTCAACATTTGTTTCATAATTAGAAAATGACTTGATTTTATCCAAACGTCCAAATTAAAGTATAGAGAAAACACATATCTAAAATTTTTTAGGTGTACATGAAGACATTATTGAGGAGGCTTGTTCTAATCGTTAAAGAATTTCTATTATCAATCGATGGATTGAAAATTCGAATTTACTGTTACTGAAGGATAAGAGAAAACATTACTGATTCTCCTCGAGTTTTTTTAAATCTAAGATGATAGGAAAAAATGTTGAATTTCAAAGTTTAGAAACAAAAGGAGTTCTAACTATTGAACTCAGGGTCATGTTGTCGCTATTATTATATATATATATATATATATATATATATATATATATATATATAGTTTTTTAAAAAATAAAATTTATAGCATTATTTATCGTTTCATAACAAATTTTAAATCAGATACAATATTTTGCTATTAGATATATTAAAATTGAGAGAGAGACGAGTTTCTGTTTATGAATTTCAGATACACATGAATTATACAAAATTATACACACAAGAGATACATGTATTTGTATGTACCTGATATGATTTACATGTATTTAGAATACATAGAAGAGTGACGTCTATGTATCACAGATACATGCGAACCCGTAGAGGAAGGCGAGTGAGATTTGCCTGTGAATTCCAAGTACATGCGAATTTACTTGGATATAGTGCATCTAGAATAAATTATACCTAATTTTTATATCATGTATTTCGAGATACATCTATGTAGTATTGCAAACTAACATATATCTAATCAATTAACTCCTAAACTAGTGAAATTTCGATAAGTTACCCAATTATATTTTGCTTTTACACACTCTTTTAAAACAAATTCGAAGTAGTATTCAATTAATTTACGGGATAATGCACAAGTACCCCATAAATCTATGTTCGAAATTTTCAAATACACACTTACACTATACTAAAGTCCAATTACGCCCTGAATTTATTTATAAAAAACTTTCTACCCCTTTCGACCTACGTAACACCATCTTGTGGGTCCAACATGTGTTGATTTTTTTTAAAATTAGTGTCACGTAGGCCAAAAGGGGTAGAAAATTATTTATAAAATAAATATAAGAGAATAATAGAACCTTAGTATAATATAAGTATGTCTCTGAGATTTCAGGCATAGATTGGTGAGGTACTTATGCATTTTCCCATTAATTTACTTATAACTCTTTTTGACGAAATTCATATTGTTAATTTTCAAAAATGAACAAAAAAAGAGGAGATTTATTCAACAGTTGAACATTGATTAACAATTAAATATGTTGTTAGATTCAAGTCACTTTAATGGAATAAAATCCTATGAATGGATAAACGTGAAGACATATTCTTCTCTAAATTAACTTTGAAAATTATAAGAGAGCTTACTTTAAGTATATAATAATGAATACTTCTAGTATTTTTATTTGAAGTCAAACCTTTGAATATTCTTATGGTTTTTCAATTATATAAACATTATAATAATTATTATAACATTATTATCATTATAAATGTTGCTAATATTCTTTTAAGTATATTCATTTAATTAAAGGAGAGGAGAAATTAAAAAAAATATGAAGCTATATATAAAGTTTGAATCTCGAATTTTAATAATTAAAAAAAGTGCAAATTAACTTTCTTTTATCAATTTTAGGAGCAAATATTTAACGCGTAGAGGATTTACTTTTAACAACGAGTAATTATAATTGTCAAGAAAATTAAATAACCTTTTGTCATAATGAGGAAATTTTTTTGGTATGTCAAATAAGATTTCTCATTGTTATTGAGCTAAATTTGATTTAATAATAAGTTATGTTGATCTTGCTAGAAATTTTAACGAATTGCAAATATCTTTTTTATTATTTGTGATTATAATTCTGATGATCCTTGTTCAAATTTTAACGATCATCATAATTTTTAATATATAAAAACTCACGAACACAATTTTATTTGAAAATTGATCAAAATTAGCTCTAATTAACTCCAAAATGGATACACTATTCCCTCCGTCCACTATTGTTTGTCATGGTTTTAATTTATAGAGTCAAACTTTAAAAACTATGACTAATATTTTTAGATGTATTTTCTCATCATATTGATATGCAAAAAATTGCAATTTATAGTACTTTTTATATAGTTTTTGAATATCTAAATTTTTTATTTAAAATATCGAATTAATATAATTTAATATAATTTTAAAAGTTAATCAAATTCACTTTCAAAAAGCTCAAAATGACAAATAAAAATGTACGAAGAAAGTACCTTCTAATATTAATTTCAATTGATATATTCAACTAAAACATTTAAAAATAAATAGATCTATTTTATATTTTCTAACATGTCTTTTTTTTTTTCAAGATGAAAAAATCAATAATGGTGTTCTTAATTTATTTAATCTAACTAGACCAATTCAAATAGTTAAGCATATATGGAATATTACCAACAAAATTTCAATTATAAAAAAAGAAATGGAAAAAAAAAGGAAAAAAAGAAGAGTAGGAGATGAAGAAGTGATTTTCTTGGTAGAATCAAAATCTGTTTGAAATTTTTATGACTTTTTGTAAGGTCAAAAATTAATTATGGACTAACCCATATTCAATATTGAAGATTCAACTTGCAGCCCAAATGGAAGTCAAAGCCCACTGTTTTAATTACCCGCTGTTCTGTCATGTTTGACTTGATACGTAATTTAAAAATTAATAAATAAAATAATAGCTTTTATTAGATTACTTTTAACTAATTATCACAATTCAATTCATTTACTCGTGCAACACTTGCTCTAACATTTAGATAAGAGAATCCTCAACTTCAAACTAAATCTTAATATGTGAAAGTGAACAAAATATAAACTTAACACTCAACTTATATATACTCAAATGCTTTTTAACTTTATTTACAACCAAAGTTATTTGCAAGAGAAACATTGTAATTTGTGATAAACATTGCACTTATCTGATCACTCAAGATCAATTGGTGATAAACATAACATAAATAAGAAACTTGGTTCACATAAATCAACATAAAAATTCAATTTTCTCATGAAATCCACACATAAATTCTTAATGTATCAATGATAATGAACCAACTTGTGTTAGTGTTTCATATCAGCCGTGGTACACGAGGCAATCGTGGTAAAATAAAGTACATCATATGATTACTAATGTCTGATTCCATCAATCCGTTATAACAGACATACATGCGCTAATATCGGTCAGTTCGGTTCAGTTTTGAAGTTTATCAGGTTGTGTTATTGGTTATCGGTTTGTGGAGATGCTTAACCGTTATAGAATCATTAAGATATTGATTGACTTATCAGTTATTGGTTTATTGATTTTTCATCGTTATCAGTTCGGTTATCGGTTTAACCGTTAAGATTTGACACAAAAAAAAAACTTGAAAATCACTTAGAAATAAAGTGACAAACCAAATAAACCATGCACTTGAATTCACATGTCACATCTTGGTCAAAATCAAACACTTTTACATTGTTGAATAAGCAAGTGTTTGAGACAACCAGAAATGAAAGTAGGAAATCAAACTCTAAGTCGAGGACTTTCTATACAAAATGGTATAAATATAGTTATTTAATTTAGTATCGGGTTATTAGTTAACCCGTTAAGAAAAAAATTCAAACCGTTAAGAACCGATAACCCGATAACAAAAAACCATTATTAAAATCACTAAACCAATAACTCAATACTATAAACCAATAACTTTTTTATCGGTTCGACTTATCAATTTCGATTCGATTTTGAATGGACCTAGGCGCTACAAACTATGCCTTCATACTAGGAGGCTTCCCGCTATAATAAGGTGATATATACACACAGTATTAATAAAAATTCATATTTCTAGACTTACCTAATTTCTTAAAGGACTTAAAATTTAAAATAATAACTAAAAACAGAGAGAAATTTTTTTAAAAACATTCACCACTAGCTTGAAATAGATGGATCTAAAAAAAAAAAATTCACCGCTAATAAATTTGTCAAACAAATTAAATGAACCAAGTTGGGTGTTTGAAAACCAAAAATTAAAATTGAACCAAGTTGAAAATGAAAGTGACAAAAAAAAAAAGAAAAAGAAATCAAGTTGAAGACAATCTCTTTTTTTTTTTTCTCTATAAAATAAACCGACACTCTTTTTAAATAGTTCGAAAGGGGCGTAAGCATATAGAGATGCATGTAACTAATCAACAGTGTGAACATATTAGCTTAAGTCGGCATCTCTAATCTTCTTTTATTTTTTTTATTATATATAAAAAAAATTGTTTTGATTTCTACTTTATGCATGTGCTCAAAGAGAAAAAACAAATCATATGAGCTAAATAAAAATTATTTTTTTTCTTTTTGTTTGTCAGTTTCAAAATCGGTGAACAGGGTATTTATAATTAACAAAAAATTAACATCTGGTTTTTTTTTTGTATTTAATTTTGTGTATATGTGCTTTTTAAAGGTGGCGAATTCAAGAAAATCAAGTGAATTTGTTAGATTTACAGATACAGATGATACACAAACCACCGCCGTGACTGCGATCGGCGGCGGTGTTGAAGGCGGTGGTCAGTTTGATTATTCGATGTATTCAGGGTATTGTGATTCTCAGGCGAGAGATATGTCGGAGATGGTGACGGAGTTTACACGTGTGGTATCGGGTCAGGATTATCGACCCGATACCAGATGTTATTCGGTTAATTCACCGTCTCCGGCTTATTCTTCGTCCAGCTCGGGTTCTAGAGCTGGACTGAAGAGAAGCCGTGATCAACAAGAATTTGGAACTGGGTTGTCATCTTCTTCCTCTGTTAAAATTGGTGAGTGAACTATTACTTTTTTTTTCGAGTTTCATATCTCAATTATCAGTGTTTAATTATTACTTAGTGTTTGTTTGAATTGACTTATTTCATTTCAGATATGTTAGTTTTGAAGTGTTAGGTAAAATAAAAATAACAAAAATAAATTAAAAGTTAATCCTAATAAATAGGTGGGGGAGTTTTAGTTTTTAGCTTTTTTAAAATGAACTAAAACACATTTTTTTTTGAAATTTTTAATTTTACTTTCATCACTTTCAGGAGGAAAAGGAAAAAAATAATTGATAGTTGAGATTAGTTTTATACCTAAATAGTTATGATTCAACTGTAAAATGAAATTTCAAAATGTTTTTTTCCTGTTATTTCTTATTTTTTTTAGTTTAGATCATCCTTTTTTGAAAATCAAAACTAAAAGAATGGACAATTTTTTTTAAAAAAAATTGCTTGCTTAGTTTTATTCCATGGTACTATTTGACATTATTTCATCATTACCATAACTAGGAAGGTAATTAAGTAAATTTAGATATATAGAAATCCCAAAAATAGTTCAATTTTTTGTTCTTCTATCCTCAATGTAAGTACTAAGACTTATTATGTTTGTATTAGAAGAAGCTACAAGTATGGTTGCACCAATTCCCGCTTTCACAACCACAATCACAACCACGACCACAACAGGTGAGGGTTCGAGCGAAGAAACAGGAGGAGATAGGAGGAGGAAATACAGAGGTGTACGACAACGACCATGGGGTAAATGGGCAGCGGAAATAAGAGATCCACATAAAGCCGCCAGAGTTTGGTTAGGAACATTCGATACAGCAGAAGCTGCAGCAAGAGCATATGATGAAGCTGCATTGAGATTTCGAGGAAACAGAGCAAAACTCAACTTCCCTGAGAACGCCAGATTGTCATCGTTACCACAAACACAAAATACTGTAACGTCAACAATCTCCAATCCATCCCCTCTAATAGCTCAACCAACGTCGTTCCTCAATCCTATCCAGAGTTCAGATACAACAAGAGACTACTGGGAATACTCACAATTGTTGCAAAATCCAGGAGATTTTACGGATCAACAACCATCAAACTTATTGGAACAAATGTTCGTTGCCTCATCGATGGCAATGTTGCATTCAAACACATTGCCATTAATATCTTCGTCTTCATCGTTAGCTACATCAGCAACTTCTTCAACGTCATATCCCCTGTTATTTTCGAGTTATTACACACCACAAACTAATCAAATTCAAGGAACCAACACAAGTAGCACCAGCACCACTAGCAGCTCAAGTTTTTCTACAACATTTTGGAGTAGCTCTAGCCAATATCCTCCATCTTCTAGTTAAATTCTCAAAATTTTCTTGCTTTTTTTGGGGTAATATTTTCACTAATTAGGGAGAAAATTAGGGGTTATATCTATTGTTTAGTAGTTGTCATTATTTTTGAATAGGTAAGAAATTTA

>SlERF3-17

AACTTCTGTTCCTGAAATTGTATTATTGCCATAGTTTTCTGCTAGAAGTGCAGACAACGGGTTTACTACCGAGGCTGTTATTGGTTTGCTGACTGAACAGTAAAGCGAAGACACATTTCACCTTGAGTCTTTTCTAGTAATTACAGTGTGTTGTACTTGGTTATGGATATGCGCTCCACTATTTAGTTGAGACCTGGAATAGATTGTTCACAGTCCAATTGCCAATACCTACTTGGGTGCAATAACATTTTTGCCACTATTCGATATGAATTTCCTGTTGGTGGGTAGCTACAGTGTTGTCTATCTGCCTTTTCATACTCACCATTTACTTTATCTTTCAAGAGGTTATGAATTTTTTGTTGGATTAGGTTACAAGCACTGAACCTATTTATATTTCATCTCTGTGTAACTTGCTTAAAACTATTCTTTCCAACTATCTTTGGATTCAAATAAGTTCTGGTGTCGCCATTTTAGTTTATGTCATTGTAATTGTGAGGTTATTTTTTCTATTAATTAAATGAGTGTTAAACATATTTCTTTGTAGATTGTCACAAAATCTGACAGATCCCTGACCATGGACCTTGTATGTGGCGAACAATTTTCTGGTCAAGCACATGCACGGAAGTTTGGCAAGCTTACAATTTTTGCTGAAGAAAGTATCAGCTCAAAAACTACAGTCGAGTTGAAATTAAGATGTTCAGAATTGGAATCGAAAGATTTGTTTTCAAAAAGTGTACAAACTCATCAGAATAGTATTATCTATTGTCATTTTGAATCATATGATGCTGAATTGCTGATATACATAATTCTTCTGCGTTTGACAGGATCCATTTCTGGTGGTATCCAAAACTACTGAAAGTGGAATTGTCGTTCCAATTTGCAAAACAGAAGTGATGAAAAATGATCATTCACCCACCTGGAAACCGGTGTTCTTGAAAATTCAACAAATTGGAAGTAAGGTACAATAGAATGCTCTTTTGAGTGATCAATTTTCTATTTTCATAAGATTGTGTTATGTTTCCTGGGATAATAGAACCCTGAAAAAGAGGGTAAAAATCATTTAAAGATTTTTTTTCTTGGTCAGGAACTATATGATATTCCAAGTAAACAAGGTATCTTATGAGCTATAAGGGCAATGTGCTGACCTTTTTCTAGGCCTTTCGGCAAGTTCAGAAAACATTGCTAGTATTTTTCCAAATGAATTCTGCAGAAGAATAAATTTATACTTCAAATGATGAAACTTCCAAACTCACTTTTGGCAGATCTTTTGTTTTCTTACAGAAAATATTTCACCCATCTATTCTCTAAAACTTGAAATCAGCTGTGTTTTTTTGACTTTATGATTGCATTAGTAATAGAATCTTGTGTTGATGGGATTCTGCAGATGTATGGGATCTTACCTTCTGTGCTATAGAAACGAACTCTACTCATCGAAGTATCTAATTGTTTTGATTTTGAGGGGCTATACTTTATGATTTTATTTATTTTAAAAGATGTGATTGATAATATATTTCCCCTGAGGAAATTATTTACTCTATATGGTATGACGCATTGCACAATATTTGTTCTTTGGTTTTGCATTTAGAAACATTTGAATTGATGCAGGAGAACCCACTAATTATCGAGTGTTATGACTTCAATAGCAATGGCAAGCATAGTTTAATTGGGTAAGCGTTTTAGTCTCTTCTTCATTTCTGACCTCTACAGACTGGAAGCTGACCATATTTCTACTGATTGTCAGAAAGATTCAGAAGTCACTATTAGATTTAGAAAAGTTGCATTCTGCGGGTACTGGTGCAAATCTTTTTATACCAACTGCTATGGGACCAAAACAAGAAAACAAGGTAAAGAGCTCGTTAATCAGACAACACAACTGCCAGCTAAGCTTATTTAATCATGACAATCACTGGATTTCTATCCAGGTTCTAAAGAGCCAACTTTTCGTGGAAAGCTATTCTGAGAAAATACAGTATACATTCCTTGATTACTTGGCGTTTGGCTATGAACTCAACTTCATGGTGGCCATTGACTTTACTGGTATGTGGTTGTGCATTGATATGTACATATATATAAACAGATTCTCGCAAGTCTTTGTTTCCATAGCTAAGCATGGAATTCATATTTGTCGTCTTCATCAATGGAACAATTTATTTAGACAAATTTGAACCTCTTCCTTATTAGGATGCACAGGGGGGCTGGATTGAGACACCAAGGGTTATTGCCTCCATTGGCAACTCTTACTTATTATTATTACCATAAATAATAACGAGAGTTATTGATGTGAACTCATCCTGATCTACCATAACCCCCCACCCCACCCCTCTTAGCAAAAGACCCTGGAAAATCATTACATTTTCTCAATAATTTTCTGTATCGTATGTATACACAGACATTATTTTTCAGAAAAAATAAGTGACCTATGCTCATCATTTTTTTGATGATGAACCTTTTGTTACACAGTTACCCTTCTTAAAAAAATAGATTTGTAGGTTAATGTTTGTAATCTTGCTTAACTTGTATCTGAGCCTGTATTCTTTTTTGCCTGAATTGTTGTTTGCCAGCTTCCAATGGAAATCCACGGCTCCCTGATTCATTACATTATATTGACCCTTCAGGACGACCAAATGCTTATCAGAGAGTAAGAAAATATTCTCAGTTGTAATGATATTTCTCTTCATATGAAGTAAGATACTTACCAGAATCCCAATTTCAGGCAATCCTGGATGTTGGGGAGGTATTGCAGTTTTATGATTCAGACAAACGTTTTCCTGCTTGGGGGTTTGGAGCACGGCCAATTGATGGCCCAGTTTCTCATTGCTTCAACTTAAACGGTAGCAGTACTTACTGTGAGGTACCTAAAGAACCTTATGTACTTCCATATTTTATGGACTTGCTTTAGCTTTCTGTATCTTATTCATTTTATAAATTTCAGCCCTTTTTTTAACTCTAGGTGGAAGGAATCCAGGGTATCATGATGGCATACATTAGTGCCCTTCATAATGTTTCACTTGCAGGTCCAACTTTATTTGGACCAGTTATTACTTCTGCTGCACATATAGCAAGTCAGTCTTTGACGAACAATGAGCGGAAGTATTTCGTTTTACTAATTATCACGGTACATCCTCCATTTTCCTGATGAGTATCTTTTTTTTAACCTTTCAAGTGAAGTGCTAACCATCATCTGTTATCACCTTGAGTTATACCAGTCAGAGTTATAGTTTATAGATCCCTTCACAGTCATATGTATAATACCCCTATGCTTTCCAATGAATAAATTTCAGTGGATTGCATAAAATGTAGAGAAACTTGAAACTTCAACTTTTGTTGGAAATCAAACTCTATCTAAATTTGTGTTTGGGTTAATTCATTTGGGACCATTTCTTTTTATAACTGAATGTCCTATGTCTTCCTTCCTGTTCTGATTATATTATAGAAAAATTTGTAGGATGGAGTAATAACAGATCTCCAAGAAACGAAGGATGCTCTTGTTCAAGCAGCGGATTTGCCATTGTCAATTCTAATTGTTGGGGTTGGAGGTGCAGACTTTAAGGAAATGGAGGTATGCAAGTGCAACAACTTGGTTCTTTTTATGTCATCCATTTTACATTAACTGTATTGGATCATTTCCGGTGTGGATTACTTTTCGCAGATACTGGATGCTGATAAAGGAGAGAGACTAGAATCTACAACTGGACGTGTTGCTGTTCGTGATATAGTACAATTTGTTCCCCTCCGTGATGTACAAAGTAGGTCATATGATAAATTTCAAGCATTCTTTAAGAGTTTTCAGAAAATTATGCATCTGTTATGATGTGATGCCTTTTCTTCGATTCGCAGATGGAGATGTCTCAGTCGTTCAATCACTTTTAGCAGAATTACCTTCTCAGTTGTTGGAATACATGCGGTCCAGAAAGATACAGCCAACTTTGTGATCGATCATACAAGACTTGTACATAAATTTTGCTTCAGAAGATTAAGCTGATATCTTTCATCAGCATAGTCTTGTAATTGATTTTGTTGATAAAAAAAAAAACTCAACTCCGTTGCCCTTTATACTTGCAAATTCTAATCTCCTTTCTGTTACAGTTACCTTCCAGTTGTCCGTTGTAACTGTCATGTTTAAGGCTGTAAGCAGTAACTATAATTTTTTCCTATTTTAAATCCCTGAAGAAAGCAATTTGTGAAATAATCTTACATTAGGTATTGTATTTCAAACTTTGATTTCCTATAACCTTCTCTTGGTTAAACTATTGTTTTTTTTCTTCTTATATTTTCTTCTACCATTAACTAATACACTACTAAATCAAATTGAAAATTTTGGATTTTTCTCTCTGATGCCTTTTTTAATTATAAAAAATGGCATACTTCTAAAATTTCAGAAAACTCAACATGAATTTTTATTCTTTTGCCCTTCAGAAATTTTTTTATTAGTGTCTTTGAATATTCATATAGATTTTAACCTAAGAGTCAAAAATCATAAGTTAAATCTTTTATTTTCTTACCTTATGAACAAATACTTAAAAAAAAATATTTTACTCAAACCATAAGATGTACTCCATATAAAACCTAGTTTGAACACTTACAAAATTAGTACCCAACTCCAAGTCATACACACAAGTTTTTCAGGGAAAAAAAAACCTTTTTTGCTCATTAAAGTCAGCTTATTTAGTCAAAATGTTCCATTTAAATCGAAATGTAATGAGTGTATATATATATATATATATATTATGATGTCCCATATGAAATAAAACAAAAGAAGAAAGCAGACAAAGTTGAATATTGTGGTCTTGGACCGTATATAGTAATAGTATATTATTTTTCCACATAACTAGTATGTATTCACATTAAGTACATAAAAAAGCTAGAGTAGTAAACCCTAATATCTTCTTCACTCCTCCTTCCTAGCTCCACTCCTTTTTCTCACCTCATTTACTAATTTTTAGTAACATAGCTTCTAGTTAAGCTCTTGTCTGAATGATTTCTTTTTTTGGATCAAAGAAATGAAAAGATCAAGCTCTAATAATGATCAGAGGGATGAAAAAGACACTAGTAACATATTCCCAATATACTCATCAGCTCGATCTCAACATGACATGTCTGCTATGGTTTCTGCTCTCTCTCAAGTCATTGGTAACTCTTCGTCTTCAGCTAGTGGTGATTCCTCCTCAGTGCATGTTAATCCACTAACCCTAATTCAGCAACATCAATCTCAATCGTCTACTCAAGATCAAGGTATATATTCTCTTATTTCTATCTGAATACTATCTCGTCTAATAATTTAAGTTATTTTTATTGGTTGGCCCCTCACACCTAGAAGCTTTGATGTGAGCGTGGGTATCATGTGTTATTTAATTCAGAAAGAAGGCGATATAGAGGAGTTCGTCAAAGGCCATGGGGTAAATGGGCAGCGGAAATTCGGGACCCGAAAAAGGCAGCTAGGGTTTGGTTAGGTACTTTTGAAACAGCGGAAGGTGCAGCACTTGCTTATGATGAAGCTGCTCTAAGATTCAAAGGAAACAAAGCTAAACTCAATTTCCCTGAAAGAGTTCAAGGACAGTTTTTCCAATGCTATGATCAACCTGCCACGTCATCAAACAACACATCTGAACAAAATTACCCTAATGTTCATCACTATGCTGATTTATTACTTCGTACTGACAATAATATCGATCTAAATTTCGATGTTTCACCAAATACTTTTTATCACTCTTTTGATATTTCACAATCATCAATGGAAGTTCCAGTTTATCATGAGGAGCAGCAACAAGTTATAACTACGCACGAAGAAGAAGAAGAAGATTTTGTGAAATATCGTGGATCACATTTTGGAAATTCTACTTCAAGTGGTGGAACTAAATAG

>SlERF1-6

CAATTTTATTTACCTTCTTTTTTTAATTGTTTAAAAAAAGTAATAGTTTTCAATTTTTTTCATAGTAGAAGTTTGTCTTTTTTTCCTAATTTTGCAGTAATTTTTTTTACTTGAGTTAACATCTTTTATTAATTTCTCTTGGTGAAACTTTTAACTTTTTTGATGATAAAATTAGGGAACTTAGTTAATTTTTTTAATGAGAAAATAGGAAAAATTAACCAATGGAAATTTAAATTAACTTTGTTTGATTTATCTTCTTTTTTTTTGTATAAAGAAATTTAACCCGAGAATTTTCATTATAAAAGTACAGTTTTTTTTTTCTTGTTTTATATAATTTAGAAAAAAGTTTTATGAAGAGAACTCTTTCATTAAGTTCACACTTAAACCCAACTCAAGATAAAATAACTTTATACACTTGCATAGCGCATGCTTTAGCTAAGTTATGTAAATATTTTAAATTGAAAATAATTTTTTTCTTTTAAAAATATCTTGAGTAACAGGTTTTAGACAAGAGGGACACTAAAAAAAACACTATTTTAGCGATAATAAATAGACACATTTAATAAAGAATCTTGAAGTCTTTATCGACATCAATTAACTATCATTAGATCCAATATTGCTAACGCTTTTAGGGACATATACAAAAAGTGTTAATTACTGTTAAAAATATATATTTAGCGGTAATTGCTAATTAATTACCGCTAAAAATCATTTTTATGTAGCGAGAATCGCTCCGCAGGCTTCTAACTTTAAAATTGTAAATTTAAGTCATCAAGAAAACAAAGGTGGGAGCTCAGGAAAGTTACCGACTCTCTCTCTCTTCTCTAAAATCATCTCTTCACTAAAATCATCATAAAATTATCTTTTTAGTAGAAAAAATCATAAAACTTTCTCTCTACTATACCAATATAATGTTGAACATTTTGGCTAATTAATATCACATTATGGGAATAATTAGTTCGTATTGAAACATAAATCAAATATTTATTAATTAGTTAATTAGTAATGACCTTAAATCTTATGATGAAGCACGCATGCATGTAAAATAATCAAAGTTGAGTCCAAGTGTAATTAACAATAAACAACAAGTGTTGAGTATTCCCTAAAACCCTCGACCGTTTCAGTTTAGTTTCAAATACAACAATTTTTCATTTGGAGTTTATTGTTAAAATTTGGCTTTTTTTTCTTGTATAAAACAAATGATAAAATTGTGAACGAGACCTACTTTTTATATAGAATCCATATATATATAAGAGATACGTTACGTTACGAGTTGTTACTGCAACTGAACTTGTACCATCAATATGGACAAACTTGTGACTTTAGTTGGCCCCCACGCCAAATGCATATAGCTAGTGGTTGTTAATGGAGACAAGGTAAAGTAATATGAGGGGGAAAAAAACTCTTTTTTTTTTTATATATAAGTAAGATAAAAGAGAAGAACCAAAATAGATGCCTTTGGTGAAATTTGATAGATGTTTTTTATTAAACGAAAGAGAGGCAAAAATGTCATTTAAGTGGGTTGAAATTGAACAAAAATGTTCTAAATTGAGATAAGGTAAAGTAATAGTTCATTCGTCTTATTTTGAGTGACACTTTTCATTTATCGAAAGACACTTTTCATTTATCGAGAGTTAACCAATTTAACTTTGATTGAATGTTTACGTAGAAGTTAAATTAAAAAATTTTAGCTATTTAAGAAAAAATTCTTTAACTTCCTGTTGTAAGATACTATATTCAAAAATTGTAATCTCAAATTTTATAGTTTTAAAATACAAGAGTTGTAAGTTTCAATCAACTAATGAATTTTAGTGCGCTATAACAAAGAATGACATTGATATAACACTTTTTTGGGGGGGATGATAACAAATTCAAATGTACCAATTTCATGGTAAACATCAACATTGCCATAACAGTTTTTTTAAAATAAACGAGATTGTTATAAAAAGGTTTTAGTTAATAATATAAACTTGACTATGTTGTGTTATTTATTTTGTAATTATAAGATACTTAGAATAAAATTTTGAAGAATTTGGTAGAATATAAAAAAGTTTAAAGAATATTTTCAAAATTAGAAAATTAAATTTTAAGGAAATTATTCACTTCAAATATCTCAGGTTAATGAAATATATTCTCTTAGGATACAACGATCTTATTAACTGATATATCGATATCTAAAACTTCGGTGTCAACTAAGCACTTAACCATAGTAAAATATATTTAAATACTAGATTTAGTACTAATATATAGTACAAGTCCAAAAAAATAGTTCAACTAAAATGAGATGAAAATCCTCGATTTATAAAAACAAAGGATAGTGTGAAAATATTCTCATTGTATTTTATCAGAAAGATTACAAACCTTTGAAAAATCACAATCTTTCAAAATGATCACAATTCTTCATTGAAATTGTAACTTTTCATAAAAATTGTAACTCTTTAATTTTTATTCACGTCTTTTAAAATTCGACATGTTAATTTTATTAACCTTCAAACTTTTATCAACTTCAAATTAAAAGTGAGTGAAAAACATTAAACTTTTAACCTAATAATCGCAAATCAAGTCCTGCTATACCCAAATCTACCTACACTCTACTATAAATAAGGATGTACAGTAGCCTTTTCTTTTGAGTTCTAGAGTTTGTTCAGTGTTTGGATTTTTGAAATCATTTCAATTGCTACCTCGATGATCTCACCCTCCAATAGGTGGCCCTTTCTTTACCGTCAGATCATATGTTACTATACATTTTTGTCTTTACCGATTAGTTAATCGTTCATTCCTCTTATAGAATTTTATAACTTTTCCTTTGGTTATTCATCAACTTTTAATTTGATCCTGAAATAATATATATATATATATATATATATATATATATATACACAACACATTTTAGAATTGGTTTAACTTATAAATTAAAAGTCATAAATGAGAATTTTTAAGTTATAATTTAGCTTACTTTTTTTTATTTTACCTTAAAAATTAAATGGTCATAAATACTTTTTTATAAATCCATCGAACATACTAAAATATTTAAAAACACTTAAAATAAACTAGTTCAAATAGACTTTGCTTTCAATTTTTATTTTGACAGTCCAGTCTAGCAACCTTATTCCATTTTCATTTTAATCTTTTAATTTTATTTTTCATGTACTATCGTCTAAGCACGAAAAATACTATTAAATATAAATTGTTGAGTAAATGACGTTAATGTATTCTATTTTTTTGTATGTTATATACTCGGTCTTCTGAATTTAAGTCAAATATTCATTTCTTTTAAATCTTAACTTAACTCACACTATGGATAAAAATGACTTATCTTCCATATAGGTAAAAAGAAAAACAAAATTCGATACATTATTAATAAAAATACTAAAATGCGACTAGTTCCGTTGGTTTATCAATTTTATTTATAAAAACAAAATTCATGTACTTCCATCGATTGTTTTTTCCATAAGAATATGCAGAAACTATGGGGAAAATTTTTTAAAACTTTTTTCTTGAATTAGCAAATAAATTGATTGAGAAATAAACAACTGATTGATGTGCATCGATTTTTTATTTAAATATTAACAAACTAATGTTACCTTTTAAAGTGTACACTGTAATTAAAGAGTATAAATAAAATTATAATAATTTATGTTTAAATAGTACATAAAAAATGGAATAAGGTTAACATATAGTGCAAATATTTAAAATGGGGATAAATTAAAGTTGTTGGATGTGTATTCGGTCTTTACCTCTAATGTTTTATTTTGTCATATGTTTTGAAATTAGAACTTGAGAAATAATTTTAAAAAGATTATATTTGAGAAAAAAATTAAATATCATAAATGAAAGTCAAAATTTAAAAACTGATCAAGATAATTTCTTAAAACTTGAAAAATATTTAAAATAAATTTTTAAAATTTTATTCAAAATCTATAATCAAATTAGTATCTTTTTAAGAAAGAATTCAAAATAGATAGCCAAAACTAGCTTCAATTCATGAACTTGATTGGTTACTACCACTACTAGCATATACATTGTCTTATGAGCTATAGAGTTCAAAATTGGTGCATACGTAAAATCGGTATGAGCGCCTATTGAGATGCTTTATTATTACATCAAGTTTTAGAACGAGTGAATCACTTAGATAATATATACTTATTTTAAAAAATTGATATAAGTTGATATTTTTTTTTTATTTTTATCTTTTAAATTATTTTCTGAGTATTCTTAAACTAATTCAAAGTTAACTTTTAAATCTGAATAAGTAATACTTATATTCTTAAAATATACCTAATTCAAAATAAACTCATAAATATTTATCGGTTTTTATAAATTGAATGATAAATTCACTATATTAATTATTCTTTGAAAATAATATATTACATCAAAGATGAATAAATAAATAGTAACGATATCAAACTTTAGAAAAAACTTTTTTATTTTTTAACTTTTTAATAGTGTATTTTAAAAGTATATATATGTTCATGTGGATATTAAAAATATTGCATAACAATAAATAACAATGTGTCTATGTAAGCACACATATATACTTATAAAATACTCTTTTAAATAATGCAGGATAAAAGAACCTCTACAAAATTTAACAACGTAATAACAATTTCAACTAAAAAAAAATACTACAAACATTTTTCTCTAAATAGTAACGGAGAAGAGCATATGAATAAGTAAAAGTAAATAGTAACTGAGGAAGTATAAAACTACATATTCGTAACGGAATCACTAGTTCCAATGTGCTTTTACGGATTAACAATTGTCAAAATCGCGTGAGTTTTTATGAAAAGTTACCATAAATGAACATTTCGAGAAGTTTCAGGTTGTATATGCTAGATTAATTATCTAAGAGGTGCAATATTAAAAGTAATTAATCACTACAGGACTAAACTGCAAATATCCTTTTCTATAAATTAGCACTCACCCCAAGCACAAATATATCGTCTCTTTCAATTACTTTCACTCAGAAAACAAAAAGAAAAAAAAAAGAGTTAGAAAAGATGTGTGGAGGTGCCATAATCTCCGATTATGATCCCGCCGGAAGCTTCTACCGGAAACTTTCTGCTCGTGACCTCTGGGCTGAGCTGGACCCTATCTCCGACTACTGGTCCTCTTCTTCCTCATCCTCAACCGTCGGAAAACCTGATTCCGCTCTGTCGCCGGTGACTCACTCCGTCGATAAGCCTAATAAATCAGATTCCGGCAAAAAAGGTAACATAGAGATATATCTGTGCTTGTAATGTATGTATCTAATGATTTATTTTTAGCTTCTTTCTGTGTTTGATAAAATTATTTAATATCTAATTTTAAAGTGAATTTGTACATAACTATCTTAAAATTTATGTATAAAAATGCACCAAGTAATGTAATTTTCTTTTTCTTTTTACGATTGAAAATATTTTTTGAAATCCGTTTGAATTTTTTTTTTCAATAGAACTCACTGTCAGGTACCAAAAAGTCTTTTTTTTTACCAGAGATTTTATTTGGATTTTAATTTTTACTCATAAGAGCTAGAAATAAATATTTGTTCAATTTATTTTTACTCGCCTTAAAATCAAAATTTTACTTTTTGTTCTTTGTAACATATTTTGGTCATTTTGGCCATCCTTGCTTAATTTTTTTAGTTACTTATTTTCAAAATTATTAGTTATTTGAAAAATATAATTTTTTTCAAAACGTTTAAAATCAGTGTGAACAGTTAATAATGTCAGGAGGTGATAAAAAAAATTGTGATAAAGAGGGAATAAATGCAATTATAGGCCGGGACAGTCTGCGAATTAAAGTGACCTATGATTTTTATAGAACATACCATCAAATTTTGTGGCTTTTTATTATTTGTAGCTATCTACACGTACGTATATACTCATTTTTAATCTTTTTTATTGCATCAGCCATCAGGATATATATTCAATTACTCTCAATTCTTTTTTGGGAAAAATAAAATAAATTTATTGCAGCGACTAGTATCTGTCAATTGCGTTTATCTATTTGCACATGTGTTCTCCATAAACTAATTTACTTACTTATATGCTGTATTTTGTTTTTGTCGTTTTCGAATCAATTATCATGCTCAAACTTATCATAATTGAAAAAAAAAATGAAAATCAATTATTATGAAGTAATATTTGATGGAAGCAAAATTTATCTGTCTTCTGTGCCAAAAGAAAGCACCCTCTTTCCTTAGCTAATTGTTTCTTCATTAATATTATGTTTGAAAACTATTCTTTGGTCATTTTTATTTAATCCATTTTAGTTTCAATTAACCAAATCGATCCTTGATGAAGGAAAATCATTTTTTAAACAGAAAATTAGACAACAAAATTATTTTATCAAGAATTAAGTGACTAGTCTTTTTTTTTTTTTACGTGTACAATTAATTGTGTGGGCCACTTAATTCATTATATTTAATATGACAGACTTTGCTTTAATTATTTTTCAACTAAAGTATTAAATCACCACTAAAAGTTGTTGGTATCGTAGAAATAATGTGAGGAACAAAAGCCATCAATTATTTATAAATCTATAATTAATAAATTTTGTATTTGCTATACAGGTAATAAGACTGTGAAGGTTGAGAAGGAGAAGAGTAGTGGACCAAGGCCAAGGAAGAACAAGTACAGAGGAATAAGACAGAGGCCATGGGGAAAATGGGCTGCTGAGATTCGCGATCCACAGAAGGGTGTACGCGTTTGGCTTGGTACATTCAACACAGCAGAAGATGCTGCTAGAGCCTATGATGAGGCTGCTAAGCGCATTCGTGGTGATAAGGCTAAACTCAACTTTCCAGCCCCATCACCACCAGCTAAGCGACAGTGCACTAGCACTGTCGCTGCTGCTGATACACCACCAGCACTACTCCTTGAGAGTTCTGACAACTCTCCTTTGATGAACTTTGGATATGATGTCCAGTATCAGAGCCAAACTCCCTACTACCCCATGGAAATGCCCATAGTTAGTGAAGATTATGAACTGAAGGAACAGATTTCCAATTTGGAATCGTTCCTGGAATTGGAGCCATCTGATCAATTTTCAGGGATCGTCGATTCTGATCCTCTTAATGTTTTTCTGATGGAGGACTTTGCTTCAACTCATCATCAGTTCTACTGA

>SlERF6-6

AAATACATCATCAAATTTTGAGAAAAAAATTATTTATGTCATCAATTAATAGTTTGATTCATCCAGTTTTCATCCGATAAAAAACTCATTTATATCATTATTTGTAAACTGAAATGATATAAACAAGTTAAACTTTAACAAAAAAAACAGAAATAATTATTTTTCTAAAAATTTTATAATATATTTAAATATTTTCTTTTAAATTAATCGAGACGATAAATATCTATAGGTTTGGTTATACAAAAATGTTTCAAAGTTTGTTAAAAGCAACAAAGCTGCAAATAGGGAAGCAGCGTAGCGTGTTGTAACTAGTGGCGCAGAAATAGACATAAAAGCAAACAACCCTTGTGAAAGGGAAGGTCAGAGTCGTAAAATCAACGTAATTATTTAGTACGGCCTTGTTTGGATTCTTTCCCTAAATAGTTGCCATCTTACTCATCATTAGTCCCCACCCAACCCCACATCTTATCCCTCCATAGGTGCTTTTTCTTCGGTGACTCAAACGAACAATTTTTTTAGTGTCGTTTCGTATAAAAGGATATTAAATTATAGTTTGGTTTTTTATTGTTGTCTAGTTTAGTTGAAAAGTTTGAGATAACTTATTTTGAAATTAAATTATGGTAATATTCTATAAGTTTTATTTTAAGCTAAATAAAAAGGAAGTTTTACTTTTAAATACATTCAATGCCATAAGGGGTGTTTGTAGTTTTGTTTGGATTGATTACTAATTAAAATCAAAATCAAATCAATCTAGTCGATTTCTCAAATTTCTAAAATCAAATTAAACAAAAAAAATAATCATCGATTTGGTTCAGTTATTCAGATTTATTCGATTTAAAAGTAATTTTTTAGGACGTACATATCTCGATGGACACAAACACCTAGTACATGAACAATGTAAAAAAAAAATTATTATGAATTCAATTAAGTAATTAAGAAGCAATAAAGTTATCATTGCACAAACAAAAGTGATTTTGATTAAGAGAAAATATGACCACTTTACGTAAAGTGTGATAGGTAAAGACTAAAATGAATTTTGATATACTTGGACTTAGATTGTTATAGTTGGCTTAGGCTTGAGAAAATGATAAAATTAAAGATTTAAAGCTAAATAAAATTTAACAAAATATTTGTATTTTATTTGTGAGTAATATATTAATCATTATAAAAGTTATATATCCAATTAACCAGCTAAGTTTGGTTAATTTTGCAGCAGTTTTTAGTAAAACTAATCAAATAGTATTAGTTTTCAAAATTTAAAATCAAACTTAACTAAACCAAATTAAATTTAATCAATTTGGTTCGAATTGCAGACCGAATTAATTTTTAATCATAACCATGAACAACCCTGAATACCATCATGAGAGTGCATATACAAAGATATTTAAGCTAAATAAAATAAAATTTTTATTTTTAAGAAGGAATAAATAAAGCTTGCAAATAAAATTCAAAACATCAAAGGATATTCTTTTAAAATCAAATAACTTGTTCTTAAAATTCATGCGATATATATTATTTTGCATATAATAAACCAAAACTCAATAAGATATAATTCCAGCACAAATTATCTTCTACTTATAAAATTATAATTAATCATATCAAAATTTGTATTCAAATCAAACAATTCCTCAAGATTGAAACTGAGGATATTTACTATCCGAGTATCTTCCTTTTATCTATCTCTAATATTTATAATTTTTATAGTTAAAAACTAAATTTATATGTAGTACATATAAGATTATTTAAAAACAAAAATTATAGTTTATGAAATATTGTAATACAATATATTAATATCCCCCACAAATATAAAATAGGGATTACCAGGCAGGTTATGATCAACTACACGTATATTAAAAAAAGTACGTTTTAATTTATTTATTTTATTTTTAAATTTTAGATATATTTAAAAGTTAGTTAAAGTACAAATAAAATATTTTTTTCAAAAAAATATGTTTTTAATATTTTATGTATTTTGTGGTTGGAAACTAAATTTATATGTAGTAGATATTAGATTCTTTAAAAATTAAAGATATAGTTTATGAAATATTATAATAGAAAATATTAATACCCCCCACAGGGATACAAGTAGGCCTAACCAGACAGGTCATGCTCAGTATGTGTTACACCAATATTAAAAAAAAATCATTTTAATTTATTCATTTATTTTTAAATTTTAGATATATTAAAAATTACTTAAAAGGTACATTTAAAACATTTTTTTAAAAGAACATATGCTTTATAAAAAAAATTTTGATTGATTCATAAAATTAATCTATTTATGCTACATAATAAGAAAAAATTAGGGACATATATTATTTAATCTAAAAACTACATAAAAAATATTGTAAATCTCAATAAATAACAACTTTTGATTGATTTTTAAAATTTTATTTATGCTATATTATAGAGACAAGAGAAGTATCATATATTATTTAAAAATTATTTTAAAAATATTATAAATTATAATCATTAACAAATTAAAATAATTGAAAAACATATTAAAATCTAATTGATTTTATAAATTCTATCTATATTATATAAAATGAGAATTTTGTATATTATAAAAGTAAAAATAAAAAAATGAACAAATGGGCAAATACCTTATTTCTGGATCTTCAGGGTATAGCGCATTTTCCCGATGCCTTTTGTCCGCTCCAATTTTTCTTCCAAATTTACGGCAAATACTTTTCCCTATGCTAAATATTTAATTATAATAAACTAATATAATTTTTATCAAAAAAATTATTTAATTATATTCCTTTTTGAATTACATAATGTATTATTTTCAGCTATAAAAATGTTTCTCATCTCTAAAATGTAAGAGATGTTTTCACTAAATTATCTTTGATCAGAAAGTACATATCTATTGATTTTGTGAAAAATCAATATTTAAAATTAGCATAATTCAAAAAAATGAATTAAATTTTCTTACAGAATATACGCTGTGGATGTATTCAAATAATAAGAATAATTTATATTTTATATTACAAATAATATAGAAGTTGGTATCTAATTTTTTTTCAATAAAAACGTGTTCTATTACTATTAACTTTGACCTCTATATTTAGCAAGTATTTAGGAGCAGTAAAACCTACTATTTGATAAATCTAGAGTTTTCTTATTTATTATATTTTCCATGAAGTTAGGTGAAAATTGTGATACTACTACGACCTAATCAAGGTCTCCTAGGAGAATGCCAGTTATATTTAATTACTCGTTTATTTTATTTGATTCTTATATCAAAAATATTTTTTAACTTTTATAAATGAATAAGAGTTTATTTGAATTTCTCTCTCTTTATGTTAACGTTATAAAATATTATTGATGAAGTTTATTTAGAATAAAAGTTTTCTTAAAAATAGTTGACTTAAATAGTATAAAATATAGGAATATCATAAAATAGTTTACTAGTAATATGATCACTATTTTGAGAGGAAATAGGCATTGATTTATCATTTATATTGTTATTTACATATTTTTTTTCAGATGTACTTCATTTATTTAATTTATACGGAAATATTTTGTAAGAAAATGTAAAATCAAAAATAAATTTGTAAGGATACAAAACCATTGACCCTTTTGCTTGATAGGGTTTTTACAAATAAAAGTTTGTTTTTTTTTAGAATTTTTAAATTGAACATGTGTCAAAATATTATTTTAAGTATAAAAAGTAATATTTATAAAACTATAAATAATTTCATTTTAATTTTTTTTGCTAGAGGTTAAATACAACGCTCGGATGAAATTAATTTCAAATATAATAATATTAATTTTTAAAACAGGTTAATAAAAAAAATCTTACATAATTTAACTTTATTACTCATTATTATTGTTATTTTAAGATGTTTCTCAAAATAAATTTACTAGCATATTTGAAAAATTGATCACCGCCTGATTTGATGTATCGTGGGATTTTTCAAAGAGAATATAGTTTTAAAATGTGTAAATTGTCCTTTAATTTTCTTGTCAAAATTAATTGTTGGAAACCAAAGATAATAAATCAAATAATGCAGAAGTAGTTAATCTAGCCACTTTTAAAAAATCCCTATTTTTTTTATTACAAAAACAATAATACAACTTAAAAAAAAGATACAATTTTATAATTAAAGATAATTCAATTCGATTTCAATTTTTAGTCTTTCATACGAGTAGCAAACAAGTGATTAAATTGAGTTTAAATGAATCAAGAGGGACTAAAACACTAAATATAAAAATTATCAAAAAAATTTAAAATCTTATCAAATAAGTAAATCATAATTTATAAATAAATTAACAAAAAATCACAAGAACTATTTTATAATTATATATTTGTGATTACATGGCTCATAAAAACAACAACAATAGCAACTAGTTAATTTTTAATATAATATTTCTACATGATACAAATAATTTATTCACATCGATCTTGAATAATCTTTTTTTACAAAACATAAATAACAAGTCTATTTTTATAAAATATAAAATTCTCAAAATGTAAATTTTTAGATCAAATTAGTTTAAAAACAAAAAAAATAATTGGAATTTGAAATCCTATTTTTTTTATAACTTGTTCGGAGAAAGACATTAATTTCGAATTATAAACTTAAAATTAAGATTTCATATTTGATACAATAACACTTACTTAATCTCATGATTTTAAACGGAAGCAAGCCATGTTATAACGTATTAATTTATTTCCTTAATCTCGTGATCTTATACAAATTACGGAGGCAAGTCATGTCATTAACGTATTAATTTCGACGTCGATCCGTATTTTGGTTTAGAAAAAAAAGAAAAGACGTAATTGAGCACCCTCGTTAGGGGTAGAAATGTAACTTACTGCGCCTCGTTCCTGCCGATATCTCGGACCTTTTTTATCTCTATATAAAATGCTTGATTCCTTCTGAACTTTGTAAAACTAAAATACATTTTCTCTTCAAATTGAGCTTTTTCTCCATTAAAATTCTCTCTGCAAATTTATAGTTTTTCTTTTTTCACTTTTTGAGAAGAAATCAAAAGCTATGTGTGGTGGTGCAATTATCTCCGATTTGGTACCTCCTAGCCGGATTTCTCGCCGGTTAACCGCTGATTTTCTATGGGGTACATCCGATCTGAACAAGAAGAAGAAGAACCCTAGTAATTACCACTCAAAGCCCTTGAGGTCTAAGTTTATTGACCTTGAAGATGAATTTGAAGCTGACTTTCAGCACTTCAAGGATAATTCTGATGATGATGATGATGTGAAGGCATTTGGTAATTTCTCCTCTCATCTGTTCTTTTTTTCAGCTTCTAGTAGGTAGGAGTTCCTTTTAGCTTTTGAAATTGTTACCATGATTTTCTGAAAGTGTGTTTTTTATTTGCGTGTCTAAATACGTTTCATGCTCTAAAAATCACCTCGAAATGATTTGATTCAATGATATTAGGAAGGATTTTTGGTCTGACCTAACAAGCTTGAGTTGGTTAATGTGGTATGGTTCATTTGTAGAACTTAATTTAGTTTTAAGTAGTATTTTGGTGTTGAATATACTGATTTAGGTGATTTGGGGTTTATAATCGCGGTTTCGATTGATTTTTCCCCTTTTTAGTATGTGAATATACATTTAAGAGGAGAAAAAAGATAACTTTTGGGTATAGGATGTTTGCGAATTGTAAAATGAAGAGTAAGATGCTGTTGTTCTTCCTAATTGGTCGAGAATGAATATTTTAGGCAACAGCGCATCCTAAGTAATTTATGCAATTGCGGATTGTGCTTGGTGCCCCCAAAAGAGTCAGAGATTTTTGGATCATAGTTTCCTAATTTGAACTCAATTTGACTGTGATATGGTCATTCATATAGCAGATTTTCTAGATGATGAACTCTGATTCTCTGGATGATAAACTTTTTATCTCCGGAGAATGTGCTACGTGATGTTGATAGCTAAAGAGAAATAATTTGGCGTAAATAACAGCAGAGAACATATTTTTGCATGTATTTTGATCTTGATTTCTCTCCTTAGTTATGATCACTGATAGATAACTTTCGAATAAATTGATTTTGGTTACCTTAGTTTTGATCACTGATAGATAACTTTCAAATAAATTGATTTTGGTAATCTTTAGTACTAGTGATATAAAAGAACCTGCTTTTCGTGCGTATTCTATGTTTCTCACTTTCATGGTATCTGTTTGTCAATTTATCTGATTGATTGTTCCAAATTTTGGTCATTTATGACTAACTGAGATGGTTAATTCTTTTTAAATTCTAAAGGCCCCAAATCCGTGAGATCTGGTGATTCAAACTGCGAAGCTGACAGATCCTCCAAGAGAAAGAGGAAGAATCAGTACCGGGGGATCAGACAGCGTCCTTGGGGTAAGTGGGCAGCTGAAATACGTGATCCAAGGAAAGGTATTCGAGTCTGGCTTGGTACTTTCAATTCAGCCGAAGAGGCAGCCAGAGCTTATGATGCTGAGGCGCGAAGGATCAGAGGCAAGAAAGCTAAGGTGAACTTTCCTGATGAAGCTCCAGTGTCTGTTTCAAGACGTGCTATTAAGCAAAATCCCCAAAAGGCACTTCGTGAGGAAACCCTGAACACAGTTCAGCCCAACATGACTTATATTAGTAACTTGGATGGTGGATCTGATGATTCGTTCAGTTTTTTCGAAGAGAAACCAGCAACCAAGCAGTACGGCTTCGAGAATGTGTCTTTTACTGCTGTAGATATGGGACTGGGCTCAGTTTCCCCTTCAGCTGGTACAAATGTTTACTTCAGCTCTGATGAAGCAAGTAACACTTTTGACTGCTCTGATTTCGGTTGGGCTGAACCGTGTGCAAGGACTCCAGAGATCTCATCTGTTCTGTCGGAAGTTCTGGAAACCAATGAGACTCATTTTGATGATGATTCCAGACCAGAGAAAAAACTGAAGTCCTGTTCCAGCACTTCATTGACAGTTGACGGTAACACTGTGAACACGCTATCTGAAGAGCTATCGGCTTTTGAATCCCAGATGAAGTTCTTGCAGATCCCATATCTCGAGGGAAATTGGGATGCATCGGTTGATGCCTTCCTCAATACAAGTGCAATTCAGGATGGTGGAAACGCCATGGACCTTTGGTCCTTCGATGATGTACCTTCTTTAATGGGAGGTGCCTACTAAGCTGCATACACATCTTCCCTTGCTAAGTTTTGTAAATAACGCTTCATTTGAGTGAAGTTTGCGCCTGCGTTTACGTTTATCACCAAACTAAAAGACTATATATGTGTTGTATTAATTTATTCAAAATTTACTCGTTTGATATATGTAAGTATGTATCCTTGTTTTCATAAGTCAATGTCTTATTTTCTTACAGTCACAAAGCATATGCCTTTCGCCTCTGTTAATGCGTTGGCGAAGGTAAAACTAACAGATTTTGAATTTTGCATTTTACTGATACATGATGATTAACTAAATTGTATTGTTTAATTTACAATTGTAAGTTGCTGATGGATCATTCTCTTAACAATCTTGTGCTTAAGCTTATCAGAAACAGTCATTAAGTTAGGTAAGCTAGACTGTCACAAGCTTTTGTGCATTATTGATCAATAATGTCAATTCGCCAGAA

>SlERF5-9

ACATAAAGCTCGTAGTGTTAAATAGTTAATTACATTATTTCCTTACTATATTTCTAAATTACAAAAATCATTTAAAATTTATGGACTTCAGATATATCATTGGACTTTCGAATGTATAGGAGAAGGTTGTATCCAAGAAGAGAGGGATGTATCAGAGAGGAGATAGAGATGTATACCATAGAGGAGATTTGACACCCGGATACTGATTGGAGGAATGTATTCGAGAGAGGGATCGGGATGTCTCAGCGATAGGAAAGTGATATATTCAAGAGAAAATTTCTTTTGAAAAAAAAATAAAAATTTTAAAAATTATAATAAAATAGTATATATATATTTAAATAATTTTTCGTTCATAAATTATATGTCCAATTCCACATAAAAAGTAAATTTCCTAAACTATTTATATATATCCCATTAATGAGTGTGTATATATGTGTATGTTTGTATACCTAAAGTCCCATTAATAGTGTGTATGTGTTTTTCATATATATCCAAAGTACTATTCTCTCCATATTTCACAAAAAAAAGAAGATTATTTATCGATTTGTATGATTTCACTTTTAATTAACGGTTTCATATTCAAACAAAATATTAAATATGTATGAAGAGAATCTTGACGAAACACTTCTCCTGATTTTGTAATCTTACTTAATGTGAATTTAAATTAATTATTTTTGACGTAAATATTGATTATCGAATAAACAATAGAATAAAATAATGGGCTCAATTTTGTAACAAAATCAGACTTTGCTTGTATAACTAATTTATTATTATTAACTCTCTTTTCCTCCTAGAATAATGGGATATGAGTAATGGTCCATTGTGATGAAATCTGCATGTGAATCTGAAAAAGAAAATCTGATATTTTGATTAAAAAATTGATCAAGTTGAATTATATTAATTAATAATTAATTAACTGAATAATTGCAGTTGATATTTCTACAGAAATCAATACTTTGCGGCTAGTTTTTTTCTTCTTCTGAATATTATGTCGATTTAGTCAATTCCTCCTTCGATATTGGTGGGGGTAATATTGGGTATATCGTTATTATCGTTAACTTAATATTTTTATTAAATTATTTAATTATAAATGTTTTGTTTTTTTCGGTGAAATTTAATTTTTATTATTTTCTTCGCTTTTACTTTTTTAATTTAATTGAGTCTAACAAGATTCAAGTACGTAACATTTTATTATAGATTGAGATTATGTAAAAGTAAAATACAATGGAACTTTGCTGATTTTGTTTTGCTATTCGCAATAACGAAAAATAATGAAAATAAGTTGATGTGCTGCTTTTTTCGTTAATTCTAAATGTACTTCTAGATCAATTCAATCAAACTAAAAAAAAAAACCAATCAAACACGATACGATATTTTCACGAGTTATCACCTTATTACATATTCAAAAAATATCACAAATATTCTTTTCACATAGTATAAATCCCTTATTATTATTATCTTAAAAGAGAAATTCAATAAGTAATTGTATATAGGATAAATTAAAATTGATGATTATTTAATATTTCTTTTTATAAGAGCCATAAGATTATTTCTACTAAAAAAATTAAACTACATTAATATCATAAGAAAATTGTACATATTTAATCTAGTATAATGAAAATTATTTTTGGACCATGAGAATATACAATAATTAATCAACTAAAAAAATTTGATCATGTCGGCTAAACATTCTGTCATATACAGGTAATTATAATAACAATAGGGCAAAATAACAAAATGTGCAGCTATTTTTGATGATTAATTAAATTAAAATTTGAAACCTGAAAGATACAAGCTATATTAATTAATACTAATATAATGCCTCTATAAAAAAAATATTAATTAGCATAGAATTTGACATTTTTTTGGCTGGTTGTTGAACAAGAAAAAGTAATTGCTATACCTAATGTGATGTTGTGTTTGGTGTTATTAAACTTTGATAGTTGCATAAATTATGAAAAAATTGTGGATTATTTTATTTGAGATATAATATAAATATGGTATTTTTAATTTTTATTGATAGACCTTGCATTATTTTATTGTGAGTAGTTATATATTTTGAATTGTTGAATTCTGTGTCAAGAATATTTTAAAAAATTATATTTTTATTTTTACAGAATATTTGCGTATATTTTTTTTTTAAAAATAACAGTCAATATTTTATTTTTATTATAATATGAATAGGATTATGAGAATACGTGCATTACAAATATATGACTAAGTCTTTCCAATAAGAAGTTTCACATGATATATAATCCCTACATTAGTATATTTTATAATTGATTGATTAAATAATTAATCTTAGTTGCTTTAGTTACATGTAATAAAAAATTGTCAATTAAATATTATTTATCAAAAAATTATATATATGCCAAATCATACAAAATACATACATAAAAAAAATCAACTTTTATATACATATTAAAATCTTAAATATTCTTAACGCAATTAATCCCAATTTAAAGAAAATAGGCCCTAAAATTAAAGAAAGTGACCAGATTCATCCCCTTAGACCTCAGAAAAAATAAAACAATTAAATGAAAGAAAAAGACAATTAAGAATAGAAAAAAAAAGAAAAAGGCAGCAACCTGGATAATTCCTAGGGCCAACATCAAGTTGATGAAAACTAAAGCAACCAAAGCAAAGATAGTGACTCTTAATCAAGTGGTTGAAAATAGGGTCGGAGTCAGAATTCAGGTTACGAGTTTTATCGAATTTAATAATTTTGATTTAAACTTAAATTCATCTTAAAAATTTTATTGGTTATGTATGAATCATAAGTTAAAATTTAATAATTGAAAAAAATAAAATTTTAAATTCTAATTTTGCCTCGTAGGAATACTTACTTGCGCTATTTACAGCCTTTTTGTTTCCCACCCAATTTCTGGATCCAATATTGGAGCTTGCAGGGTTTATATTTGGTAACACTGATTTTCGATATAGTATTCAGAGTTTTCATTCAAGTCGCAATTATCTCTACATGAATCTTCCAACATAATTTCTTTCATATTTATATTCAAATTTAAAATTTTTAATTAAATCGAAGCAATCTCACCACTGCAATACAACTTATTGAGATTGCGTTGTTAGAGTTGATGTTGAATACAAGTGAAGTATATCTAAGTATACACATAGTAGTTACTATTACTATAAATAGTGAAAGTATAATAATACAAAAATCAGATTAAAATATAAAAAATCGTTGAAAAATATGTTTAAAAAGGGAACAATAAAGTCAAACTATGCTATTTTTGTCGATTACGTGCCATATTTTGCTAGCTACTTTCTATTCATGTGATATACATACGTGATACAACTTTTTATGTCTTTGACAATACTAATAATTTTTTTCTTTTCTTGGTTAAGTGTTTAAATTTTGCTTTGGCTTATTTTTTTTCTCTATAAAAAAAATAAATTAAACACACCCTTTGACTATACATTTTTGAGTAGTTCTTAAGAAAATATTGGAAATAATTTCTGGCCATGAAATTTGATCATTTAATTTATCCGGTTAAGATAAGTTTTTTTTCTTTTGTAAAATTTCACTTTCACTCATCCAATTTCGAAAGAAAATTCAGGCAAAATGGCAATTAAAACATAATTTTGAATTCTAAAAATTATTTTTCAACACAAATATATATATAAAAAAAGGCAATATACATAAATAATATCTAATGTAAAACCATTTTTTCATGTTAATGTAGGAATAAACACTAGCTATATATGTTTTAGAAATAAGTGGAGAAATATTTTTGGGAAAAAGTTGGGATTGGATACCAATAAAGAATATATACTATAAGTATTAGTTTTTTTTTTTTTTACTTAATTAACTAGTATTTGGTTTGGGAAACTATTCCATTTTTTTTTTTTAAAAAATGGAGTACAAGTATATATGGGTTAAAGTGCGATTCTAATATTGGATGTATTAAAAATGATATGACAATCAATTTTTCATCAGATTCAACTCAACAAGAATGAACCAAACACAACCCTATGTCACTTTCGAGTAATGTGAACCCATATCCCTTCCATTATATACTAGAATAATAATTTATTTTTCAAATTATTAATTTTATTTATTGTGTTCAATTACTTGTCAGTCTAAATTATCTATTTTATATTTTAAAGGTTGTGCAATAATTTTACACCATCAATTCATATTACAAGTATAATATTATGGAGAAAAATTATTCAAAATAAATTCTTACTTTAATAGTGTTTGTCATTGTCATTATACTAATATTATTATAATTTTTGTTTCTTATATTTGATTATCACACCATTTTATTATTATTTGCTTTAGTTTTGTCATTGCTACACTTGAGTTTGCTGTATTTGAGCCAAGGATCTTCCGAGAACAATGTGTCTACCTTTCATACCTTTATCCTCTTCAAAATCTAAATTCAATTTTACTGAATATATTATTATTGTTGTTTTAACTTCTAAAGTTAAAACTGAAAACTTCTCAATAAGAAAAGAGCTTATCGATAGTTCATAAGTCTTAAACCTTAATAGATAACAAAAAGGTGCATTCCAATTCTCTCAAAAAATTTCCTTTTAAATCTATTTATGTAGATTACAAATGAAAAATACTAAATAAAAATATAATTATTATATTATCAGTTTACTTGAATATAAAGTTTTATCTAAAATAGTCTCTCTATTCTTATAAAATATAATTAAAAAAATAACCACATCATTCTCTTTAAATAAAATTTCACTTCATATATATATTGTTTTTCATTGTATTTCCTATAGTATCAATTTTCTTCATTCTTTCATGTAAAAAAAATGAAAGAAATGTTATTAAAAAGAATGAAAGAAGCAACCTAGAAATAGGGGTCCATAGGTGGAGGATACATCATGAATTACACTAAAAACATCATCTATTTCATTATTATTATTTTGTTATTAAAAAAGAATATCTTTAATCATCCATTATTATTATTATTATATTAAATAACAAGTGGTGTACCCATATATACTTTTAAAAAATGAAAATGGATAAGCCCCACAAGTGGCTTCCTTGTTCTAACCAAGAAGGCCCTAAGAAACCTCCATTGAAATTAAAATTTTCCACCCTTTATAATCTCCATTTTCACTCTTCATTTTCCACTCAATTCAATTACCAACAATATTTTTCAAGAAAAAAAATGGATTCTTCTTCACTAGAAATGATAAGACAACATCTTCTTGATGATGTTGTTTTCATGGAAACTTGTTCTTCTTCTTCTTCTTCTTCATTAGAAACAACAAGTAGTACACTTTATTCTCAAACCTCATCGAATTCGGAATCTTTAGAATCATTAACCTCTGAGATCAAACTTGAAAGCAATTTCTCTGTTTATCCTGATTTCATCAATACACCTCAAAGTTCAAATCTTGAATCTGTCTCTCGTTTTTTCGATAACTCAACTATTGAATTCCAAGCTAAACCCCAAAAGAAAAGAAGTTTCAATGATCGAAAACCTTCGTTAAACATTTCGATTCCTTCTGTTAAGAAAACAGAGGAACCAAAAACAGGGGAAGTAAAAACAGGGGAACCAAAAACAGAGGAGCCAAAAACAGGGGAAGTGAAAACAGAGTACTCTGTTAAGGAGAAAATGGTAGAAAATTCGGAGAAAAAGCGATACAGAGGAGTGAGACAAAGGCCATGGGGGAAATTTGCAGCGGAGATTCGTGACCCAACTAGAAAGGGGACACGAGTTTGGTTAGGAACATTCGATACTGCAATGGATGCAGCCATGGCATATGACAGAGCAGCATTTAGGCTCAGAGGGAGTAAAGCAATCTTGAATTTTCCACTTGAGGTAAGCAATTTCAAGCAAGAAAATCATGAGATTGAGAAAAATGTTGTGAATTTGAATTCGAATACGAATTCTTGTGGGAAAAGGGTGAGAGGGGAAATGGAGAATGATGACGGAATTGTAATGAAGAAAGAGGTGAAAAGAGAACAAATGGTGGCAACTCCATTAACACCTTCAAATTGGTCTTCAATTTGGGATTGTGGAAATGGAAAAGGTATTTTTGAAGTGCCACCTTTGTCACCATTATCACCACATTCAAATTTTGGTTATTCTCAACTTTTGGTATCATAG

>SlERF3-10

GACACTCTCTGATGAAGTGCCCAGCTTTTTCACACTTGTAGCAAGTATCACCTTGAGCAGCATTTCGAGTATCATTTGATCCTCTTTTATAAAATTTGTTTTTTCTCACAATTTTTTGAAATCTACTGATGAGATATGTCATATCATCATCACTGGGATCTTCATCTAATTTGTACTTCAGCATCAATGACTTGTCCTTCTTGGCTTCCTTCTTTGATAAATCGTAATTTCGATTCATCTCATGTGTCTTCAGATTACCAATCAAAGCATCCATAGTCAGCACCTTCAAATCCTTGGCTTCTGTAATGGCATCAATCTTGATCTCCCAAGACTTTGGAAGAATTCGAAGCACTTTTCTGAATTGTTTGGTCATTCTTATAGGTTCACCCAGACTTCGCAGCTCATTTGTAATGGAAGACAACTTGGTGAACATGTCATGTATTGTTTCTCCTTCCTTCATTTTGAAGTTCTCATATTGTGAGGTGAGCATGTCAATCTTAGATTCTTTGACTTGTTCAGTTCCTTCATGTGCAGTCTTCAAGCAGTCCCAAATTTCCTTAGTAGACTCACAGGCTGACACTCTGTTGTACTCATCAGGTCCTATCCCACAGACAAGAAGAGTTTTAGCTTTGAAACCTTTTTCAATCTTTTTCCTGTCAGCTTCGTCATATTTCTGCCTAGGCTTTGGAACAAGAATAGTCTTCTATCCATCCTTTTCTTCCATCGTCAGAACAAATGGTCCATCCAGTACAATATCCCATAACTCACTGTCTTCAGCCATTAGGTAATCGTGCATTCTAACTTTCCACCAACTGTAGAAATGTCCATTGAAACGAGGAGGTCTGTTTGACGACTGACGTTCTTCGAGGTTAAGTGGAGATGTCATTCTAGAGCAAATATCACTTCCTTGGTGTTAACCAAATAGATAGTGTCTGCTCTGATACCACTTGATAGAATATATGCCTTCACTTAACAAGTAATGGACCAAGTCCCTTACTACACTAATGAATAAAACAGAAATTTAAATGCAGTAAAATCAACACAATGATTTTACGTGGAAACCTCCTTGCTTAAGGGAGTAAAACCACGACCTGTCTCACAGGATTTTCAATCGTTTTTCACTAATCTTCAAAAGCAAAAGTAAAACACGATTACACCAAATGTAAGAAAGATTTTTCAATCTTACAGTTAAGCAATAGTCCCTCTTGCTTAACAAGCCTAAGTAGAAAACTATCTACTCACTAAGTTATCCCACTTGGACAACCTAGACTTTTAACACTACACAAAAATTCCTTTATAGATTGAGGAATGATTTACAGTTTGCGAACAAGAGAATGTATTCCTAAACAACTATACGAAAAGCTCCAGAGATTGTTGTTGTTGTTGGAATACTTCTGCCTTTGTTTTCTTGCAGCCTTTGCAAATGTTCTTGAAGAGGTTTCTCTCAAGTTGCAAAAACTACTCAAAATGTTTAGGAAAGTGCCTTTTATATGGGCAAGTCACTTTCCTAAACCTCTTTGCCATTGGCTGGAAAAGTCACACTTTCTGACGCCATCGGGAAGTGTGCACCTACTTTCTGTACCGTCTCCAGCTGGCAATCAACTGGCTCTTCATCATGAGAGTCTGGTACCTCTACTAGGTCCCTGAGTTTGTTTCATCTGCAATACTCAAGCAAGAACCCTGCAATACTCAAGTAAGAAACTGTACCTTTACAAGGTCCCCAAGTTTGTCAAATCATCAAAACTACAAATAACATTTATATACATTGATTATACAATATGTATAAATTTTTAAGTTTTTTTTAAATCCCGTACAACTCATATTTCTTTTAAGCATCTCAAAATTTAAGATAGGAGCTTCTTTTGATGTTAAAGCTTGATAATATCAAAATTTGAAATAAGCTTTAATGATCGAACGTTGATTCTTTTAATGTGGGCCAACTCAAAACCTCCTCTAAAATATTCAAAATTTTAAATATGAGCTCAAGAAGAAGAACGATGGAAGGAAGAACAAGGAGGAGAAAGAAGAAAAGAAGGCAACCAAAATTATGCTCCCTTCGTTCAGAAATGTTCGTCATGTTCTGCTTATCGAAATTCGAAAGTCTTTTTGATTAATTTTTAAAGATAAATTAGATTACATTAATTCGATGTTTTAAACAAAAAAAATTAAATATTCTAAAACTAGATGAAAAGTACTATAAATTACAATTTTTTACATATTAATATGATGAAAAAATGCATCTTAAAATGTTAGTCAAAGTTTTTATAATTTAAATCTAAAAATAAAAATCATGACAAACAATACCGAACGAAGGGAGTACTTTGTTGTTCATAAATAGAGCCGAATGATATCTTTGAGTTATTAATGATGATTGAGTTATTTTGAATAAAAAAATAAATTTAATTTGATAAGTATTTTTTGTGTAATTTATCAATAAATAAATGAATTATTAATTAATTTCAATTTAAATAAAAGAAATAACTAATAATTTTATAGATTAATGTTATGGACAAACAAGAATGGTACGTGACCTATATTATTATTTGTATTATCTCCTACATTATATATTGTTTTAAATATTACAAAGCGTTAATGCCGTTGAAAAGTAGGTTTGAGAGGAACAATTAAGGGCCTATTTGGATGGGCTTAATAACAGCAGCTTTAAAAAAATACTTTTAAAAGTGTTGAAACTTATTTTTAAAATAAGCAGTTATGCGTTTGGATAAAAGTGCTGAAGTTGTTATGTCAAACCTGAAAAGGGAAAAATGGAAGAAAGAAATGTTAGGGTTATATGGGTAATTTGGAGATTGTATAAAAATATTAAGCACAAAAAGATAAAAATGTGGTCAACTTAAAATAGCTTATAAGCTAAAAAAAAAAAACACTCCTATCCCAGCTTTTAACTTTTGGCTTAAAATAAGTTTTTTTTAACTTAAAATAAGTTGTTTTGAGTATTGTCAAATAGCTAAATAAGTCAAAAACCAGCTTTTAAGTCAGTTTGATCAGCTTTTAAGCTGAGCCAAACAGGCTCTAAGTCAACTGATGCGGTTCTTTTTGCCTTTGGTCATTTCAACGTGTTATGTTTTTTGTTTGATTGCAGCTTCCTGTTTCTTATAAAATAAAAAAAGTTAAAATTACGTGCAATGATAATTTATAATATTTTCTTACAAATGAATTAGTTTCATTTAAAAATAATGATAGTTGGTATCATTGATTGATAACCTGTAATAAATAGTAAATAATCTGTTAAGTTAGATTATTATAGACATAACACATAAATATATTTTTTAAATTTAGCTTTAAATCATATTTATACACTTTAATTTTGTATGTGCACAAGTAGACACATAAACTTGTATAAAGTTGAACAAATAGACACACATGTCTTACATGTCATCCTACATGACATTTTTTATCCCGCGTAGTGTTCTACGTGTATTATGTCATGTAGGAGTCACGTGCTTATTTATTTAAAAGTTAAATAGTTAAAGTATCTGTTTGTACATTATGAAAGTTGAAGATTAAAGTTAAAATTTTAAGTCAAATTTAGGTCCAACATATGTGTTATGTCATTATTATATTATACTCTCTTTATTTCAAATTAATTAAATAATTGAGATATTTTTTTATTTTTAAATTTATTTATTTGTTCAATTTGTAAGATCACATTTAGATTTTTTTTTAATTTCACCCTTCATTAATTAGTACTAAAATTATTTATTAATTAATGTTTAATTATTTTAATTATAAATTTTAACAATAATTAATAACCGGAAATGAAAAACTTTACCTAAGTTATAACTCAATTTTTTTCTTAAAGTACATAAAATACCTCTAGAATAGAAAAATTTTAATTAATATAAAATAAAGAGAGTATAGTTATGTATAAATGTTTTTTCAAAAGTTATAAGATATGCATTAATTGACATTGTGGAAAATAAATTTATGCAATTAAATTACTTAGAAATTAATTACAATAAGCTAACAAAATGACCAAAATAGCACATTTCATCACTTTGTTTTGTTTGAAACAGCAATCTTTAAATGTATTTTAATATTTCCCAAATTTCAAAATAATTATCACGTTTTATATTTTAAAAATTAATTTGATTAATTTTCAAGACTAACTTAGATTAAATTAATTTGTTATTTTGAAGCAAAAAATGCAGATATTAAAAAAAAATACGGAAAAATAGTATGAATTTGAATTTTCTCATATCAATGAAAAAAATACTTCTTAAAATATTGTTAAAATTTATTTTAATTGACGCTCAAAAAGAAAATTATGACGAGTATTTTCATGATCCTAAAATGTACTATAACAATGTACGTAAATGTCGTCCATCTTTAGTTTTAAACATATTTTGTGAAAAATTAAAAATTAATTTTTTTTTTAAAAAAACATTCTTTTATTGAAATTGGAGTTGAATTATAACATCAAAAAATATATATTTTTTATTTAGTTAACAAATATATAGATTTTCACAATCAAATTTAAACTATTTGTTTGTCGAAACAAAATATTAAAACAGAAGAAGAATTGACTTAATAGTAAAACTATTCAAAACTAATTTTAAACACCCAATATCAACATGGGTTGTGCTGCATTAGAGGAAATATTTCATCCTTAATCAGTTATCTCGAATTCGAGTCCTGAGTATGAAGAAAATTTTGTTGATAAGATCACCCTCAAATAGTGAATAAGCAATCTGAATTTAACCGAAACTCTAACGTGAACTTGAGACACAAGAGCGGATTTTTTTTTTTAACTCCCGATAAATAATGCGAGGGACTAAGATTTTGGATTAGGGCTGAGGATGCTTCCTCTCTCCACCTGAGAAGCCTCCTAAGAAACCTCATCTCTTCATTTCTCCTCTTTATAACCCATCCTCCACACACTCTCTTTTCCACTCAATTTTCTCTTCAACTTTGAAGTGTTAACATAAATAGTTGACTTCTCCAACACAAATTATTCTCTTTGGTTGATTAGTGGAAGAAAAATGGGCTCTCCACAAGAGATTTATACTTCACTTGATTCCATTAGGGAACATCTTCTTGATGATGATGTTGCTTTCATGGAATATTACTGCTCTAAATCTTGTTTCTCTTTTCAAACTTCAAATCTTGATCATACCTCAAAAACAGAGTACGATGGTTTTTTCAAATTTGAGGCAAAACCACATGTTATCAGTTCAAATTCTCCGAAACAGAGTAACTTGAGAGAACGGAAGCCATCTCTGAACATTGCGATACCCGCGAAGCCAATTGTTGTTGTAGAGAATGTTGAGAGTGAGAAGAAGAAGCATTACAGGGGAGTTAGACAGAGGCCATGGGGGAAGTTTGCAGCGGAGATCCGTGACCCAAATAGAAAGGGGACTCGAGTTTGGTTAGGAACATTCGATACTGCTGTGGATGCAGCTAAGGCATATGACAGGGCAGCGTTTAAGCTTAGAGGAAGCAAAGCAATATTGAATTTCCCACTCGAAGTTGCAAACTTTAAGCAACAAAATGATGAGACTAAAACAGAGATGAAGTCATCAGGCAGTAAAAGAATGAGAGGAGAAACAGAGGAATTGGTAATCAAAAAAGAAAGAAAATTACAAGAAGAAAGAGTAGTGCCAATGGCATCTCCATTAACACCGTCAAATTGGTCGACGATTTGGGATGGAACAGGTATTTTTGAGGTGCCACCATTGTCACCCTTGTCTCAGCTTGTAATGATATAA

>SlERF3-9

TGTGACGGAATGGCAGGACGAACCGTCACAGATGTGACGGACCGTCACAGACCCTTGGTGGAAATTTGGGTCTCTGAACTTTGCGACGACCTGCAAAACGGACCGTCGCAGGCACGACGGCCCGTCACAGGTTGCGTAATCCCAGTGGAAGTCGGATTTCTTGATACGTTTTAAGGGACGTTTTTGACTATTCTTGCCTTATTTATAAAGTTCGTGGGTTTATATTAATAACTCAAATTCTTGGGGGTTAAAAGAGGTAACCCTAAGTTAATTAGTGGGGTATTATTGTCACCTTTTATTCTTAAATATATACTAATTAGGGTAAAAGAAAGAGGGTTTGAATAAGAAAATAGAAAGAACAAAGACAGACAGAAAAAGAAAGAGAACGAGTAGAGAGAGAGAGAAACGAAGAGGATAGCAAGGATTTTGAGAAGATAGCTTGTTGATCGCAATTCTTCGGTGGAGGTAGGTTATGGTTATTTCATGCTATTCGTAGTAAACTCTTAATAGCGAATGATATGTGTTGGTAGTATTGTAAACCTACTATATGCTTAATTGTATGTTTGCATGAATATGATTATGTGATTGTGATAAGACAACATGATGAAAATATTGAATCCCAAATCTTGAAAAGAAACTTTAATATACATTATTACTGAGGATGCCTTGGTATAGAAGAAGGCTTGATGAATTAAAGTAATGGGATTGATGATGTCTTGGAATAGAGAAGGCTTGATGATTTACAGAATGATATTAGTGGATCGGGTGCCACGTTCCGGTACCAGGATAGTATATGAGGATCGGAGTGTCACGTTCCGACACCAGGATAGTATATGGATCGGGTGCCACGTTCCGGTACCAGGATAGAATATGGATCGGGTGTCACGTTCCGACACCAGGATAGAATATGGATCGGGTGCCACGTTCCGGTACCAGGATAGAATGAGGATCGGAGTGTCACGTTCCGACACCAGGATAGAATATGGATCGGGTGCCACGTTCCGGTACTAGGATAGAATGAGGATCGGAGTGTCACGTTCCGACACCAGGATAGAATATGGATCGGGTGTCACGTTCCGACACCAGGATAGAATATGGATCAGGTGCCACGTTCCGGTACCAGGATAGTATATGAGGATCGGAGTGTCACGTTCCGACACCAGGATAGAATATGGATCGGGTGCCACGTTCCGGTACCAGGATAGTATATGAAAATCGGAGTGTCACGTTCCGACACCAGGATAGAATATGGATCGGGTGCCACGTTCCGGTACCAGGATAGTATATGAGGATCGGAGTGTCACGTTCCGACACAAGGATAGAATATGGATCGGGTGCCACGTTCTGGTACCAGGATAGTATATGAGGATCGGAGTGTCACGTTCCGACACCAGGATTAGTAAAGAGAATGAATCTTGAAATATGTTAATATTCTCAATTTAATGAACCTAAATCCCAAATGAGTATGATGGGGAGGCGTGGGTCCTCATTGATGAGCTTGGTGTTATGACCAAGGGTTATGGTAATTGTAAATGCCGCATGTTGAGTATAGTAGTTGATTTTATGATATTATCTGATATATACTGTTTTCTATTTTGAGTTGGCCGATGATATCTACTCAGTACCCGTGTTTTGTACTGACCCCTACTTGTATGTTTTCTTTTGATTATTTGTGGAGTGCAGCAAACGTGTCGTCATCTTCAACTCAACCGCAACTCTAGCCAGTCTTCATTACTCCGGATTTCAGGGTGAGATAATGCTTCTAGCTTGGACTGGATCTTCCTCTTCATGTCTTGATGCCTTGAAGTTCCGGCATGGACTAGCTGTTTACGTATTTTAGCTTCTTAGATACTCTTAGATTTAGTAAATTGAAGTAGATGTTCTTGTGATGATGACTTCCAGATTTTGGGGATAATAATAGTTGTTGAGTTTTTAGAAGTTATATAATTGATTTTTATTAATGAGTTTAAGCCTTCCGCATTATTTTATGTTATTATTGGTAAATGATTGGGGTTAGATTGGTTGGTTCGCTCACATAATAGGATAAGTGTGGGTGCCAGTCGCGGCCCGTTTTGGGTCGTGATAACTCTACTTTGTCACTTTCATTGATGATTGCTCGAGAAAACTTTGGGTCTATGTCTTGAAGATTAAAGACCAAGTGTTAGGTGTCTTCAAGCAGTTTCAGGCTTCAGTTAAAAGAGAAACTGGAAAGAAATTGAAATATAATCGTACTGATAATGGTGGTTAATATTGTGGACCATTTGACGAATACTGCAAGCATCAAGATATTAGAACCAGAAGACTCCTCAACTTAATGGTTTGGTTGAGAGAATGAACAAGACCTTGATGGAAAGATTAGATGTTTGCTTTCTGAAGAAAAGTTGTCGAACTTATTTTAGGGTGAGACTTTAATGAACGTTGCACATGTTATTAATCTATCTCCAGCTGTTGCTTTGTAAAGTGATGTACCAAATAGTGTTTGGTACGGAAAAGATGTTTCCTATGACCATTTGAGAGTATTTGGATGGAAAGCTTTTGTACATGTGCCAAAAGATGAGAGGTCAAAGTTAGATGCCAAGAAAAGGCAGTGCATCTTCATTGGATATGGCCATGATGAATTTAATTACAGGCTATATAATCCAATTGAAAAGAAACTTGTGAGAAGTCGTGATACTATTTTCATGGAGAATCAAACAATTGAAGATATTTAAAAAGTGGAAAAGGTAGAATTTTCAAGTTTGATGGTATAGTTCAGCATGATGATGTTCTTCACACAGGTGTGCATGATGTTATTGGGTTTGATAATTATGGTGACACCCAGAATCATGTATCGAATCAACATATTGATTTTTATAATAACAATGATATTGTTATTGATGATCTTATTACTCATGAAGTTATGGACGAATCAAATATTTCACTTCGAAAGCCATAGGACAACGATTTTCTTTCTCTCGTTATTCACCCAATGAGTATGTGTTACTCACTAACGGGGGATAACTGAATGTTATGAAGAGGCTATGGAAGATGAGCACAAGAATCAATGGATTGAAGCATGCAAGATTAGATGGACTCTCTGCATGAGAACCACACCTATGAGTTGGTAAAATTACCCAAGGGCATGAGAGCTTTGAAGAACAAGTGGATGTTCAAAGTTAAAGTTGAAGAACACAACTTGAAGACCAGGTACAAAGCTAGATTGGTTAAGGATTCCGGTCAAAGGAAGGGTAATGACTTTGACGAAAGATTTTCTCCTATATTTATCAATGTCATTGGTGACATAGGCATGATTTTATCCTTCTATAAATAGAGCATTTTTGCTCATTTGTAGAACACACAAAGTTAGAGAGAAAAATCATATCGGAGAGCAAAGTGAGGTATTCCATAGACTATAGAAGAAAAATAGTCTGTGAAGAAAAATAAAGTGTGAGCGATATTTTAGTAAGGTAGAAAACAAAAGAGTGTTGTTCCTTTTGAGTGTATAGTAATCACTTTGAGTATTCTATTCGTGACTACACAGTGTAAAATTACTTACTATAGTAATATCAGTTGCTCCACTTGACCCATGATTTTTTTCCTTATTTAGAAGGGTTTTTCACGTAAAACTTTTGGTTTCATTATTTTCCCATTTTATTTCCATTACTTTTACCATATTATTTTTGTGTTTATCCGCTTATTCCCAACATTTACAATGCTTTTAAATGCATGCATTAGTTGTTTAAAAATATTATTTCTTCCTCAAATTTCTTTTTCACTATTTCACTATTAGTGAAAGAGAATTTTGTAAATAAATAATTTAATCCCTTCTTCTTAGTTTTTTTTTTGAAAAAAAATTATTTATCTGATTTTGATTCGATTTAAAATTTAAGAAAAAAATAAATTTTATAGTCTTAAATTAAAGATGTATATAAACACTTTCAGAAGTTAAAAACTGACTTAGACTTTTAAGTCAATTCAAACGGGCTCTAATCTTCTGTCGAGATTTATTCTTATATCGCTCGAGTACAAACATCCCTAGGATGATGTTTGATAGTTGAATAGAGTTATACAGTGGCGCAACCAGTAATTTTAGGAAGGGTGTCCAAAAATCTCATAGCTCCTAAAAATAATTTAAAGAATGGGTGTACTTAAATTTTGTAAAGCAAACTACATAACATGATTAATTTTTTAAATTAAAATGTATCACAAAATTTTAAAATCAAACTACATAATATTTAGCTTTATTATTACGTTTATTGATAGGAAGTGAAGAAATAAAGAAAAAGAGATATATTTCAGTTTAGAGTGAATTCATTTTTATGGTTCAACATTTCACAAACCTCTGATGACTAACAACAAAAAATGAAAAATAAATAATAGAATCAAACAAGTTATATTAAAGAGAATTTATATAAAGTATTTTCACTTTTAATAGCTTAATGGAACAAATAAATAGGATTTTCTTAATAACTTTAAATTTAATAATTCTTTTAAATTTTATTTAAATTTTTAATAAAAAGAAAATAATCAAAGTCAAATAACGGGAATTGAACCAGTGAAAGTTGCAAAGAACTGAGTAGGAGTTGAACGACTAAAATTACTAAGCTAGAAATTTTTTATTGTTTTTCAAAAGGTGTCCAAATTTGTGTTATTTAATCATTTAAAATATTATCTTTTATAAATACGATTTAATTTTTTGACTAAGGGTATCCTATGCCCCGGAAGTTATATATGTATTAATAATGCCCTGAAGTTATACATGTATTAATAAAGTGAGAATTAATTATGCGGATATTTGTTAACAAAGATAAACTATGAGAGGGACTAAGATTTTGGATTAAGGCGGAGGATGCTTCGTTTCTCCACCTGAGAAGTCTCGTAAGAAACCTCATCTCTTCATTTCTCCTCTTTATAACCCATCCTCCACACACTCTCTTTTCTACTCAATTTTCTCTTCTACTTTGAAGCTTTAACACAAATAGTTGACTTCTCCAACAGTTTGTTTGATTAGTGGAAGAAAAATGGGCTCTTCACAAGAGATTTATACTTCACTTGATTCCATTAGGGAACATCTTCTTGATGATGATGTTACTTTTATGGAATATTACTGCTCTAAATCTTGTTTCTCTTTTCAAACTTCAAATCTTGACCATACCTCAAAAACAGAGTATGATGGTTTTTTCGAATTTGAGGCAAAACCACATGTTATCAGTTCAAATTCCCCGAAACAGAGTAACTTGAGAGAACGGAAGCCATCTCTGAACATTGCGATACCCGCGAAGCCAGTTGTTGTTGTAGAGAACGTTGAGAGTGAGAAGAAGCATTACAGGGGAGTTAGACAGAGGCCATGGGGGAAGTTTGCAGCGGAGATTCGTGACCCAAATAGAAAGGGGACTCGAGTTTGGTTAGGAACATTCGATACTGCTGTGGATGCGGCAAAGGCATATGACAGGGCAGCGTTTAAGCTTAGAGGAAGCAAAGCAATATTGAATTTCCCACTCGAAGTTGCAAACTTTAAGCAACAAAATGATGAGACTAAAACAGAGATGATGTCATCAGGCAGTAAAAGGGGGAGAGGAGAAACAGAGGAATTAGTAATCAAAAAGGAAGATGAAAGAGTTGTTCCAATGGCATCACCATTAACACCGTCGAATTGGTCGACGATTTGGGAGAGTGGAGATGGAAAAGGCATTTTTGATTTACTTTGA

>SlERF5-8

ATGAAAAAAATATAAAATTAGCACCAAAAATTAAAAAATAAATACAGTAACATTAAATCCAACAATTTCAACATTTTTTTTATTTTTATTTTTTCGAAAAATTCCAAAAATGAGTTTATCAACCAAAAAATATGAAAAAAATATAAAAATTATGCGAAAAATTCACAAATAAAAAATCGAAAAATATAAAAAAATTGTTGAAATTATTAAATTTAAGTTTACTATATTTATTTGTGAATTTTGACGTATATTTTTTATTTTGTTATCATATTTTTCCCTTTCTATTAAAAAGTTACTCATTTTCGAGATTTGCCGAAAAATATAAAAATTTTAAAATATTGTAGATTTAAATGTTACTATATTTATTTGTGAACTTTTGGAATTAATTCATATTTTTTTCATACTTTTCATATTTTTTATTAATTAATTACTCATTTTCGAGTTTTATCAAAAAATAAAAAAATTGAAATGTTAGATTTGAGGTTACTATATTTATTTGTGAATTTTTTGGCGTTAATTTTATATTTTTCCTATTTTTTGTTAATTAATTACTCATTTTTGAGATTTACTGCAATAATAAAAATTAAACAAAATTGTTAAAAATTGGTCGGATAGTGAATTTAAAAGAAGCTAATTTATGGGTTGGATTAGGTGTTTACGAGTCCGGATGTCTTTCCATTTTCATATAAATGTTATGTGGTAAGGTCAAAATGACATGACAATTCCATGTGACATTTAAAGAAATGAAAAATGAGTTGGATATGTCCAACATGACAACTAACGCCCATAAGGGCATATTTGGACTAAAAGTTGGACGGCGAGGACATGAGTGAACCAAACTTTAAACGGAAGGTATATCTAGACTTTTTCAAATAGTTTAGGGCATATTTGACCCTTTTCCCATAATCAATACTATTAATTATGTAAACTTTCTTTTTGTCTTAAGTAATAGAGATATTTAATATCAATAGATTTTTTTCGGTGATAATATAATTGAGTTAAGTTATTATATTTTGAATCGGTAAAATTTTATCGGATATCTATTAGAGTATTGTTGATTATAAATATAAGAAAATGTTGTATTGTTGTGGTTTAATATAATATTAGATAAGTTGATGATTATATTGCTATCGTTAAATTCTTTATTTGTTGTTTCGTGTTACAAGATATACGAAATCTCATGAATAATTAACTTCTCAAAAAGTGTATTATTAGAATTTAGAAATGATAAGTTTGGTGTACTAAAGCAAATATTAGCAATTATTTACTCTCATGATTATTATAATTATTATTATTAATAGTTATTATAAACCGTTTTTACCCTAAGAAATCAGACAAATTTGATTTTTAGAACCAAGACTTTTTTTCTTCTTATTATTTTAGAATTTATAAACTTAACTATTTCATCATTAATTAATTACTATTTTTCTATCATTACATGTATCACTTAATTACCATGTAACTCTATTCATTAAGAAAGAAATATAGTCTCTCTCTTTACTCAAAAATACCATAATTTAGTAGGTTATAAGTTTATCTTTAAAATTCAATATCCAACTTCAAATTGAAATTAAACTTTTCAAGATCAAAATTGATTTTTTTTTAAAAAAAATATGTCAAAAAAGAAAAAGTAATTCTCAAATCAAATGAGTCCTTAGTATTTTGGTCCCGTGTATATATGGGTGTTAAAAGAGTGACTATATACATGCATGTCTATAAAAGCATACATAGTGAAAGGTTTTTTTTTTCTTGTTCGATGTACGATATTCATATATTTGGATTCGTACAAAAGGATTTCATATTAGGGGTAAAACACTTCATAACAGAAGTAACTTCATATTTATAGAAATTCAAATACGAAATTTTTGATTAAGGATGAAAGAATACTTACGACTATATCGCCTTTATTATATAGTGAAAATTATTTTAAAGTAAGATGGTCCATAACCTTTTTTGAACTACTAACAAATAATAATTTATGACAAGTTCTATTCATATTTCATCCTAAAATCTTTTGCCTTTCTCATGAATTTTGAGTTGAATTAATATTCACTTTTGACTTTTGCTCATACTCAAATAACTCGTGGCTTGAATAAAGCTTTGAATTAGATGATTTGTTAAGGGTGTGTTTGGAAGAATTTTTTTTCATGTTCAATTAGTTAAAATTTTCGAAATATAATTCTTTTAAAAATTCTTTTTTCTAATAAAAGTTGGAATGACATTCTATGTGAAATATGTTTTCGCCGTCCTCCGATGCATCCTATCTTCATCCTTCAATTACAGTCTAAGAAAATTGTAATCCGCGGCTACAACTTTTTGTCTGAATTTTATATTCAGAATTTGTATAATTTAGTTCCTGGTTGTATAAATTTAGAATTTTGTATATGTTTGTCCTATCTATCTATCTATCTATCTATCTATCTATCTATCTATATACGATTAATAAAAAAATCACACTCATAAATTACCATGTTTGTATATTGACAAGAGAATATATACAAAGAAAGTTTTAAAATTACTTAATATAAAATTTATATATACTTACCTTATGTATAGATCCGCAAGCGAAATATACAATAGATAAAAATATACAAATCGTAGCCTCCGTAACAAATAAATTTAACACATAAAATTATCAAAATTATAGTTATAGAGGTTATTATATCTATTATATTAACTATTCCCGCATTCTCGTAATACATCCATCTCTCAAAAAATATTAATTAAAAGGAATGTTTAGCTATATTACATTTATTTTTATCTTTAAGATAGTAATTTATATTTATTATATTTATATACACTACAATTGAAATGTTTATATATTTATATTTATATTTATGAGAGGTGACCCGACTTCCTTCCTACTTGTGATAAAAGATAAAAGAAAACAAATAAAAGTATTTTGTCTTTTAACCATTTGTAAACACCAACAAATTAATTCCTCTTTCTCCAAAGTCATAGGACACAATTTGCATCTATTTCCATGCACCCCACGAGCTACATCACACTCCCATCCTTCTTAACTCATTTACGAGTCTGTTTCATTTTAATTTATGTGATATTATTTAAAAAATGATATAAAATTAAAATAGATAACGTATTATACTTTTGACAAAAAGCGCTAAAGTATAATGTATGGCGATGATTATGTCACTAGATATTTATACTTATTGTATAATTTAACATGTTTGAGTAAGTTTATAGGAGATTAGGAGAAATGGTTTTATATCTTTACGAGATAAGAATGAGTATACAATACATGATACAAAAGTATACCAAGGACAAAACACGATCGAATACATTAATATAAATCTATTGCTAGTTGCATATATACTCAAATTCCTTGATACTGGGTGAATATACCAGGAACAAATATACTTGACACAATTATATTTGTTGGTACATATAATAAATTCGTGAAACAACCCAATATAACAAATATAATATGTATTCATTTTCTCTCTCCTAAATTTCACTCGCCCCTTTCATTCATTTTTTAATTCTGTTTGTCTCTCTCCTCCTTCTAACATATAATTATAAACTGTAATGAAGTAAACTATAACTATAGATATCTAATTAAGTTCAAACTATCGTCATTTTTTTTTATAAAATTAGCTATCCCAATATAATACAAATAGTGAGATAAATATTCTTGAAATTAACTAATACCCCTAACATAGATTTTATAGAAAATTATAAATTGAGTTTCTTTGGTCCTAAAACTTAAATACTAGAATAGAGAAAATCTCATTTATCCAATCATAGCAAAAACTTATCCATATAAAAGTGATTAAGCCAAATCATGTCATATGCTACACACTATGGATAACTCTTCCTAAAATTAACCCAAAAACAAAAAGTTGCTTTCTCTTTTTTCCAGCTAATCACACAAATAAAAAAAAACAGTGTCCAAACCGTATATACCCAATGCCATAGACCCCACCAACCAGACTTTTTTTCTTTTTTTGCCGTGACCTTCGGATCCGACTAGCTGTATTCATCGGATCTTACTGTTTCAAGGTGAGTGTACCACGATTAATTTCACGGGGCTCTGGAGATACGGTCGCTTTGTAATGCCTCCAGTGGCCCTGCTGGGGCTCGAAATAGCGACCTGAAGGACTTTGTCCTCAGGTCTTTACCAGCCGAGCTACCCCTCATGGTTACCCACCAGACTTCTTTATTCTTAATCAAAGATTTTAGGTATGACTTTTTCACAAGTGTAAATTATTTCGTAGAGAATGCTTTACTCTCGATATAAAACTTTTCGATAAAATTTAAATTTAATCGAATTTCAATATGATACTGAACACCAAATTAAAAGCCGAAAAATGTAAAATCATAAACACCACCTCTCCCCCTCAATATGTGCAGATATATAATACTACTACTCATCTATAGAGAACTTCAAACAAAAAGGTACTCCTTTATAGTGACGGATCTAAAATTTTCATAGGGGTTCGAAAAGAAAAAAAAAGTAATACTTAAAATTTGAGATTTTGAAACCTCAAAGTTAATTTTGAACCCCTCAACTACTAAATCAATCTCTGATCTTACATTTAAGAGGTTCAAAATTAATATACAGACATAAAAATTCTTTAAAATACATTAAATATACAACATAATTGTTCACCGAGAGAGTTCTCTAGGTTCGCCCCTACTCTTTCATTCTTAATTAGAAGTCTTAAGTTCAAGTTCTTACGAGTACAAATGACCCTTATTAAACAATATCTTATCCTTAATATAAAACTTTTTTACACGAATTCAAATTTAATCGAACTCCATTACAAATATCAAAATAAATGAAAAGCAAAAAAAATGGCATAACCACCCTCCACCCCTACCCCACCCCACCCCCACCTCCTCATATATATACATGTGTGTGCATATATAATACACTACTACTCCATATATAGACACACAAACACCTTCACTAGAAAAAATTCAGATTCACAGAGACATAAACACAAACACCTTCACTGCAATTCTCAAAATACTGCAAAATGACGAAACAAGATGAAGGATTAACATTAGAACTCATACGACAACATCTCCTCGAAGATTTCACAACTACAGAATCATTCATCGACAGTCTCAATTCTTGTTTTTCCGATCACATCTCCTCCTCCGATGACATCTCCCCTGTTTTCACTTCAGTAAAAACAGAGCCATCTACATCCAATTCCCTCTCAGATTCACCCAATTCCTCATACCCAAATGAACCCAACTCCCCAATTTCCCGTTACTTCAATCTCCGCTCCGATTTCCCTGAATTCAAAATCGATTCAGATACCATCCTCAGTCCAGTTTTCGACAGCTCCGCCGGTTCTAATGAAGACAATAATAAGAAGAAGAATTACAGAGGGGTAAGGAGAAGGCCATGGGGGAAATTTGCGGCGGAGATAAGAGATCCAAGTCGAAAAGGATCGAGGATTTGGTTGGGTACTTTTGATACTGATATTGATGCTGCTAGAGCTTATGATTGTGCAGCGTTTAAGATGAGAGGAAGAAAAGCTATTCTGAATTTTCCGTTGGATGCCGGAAAATCTGGTGCTCCGGCGAATGTTGGCCGGAAAAGGAGAAGAGAGAACAAGATGGAGTTGGTGTAG

>SlERF11-2

TCAGAAACACAAGAATCTATAGAAGTTGTTTCCATGATCAATTTATGAATAGCAGAAGTGTTATTTCTAATTAATCTCTCCCTTATTTTTTTAAAGTTTATCTACTAAAATAATTAGGCCTCCATATACAACAGTGTCACAGTGTTGTGCAAACTAGCACATCCAAGAAAGTATAATTTAGCTGAATAAGAATGGCATCTGTGATGAGTAACGGAACGAAGTCATCTGCTTTATCATAGACTTGAGGCATTAAATTTTGTAGGATGCCCTTAATTCCAAGTATGAGAAGCAATGAGGCTTAATATATACTTGTATAAACTTTTGGATTCATAAACTTATATATACACAGTGCAAAAAGTTTTCCTTTTTCTGTGCATTTCTCAAGATTATGTGTTACTTGTTCATTCTTATATCATCATCTAGAGTTAAGTATGTCTTTAGTTGTTCTAATATCATACTTGAGTCTTCATTTTAGATTTCAAGACTTGTTTTATCACCTAATTGCTTCTGAAGATACCACAAAATGTTCACACAGGATGCTAACTTAGAAGACAAGCCCTGCAATACTTCAGATGCCGTTGTCTCTCATCTCTGCCACCCTAATGCAGATACTTCAGCAAAGCCTGATTAGAACATACTCATGCATATACATAGAAGGAGGCATAGTTTAGTGAAATGCTTGCAATAATAAAAGTGGTACCAATTCAGGATTAGATTTTCAAGTCTTCTCATAACCAAATGTAAAAGTGAAAACAGTGGATATATTCAATTTTCTCTGATAAAATAATATTCAATCTGTACGATGTGCTTTACAATTGTGTTCTTCATTCTATGCCCTATCTAAACTAGAATGTAATTAAAAAAAATGTTGATTATGACTTAACAAACAAGAAGCTGTAGGTTTCCACTTTCTCGGTGCAAAGAAGATGCCGAACAGCAACTAGAAGGAATTGATAGGAGCAATGCTTTGTGAAGATCTCCTGCAGTTACGCCTCTGCTTATTAACGAAATGATATAGTCCATGAAGGCTGAATCACAAGGTAATGTAATAGGACCACCACAAGGAAGCCCAAATTCTTCTTCAGACATGTTCAAGAGTTGCCTAATGACTTCATTTTCAAGGTAAGCCAATGGAATGACAAAGCGTGCTTGATCAGCTGTATATACTACAAAATGTCCTTTTTCAACTATAGACGATGAAGACGTACTACAACTGTCTGCATCACTACCATTTCTTGGAAATGAAATCCTTTTCCTCTGCATGGCTGCAAACTTCTGCCATTTCTTAGCCATTTTGATGAGTTTTTTAGTACTAATCATTGCCATTTTCTTTTCCAGAAGATTGAAAACAAGAAACTAAATAACAAATTTAGAGACGTTTGATGGCTGAATTGATAATGCTTGTGCTTGATGATGAAAGGCTCATGAAAGAGGGCTTATTTATACAAGTTGAGCTGATAAGATTGTTAGGCAAAGCCATATGCTGATGCCTGTTGGTCATGTTCATATGGGGCCTTCAAAATTTGTGGACAAAAGAAGGTAAGTTTCTTCCTTTCTTCTCTTTAATCAACAAACGATTAGCTATGACATCAGCTCGTATTTGCTACTTCCCACCAAACGCCAATAATTTGAAACCATTAAATGAACTAGTACTGGTATGAAACAGAATCATGTTCTGCTCTCTTCTTTGTCATGTAGATAGGTGACCTTATCAAAACCATGATGCATTTTTTTTTCTCCGTTTTTCGCAGGATAACAACAAATAGGGCTATATAAACATATATGTGGTATTGTTTTCCACCAATATTATAAGTATCTATCAAAGATCTTATAAGGCAATACCATGTGATACATCATATGGTATTGTCTTTTTTTTCACTTGTGCTCAACTTGAATGAAGTTGACAAACTTCACTAACATTGTTTCGTATTCACTTGACAGTGAAATCTTCTGAGATCATTTGGTAGAAGGCACCAGTACTGTTGTATCAAACAATATGGTCAAAATTTATAAATACTTGACCATTCATTCTATGACTTATCAGCTATCGAGTTCATTATGGAGAGGAACTCTTCATTTATCTCATTTCGCGCTTCATTAGGACACCAGAGGTGAAACAAATCTCTGTTTTGGGCAGCTCTACTTTGTAAAACAACGTGAATTTCATACAGAGCTGATGGAACGTTGTCTGAAATCGGAGTTCCTGAGGGAATGTGCGTTGCAAACAGTTGTAACGAGTACAATTTTGACGTAGTTCCAATGAAAACGCCATCAAAATAGTACAATTTCGATGGATTTTCAATGAAAACGCCATCAAAATAGTACAATTTTGACGTATATCCAATGAAAATGCCATCAAAATAGTACAATTTTGACGTATATCCAATGAAAACGCCATCAAAATAGTACAATTTTGATGGATTTTCAATGAAAGAACCATAAAAAATTCGCGTATTTCTGGTAGTGCAAGTTATCACATCAACAAATGCAGAACCAAGATATAAGTTATTGCAAACATTTAGTCAATACATCGTCCCATTTCATCGATAGATAAGTATATAGAAGTAAAATGGAAGGCATATTATTTTCATCAAAATCTACGTATTTTCGTTGAATAATAGTGTATTATTCGACGGAGATCAAACTTCTAATAAGAACATGTTCTCTTTACTTGATTTTGTACATTGTGAATTTGAATTAACAAGTTGTAAGATGAAGAAATTTCAGATAGAGAGCACGTCATTCGCGTTAATAAGTTATGTTACGTAAATTTGAATAAATGAATTATATAAAAAGATAAAAAAAAACAGAATTATACAAAGGGGATGCATTTTATTTTTTCAATTATTACCTACTATTTATGTAAACTATTAGATATAAAATTTGGACCACTTTGACATGATGTATGCTATTTCTTTGAATTGAAATCATTCCTAAAACCTAGCTACATGCTACAAAAAAAAAATGGACCACTTTGACCAGATTTATGCCATTATTTACCTATTTTAATTGCAATCAAAAAAATGACGGCGTTTGAAATTCAATATTTTTTTTACTGTTATAAAAAATAAATAATATAACATAGCATAAAAGATCGATTTCACATAAATAAGATTGTTACTGTGAAATATTGTTACAAAAAAGAGATTGTTATAGATTGCTTATTTTTTCTGAATACTAATAATAGTAATTGTTAAATGAGGAAGATAATTATAAGTAATATAATTAAACAAATTTTAGATTTATTATTATTCTTCATTTCCTGCACACAAGCTAAGTCTTAAAAGTCAATATAATTCGTATGTGATAGTAATAAAAAATTTAGCAATGCTAATAAGAATTCTCTTAAATTCAAAATTATATTGAAATTGAAACTTCTTATTGGACCATTTATAGCTAGTTAATTAAATGATTGACTTTATAGACATGTTTATTCATTCTAGTGACATCATTATAAGTATTTATAGATAAATAATGTTTCTTGGATATTATATATATTTAATTAGTACTTATTCACAATTATTAGTATAAAAGTAATAATTTTAGGATTACTTAATAATATCACATACCCAAGAATCTCGTAACATTCACAAAAATTAAAAATTCTTTCCAAAGACTCTACTAATTAAGGTTTAGTGGAATGATAGGTTTTTGTATTTTTAATTTTTAATCAGACGTCTTAAATTTAATTTGGTAAATGAAATTTAAAGGTTAAAATTAGGAAAAATTATAAAATATGGCAAACATTAATATACATTTAAAAGAAATAGCTATAATTCATTTTTTTATGTTATATGGACATAATTACACAATAAATAGCAAAGTTAATAATATATAAAATAATTACACACCTAATTTATTATGTCTAAGCATGTTTTTATGTGATTGGTTTCCCATGTTTACCCTCACTAATACTTTTTCTTTCATCAATTTTTCTATCTATATTACAAAATTTATGTATTTTTCTCTCAAATTTCTTTCAATTTAAGATCTTTTTGATGACTTTAGGAGCAATTTTTGTAATTTGTATTCATCAGATATTATTTGATTGATTTTCTCCTCATTTTTATGAAGATCATAGTTATATACATATATGTAGCATCTATTTTTATAGGCTTTCGATTGACTTGTTTCTACATTACAATTACTATAGTTATAAAATAAAAATAAAAATGCACAATACCCCTCAACCTATGCCTGAAATCTCAGAGATACACTTATACTATACAAAAGTTCTATTACCCCCTGAACTTATTTTATAAGTAATTTTCTAGCCATTTTCGATCTACGACACTAGCTTGGAAAAAAAAGTCAACCAGCGTTGGGCCCACAAGATAGTGGAGAAATTATTACTCAAATATATGTTTTATGTTTTCCTTATTTTCCATATTCCCTTTGATTTTTAAAAATCTCTAAAATCCTCTTATTTCTCTCCTAACGTATTTAATATATTCGAGCTACATATTATGTATCTGATTTATTTTATGATGTATTGGAGCTAGTTTTTAATGTATCGAACTCTAATAAACCTCACATAATAGACGTTTTACTATACAAATTCAGATTAATCAATCTAGTAACCAATTCAATAAATACATCATACAGTAAAATCTATTTATAACAATTGATCTCTGCTAAATAAAAATCTCATATAACGAATTTAAATTTCTTTGAATCAATTAGTATAATTTTTTTTATAACAACGTATTGTAGCCCAGTTAAAAATATTTGAATAACTAATGTAAAAATTTAATAAATGAAAAACTTATTAAATAAATCACATCAATCAAATCTAATGCCATATATTGGCATCCTAGTTGTCAAAGAATCTTGCAACTTTTTTTTTTCCATTTTCTATGATCCAAAATGGCCCTTCCATTTTCCATTTCTTCATCACAAAAACATCTATTAAAGCTATAATTTGTTAAGATCTTATCATTTCTACAATTTTTTTTCTTCATAATCATAAAAAATAAAATAAAAACTCATCCAAATCAAAGATGAATCATTCTTTTTCAATAGAAACAAGTTGTTATGAAGAGATCAAAATCAAAGATGAAGAAGAGATTATTATAAATTTCGATAATAATATTCAAGATGAAGAAAATACACAAAAGGGTAAACAATACATTGGAGTTCGAAAACGTCCATGGGGAAAATATGCAGCTGAAATTAGGGATTCGACGCGAAATGGAATTAGGGTTTGGCTAGGTACGTTTGATACTGCTGAAGAAGCTGCTTTAGCTTACGACCAAGCTGCACTCTCAATGCGTGGTCCTACAACATGTCTTAATTTTGGAGTCGAGATAGTTCAAGAATCGTTAAGAGGATTTAAATATACGAGTCAAGAGGGTTCAACATCGTCTCCTGCTCAAGTTATAAAGGAAAAACACAAGAAGAGAAATAGTAAAGGTAAAAAAACAATAAAATCGAATAACCAAAAAAATAATAATAATGTATTGGTCCTTGAAGATTTAGGTACTGATTTATTAGATCAACTTTTATCTACTAAGTGTTCTACTTCTGAATAA

>SlERF4-3

GGAAAAAATTGTAGTGAACTAAAAAGTGTATTGTTAAAAACTAGGGGCATTATGGTAATTTCGCATGTAACATAAGCATTTGTTTATCTTGTGCATCTTTTTGGCCAAGCATTAAAACCTGCTTGGCAGCGGTGATGGTTATTTTTTCTTTCAGTATAATTACTATAGAAAATCATGAATGTAAAATAATATTTCCCATTTTTTTAATATTTGTGTTGGATTCTACTTCTCTCTCTTGTATTTTGAGATGGGTCATGGAGGCCCAAACCTAATTTTACCTCGAGTAAAGTTGAAAGGAACGCTAAAATACAGGTTTTGAGGAGGTGTTGATACATGCTGATACACAACTGATATACATGTGTAAAAGGGATTGGTAACAAGAGCCCACACTAGTGAAAAGTGCCCAAATTCATCATCTCTTTGCTTTTTTAATTATTTTGATTGTAAATGTTTCATTAATAGGAAAAATTGTATACAACGGCAAACTAATAAATCAAATTAAATGTTATAACTACACTTTGATATAATTGTGCAATGTAACAAACTGTTTATTAGTTGCAAATACATATATCTTCGTCCTATACACTTATAATTATACAATACAAATATTCTCTTGCCCAATTTTCTTTTATCTTGCTCTATTTCTCGTTTTATACATACACAAATTATACAATTGCTTTGTGTACAACTATTTTCTTTTGTATATGTATAATGAATTATACAATTGATTTCTTTGTATATGTATATCGAATTATACAATTGTGTTTTCTTTTGTATATGTGTAGCGAAATATACATCTTTATGTTTGCTATGGAGCACAATTATGCAAACTTTACTATAACATACAAATATAAATTTTGTGTTTGCTATAAGTGAAAGTTATTCTGAATAATATTAGTTAACTCTTTAACTTATTTAATTAGTTTAATTAAACTTTCCAAAAGATGAGCTATGTTTTAACTGGCTTAATTTTTTAAAAAATCATTTTCATCAAGTCATTTGTTTTTTTTATTTATCTAACAAAATATGTTGACCGAATTACTTATCTTTAATTGTTTAATTTTGTTCTAGTTAGAACTCCTCTTAAAAAATGAATAATCTCAATTTTTCTTTTACTCAACCATATTCTACTCTCTTTTATAAATACTACAATTGTTTCATGTTAACTTTTTCCTCAAATGACCTTTAAAAATGCTTTTTTTACTTAATTATTTTTTCGCAAATGTTAATCAAATATTTAGTGAAATCATCAGATAATCGTATGATAGCAGCATATCTAACTCTTTAAGTGCTACCTTTTTAAAGTAGAACTAAAAACCTCTTATCCATTTTCTCTAGATTTTTAAAGATTTATTTATTTAGACCTTCGTAAATTAAGTTTATAATAATTAAAAATTAACTGAGTATTCTTTTATTAAGTATTTTTAAAAATATATATATATGCTTAACTTTTTGAAAATAATTTTTCTAAAAATAGTAAAATCACACATATTAATATTCAAATTTATTTGCATATCTAATTTTTTTAGACTAAATAAAAGAGAACTAAATAGAAAGAATTTTACCTACCCTTTTTTCTTGAGCTAAAGTGTTTGCGTATCTAATTTAGACCAGAAAAAATCATTCGTATCTGAATGATTATGATGTCTATAACTTTACCAGACTAATCACTATTTAATTCAATTTAATTAGTAATTAAGTTCATTTTAAAGTCAACAAATTAGTGCCTTCTATAGAATTACAAAGTTATTCTTGCACGTGCTCCACCTCAGCTCACCTAATGATGTTGCTCTATTTCCTCTAATTTTGAATTATAGAGGTGTTAAAAGTAACAAAATATAAACTGATTATGGTGAAGCGATTTAATGATGTGAGAACATATAAAATAAGTAATCATAGTTAAATGAAAAAGAAAGTATATATATGTAAGAAATAATTTCTTATGATAGGTGGTAAATTTAAAAAGTCCCTTAAATATAGAAATAGTTTGGGTCCTTAAAGTTAATTAAAATTGAGTGTTTTTTTTTTTGTCTCCTTTAATATTTATCAAATTTTGCCTGTTTGCCTGTTAGATTTAACAAAAACTTATATGTACTTATTGAGTTTTTTTGTCATTTTTTTTCTTTTTAGATTCACGCTAAATAATTAAATTCAACTGGCAATGGCCAGTTAGATATTTAGCGAAAACTAAAAATATATAGTTTTGACAAATTTAAAAGATCAAAATTGCTCAAAGATAAATTTAAGAAATAATTTGAAATTTGTCCTCCAATATAAAGAACCTTTTTTATTTTTATATTATTATTGTCGATTCACTTTTTTTAGCAATTTAAGAAAGAACAGAGTGCATTTACGTAAACACTAACATATTCTTTCGACTTACGCATTCCAGGTCTTATTTCTTTTTTATCGCACTCAAAATTGTCAAATAATGGGATTTGAATTTGCATCAATCAACTATAATATAAATCCTAAATTCTACAAAAAAAACTCACTCCGGGGAAGTTGGGGGGCATTTGTGAAATTAGGTCTTAGGCGTAACTCACACCCCAAAAGCTAGCTCAAAGGGAGGAGGATTGTCTAAGACTTATTAAGGAGTCCACCCACCTCATTAACCACCGATGTTGGACTTTTGTCATTCTTTAACACCCCTCCTCACGCTCAGTGCTAACATCTGGTGCGTGGACAATTTTGATTCTGGGGGTCCCAACATCGGGTGATACGGGTCCTGCTCTAATAACATGTAAAATTAGGTCTTAAGCTTAACTCACACCCCAAAAGCTAGCTCAAAAGGAGGTGGATTGCCTAAGACTTATTAAGGAGTCCACATATCTCATTAATCATTGATGTAGAACTTTTGTTATTCTTTAACAGTATTCAAAAAGAGGATCCACTTTCTTTCCTCGTAGAAAAAGTCTTGATTTTATATGGCACTAGAGAAAGTTTGTTATGATTATTCAATTATATAGAAAAGGTGTGTGTGGCTTCAAACCGCTTTCTTTATGAATAGTTTCAATTAAAATTTCATCAACTGTAATTAGTGGTTATTTGGATCGATAAGTAAAGATAAAGGTAGACCTATTAATTAATCATCGTATTTTAAATCGTGTGTCATTTATCTTTAAGAAATTATTCGGTTCATAAAAAATATTTTTTAAAAAAATGATGAGAAGTTAAGATATGATAATTCAAGCACAAAAGTAGAAGAAAAATTAAAAGTTAATGATGTTCTTTCAATGATAAATTTCTTAACCTTTTTTTAGCTTGGTTATATTAAGTATTTTGATTGGAACTCAATTAAATTTAAATTTACGTACATAAATTTAACCAATATTCAAATTTAAAAAAAATTTAATTAAAAATAAAAAATACTTATCGCTAACACTCAATATCATTCTACTGCAATTCTTATTGGTAATTTTCTAAACCCTGCCATGACAAGTATGTTTATTGAAATTGAAAAAGAAAAGGACAATAATTTATTCATTATTGATCGACTAGACATATGAAAATGATATATAAGCATTTAACGATGGAGAACAACTAATATGTCAAAGTCATTACAATTTACAAATGCTTCAATTATCACGTAATCAAATTACAATAGGATAAAAGAAAACTCTTAAAGAAATATTATATATAAATAAAAAACGAACTCAAAATTTAAAATTTTATAGGATTTAATTTTAATAAAGCAATATAAGATGTTAATAATATATTGAATTTGAAGTTAAAGTTTTATATGTATAATAATATTCTTAATATAAATACATCACTTAAATTAAAATTATATTATTCACTTCGTCGGTAACCTTTTAATTTATATTAGTCTCTTTTTTCTTTTTTTTTTTGCAAGGTGGCAGTTGCTTAATTCTTTGTAATAATAAATGGGAAAACACTCTCGATTTGCTTCAAATATCTTTCAATCGAATGAAAGAGACCATTTTGTTTTGGCATATCTTTTTTTAAAAAAAATTGAACAAATTTCATATATAAATGAAGCGTAAAAACTTATTAACACCTAATATTTTTGTCAAAATATATTAATTTATGATAATTAAATATAACACATTGAAAATATATACACATAATATAAAAATAATAATATAATAAAAAATATATATTATATATTAATTGATTCGATATAAATATCGGTAAGTGGAATAATCAAAGCTTCCATTCTAGATATTTCATGAGTTTTTCATCTTAAAATATGAACATTTAAATGTTGCCACTTGGACAATATGCCAATTTAGTACAATCATGACACTAAATTCTTGACAATTAGGTGTATTGCTTCTCTTTGGGTTTACATCTGCCCCCTACCCTATACTTTTTTTTAGAATTTAAAATGTAATGGAGTAATATATATTGACTATCATAAAATATTTAAGCATTACTCATAGTTAGTTATAAGTGAATTTCTTGATTTTACACAAATAAGTTATATGTTAAAAACTAAAATTAAGTCTAAAAGAATACTTATAATTGAGTCGAGGATTTGTTATATCTTTTTCACTATTTTTATGAAATAGAAATAAAAGTTATATATTCATCGGTCTTTCGAAATCCTTCATAAGTTATTATTATTATTATTTGTTTTAGAAGAACATTTACGTTATCGACATGTAGAAGTTCAAATTTTAATTTTTTTTATGCTTTTGCAAAAGGATGAATATGACACTTCTTTCTAATCCAATTTATTATAATTATTGATTCTTTATGAGGAAGACTTTGAGGTGTTTTGGATCAAGTCAAGTCAAGATAGGAAGTGGGAAAAGTATTTGAACAAATATTGAAATTGTCGTGTCCACATATAATAATGGTCCATCATCAATAAATAAATTTCATAACTTGTTACAACAATTAACATATTTTTATGATCTCCCCTTCCTCATCCAATGTACCTTCTATATAAACCTTAGTTATCCAAGATTATTTTTTTTAGACTCTTATTAACATCCCAAACACCAATAATATCCAATTTCTTATAATATACAATGGAATCTTCATCCCCTAAAACTCAATATCCAAATTTCAATTTCTTCCAAGATCAATCATCATTACCATGGAATGATCAACATTTCTTAGATGAATATTTGACTAACATCGACCAAAACAACGATCATTCTCTACCAGGAAGTACTTGTTCATTCTTAACCTCGAAAGAAAGTTATAGACGGGAAGTTTCCTCCTCCAACCTACATCAATTACCAAGAAGTTGGTCATCCTCAAACGATACGAATTCCTCTAAAGAAAGCAATAATCGTCATGAAATCGAAGAGGTCACGTCTCATCACCATGATAAGAATAACTCCACCAAACACTACATAGGAGTTAGAAAGAGACCATGGGGAAAATATGCAGCGGAAATAAGGGATTCAACAAGAAATGGGATTAGGGTTTGGTTAGGAACATTTAATACTTGTGAAGAAGCTGCTTTAGCGTATGATCAAGCTGCACTTACAATGAGAGGTCCATTGGCACTTTTAAATTTTCCAATGGACAAAGTAAGAGAATCACTTGAAAATATTAAGTATATTTGTGAAGATGGGATTTCACCAGCTGCTGTTTTAAAGGCAACAAATAAAATGAGACGTGTTAAACATAAGAGAAATAGGAAGAAGAGAAATGTTTTGGTTTTTGAAGATTTGGGTGCTGAATTGTTAGAGGAACTTTTGACGAGTACTTCCTCTAATTAG

>SlERF2-4

GCATGTGCTTCGTGTAAGCATCAAAGAAAAAAGTGTATGTCAGGGGACTGTGTTATGTGGCGCCACTTTCCAGCTAGCAAGATGGATGAATTTCTTGGAGTTCACAAGGTTTTTGGTATATCTAATGTTACAAAAAAGATTAAGAGTTTTGATGATGTTGCTCAACAGGATGAATCCATCAAGTCCTTCTTGTGGGAAGCAAAGTTGTGGCAGGTAACTCTACATGCACATTCTGTTAGGTTGTGGAGCCTGATTTATTAATATATATACCCTATCGTCGTGGAAGTTTACTTTCGGAGTGTTACGAAATATTGGTTTCTCTCTTTCTCTCTCTAGATCTTTCATCTCTTTCTCTTGAAAGTTGTTGTGTTCGTGTGTGTGAATTCGATCCTAACATGATTTGAAGTTTACCTTTAATTAAGGTTATGTTATCTATTATTCATCAGAAGACTCAACTTTAACTTGATGATGAATTCAATTCTGAAGTTTTTATTTTGAATTTGAAATTTAGTGTTTTGTTGAAACTTTAGAGGGTTTTTATTATATCTATATATCCAGGTTTAGTGGATTTATATATCACATGTTCACCTAGAATAAAATAATATCTCCAGCTAGAAAACCTCAATATATAAAATAATGTCTCCCGCTAGAAAACCTCAATGTATTAGATGGAGAATTTGTGAGACAGCACCTTCAGATTGAACAGAATTTGTGAGACATAACTATTATGTTAACGCCAAAATAATTTTTAGCGACAATAAATAGATACATTAATACAAAAATACATATTTAATTGTAAATTGTTAATTAATTGCAGCTTCTCGAATGTTTGTCTTTTGTATATTTTTCGAGGTTTGAGCTTCCAAATGACACCTGTGACACCTCTCCATGACAGGAAGATCCAGTTCACGGGCCATTAGGGGAATACAAGAAGTTGGAAAAACAACTTATGAAGGAACAAAGGAACAAACAACTTCAAATAGTTCAATTCCCTCGTGTACCCCAGGTTCCAATAACAACAACATCAGAGAGTACAACTAGCTACGTCCCCCATTTGGCAATAGATCAACATCATAGAGAAAATGGTGCAAATCATAGTCAACAAGACTATAACATGCTGAACTCAAGTGTACCGAATTATGCTAATCATTTCCAATGGCATGGACCACAAGGAGTAGAAACTCAAGGAATTACTAGTAATGGTTCTAATTTGATGAATATTGGTTTTGGCAACGATATGTTTGGATATGGTAATTTACCATATCGACCAATCGAACAAATTCGAAATGGTTATATCCCAATGGTTCATCGTCAAGCTACTTACAATAACGGTGGACATGTTCAATTACATGAACAAAGATTTCCATACAACAATGTTGATTTGGGCCTTGAAAGAAGAAGAAAGGACCAAACTGATCAATTTGTGCATCCAAATTTTAATGGTAGACATGTACGAGGAAGGGGAATTGGGTCAATTGGCCCAATGATTAGACATAGCCCAATTATATCAAATTGTAGTTCTGGTTCTAGTGTTGACTCGATTATTGTTAATGCTACGGGGACAATGGCGAATCATAGTGTTAATGGAAGACATGTCGAAGATGGAAGAAATCAAAGAGCACAAGGTGTTGCAACAGTTCTTCAGGACTTCACTAACTATAACCACAATGATTTACCAGGTAAATCTTTTATAAGTTTCGAGTTATATAGACCATCAAATTATTTTTGCCTGGTCCAATCGATTTTAGATTGGATTGACTCTTGAGCCGATTATTGTCTTTAACTCGTCCAAATTTGATCCTAATTGCCACTTCTAGTAGTAGGTAACATACTTATTTTAAAATTTTAGATTGTACATTTAATTTAAATATAGTTTACCTCTACGTATATTTTGATGACCTGAATGGTGTATAAAAATTTACTCTTTTTTTTCTCAAGTTTTCATTATATATATTTTGATGACCTGATGGTGTATAAAAATTTACTCTTTTTTTTCTGAATTTTTCATCGCCGAATAATACAAGTGCTAATATCAAATTACTAGATTTGAAATTTTCAAGATTATGATGCCTCTAATGTTTTAAACCATAACGTATATCTAATAATCATTTTTTTTGTTCTCTATATTTGAAGGTGAACATAAGCAACGAGATATGGTTAATAGAGATCATTCAACCTCCAAATAGCACATTTTTCACATTATTTCTAAGTTTGTTGCCATATGATATAAGGGGGAAAAGGTGGTAATTACAGATTCCTCCTCCATCATTCATTTGTAAGTTCTCCTAAAAGTCCTATCTCATATTATATTATCCTAAATTTTATCTTTTTCAATATATTTTATTTTCAGGTAAATAAGAATTTTATTTTCAAAGAAAAGTGTATCGTTCAAAAAAAAATAGAAAGAAATTATTCTGACTAGATCAAATATTAGTTAATTTCATTTTGAGACAGCCTTTGAAATATAGATATTAAATTATTCTCTTCATTAACATATCGATACTTTGTTGAAAAACAATTTTATTCCTCTCTTATCATATATGATAATGCAGCCGGTTAAGGATATATTGTTGTTATAGCACTATCTTGCTCAGAACTGTGTATACCAAGTCAAATAATTTTCAAATGTACATGTCTCTTTTTTAAAAGTTAAATATATTTGCACCTACTTTTTGCACAAATAAATGTGTGCACATTAGTTTGGCGACCATGTGTGATGTGTCGTGCCTGTGGGGTTCATAGTTTGGCGCAGTAGTTTGTCGCAGCCAAAGACAGTTTAAAGGAACAACTTCATATATAGCAAATAAAAAATATATATTTGTATGTTATAACAAAATTTGTATAATTGCGTTACATAATAAACATATAACTGTATAATTCGCTGGCCTAAATTGTATAATTCGCTGACCTATTTCGCTGCAATTGTATAATTCGCTCGCCTATTTCACTGCAGTTGTATAATGCGCAATTATATAATTCGCTGACCTATTTTGCTGGAATATGTGTATAAAATTTGCTTTGCATACAATTAAATCGAAGTAAAAGGTTTATATATTGTATAATTATAAGTGTATATATAGGAAGAAGATATATGTTTTTCGCTTGCTTTATACAAAAACAGAAACACAATTTATACACTTCTGTTGTATAAAGCGAGATAATTCGTTGGCGTTTTTCGCTGTAATATTTGTATAAAATTTGCATTTGTATACAATTGAATCGAAGTAAAATATTTTTAAATTGTATAATTAAGTGTATAGCACGAAAATATACATTTTTTACATGTGTATATACAATTTTCTCTCGCTTTATACAAAACAAAAATACAATTTATACACTTCTGTTGCATAAAGCGAGAGAGGCAAGCAGATGGAGAGTGGTGAGCGAGAGTTTTGGAAGAGAGGCGACTGGCAAAGACAAAGGTTTGCTATGAAGCACAATTAAATCAAACAGTAACTACTCCATTTATTTTAGGTTACTAATTTGCTACTATATAGAATTATCCCTTAATAAAATTCTTATCATATAGTATAATATATGAATTGAATACCCATCATTTCAAAAAATTAAACTAGTCATTTGAATGTCTATATCATTATAAAACCATGTGAAAAATTAGATTATTCTTCGTGTAAATTACTCGCTCAGTCTTATTTATCTTTTTAGTCTGATTCACATAATTTTTAAAAGTTTTACATAACATGTTTAAAATCATAAAATTAAAGACATTAATATATATTTACACATCTTTAATTTATGACATATATTTTTTTTTAAAAAATCTTTTTTAAATTAATTTTAAATTAAAATAATAAAATGAAACACATAAATAAAGTAATAATAATTATCATCGTATATTGATGAACAAATGAGTTGATGTTTGATCCTATTCTTAGTCAAGCAAATAGAAGTCTAAATTAGCACTTTTTGTTTTTAATTTATTGATGATTCATGAACCTTGTTTGTTAAATTCAAGACTATATATTACAGTCAATTGTAAAACAAAATGTCTTGGAAAATTAGATCCTTTTATTGGCCCACCAAATAGACCTAGGAAACCCATACGAACCCAGTGAAGTCACATCGATGTGGAGTCTCTAGAACGTCAAATGTGAGTCATCTTGCTCATCCTTTTGTCGAAATTTCTTCTATTGGGTCAAATAAGAAATATGTGCCATTCTCGTTCTTATTAAATAGACATAATATACACTTTTAATTAAATTAATATGTGTCATGTAATGATTTGTACGTAAATATGACTGATTTAAAAATAATATAATAATAATTGTTCAAAATTACATAGTGAATTAATAGATAATTACAAAATAAGTTGTCTTTTTAAAAATCTTGGACAACTTCAATGGTGTCTTTACTTCGTTTTCTCTTTATAAATTTAAAGCAAAAAAATATTTATATGATGTCAATTGACCAAACACATGGATGACCTGGATCTAATTTCTTCGAAGAGCATTATCTCATCTTGTTTATTTGGAGTACTATCTTTTCTTATCTCACTGAATGCCTACAAACTAAGCATAATTTTATTCTTCTGAAGATTAGCTTGCTCATAAAACCCTCGTATCATTTTGAGCTATTTGTAATTGAAAACGATGTTTTCATCACTATTATTTATTTTAAAAAGGCGTTTTTTTCGAGCAATAAATTCTCTTTATTTTAATGGTGGTCAAATTATGTTCAATTTTCTTATCGTACATAACAATTAACTAAACTTTGAAATATCAACGCAAACAAGGGGTAATTAATTAAACCATGTCACAACCCATCAAGACACCAACTATATAATATAAATTGAAAAAGACGTGCACAATTTTTCACTTGTTTCCTTATACTTTCCTTTTTCTCCACGAGATAAATAAAATTCATAAAAAGAGCCCCACGATTAAACGATTACTTTTCTAGTATTCCTTTATGTTAAATACCTCATTAAACTATAACATTTTCCCCCAACAAATTAACCAAAATCCCCTCTAATAAAAGCTATGGTTCCAACTCCTCAAAGTGATTTACCTCTTAATGAGAATGACTCACAAGAGATGGTATTATATGAAGTTCTTAATGAAGCTAATGCTCTAAATATTCCTTATTTACCCCAACGAAATCAATTACTCCCTAGAAATAATATTCTTCGTCCATTACAGTGCATAGGCAAGAAATACAGAGGAGTACGACGTCGTCCGTGGGGGAAATACGCTGCGGAAATTCGCGATTCGGCTAGACATGGTGCGAGAGTATGGCTAGGTACGTTCGAAACTGCTGAAGAAGCTGCGTTAGCTTATGATAGAGCGGCTTTTAGAATGCGAGGTGCTAAGGCACTACTTAATTTTCCATCTGAAATAGTGAACGCCTCTGTTTCAGTAGACAAATTAAGTTTGTGCTCAAATAGTTACACTACGAATAATAATTCAGATTCAAGTTTAAATGAAGTTTCAAGTGGAACTAATGATGTATTTGAATCAAGATGTTAA

>SlERF3-12

TGAAGGATCTGTGACGGCCCGTCACGCCTGTGACGGTCCGTCCTGCCATTCCGTTACGAAGTTCAGAGAATCGATTTCAGTATCCAAATTTCAGAAATCTAAGTGTTTTGGAACGAGACCCCCTCGACGGTCCGTCGTGCTCATGACGGTCCGTCGTGGGATCCGTCGTCTCAGCCAGTTTTTCCAGAAATAAAATCTGCTGCTGAAAACGACTAGACAGGTCGTTACAAAAAGGCACCTAAAATTCAGGGGCGGATCAACACAATTCGAGGCCTAAAGCGAAATTTTAATTAGAGGCCTAAATATTTTTAAAAAATTATTTGTATTTATATTACAATTTTTAGATGTAAATTATTACTTAATATCCTTTTTATAAATGATTTTTTTCAAAAACTTTTTTAAGATATTATTTTTAAGAATTTTTTTTATCAATTCTACATTGAAAATATTATTTTATTGAGAAAATTATTATATGAATCGAAAATATAGTTCTATAAGTAATAAGATGATAACTTTTAAATAAAGATAAGAGTTAGAATTATAGAATATGATTCAAAAAGTTGACAACTTGATATTTCTTACTTTAATATAAAAATTACGAAGAAATAAAAGAATATTTGTAATTTACTTTTTTCAACCACTTTTCAGCTGTTGATTCATACTTTTGACAGAATATTACTCTAATCATCAAAGATTTGAAAAAATAGAGTGCATAAGGAGTTAATTTTTTTTAAAAAAATATGAATATTAACAAAAAAATATAATATATTTATACTTTTTATTATGAAGAATAATTTTTTTTTCATAAAAAAATTAAACATGATATTTTATTGTATAGAAATTTGAGGCCCCAAAAAATTGCGGGCTAAGGCAGTGGCCTAAAAAATGATAGGCTAAGAGCCCGCACTGCTAAAATTAATCATGAATAGTTCAAATTTTATAGATTATATAGTTGCCTTTGCATAATATGCACTTATCTGAAGCGTTAAATCATTGCTAGTACTGTTACACAAAAAAAGAAATTGAGTTGTTACACTCATTTCTTTTGATCTTGTAATTTTCAATTAAATTAGTGTTGTAACTTTTGTTCTCCATTACTAACCATGGTCAATTTTAAGACGAAGATTATTTTATTAGTGTATATGAATAATATTAAATATAATGTATTAAAATATTGTCATTAGCTACGTTATATTTAACATTATTTATTATGCGGTGTTTGATTTAATGTATTAAAATAATATATACTTTAAAAATACATAAATCATTATCTATAAAAAAAATACCCTCCAATAAATATAGTGAGAAAATGTAACTCTTTTTAAAAAATAATTATATTTTTAACCAAACTAATGAATGCATCAAAACCTCTTATACTGCTAATAATATATTTTTCATGTATCATTTATGCACAAAATAATATCAACTAGAATTATACATCAATTTGAAAACAAGTACCAAACAATATTGTAATATTATTTTTTCTAATATAATTTACCAAATTGACTCCTTAGTGAGATAAGAATGAGTAGTACGACATCAAATTAAAATACACACTTTTTTTGAACCCATAACACCAAAGAGACACTCTTTAACAACACTCACCAAAACAAACGGCGAAGTATAATTATCATCTTCTAACACCATCATACTTATTAACTCCAAATTTATTCAAAATAATATTGTTAGACAAGCTTTTAAGGTAGTTCACCCACAAATGGATGAACTGCAATTTTTTTTTATTAACTCTTCATGTAAATCTATGTCTCTTTCTCAAGTTCAAGTCTACTGTAAAGTTCCTTTTATACATAAAATCATGATTTTTCTTCACAATAATAAAAGTATTAAAAACTTATGAATTTTTTTAAAGAATAAATTTTACTATTTATTTGCTAACGATAATAGTAAAAAGCTACCGAGGTTGAGGAGACCATGATTCCTCTCATGATCAGTTTCTCGAGATAAACTATACTGTTTCTGTTATGCGACTTGGAGCAATGTCCATTTTATTTCTATGACATTTGGTACTGTTGACTTAAAAAATTTTCAAATATTAAATATTTACAATTAAATTGAATGGATAATGTGACCAATATAACTCATCTTCCAATGAAAAAATGTAGTTTTGCAGTTTCAATTGAGTATTTGTTTTTTTGAAAACATTAAATTTATTTATTTGAATTTTTCGTTAGTTCTAAGACCATATGAAAATTAACGAAATTGTCACATTTTTAAAAAGAATATGTATTGGGATTTGTCCAATTACGGATGAACTCCATGATTAAAAAAATAAATAAATTATTGTAGTTCATAAGCTAAGACACATTTTTGTTATAAATTTTCTTTAGGGTTTGCTACTAATGGGGTGAAATCCAATCTTGTTCTTTTAGAAAAATGTTGTAGTTCGTTTAGTTACGGAGAAACTCTAATATTTTTCTTTAAAAAATTGAACCAACTAAGACAAGGCCGAATGCCTAAGTTTTTTTTATGAGCATGTTTCCTGCCTATCAAAAATAATCACATCCATCATATTTCTAAAATATTTTATTCATTTCATATTAATTTAATTTTTGAGATATTTTTCAATTTTAAAATTAACATAATTGTTTAGTTTTCAAGATTATTTTAAGTGTTTTTTCAATTTTATCTTTTATTAATTAATATTGAAACTACTGATTAATTAATATTTAGTTATTTTAATAAAAAAACTGACAACAGTTAATAAGGGTAAAAATAAAAAATTATGTTCAATTTATGTCTTAATTTTTTTTTTTAAAGAGTGTGAAACACCTCAAAAATTAAATTAATATGGAATGAAGGGAGGAAAAAAACAAGAAAACAAGTGTTTATCCAAATATTTTGAAGTAAATAACTTTTTTTCTTTTTCTTTTTCTACCTTTGAAGGCGCACAACTCCAAAATTTTCGCCAGAAATTTTTTGAAGTCCTCAATATTTTATTTTACAATATTCTTTGCATTTATAAACATCTGGTTCAAATGAATATGCTGACTTTCTAAATTGTTTACACGTGACTATCAAATTTCAAAAATCAAAATATAAAGTATATATAAAAAGTAAAAGATGGATGAGAAAACGTGGAGTGAAGAATTCATATTTATTATTGATGAAAGAAATATAATTTCACATGAACAAGAAAAAGATGTAAATATACGTGGAGTTAATAAAAATAATTGTAATTTGCTAATTTTTGAGCTAATTACTATCTTTAAAGTTTATGTAATACTGTTAAAAGTGAGAGTGAACTATCCGTTCTAATGTGCGTTTTTAAAGAACGTCCTTTTTAATATTTATAGTTCCAAAAATGTTTGTTTTAATATTGGACTCAAAGAATGAGTGTCTCATAACCAATCATTTTCTTGTTTTTGTCTTTTGATAAAATTTAGCCACCACAATGGCTTTATTTTAAAATAAATGTGTCTACAATGAAAAAGGAGGGGAAGCAAATGATAGAAAGCAGACGGTATTCCAAAGAACATCATGATTGTTGATTATTATTTCTTAATTGTTTTACAATATTTTAATGAATATTGTAAAGAGAAGATTTGGAGGATGTTTCTTAGCGACTTCACGCACCAATTAAGTGACCATTAGTTGATTTTATGATTATTTATTAGGGGTCAATAAAGAATAAATTCAGGTGGGTGGTTTACCATCTATTTTTTATTTTATTACGTAGAAAAATTGGTATGGTCAGAAAATTTGGTCAAAATAATATTTTTTTTAATGTTATTTCATCTTTTAAAAATATTTTTATTCTTTTAATTGTAACACCTTTTAATAAAAAAATGAAAACAAAATCAGATTATTTTAGAATAAAAAAATATTATAATTTTTTAAATTTTCTGATCAAATTTTTATAAAATTTTCTGACGATTTCCCTATATATATATGGAAAGCACCTGCCATTATTCTATGATAGTAAAGATAAAAGAGTGAGGCAAATACAATAATGTTAAATGTATATTAAAATACTTATCCTATCTCAATCTGGCCAATAAACACCGCTATAATCTAGCCACCAAAAGGTTTTGTTAAAGTTAAAGGGCCATTTACAAGATATGGATATATCACTAATCACATAAGGCCATTATTAGATAAGCATTTCCAGGAACCTAGCCCCTTATTCTTAGTGCACTTTACATGGCTGTGAATCTAATAACTAAACATGTAGAAATAAATTATCATTTTGTTAGAAAAAAAGAGGTAAGGGAATAACTCATAACAAATTTTGTTTGATCACAAGATCACATAGCAACAATTTGTTGTCTTTCGAGTGGTCTCTGATCACTAGCTTGAGCGGAAGTGTTGGGAGATTAGCGTAAGGAGTAGAATTGTGTTAGCATTAAGAGAGTGCATATATGTGTTGCGCTAGAAGTGTAAAAATTCTTTTTAACTTGTTATTGTTTCATGGGCCAAATACATAAAAATTCTTTTTAATTTAAATGACGATGATGTTTAAACTCAAAAACTCTGATTGTTTGATAGTGTATAATGTAGATGTTAGGTTTTTTGATAGGAAATTTGGTTGTTTGGTGAAAAGAATCTTATAACTCACCCAATCATATGTATTATAAGTTGGAAAAGGATGAAAAATTATTTTCTGATTATGGAGGAAAAAACTACACATCATTAGGTTCATAAAGATCGATTTATCAACTAATGTAAGTTCTATTTGATTTAATTTAATTTTAATCCAAAATCAAAGGACAAAATTTTTTTTTGATGTCGATGAATGAAAGTCTTTTTGTTTTGTTTTTTTTGTGCACGTGATAGAAAAACATGGAAAAATGGCTTTAACAACTAGTCCACTTGCCTAGTGAGAAACTTCAAAGAAGCCTCAAATTCTAAATAATAATTAACTCTTTTTCAGGTGCATGTACCTTGTTGCTCCTATATATACCCCAATTCCTTCTTTTCTTCCAAAATTCACAATTCTTTTCTCTCTTTTACCTTGTATACAAAACCTTCAAATACTCATAATGTATCAACTTCCCACTTCTACTGAGTTAACTTTTTTTCCGGCAGAATTCCCGGTGTATTGCCGGAGTTCAAGTTTCAGTAGTCTCATGCCATGTTTAACCGAATCATGGGGTGACTTGCCGTTAAAAGTTAACGATTCCGAAGATATGGTAATTTATGGGTTTCTACAAGACGCTTTTAGTATCGGATGGACGCCGTCAAATTTAACGTCCGAGGAAGTGAAACTCGAGCCGAGGGAGGAGATTGAGCCAGCTATGAGTACTTCTGTTTCTCCGCCGACAGTGGCTCCAGCGGCTTTGCAGCCTAAAGGAAGGCATTACAGGGGCGTTAGACAAAGGCCATGGGGAAAATTTGCAGCGGAAATAAGAGATCCGGCTAAAAACGGCGCACGGGTTTGGCTTGGAACTTACGAGTCGGCTGAGGAAGCCGCACTCGCTTATGATAAAGCCGCTTTTAGGATGCGCGGTACTAAGGCTCTATTGAATTTCCCGCATAGAATTGGTTTAAATGAGCCGGAGCCGGTTAGAGTGACGGTTAAGAGACGATTATCTGAATCGGCTAGTTCATCGGTATCATCAGCTTCGGAAAGTGGCTCGCCTAAGAGGAGGAGAAAGGGTGTAGCGGCTAAGCAAGCCGAATTAGAAGTTGAGAGCCGGGGACCAAATGTTATGAAAGTTGGTTGCCAAATGGAACAATTTCCAGTTGGCGAGCAGCTATTGGTTAGTTAA

>SlERF5-10

GACCAAATAACATACACAATCCAACCAAACCTACTTATGAGTAGCCCTAATTGTGTGGGTTGCATGATACTAGCACATAGTGGCGGAGCCACATATACTTAAGGGGTACCGATCGATACCTTTTGTTAAAAAATTATATTCTGTAGTTAAGTATGAATATTGTTTTTTATGAATATATATCTCATATATTGACATTTTTTGACTTCTACACAATTTAATTTTTTATGTTTTGACACACTTCAACTAAAACTCTATCAAATTTAAAGTAGTGCATATAGATCATAGTATAATTACCCCTATCGAGTTAAAATTCTAAATCCCGCTTTAATAGTCATTATACCCATGATTAATTTGCAATTTTGTAGTTAATTATTTATTAATGCAGGTTGATTCTTCCTTTTTTGTTAACATGGTGAAAATAAATAAATAGAAGTACCACAGTGTGATATAAATAAATTAATTAGAAGATAAATTTAATATAATTAATAAATTTAACTAATTATTACGTAGTCTAAGATCCTGTTAAAGAAATTTTATTTTCATTTTCAATGGGAATGAGATATGTATATTCAACTCCAATCTTCAAAATCAGGCCACAAGTATTAATATAATATTGTTAATAAACATTATTCTTAATTAATTTTTGCTTAAGATAAACATCAAAGTACATACCAAACTTCTTACCTAAGCATGATGTAATTTTTTGAAGAAAAATAAAGTGACGGCACTAATAGATGTCTAATTGTATTATAAACTATGTTTAACACGCATCTATTTAACCTTATATTATTAATCAAGATAACATATTATTATTATTATTACATGTACGGACAAAGTCAATGGTTTAAAATAAGAAAATACAAAGATAATTAGAGTGAACATTTTAAGAAAGTTATATGAATTTGATTCAAAATTGATAATATCATACCATGAATATTGATTATTATGAACTTGACTTATAACTACGGTCTGGTAAACTAATGATTCATCGAGATGAATATAAAAATGACGAAGTGAGTCCAATTTGATGTTTTTGCTTGAATATGAATCAATTTACTATTTTTATTTATAGCTTTGAAGACATTTACGAAAAAAATGAAGTATGTGAGCCTTGATATGGTGATCATATACACTTGGATGATATGAGTAGCACAAGGTTAATCTCTAAATAAGCTCATAAAGTGATGGATCACGTAAAACTTATTAGTTCATGATGGGCCACGTGGAATTTAGTTTAGATAAAAGAATATCGTAGGGTTCGCGCTCAACCATTATAAATGAAAAAACTTATAAGTATCTCATACATTTGCATCCTATTTTGAAAGTTTTTCCGATTATAATTTTTTTAGATTTTTCGTTGCGCTCAGATACATTTCTAGGTGGACTTATACATAGCAAGTGATACATTACATAATACGAGAGATGTATTCTAGAGAAGATAGGAATGTATCCAAGAGGGAATAAGGATGTATCCGAGAGACGATAGAGATGAATCGAAGACTGATTTTTTTTTATAATTATAGTAACAGGGAATTTTAGAAATCATAATATCTTAAGTTGTGTTTTTATATAATTTTTCATTTCAAAATATTTTGATTAATCAAATCCGTGTGAAATTTGAATTAAAAGGTTTAAGATGTGTTTGGCCATATTTTTAATAACATATTTTACCTTTAACAAAAATGATTTATAATCATAACGAAAATATATTTGACCTAACATCAAACATAATTTATATCCCTAAACTCTAATAATTAACAAAAATACCCAACCATACCACAGAAACAATACTAGCTAATCACAAATTATGCAGAATCTTCGTTGATGACAAATCACAATCTTATATTCATATAAGCTTGCAAATAAAATTATCTTTATATATATATATATATATATATATATATATATATATATATATATATATATATATATATATATTCTCTAGCGTAAGTGTGGGGATGGACTTCACATTGATAGTTGGAAAGAGAAAAAAAGCTATTACTCTCTCCGTTCATTTTTATTTATCATATTGTGCTTTTCGAAAGTCATTTTAATCAATTTTCAAAGTTAAATTAGATTACATTGATTCGATATTTTAAACAAATATTTAAATATTCGAAAACTATAAGAAAAATACTATAAATTGCAGTTTTTTGCATATCAATATGGTGAAAAAACACATCTTAAAATGTTAGTCAAAGTTCTTATAGTTTGACTCTAAAAATAGAAACCATGACAAACAATAATGGACGGAGGGAGTAATAAAATTAGATAGATACTCTTAATGACGTAAGATTTTTCGAGAAACCGATACAAAATTTGACCTAAAGCAATCAATCAACCATGCTGAGGCTAGCATAACAATCATCCATCTGTTTCATATTAGTTGATTATTTTTCTTTGTATAATGCATATTAAAAAAATCATAAATAAGATGATAAATTTTACTAATTTACCCCTCAAAAAGTTTCTTGAAAATTTTACAAGTAAATGTAAACACTTTCGAAAAAAATTATTTGTAAAAGTGATATAGGAAAACAATCTAATTAGTACTTTCTAATTTAGTAAGGTAAAAAACTAAGCAATTATTTTTAATAAAATGATCAACCAATATGAAGCGGAGGAAGTTATATATAATATAATGTACAAGTATCCCCTTAACCTATACTCGAAATCTCAAAGACGGACTTATACTATACTAAGGTCCTATTACCCCTCTGAACTTATTTTATCAATAATTTTTACACCTTTTCGACCTACATGACACTATCTTGTGGGTCCAACGCCGGTTGACTTTTTTTTCAAGCTAGTACCACGTAGGCCAAAAAGGAATAAAAAATTACTTAAAAAATAAGTTCAGGAGATAATAGAACTTTAGTATAGCATAAGTGTATATCCGAAATTTCAGGCATAAATTAAGGGGGTATTTGTGCATTTTCCCTATATTATATAATTTCTTGAACAATATCATTCAATTTTGTTGTCGACATAAAAAGTTAGAGAAATTAAAGATTCGATTGGTTAATTAAGTAAAAGGTCATCGACAAGATATATAGGGGACCATAAGTAAAAAAAAAAACGAAAAAAAAAAGAGGAACATAAATCCATAATCATAATTAACAAGTGATCATAGCAGAAAGACAACTTAATTGGCTGTTTAATTTATGTCTAAGTTACTTAATTATCTCAACGTTAACCACTAGTCAAAGGTTAACGGATTAATTATATTACATAATCAATAGCTAATAGCTTACGTGATAACAGTATTTAAAATAAATATTGGTATAATAAATTTTTAGGAATCTAATCATTTTGTCTTTATTTGTCAACAAAATAAATAACAATGAATAGAATAAAAATAAACAAACAAGAATTTTGATGGAGCGTTAATTACTATTTCATTCTTGAGTAGAGATCTCAATCTTAATTCATTGGATGTGCAATCTTTTCGATAAAAAGTAATTTAATTTCCTAATATAAATTCAAAATTAATCGAACCTCAATACATCAGATTAAAAACCCGGTAATAATATATCGTGAAATGACAAATTATGTATGCAATTCTTTCTAGAAGATTAGCAAACGACATAAGCACATGCCAAGCACAAATAACCACATTTAAATATTTGATAATATTAATTAAATCAACTAACTTTCGTGCCAAGTAATCTAATATGAACATATATAATTATCTTTAATCTAACTCAAAGAAATGTATTTATTAATTTAAAAATTTTGAAATAATATTAGCAAATTAATATTTGATTCACATATGGTCTTTGGTCCACTACTATTTCAACTTCAACTAGAAAAAAAAAACTATTTGAAGGTTGAAGACTAATTTAAAAGTATTTCAAGAACAATAATGATTTTATTCGTAAGAAAATTAATTTAAAAAAATTAATTATTCATATATAAAATAATATGTTATTTGATTTTAACCCAAGAAATTTACGTAACTAATATATACATAAATTATGTATAATTTTATGTGTTTACACAAAATAAAATTACTTATCAATAATTACTAATACATATATAACTCTAATAAACCATTAAACGAAACTATATATTTAAACTATAAATAAATATTTATGAAATAAAATATGGTGTAATCAATGACGTCATTAATGGCCCCTTATTCAAGTTGTGTTTTTGCCTTTTTGGCCACGTGATATAATATTGCACTAAACAATATATCCACTTGCCCACAATAGAAACTTCTAAGAACCCACCTTATCACCAATACATATAAACTTTTTATATTACCATTTATAATCAATGAGAAATAGTAATTACTCATTCATTACGAATTAACACAAAAAATTACAATGTATATCGTGATGATGGATTGAGATTCTTCTTTTCTTTATCCAATGTCGTGAATTTAAATTTTATGTATAAAATCTTTTTAATTAATCGTATTATCTTTTTTGACGAGTTTAATAATATACATAAAAAAATATATCCTCGAACTATAGTAAATGATATGCAAATATCTTTTTTAATATTTTTGGGACAATAATGTCTTTGTCGTTTAAAAATTAGACCATATATATTGTTTATATTAACGGACAAACAAATGTTATAATCATATTCATTAATTTGACAGTTTGACGTATAAGATTATATCATACATGTCCCTATTTAACCTTCCGTCAGAGTAAAGTTATATATGCTTTACTTTTTGGACGACAGAAACATGTCATCAATATTCCAAAAGTATGACGAAGGATTTTTGTATATCATTTAAGATAGCTAGAAAATATTTTTATCTTTTTTCCGTATGTAAAATTGAATTAGTGAAAACTTTTTTAATATAGATATCCAAAATCGAATAGGAAAAAATATGACAACATCATAAATCCTTTTATAGATGCATGAATCTCATTCCTCCTCACTTCCCTATATATATTTCCTCCCTCATTCTTCTCAAATCCAAATTGAACGCCAAATACCTCATAAATCACTAGAAAAAAAAAACTAAAATTCAAAGCGAAATGGATCAACAGTTACCACCGACGAACTTCCCGGTAGATTTTCCGGTGTATCGCCGGAATTCAAGCTTCAGTCGTCTAATTCCCTGTTTAACTGAAAAATGGGGAGATTTACCACTAAAAGTCGATGATTCCGAAGATATGGTAATTTACGGTCTATTAAAAGACGCTCTAAGCGTCGGATGGTCGCCGTTTAATTTCACCGCCGGCGAAGTAAAATCGGAGCCGAGAGAAGAAATTGAATCGTCGCTTGAATTTTCACCTTCTCCGGCGGAGACCACGGCAGCTCCGGCGGCTGAAACACCGAAAGGAAGACATTATAGAGGCGTTAGACAGCGTCCGTGGGGGAAATTTGCGGCGGAGATTAGAGATCCGGCGAAGAACGGAGCTAGGGTTTGGCTTGGAACGTACGAAACAGCTGAAGAAGCTGCAATTGCTTATGATAAAGCTGCTTATAGAATGAGAGGATCAAAAGCACATTTGAATTTCCCGCACCGGATCGGTTTGAATGAACCGGAACCGGTTCGAGTTACGGCGAAAAGGCGAGCATCGCCGGAACCGGCAAGCTCGTCGGGAAACGGTTCCATGAAACGGAGAAGAAAAGCCGTTCAGAAATGTGATGGAGAAATGGCGAGTAGATCAAGTGTCATGCAAGTTGAATGTCAAATTGAACAATTGACAGGTGTCCATCAACTATTGGTCATTTAA

>SlERF8-8

ATATTATACATATGTATTTGTATATATGGAAAGCGAGACTGGGAGAGGGAAGAGAGAGGCGATGGATATTGGGAGAGAGAGGAGAGAGGCAAGTGAGATTGGGAGAGAGAGAAGAGGGGCGAGCGAGAGAGGACAAAGAGTGGTAGAGAGGTGAACTATAAATATATATCAGTTAGATAATTGTATATTATAAATATGTATTTGTATACTCTGACTAATTATACAAACTCAAAGTCAGCCCAATTAATTAATGTAGAATGTTAGTCACGAGTGATAATTATAGCAAATTATAACTATGATAAATAATTAAATAGTATAATTTTGCTTCACTGCAGAATTTTTCCTTTTTCTAATTGTAGCCCCGAGAATTTGTCAAAGACTCAAAAGTCCCTCCAAAATTCATTGAAGAGTCAATATATTTTTTCAGAGTCAAATAATAGGATTTTAATTTTATAAAAATACATTAATATCTCTCGAAAAGTAAAATATGATAACTATTTAAAAGCAAATAAGTGTGACAGATAGAAGTAAAAAAAACAAAAAGTTCAAAGCTAAATAGTCCCTCAAATAATGCGGCCAAAAATGCATGTTTAATTTGTTATTTTTGGCATGTAGAGGCTGTGTTTGTACATTTTTATTTATTTTTGACATCTTAGGATTTTCTTTTAAATTTTGAATATTAATACGTAATATAAATTTATGTCAAAGTCATAGAGCAAATACAATTTGGTTTGCATCGTCTTGTGTGCCATTCTTCATTAATGTCATAACGTTCTTCTGCGACTAAAGTAAAAACATTACAAGTAGTCTTTATAAATATATTTCAAATTTGGTCAAAATTTTGAATAAAGTTGTTGATGAATTATTAATTCAATATATCTACTTGCTTAAAAAGAAACACAATGATGTCTAAATGATATGTATAATGTTTTTTAATTTAGTTATAAAAATCACAATAAAAATATATCCATTGGAATATTACAAAGTGTGACAGAAAAAAATAGGATATAAACATATCTAAATTATCACTGTTTTATGGAGGTAGAGAGACTATTTTCGAAAGATTCTCAGTTAATATGTATTAAACTTAAGTAAAAGAAAAATAAAACAGTAACAATAACAAAATAATGTGATAATCGAAATATAATAAACAACATATAATAAAAACTACTTTAAAGGCACGAGCGATTACTCCTACGAAAGCACTAAACTTCCACGCGTGATGTGACAAAACAATATTTTGTCCATATTAACTTTTCTATCCAAGTACGCGATCTTTATTTTTTTCTATCTAAAATTATATCCTCGATAATGTGAATTTGTGTTATATAAATCACGTATGATCACCTCTGCACATGTTACATTTTTTCACAAATGAAAAAAATAAAAAATTCAGCCTTCTTAAAATTAAAAAGAAAAAACACAGTACAGTTTTTATTCTTAATCAATTTAGAAAATGACAGAACTGTCTTAATGAATAGAACCGCCATCGTCTTTAAATTATTTTCTCTATTTATTTGTGTGTGGAGGCTTTTTTTTATATATATATAAAAGAAATACTTTAAGATTCATCTGTTTCCCAAATCTTGCAAATTTAGTAACGTACGTTAAAGTTAGAAGCAGATAAAAAATATTGTTGTTTATGCTAATGTTCATGTAAGCAAATATAATTAGCAATTTTATATTTTATAAAGTTGGATTAGTTAATTAATTAATAGAAATAAAAGTTCAATAAATATCATTAACGTGTCGTCACTTGACGGCTCGACACATGGGGTAGTACATGTTCCTAGTGTTACGTTGATGTAGAGGAGATTTGTTTTTTAATTCTTGTTAATTTTTTTTGTTTTATGTTTTTTTAATAAGAAAATTTTATTTTTACGTATGAGAAATCATTTTAACTAGAACATATTAACATCAAAATACTTTAATGAGAATTAAATACAAACTATAAAAAAAAGGCAAATATGAAACATGCAAATTATAAATAGGTAAAATATAATGTGATCAATAAGAATTTTGAACCTTGAATGCATTTTGAATGCCTTGAGATACTGATCTGATCTTTTACATTTTGTTAAGATATATTTATTCTTTAACTCGATTTTAACTGGCATAATATACGCATGTGATATATTTTTTAATTTTGAATTTGCGTAAGTAATCACTTATATTTATATAGAATTGAACGAATTGACACATTCATCCATTGTGGTGTGTCTTATATGATAATATTGTGTCTTACGTCACATCTTACGTGTATTATGTTAGGATGCATATGACTACATTTCAATTTTATATCAGTTTAAATGTCTACTTGTGCTCACCCAAAGTTGAAAGGCATAAAAGCCGATCAACTAAAATAAATTAAATGACAAATTTATTTATTATGTTTTTTATAAAAAATCTACTACATATATATGTATATAATATAATGTTTTGCTGCGAATAGTGATGGATCTAGATTTTGAATGCTCCCTTGCTATCAATCACAAAATAGAATAAGTAAAATTTATGACTCGTAGAGAGACGATCATATTAAAGTACAAAAGCAGAAAAACAGCTAGAGAAAGTAAGAAGAGAATTAAGAAAAACTTGTCTTAAAAATTTTAAGAAATGCCTACACAAATGCACATAAACCTTAAGCACCCACTTATGACCATTGAGCCAAATCACACTACTTATTACATGGCAACTCACTTTTTAATTTTATCTCAATACTTTTTAACTTTTAATATAAATATAATTATTTTGATACAAAATTAATAGATGCTGAAGTATTTGAAAAGGGAAATGTAGATCTACCGAAAACACGAGTGAACATCCTCTCGCACCTTACATTCGTTCCTTATTATAAAGCTTCAAACTACATCGATAGAACTCTATAATAATGCATTATAAGTTTCTTTCTATAGCCAAACGATATAGTAGTACACAAAGAAAACTATATGTATCGACTAGAAGAGGCTCAATTGGTCACTAACACAATTAAAATATTTTACATTTGTAAGTCTAAATTTGAGAGGATATAGCGTACAAGTCCTATCAAATAAGGAGTTTATTCCTGAAAGTACCATACAACCAATTAGCCATCGTTGATGTTGACTAGAATTTTATTACAAGGTTAAGAGAGATTCAAATGATTAAATTTGTAAGAACTAAACTATTAAATATAGACACGTGTGCAAATGAGACCAACTATATAGTAGACTAGCTAGTTAATTTTTAATTAATATAACAAGTGGGACAATTTGTGTTTTTTTTTTTAATACAAGAAAATTAACGCCCTATGACAAATTCATTTTTGTCTAAAATCAATGAACAATGTCAATTGAGGGTCCAATAAGATATTTGCCTTTTTACTTGTCCAACATTTTGGCTCTCTCTTGACGTGGAATTAAAATGAAGAATTTTAAACTAGACAAGTTGATGTTATTATTGTTAGGAACTTACAAGTGTAATTTCCTATTTTACTATCAATTCGTGTATTAATTAACCTAAAAGATTTAAAGTAAGTGGAAAATCTTACTTAAAAGTTTCGTGTTTAAGTTTTCTTTGTTCAAGAGAAATATTTTACTTTATCTGCAGACAAATACAAACAAGGTAATGACGACCTTAATGCTAAGTATCAACATTGAGATATTGATATTTGAAGATGAAAGGGATGAGCCTAAATATTGAAAACATAGCATGAAATTTCATTTTCAATATCATGGATTTTTCTTATTGTCCATTCGAATTATTGGTCTGACGAGAATAGTATGTATCCAAATTTTATTCCTATCTTGTGCAAATACGCAGGTTATGTTAAGATTGAAATAATTAAGCATATGAAAGTTAGTAAATATAAATCTAAAAAGACCGGAATAACAAGACAAATGAGAAATATATCAGAAGAAACATAAATATTTAACGTGATTTGATCAATCAAACTATATCTACAAAAATAAATGACAAATCGACTATATATTCTATATGAGAATGTACTAAAATATCGAAAGAAATAATTCCACACAATTCTTTTAGAAGAAAAAGAGGTGCACACAAAACACTTATCTCAAATATCCTCCATCTACGCAAAACACTCAAAGTCTTTAACGGTATATATGAGAGTGTAGTAGAATATCGAGAGAAATAATCTCACACAATATTTTTTAAATAAAAAGAGGTACGCAAAAAACACTTTATCTCAACTATCCTCCATCTATACATAACACTAAAAGTCTTTAACGGTATTAATGAATGCTACCAATTCAAAAAAATTGTGCTTCTATTTATATCATCATAAACTTTTTTCAACAAGAAAAAGACTAATCATTTTTCTTTTTGAAAAAACATAAACGGAAAGCATTACATGGTATATTGAAAGAGAAAAAAAATAACAATACCAAAAAATATGATAATCAAAATAAAAAATAACATATACTAGCAATAAAATTGTTTTTTTTTAAAAAAAAAACTAAGATAACAATAAATGAAAGAAAAAACATATACAATTATTCAAATTATCACCAAACCTATCCAACAACACACTTCCACACCTCCCATAATATTATCTCGATCAATCATATCATAATTCTAACGAAAAGCAAAAGAAAGACTTTAATGGAAGTCATGGTCCATATGTCTACGACAAATAGTGAATTACTAGAAAAATGCGGCTCAGTAATTTTATCTTATTAGTATTATTCAAAATACCTAATTTCCAATTTTCAAATACAACACGTGCTTATTGAAACTCTCTATTTCCCTCTCAAAAACTTAGAACTTTTCTCCATTTAGAAGTTTCAAGGAAAGCTCAAATTCCAAACAAACAATATTTTAATCATTATTGTTATTAAATTTTCCCACCCGTGCATGAACCAAATTTCATATAAATATTTCCCTTTCCCCGCTTTAATCCAGGAAAAAAAAACTCATAACTTCCTATTGTTTTTTCTTGATTTGCTCTATTTGTACTAAATTCGCTTCGATATAAAAATTCATAACCAAAATTCAAAATGTATTCAAATTGTGAACTAGAAAATGATTTTTCAGTACTCGAATCAATTAGAAGATACTTACTTGAAGATTGGGAAGCTCCATTAACGAGCTCTGAAAACTCAACATCCTCAGAGTTCAGCCGGAGCAACAGCATTGAATCCAATATGTTTAGTAATTCATTTGATTATACACCTGAAATTTTTCAAAATGATATTCTTAATGAAGGATTTGGATTTGGATTTGAATTCGAGACTTCTGATTTTATAATCCCTAAATTAGAGTCACAAATGTCAATCGAATCACCTGAAATGTGGAATTTACCGGAATTTGTGGCTCCATTAGAGACGGCGGCGGAGGTGAAAGTTGAAACACCGGTTGAGATGACAACTACGACGACGAAGCCAAAGGCAAAGCATTATAGAGGTGTGAGAGTGAGGCCATGGGGGAAATTCGCGGCGGAAATTAGAGATCCGGCGAAAAATGGAGCACGAGTTTGGCTCGGTACATATGAGACGGCGGAGGATGCGGCGTTGGCTTACGACAAGGCGGCTTTTCGCATGCGGGGATCACGTGCATTGCTGAATTTTCCGTTGAGGATTAATTCCGGTGAACCGGATCCTGTTAGAGTTGGATCGAAGAGATCGTCAATGTCGCCGGAGCATTGTTCATCGGCGTCGTCGACGAAGAGGAGGAAGAAGGTTGCTCGTGGAACAAAGCAATAA

>SlERF1-11

TAAATGTACTCCATAGCAAACGTTTGTCAAAATTTGTCAGTCGTCCCTCTCTCAAAAATATCGCTCGCCACTCTCTCATTCTCGTTCCAATCTCTCGCTCGCTTCCTCACTTTTTATATAAATACAAGTCTATAAAAACTGTTTCTATTTGTATAAAGCATAGAAAAATTGTATAAATACATATATTTTTGTTCTCATTTCTCCCTCTTCCAGATCTCGCTCGCAACCCTCGTCTTTCCCACATATACAAATAGATACGAAATGTATAAATTGTGTTTCTGTTTGTATAAAGCGTGAAAAAATTGTATATACACATGCAAGTACATATATTTTCATCCTATACACTTATAATTATACAATCTCACGTGCCCTGTTTTTTTTTCCTCTCTCTTTCTCGTTTTATACAAATTCAAATTGTATCTAATTTCACTCTTTCTTGTTTTATACAATTCGATTTAATTGTATATTCCCTATCCAAGTCTCTTTTATCTTTCTCATTCTCATTTTATACAAATTCAAATTGTATATAATCGTTCTATACACTTTTAATCATACAATTCGTTTTATACACTTCGTTTTTATACAAATCTCTGTCCAAGTATTTCTCTCTTTCTCATTTTATACAATTCGCTTCAACTTTATATGTATAACGAATTATACCGTTTTTATGTTTGCTATGGAGCGCAATTATGCAAATTTTGCTATAACATACAAATATGAATTTTTTATTTGCTATATGTGAAAGTTGCCATTTTTTCATCCATGCTTCTTTTATATATATATAATAAAAATAAGAAATTCAAAAAAATGATCATCACATATGGAAAGAGTATATATGAACCATATTTGGTATGATAGAGATAATATGGTAAGAATATATATTTTTGTCATTTTCGTAATAAGAAATATATTAACTTTAAATAATACAATAACAACAACTAATAAATAAAATAATAATTTATATAATAAAATAACAACAACTAATAAATAAAATAATTTATAGGGGGCAGAGAATAAAATAGTACTGCAAGGATCCCGTGATCCAAAATAAGTTTATATTTTTGCAAGTACAGTTGCTTTCTCACTTTTCAACAAGGACCGTCATTTTGGTTAAAAGAAAATTACAGTACATTATTTATTTCTCTTGCAATAAAAACAAAATTATTGTCTTAAAAGTATTTAGGGAAAAAGATTAAAAAAAAATATTTCACAATTTTTATTTATTAATTGACTTTATTCTTACCTCTAAAATAAAGCTAAAAATTACTCTTACAATATTGCTATACTTATTATTTATGTAACTTTATGAACGAAAATCCCAAAATCAATTTACCCTTTTATAACCCAAAGTTGATTTGGAAATTCCCGTTCACATAGAGGCACGAATGATAAAGTATAGTAACGATAGAGATAAGAATAATTTTTATTCAACAGTTATAAAGTGGACACATATTATTGGACATTCTAAAATAATATAATGAACAATATTCTTGGACAAAGAGAGTAATAAAATGTTATAAAAAACATATAGAAAATCCTGTTAATTTTTTTTATTAGTTTGATATGTTAAATATAGTGATAATAAAGAAAAAATAATTTTACGAGTTTCTAAAATACTAGAAAAGAACGAAGAAAACTTTCTAAAATTGAATTGAGTTGGTTTAACCCAATTCATTTTTACCCAAATAAATTTGAATTAAATCTTGATCTGTTATAACATATTTAAATTCGATTTTACGTCACCCAAAGAGATAATAAACTTAAAATGTTACCCAAAAACACCAAGTACAACGAATCGAAATATTCTAAGGAATCCCTACAAAAATATTAAGATTTAGGTCTAGTGGTAGGTGAATAAGTTAAAGGGAGAAACTTTATTATGTACGAAGCACAACTTATACTAATTGTATGAGTGAGTACTTTATTATTATTATATTTGAACATGTGGGACCTGCACTTTATTTTTCCTTCTTTGTGTTTCTTCTCTTTCCCTTTTTTTTCTTTAAATTTATTTTGAAACTAATTTAATTATTTTATCATTTTGTGCATATATAAATAATTTTTTAATCTAATTATTTTAGTCGAATTATTAAATTTTTTCATTGATTTAAAAAGGTACGAGCATACTAAAGTTCAACTCAATTCTTTAAATGGTCACATATATCATAAAATTGACCACTAAAATTATTTCTATTTCTTTTCTTCCATTTTTCATTTATAATACTTTAAAAATTTTCACCTTTGTATTTCGTATCGTTTTAATTTTTACTTTAAAATTTATCTAAAAATCATTTTTACTTTATTTAAAAATTAATGTTTGATCATGAAAATTTAAAATACTACTTTTCAACTTGAAGGTAAATTCAAATTTGAAAAACATGTTAGTATATATGTTTTTAAACTCATATATATATATATATATATATATATATATATATATATTGTTTTAGAGATTCATTTTACTAAATTAATCTTATTAATTACATTATTAAAAATTATGGTAATTATTAATATCTTAAGTAGTTTTGATAAGAATATTATGTTTTAATTTTTTTTTGTTAAAACAAACAATACAGAGTAACTGAATACTTTTATATTAAAATAATCTATTTTTTTAACAAAAAATACATTTAAAATATTTTTTTTATCATAACTACATAAGATACTAATAATTAACATTATTTTCTTAAATGTTATAAATAAAACTAATTTAGTAAGATGGATCTGTAATATAATACAATTTAAAATAAATTTAAAGGGAAAAAAACAAAAAATAGAAAAAAAAACTGGAAAGAGAAATAAGAGGCAAGGAATATAAATAAGTGTAGGCCTACGAATTCAAATTTAATAATGATAAAGTACTCCCTCATATAGCTACGGTCAGTTGTGTTTCATCCACAATAAAATGTTTCAAAGTATTCTCTCCATCTCAATTTATGTGACACTTTTTAAATTTTGAGGCTCAAACAAATATTTTTTATCGTAAGTATTTATTATCTTTTAAATATTTTGAAATGTCAACTGTTATGACTTACGTACTTTTTAGATAAAGGACGTAAAGTCACTACTATAGTTATCCTAAATTTTTAAAAACACATCTAAATTTTGCAATCGTTTTATTACTCACTAAACAATATTTAAGTGATATCATTATATCATTTTTCAATCAACCCAAATTTAAAGAAATAAATATATTCATATACCATCATTACATGAGACGTGCTAATTTCATTAAAAATATAATTTTCTTTCTCTCTCTATGACTTATTTTTATTTTGATTTTGATTTCAATTTCAATTTCACTTCATCCCCTCCAACCCCCCTAAAAAAAACTTTTAAGTTGATCCATCCCTCCATTTTTTGCAAACCCTTCACCTCACGCCGACCCACCATGTCAAGTTGATCCACCTCCCATTTTTTATTTTTTTTCTTCTTCATCTCCAATCAAATACTTTATTTTAATTTTTCTAAATAAAAAATCGTATAATCTTCTAGACTTTATTGAATTATTGATGACAAAACTAATTATTTTTCTAAAAAAATTTAAAATTTTGAAATTACTCTTTCTAATATAACTTCAAAAATAGAAATGATAATATTAAAATAATTGGTGAATTCCCCAAAAATTGAAAGAACTTGATTGAAGTCGGATAAAAAAACTATATAATACCTCCTAATCATCTTGAAATCTAATAGGTTTATATGTAAGAAAATATTTAGATAAAATTTTAAAATCAAAGAGAATAAAAGTAATAGTAGAAAAAAAAAGATGAAGTTGTGGTAAAAATCGTGAAATTAAGAGTGAGAAGTTTAAATGGAGATGACAATAATTTTTTTTAAGGAGAAAGAAGAAAGAGAAAAAATAACGAGGAAAAAAGTTAAAATCGTAAGAAAATAAATTACTAGTAAAAATAAAATTATATTGTCTATCAAGTGGACAAAAAAATATGTAAAAATATTTGTTCATGATTGTTTTATGGAGGTAATAGGATGCCCACATAGTCAAGGTAAATTTTATCATGGTAGAGACCCAATAATGTGTGAGTCTCCATTTATTTTTTTGACACATGGACAAGTGGGCCATTTATGTATTTTTCTAAATACATAAATAGTAAAGAACTTGATTGAAATCGAATTTTTAAAAAACTATATAATATCTCCTAATCATCTTGAAATCTAATGGTTATTAAATTGTTCGGATCGTAAGAAAATATTTAGATAATGTTTTAAAATTAAAGAGAGTAAAAGTAATAATCCCTCCGTTTCGAAAAGGATGACCTAGTTTGACTTGAAACGGAGTTTTAGAAAAAAAGAAATTTTTTTAATCTTGTGGTTCTAAATTAAAGTTCTGTCAAATGTACCAAAATACCCTTTAATCATGTGGCCTTAAGCATGCCATGTGGAAAAATTAAAGTTAAAGTGTTGCTAAAAAAGAAAAAGCAGACATTCTTATTAAAAAGGAAATACAGTCATTCTTTTTTAAACGGAGGGAGTAATATAAGGAAAAGGAAGGTGAGAAGTTTCAAAGAAGATGACAATAAATTTTTGAACAAGAAAGTGAAAAAATAAAAGAAAAAAGGTGAAAAATAATAAGAAAATATCTTTTATAATAAAATACTTTAAAAAAATAAAATTATATTAGTTATTTTAGGTTTGTCCACTCTTATGTCAAGTGGACAATAAATAATAGAATTATAATAATATCAGTTCAAGATTGTTCAGTGAGATAATAAAATTAACGTGTATTTTTGAAAATTTAAAACAATTTCACGTGTCAGTTTATACATTTTTTTCCTCTATTTTTTAAAGTAGTATATAAAAAAGATTCCATGTAAAAAAATTTTTTGATTAGTCTTAAATTATTTGATTATGGGAAAAGAAAAAACGAAAAGTGTCGTATAGACGGGGTGAGATGGAAGTAGTAGTATATATAAGTAGCACTAGAGTTCCACTACTAATATTACATTACATTCATCACATATTATTCATCCAAAACAAGAATGAGTATTGTAATTGATGATGATGAAATCTTCTCTTTACCTAGCCTTGATGAACTTGAATCCATCACACATCTTCTTTATGACGACGATTCCGATTTTTTCGAAACTCTTTCCCCAATGAGTTTAGATGTTACAACATTATTGCCTAATATTCCTACCTCCAATTCAATTGAATCCCCCGTAACACCGGAGGAAACAAAAGAACCATCTGTGGCGTGTGAGGACGCGCCACAAGATTGGAGGCGGTTCATAGGGGTGAGGCGGAGGCAGTGGGGCACGTTTTCAGCCGAAATAAGAGATCCAAATAGGAGAGGAGCGAGGCTGTGGCTCGGAACTTATGAGTCCCCGAGGGATGCAGCATTAGCTTATGACCAAGCCGCTTACAAGATTCGGGGAACCAAAGTTCGGCTTAATTTTCCTGACCTGATTGGCTCGGACGTACCTATGCCACCTAGAGTAACGGCTAGGCGTCGTACACGCTCACGCTCACGCTCACCCGAGCCATTAACAACTTCGTCCTCGTCATCCTCATCATCCTCGTCCTCGTCCTCGTCCTCGTCGGAAAATGGAACGAAGAAAAGGAAAATAGATTTGATAAACTCAATAGCAAAATCCAAATTACTTTGTGGGATGGATTTACAAATGTTAATACAAATGTGA

>SlERF6-3

TGAATGATACCTCATATCAAAGATAACATTGATAGTATTTGATATCGATTCCAACAAAACATAACTTATCGAGATCTCTTCATTTTCATTATTTTTCAGGTACCTGAACCTTTTCGAGGTTTTATGAAGTGTTATGTCAACGTGCTCTTTTATAGCACTTGTCTCATTATCATGATCATACTGATCATTTGCTCATCTCCTTCTTCAAGGAATTTTATATTTGGAATCGATTGGTCTATTACGCTTCCGGCGTATCATAGACTCTGTCCTTCAAGGATTTCACATTGAAGTATTTGCAGCTGAATTATATCATAGGGCCAGTACATTCATCTGACAACTGATTTGCAACATCTTGCAAATGAATTATCTCTTGAACTTTAAGTTCACATATTTATTTAACGAGGATCTTATTAATTCATAATTTACCTCACATAATTTTCAGCTGTCAAAATCATCTCTCCCCTAATGTTAGAAAATCTAACATTCATCCCCTAATGCTTATATTAGTAATACACTCTATTGTGTATTAAAGAGTGTATTACTAGTACCATCTATTTCAATGCATTAGTAATGCAATGGGTTCTAATGCATGCATTAATATTTTAAAGACACAATTGCCCTCAAAAACCTTTTTTTACATCCTTTCCATTATATTTTTGAAGGATACTTTTGTGAACAAATACTTTTTCCAAAAGAAATATGTAATGCATAATATTTTTAGTACATTAAACCAAACATTGCATTAAAAGAATCTCATTATAACTGATACAAACATTACTAGTACATTATATTTTGCATTATTCTTATATACTTTACCACACAATCCCTTATACTGTTAAAAAGAAGTTAATTATAAAAATACATATATTTAGTTCATTCTATTTTGATTTTGGTAATTTACCAAGATAACTAATATCCAAGTTATTGTATTATGTATCTATTCTATGTGTTGGCAATGAATATTTTTGAAAATTTAGCAACGGTTGAATGGTGATTAAGAAAGAAGATGGCATGTGTAGCGCCACAATTCCATAAATGTGTGCAGAGAAGTAGAACATGTGAGCACTCTCACATAATGAAAGTTCAGCTTTTTATTGATTTTACGTGCCAGCATCCCAAGACGGACACAAAGTACCATCTCCCACTTGCCTATTTTTCCACTTCCACTTCTTCTGTAGCTAATGTAATCCAACTGCAAAACATTTGTATTATAGTAAGTAAATTAACACAATGTATTCACCAATATTGTACGAATTTTGCATTCAATCTAAAAGTTATAACCCCATAACTAACCGCAATCTTCGATTCTTCTCGAAAACCATACTCCGTTTATGTGTTTGATACAAAGAAAAATATTTTTATATTTTTAGTTTTTATCATTTTTTTTGGCAAATATTTTATTTATGAAAAAAATAATTTTTTTAAAGATAAAAAAATGACTTAATTTATAAAAGTAAGAAAATATTGTTTCATGAGCGATAGTCTCTTAGGCTCCATCCCCACATCTCTACCCGACCCATCTATAATATACTAAAGATATTATTTTATGTTGCATATCAAACACGGAAAAAAATAAGTAATTATTTATCGAATCAATCTTTAGGTGGAACTTTTTTTTCTTCGTACCAAAACACACCCTTAAAATGTAATTTGTATTATTTCAATGCTGATGTTACTCCAGCAACCAATAATGTTGTTATAGTCATTGATAGGTTGGGCTGTATTTCTTTTCATTATTAATCTCCTCACATTACTTTTTTTTTGAATTTAGATTTTGTATACTCAAATGTTTTAAAATATTTTTACACAAAAAATGTTTCTAATGAGGTGTTGCTCACTACTATTATTTTCGACTAATCAACTGATATATAAGTAAAGTTTTAGTAATGGCTAAGATTAGTGTTTGAATAGTTTAAATAACTAAAGTCGGATCCTTAAAAAAAGTACAATTAATTAGGGGTTAAAAATTTAAAATTCTAATATAAAACAGTACGATTAATGAAACAGAGTTCCAAGGGGATAATCATATTTTATTCCGGGTGGAGCTAGCCCGATGCTAGAAGTTCATTTAGATCTCTTCTGCGAAAATGATATTGTTTATATATGATTAAAATTATTTTTTAAGTATATCTAAATATAATAGATCATGAACTCTTGGCGGAGCCAACTTAGACTTGAAGGGATTCATCAAAAATCCTAATAAAAAATTACACTAGCTATAAATAGTTAAAATTATTTTTATGAATCCTTTTATTAATTCATATGATTACCTATTATTTTTATGAATCCTTTTATTAATTCATATGATTACCTATTTATATTTAGATACTTAGTAATAATCCTATGTATCGTTAATTCTAACTCAATCTTATATTTACTTTTTTATATTTTAAAATTGTCTACATAAAATTTTTGAAACCATCACGATTATTTATTTGTATCATACGTACTATTTCACTTGTCCATGTGCCACTTTGTGGGGTGAAAGATTGTTATTGCCAACATTTTTACTGAAAATAGCGTCGACAATTGGGACACTTGCTTCTGGTAAAAAAAAGAGAGGCTCCAGCCATACATACCCAACACGCATCAATGCGCGTCTGTACCACACGCTCTAAAAGAAAGGGTATTAGTGCTGTACGTCTCCACCACGAAGACGAGAATCGCGTGGGTTCAGTTAGAATCCTCGATGAAAGTAAATATAAAGAAGGTAAAACCCAGAGCCGTACAAAACCAAATTATTAATGGCGGCTAATTCTAGGTTGTCCACCACGTTATTCCTAGCTGTAAAAGTAAAAAAAAAAAATCATTTAAACTTCAAAATTCATCTTTGTCCAAAGGAAAGTCCAAGAATCAGAAATTTTGATCATAATGTATTTGTCATACACAATTAATGCACATAAACTTTTCCTCCTACTCAGTGTCAATGAATTTGTTTGGGAGGATCAAATACTAGATTATATAAAAAAAGAATAAAAATTATTTTTATTTTTTCATCCAAATTAAAAAAATATGATGCAATAAATAAGACTATTTTCTTCTTTTATCTAGGTCATCAAACATAGTCTGTATTCTAACAACGTAGAGCAAAACAAAAAGAAAAATTGAACGTCACTTGACTATATGGAGAAAATAACTAAGCTGATTATTATTACATCGTTTCACTTGAAAATCCTTAATAAGCAGTATTTTTTCAGGTATTCAAAATAATCGAACCCCAAAATTTATTTCATTGACTTGATTGATAAGCATGTTTTAAATAGAAAAAAATCTCATTCCATAAAATTACCAAAATAAAGGAGCAGTAATAATTATAAAAGTTACTACTTATTTATATGTATTTAATGGTCAAAGAAATGAGTTGTTAGCCAATTGCATAAGTATTTGACCGACATAATATTTTTAACAGAGCGGGCATCCTGGGCCTCACGTGACCGGTCACACGGGCTTCGAGATTTTAAAAATAAAATGCTGACTCATCTGTAATTTGAAGTTCATACATTGGACCAACCATATTACTGGGGCCCACTATACTTACGTGTCAAATCTTTCGTGGTTTTTTTTCAAACTTACATATATGCGGCGAGTAGTTTTCTCTCACCGTCGACGGAATCTTTGGGTCCCATAACACCGACGGCCGGTGACTACCCCTTTCACAAAACAACTACTCCGATCAGCTAGTATTTCCTATGTTTCTTTTTAATTTAAAACATCGATGTTATGCCACACAACATATATAAAATTTTGCATAATTCAAAAGGCATTTTGATACGTCATATATATTTTTAGTTAGATCAGTATAGTGGTGTAGCTATTTGGTTGTCGAGGTGTCCAATTATTTCGAAAGAAAATATCGTATATACAAGAAATGATTAAATAACATCTTTTGATATCCTTAACACAACAAGCCAGTGGCGGAGCCACACTGTCATTAGGGGTTCATCCGAACTCCTTTCAGTGGAAAATTATAGTTTTTTACATGATTAAAATAATTTTTTATGTATATATAGTAGGTGTCAAACCCCCTTCGACTATTTCGTATGTCTATTTCTTCAATTTTTGAACCCCTTACTAATAATTTTGGCTCTGACACTGCAACAAGCTATTGTAGGGTAGTAATTTCATATGCTTGAAATGAAATAGTGCTTGCGTGTTTGAATCTCACGAGCATCAAATGTTTTCTCCACTTTTATTGTGTTATTTCTAAATATTTTTTTAATGAAATTTTTAACTTTGCTATTAGATTACAAGATTCAGAAGTTATATTTCATAAATTTCGTTTGTTCGGAGGAGGGAAGATATTTTTAGAATGTGATAGTGTATGGTACTACTACTGCCTTTGTTTTGTTTTTGGGTACATGTATGGTACTACTGCCATATTTATGTAACATTTAATAATAGTGCAATTTATTATAAAAATAAAAGTAATAGTAATGTACATATATTGGAATTTCGGCTTGTATCCTATAAAAGAGGTTGTAGTCTCACGGACTTGAAGAAACACAACTCTGTCTCTCCAAAACTTTTCTCTTTCATTCATATTTCATCACACAACAACAATACTAGCAAGTATTTGTTTGAGAAACATAGGATTACGAGTTTTTGATTCCTTACCTTTTTTTTGTTTTTTAGACCATATGGTATTTCCCTTTATTGTCCACAAACCTTTTTAACCCTCACGCTTACACTAAATCCTTTTTAGCTCTTCTTTCTTTCTTAGAGGTTTCACTTTGTTAAAAGGGTTTTGCTTTGTTATCACCTTCAAAGCTTTGTGCTCCGTTTCTGTGAAGCTTGTGGTGCTCCTTCTACTTTCTTCTTTTCTTTTTGCCCCCTTTAACTCTATATATTCCATCTTTTAACGAACCTTGCTCCTCTTTTTGTCAGATGTCTTTGGTTCTCTCCTTCTCTTTCAAGCTTCCCTTTGACTATTCCTTTCCTTTTTAATACCTTGGCGCAGTGCCACTCTCAAAACATTGTCATCCTTTTTCTGAATTTTTACTATGGATCATCACAGTTCGCTTTGTCCGATCAAATATACCGAACACAAGAGAACAATCAGAAAAGTTACTAAGCCTTCCGTTATTAAGCCTAAGAAGGTATCAGATGTTCGGAAATCCTCGGAGTATAATCCGAGGACTGTACGGATATGTGTTACTGATCCAGATGCAACTGACTCTTCAAGTGATGAAGATGAGCTTTTTGGGCGTAAGCGTGTGAAAAGATACATTAGTGAAATCAGTATTGAGAGTCCATCAGTTAATGATGTGAAAACTTTGAGTAGTGGTAATGGGAAGAAGCGAGTAGCTGAAGGTTCGCAGGCGAAGCAGAAGGCGTTGAAAGGGAAGGAAGTGGCTGATAAAACGGTTCGGAAGTTCAGGGGAGTGCGTCAACGGCCGTGGGGTAAATGGGCTGCTGAAATCAGAGACCCAGCGCGTCGCGTTAGGCTCTGGTTAGGTACTTATGATACTGCTGAAGAAGCTGCTATGGTTTATGATAATGCAGCTATTAAGCTCCGTGGACCAGATGCTTTAACTAATTTCATCACTCCTCCAATAAAAGAGAAGCCGGAAGTGAATGTGGCTTCGAATTCGGGGTACGAATCTGGTGATGAATCACACAATCTTTCTTCTCCTACCTCTGTTTTGCGGTTCAGAAGTAGCGAATCCAGTGAAGAAGCAGAACCCGGTCTCGAAGACATAAAAGAAAATTGCACAGTATTGGTGGAAGAAGAACCAAACAGTGAGCACCTTGAGTGCCAAGGCGAGACACTTACAGTCATACCAGACTATTCAAATGACTACTTACCCACAGACGTTTCCTTCTTAGACGATTATTTCAACTTTGCAGATGCAGAACAGTCACTGTTTAATGACACAACAAGTTTCACCAATGATGATTTGTTTTCGTCTTGGGATTTCACAAATGACTCTGTGTTAGACCCAGAGATCTGCAAATTTGATGATTCATTTCTAGACTTGGGTGCATTAGAAGTGGATAATTATTTCAAAGACATTGGAGATTTTTCCAGTGTTGATGTACTAATGGCGTTATAG

>SlERF6-11

ACTGCCTGGAACTTCCTTATGATCATTCCCCACTACTTGAGATGATATTTGAAGTGTTTTGTTGAGAAGCTTATCTAGTCTCTGCTCTCTTATGTTCTGGTGCTAGAGTGAGCTGGTACTTCAGGGTGCTGTTCATGCTTATAAGTATGCTAGTTTTTACACCACAGTGTCCACATAACATGATGTAGAAGATGACTACATAATGTAGAAGATGGCTGGCGTTTGTTTGCTGCTAGATTAGTTTTACCTTCATGGTGGTTCTGTAGGTTCTAGGAATTTTGTTTGTTTCAGGCACTGTTTGAAATTTGCAGAATGAGTTCTCTCTGTCAATTCTGAGAACGTGTTCGATTAATAGTTTGACTGGTTTTGTGGTGATGAGTCAAGGGTCTGAATCTGATCTTTTAATTGTTTTTTTGTTTCAGGTGGCATTTAGAACTAAGGTTTTCCATCCTAACATCAATAGCAATGGAAGTATATGTTTGGATATCCTTAAAGAGCAATGGAGTCCGGCTTTGACCATATCTAAGGTTTGTATCTTGGTCTTTGTTTGTGTCCCAATTAAATGTTGCTATGTTTTTTTCTGGTCTTAGATTTCTTGATGAATGTGAGTCCAAGCCAGAGTCTGGATTTAACAAAATAATGATGATGGTGGTGGTGTATGATTTGATTTGGTGGAGAATGCTAGACTTTTGTTATGTTTTGTTGTCCAGACTTGTTGAGTTTTGATCTCCTCTCTCTTCATAATTAAGGACTTTGAGGCATTGGAATCATGGCTCAGTAGGCCAAATAATCTGGCCTGGGAGTATACTTAGTTAAAAAAGCCTTATGTCAATATAAATGAATGTGAGAACTGTGATTGGCAAGTTATACTATGGCTTTACTAATGTGATAGTGCTCTGGAAATAGGATGATTTAGACCAGCATGTTTGTATTTGAAGAGAGAATGCGAGCAGTAATGCCAATCATAAGGAAGGATAGAGTAGAAAAGGAAAGAACAGTAACTAAACATTATCATGACTTGTTTAAACTAAAAACAGTAACTATATGACAACATACCCAGTATAATCAGACTTTACTTCTACCTCAGAGGTAGAGAGGTTGTTTTGACCTACGACGCAAGGAAAAGTAGTCCAGAAAAAAGTAACAGAAGTCAAAAAGATATTTATTAATAAACAAATATCTATGCCCTTCAAAACATGCTTACCTTAAATAACATATTTGTTCTGTCAGATGTCAAGCAGCACCTTAGCAAGTATGATTCCATTTTTAGAATAAAAGAAAGGAAAGTGAGGGGAAGTATCATGTTCATTTCTTTATTAAAGAAAGTCGAGTGTGATATCTTTTGACATTTAGGCTCCTATTATAAAAGTATTTACTATATTATTGTCTGATGTAATGACTAGTCCTTCTGACTTGCTTCTGCTAAATCTTTGGTAGAAATTAGGTGAACTACTTGTGGCAATTTTGTCTCTTTCTGTCTTCCCTTTTCCCTCTAGTTGATGTTGAAGCAGATGATTTATTTTCTAACTTTTGTTGTACGCGTTTGTTGTATTGAGTTGCTGAGTTGAGGTTTGGGTGTTCAGATTTTTATTTAAATGAGAGATAATTTAGTGGTAATATTGAGCTGTTACTGCAGTAAAATCTTTTGAGCAATACCTGCCACTTTAGATTGCTCTTTTTGCTCCAGAAATGGTTTTCCTACAACTCTCATATAATCAACTAATTCGATTCTTCTATTTTATGTTCTTTTTTTTTCAATAAAAATTTATGTTAGTTGCTTTTCTTGATGTCCTCTTCTTAGCATGTTTCTATTTAACTATCCAAAATGTGATGAAAATCAGTTTATGTACCAAGTCACAACTAAATCTTCTGGAAAAAAGGCGAGGGAAGAGTGTCTCTGGTTTATATGTCTTATTTTTCAATTGTCATTTGGATTTTGTCTCACACTGCTATTTTATGTTTTAGGTCCTGTTGTCCATTTGCTCTTTATTGACAGATCCAAACCCAGACGACCCACTTGTACCAGAAATTGCTCATATGTACAAGACTGACAGGGCCAAATACGAGACCACTGCTCGTAGCTGGACTCAAAAATATGCGATGGGATAACGGCATTGTCCTCGGGTGTGTCTAAGACACTTTCAATTTCAATTACTTCATTTTGCTTTCACTCCTAGATGCAATATCTTCATTGTGCTTTGGATGAAAGAACAAAATGTTGGTAAGGAAATCAGTTTAAGTAGACTCCGATCATGTCAAAGTTGTCAAGACCGAGATCTTTCAATATGTGAATATCTATTTGTTGAATTTTAGTGAGGGGAGAACCTGAAATGTAATTTTATTTTTACTCCTTCCTGACGTTTCTTGTATGGGTGCTATCTTTTGAGTGGCGAAAATTCCAGTGATTAAACAGCAACTTTTTCTCTTTATGAGACCATTTTCCTGTGATCTGTTATATTATTAGTTGTGTGCAAAGCAGGTTCTTGAAAATCCGTGTAGCTAGCTAACCATGGCTCGCTGCCGTCTTCCATGAGATAAGTTCTCCCTTGTGTTTTGGTCCTTAAAATTTGACTATTTCCTTCATGTGCCTGGAGATATCGGTGACTCTGGATTCAAGGCTTTTGGTCATGTCGTTTTAGGACAGCTGCAAGAGGTGCTGACGATTCGGCTGAGGCTAGTTAATGTGATGGATTGAGCAGATGATTCTAGAAAAGTGGGAACCACAGTCTGCAGGACGTCAGGGCGACTGACTCTAATGCACACTGAGGTCATTATCTGAACCTTGGGAATCGTTATTGTGCTAATTTTCTTAACGCATCCCCTCAATTTCGTGAAGAGGTCAGCAGTGTGTCAATAAACCAATAGTAGTTATTTTTGGAAGAGTAGTTTGTTCTGTATAAAAGTTTAGCTTGTGTATGGATCACTATCAAGAAAGGTCGAAAGTATGGTGGTGCAATTAGGAAGCTCCAGATGTTAAGAGTTGCATTATTTTGAGGTGAAATTTGTCTGTTAGTATAATTGAGTTGAAATTAGAAGGGGTTAGGATGATTATGCAAGAGTGGGTTAATAAAGAAAACTATATGTGCAAATGATTATCACTTGGTTTACAAGCAAACAAGTCTTTTCAGCCTATAGATTAGATTCACGTGCCATGCTATCAGCCCTTTTTTCTTTTTGCTTCTTATTCAAGTTAAACGTTTCGATATATCTTGGATCGCCTTTCTTGATAACAGACATTTGATGCTTCGTTTAACTCGTGTCAGTTGTGTGAAACACTCATTTGTATTATATGCTAGGTAAAAAGTTGTTATCTAAAGAGAATAGCTTTCTCATGGATTTGCAGTTAAAGACAAGGTGCGTCAACTTCGCAATAGTTTCCTCCTAATTTCTTTAATACTTTTGTGGACTGACATGAATCCTTTAAGCCTTTTCCCGTTTGAAAAGATGATATAACTAATGGCATGGTTGAATCATAATTGATTACGTATATAAAATATTTTTGCAAATGTAATACTATTTGCAATTAACTAGTAAGTCTTTTCTCATTTAATAATAATAGATATGAATATAGACGAGTCTTTTTTCATAGTTCACTGTAGCTTTTTCCAACTATTAAAGAGGTAGGACCTTGGCTCATTATAGAAACCTCAGAGAGTCGTAGAGCACCAATGGGGGCTTCCTTGAAATTTCGTATTCCAAAATCAAGAGAACATTGTGCAAAGTTAGGTAAAAAGCAATATGCAAGACCAGAGACTTTCTGCTGTGTATATAAATACAAAGAAAAGAAAGTCGGGGCTGCCGGGTGTGGGGCTGGGGATGTTTGACATGTCTAAAATCACCAGCTGTAAAGAAGATCCTCTTGTTTTTAGTCAAGTATAAAACTAATATTTTTACTTCTTCCGTTTTAAATTTATGTAACACTATTCAATGGACACATAACTTAAAAATAAAAAGACTTTTAGAATGTACTGTTTGAAATAAGACTTAATGAGAATTATGTCAATAAACATAAATGAGAATTTTAAAACTAAATTATTTTTAAAACATTCTTTAAATACGTGTCAAACTAATTAGGACTAGAAGATCCTATACCCATTTGCCTGCATTTTTAGTCCACTTGCACAACACTTCTCTACTAGTTGCGAACTTTCTCTTAAAAATATTGAAGTTGTCTATAGAATATAGAACATATCATGTTAAAGTAAAAACACGAAAATTCATATAGATAACCGATTGAATTATTAAAATTTCTCGAATAACCTATTTTGACTTAAGACTCTAACTGACTGACAGGGAAGGTTGTTTCTTTTTTTTACTCTTCTCTAGAAACTTCCATATTTTTAACTTTTTTTTAATGACTCAAACATGTATACTTCCTCGAGATTAAAGGTAAAGGGTGTTTATCTACATTTTCAAGGGAAGCTTGACGTGGTAATAAACAAAAGCATGTATGTATATAGTATACTTTAAGTTATATTTATTACATCCAAATTTTAAATTTTGTTGTGCTTCACATTGTATATGTCCCAGTGTGGAATCGGATCCAAATTGGGGCCCCAACCTGTTTTGTCTGCGTGGTATATGTGGCCTTTCAGAGCTGTACATTATTGTCCCATGCACTATATATCTTGAGCGGAGTGTTAAACCCCTGCCTTCTCTGTTTATACTTCCTCTCTTTTCTTCCCTATTTTTGGCTTTATACCTCTAATTATATTGTTCTAATTATATGGTAGAAAGATCTACTTCCCGCCAAAAACAACAAAGAAAGTAATCTCTTTTTCTTTGTTCACTCATCAACTTGTTTCTCAAATCATTTGTATCACTGCAACTTTTTCCACACTTAAAAACTTTTTATACAATAATATTGGTCACTATTCACTCACTTCAACCAGTTCTTGATTGTTTTAGTACTCCTTTTTGAGCTTATGATGATTTTTTTTTGTGCTCTTTGAAAAAAATATCTTTTAAATCGAACTGTAACTTTAAGTTTTTGGTATACCATGACGGAAAATTCAGTTCCGGTGATTAAATTCACTCAACACATAGTAACTACAAACAAGCATGTTTTTTCTGAGCATAACGAAAAATCCAATTCAGAGTTACAAAGAGTTGTGAGGATTATACTTACAGATGCCGATGCTACAGATTCTTCCGATGATGAAGGCCGGAATACTGTACGGAGAGTGAAGAGGCACGTGACGGAGATCAACCTTATGCCGTCAACCAAATCGATCGGCGACAGAAAACGAAGATCGGTGTCTCCGGATTCTGACGTCACTCGTCGGAAAAAGTTTAGAGGCGTTCGTCAAAGACCGTGGGGTCGTTGGGCTGCAGAGATTCGGGACCCGACCCGGGGAAAACGGGTGTGGTTGGGTACTTATGACACCCCAGAAGAAGCAGCTGTCGTTTACGATAAAGCTGCAGTTAAGCTCAAAGGTCCTGACGCCGTTACCAATTTTCCGGTATCAACAACGGCGGAGGTAACGGTGACGGTTACGGAAACCGAAACCGAGTCTGTTGCCGACGGTGGAGATAAAAGCGAAAACGATGTCGCTTTGTCACCCACCTCAGTTCTCTGTGACAATGATTTTGCGCCGTTTGACAATCTAGGGTTCTGCGAAGTGGATGCTTTTGGTTTCGACGTTGATTCACTTTTCCGGCTGCCGGATTTTGCTATGACGGAGAAATACTACGGCGATGAATTCGGCGAATTTGACTTTGACGATTTTGCCCTTGAAGCTCGATAG

>SlERF3-4

TTAAATAGAGCCACTTGATTCAATGTGTGATTTATTTAAAATAATTTTAAAAAATTAAGTTTGTTACAAAGGATAAAAATGGTATTTTTTTTTAGTGAATGTAGGAAATATAAATTTTATATGTAAATTTCAAGTGTATAGTTATCTCCTTTTCACCTATTCAAACGTCTTCATATAATAATTCGATTCGTTTATAAAATAAATAAGAACATATTTAATAAAATGAAGTTTGAAGGGTAGAACGAAGTTATCCCTTAGACTCTTTTAAGACCTTAACTCATTTAGTTTATTTAATTGAGTAAGTCTACATTTATATATTTAATAATATATATCTAAATGACATAGATTGAACATTAAATCAAAATTAAATATTTACTTTGAATAAATTGTTTATTTAAAACCGGACTTAATATTTAAATTTTTAATTAAATTAAAGCATTATATATATATATATATATATATATATATATATATATATATATATATTAATTTCAATTCGAATTATTGAATTACCCATAATTTGTTCCTTTTACACACTGTTGGTTGATGTAGAACCAGATTTTGGGAGGAGTATTGATAGAGGATACACATGTTGTTTCAAAACTCATCAGTCCAACTGATAGTGAAAATGTTCGAGAATAATTATGTTATACTTTAAATCACTTGAACACATTATAAAATAAATAAGACTAACACACAGTTTGTCTTCATTCCTTCAATCAAAATCATTATAGTACGAACACAATTAAAGACATTCACAATTAAAAAAATCAATCAAAATCAATTAATAATATTTTTAAAAAATAAATCATTAATAATTCAGCAGAAAAATGACATAACTAATGTACCTTAAGAAGAAAAAAAAACAATAAATTTAAGAAACTATTTATGAGTTCGACGTATATAATATTTTCCAACCTACCAAATATGATGTTTTAAGTAATAAAAATACAAAAAAAAAAAATTAATTAAGCACCCCCATCCACTATATTTGTTGATTGGTAGTAAGTAGAGATTTAAAAGTATTACTTACTATACTATGAATTCCTCTTAAGCAAACACGTCTACACATACGTGGCTTCCTCTTACATGGCTCCGCCGACTTAGCCGATCAAATTTTATTGGCCCCCACCCGAGCCGTAAAATACAAACTGAGCCGCCGATAACGTCACTTAATATAAAAAAATTAAAATAAATCCATTTTATGCGGCGATAAATCCAACCGCCGCCGACAGGCGACAACTCAATTTTTACCGGTTTCCCAGTATTCTTTTTCTTTTTAGTTCACTAGCTGACACGTGTACTGTTTATTTTTTTTAATTTTTTTGGAAAAAAAAAGGAGTAGTACTGAATTTATTATTTTACTTTCGGTGGGGTTTTTTTTTTTTTCACTTCGGTGATAGTCTCATGTAATATTCTAAAAGAAAAAGATAATTTTATATTTAAGGAGACTGAAATTTGAAAATTCTAGTTAAGAATGAAAAAATATTTAAAATGTATTATAATTTTCTTGGTTGGATTTGAACTTTAAAAAATATATTAATATGACTATTTATTTTTAGTATTTTATGTTATGAGGTGATTTGAAAAATAATACTCTCTCCGTTTCAAATAGAATTTTTCTATTTTTTGTTAGTTTGTTTTAAAATGAATGATTTTTTTTTGGGCAACACTTTAACTTTAACTTTTCACGTGGCATGTCTAAAATCACAAAATTAAAAGGTATTTTAATACATTTGACATAACTTTAATTTAAAACTATAAAAATAAAAAAAAATTTAAGTCGTTCTTTTTTAAATAAAGGGAATAGTTAATATTAGAAATAAGTTTGATTAGATATAAATTATTTTGACAGGTTAAAGTAAATAAAAGTGAATAGAAATAGATAGAATTTCAAGAAAACTTTTAAATAATTGTAATTTCCTTTTTTCTTTTATATTTTCGAGATAATTAAGCTTTTGATCTCATAAATATATTTTAATTAATTACTAAAAGTTTCTTTACCTTTTTAATTTTACCTGTTACACCTCCCTGTCTAACATAGTTGATGCTTTAGCTTAAAATGGTAATATTTCATTAGGGATTAGAAATGTTTCAATAGCAAATTTGAAAAAAAAAAAAAGAACTAAATATATTTTGAATTTTGTGAAACATCCACTATTTTAGAATAAACGAACTATTTTTTTTTATTTTGTGAAATTTCAAGTAATTATATTGCATTTTATTTAGGAACTCTAATTATTTAACATAAAATATAAAAATTGTATTATTTGCGTGTTATTTTATCATTCACTCAATAATTTTGAAGGCGATTATATTTTACAAATTATGTGTTTTATTCATTGTAAAATCAGAATTATTACGACGTAATTTTTTATAAGCGTACATAATCAAATCAATTTTATGCGACCATGTCCGCATGATATGTCCTTTATTGTTTTACCGTCTAAACGCTTTAAGATAAAAATTGTTATCATTTGCAATATTTAGTTATCATAATTGTGGTATTTGTTATTTACTTACTTATTTTGAAAGCGTTATATTTTGCAAATTATATATTTTATTTATTGCAGAATCAAAATCATTGTGACGAATTTTTTTTTATAAGTGTACGTAATCAAATCAGTTTTATGCGGCGACGTCTACGGAATATGTACTTTATTGTCACGTACTGTAAAAATAGTTATTATGAATTGTGTTAAGATCGTTTGAAAGTTTAAAATTCCCAAGATAAGCTCTTCACTTACGACATCTAGTTAGCAAAAATGAATTAATGGTCGTTTGTTCGGTAAATTTGAATGTATTTATATCTTACTACTCATGTTTTGTTCAACATCGAAAACATTACGATATAACTTTTAAAAGTGTGCGTAATCAAATCAATTTTATACGGCGATGTCTGTGTGATATTACGTACTTTATTGTCGGATACGTTAAAAGATAGTTATTACGAATTATGTTCGAGATCGTTTGAAAGTCCAAAATCTCCAAGATAAACTCTGTCATTTACGAAATCTAAGCAAAACTGTTAATTGTCATTTATTCGGTAAATTTGAATGCATTTATGCCTTACTAGTCACGTTTTATTTATTGCGGAGCAGAAAACGTTATGATATAACTTTTTAAAAGCGTACGTAATCACATTAGTTTTATACGGCAACGTCTGCATGATATGTACTTTATTATCGTGTATCGCAAAAGATAGTTATTACAAATTATGTTTGAGATCGTTTGAAAGTCCAAAATGTGCAAGATAAACTCTGACAGTTACGACATCTGGTCAAGAAAATTGAGTTAATTGTCATTTATTTGATAAATTTGAATGCATTTATATGAAGCGTAATATAATCACTTTTACCGAAAGATTATATTACGTTTTATATAGATACTCTAATTATATCTTTGAAAGTAAAAATAAAGATTTTTATGCTTCTTTTGAATTATGTTCGAAGTTGTTTGATAATTTGAAATACTCGACATAAAATATATACAATTTACAATATTTAATAACCAAATTAAATTATTTATTTTTAGTATATTTAACTTTACGAATTATATTTTATTTATTACTAAACTAAAAACTTTACGATACAATCTTTTCTACGTATATATAATCAAATCAAATTATACAATGATGTACAAGTGATTTTATTTTTTATCACATAATATAAAGAACAATTATTTTAATTATTATGAGCGCAAACATAAATATATCATTCTTTTCATAATTTAATTCGACCATAGAAAAAAATTATTTTATTTGGTGATTTTCCTCAAAACATAAAAAATCCTCCATATTTTTGAGGTAGGTAAAAAGTAATAAACTCCAAATTAAAAATTACTTTTCCCATTTATTACTTATTCTTTTTTGAGTTCGTGGATACATGCACGTCGCATTAGAAATAAAATAAAAACAACTAGCCCATGCAAGTAAATATATATATATTTGGATTTTTGATTTAATCAGTAAAAAAATTGCTTCGATTATAAATACATGATGTTCATTAAAATAGAAATAGCACCCAAAACACAATTTTCTACATTGATCACTTTTGTTTAACACATAATTATTAAAATTACTTCTAAAGACCCATGCAGTATAAATTGAATAAACAACATTAATTCTTCGTATTTATCACAATCAAATCCAAAATAAACATTATATTAATTTACGTAATAAGGAGTAATGCTAATTTGTGTTACCTACAACTAAAATAACACTAAAATTTAACAAATAAAAGTAAAATTTTAAAGTCGTTAATATATAATACTTCCTCCGTTTCATATAAATTAATTTTAAAGGTGTTTAACACCCTTTAAAAAAAGTAGATTAACATATAAATTGAACTTACTTTTTCATTTTTAACCATTATTAATTATTGTCAAATTTATCATCAAAATAACTAAATATTAATTAATCATCAATTCCAATACTAACTAATGAAAAACTGAAAGAACACACTAAAAGTAATTTTGAGAACTCAATAATAAAATTAATCTACTTAACTAAATATGGAATGAAAAAAGTACTCATTCTTTCCAATTTACAGAGTCAAACAGTTTCGATTTGACCAAAAATTTGCTGATAAAATCTTTATGGTCAAAATATTTATAACATTTATGAAAAATTTACGATTAAAATTCAAAAATTGACATAAAAAATGGGAATGATATTCCTCAATTGTAAAAAATGTATTTGACTAGAAAACCACCAGGACTAATATTAAATATAATTATCCAAATTTAATATACTATTTTTTTTAAAAAAAAAAACATTCCATTTTTAAGAAATAAAATTAAAACAATTATTTATATAATTATATGGTCCCTACATTTTCATCAGTCAAAGACTCCATTGACTGTTATTTTTCTTCCTATATAAAGAGGGCACTTTCCCACCTACGATTTCTCTCAACCGTGCCCTAATTCTCTAATGACTTGTTCAAATTCTATATATATATATATATAATCGTCCATTTCCAGTTAACGGCGAACTGAAAATGGCGCCCAAGCCAAAATGTACGACGGCGGCGACGGCGGCGACGGCGGCGGTGGTGAAGGAGGTGCATTACAGAGGCGTCCGGAAGAGGCCTTGGGGAAGGTACGCGGCGGAGATAAGAGATCCAGGGAAAAAATGCCGTGTTTGGTTAGGTACGTTTGATACCGCTGAAGAAGCAGCGAGAGCGTACGATAAAGCTGCGATTGAGTTTCGAGGTGCGAAAGCGAAGACTAATTTTCAGATGCAGCAGCAGCCGGACGATGTGATTCGGAGTCCGAGTGATACGAGTACGGTTGAATCGTCGTCTGCGGCGGTTGCGGTTGCGAAGGCGCCGGCAATGGTGGAATCGATTCCGTTAGATCTAAGCTTAGGAAGCTCATCATCGGCTGTTGGAATTTTTAGCAGCGGTGTAGGTAAGTTTTTGTTTCAGAATTCTCCGCCGATGTACTATTTTCAAGGAGCTGGAGTGATTCGTAACGGCGGCGGCGGCGGTGGTGGTAGTGGAGATGGAGGTGGTGCTGGTGCTAGTGGTGCCGGCGGGATGATGAAGAGTGAATCCGATTCATCAACTGTGATAGATTTTATGGGTAACAATTTCAAGCCAAAAGCTAAGTTCGACCTTAATCTTCTTCCGACACCGGAAGACATGTGA

>SlERF10-2

ATATAACACGTTTATATATTACTTTCATTTTCAACAACCGCACGCAGGGGCAGAGCTATCCTTAACCTCTTCAACAGAAAACTATATTATTTATATATGGTTAAAATAAATTTATATGTATATATAGTAGATATTGAACCCCTTCGACTATCTATTTCTACAAATTTTGAACCCCTTATTGAAAATTTTAACTCTGTCTCTGACCACATAAGCAAGGCACATCATCCTACCTATGTATAATGGAGAAGCAACCATTATTGTCATTGAAAGTGATATACACTAAACATATATACTATAGTTTGACTATATTTCTAACCATAAAAATCACAACATATATCATAATCTGAAACAAATTTACGCGTTTGTTTAGCTACTCCACTAACTCACAAATAACAAATTGACTTCTAGCTAGTATATATATATATATATATACACACTGATATTTTATCTATTTCATGTTCGATATTTTATATTTGAGTTGATTAAAATTTGATATAAGTCGAGAAATTTGATATTAGGAGTAAAAGACAACTTAATAATGATGATTGGATACTCTGGGGGATTCAAACTCGAGGACTTTGATTGAGACTGAAGGAGTAGTTATCACTCCACCACAATCCTTATCAGTTATATACACGTTAGTTGTTACGTCAAAACAAGGCTGAGCATACTGGTGAACTCAAGTTGGCATCAAGTATCACTTAAGACACCTTAACCGAGCTTTGTTCCTTTTAGACATCTTATGTACAATCCCGTTATATCATTTCAACACTTTTTGTTGATATGACATAGTGAGTGTTATACTCACACTAACTGTGCGTGAGGGCTTTATTTCATTATTTTTAAAAATAAGATTCTTCTTCTCCATCTTTTAGCTATCATTTTCCAAACTTAAACTTTTTCAATAATGAAAAGGTTTAATGATGCAATGATAAATAAGTAAATTTCAGCCTACGTGATTTAGATATGACGGAGAAGACGACTAGTTAGGCATGAGTGAATGTAATGAATTTTGTGCAAGAAAAAATATATATTATTCGAGCTTAAAATGTCACGTGTCATCAAGTTCAATGGTCACTTTTCTACTCAAAAAATATTATTTGAGCATTATCTTTCATATACATATCACATGTCTTTGTTTGATTCATTACTTTAACATATTAACAACAAAAATGTTATACATACTTTATATATTTGAATGATTATCAAAAAATATCAAAACAATAATATCACAAACTCTACATAAGATATCTAAAAAAAATAGCGATAACTTTAAAGACATCGATAAATTCAACCTTAAAATATCAAATATGAAACATGTAACTAATTAAGAAGTGTTTGGTTGTTTACAAAACTTGTGGTTGTCACATTTTTATAAGTAGACAAGAAATAATTCTTTATTTGTGGACACAATTTTGACAGGACCATATATCATTCATTATCTTAATTAAATGTGACATTTTTCGATTGTACGTACAAGGTAATTCAAGTACATATACTAACTTCAAATTTGTTCAACGAAAAGCATTTAAATTTAGTTAAACAGATATTTAATATTGGCACAAATTTTTATGAGTTCGGAATCTAAAAGATATAAAATATTAACAAAAATTTTAAAATGGTATATAAAATTTTATATTCATCAAAATGGCAAAATCACTGGAACAACAACAATTTACTCCACCGAGAAAAGCACGATTTTGCAAAATGTGATATTTTTTTAATTTCTTTTTTAAAAAATCGCTGTCTAGGCAGCAATTTACATTTTTTTTAAAATATCGCTGCCTTTTATTTTTATTTTTATTTTTTAAAAATGGAAATCGCTGTCTAGGTAGCGATTTTCAAAAAAAAAATTAAAAAACAAAAATTGAAAATCGCTGTCTAGGTAGCGATTTGAAGTTTTTTTTTAAAAAAAAATCACATTTTGTAAAATCGCTGCAGTAGCAACGATTTTGCTTTTTTTTGTGGAGTAAATCGTTGTTGTTCCAGCGATTTTACTGTTTTGGTTAAAATAAAATTTTATATATCATTTTGAAATTTTTATTAATATTTTATACCATTTAAGCTTTGGACTCAAATTTTTATCGGTACATAAGTACCTCTCGATTAAGTTATGTACCTAGTAATATTATAATACAAGTACCTCTCGATTAAGTTATGTACCTAGTAATATTATAATACAAATAGTTTTTAACATAATAATTATTAATGTTGATAAAAATATTAACATCTTTACCATTATCAATTAAGTATTATTAAAATTAATATCGCTAGAGACTTTATATAAAAATTTCTAATTACTACTAAAATACATATTTAAACATTAATAATATCTTTTATGCCGCGTGCTTGTGTGAATATTACATTATTCAAAAATTGTCATATTAAACGTTTTATTGACAATTAAAAAAGAGAGAGAACAAGATAATCCAAGCATTTATTGAGAGATTTATCTTTTATTTTAATATAAAAGAGAAAAATGTAATATACAAATTAAATAAAAAAGATTCTTGGAATATTATATTATATAGTTTAATAATGTATCTACAGCAAAAAAAAAATGTTGATAACAGATCGTCAAAGGCAATCATATTGTTAAATATATTCCCAAAAAATAGCACAATAATTTTGTGGAGCCTTTTTCAGCCGCCTCACAAAGTATATAATATAAAAATATTTATACACACATATTAATAAATTAATTATGTGATATGATCACTGAGTATCGCCGAAAAAAGCATTTGTTATATATCTAAAAGAATCATGTGATAATACTGTATATATTATTCAAATGTCAGAATCAGAACAAACATAAATTAATGAGATGGTTAAATATAAATATTTTTGAGTTTAAATTCTGAAAATATTTTTTTTTATTAGAATACTTATGTTCAAAATAAATATTATAATATTTATTAAATTTTAATATAAAGCAAGAAATAAAAAAATATTGAAGCAACATAGAGTGAACGACGACATGTATTTATTATAAGGACTAGCTATATATATATATATTATTTAACTTGAATAATTGTGTTCATATATATATATCTATTTTATATTTAAAAAGCAAAAACTTTATGATCAGTTGAACCCATAATACATTTTCAGAAAAATATATATATATAATATGGTGTGTGTTGCGTGGCACTTAATTAAATTGACAACATGACTTTTTTTTCTACTATATGTTATGTAAGAGAAAATAGTTAACAGTTGACAGCTAGTGATAATGGGTTAATTAATATATAACCTGGATCGCGATGAATGATCGAGATAATTATAATTTTTAAATTTAAAATTTAAATAATTAAAAATATTATTATTAAAAACGAACTTTATGTAATATGTGAATTTAAATTTATCGCTATGAATCTTTTTAAGTCTTATTGGGTCAATATCAGCCGATCGAATCTTTGTAGCACTATAAATTAATACTACATTTTATTTTTGCATTTTCCATTTTTTTTTCCTTTGTTTAGTGAATCATGACAAGTGAGGCGGACCTACGTGAAATTTGTGGATTCGAATAAATTTACTATTTTTTTTATATTTATATTTGTACTTGAATTTTTTTTATATAATCTCATTTAATATCTATACATAATTTTGAAATTTACGTTTATTTATTCTCCAAAAAAAATATATATAAAAAAATAAAAAAATAGAAATGTGTCAACACGCGCTGGAGTAAGCGCGTGTGTGATAAAATAGAATCCTGCTGGTTTTATAGAGAACGATGGGCGGACTTGCCGGCTTAGTTATCCGCAGACACGTCTACTTTTAATTTTTTTCTACAAATACGTCGTGTCAAATTCTCATTGGTTCAAACATGGTGGACCCCACGTCAATGTGGGACCCAAACACCATTAATGAAAATTGTAAGGAAAAAACGGATCCATACCACGCGGCGGAATACCTGAAGCTTAGCCGACAAACCCGGTTCAACCCGGTTTTGTCACCGTTTTCCCCACACTTACCAGTTATCTGCCACCTGTCCATTATCTCTCAAGCTTTCTAGTATCTTCTCCGACAAACATACGTGAAATATACCTCTAAATTCTCGATCAACTTTAGCATTATCTTCAATGTTAAAAAAACCATAAAATATATGTTCTTCACTTTAATTCTTATCCGTTGATTCAATATTTAATAAATGTCGAATCTGTTATGTTGTGGAGCCTGATTAATATTTGATGTACTGTCAAATTTTAGGCTGTACTGTTCATTCTCAATTTATTCTTTGTAACTGTTCATACTCCGAATTATTAATATATACCCCACCGCCGTGAAAGTTTACCCACGGAGTGTTACCACGAACTATTGGTTTCTCTCTTTAGATCTCTCTCGAAAGTTCTTGTGTTCTTCATTCTCAAGTGTGTGTGAATGAATTCGATCCTAACAAAATCCATAAATAAAATTATGACATATGTATATCATTAGTACAAAGGATACACACACGATGACAAAAATATAACGAAGAATATCTCATATTTTTCTTTTTTCCCCCCTTTATTTGTTTGGTCAACTTAAAAAATGAAGTATACCATATGAAGTAAATAAACACTAATAAATATAGTAATTGATTGTAATAAATTACTTACCCTTAAGATTATGCGTTATTGATAATTTTCTTAAATTAAAACTCTGGACTCTGGAGTATAGCAATGTATATATATTATAATAATTATAAGTATCATAAATGGATAGGAAAAGTTGTTTTCTTTGGTAACGAATATATTTTATTAAAAAATAATAAATATATAATATTTGTCATTTGTTTATTTTTTTAAAAAAAAAGTAAAAAAAAAACACAGAGTATGCCGCCATCAAGCACACAAATGTCACGTAACTGACTAGGCCCCACATTTTGTAGAAAAAAGAAGGGCCCCACAAAAAACCCATGGGGGCGGCTATTTCTCATTAGTCAAAATTTTCCTATATAATCTCTCTTCACCCTTTCTCCATTTTCTTCATCTCTGTAACATTTTGAATTAGCTATGGCGCCTAAGGAAAAAATTGGTGCAGTTACAGCTATGGCAATGGTGAATTTAAATGGAATTTCGAAAGAGGTGCATTATAGAGGTGTAAGGAAGAGGCCATGGGGGAGATACGCGGCGGAGATTAGAGATCCTGGGAAAAAAAGTAGGGTTTGGTTAGGTACTTTCGATACTGCGGAGGAGGCGGCTAGAGCTTATGATAACGCTGCTAGAGAATTTCGTGGAGCGAAAGCGAAAACTAATTTTCCGAAATTAGAAATGGAAAAAGAGGAAGATCTGAAATTCGCTGTGAAAAATGAAATCAATCGGAGTCCGAGTCAGACTAGTACTGTGGAGTCATCGAGTCCGGTTATGGTTGATTCATCATCGCCGTTAGATCTAAGTCTCTGTGGATCAATCGGCGGGTTTAATCATCATACGGTTAAGTTCCCGAGCTCCGGTGGAGGTTTTACCGGTTCGGTACAGGCGGTGAATCATATGTACTATATAGAAGCACTTGCACGCGCCGGAGTTATAAAGTTAGAAACAAATCGGAAGAAAACGGTAGATTACCTCGGTGGTGGTGACTCTGATTCATCAACGGTAATTGATTTTATGCGTGTTGACGTGAAATCAACCACCGCCGGTTTAAATCTGGATCTCAACTTTCCTCCACCGGAAAACATGTGA

>SlERF2-6

TATTTTAATAAATAATGCTCTATCATTTTGATAAAATATAAAAAGTTTTAAACCACCTAAAATAACATATATTCATAATTTATTCCACTCCACATTAATTTTAGAAAAACGGCTTTATATTCGATCCGCCCACACAACCTTAAATACGCTCCATTAACCTTAAACCCACCTTGCAGTCCACTTCGGTCCATTATCATCTCGTAATTGTAGTGAGGCAGGAAAACCAAAGCAACACTCAAATGAAGAGTTATATACAGTGAGGTCAGCATTCAGAAAATCATAATCTCTGTGCGGTGTATTGTGAGTTGTTATGTCTGTCTGAGTTCTGAGTGCTGACCCAAAAATCGAGTCAATATATTGACAAAGAGAAACCCACATTTGGCCCATAAAATCCAGCATCTACTCTTCTGTCTCCCCAATTCAGACACCACCTACTCTTTTATTTCACTCATTTAACCAAACGCCTTCTTTTATTCTTTATCCATCCACTCTTTTCTTGACACCACAAATATATTATTCCTCTACTTCCTCATATTAATTTCATATACATTTAACAAAATAATTTTAGATATCCTATATGAAAAAAGAAAAAAATTAAAAATTATTAATGACAAAAAGATATTTTAAAAAATAAAAAATCAATTAAATCGGAAAGAATGTGTTGTTGTGAGCAGTGACCGGAACAAAACATATTTTAGGAAATGCTCAAACGACTACACTTTGAAATGTTGCCATCAATGACCCCTTTTTAGTACTTGCTTCATTTTTTACTTACTATTTTGATTTGACCAACCATTAAAAATATGTATTTTTATATAATATTGTTTTATTAACTAATGCTAAGGATAGAATATAGAAAAACTTTAATTATTTTTTTAATTTTTCAAAAATTACAAGTAAAAATAAAAATATATTTTAAAAATAAATTATAGATAAAATGAAACAAGGGAGTATTTTTTTAAGACGGAATTCAGTTTTAATAAGTACTCCCTAGTTCCTTTTATTTCGTCAATAATAAAAAAACTAGTTAATTAGTAGTCTTAATTATAAAATTCTCATTAATATTATTTAAAGTCGTATAAATATATATTACTTATTTTTAAAAAATTCTTTTTTCTATTAACTCATATAATTAGGACAGAGGGAACAGTATAGTTAATTATTTGTGTTAATGCCGTGTCATGGGAGGTGAGTCTGTGAACCATTAATAGCCTTTTCAGATAACAATACAATATGCAGTATAGTTTTTATTTTTTCAACAGAACATATCAGTAGCTTTATACGCACAAATAATATAATAATAATAATAATTCTGGAACCCTCGAAAAATCGCAATTGTAATCTTTTCGGCGATGCACCAAATATAAAATCTGATCTCCCTACCATAGCTAGCGAATAATACAAGTATCTTACTAGACAAGTTTTACGCAACAATAAATTTATTATCACATTAAATTAAATTTCATTTTATTTTTAGTTCGCACTTGCTAATTAGCATATGTAAGAAAAATTTATCTATTTAAATTCGTATCAGATAAAGCTTTAAAAAAATATTTCTAACACACAATTTTTTAACTAAAAGAAAAATAATCACATCTACTTCGATGGCTGATTTGTACTAAATTGTTAGTAGAGGGGTGGGTAGGGTAGGGGGGCCTTCATTATTATACACATATAACGACAAGGGATATTCCAGTGTATAAAAATATATATATGTGTGTCAGCCTATTGAAAATGATGCAATAAATAAACAATTGAAAATATTAAATCTAAAGAAAGAAAAAATATAAAAAAAAAAGATGGAGGCGCCGCTGAACAGGCGCCGTACAGTGAAGGGTTAAATAGGGTGTAGGGACTCTATAGGGCTGATGTAAGACTCCACCATAGAGAGACAGAGAATAGGACCCTAAATGGGAAAAAGGATAAGTAAGAATATCCACGTGTTACAATATTATTATTACTTCAAATTAATAAAGTTCCCAAATCATTCAAAGATAATCACATTATTTTAATAGTAACAAAAAATGATATTAAATTTTGTTATTATTATCAAAAGATACAATTGTAATATCATCGATGATGAAATTGTTTCTCGCTTAATTGTAATCGAAAAATAAAAATCTGCATTTAGCACATAAGTCGAGAGTAGAATAACACGCCCTACTCATGTGATTGACACGTCCCATCAGCCGGCGCCTCTCTTCCTCTCTATTTTTTAATCTTATTTTATATATGCACTCCACCTACTTATACGACAAATAACATAAAACTCATGTATTCTGGGTTTTTTTATTATATAATTATAATATAATAATATATATTATTGTACTACTATAAGCTATTTCGATATATCAGCATAATATATATATATATATATAAAAAAAAAAAGGTATCTCCACAATTTGACAAATTGGTGGATTTAATTGTCCCACTTTCAGCTTCACATACTTTGGATATATCCACTAACTATACCGTGTGAATAAATAAGTTTCGAGGTATCTAAGTACAGGTATACGATCTATGTGTACTTTAAATTCACTCAATATATTGTTATGTTTTTTTAAAACAATATTGGTTAAAAATATATTGTTATTTTTAAATTTATTTGGATTTTTTTCTCATGTATTTTAATTACGCTATTTTTAAATTAAATTATTGAAACACTAATAATTTGTTTTCTTAAAACAATATTGATTAATTTTGATCAGTTTTTCTATTGTAAATGTCTTTAATTGTGTTTGAATTGTAATAATTTTGATTGAAGGAATGAAGACAAATCATGCTAGTCTCATCTGTTTTCTAATGTGTTCAAATAACTTTTAAGTAAAACACAATTATTCTCAAATATTTTCACCATCAATTAGACTAATGATTCGTGGAACAACATATGTACCCTCTATCAATATCCTTCAAAAATCTGGTTCCACATCAGACAACGGTGTTTGTTTAAACGGAACAAATTATGGGTTCAATGATTCAATAAGAAAATGACGTGATTGAGGTATTTGAGGGGGGAAAACCCAACAATTTGGCCTATTATTATTATTATTATTAATAATAATGATAAAATTATTAATATAAAAAAATGAAAAGATTTAAAATACGCAATTTAATTAGTAAAATGTAACAAATAATATACCTAGTCAAACACTATAATAAGTATCAATTAATATGTTTTAATTTTTTAAAAATAAAGTGCAGCTGCTCCGCTAAAATAGTTTCTGTCTTCAAAATATGTTAGTTTCAATAGTAACTTCAATTGTAGACATAATTTTTTCTAGAATCGAATCAACCCACGTGATTTTGATGCAAGTAAATTATACGTTCGTCAATTTATCATCAGTGGAATTAAACTATAGGCTAAAAGTTGGATTTTAAAAAATATTATCCCCTGTTGTATTTTGACTTTAGGCTATTTCTTAATCTTTATCAATACTAAGAAAGAGATTTTTTAGAAACGTGGGTGGAAATGAAAGATTAATTGAATCCACCATAATTAGAACAAACTTGCAAATTCTTCAAACGACTTCACTAGTGACTTAAAAACCAAAAATAAAATTAAAAAATATTTATTAATTTAAATTATCATATTTTTAATTAAAAGATTTATTTTGTAAATAAGAAAAGTTATATTTTATATAGACATAATACATAAACATGAATCTTAACTTGACTCGGTTGAAAACTATGACATTTAATTTTGGGTGTGCACAAGTAAACACTTAAATTTATATAAAATTAAGCAAATGAACACATTTGTTCTACATAATACTTTACATGGCAATTTATTGTCCTACATGACGTTCTAGATGTATTGTGCCATGTAAACATTTGTATCTACTTATTTTATTTTATACAAATTTAAATATCTATTTGTGCATATTCAACGTTGAAGGACATAAGTATTTACTAAAATCAAATTAAATAATTATGTTTATGTAATATGATTTTATATTATAAATCATAGTCTTAAATACAATATCTTTGTAAATTTTCCTTAAAAGTTGTCACCTGCCTTTGTGAAAAATCAGGAGTGTATATCTTATCAAAGAAATACTACTACTTCATCGAAGTTTGTAGCTTCTCATGGAAGCAATTTAGAAACAAGAGAGAAAGAAAATTTTCTCTTTTTAGGACCGACTTACCATTACCAAAAGTCTATATTTATCATTCCATCTATAGCATGACTATAGAATTTGTAAAATATAAGATATATAATTGTTTATTTGCAAAATAATTTATTGAAATCGCATATGTTTAACTATTACTTCACCTATTTTTAGTTTGATATGTCAAAACAATGGCGTATACCATGCTACCTAGTTTTTTTCTCTTTCGAAATAATACCCCTACTATATATGATTTTTCTTATTTATTTAAGTTTTGCTAAGTAAAATTAATTTTTATTCGCCCCTAAATTTGTAACTTACTACAACAATTAATGCTAATGATTTAACATATGTATTAGTATGAGTAAAGACCCTACTATCCTTCAAAATTCTTTTTATAGCTTTTTAGTATAATAGTGCAGAATATTTTTATAAATAAATATTTTTTGTATTATACATGTTATTTTTAATAGATCAAATCAAACGCATAAGAAAAAAATCTCTCTTTATATATAATTAATATAGACATTATTACTATATTTTATTTAACATTATTTTTATGTAACTTACTAACCGATTCTCCAAAAATAAGTATTAAAATACAGTACCATGAAATGATAAAAGTATTCGTTCATTTTTTAAGAGCCTGTTTATAATTTCGGAACATTTTCCTTCCGACCAAACACATGTTCACCTAAATATTTTGCCTATTTTAGGAGAGAGGTCGTGCCGTCCAACCGACGATTACGGGTGTCTGCGTGGGTCCCTTTTTTATTTTTACTACTGCTGCCCAAATATACTCTTTTTTAGCTCCCAAAACCCCACACAAACACAACACTCATACACTTTCTCTCTCTAAATCCCCCCCTCTCTCTCTCCTCCCCCATTTCCATGGCTTCCACTCGTGAGGGTCACTACAGAGGAGTGAGAAAGCGTCCCTGGGGCCGTTACGCCGCAGAGATTCGCGACCCGTGGAAGAAAACACGTGTCTGGTTAGGCACTTTCGACACTCCAGAAGAAGCGGCACTCGCTTACGACGGCGCCGCCCGTTCACTCCGCGGCGCTAAAGCAAAAACAAACTTCCCCCCTCCTCCGCCGCCGCCGCCGCAGCCTTCTTCCGCTCCTCTTACACTCGATCTCAACCTTCCATCTGATCACCGGTGGACTTCCCCCTCCGGACGTAGGCTCATGATTGGAGAGTTTCTACAAGTAGGTCCGCCGCCGGAACTTAACTTGCCGGTCACCGTCGCTGCGCCGGCGAAGGAAAACGATGTTGGAGCTGCAGCTATGTATTTTGGGATTGTGAGACGTGGGTTGCCTATTGACTTGAACGAACCGCCGCCGTTGTGGATGTGA

>SlERF3-13

ACCCATCCAATTACCCTTGTAACCACGAAAGCTGAAAGAGAGAGCGGAGAAGACAGGGGGAAGTAGAAGATATGCCAGGAATAATGTGCCACCAGCCACTGCAATAACAGCTGGATCAGTTGATGAAATGGTTTCAACTGTGGATGTGGCAATTGGTTTGGCCCCTTCGATCACCTTGGATGTCTGTTGAGCTGCATCAACTACTGTCTGCAACCAAACAATCATGCATCTCTTAAAGTTGAGTAAAGAAAGATGAAACTCAAATCACAACCAACGCCGAAAGTCCAAGATCATCCTATCATGGCCAAAAATGGTATAAAACGAATGAAGTATTGAAAATGTTTTTGTTCTATACCAAATGCTAGTAGCTTTGTCAACCAATATACTTCTTCCTCCTACATATAATTTATATCCATGTTGTTTCTTATAAGTAAATTTCTCTAATCAAAAAGAAATGATGGTTAGCTGGCAAATTATTCACATTGCACATATGAATTTAAATGGACTTAAAGCCTTGTCAAACATGAACTATTATAGCCAAACCTTAGCTGCAGTCATCACTGGCTCAGAGTCCATGCCAGCACTCTGCATTGCCTCTTGGGCCTTCTTAGTGGCATCCGATATGACAGGAGAAGCATTCTTTAAAACTTCCTCTCCTGCCTGCTTTACTAAAGGTAATGCTGCATCAATCCCGGGTTTCACAAATTCAATCACGGGCCCAATCACTCCGCTTACAGTATCAAAGATGTTCGAACCAACCTCTTGCGCTTGATTAACTACCGATTCTACCTGGAAAGAGAAAGCTTCCAATAAATTGCTAAATACCAAATCTCAAGAAACAAGTTTTATTCACAAACACAACACAAAACTTTGTGGAACTGTACCTGCAACTCCCTCCGTCTCAATTTGTTTGTCTAGACAATTTAAATGTCTAGTTTTTTTAGCAACTCTACAATTTCATCTTTTTCACATAACATGTTTAATACCATAACATTAAAAAAAAACATTTTGATACATTTACACATATCTTTAGTGAAAGACCAAGATCATTTTGATACATTCCACATATTTTTACCATAAAATTCATAAACTATGTATCGAAAACTAGGGATGGCAATTGGACGGATTGGGTAAAACTAGGCGGGTGTTTAAAGACAATAAGACCCTACCAAATCCAAAGTTGCTTGGGTCAACTTCAATTTAACAATAACAATACATCAGAACTGCATGCAGTAGAAATTATTTGACCAAAAAAAGATATTTGAAGAAATGGGTTTTAGGGGGAAAATTGTGATGGGTACCTGATTAAGGGAAGAGACGATGTCTTCCTTGGGTAAGTTGATTGCTCTTGCTTCATGGGTCGCAGTGAAAAGTGGAAAGAGGAAAAGTGCAGTGGATGTAGAGAATGATACTGATACAGGTTTTTGAGTAAGTTTAGGAAGACTAAAAACTTTAGGTGGTGATGAAGTAGAAGAAGAAGAAGAAGGAGGAGGAGGGGGCAGAGGAAGAGGTGATTTAGCGGTGGCTGAAGCTCTAAGTGCCATCACTTTTCAGCCTCTTTGCCACTGCTTGCTTGCAGAGTCTTATTGATTATGATGAATTAATGGATTTTGTCCTACTAATTGAAACTATGATGGGCAATGTGTGATGAGAAATTGAGAAACAACTCTTTAACGGCTGAATTTGATGTTCCTCTTCTTTGGTCCTTATTATCCGATGTGGCATTACAAAATACTGATGAAGCTATAAGATGATGACACGTGGATTGTAAGATGAGAAAACGTGGGGCCCTATCCTTCTCACCATTTGCTTAGACAATAGTGCTTTCTTGACGTCAATTTTAATTTTGTTTCAAAAAGTAGACAGTAGTACTGGGTTGTGAGATTAGTAGTACAACAATTTTGTTTTTTCTTAAATCAAAGTGAAATTCATGGTAGTTATTTTGATTAATTCAAGAGATGTATATTATTTTTCATTAATAATAAGTATATTAGATGATTTTATTAACTAAGTCTAGGACCTATGGGAATTGAGACCTTTCATTCTTTAGTCATTCCTTATAACATTGTTGTTTGTTCAACTTTACACAATCGATTTCATTTCATTCCATAAGTGAATTAGGAAAGATAAAAAGGATATGCAAAATAAATTACTTTATGTGACATTATTTTCTTTTTGATTAATACCAAAAAAAATATCATATTTTCTTTTATGTTAAGTATTTAAAGGTATAATTTCTTCTTAATTCTTATTGGTCCCATTTAATTTTCTAATACATTTATGAAGATAGAAAAAAATAATTACTTTTTTAAAGAATAATTTGATAAATATTTTAAAATCTTTATGATTTCTTAAATTCTATATCGAGTTAAATATTATCATATAAAATAAAACAAAGAGAAAGTCTTATTTTCGATAACTTTTAACAATAAATAGTGCTGAAAATCTTACTGCTATAGTACTATTGAGCACACTGTTTCAGAATCCTCGGGGAATATGCCAAAGTACATTACATATATGACCTGAAGTAATCAGCTCCGGCTTAAGCATAATAATAGCTTAAAATAAAAATAAATAGGGTCATAAATATAAAATTTTTAATAATTTTTATATAAATTAAATTTTTTAAGTTTTATCGGATAATATAATTATTCTTATGATTATTTTTTCATAAAATATAATACTCTAAATATGTCAAATTTATTCTAATAATCCCTTTATTTTAATGAAATATTTAATTTTTCTACAAATAGTATTTAATATTTATTAAAAAATACTAACTTATAACCTATTATTATTAAAAATGAGGCGTCTTAAATTTGGTAAAACACATGTTTTTTTAAGTACGATCGAGCCACCCCTGAAAATAGGTCGTTTTGGTCGTGGTGAACAACCGACTCCATAGATAAGATCTTAATGGACATCCGTGTAATTTTTTGGCAAAAATGATCTCGAAGTTCTGAATGACCAAAAAAAATGATGGACTATAACACACGAAAATCAGCAAAATAGGAATTTATCTGCTTTGGGGCTCGTTTAACCTTGAAAATAGATTGTTTTGGTCGTGGTGACTAACCGGCTCCATAGACAAAAATCAGCAAAATAGGGAGTTTATCAGCTCTAGGACTCATTTGACCTTGAAAATGGACCGTTTTGATCGTAGTGACCAACCGACTCCATAGATGAGGTCTTAACGGATGTCCGTGTAAATATTTTGACAAAAATGATATCGAAATGCAAGTAGTATAACATGACATAATGTTGATAAAATTCTCTGCAAAAACAACGTTGAATCCAGATTTTGCTGCCAATACACACTTTGCAAAGACCTTTAGCCACTCACTAAATTTTGCTTCTTTTTTTATTTATTTTTTTGGCTATTATCCGATTAATTATTTATTTATATATTTATTTTTCTGAAAAATAATTGATGGCCTATTTAGCCTTTTATTTATATTTTATTTTAGAGAAGAAAAAATAAAATTCATGGTCAATCCTAATTAGCGAGGTGTATATTTTGGGCTTTAAACTTGCAAGTTCCGTTTGGGAGATCAATGGACCACCAGTGTCTAATCACCTTGGGCCAAAGACCTATTAGCTTTTAGCCCAACACAGTAAACCCACATTGAAACCTAGATCTGGGTTGATTGAGCTCACTAATTAAGCCCAATTCAAAAGTTTAAAGATAAGAGGCAATCAACTCCTTACATGGGACTTCCCGGCGCGAATTCGGACTTGGTCAGGCTCCAAAGTGGGTGTGGGACACCACGTGAAAAACAAAATAAAAAAGAACTTGTATTATTTGCTTATAAAATCCAAAAATATTATTGAATGTTAAAAAGATAAGAAATCTCATTTTCTTCGGAAGTACAAAAAAATTAGCTCTATTTTCTTGGTTGCGCATTTTCTTTTGAAACTACTGGTCATTTTGAGCGCACCTCAACTACTTTACATGTTGATCAGAGATAGCAGATACCCCATGGGGACACTAACCAGAATTTCTGCAGCAATATCTAAACCATAAATATTGGAACAATCAACAATGTGAGGATTATTTTGAACAGTGCATGACAAATTCCACCAACAGTTTTACGTAAGCACCTGTTAAAAGTACAATTAATGAGAATCGGAGTATGAGAGTATTAAAATGAGAATGTTCTAAGAGACTAACAAAGACATACACTATGTGACAACAACAAAATATTAGTTTTTCATCTTTCTGAGAGGAGAGAGAGTGAAGTCTAATAATCAGAAAGAAACACTAAAGAGCTCTATTCATTTTTGGACTTTTGATGAATTAATTTTTGTTTGTTTGTTGGCTCTTGGAAGTACTTAGCCAAGGGCATTAACCTTTTATCATTTTAAGTTAATCCTATAACAAGGCATGACAAATATTATTTGTAGTTTAGCACCTAATGTTTCAACATTTCAGCTCTATTATCAATTTTTCATATCATAGCTAACTTATAATAATTTAAAAATACTATTGTTTTATCCCTAACTACTTGTTCATTTTTACTTAACACATTTATCAAGAAAATAATGATTGATATAATGATTTTACTGTTCTGCCTTGTAAATTGATAGAATTCTAAAATCAATTTTCTCATTCTAGTTTTACCTTGTTCAAAAACATTAACAACAATGATATAATATGAATATTAATAACAACACTTTATGTAAATATCCTTAAATGCTTTAGTAACTAATCTTATTTGGATTTACACTAAGCAATATGTTAAATGAGTTCAAACGGAATGATATGATCAATGAAACCTAATTTATAAATTGAGAGCGCAATTTTTTGTATTTTGGTATCAATGATGACGTCATTTATAACACGTATTTTTATAATAAATATTACTCTTTCAACTACTAAAATATCCAAATAAGGACAATCAACATTCCATTATATACAAAAAAAAACCAATATTAGTTTCCCCAACCACCTAATCATAAAACTAAACCCCTTCTAATCTCCTGTCTAACTAAGTATTAGAATGGAAAACTCACAATCCCCATCAAAGTCCTTAAACAACTCATCCAAAAACATCCAACAAAAAATAAATCCAATTTCCTCAAATGATGGAAAGAGATTTATTGGAGTTAGACAAAGGCCATCAGGCAGATGGGTTGCTGAAATCAAACAAACTTCACAAAAACTCAGGCTGTGGCTTGGTACTTTTGACAAAGCAGAGGAAGCTGCTATGGCTTATGATAGTGCTGCACGTCTTCTTCGTGGGAAAAACGCGAAAACAAATTTCAACAACCATGGAATCTTTAAACCTAATGAAGAGAATTACAGTTTGTTGGAAAAAAATCCAAGATTATATCAACTTATGAAACATGCCATCATGAAGAAATTTGCAGGGAAATATCAAAATAATGAATGTTTAGAAACAGAGGAGGTGGTATTAGTTGAAGAAAGTATAAATAAAGAAGAGATCTGTGCGATTCAATTACAGGGAAGTTCAAAGGTTTATTCATCAGTGATTGTCGCTCCTTCTTTTAGTAATAATGAAAAATCGTATCAACTTTAG
